# Intramolecular Nickel‐Catalyzed Reductive Coupling: Enantioselective Synthesis of Tetrahydroisoquinolines and Related Heterocycles

**DOI:** 10.1002/anie.6219487

**Published:** 2026-09-02

**Authors:** Thilo Bender, Markus Leutzsch, Yannik Sell, Alois Fürstner

**Affiliations:** ^1^ Max‐Planck‐Institut für Kohlenforschung Mülheim/Ruhr Germany

**Keywords:** asymmetric catalysis, 1,3‐dienes, heterocycle synthesis, metallacycles, nickel catalysis, reductive coupling

## Abstract

The *P*‐chiral phosphine AntPhos was found to be the ligand of choice for nickel‐catalyzed intramolecular reductive coupling reactions of 5‐amino substituted 2,4‐dienoates bearing a tethered aldehyde. This approach to heterocyclic compounds has little precedent in the literature; it provides, inter alia, ready access to hydroxylated tetrahydroisoquinoline derivatives comprising an *anti*‐configured amino alcohol motif, which are difficult to make in optically active form otherwise. These compounds qualify for diverse downstream functionalization as exemplified by the swift elaboration of a representative member of the series into a fluorinated heterocyclic building block as well as into the core region of potently cytotoxic phenanthroquinolizidine alkaloids in two differently substituted formats. Detailed NMR studies in combination with crystallographic data allowed the reactive oxanickelacycle intermediates primarily formed to be characterized with regard to the bonding mode of the allylnickel subunit that they comprise. Moreover, insights into conformational aspects pertinent to the understanding of the stereochemical course of this metal‐catalyzed C─C‐bond formation were gained, which leads to the generation of two stereogenic centers at the *vic*‐amino alcohol subunit.

## Graphical abstract

The successful extension of the nickel‐catalyzed asymmetric coupling of polarized 1,3‐dienes with aldehydes to an intramolecular setting opens an efficient new entry into hydroxylated N‐heterocyclic compounds, most notably valuable tetrahydroisoquinolines. The reactive oxanickelacycle intermediates primarily formed were characterized (NMR, X‐ray) and conclusions drawn as to the stereodetermining step of the reductive C─C‐bond formation.

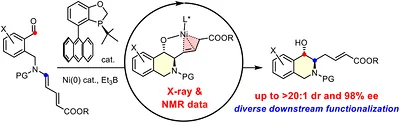

## 1 Introduction

The VAPOL‐derived phosphoramidite **L1** is, so far, the only known ligand capable of reverting the course of nickel‐catalyzed coupling reactions of aldehydes with functionalized 1,3‐dienes while imposing high levels of diastereo‐ as well as enantioselectivity onto the reductive C─C‐bond formation. This favorable profile applies to dienes that are electron‐rich [1, 2], electron‐deficient [3, 4], or have a push–pull character [5, 6, 7, 8]. Carbamate protected 5‐amino‐2,4‐dienoates such as **1a** (R═H) fall into this latter category: under ligand‐free conditions, it is the alkene flanking the ester group that reacts to give the aldol derivative *rac*‐**3**. In the presence of **L1** [9, 10], however, the outcome is completely changed and the *anti*‐configured amino alcohol derivative **2a** formed as virtually the only isomer in good yield and excellent optical purity (91% ee; 99% ee after recrystallization) (Scheme 1) [5].

**SCHEME 1:**
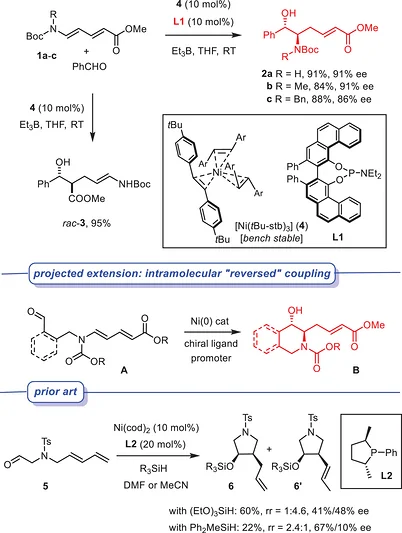
Projected heterocycle synthesis by nickel‐catalyzed reversed reductive coupling in the light of the prior art; Boc = *tert*‐butoxycarbonyl; cod = 1,5‐cyclooctadiene.

Preliminary data suggested that N‐alkylation does not disrupt the course of the reaction as manifested in the conversion of **1b,c** into amino alcohols **2b**,**c**, respectively. Therefore, it seemed possible to extend this type of transformation to an intramolecular setting by incorporation of an N‐substituent bearing a suitably positioned carbonyl group. This conceived approach to N‐heterocyclic ring systems seems all the more compelling if one considers that intramolecular diene‐aldehyde coupling reactions actually marked the very beginnings of this particular type of nickel catalysis, well before the turn of the millennium [11, 12, 13, 14, 15, 16, 17, 18, 19, 20, 21]. Yet, only a single report describing an asymmetric diene cyclization reaction using a chiral ligand seems to have been published, which, in turn, contains only a single heterocyclic example [22, 23]. Specifically, compound **5** was converted into a mixture of the isomeric pyrrolidines **6**/**6′** using Ni(cod)_2_ (10 mol%) in combination with the *C*_2_‐symmetric chiral phosphine **L2** (20 mol%) and R_3_SiH as the terminal reductant; the ee's obtained were encouraging, though certainly insufficient by today's standards. To the best of our knowledge, no further attempts at improving the outcome or generalizing the concept were made in the more than 25 years since the publication of this pioneering work. This lack is striking since other types of unsaturated substrates were studied during this period of time with considerable success [24, 25, 26, 27, 28, 29, 30, 31, 32]; it may well reflect the difficulties encountered in asymmetric nickel‐catalyzed (reductive) coupling reactions of 1,3‐dienes in general, which have been called a “largely unresolved challenge” even in the not too distant past [33, 34, 35, 36]. Only recently has a number of relevant case studies been published that promise a broad scope, all of which are focused on intermolecular processes [37, 38, 39, 40, 41, 42]. The success achieved with **L1** encouraged us to take on the challenge of asymmetric heterocycle synthesis by nickel‐catalyzed intramolecular reductive coupling reactions of dienes of type **A**, not least because the resulting products **B** are valuable and amenable to diversifying downstream functionalization. As will be outlined below, this novel transformation opened entry into optically active tetrahydroisoquinoline derivatives, which are difficult to make otherwise yet arguably valuable building blocks for medicinal chemistry and alkaloid synthesis.

## 2 Results and Discussion

### 2.1 Reaction Optimization

The model compound **7a** was readily prepared by N‐alkylation of **1a** (R═H) with 2‐(bromomethyl)benzaldehyde (for details, see the Supporting Information). When reacted under the conditions previously optimized for the intermolecular coupling of diene **1a** with benzaldehyde, the desired product **8a** was cleanly obtained; very much to our dismay, however, the ee was only 13% and hence far off the excellent optical purity attained in the formation of amino alcohol **2a** [5]. To better understand this unexpected outcome, it was first checked if a racemic background reaction might interfere. Indeed, the cyclization proceeds even in the absence of **L1** to give *rac*‐**8a** in respectable yield. This result shows that the ligand is not needed for coupling at the amine‐substituted end of the dienoate subunit; rather, the connectivity of the substrate controls the regiochemical course, likely because the formation of the six‐membered tetrahydroisoquinoline is kinetically highly favored. In line with this notion, simple phosphines such as Ph_3_P and Cy_3_P in combination with the air‐stable nickel stilbene complex [Ni(*t*Bu‐stb)_3_] (**4**) [43, 44] as precatalyst and Et_3_B as promoter in THF also furnished *rac*‐**8a**, whereas the use of 1,2‐bis(diphenylphosphino)ethane (dppe) brought the conversion almost to a halt. The fact that the dr of the coupling reaction depends on the chosen phosphine, however, suggests that the ligand is on the metal in the stereodetermining step. Therefore, it should be possible to find a suitable chiral ligand capable of imposing a high level of asymmetric induction onto the bond formation; since regiocontrol is of no concern, the search can be largely unrestricted, with a focus on monodentate two‐electron donors.

The sampling of different P‐based ligands showed that the BINOL‐ and SPINOL‐derived phosphoramidites **L3** and **L4** largely suppressed the reaction, whereas TADDOL‐based phosphoramidites and phosphonites gave promising results (Table 1). Even though a number of such ligands was tested in an attempt at optimization, the optical purity of **8a** ranged only between 53% and 77% ee. Gratifyingly, the commercial *P*‐chiral phosphines (*S*)‐BIDIME (**L16**) [45, 46] and (*S*)‐AntPhos (**L17**) [47, 48] paved a way forward [49]. The best result was achieved with [Ni(*t*Bu‐stb)_3_] (**4**, 10 mol%) in combination with (*S*)‐AntPhos (**L17**, 11 mol%) in THF at 0°C; under these conditions, the tetrahydroisoquinoline derivative **8a** was obtained in 88% yield virtually as a single isomer with a dr > 20:1 and an optical purity of 92% ee [50]. The conditions are robust in that the cyclization proceeded equally well on 1 mmol scale (87%, 92% ee). Although Et_3_B is the terminal reductant of choice, Et_2_Zn can also be used (entry 22) [51]; Et_3_SiH, in contrast, proved inadequate as no conversion was observed even upon prolonged stirring (entry 24); a possible reason for this failure is discussed below in the mechanistic section.

**TABLE 1:** Ligand screening and optimization of the reaction conditions.^a^

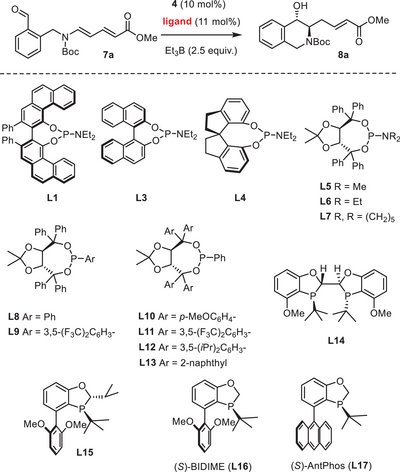						
**Entries**	**Ligands**	**Solvents**	**dr** ^**b**^	**ee** ^**b**^	**Conversion**	**Yield** ^**c**^
1	—	THF	4:1	—	Quant.	50%
2	PPh_3_	THF	4:1	—	Quant.	n. d.
3	PCy_3_	THF	11:1	—	Quant.	80%
4	dppe	THF	n.d.	—	<20%	n.d.
5	**L1**	THF	18:1	13%	Quant.	84%
6	**L3**	THF	n.d.	n.d.	<30%	n.d.
7	**L4**	THF	n.d.	n.d.	<5%	n.d.
8	**L5**	THF	6:1	(−)‐77%	Quant.	n.d.
9	**L6**	THF	5:1	(−)‐77%	Quant.	71%
10	**L6**	Toluene	2:1	(−)‐59%	Quant.	n.d.
11	**L7**	THF	6:1	(−)‐77%	Quant.	n.d.
12	**L8**	THF	3:1	(−)‐72%	Quant.	29%
13	**L9**	THF	n.d.	(−)‐66%	<50%	n.d.
14	**L10**	THF	6:1	(−)‐54%	Quant.	72%
15	**L11**	THF	8:1	(−)‐53%	Quant.	72%
16	**L12**	THF	11:1	(−)‐67%	Quant.	92%
17	**L13**	THF	11:1	(−)‐54%	Quant.	92%
18	**L14**	THF	1:20	2%	50%	40%
19	**L15**	THF	6:1	(−)‐22%	Quant.	n.d.
20	**L16**	THF	8:1	(−)‐82%	Quant.	71%
21	**L17**	THF	>20:1	90%	Quant.	86%
22	**L17** ^d^	THF	>20:1	90%	Quant.	87%
**23**	**L17** ^e^	**THF**	**>20:1**	**92%**	**Quant**.	**88%**
24	**L17** ^f^	THF	—	—	—	—
25	**L17**	1,4‐Dioxane	>20:1	87%	Quant.	83%
26	**L17**	Et_2_O	>20:1	88%	Quant.	82%
27	**L17**	2‐Me‐THF	>20:1	89%	Quant.	85%

^a^ All reactions were performed with Et_3_B (2.5 equiv.) as the promoter at room temperature for 16–48 h, unless stated otherwise.

^b^ The dr and the ee were determined using the crude product.

^c^ Yields of isolated compound.

^d^ Using Et_2_Zn instead of Et_3_B; the reaction was complete within 1 h.

^e^ At 0°C.

^f^ Using Et_3_SiH instead of Et_3_B; no conversion was observed even after 7 d; n.d. = not determined.

Another aspect of the ligand screening deserves explicit mentioning, which can be easily overlooked: the chosen ligands BIDIME (**L16**) and (*S*)‐AntPhos (**L17**) are both (*S*)‐configured at their stereogenic phosphorus center, yet lead to the antipodal products (−)‐**8a** and (+)‐**8a**, respectively; the size of their lateral biaryl entites is obviously a critical determinant. This finding suggests that peripheral interactions involving the extended π‐system of the anthracenyl substituent of **L17** play a crucial role in the enantiodetermining transition state; these non‐covalent interactions do not just govern the level but determine even the sense of asymmetric induction. Increasing the bulk about the P‐atom, as manifested in **L15**, is detrimental; just like dppe, the chelating variant **L14** proved unsuitable as the conversion gets unduly slow and the ee was marginal.

### 2.2 Scope and Limitations

The optimized conditions were then used to explore the scope and limitations of the reaction in detail (Scheme 2). In line with the intermolecular reductive coupling chemistry previously pursued in our laboratory, the carbamate protecting group on the nitrogen linker plays a minor role [5]; thus, the cyclization of the N‐Boc and the N‐Cbz derivatives furnished products **8a** and **8b**, respectively, in similarly good yields and optical purities [52]. Next, a “fluoride scan” was performed to assess whether the positioning of a heteroatom on the benzene ring impacts the outcome and, if so, to which extent. To this end, the four isomeric fluorinated derivatives **8c**–**f** were prepared; while **8c**–**e** were obtained in excellent yield with impeccable ee's, compound **8f** differed in that the desired product was admixed with the deconjugated alkene isomer **8f’** (rr ≈ 4:1, NMR). Together with the notably lower ee's of 55%/74%, this result indicates that a substituent at the *peri*‐position perturbs the otherwise highly ordered transition state and hence marks a certain limitation; a possible explanation is discussed below.

**SCHEME 2:**
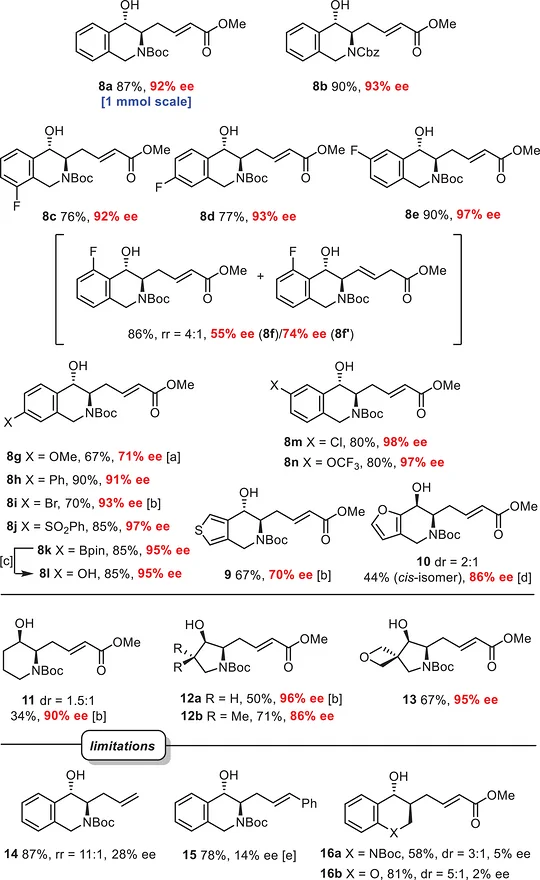
Scope and limitations of the intramolecular reductive coupling reaction with formation of hydroxylated N‐heterocyclic products. Unless stated otherwise, all reactions were performed on 0.1 mmol scale with [Ni(*t*Bu‐stb)_3_] (10 mol%), **L17** (11 mol%) and Et_3_B (2.5 equiv.) in THF at 0°C with reaction times between 16 and 24 h. The resulting products had a dr > 20:1 (unless stated otherwise); the yields refer to analytically pure, isolated compound; [a] 48 h reaction time; [b] using Et_2_Zn; [c] formed by oxidative work‐up of compound **8k** with aq. H_2_O_2_, see text; [d] performed with [Ni(*t*Bu‐stb)_3_] (20 mol%) and **L17** (22 mol%) and Et_3_B (2.5 equiv.) in THF at 60°C; [e] 72 h reaction time; Cbz = benzyloxycarbonyl.

This subtlety notwithstanding, numerous functionalities proved compatible with the reaction conditions. The list includes a chloride and even a bromide substituent that one might consider endangered by the Ni(0) catalyst. Likewise, an electron rich thiophene derivative was compliant despite the pronounced thiophilicity of low‐valent nickel, although the optical purity of product **9** was more modest. This fact seems to align with the general trend that electron deficient aldehydes lead to higher ee's; the comparison of the methoxy derivative **8g** with compounds **8j** and **8k** bearing either a phenylsulfone or a ─Bpin group at the same position of the aromatic ring corroborates this notion. However, the compatibility of the boron substituent, which is of high value for downstream functionalization anyway, offers a convenient workaround: an oxidative quench suffices to unmask the corresponding phenol derivative **8l**, which retains the excellent optical purity of its borylated precursor **8k** and hence surpasses that of the corresponding methoxy derivative **8g** to a notable extent. In all but one case, the diastereoselectivity in favor of an *anti*‐configured amino alcohol subunit was at the limits of detection (dr > 20:1). The only exception was the furan derivative **10**, which was obtained as a mixture of diastereomers, with the *syn*‐isomer predominating; gratifyingly, *cis*‐**10** could be isolated in analytically pure form by routine flash chromatography.

Two important limitations also have to be mentioned: the ester terminus on the diene moiety was found to play a surprisingly critical role; its removal or its formal replacement by a phenyl group resulted in a massive erosion of the optical purity of the resulting products **14** and **15**. Equally, if not even more striking is the fact that compounds **16a**,**b**, in which the heteroatom linker is directly bound to the aromatic ring, were obtained in almost racemic form. A number of other chiral ligands was also tested, but, so far, to no avail (for details, see the Supporting Information) [53]; a reductive asymmetric approach to products of this type has hence to await further study.

The crystalline sulfone derivative **8j** allowed the relative and absolute configuration to be determined by X‐ray diffraction analysis (Figure 1) [54, 55]. The heterocyclic ring adopts a distorted boat‐like conformation, in which the N‐Boc group is equatorially oriented, whereas the C7‐OH substituent and the side chain branching off C6 are both axially disposed. As a result, the dihedral angle between the O‐ and the N‐atom of the *anti*‐configured amino alcohol subunit is −67.4(3)°; accordingly, the H‐atoms at these two stereogenic centers also adopt a gauche orientation (H6‐C6‐C7‐H7 −67.8(3)°), which in turn nicely explains the observed coupling constant (^3^ *J*_H6,H7_ = 2.8 Hz) [56]. The same diagnostic value (2.7–2.8 Hz) appears in the ^1^H NMR spectra of all other products shown in Scheme 2 (except for the major isomer of **10**), as does an equally indicative pattern of NOE effects (for details, see the Supporting Information). Therefore, all of these examples were confidently assigned by analogy.

**FIGURE 1:**
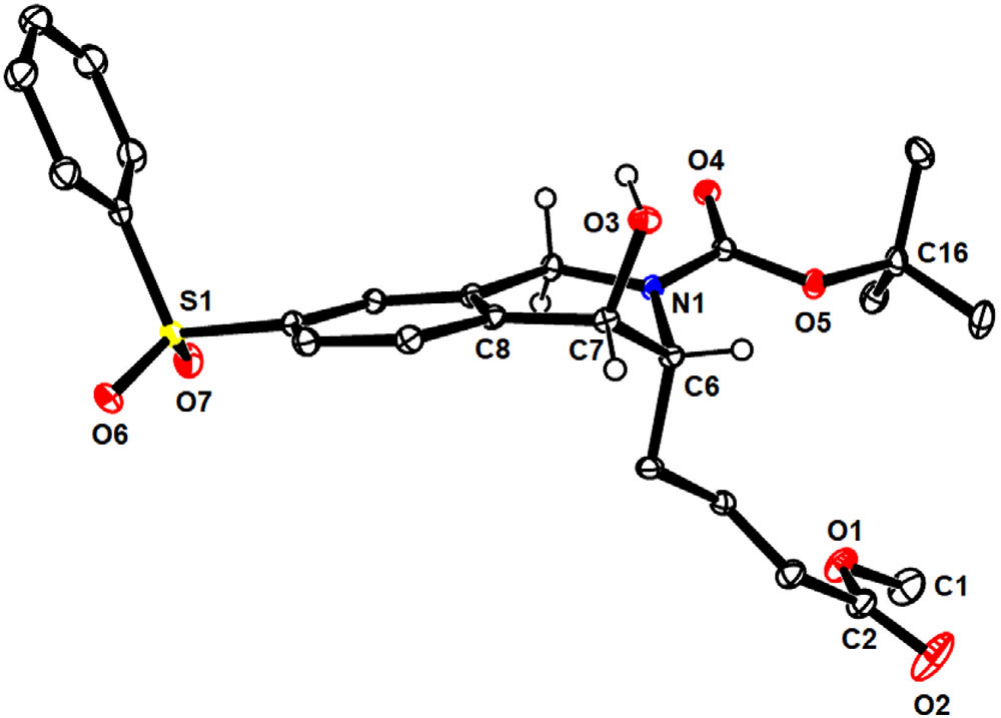
Structure of compound **8j** in the solid state; H‐atoms omitted for clarity except those of the ─OH group and on the heterocyclic ring (crystallographic numbering scheme).

This aspect became genuinely important when the aromatic tether between the diene unit and the aldehyde in the substrates was replaced by an alkyl chain. The relevant protons of the resulting saturated heterocycles **11–13** showed ^3^ *J* coupling constants in the range of 5.5–6.3 Hz, which speaks for a *syn*‐orientation and hence for *cis*‐configured products. The altered stereochemical pattern was confirmed by Mosher ester and NOE analyses [57], which proved that the hydroxylated stereogenic center was (*R*)‐configured, opposite to the respective site in the tetrahydroisoquinolines **8** (for details, see the Supporting Information). The remarkably high optical purity of **11–13**, however, is a noteworthy asset, although the yield was modest in case of the hydroxypiperidine derivative *cis*‐**11** after separation from the diastereomer.

### 2.3 Applications

To assess the significance of the new method, it may suffice to emphasize the enormous importance of tetrahydroisoquinoline derivatives in general; the stereoselective preparation of such compounds has been the subject of innumerous campaigns, covering the entire synthetic spectrum ranging from the use of substrates from the chiral pool to asymmetric catalysis [58, 59, 60, 61]. Yet, flexible, straightforward and high yielding access routes to the particular type of hydroxylated derivatives presented in this study, comprising an *anti*‐configured amino alcohol subunit are largely missing [62, 63, 64]. In addition, the benzylic ─OH group, the endocyclic N‐atom, and the enoate side chain provide ample opportunity for further functionalization. The examples shown in Scheme 3 illustrate this point.

**SCHEME 3:**
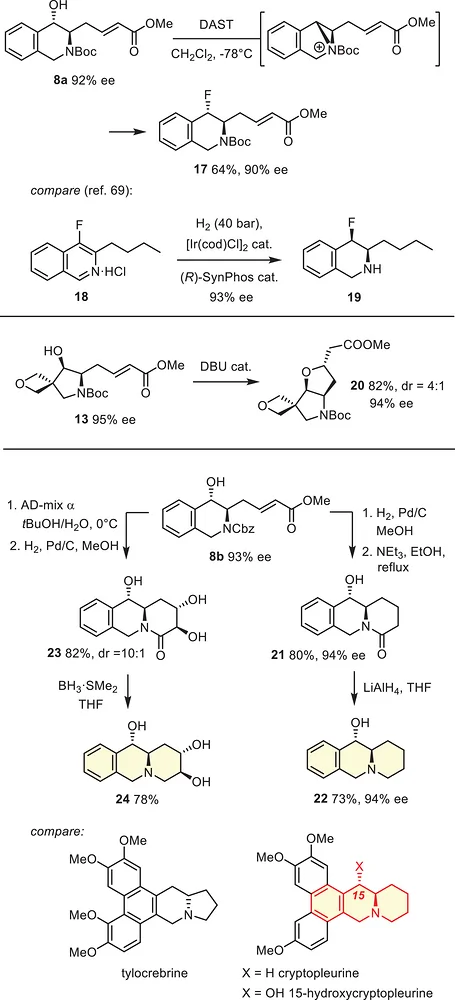
Downstream derivatization.

The ever‐growing importance of fluorinated heterocyclic compounds inspired us to convert product **8a** into the corresponding fluorine derivative **17** [65, 66, 67]. The reaction proceeded smoothly with the aid of DAST at low temperature; not unexpectedly, retention of configuration was observed—without any significant loss in optical purity—certainly due to the intervention of an *N*‐acyl aziridinium species as reactive intermediate formed by intervention of the N‐Boc group [68]. This outcome is complementary to the stereochemical course of iridium‐catalyzed asymmetric hydrogenations of fluorinated isoquinolines previously described in the literature, which furnished *cis*‐configured fluorinated tetrahydroisoquinolines such as **19** by virtue of the *syn*‐delivery of dihydrogen to the ring system of the heteroaromatic precursor **18** [61, 69, 70]. The conversion of pyrrolidine **13** into the corresponding spiro‐tricyclic product **21** by base‐promoted oxa‐Michael addition is perhaps more conventional in purely chemical terms but arguably meets the ever‐growing demand for small curved spirocyclic building blocks [71, 72].

Next, we took advantage of the lateral enoate moiety to annulate an additional ring to the tetrahydroisoquinoline core in two different formats. The N‐Cbz derivative **8b** was chosen because this protecting group is hydrogenolytically cleaved while saturating the double bond over Pd/C as the catalyst; treatment of the resulting crude material with Et_3_N in refluxing EtOH entailed cyclization with formation of lactam **21**, which was then reduced with LiAlH_4_ to furnish product **22** without any loss of optical purity. This compound represents the core region of phenanthroquinolizidine alkaloids, a large and important class of natural products with promising and diverse biological properties; they are particularly renowned for their remarkable antitumor activity [73, 74, 75, 76]. In this context, derivatives hydroxylated at the C15 position such as 15‐hydroxycryptopleurine had evoked particular interest in the past for their exceptional potency in the sub‐nanomolar range [77, 78]. Studies into “downsized” benzoquinolizidinol analogues such as **22**, which the new approach can provide in just a few high‐yielding steps, seem worthwhile, not least because skeletal modifications can alter even the mode of action of alkaloids of this type [79, 80, 81, 82]. Yet another aspect is potentially relevant: clinical trials with the closely related phenanthroindolizidine alkaloid tylocrebrine had to be stopped due to serious side effects on the central nervous system [83]. In the same study, however, it was proposed that more polar analogues should be tested as they might not be able to clear the blood/brain barrier and could therefore allow cyto‐ and CNS‐toxicity to be uncoupled. The preparation of compound **24** from the same precursor **8b** illustrates one way how such decorated variants can be accessed: rather than simply hydrogenating the substrate in the first step, the double bond was subjected to Sharpless asymmetric dihydroxylation [84] prior to the annulation of the third ring and subsequent reduction of the lactam entity of **23** thus formed. Many more late‐stage diversifications taking advantage of the functionality present in compounds of type **8** and their siblings can be envisaged that might empower drug development campaigns.

### 2.4 Mechanistic Aspects

Nickel‐catalyzed reductive coupling reactions of unsaturated compounds with carbonyl derivatives are assumed to follow a common mechanistic manifold (Scheme 4) [85, 86, 87]. Largely based on computational evidence [88, 89], they are thought to commence with the formation of a heteroleptic complex **C**, in which Ni(0) is ligated to the π‐bond of the chosen unsaturated substrate, the aldehyde, as well as one ancillary ligand L (phosphine, phosphoramidite, phosphonite, NHC, etc.). Since aldehydes are weaker donors than such ligands L, an unfavorable preequilibrium with **D** has to be considered, which accounts for a presumably low concentration of the loaded complex **C** in solution; we have recently been able to provide rigorous experimental proof for the intervention and significance of this off‐cycle speciation [4]. In order to enter into productive turnover, it is mandatory that the carbonyl group adopts a “side‐on” *η* ^2^‐coordination mode as shown in **E**, which allows oxidative cyclization with formation of an oxa‐nickelacycle **F** to take place [88, 90]. It is generally believed that Et_3_B (or any other terminal reductant) does not play a role up to this point; the oxophilicity of this promoter then entails a highly exergonic transmetalation resulting in the formation of an alkylnickel species **G** set up for β‐hydride elimination. Finally, the catalytic cycle is closed by reductive elimination of the resulting transient hydride complex **H** with release of the borinate ester **J** as the primary product prior to hydrolytic work up.

**SCHEME 4:**
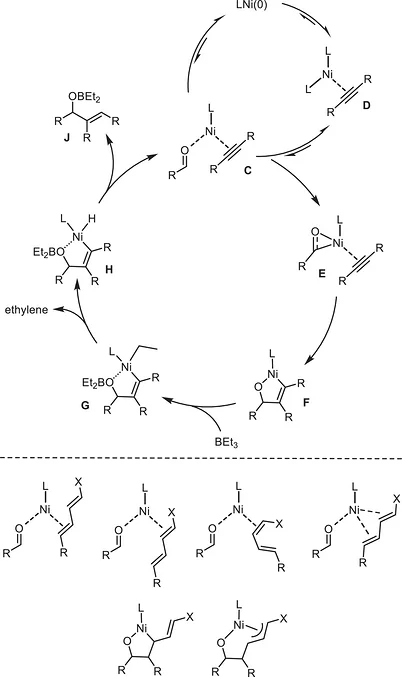
Top: Elementary catalytic cycle of nickel‐catalyzed reductive coupling reactions of unsaturated hydrocarbons with carbonyl derivatives; the mechanism is shown for an alkyne as this type of substrate has been thoroughly studied by computational means [88, 89]; bottom: some additional intricacies when using functionalized dienes as the substrates, cf. Text.

In case of (polarized) 1,3‐dienes as the substrates, however, numerous non‐trivial aspects obscure this basic mechanistic picture. Early studies had suggested that a diene might be *η* ^4^‐bound in a rather unusual *s*‐*trans* conformation, although this assumption has never been substantiated [91]. For most substituted dienes, it is not clear at all which of the two inequivalent yet conjugated double bonds ligates the metal center at the outset and whether any such regiochemical preference in complexation matters for the outcome of the reaction [92]. In any case, the subsequent oxidative cyclization step leads to an allylnickel intermediate as integral part of the ensuing oxametallacycle; a few such complexes have been characterized in the past, but all of them derived from electronically unbiased substrates such as butadiene, isoprene or 2,3‐dimethylbuta‐1,3‐diene [87, 91, 93]. In the present study, however, the conjugated ester group polarizes the emerging allyl nickel moiety, which may or may not alter the bonding mode. Moreover, it cannot be ruled out that the terminal reducing agent plays a much more intricate role than the generally accepted mechanism seems to imply: if its sole function is to transmetalate the oxa‐nickelacycle initially formed, it is difficult to explain why the choice of the promoter is of critical importance for success in certain settings, whereas other, seemingly related, transformations are not particularly sensitive to this parameter [94]. It is conceivable that a strong Lewis acid such as Et_3_B lowers the LUMO of the aldehyde by coordination to the carbonyl group and thus facilitates metallacycle formation; recent studies from our laboratory support this notion [4]. It is unclear if any such assistance is necessary in entropically favorable intramolecular cases such as the current heterocycle synthesis. Finally, the possible intervention of functional groups present in a given substrate has to be considered: the peripheral carbonyl groups of the N‐Boc group and/or the enoate terminus of a substrate such as **7** might compete with the aldehyde and/or the (chiral) ligand for coordination onto the central atom prior to and/or after reductive bond formation.

Although some of these issues have been addressed computationally in related (three‐component‐coupling) reactions involving 1,3‐dienes [40, 42, 95], pertinent experimental studies are exceedingly demanding and notably rare [91]. Despite the challenge, we felt compelled to embark into such an endeavor in the hope of finding at least some answers to the many open questions mentioned above. We conjectured that the intramolecular reaction described in this study might be a good candidate for such an investigation due to the preorganization of the reacting sites, which effectively reduces the entropic penalty.

To this end, we first tried to co‐crystallize a 1:1:1 mixture of the diene substrate **7a**, (*S*)‐AntPhos (**L17**) and [Ni(cod)_2_] [96] in the hope of either isolating a “loaded” complex or the derived metallacycle; however, all attempts met with failure. Since, in the present case, the ligand **L17** is solely needed to impose enantioselectivity onto the reaction but is not required for regiocontrol, we were hoping to simplify the problem by resorting to PCy_3_, which leads to the same outcome except that it delivers product **8a** in racemic form (compare Table 1, entry 3). After considerable experimentation, we managed to isolate single crystals suitable for X‐ray diffraction analysis, which turned out to consist of the corresponding oxanickelacycle **25a** (for the structure, see the Supporting Information) [55]. An even better data set was obtained when the fluorinated derivative **7e** was used (Scheme 5). The structure of the resulting complex **25e** in the solid state (Figure 2) [55] shows that all three C‐atoms of the allyl group are in tight contact with the Ni‐center; actually, the Ni1‐C3 and Ni1‐C5 distances are almost identical. Yet, the C3─C4 bond (1.3909(14)) is notably shorter than the C4─C5 bond (1.4257(14)), speaking against significant delocalization of the π‐system. Rather, the actual bonding mode seems to be an *η* ^3^:*η* ^1^‐hybrid between a canonical π‐allylnickel species and the corresponding σ‐complex; as discussed below, this conclusion is fully congruent with the picture deduced from the NMR data. Complex **25e** gains some nickel C‐enolate character by virtue of the adjacent ester. The effect imposed by the polarizing functional group is not overly strong: it becomes apparent, however, upon comparison with the nickelacyclic complex **26** previously described in the literature, which is derived from butadiene, benzaldehyde, and PCy_3_ [91]: for the lack of the electron withdrawing substituent, the pattern of alternating bond lengths in **26** is actually more pronounced (1.37(2)/1.45(2) Å).

**SCHEME 5:**
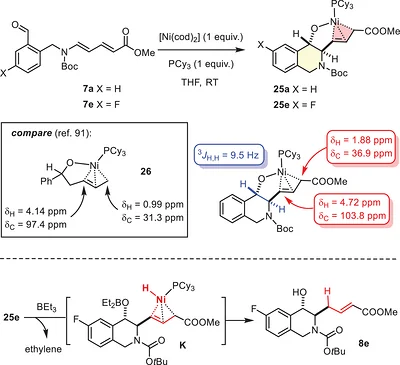
Preparation and characterization of oxanickelacycle intermediates formed upon nickel‐catalyzed intramolecular reductive diene/aldehyde coupling.

**FIGURE 2:**
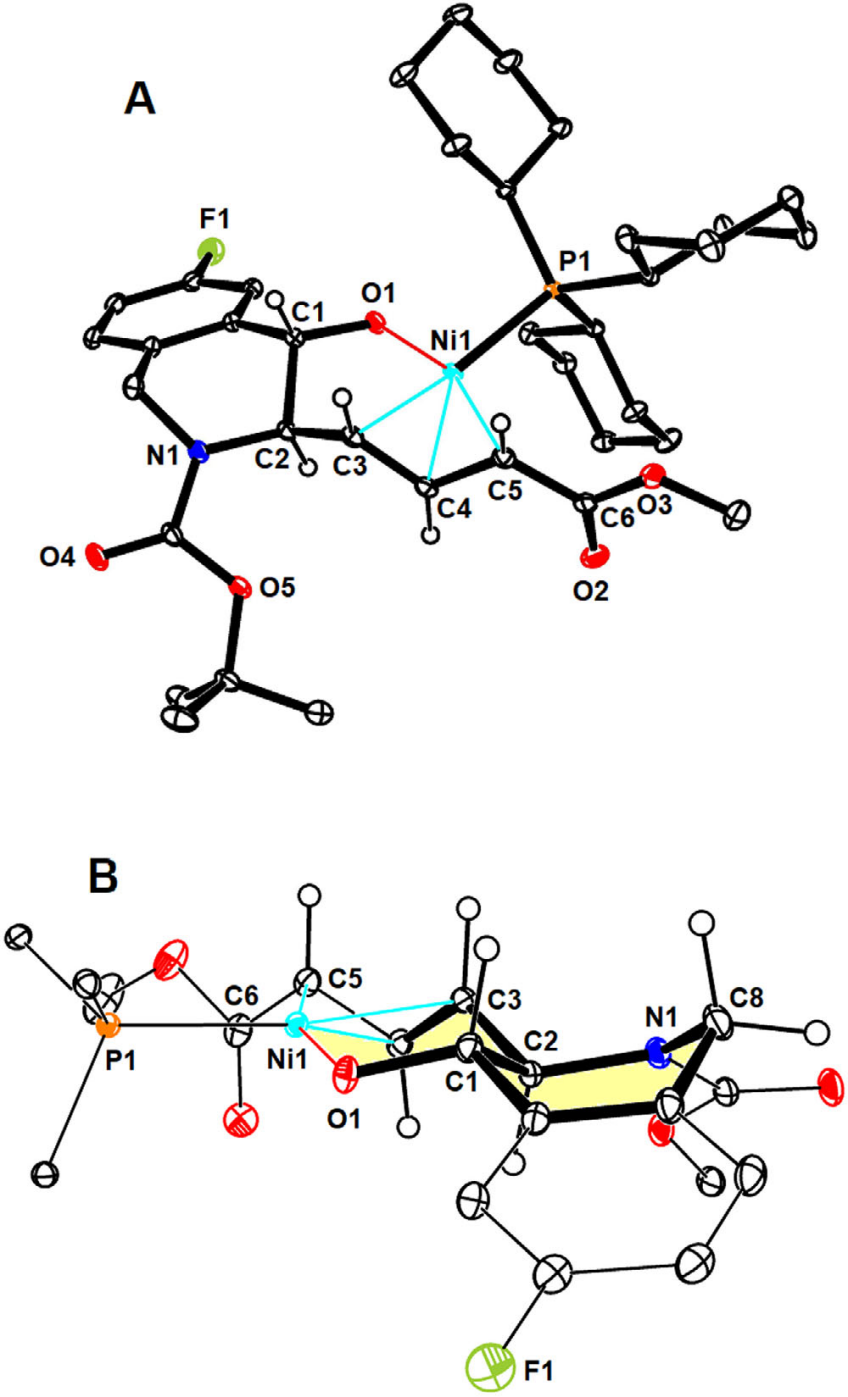
(A) Structure of the oxanickelacycle **25e** in the solid state; solute pentane and H‐atoms not shown for clarity (except for the H‐atoms at the stereogenic centers and the allyl subunit); crystallographic numbering scheme. Selected bond lengths (Å) and angles (°): Ni1‐P1 2.2019(3), Ni1‐O1 1.8581(7), Ni1‐C3 2.0301(9), Ni1‐C4 1.9682(19), Ni1‐C5 2.0314(10), C1─C2 1.5531(13), C3─C4 1.3909(14), C4─C5 1.4257(14), O1‐Ni1‐P1 91.98(2), O1‐Ni1‐C3 87.02(3), C3‐Ni1‐C5 72.63(4), C5‐Ni1‐P1 108.83(3), and O1‐C1‐C2‐N1 −177.53(3). (B) Truncated structure of the same complex showing the puckering of the core region: the chair‐configured oxanickelacycle is annulated to a boat‐configured aza‐heterocycle.

A particularly important aspect concerns the conformation of the core region: similar to what is observed in the structure of the final tetrahydroisoquinoline product **8j** (and, by analogy, **8e**) the heterocyclic ring of **25e** adopts a (distorted) boat conformation. In striking contrast to **8e**, however, the annulated metallacycle forces the C1‐O1 alkoxide entity, the N‐Boc group, as well as the allyl side chain (C3‐C4‐C5) into an equatorial orientation each. We reiterate that the oxygen substituent and the side chain are both axially disposed in the derived final products (compare Figure 1). The clearly visible antiperiplanar array of the heteroatoms O1 and N1 in the metalacyclic intermediate **25e** comes along with an equally antiperiplanar orientation of the protons at the stereogenic centers C1 and C2 (−177.6°), which, however, will adopt a gauche orientation once the tetrahydroisoquinoline is released. This pronounced conformational difference explains why the coupling constant between these two protons is large in the nickelacycle **25e** (^3^ *J* = 9.5 Hz), as discussed below, but small in the derived product **8e** (^3^ *J* = 2.8 Hz). The constraints imposed by the metal center determine the shape of the reactive intermediate in that they evidently override the inherent conformational preference of the organic ligand once freed from the nickel coordination sphere.

As expected, the coordination geometry about the Ni(+2) center itself is close to square planar (if one disregards the contact to the central C‐atom of the allyl group); the observed distortions are attributed to the notably different bond lengths to the C‐, O‐, and P‐atoms directly bound to the central metal. The Ni─O entity resides *cis* to the Ni─C3 bond: as a result, the derived Ni─H species **K** generated upon reaction of the oxametallacycle **25e** with Et_3_B followed by β‐hydride elimination is ideally set up for reductive elimination with the distal site of the allylnickel species to release the conjugated enoate (Scheme 5); it is hence assumed that **8e** is not just the thermodynamically favorable isomer but likely also the kinetic product.

A substituent at the *peri*‐position might interfere with this course of the final step of the proposed catalytic cycle: any group larger than a proton entails a *syn*‐pentane‐type interaction with the C1─O1 bond. If this unfavorable array alters the puckering of the core such that the derived nickel hydride species adopts a different orientation, competing reductive coupling with the C5 position might ensue, resulting in the formation of the deconjugated olefin isomer as observed solely in case of compounds **8f**/**8f′**.

The successful isolation of the two oxanickelacycles **25a**,**e** encouraged us to study the nickel speciation in solution. Indeed, the spectra of a reaction mixture comprised of [Ni(cod)_2_] [96], PCy_3_ and **8a** (1:1:1) in [D_8_]‐THF also contained the metalacyclic intermediate **25a** as the only detectable species (a serious complication in interpreting the data arouse from rotameric exchange of numerous signals caused by the N‐Boc group) (Scheme 5). No signs of the preceding π‐complex with an intact diene ligand, with or without coordination of the aldehyde onto the nickel center, were observed. Reductive C─C‐bond formation is hence virtually quantitative at room temperature within ≤20 min even in the absence of BEt_3_ as the promoter. The most conspicuous spectral features of **25a** concern the large coupling constant of the protons at the stereogenic centers (^3^ *J* = 9.5 Hz), which perfectly corresponds to the conformation of the metalacyclic intermediate adopted in the solid state (see above). Equally diagnostic is the significant shielding of C5/H5 (36.9 ppm/1.88 ppm) relative to C3/H3 (103.8 ppm/4.72 ppm) as the distal end of the allylnickel unit. As has already been deduced from the pertinent bond distances in the crystal structure of **25e**, C3 evidently has a much higher sp^2^ character than C5, which resonates in the aliphatic region. Hence, the π‐system cannot be strongly delocalized and the allylnickel species is best viewed as an *η* ^3^:*η* ^1^ hybrid. Overall, the congruence suggests that the structure in the solid state and in solution must be largely identical. Parenthetically we note that the peripheral functional groups (─COOMe, ─NHC(O)O*t*Bu) are not involved at all in coordination at the product stage. The comparison of the NMR data of **25a** with those of the nickelacycle **26** derived from 1,3‐butadiene described in the literature provides additional information [91]: as already mentioned above, the overall bonding mode of **25** and **26** is similar but the lack of the electron withdrawing ester group and missing C‐enolate character in **26** entails a gradually more pronounced alternation of the bond lengths within the allylnickel entity. The pertinent resonances of **25** and **26** corroborate this conclusion: they fall into the same spectral ranges, but those of **26** are somewhat more shielded (31.3 ppm/0.99 ppm and 97.4 ppm/4.14 ppm, cf. Scheme 5).

After the key metallacyclic intermediate **25a** had been unambiguously identified in this stoichiometric experiment, we went on to measure the kinetic profile of the catalytic reductive cyclization reaction (for details, see the Supporting Information). Interestingly, trace amounts of the nickelacycle **25a** were detected by ^31^P NMR, especially at the beginning of the reaction. The fact that this reactive intermediate is visible implies that the turnover‐limiting step of the catalytic cycle likely comes after its formation; the very low concentration, however, suggests that the rate of formation of **25a** and the rate of its reaction with Et_3_B cannot be much different [51]. Otherwise, clean consumption of substrate **7a** and formation of product **8a** in form of the corresponding borinate ester **8a·BEt_2_** was observed, accompanied by the generation of one equivalent of ethylene. The latter turns out to be non‐innocent; it leads to the formation of the bis‐ethylene complex [(C_2_H_4_)_2_Ni(PCy_3_)], which seems to be a catalyst resting state in solution (Scheme 6). Excess ethylene is hence able to replace the 1,5‐cyclooctadiene (cod) ligands of the precatalyst, even though the strained olefins are arguably the far better π‐acceptor ligands.

**SCHEME 6:**
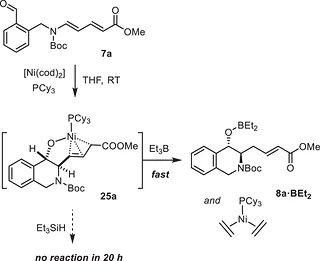
Probing the final step of the catalytic cycle.

The ability to cleanly generate nickelacycle **25a** as the key reactive intermediate also allowed us to study why Et_3_SiH does not qualify as terminal reductant in the present heterocycle formation (see Table 1, entry 24). Virtually no reaction was observed upon addition of one equivalent of this silane to a solution of **25a** in THF at 298 K even after 20 h; evidently, neither σ‐bond metathesis [22] nor oxidative insertion takes place once the metallacycle has formed. We hence conclude that silane‐promoted nickel‐catalyzed reductive coupling reactions between unsaturated substrates and carbonyl derivatives, which have a long and successful history, may proceed through pathways that are fundamentally different from those of the seemingly closely related reactions mediated by Et_3_B or Et_2_Zn. In this context, we would like to recall the original mechanistic proposals made by Mori et al., which did not involve an oxidative cyclization step [11, 22]; rather, these authors considered the formation of nickel hydride species by oxidative insertion of R_3_Si‐H into the Ni(0) center in the first place [97], which then adds to the diene to give an allylnickel species prone to undergo a more classical allylation reaction with the aldehyde partner en route to product.

In a final foray, we returned to the use of (*S*)‐AntPhos (**L17**), even though all efforts to grow crystals of relevant nickel complexes using this chiral ligand had been unsuccessful. A first attempt trying to emulate the stoichiometric NMR study with PCy_3_ outlined above also met with failure; the spectra recorded at room temperature were much too complex and did not allow us to identify any intermediate or product present in the mixture. At −40°C, however, the situation was improved to the extent that all relevant diagnostic signals could be confidently assigned; regions obscured by massive signal overlap still existed but could be deconvoluted by 1D selective TOCSY and various 2D NMR correlation experiments (for details, see the Supporting Information).

The spectra recorded after mixing of the components at −40°C showed the presence of unreacted starting material **7a** (≈80 mol%), residual free ligand **L17** (≈25 mol%), a nickel complex of type [Ni(**L17**)_2_] (≈30 mol%), as well as the oxanickelacycle **27** (≈20 mol%). No signs of the π‐complex involving the diene unit of the substrate were observed even at this temperature, which implies that its concentration is too low for detection. Over the course of 12 h at −40°C, the concentration of **7a** constantly decreased to ≈30 mol% while that of the oxanickelacycle **27** increased, although the mass balance was somewhat off, suggesting that other, as yet unidentified nickel species must also be present in solution.

The oxanickelacycle exists in two diastereomeric forms **27a** and **27b** in solution (dr ≈ 3:1) (Scheme 7). Armed with the information about the constitution and stereostructure of the analogous oxanickelacycles **25** carrying a PCy_3_ ligand, the recorded NMR signals allowed us to conclude that the bonding mode of the allyl subunit of both isomers is very similar to that observed in **25**: C12 carrying the ester substituent, for example, resonates at 40.6 ppm (**27a**)/39.2 ppm (**27b**), which is similar to the shielding of the analogous position observed for **25a** (36.9 ppm). Likewise, the large coupling constant between the protons H8 and H9 on the stereogenic centers (^3^ *J*_H,H_ ≈ 9.8 Hz) indicates that the tetrahydroisoquinoline core region of both isomers adopts the same conformation. The truly distinguishing spectral features relate to the massive shielding of H12 in the major isomer **27a** (*δ*_H_ = −0.44 ppm (versus 1.90 ppm in **27b**)), whereas it is the aromatic proton H6 of the minor isomer **27b** which experiences a similar, though less pronounced, effect (*δ*_H_ = 6.25 ppm (versus 7.26 ppm in **27a**)). These data speak for an atropisomerism caused by the different positioning of the anthracenyl group of **L17** vis‐à‐vis the ligated product [98]: in **27a**, this substituent seems to lay directly above the oxanickelacycle such that H12 (and to a minor extent also H10) experiences the aromatic ring current; in **27b**, in contrast, the anthracenyl group is evidently rotated such that H6 resides in its vicinity. DFT calculations at the B3LYP/D3BJ/def2‐TZVP/CPCM(THF) level of theory were performed to gain a more detailed picture [99]; the crystallographically determined gross structure of complex **25e** was used as the starting point, replacing the PCy_3_ ligand by (*S*)‐AntPhos (**L17**) (Figure 3). Gratifyingly, the optimized minima nicely explain all the experimentally observed spectral features of both atropisomers outlined above, as well as a number of diagnostic ROESY cross peaks between specific atoms of the oxacycle and the chiral ligand.

**SCHEME 7:**
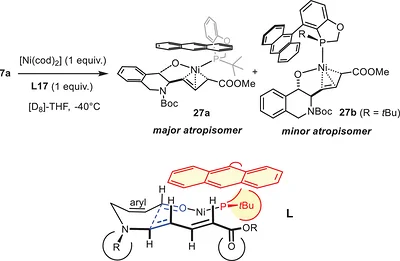
The two atropisomers of the oxanickelacycle formed in the presence of the *P*‐chiral ligand **L17** and putative model of the stereodetermining transition state; for the sake of clarity, the aryl ring of the substrate is only schematically indicated.

**FIGURE 3:**
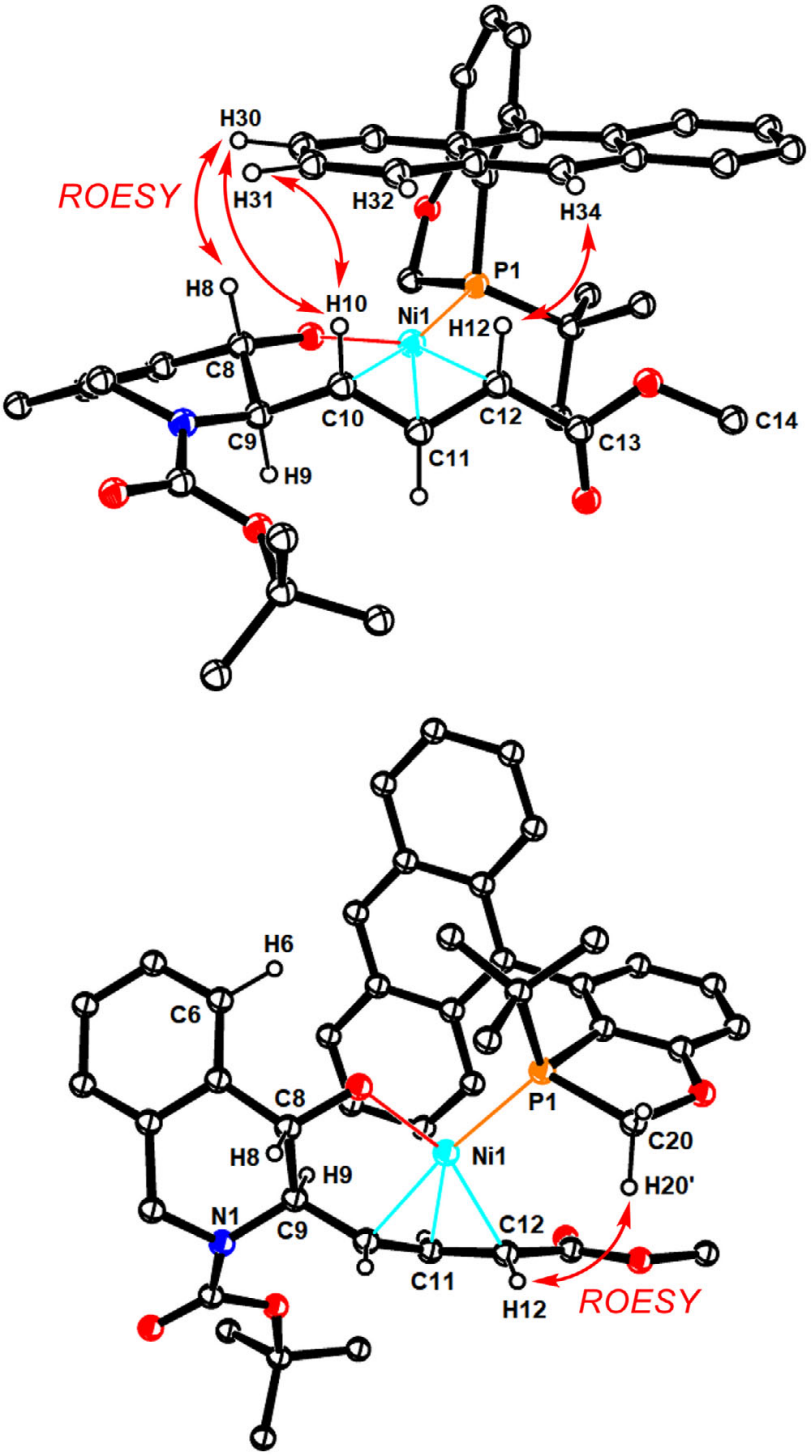
Computed structures of the atropisomeric oxanickelacycle intermediates formed upon intramolecular reductive coupling of **7a** in the presence of the chiral ligand **L17**; top: major isomer (**27a**); H12 and, to a minor degree, H10 reside under the umbrella of the anthracenyl substituent of the ligand and hence experience notable shielding by virtue of the aromatic ring current; bottom: minor isomer (**27b**), in which the aromatic “peri”‐proton H6 is close to the anthracenyl group and hence experiences shielding.

Although the presence of the two interconverting atropisomers makes it most difficult to draw conclusions about the possible structure of the stereodetermining transition state, a model of type **L** may not be entirely off the mark (Scheme 7). Under the umbrella of the anthracene ring system, only the protons as the smallest substituents of the substrate will find space when upward oriented. The ensuing H···π interactions, which are so clearly manifested in the shielding of the pertinent protons in the resulting oxanickelacycle, are thought to explain the observation made in the initial ligand screening: non‐covalent interactions involving the anthracenyl moiety play a major role in that they (co)determine the level as well as the sense of asymmetric induction imposed by the *P*‐chiral ligand **L17**. At the same time, the bulk of the *tert*‐Bu group on phosphorus blocks the backside of the complex, forcing the ligated substrate's backbone to come to the front. The directionality also follows from the positioning of the *t*Bu entity, which leaves enough space for the slim ─COOR group next to it but would clash into the substrate's aryl ring if the opposite orientation were taken. It is tempting to speculate that dipole minimization further stabilizes this array, which might explain why the ester group is important for reaching high ee's (compare products **8** with compounds **14** and **15**).

## 3 Conclusion

Inspired by a series of reductive coupling reactions of various 1,3‐dienes with aldehydes previously developed in our laboratory, which take a reverted regiochemical course yet benefit from high levels of asymmetric induction, an intramolecular variant was developed which opens entry into various types of hydroxylated N‐heterocyclic compounds in optically active form. Commercially available P‐chiral (*S*)‐AntPhos (**L17**) was identified as the ligand of choice, as it affords generally good yields and excellent ee's. The new method gives access to hydroxylated tetrahydroisoquinoline derivatives comprising an *anti*‐configured amino alcohol subunit, which are difficult to make otherwise. Products of this type are valuable building blocks as exemplified, inter alia, by the elaboration of a representative example to the core structure of potently cytotoxic phenanthroquinolizidine alkaloids in differently functionalized formats.

In addition, it has been possible to draw a fairly detailed portrait of the reactive oxanickelacycle intermediates formed as the primary products in the actual oxidative cyclization step. Crystallographic data in combination with NMR spectroscopic evidence allowed the bonding mode of their allylnickel subunit to be analyzed. At the same time, insights into conformational aspects pertinent to the understanding of the stereochemical course of the reaction were gained. In this context, it is of particular note that ligation to the metal center imposes a conformation onto the emerging product that is notably different from the conformation adopted by the tetrahydroisoquinoline after its release from the nickel coordination sphere. Although the P‐chiral AntPhos ligand renders the analysis quite challenging as it leads to atropisomers in solution, some conclusions as to possibly critical interactions in the stereodetermining transition state could be inferred from the acquired data. Finally, light could be shed on the role of the terminal reductant (Et_3_B and Et_2_Zn vs. Et_3_SiH). Further studies into nickel‐catalyzed asymmetric coupling reactions, including additional mechanistic investigations, are ongoing in this laboratory and will be disclosed in due course.

## Author Contributions

**Thilo Bender**: investigation, conceptualization, writing – review and editing, writing – original draft, and data curation. **Markus Leutzsch**: investigation, writing – review and editing, and data curation. **Yannik Sell**: investigation. **Alois Fürstner**: conceptualization, funding acquisition, writing – original draft, writing – review and editing, formal analysis, supervision, and resources.

## Conflicts of Interest

The authors declare no conflicts of interest.

## Supporting information




**Supporting File**: anie74515‐sup‐0001‐SuppMat.pdf.

## Data Availability

The data that supports the findings of this study are available in the Supporting Information of this article.
