# Supplementary material for: Intramolecular Nickel‐Catalyzed Reductive Coupling: Enantioselective Synthesis of Tetrahydroisoquinolines and Related Heterocycles
Source: Angew Chem Int Ed Engl. 2026 Sep 2;65(43):e6219487. doi: 10.1002/anie.6219487 (PMC13676398; doi:10.1002/anie.6219487)
Supplement: Supplementary file 1 [file ANIE-65-e6219487-s001.pdf]

SUPPORTING INFORMATION

Intramolecular Nickel-Catalyzed Reductive Coupling:

Enantioselective Synthesis of Tetrahydroisoquinolines and Related Heterocycles

Thilo Bender, Markus Leutzsch, Yannik Sell, and Alois Fürstner*

Max-Planck-Institut für Kohlenforschung, 45470 Mülheim/Ruhr, Germany

E-mail: fuerstner@kofo.mpg.de

Table of Content

Supporting Crystallographic Information S2
General Information S26
Building Blocks S27
Substrates S35
Ni-Catalyzed Reductive Coupling Reactions S43
    Reaction Optimization S43
    Rotamer Formation S43
Determination of the Relative and Absolute Configuration S71
    The Mosher Ester Derivatives S75
Nickel-Catalyzed Synthesis of Chromanol Derivatives: Preliminary Ligand Screening S78
Applications S80
Kinetic Reaction Profile of a Reductive Cyclization Reaction and Speciation of the Crude Reaction Mixture S86
  Control Experiment with Triethylsilane S90
Preparation and Analysis of the Oxanickelacyclic Complexes S92
NMR-based DFT Models of Rotamers 27a/b S112
NMR Spectra S117
References S205

Supporting Crystallographic Information

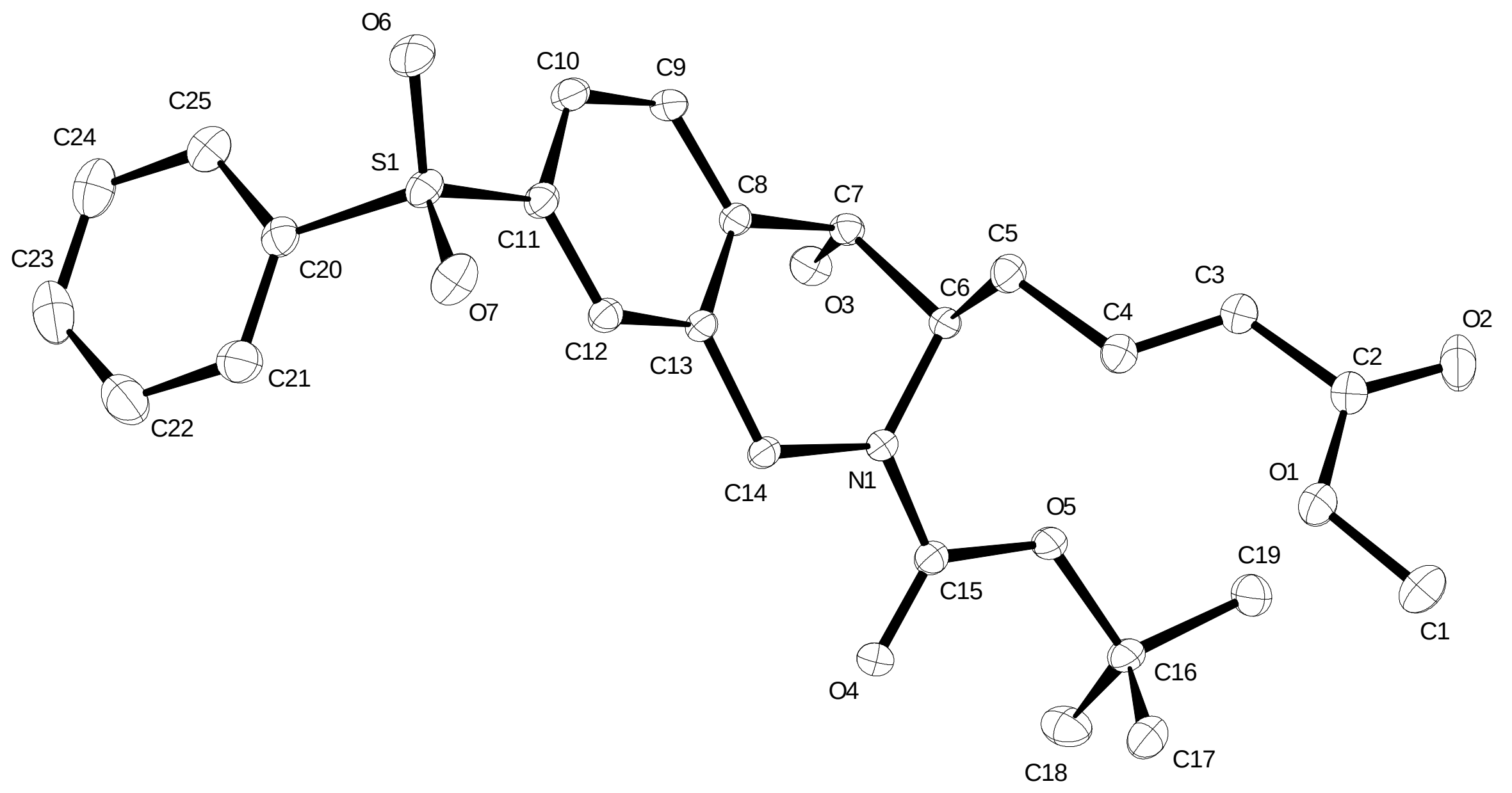

Figure S1. Structure of compound **8j** in the solid state; H-atoms have been removed for clarity.

$C_{25}H_{29}NO_7S$, M_r = 487.55 g·mol^{-1}, colorless plate, crystal size 0.280 x 0.063 x 0.041 mm^3, monoclinic, space group $P2_1$ [4], a = 12.9929(17) Å, b = 6.1545(6) Å, c = 15.306(2) Å, β = 94.309(5)°, V = 1220.5(3) Å^3, T = 100(2) K, Z = 2, D_{calc} = 1.327 g·cm^3, λ = 0.71073 Å, μ(*Mo-K*$_\alpha$) = 0.178 mm^{-1}, Gaussian absorption correction (T_{min} = 0.98, T_{max} = 0.99), Bruker AXS D8-Venture diffractometer with IµS Diamond Mo-anode X-ray source and PHOTON III detector, 1.984 < θ < 30.930°, 178581 measured reflections, 7694 independent reflections, 7334 reflections with $I > 2\sigma(I)$, R_{int} = 0.0684, Absolute structure parameter = -0.009(16). The structure was solved by *SHELXT* and refined by full-matrix least-squares (*SHELXL*) against F^2 to R_1 = 0.0288 [$I > 2\sigma(I)$], wR_2 = 0.0769, 323 parameters. **CCDC-2552556**

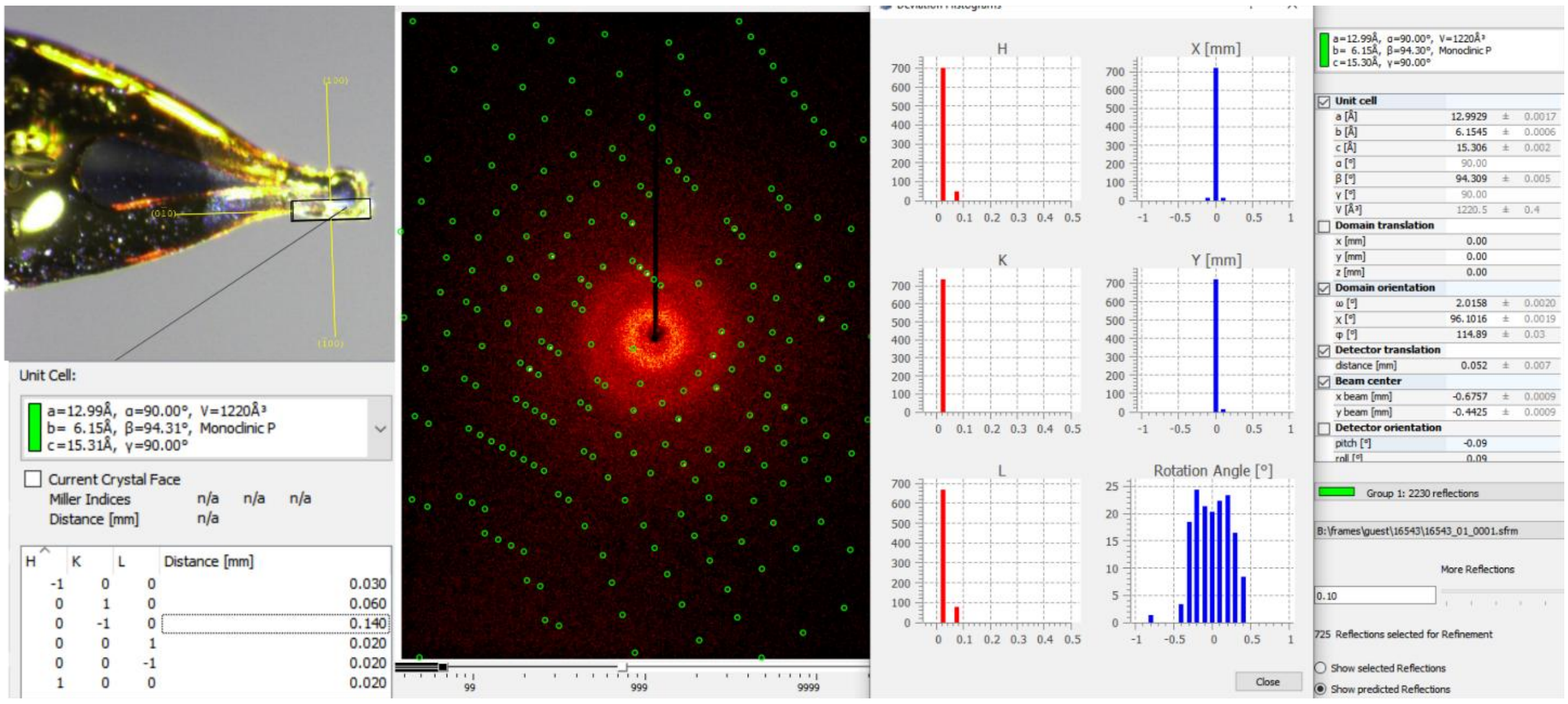

Figure S2. Crystal faces and unit cell determination/refinement of compound **8j**.

INTENSITY STATISTICS FOR DATASET

Resolution	#Data	#Theory	%Complete	Redundancy	Mean I	Mean I/s	Rmerge	Rsigma
Inf - 2.63	136	143	95.1	23.28	88.05	106.90	0.0333	0.0167
2.63 - 1.77	322	322	100.0	31.31	38.40	113.94	0.0297	0.0075
1.77 - 1.41	453	453	100.0	31.76	15.90	82.20	0.0468	0.0097
1.41 - 1.23	445	445	100.0	30.29	15.72	74.37	0.0512	0.0106
1.23 - 1.12	457	457	100.0	28.83	12.95	64.61	0.0595	0.0121
1.12 - 1.03	479	479	100.0	28.88	10.43	57.89	0.0724	0.0142
1.03 - 0.97	502	502	100.0	27.70	7.17	46.09	0.0915	0.0177
0.97 - 0.93	370	370	100.0	27.21	5.75	40.16	0.1066	0.0205
0.93 - 0.88	549	549	100.0	27.53	4.52	35.03	0.1279	0.0243
0.88 - 0.85	415	415	100.0	25.69	4.19	31.80	0.1329	0.0260
0.85 - 0.82	499	499	100.0	22.94	3.76	28.43	0.1432	0.0299
0.82 - 0.79	539	539	100.0	21.45	3.28	25.56	0.1561	0.0337
0.79 - 0.77	392	392	100.0	20.32	3.04	23.42	0.1685	0.0383
0.77 - 0.75	460	460	100.0	17.90	3.04	21.95	0.1615	0.0406
0.75 - 0.73	535	535	100.0	15.44	2.96	20.06	0.1624	0.0469
0.73 - 0.72	260	260	100.0	14.06	2.60	16.93	0.1727	0.0569
0.72 - 0.70	601	604	99.5	11.24	2.49	15.35	0.1785	0.0804
0.70 - 0.69	289	299	96.7	9.22	2.63	14.66	0.1700	0.0928
0.69 - 0.67	645	739	87.3	7.59	2.07	12.75	0.1883	0.0928
0.67 - 0.66	336	378	88.9	6.67	2.23	11.97	0.1893	0.1053
0.66 - 0.65	363	484	75.0	5.29	1.68	10.06	0.2107	0.1207
0.75 - 0.65	3029	3299	91.8	9.75	2.38	14.69	0.1760	0.0804
Inf - 0.65	9047	9324	97.0	20.31	8.05	37.69	0.0695	0.0218

Table S1. Crystal data and structure refinement of compound **8j.**

Identification code	16543	
Empirical formula	$C_{25}H_{29}NO_7S$	
Color	colourless	
Formula weight	487.55 g · mol^{-1}	
Temperature	100(2) K	
Wavelength	0.71073 Å	
Crystal system	MONOCLINIC	
Space group	**P2$_1$, (no. 4)**	
Unit cell dimensions	a = 12.9929(17) Å	α= 90°.
	b = 6.1545(6) Å	β= 94.309(5)°.
	c = 15.306(2) Å	γ = 90°.
Volume	1220.5(3) Å^3	
Z	2	
Density (calculated)	1.327 Mg · m^{-3}	
Absorption coefficient	0.178 mm^{-1}	
F(000)	516 e	
Crystal size	0.280 x 0.063 x 0.041 mm^3	
θ range for data collection	1.984 to 30.930°.	
Index ranges	-18 ≤ h ≤ 18, -8≤ k ≤ 8, -22 ≤ l ≤ 22	
Reflections collected	178581	
Independent reflections	7694 [R_{int} = 0.0684]	
Reflections with I>2σ(I)	7334	
Completeness to θ = 25.242°	99.8 %	
Absorption correction	Gaussian	
Max. and min. transmission	0.99 and 0.98	
Refinement method	Full-matrix least-squares on F^2	
Data / restraints / parameters	7694 / 1 / 323	
Goodness-of-fit on F^2	1.055	
Final R indices [I>2σ(I)]	R_1 = 0.0288	wR^2 = 0.0752
R indices (all data)	R_1 = 0.0315	wR^2 = 0.0769
Absolute structure parameter	-0.009(16)	
Largest diff. peak and hole	0.3 and -0.2 e · Å^{-3}	

Table S2. Bond lengths [Å] and angles [°] of compound **8j**

S(1)-O(6)	1.4441(12)	S(1)-O(7)	1.4456(13)
S(1)-C(11)	1.7678(15)	S(1)-C(20)	1.7716(16)
O(1)-C(1)	1.445(2)	O(1)-C(2)	1.341(2)
O(4)-C(15)	1.2266(17)	O(5)-C(15)	1.3517(16)
O(5)-C(16)	1.4746(16)	O(2)-C(2)	1.210(2)
O(3)-C(7)	1.4277(16)	O(3)-H(3)	0.82(2)
N(1)-C(6)	1.4677(17)	N(1)-C(14)	1.4663(16)
N(1)-C(15)	1.3584(16)	C(2)-C(3)	1.469(2)
C(3)-C(4)	1.335(2)	C(4)-C(5)	1.495(2)
C(5)-C(6)	1.5411(19)	C(6)-C(7)	1.5328(18)
C(6)-H(6)	0.95(2)	C(14)-C(13)	1.5078(18)
C(13)-C(12)	1.3985(18)	C(13)-C(8)	1.4013(18)
C(12)-C(11)	1.3955(18)	C(11)-C(10)	1.401(2)
C(20)-C(25)	1.396(2)	C(20)-C(21)	1.396(2)
C(25)-C(24)	1.396(2)	C(24)-C(23)	1.387(3)
C(23)-C(22)	1.393(3)	C(16)-C(17)	1.522(2)
C(16)-C(18)	1.523(2)	C(16)-C(19)	1.528(2)
C(7)-C(8)	1.5117(19)	C(7)-H(7)	0.95(2)
C(8)-C(9)	1.4027(18)	C(9)-C(10)	1.391(2)
C(21)-C(22)	1.393(2)		
O(6)-S(1)-O(7)	119.67(8)	O(6)-S(1)-C(11)	107.22(7)
O(6)-S(1)-C(20)	107.58(7)	O(7)-S(1)-C(11)	108.24(7)
O(7)-S(1)-C(20)	107.42(8)	C(11)-S(1)-C(20)	105.94(7)
C(2)-O(1)-C(1)	115.59(13)	C(15)-O(5)-C(16)	120.16(11)
C(7)-O(3)-H(3)	108.7(17)	C(14)-N(1)-C(6)	119.88(10)
C(15)-N(1)-C(6)	123.96(11)	C(15)-N(1)-C(14)	115.93(11)
O(1)-C(2)-C(3)	113.46(13)	O(2)-C(2)-O(1)	122.75(15)
O(2)-C(2)-C(3)	123.78(16)	C(4)-C(3)-C(2)	124.23(14)
C(3)-C(4)-C(5)	123.70(14)	C(4)-C(5)-C(6)	111.51(11)
N(1)-C(6)-C(5)	112.74(11)	N(1)-C(6)-C(7)	107.94(11)
N(1)-C(6)-H(6)	106.9(12)	C(5)-C(6)-H(6)	109.2(12)
C(7)-C(6)-C(5)	112.08(11)	C(7)-C(6)-H(6)	107.7(13)
N(1)-C(14)-C(13)	113.05(11)	C(12)-C(13)-C(14)	118.22(12)
C(12)-C(13)-C(8)	119.59(12)	C(8)-C(13)-C(14)	122.19(12)
C(11)-C(12)-C(13)	119.63(13)	C(12)-C(11)-S(1)	118.58(11)
C(12)-C(11)-C(10)	121.48(13)	C(10)-C(11)-S(1)	119.92(10)
C(25)-C(20)-S(1)	119.50(12)	C(25)-C(20)-C(21)	121.22(15)
C(21)-C(20)-S(1)	119.27(12)	C(20)-C(25)-C(24)	118.76(16)
C(23)-C(24)-C(25)	120.15(18)	C(24)-C(23)-C(22)	120.97(17)

O(4)-C(15)-O(5)	125.10(12)	O(4)-C(15)-N(1)	123.46(12)
O(5)-C(15)-N(1)	111.44(11)	O(5)-C(16)-C(17)	110.30(12)
O(5)-C(16)-C(18)	110.19(13)	O(5)-C(16)-C(19)	102.15(11)
C(17)-C(16)-C(18)	113.62(13)	C(17)-C(16)-C(19)	110.17(14)
C(18)-C(16)-C(19)	109.81(13)	O(3)-C(7)-C(6)	109.93(11)
O(3)-C(7)-C(8)	112.94(11)	O(3)-C(7)-H(7)	106.1(12)
C(6)-C(7)-H(7)	108.4(12)	C(8)-C(7)-C(6)	110.20(11)
C(8)-C(7)-H(7)	109.1(12)	C(13)-C(8)-C(7)	118.75(12)
C(13)-C(8)-C(9)	119.75(13)	C(9)-C(8)-C(7)	121.47(12)
C(10)-C(9)-C(8)	121.17(13)	C(9)-C(10)-C(11)	118.26(13)
C(22)-C(21)-C(20)	119.49(16)	C(21)-C(22)-C(23)	119.41(17)

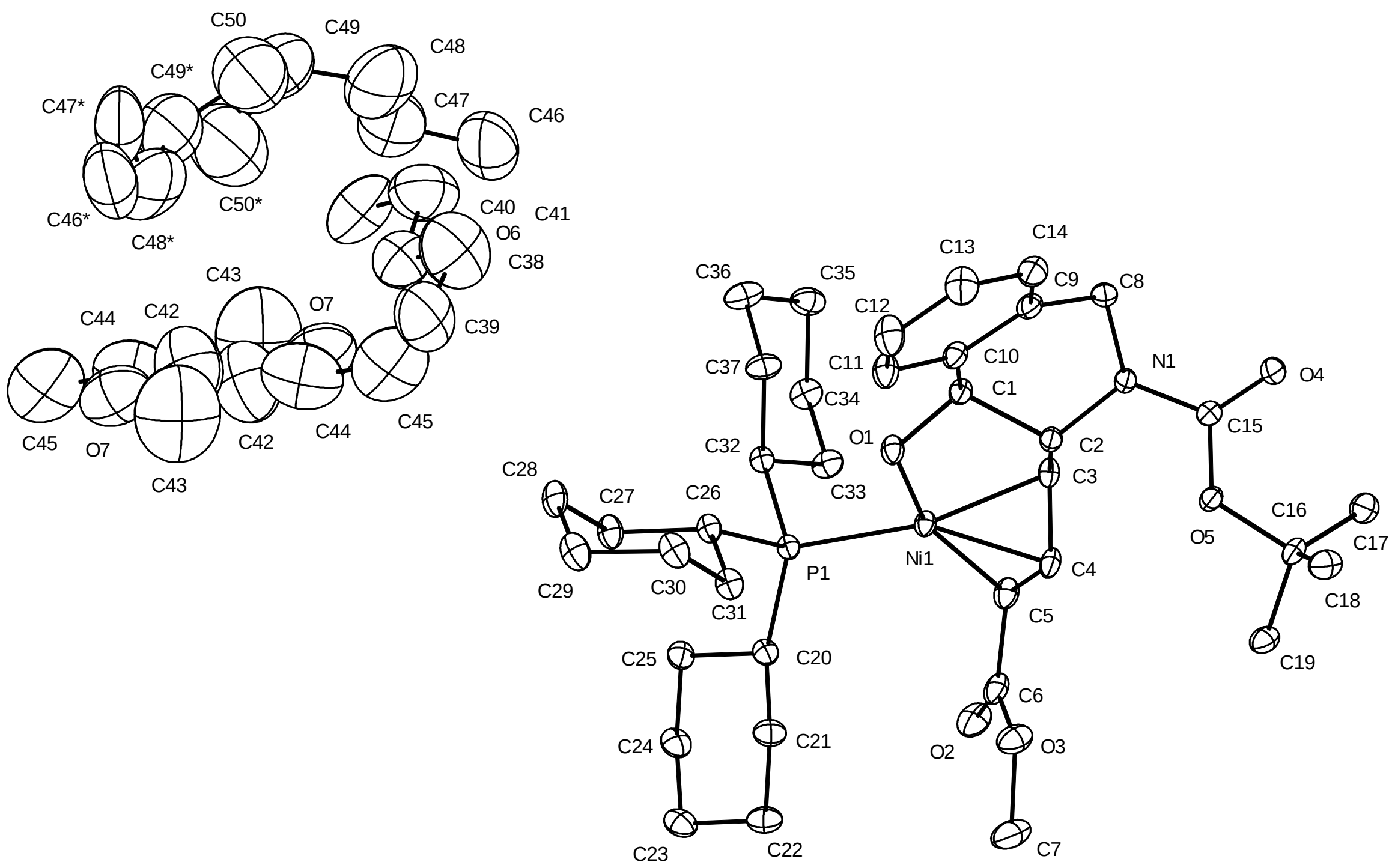

Figure S3. The asymmetric unit of complex **25a**; H-atoms have been removed for clarity.

C_{87} H_{144} N_2 Ni_2 O_{12} P_2, M_r = 1589.39 g·mol^{-1}, orange block, crystal size 0.196 x 0.114 x 0.092 mm^3, monoclinic, space group *C*2/*c* [15], *a* = 39.083(4) Å, *b* = 13.6370(12) Å, *c* = 18.1640(16) Å, β = 115.551(4)°, *V* = 8734.1(14) Å^3, *T* = 100(2) K, *Z* = 4, D_{calc} = 1.209 g·cm^3, λ = 0.71073 Å, μ(*Mo-K*$_\alpha$) = 0.526 mm^{-1}, Gaussian absorption correction ($T_{\min}$ = 0.93764, $T_{\max}$ = 0.97015), Bruker-AXS Kappa Mach3 APEX-II diffractometer with IµS Mo-anode X-ray source, 1.155 < θ < 33.142°, 159662 measured reflections, 16630 independent reflections, 11645 reflections with $I > 2\sigma(I)$, R_{int} = 0.0797. The structure was solved by *SHELXT* and refined by full-matrix least-squares (*SHELXL*) against F^2 to R_1 = 0.0463 [$I > 2\sigma(I)$], wR_2 = 0.1279, 564 parameters. **CCDC-2552555**

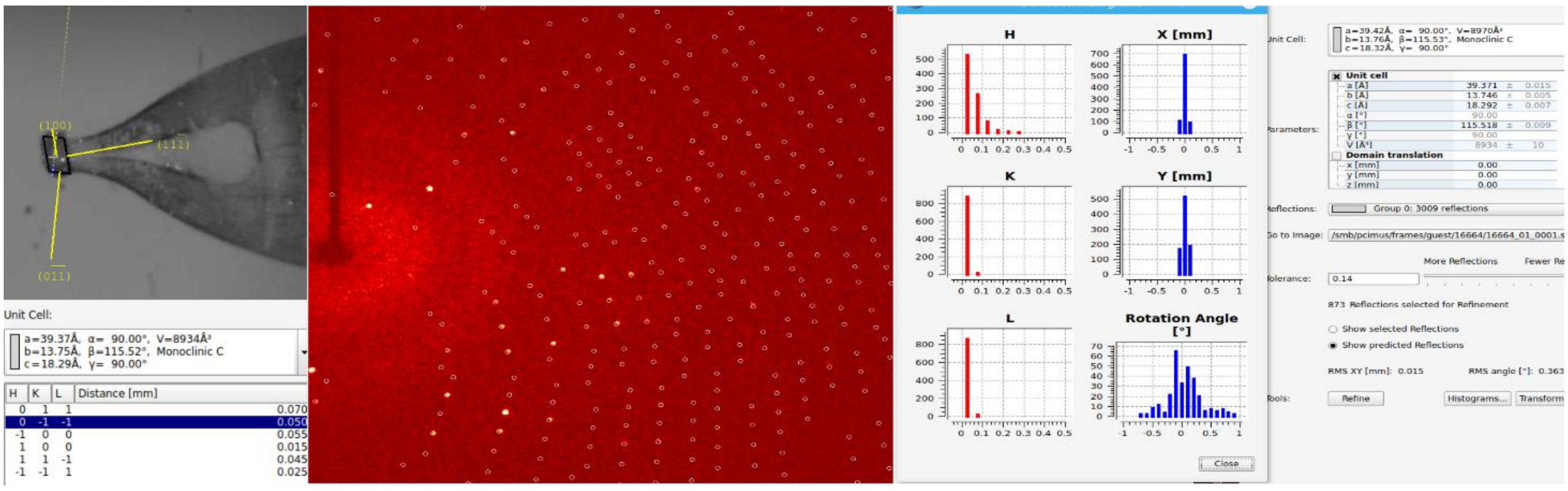

Figure S4. Crystal faces and unit cell determination/refinement of complex **25a**

INTENSITY STATISTICS FOR DATASET

Resolution	#Data	#Theory	%Complete	Redundancy	Mean I	Mean I/s	Rmerge	Rsigma
Inf - 2.61	292	292	100.0	16.81	84.59	69.61	0.0257	0.0093
2.61 - 1.72	685	685	100.0	18.07	43.43	59.51	0.0334	0.0110
1.72 - 1.36	973	973	100.0	18.23	26.63	47.84	0.0472	0.0143
1.36 - 1.18	1020	1020	100.0	17.77	18.15	34.49	0.0628	0.0189
1.18 - 1.07	990	990	100.0	15.32	14.50	27.19	0.0782	0.0259
1.07 - 0.99	1013	1013	100.0	11.27	11.14	18.30	0.0999	0.0397
0.99 - 0.93	1015	1015	100.0	9.38	9.56	14.61	0.1182	0.0518
0.93 - 0.89	824	824	100.0	8.16	8.31	11.53	0.1325	0.0646
0.89 - 0.85	985	985	100.0	7.70	6.95	9.57	0.1577	0.0796
0.85 - 0.81	1190	1190	100.0	7.31	6.06	8.07	0.1807	0.0957
0.81 - 0.78	1074	1074	100.0	6.99	5.41	6.82	0.2053	0.1130
0.78 - 0.76	773	773	100.0	6.72	4.55	5.59	0.2358	0.1375
0.76 - 0.74	922	922	100.0	6.57	4.15	5.25	0.2630	0.1564
0.74 - 0.72	981	983	99.8	6.27	3.63	4.37	0.2965	0.1860
0.72 - 0.70	1112	1112	100.0	6.12	3.35	4.04	0.3149	0.2119
0.70 - 0.68	1245	1248	99.8	5.84	3.22	3.64	0.3412	0.2360
0.68 - 0.67	670	672	99.7	5.66	2.90	3.22	0.3624	0.2734
0.67 - 0.65	1461	1467	99.6	5.49	2.76	2.96	0.3936	0.3001
0.65 - 0.64	821	826	99.4	5.32	2.24	2.36	0.4468	0.3847
0.64 - 0.63	846	853	99.2	5.21	2.11	2.12	0.4761	0.4209
0.63 - 0.62	532	609	87.4	3.22	1.92	1.54	0.4946	0.6315
0.72 - 0.62	6687	6787	98.5	5.42	2.75	3.00	0.3786	0.3041
Inf - 0.62	19424	19526	99.5	8.91	9.79	14.03	0.0800	0.0645

Table S3. Crystal data and structure refinement of complex **25a**

Empirical formula	$C_{87} H_{144} N_2 Ni_2 O_{12} P_2$	
Color	orange	
Formula weight	1589.39 g·mol^{-1}	
Temperature	100(2) K	
Wavelength	0.71073 Å	
Crystal system	Monoclinic	
Space group	*C*2/*c*, (no. 15)	
Unit cell dimensions	a = 39.083(4) Å	α= 90°.
	b = 13.6370(12) Å	β= 115.551(4)°.
	c = 18.1640(16) Å	γ = 90°.
Volume	8734.1(14) Å^3	
Z	4	
Density (calculated)	1.209 Mg·m^{-3}	
Absorption coefficient	0.526 mm^{-1}	
F(000)	3448 e	
Crystal size	0.196 x 0.114 x 0.092 mm^3	
θ range for data collection	1.155 to 33.142°.	
Index ranges	-59 ≤ h ≤ 60, -20≤ k ≤ 20, -27 ≤ l ≤ 27	
Reflections collected	159662	
Independent reflections	16630 [R_{int} = 0.0797]	
Reflections with I>2σ(I)	11645	
Completeness to θ = 25.242°	100.0 %	
Absorption correction	Gaussian	
Max. and min. transmission	0.97015 and 0.93764	
Refinement method	Full-matrix least-squares on F^2	
Data / restraints / parameters	16630 / 259 / 564	
Goodness-of-fit on F^2	1.045	
Final R indices [I>2σ(I)]	R_1 = 0.0463	wR^2 = 0.1125
R indices (all data)	R_1 = 0.0811	wR^2 = 0.1279
Largest diff. peak and hole	0.711 and -0.575 e·Å^{-3}	

Table S4. Bond lengths [Å] and angles [°] of complex **25a**

Ni(1)-P(1)	2.2022(4)	Ni(1)-O(1)	1.8701(11)
Ni(1)-C(3)	2.0210(15)	Ni(1)-C(4)	1.9701(15)
Ni(1)-C(5)	2.0379(16)	P(1)-C(20)	1.8530(15)
P(1)-C(26)	1.8430(16)	P(1)-C(32)	1.8447(16)
O(1)-C(1)	1.3942(18)	O(2)-C(6)	1.208(2)
O(3)-C(6)	1.349(2)	O(3)-C(7)	1.441(2)
O(4)-C(15)	1.2199(18)	O(5)-C(15)	1.3465(18)
O(5)-C(16)	1.4744(18)	N(1)-C(2)	1.4658(19)
N(1)-C(8)	1.476(2)	N(1)-C(15)	1.359(2)
C(1)-H(1)	1.0000	C(1)-C(2)	1.545(2)
C(1)-C(10)	1.509(2)	C(2)-H(2)	1.0000
C(2)-C(3)	1.499(2)	C(3)-H(3)	1.01(2)
C(3)-C(4)	1.390(2)	C(4)-H(4)	0.96(2)
C(4)-C(5)	1.424(2)	C(5)-H(5)	0.91(2)
C(5)-C(6)	1.470(2)	C(7)-H(7A)	0.9800
C(7)-H(7B)	0.9800	C(7)-H(7C)	0.9800
C(8)-H(8A)	0.9900	C(8)-H(8B)	0.9900
C(8)-C(9)	1.506(2)	C(9)-C(10)	1.399(2)
C(9)-C(14)	1.391(2)	C(10)-C(11)	1.384(2)
C(11)-H(11)	0.9500	C(11)-C(12)	1.399(2)
C(12)-H(12)	0.9500	C(12)-C(13)	1.383(3)
C(13)-H(13)	0.9500	C(13)-C(14)	1.391(3)
C(14)-H(14)	0.9500	C(16)-C(17)	1.514(2)
C(16)-C(18)	1.517(2)	C(16)-C(19)	1.521(2)
C(17)-H(17A)	0.9800	C(17)-H(17B)	0.9800
C(17)-H(17C)	0.9800	C(18)-H(18A)	0.9800
C(18)-H(18B)	0.9800	C(18)-H(18C)	0.9800
C(19)-H(19A)	0.9800	C(19)-H(19B)	0.9800
C(19)-H(19C)	0.9800	C(20)-H(20)	1.0000
C(20)-C(21)	1.532(2)	C(20)-C(25)	1.536(2)
C(21)-H(21A)	0.9900	C(21)-H(21B)	0.9900
C(21)-C(22)	1.531(2)	C(22)-H(22A)	0.9900
C(22)-H(22B)	0.9900	C(22)-C(23)	1.522(3)
C(23)-H(23A)	0.9900	C(23)-H(23B)	0.9900
C(23)-C(24)	1.521(2)	C(24)-H(24A)	0.9900
C(24)-H(24B)	0.9900	C(24)-C(25)	1.532(2)
C(25)-H(25A)	0.9900	C(25)-H(25B)	0.9900
C(26)-H(26)	1.0000	C(26)-C(27)	1.528(2)
C(26)-C(31)	1.533(2)	C(27)-H(27A)	0.9900
C(27)-H(27B)	0.9900	C(27)-C(28)	1.529(2)
C(28)-H(28A)	0.9900	C(28)-H(28B)	0.9900
C(28)-C(29)	1.520(3)	C(29)-H(29A)	0.9900

C(29)-H(29B)	0.9900	C(29)-C(30)	1.522(2)
C(30)-H(30A)	0.9900	C(30)-H(30B)	0.9900
C(30)-C(31)	1.527(2)	C(31)-H(31A)	0.9900
C(31)-H(31B)	0.9900	C(32)-H(32)	1.0000
C(32)-C(33)	1.533(2)	C(32)-C(37)	1.531(2)
C(33)-H(33A)	0.9900	C(33)-H(33B)	0.9900
C(33)-C(34)	1.521(2)	C(34)-H(34A)	0.9900
C(34)-H(34B)	0.9900	C(34)-C(35)	1.522(2)
C(35)-H(35A)	0.9900	C(35)-H(35B)	0.9900
C(35)-C(36)	1.521(3)	C(36)-H(36A)	0.9900
C(36)-H(36B)	0.9900	C(36)-C(37)	1.527(2)
C(37)-H(37A)	0.9900	C(37)-H(37B)	0.9900
O(6)-C(38)	1.421(8)	O(6)-C(40)	1.509(8)
C(38)-H(38A)	0.9900	C(38)-H(38B)	0.9900
C(38)-C(39)	1.458(10)	C(39)-H(39A)	0.9800
C(39)-H(39B)	0.9800	C(39)-H(39C)	0.9800
C(39)-C(45)	0.917(14)	C(40)-H(40A)	0.9900
C(40)-H(40B)	0.9900	C(40)-C(41)	1.425(9)
C(40)-C(46)	1.250(12)	C(40)-C(47)	1.262(13)
C(41)-H(41A)	0.9800	C(41)-H(41B)	0.9800
C(41)-H(41C)	0.9800	O(7)-C(42)	1.370(10)
O(7)-C(44)	1.413(7)	C(42)-H(42A)	0.9900
C(42)-H(42B)	0.9900	C(42)-C(43)	1.543(12)
C(43)-H(43A)	0.9800	C(43)-H(43B)	0.9800
C(43)-H(43C)	0.9800	C(44)-H(44A)	0.9900
C(44)-H(44B)	0.9900	C(44)-C(45)	1.463(11)
C(45)-H(45A)	0.9800	C(45)-H(45B)	0.9800
C(45)-H(45C)	0.9800	C(46)-H(46A)	0.9800
C(46)-H(46B)	0.9800	C(46)-H(46C)	0.9800
C(46)-C(47)	1.489(9)	C(47)-H(47A)	0.9900
C(47)-H(47B)	0.9900	C(47)-C(48)	1.434(9)
C(48)-H(48A)	0.9900	C(48)-H(48B)	0.9900
C(48)-C(49)	1.556(10)	C(49)-H(49A)	0.9900
C(49)-H(49B)	0.9900	C(49)-C(50)	1.463(10)
C(50)-H(50A)	0.9800	C(50)-H(50B)	0.9800
C(50)-H(50C)	0.9800		

Ni(1)-P(1)	2.2022(4)	Ni(1)-O(1)	1.8701(11)
Ni(1)-C(3)	2.0210(15)	Ni(1)-C(4)	1.9701(15)
Ni(1)-C(5)	2.0379(16)	P(1)-C(20)	1.8530(15)
P(1)-C(26)	1.8430(16)	P(1)-C(32)	1.8447(16)
O(1)-C(1)	1.3942(18)	O(2)-C(6)	1.208(2)
O(3)-C(6)	1.349(2)	O(3)-C(7)	1.441(2)
O(4)-C(15)	1.2199(18)	O(5)-C(15)	1.3465(18)

O(5)-C(16)	1.4744(18)	N(1)-C(2)	1.4658(19)
N(1)-C(8)	1.476(2)	N(1)-C(15)	1.359(2)
C(1)-H(1)	1.0000	C(1)-C(2)	1.545(2)
C(1)-C(10)	1.509(2)	C(2)-H(2)	1.0000
C(2)-C(3)	1.499(2)	C(3)-H(3)	1.01(2)
C(3)-C(4)	1.390(2)	C(4)-H(4)	0.96(2)
C(4)-C(5)	1.424(2)	C(5)-H(5)	0.91(2)
C(5)-C(6)	1.470(2)	C(7)-H(7A)	0.9800
C(7)-H(7B)	0.9800	C(7)-H(7C)	0.9800
C(8)-H(8A)	0.9900	C(8)-H(8B)	0.9900
C(8)-C(9)	1.506(2)	C(9)-C(10)	1.399(2)
C(9)-C(14)	1.391(2)	C(10)-C(11)	1.384(2)
C(11)-H(11)	0.9500	C(11)-C(12)	1.399(2)
C(12)-H(12)	0.9500	C(12)-C(13)	1.383(3)
C(13)-H(13)	0.9500	C(13)-C(14)	1.391(3)
C(14)-H(14)	0.9500	C(16)-C(17)	1.514(2)
C(16)-C(18)	1.517(2)	C(16)-C(19)	1.521(2)
C(17)-H(17A)	0.9800	C(17)-H(17B)	0.9800
C(17)-H(17C)	0.9800	C(18)-H(18A)	0.9800
C(18)-H(18B)	0.9800	C(18)-H(18C)	0.9800
C(19)-H(19A)	0.9800	C(19)-H(19B)	0.9800
C(19)-H(19C)	0.9800	C(20)-H(20)	1.0000
C(20)-C(21)	1.532(2)	C(20)-C(25)	1.536(2)
C(21)-H(21A)	0.9900	C(21)-H(21B)	0.9900
C(21)-C(22)	1.531(2)	C(22)-H(22A)	0.9900
C(22)-H(22B)	0.9900	C(22)-C(23)	1.522(3)
C(23)-H(23A)	0.9900	C(23)-H(23B)	0.9900
C(23)-C(24)	1.521(2)	C(24)-H(24A)	0.9900
C(24)-H(24B)	0.9900	C(24)-C(25)	1.532(2)
C(25)-H(25A)	0.9900	C(25)-H(25B)	0.9900
C(26)-H(26)	1.0000	C(26)-C(27)	1.528(2)
C(26)-C(31)	1.533(2)	C(27)-H(27A)	0.9900
C(27)-H(27B)	0.9900	C(27)-C(28)	1.529(2)
C(28)-H(28A)	0.9900	C(28)-H(28B)	0.9900
C(28)-C(29)	1.520(3)	C(29)-H(29A)	0.9900
C(29)-H(29B)	0.9900	C(29)-C(30)	1.522(2)
C(30)-H(30A)	0.9900	C(30)-H(30B)	0.9900
C(30)-C(31)	1.527(2)	C(31)-H(31A)	0.9900
C(31)-H(31B)	0.9900	C(32)-H(32)	1.0000
C(32)-C(33)	1.533(2)	C(32)-C(37)	1.531(2)
C(33)-H(33A)	0.9900	C(33)-H(33B)	0.9900
C(33)-C(34)	1.521(2)	C(34)-H(34A)	0.9900
C(34)-H(34B)	0.9900	C(34)-C(35)	1.522(2)
C(35)-H(35A)	0.9900	C(35)-H(35B)	0.9900

C(35)-C(36)	1.521(3)	C(36)-H(36A)	0.9900
C(36)-H(36B)	0.9900	C(36)-C(37)	1.527(2)
C(37)-H(37A)	0.9900	C(37)-H(37B)	0.9900
O(6)-C(38)	1.421(8)	O(6)-C(40)	1.509(8)
C(38)-H(38A)	0.9900	C(38)-H(38B)	0.9900
C(38)-C(39)	1.458(10)	C(39)-H(39A)	0.9800
C(39)-H(39B)	0.9800	C(39)-H(39C)	0.9800
C(39)-C(45)	0.917(14)	C(40)-H(40A)	0.9900
C(40)-H(40B)	0.9900	C(40)-C(41)	1.425(9)
C(40)-C(46)	1.250(12)	C(40)-C(47)	1.262(13)
C(41)-H(41A)	0.9800	C(41)-H(41B)	0.9800
C(41)-H(41C)	0.9800	O(7)-C(42)	1.370(10)
O(7)-C(44)	1.413(7)	C(42)-H(42A)	0.9900
C(42)-H(42B)	0.9900	C(42)-C(43)	1.543(12)
C(43)-H(43A)	0.9800	C(43)-H(43B)	0.9800
C(43)-H(43C)	0.9800	C(44)-H(44A)	0.9900
C(44)-H(44B)	0.9900	C(44)-C(45)	1.463(11)
C(45)-H(45A)	0.9800	C(45)-H(45B)	0.9800
C(45)-H(45C)	0.9800	C(46)-H(46A)	0.9800
C(46)-H(46B)	0.9800	C(46)-H(46C)	0.9800
C(46)-C(47)	1.489(9)	C(47)-H(47A)	0.9900
C(47)-H(47B)	0.9900	C(47)-C(48)	1.434(9)
C(48)-H(48A)	0.9900	C(48)-H(48B)	0.9900
C(48)-C(49)	1.556(10)	C(49)-H(49A)	0.9900
C(49)-H(49B)	0.9900	C(49)-C(50)	1.463(10)
C(50)-H(50A)	0.9800	C(50)-H(50B)	0.9800
C(50)-H(50C)	0.9800		

O(1)-Ni(1)-P(1)	95.80(4)	O(1)-Ni(1)-C(3)	86.19(6)
O(1)-Ni(1)-C(4)	117.22(6)	O(1)-Ni(1)-C(5)	158.37(6)
C(3)-Ni(1)-P(1)	162.10(5)	C(3)-Ni(1)-C(5)	73.20(6)
C(4)-Ni(1)-P(1)	145.72(5)	C(4)-Ni(1)-C(3)	40.74(7)
C(4)-Ni(1)-C(5)	41.58(6)	C(5)-Ni(1)-P(1)	105.82(5)
C(20)-P(1)-Ni(1)	118.11(5)	C(26)-P(1)-Ni(1)	109.40(5)
C(26)-P(1)-C(20)	112.10(7)	C(26)-P(1)-C(32)	104.55(7)
C(32)-P(1)-Ni(1)	107.73(5)	C(32)-P(1)-C(20)	103.83(7)
C(1)-O(1)-Ni(1)	112.33(9)	C(6)-O(3)-C(7)	116.22(14)
C(15)-O(5)-C(16)	121.77(12)	C(2)-N(1)-C(8)	117.86(12)
C(15)-N(1)-C(2)	120.41(12)	C(15)-N(1)-C(8)	118.58(13)
O(1)-C(1)-H(1)	108.2	O(1)-C(1)-C(2)	108.70(12)
O(1)-C(1)-C(10)	115.46(13)	C(2)-C(1)-H(1)	108.2
C(10)-C(1)-H(1)	108.2	C(10)-C(1)-C(2)	107.99(12)
N(1)-C(2)-C(1)	111.15(12)	N(1)-C(2)-H(2)	108.7
N(1)-C(2)-C(3)	115.81(13)	C(1)-C(2)-H(2)	108.7

C(3)-C(2)-C(1)	103.47(12)	C(3)-C(2)-H(2)	108.7
Ni(1)-C(3)-H(3)	98.7(11)	C(2)-C(3)-Ni(1)	104.20(10)
C(2)-C(3)-H(3)	115.7(11)	C(4)-C(3)-Ni(1)	67.66(9)
C(4)-C(3)-C(2)	121.62(15)	C(4)-C(3)-H(3)	122.7(11)
Ni(1)-C(4)-H(4)	116.0(13)	C(3)-C(4)-Ni(1)	71.60(9)
C(3)-C(4)-H(4)	119.2(12)	C(3)-C(4)-C(5)	118.64(15)
C(5)-C(4)-Ni(1)	71.76(9)	C(5)-C(4)-H(4)	120.6(12)
Ni(1)-C(5)-H(5)	102.0(14)	C(4)-C(5)-Ni(1)	66.66(9)
C(4)-C(5)-H(5)	122.9(14)	C(4)-C(5)-C(6)	120.99(15)
C(6)-C(5)-Ni(1)	123.71(12)	C(6)-C(5)-H(5)	111.6(14)
O(2)-C(6)-O(3)	123.02(16)	O(2)-C(6)-C(5)	124.95(17)
O(3)-C(6)-C(5)	112.04(14)	O(3)-C(7)-H(7A)	109.5
O(3)-C(7)-H(7B)	109.5	O(3)-C(7)-H(7C)	109.5
H(7A)-C(7)-H(7B)	109.5	H(7A)-C(7)-H(7C)	109.5
H(7B)-C(7)-H(7C)	109.5	N(1)-C(8)-H(8A)	109.6
N(1)-C(8)-H(8B)	109.6	N(1)-C(8)-C(9)	110.33(13)
H(8A)-C(8)-H(8B)	108.1	C(9)-C(8)-H(8A)	109.6
C(9)-C(8)-H(8B)	109.6	C(10)-C(9)-C(8)	117.20(14)
C(14)-C(9)-C(8)	122.91(15)	C(14)-C(9)-C(10)	119.88(16)
C(9)-C(10)-C(1)	115.10(14)	C(11)-C(10)-C(1)	124.66(14)
C(11)-C(10)-C(9)	120.17(15)	C(10)-C(11)-H(11)	120.3
C(10)-C(11)-C(12)	119.47(16)	C(12)-C(11)-H(11)	120.3
C(11)-C(12)-H(12)	119.7	C(13)-C(12)-C(11)	120.55(17)
C(13)-C(12)-H(12)	119.7	C(12)-C(13)-H(13)	120.0
C(12)-C(13)-C(14)	119.94(17)	C(14)-C(13)-H(13)	120.0
C(9)-C(14)-H(14)	120.0	C(13)-C(14)-C(9)	119.96(16)
C(13)-C(14)-H(14)	120.0	O(4)-C(15)-O(5)	125.68(14)
O(4)-C(15)-N(1)	124.48(14)	O(5)-C(15)-N(1)	109.83(13)
O(5)-C(16)-C(17)	110.70(13)	O(5)-C(16)-C(18)	109.73(13)
O(5)-C(16)-C(19)	102.61(12)	C(17)-C(16)-C(18)	112.62(14)
C(17)-C(16)-C(19)	110.58(14)	C(18)-C(16)-C(19)	110.15(13)
C(16)-C(17)-H(17A)	109.5	C(16)-C(17)-H(17B)	109.5
C(16)-C(17)-H(17C)	109.5	H(17A)-C(17)-H(17B)	109.5
H(17A)-C(17)-H(17C)	109.5	H(17B)-C(17)-H(17C)	109.5
C(16)-C(18)-H(18A)	109.5	C(16)-C(18)-H(18B)	109.5
C(16)-C(18)-H(18C)	109.5	H(18A)-C(18)-H(18B)	109.5
H(18A)-C(18)-H(18C)	109.5	H(18B)-C(18)-H(18C)	109.5
C(16)-C(19)-H(19A)	109.5	C(16)-C(19)-H(19B)	109.5
C(16)-C(19)-H(19C)	109.5	H(19A)-C(19)-H(19B)	109.5
H(19A)-C(19)-H(19C)	109.5	H(19B)-C(19)-H(19C)	109.5
P(1)-C(20)-H(20)	105.5	C(21)-C(20)-P(1)	111.52(11)
C(21)-C(20)-H(20)	105.5	C(21)-C(20)-C(25)	110.65(13)
C(25)-C(20)-P(1)	117.12(11)	C(25)-C(20)-H(20)	105.5
C(20)-C(21)-H(21A)	109.5	C(20)-C(21)-H(21B)	109.5

H(21A)-C(21)-H(21B)	108.1	C(22)-C(21)-C(20)	110.85(14)
C(22)-C(21)-H(21A)	109.5	C(22)-C(21)-H(21B)	109.5
C(21)-C(22)-H(22A)	109.5	C(21)-C(22)-H(22B)	109.5
H(22A)-C(22)-H(22B)	108.1	C(23)-C(22)-C(21)	110.85(14)
C(23)-C(22)-H(22A)	109.5	C(23)-C(22)-H(22B)	109.5
C(22)-C(23)-H(23A)	109.5	C(22)-C(23)-H(23B)	109.5
H(23A)-C(23)-H(23B)	108.1	C(24)-C(23)-C(22)	110.76(14)
C(24)-C(23)-H(23A)	109.5	C(24)-C(23)-H(23B)	109.5
C(23)-C(24)-H(24A)	109.3	C(23)-C(24)-H(24B)	109.3
C(23)-C(24)-C(25)	111.49(14)	H(24A)-C(24)-H(24B)	108.0
C(25)-C(24)-H(24A)	109.3	C(25)-C(24)-H(24B)	109.3
C(20)-C(25)-H(25A)	109.5	C(20)-C(25)-H(25B)	109.5
C(24)-C(25)-C(20)	110.59(13)	C(24)-C(25)-H(25A)	109.5
C(24)-C(25)-H(25B)	109.5	H(25A)-C(25)-H(25B)	108.1
P(1)-C(26)-H(26)	104.6	C(27)-C(26)-P(1)	117.25(11)
C(27)-C(26)-H(26)	104.6	C(27)-C(26)-C(31)	110.87(13)
C(31)-C(26)-P(1)	113.55(11)	C(31)-C(26)-H(26)	104.6
C(26)-C(27)-H(27A)	109.6	C(26)-C(27)-H(27B)	109.6
C(26)-C(27)-C(28)	110.23(13)	H(27A)-C(27)-H(27B)	108.1
C(28)-C(27)-H(27A)	109.6	C(28)-C(27)-H(27B)	109.6
C(27)-C(28)-H(28A)	109.3	C(27)-C(28)-H(28B)	109.3
H(28A)-C(28)-H(28B)	108.0	C(29)-C(28)-C(27)	111.44(15)
C(29)-C(28)-H(28A)	109.3	C(29)-C(28)-H(28B)	109.3
C(28)-C(29)-H(29A)	109.3	C(28)-C(29)-H(29B)	109.3
C(28)-C(29)-C(30)	111.52(14)	H(29A)-C(29)-H(29B)	108.0
C(30)-C(29)-H(29A)	109.3	C(30)-C(29)-H(29B)	109.3
C(29)-C(30)-H(30A)	109.4	C(29)-C(30)-H(30B)	109.4
C(29)-C(30)-C(31)	111.18(14)	H(30A)-C(30)-H(30B)	108.0
C(31)-C(30)-H(30A)	109.4	C(31)-C(30)-H(30B)	109.4
C(26)-C(31)-H(31A)	109.6	C(26)-C(31)-H(31B)	109.6
C(30)-C(31)-C(26)	110.06(14)	C(30)-C(31)-H(31A)	109.6
C(30)-C(31)-H(31B)	109.6	H(31A)-C(31)-H(31B)	108.2
P(1)-C(32)-H(32)	108.2	C(33)-C(32)-P(1)	109.95(11)
C(33)-C(32)-H(32)	108.2	C(37)-C(32)-P(1)	112.19(11)
C(37)-C(32)-H(32)	108.2	C(37)-C(32)-C(33)	109.89(13)
C(32)-C(33)-H(33A)	109.4	C(32)-C(33)-H(33B)	109.4
H(33A)-C(33)-H(33B)	108.0	C(34)-C(33)-C(32)	111.08(13)
C(34)-C(33)-H(33A)	109.4	C(34)-C(33)-H(33B)	109.4
C(33)-C(34)-H(34A)	109.5	C(33)-C(34)-H(34B)	109.5
C(33)-C(34)-C(35)	110.64(14)	H(34A)-C(34)-H(34B)	108.1
C(35)-C(34)-H(34A)	109.5	C(35)-C(34)-H(34B)	109.5
C(34)-C(35)-H(35A)	109.4	C(34)-C(35)-H(35B)	109.4
H(35A)-C(35)-H(35B)	108.0	C(36)-C(35)-C(34)	111.00(14)
C(36)-C(35)-H(35A)	109.4	C(36)-C(35)-H(35B)	109.4

C(35)-C(36)-H(36A)	109.4	C(35)-C(36)-H(36B)	109.4
C(35)-C(36)-C(37)	111.32(15)	H(36A)-C(36)-H(36B)	108.0
C(37)-C(36)-H(36A)	109.4	C(37)-C(36)-H(36B)	109.4
C(32)-C(37)-H(37A)	109.4	C(32)-C(37)-H(37B)	109.4
C(36)-C(37)-C(32)	111.14(13)	C(36)-C(37)-H(37A)	109.4
C(36)-C(37)-H(37B)	109.4	H(37A)-C(37)-H(37B)	108.0
C(38)-O(6)-C(40)	113.4(6)	O(6)-C(38)-H(38A)	110.2
O(6)-C(38)-H(38B)	110.2	O(6)-C(38)-C(39)	107.7(7)
H(38A)-C(38)-H(38B)	108.5	C(39)-C(38)-H(38A)	110.2
C(39)-C(38)-H(38B)	110.2	C(38)-C(39)-H(39A)	109.5
C(38)-C(39)-H(39B)	109.5	C(38)-C(39)-H(39C)	109.5
H(39A)-C(39)-H(39B)	109.5	H(39A)-C(39)-H(39C)	109.5
H(39B)-C(39)-H(39C)	109.5	C(45)-C(39)-C(38)	121.4(13)
C(45)-C(39)-H(39A)	12.1	C(45)-C(39)-H(39B)	101.5
C(45)-C(39)-H(39C)	104.9	O(6)-C(40)-H(40A)	110.7
O(6)-C(40)-H(40B)	110.7	H(40A)-C(40)-H(40B)	108.8
C(41)-C(40)-O(6)	105.1(7)	C(41)-C(40)-H(40A)	110.7
C(41)-C(40)-H(40B)	110.7	C(46)-C(40)-O(6)	109.8(8)
C(46)-C(40)-H(40A)	55.8	C(46)-C(40)-H(40B)	56.9
C(46)-C(40)-C(41)	145.1(9)	C(46)-C(40)-C(47)	72.7(7)
C(47)-C(40)-O(6)	99.8(8)	C(47)-C(40)-H(40A)	18.3
C(47)-C(40)-H(40B)	127.1	C(47)-C(40)-C(41)	101.1(9)
C(40)-C(41)-H(41A)	109.5	C(40)-C(41)-H(41B)	109.5
C(40)-C(41)-H(41C)	109.5	H(41A)-C(41)-H(41B)	109.5
H(41A)-C(41)-H(41C)	109.5	H(41B)-C(41)-H(41C)	109.5
C(42)-O(7)-C(44)	105.4(8)	O(7)-C(42)-H(42A)	111.2
O(7)-C(42)-H(42B)	111.2	O(7)-C(42)-C(43)	103.0(9)
H(42A)-C(42)-H(42B)	109.1	C(43)-C(42)-H(42A)	111.2
C(43)-C(42)-H(42B)	111.2	C(42)-C(43)-H(43A)	109.5
C(42)-C(43)-H(43B)	109.5	C(42)-C(43)-H(43C)	109.5
H(43A)-C(43)-H(43B)	109.5	H(43A)-C(43)-H(43C)	109.5
H(43B)-C(43)-H(43C)	109.5	O(7)-C(44)-H(44A)	111.5
O(7)-C(44)-H(44B)	111.5	O(7)-C(44)-C(45)	101.3(7)
H(44A)-C(44)-H(44B)	109.3	C(45)-C(44)-H(44A)	111.5
C(45)-C(44)-H(44B)	111.5	C(39)-C(45)-C(44)	135.8(16)
C(39)-C(45)-H(45A)	95.1	C(39)-C(45)-H(45B)	26.3
C(39)-C(45)-H(45C)	95.2	C(44)-C(45)-H(45A)	109.5
C(44)-C(45)-H(45B)	109.5	C(44)-C(45)-H(45C)	109.5
H(45A)-C(45)-H(45B)	109.5	H(45A)-C(45)-H(45C)	109.5
H(45B)-C(45)-H(45C)	109.5	C(40)-C(46)-H(46A)	155.2
C(40)-C(46)-H(46B)	66.6	C(40)-C(46)-H(46C)	94.4
C(40)-C(46)-C(47)	54.0(7)	H(46A)-C(46)-H(46B)	109.5
H(46A)-C(46)-H(46C)	109.5	H(46B)-C(46)-H(46C)	109.5
C(47)-C(46)-H(46A)	109.5	C(47)-C(46)-H(46B)	109.5

C(47)-C(46)-H(46C)	109.5	C(40)-C(47)-C(46)	53.3(6)
C(40)-C(47)-H(47A)	109.0	C(40)-C(47)-H(47B)	60.1
C(40)-C(47)-C(48)	140.6(10)	C(46)-C(47)-H(47A)	110.2
C(46)-C(47)-H(47B)	110.2	H(47A)-C(47)-H(47B)	108.5
C(48)-C(47)-C(46)	107.6(9)	C(48)-C(47)-H(47A)	110.2
C(48)-C(47)-H(47B)	110.2	C(47)-C(48)-H(48A)	109.6
C(47)-C(48)-H(48B)	109.6	C(47)-C(48)-C(49)	110.4(8)
H(48A)-C(48)-H(48B)	108.1	C(49)-C(48)-H(48A)	109.6
C(49)-C(48)-H(48B)	109.6	C(48)-C(49)-H(49A)	108.1
C(48)-C(49)-H(49B)	108.1	H(49A)-C(49)-H(49B)	107.3
C(50)-C(49)-C(48)	116.6(10)	C(50)-C(49)-H(49A)	108.1
C(50)-C(49)-H(49B)	108.1	C(49)-C(50)-H(50A)	109.5
C(49)-C(50)-H(50B)	109.5	C(49)-C(50)-H(50C)	109.5
H(50A)-C(50)-H(50B)	109.5	H(50A)-C(50)-H(50C)	109.5
H(50B)-C(50)-H(50C)	109.5		

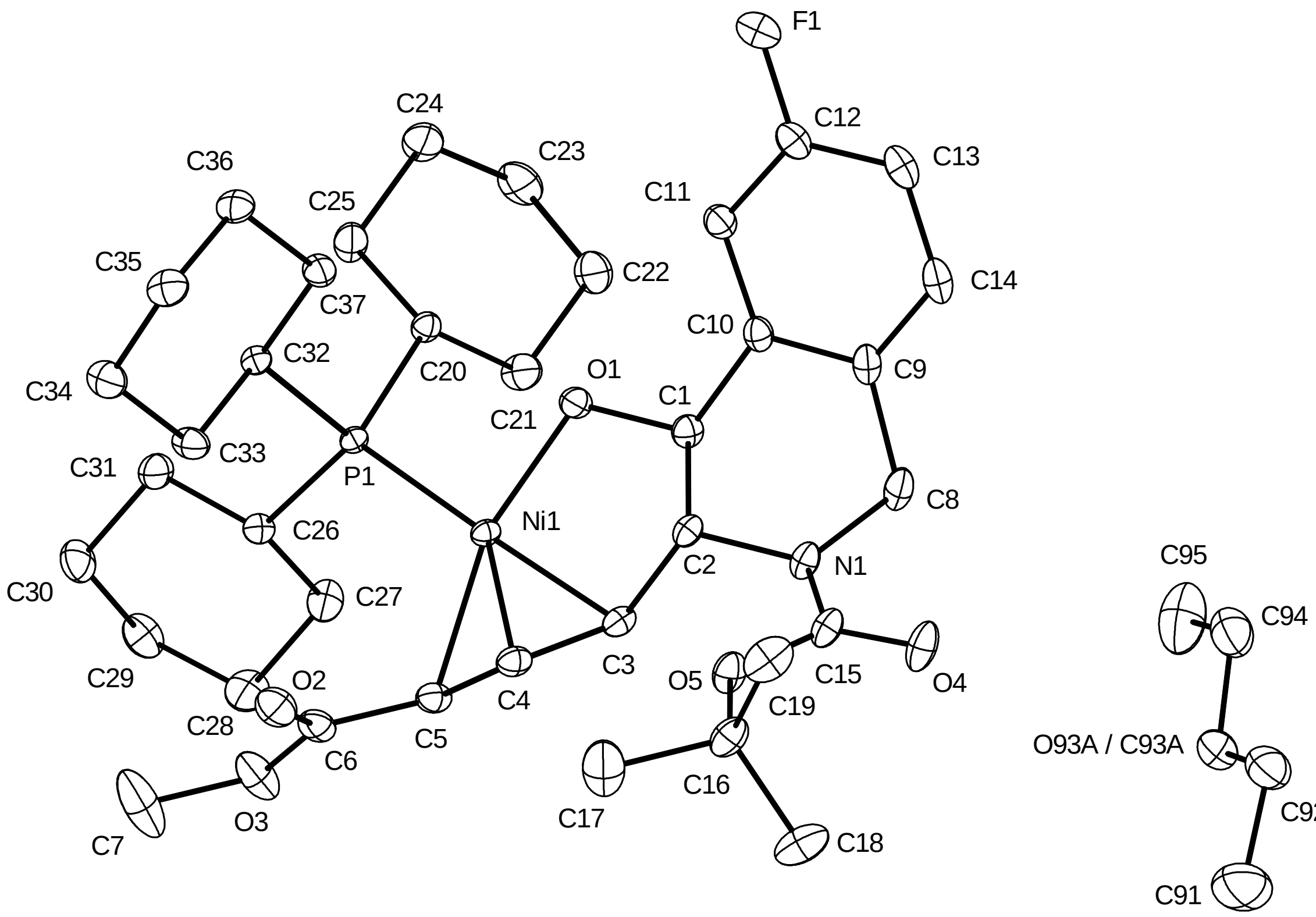

Figure S5. The asymmetric unit of complex **25e**; H-atoms have been removed for clarity.

$C_{41.36}$ $H_{65.72}$ F N Ni $O_{5.64}$ P, M_r = 775.932 g·mol^{-1}, orange prism, crystal size 0.226 x 0.191 x 0.103 mm^3, triclinic, space group *P*-1 [2], *a* = 10.4154(7) Å, *b* = 11.7385(8) Å, *c* = 17.3245(11) Å, α = 96.390(2)°, β = 99.258(2)°, γ = 95.742(3)°, *V* = 2062.5(2) Å^3, *T* = 100(2) K, *Z* = 2, D_{calc} = 1.249 g·cm^3, λ = 0.71073 Å, μ(*Mo-K*$_{\alpha}$) = 0.558 mm^{-1}, Gaussian absorption correction (T_{min} = 0.91542, T_{max} = 0.95970), Bruker-AXS Kappa Mach3 APEX-II diffractometer with IµS Mo-anode X-ray source, 1.20 < θ < 34.91°, 81912 measured reflections, 17697 independent reflections, 14482 reflections with $I > 2\sigma(I)$, R_{int} = 0.0353. The structure was solved by *SHELXT* and refined by full-matrix least-squares (*SHELXL*). The final structure refinement was performed by olex2.refine 1.5 (L-M) together with NoSpherA2 (atomic form factors) against F^2 to R_1 = 0.0267 [$I > 2\sigma(I)$], wR_2 = 0.0529, 679 parameters. **CCDC-2552554**

The final structure refinement was carried out using aspherical scattering factors with NoSpherA2. NoSpherA2 implementation of Hirshfeld Atom Refinement (HAR) makes use of tailor-made aspherical atomic form factors calculated on-the-fly from a Hirshfeld-partitioned electron density (ED) - not from spherical-atom form factors. The ED is calculated from a Gaussian basis set single determinant SCF wave function for a fragment of the crystal. This fragment can be embedded in an electrostatic crystal field by employing cluster charges or modelled using implicit solvation models, depending on the software used. The following options were chosen:

SOFTWARE: ORCA 6.0
PARTITIONING: NoSpherA2
INT ACCURACY: Normal
METHOD: PBE
BASIS SET: def2-TZVPP
CHARGE: 0
MULTIPLICITY: 1

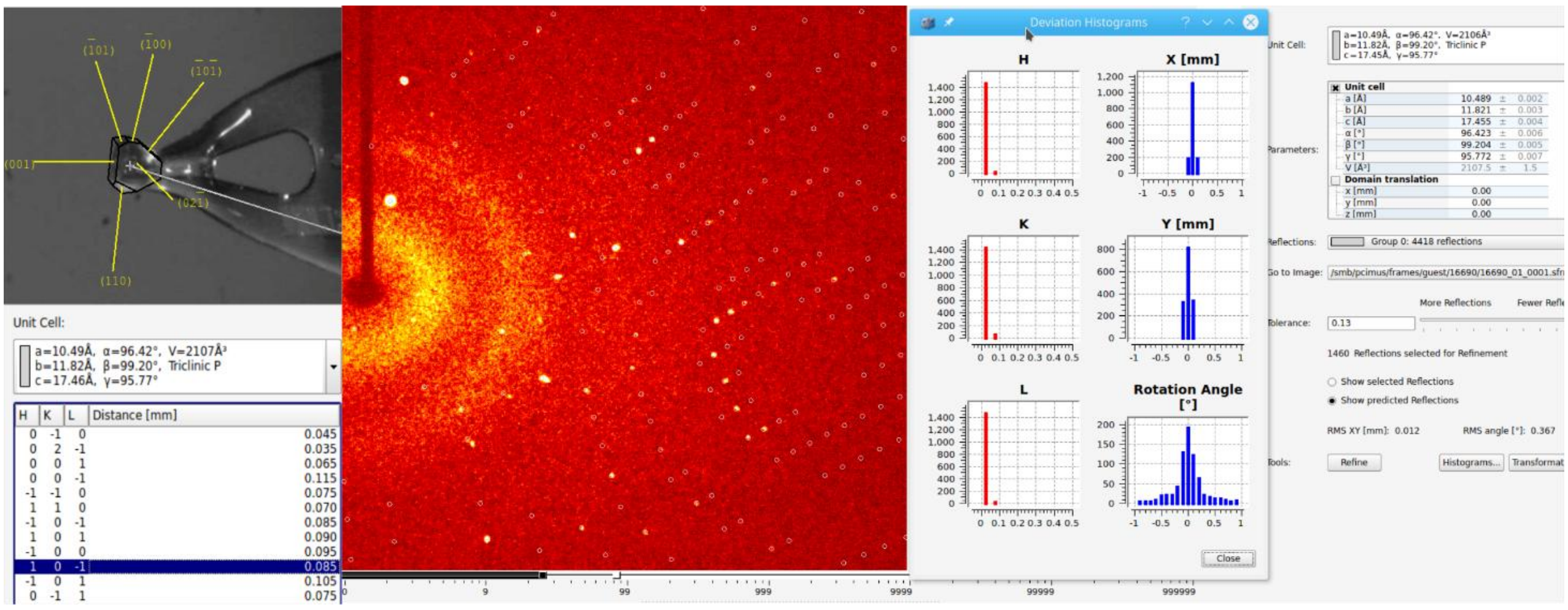

Figure S6. Crystal faces and unit cell determination/refinement of complex **25e**.

INTENSITY STATISTICS FOR DATASET

Resolution	#Data	#Theory	%Complete	Redundancy	Mean I	Mean I/s	Rmerge	Rsigma
Inf - 2.54	266	266	100.0	9.41	55.69	78.53	0.0210	0.0104
2.54 - 1.69	620	620	100.0	9.59	27.85	69.83	0.0232	0.0109
1.69 - 1.34	904	904	100.0	9.52	16.45	59.34	0.0251	0.0123
1.34 - 1.17	894	894	100.0	9.20	11.54	48.71	0.0302	0.0145
1.17 - 1.06	944	944	100.0	7.69	8.98	38.47	0.0360	0.0184
1.06 - 0.99	818	818	100.0	5.78	6.73	28.49	0.0432	0.0251
0.99 - 0.93	926	926	100.0	4.78	6.34	25.03	0.0467	0.0293
0.93 - 0.88	967	967	100.0	4.22	5.13	20.61	0.0487	0.0361
0.88 - 0.84	960	960	100.0	3.84	4.10	16.65	0.0600	0.0443
0.84 - 0.81	831	831	100.0	3.80	3.98	15.79	0.0598	0.0477
0.81 - 0.78	963	963	100.0	3.57	3.32	13.36	0.0685	0.0574
0.78 - 0.76	753	753	100.0	3.47	2.96	11.90	0.0754	0.0641
0.76 - 0.74	813	815	99.8	3.38	2.67	10.70	0.0827	0.0722
0.74 - 0.72	920	922	99.8	3.20	2.54	9.66	0.0855	0.0801
0.72 - 0.70	1013	1020	99.3	3.12	2.27	8.97	0.0966	0.0884
0.70 - 0.68	1137	1148	99.0	2.97	2.18	8.06	0.1070	0.0989
0.68 - 0.67	621	625	99.4	2.92	2.16	7.77	0.1069	0.1026
0.67 - 0.65	1302	1327	98.1	2.82	1.87	6.83	0.1259	0.1189
0.65 - 0.64	751	764	98.3	2.61	1.72	5.97	0.1278	0.1387
0.64 - 0.63	812	833	97.5	2.65	1.45	5.45	0.1519	0.1556
0.63 - 0.62	482	716	67.3	1.63	1.28	4.58	0.1626	0.1842
0.72 - 0.62	6118	6433	95.1	2.73	1.90	7.04	0.1169	0.1161
Inf - 0.62	17697	18016	98.2	4.55	6.27	21.23	0.0350	0.0331

Table S5. Crystal data and structure refinement of complex **25e**

Empirical formula	$C_{41.36} H_{65.72} F N Ni O_{5.64} P$	
Color	orange	
Formula weight	775.932 g·mol^{-1}	
Temperature	100(2) K	
Wavelength	0.71073 Å	
Crystal system	Triclinic	
Space group	*P*-1, (no. 2)	
Unit cell dimensions	a = 10.4154(7) Å	α= 96.390(2)°.
	b = 11.7385(8) Å	β= 99.258(2)°.
	c = 17.3245(11) Å	γ = 95.742(3)°.
Volume	2062.5(2) Å^3	
Z	2	
Density (calculated)	1.249 Mg·m^{-3}	
Absorption coefficient	0.558 mm^{-1}	
F(000)	837.430 e	
Crystal size	0.226 x 0.191 x 0.103 mm^3	
θ range for data collection	1.20 to 34.91°.	
Index ranges	$-16 \le h \le 16$, $-18 \le k \le 18$, $-27 \le l \le 27$	
Reflections collected	81912	
Independent reflections	17697 [R_{int} = 0.0353]	
Reflections with I>2σ(I)	14482	
Completeness to θ = 25.2417°	100.00 %	
Absorption correction	Gaussian	
Max. and min. transmission	0.95970 and 0.91542	
Refinement method	Full-matrix least-squares on F^2	
Data / restraints / parameters	17697 / 0 / 679	
Goodness-of-fit on F^2	1.0216	
Final R indices [I>2σ(I)]	R_1 = 0.0267	wR^2 = 0.0494
R indices (all data)	R_1 = 0.0400	wR^2 = 0.0529
Largest diff. peak and hole	0.4022 and -0.3483 e·Å^{-3}	

Table S6. Bond lengths [Å] and angles [°] of complex **25e**

Ni(1)-P(1)	2.2018(2)	Ni(1)-O(1)	1.8587(5)
Ni(1)-C(3)	2.0304(6)	Ni(1)-C(4)	1.9697(6)
Ni(1)-C(5)	2.0314(6)	P(1)-C(20)	1.8420(6)
P(1)-C(26)	1.8400(6)	P(1)-C(32)	1.8439(6)
F(1)-C(12)	1.3528(8)	O(1)-C(1)	1.3818(7)
O(2)-C(6)	1.2103(9)	O(3)-C(6)	1.3477(9)
O(3)-C(7)	1.4339(10)	O(4)-C(15)	1.2167(8)
O(5)-C(15)	1.3429(8)	O(5)-C(16)	1.4679(8)
N(1)-C(2)	1.4650(8)	N(1)-C(8)	1.4694(9)
N(1)-C(15)	1.3652(9)	C(1)-C(2)	1.5550(9)
C(1)-C(10)	1.4981(9)	C(1)-H(1)	1.101(8)
C(2)-C(3)	1.5010(9)	C(2)-H(2)	1.072(8)
C(3)-H(3)	1.082(9)	C(3)-C(4)	1.3940(9)
C(4)-H(4)	1.076(9)	C(4)-C(5)	1.4267(9)
C(5)-H(5)	1.068(9)	C(5)-C(6)	1.4685(9)
C(7)-H(7a)	1.090(12)	C(7)-H(7b)	1.063(12)
C(7)-H(7c)	1.050(13)	C(8)-H(8a)	1.101(8)
C(8)-H(8b)	1.085(8)	C(8)-C(9)	1.5037(10)
C(9)-C(10)	1.3988(9)	C(9)-C(14)	1.3888(10)
C(10)-C(11)	1.3860(9)	C(11)-H(11)	1.084(8)
C(11)-C(12)	1.3857(9)	C(12)-C(13)	1.3817(10)
C(13)-H(13)	1.066(8)	C(13)-C(14)	1.3925(10)
C(14)-H(14)	1.080(8)	C(16)-C(17)	1.5198(11)
C(16)-C(18)	1.5191(10)	C(16)-C(19)	1.5206(11)
C(17)-H(17a)	1.049(11)	C(17)-H(17b)	1.084(11)
C(17)-H(17c)	1.090(11)	C(18)-H(18a)	1.085(10)
C(18)-H(18b)	1.076(10)	C(18)-H(18c)	1.055(10)
C(19)-H(19a)	1.082(10)	C(19)-H(19b)	1.088(10)
C(19)-H(19c)	1.070(10)	C(20)-H(20)	1.091(8)
C(20)-C(21)	1.5290(9)	C(20)-C(25)	1.5248(9)
C(21)-H(21a)	1.072(8)	C(21)-H(21b)	1.052(8)
C(21)-C(22)	1.5306(10)	C(22)-H(22a)	1.089(9)
C(22)-H(22b)	1.088(8)	C(22)-C(23)	1.5251(11)
C(23)-H(23a)	1.091(9)	C(23)-H(23b)	1.088(9)
C(23)-C(24)	1.5227(11)	C(24)-H(24a)	1.106(9)
C(24)-H(24b)	1.066(9)	C(24)-C(25)	1.5275(10)
C(25)-H(25a)	1.100(9)	C(25)-H(25b)	1.080(8)
C(26)-H(26)	1.092(8)	C(26)-C(27)	1.5312(9)
C(26)-C(31)	1.5323(9)	C(27)-H(27a)	1.071(8)
C(27)-H(27b)	1.099(8)	C(27)-C(28)	1.5259(10)
C(28)-H(28a)	1.057(9)	C(28)-H(28b)	1.105(9)
C(28)-C(29)	1.5243(11)	C(29)-H(29a)	1.066(9)

C(29)-H(29b)	1.093(8)	C(29)-C(30)	1.5241(11)
C(30)-H(30a)	1.078(8)	C(30)-H(30b)	1.091(9)
C(30)-C(31)	1.5303(10)	C(31)-H(31a)	1.075(8)
C(31)-H(31b)	1.095(8)	C(32)-H(32)	1.087(7)
C(32)-C(33)	1.5348(9)	C(32)-C(37)	1.5355(9)
C(33)-H(33a)	1.073(8)	C(33)-H(33b)	1.082(8)
C(33)-C(34)	1.5287(9)	C(34)-H(34a)	1.093(8)
C(34)-H(34b)	1.066(8)	C(34)-C(35)	1.5239(10)
C(35)-H(35a)	1.097(8)	C(35)-H(35b)	1.072(8)
C(35)-C(36)	1.5234(10)	C(36)-H(36a)	1.112(8)
C(36)-H(36b)	1.089(8)	C(36)-C(37)	1.5284(9)
C(37)-H(37a)	1.092(8)	C(37)-H(37b)	1.087(8)
C(93B)-H(93a)	1.10(3)	C(93B)-H(93b)	1.06(3)
C(93B)-C(92)	1.4363(11)	C(93B)-C(94)	1.4571(11)
C(91)-H(91a)	1.098(13)	C(91)-H(91b)	1.089(13)
C(91)-H(91c)	1.092(13)	C(91)-C(92)	1.5080(13)
C(92)-H(92c)	1.28(3)	C(92)-H(92d)	1.13(4)
C(92)-H(92a)	1.10(2)	C(92)-H(92b)	0.997(19)
C(92)-O(93A)	1.4363(11)	C(94)-H(94c)	1.13(4)
C(94)-H(94d)	1.23(3)	C(94)-H(94a)	1.07(2)
C(94)-H(94b)	1.08(3)	C(94)-C(95)	1.5098(14)
C(94)-O(93A)	1.4571(11)	C(95)-H(95a)	1.064(13)
C(95)-H(95b)	1.094(13)	C(95)-H(95c)	1.049(13)

O(1)-Ni(1)-P(1)	92.064(14)	C(3)-Ni(1)-P(1)	171.29(2)
C(3)-Ni(1)-O(1)	86.90(2)	C(4)-Ni(1)-P(1)	144.906(19)
C(4)-Ni(1)-O(1)	118.21(2)	C(4)-Ni(1)-C(3)	40.76(3)
C(5)-Ni(1)-P(1)	108.831(19)	C(5)-Ni(1)-O(1)	159.06(2)
C(5)-Ni(1)-C(3)	72.66(3)	C(5)-Ni(1)-C(4)	41.75(3)
C(20)-P(1)-Ni(1)	107.92(2)	C(26)-P(1)-Ni(1)	115.64(2)
C(26)-P(1)-C(20)	112.50(3)	C(32)-P(1)-Ni(1)	111.81(2)
C(32)-P(1)-C(20)	104.06(3)	C(32)-P(1)-C(26)	104.31(3)
C(1)-O(1)-Ni(1)	111.03(4)	C(7)-O(3)-C(6)	115.69(7)
C(16)-O(5)-C(15)	120.60(5)	C(8)-N(1)-C(2)	118.05(5)
C(15)-N(1)-C(2)	121.28(5)	C(15)-N(1)-C(8)	117.75(5)
C(2)-C(1)-O(1)	108.70(5)	C(10)-C(1)-O(1)	114.57(5)
C(10)-C(1)-C(2)	109.53(5)	H(1)-C(1)-O(1)	112.7(4)
H(1)-C(1)-C(2)	103.9(4)	H(1)-C(1)-C(10)	106.8(4)
C(1)-C(2)-N(1)	110.62(5)	C(3)-C(2)-N(1)	117.03(5)
C(3)-C(2)-C(1)	102.56(5)	H(2)-C(2)-N(1)	110.6(4)
H(2)-C(2)-C(1)	106.6(4)	H(2)-C(2)-C(3)	108.7(4)
C(2)-C(3)-Ni(1)	103.46(4)	H(3)-C(3)-Ni(1)	99.1(5)
H(3)-C(3)-C(2)	117.2(4)	C(4)-C(3)-Ni(1)	67.28(3)
C(4)-C(3)-C(2)	123.69(6)	C(4)-C(3)-H(3)	119.1(5)

C(3)-C(4)-Ni(1)	71.96(4)	H(4)-C(4)-Ni(1)	112.7(5)
H(4)-C(4)-C(3)	120.6(4)	C(5)-C(4)-Ni(1)	71.44(4)
C(5)-C(4)-C(3)	117.09(6)	C(5)-C(4)-H(4)	120.1(4)
C(4)-C(5)-Ni(1)	66.81(4)	H(5)-C(5)-Ni(1)	103.2(5)
H(5)-C(5)-C(4)	119.8(5)	C(6)-C(5)-Ni(1)	121.58(5)
C(6)-C(5)-C(4)	118.57(6)	C(6)-C(5)-H(5)	116.3(5)
O(3)-C(6)-O(2)	122.80(6)	C(5)-C(6)-O(2)	126.15(6)
C(5)-C(6)-O(3)	111.05(6)	H(7a)-C(7)-O(3)	109.3(6)
H(7b)-C(7)-O(3)	106.8(6)	H(7b)-C(7)-H(7a)	114.7(9)
H(7c)-C(7)-O(3)	111.1(6)	H(7c)-C(7)-H(7a)	105.0(9)
H(7c)-C(7)-H(7b)	110.0(9)	H(8a)-C(8)-N(1)	109.2(4)
H(8b)-C(8)-N(1)	107.2(4)	H(8b)-C(8)-H(8a)	107.2(6)
C(9)-C(8)-N(1)	110.25(5)	C(9)-C(8)-H(8a)	110.7(4)
C(9)-C(8)-H(8b)	112.1(4)	C(10)-C(9)-C(8)	116.47(6)
C(14)-C(9)-C(8)	123.86(6)	C(14)-C(9)-C(10)	119.65(6)
C(9)-C(10)-C(1)	115.38(6)	C(11)-C(10)-C(1)	123.46(5)
C(11)-C(10)-C(9)	121.13(6)	H(11)-C(11)-C(10)	121.5(4)
C(12)-C(11)-C(10)	117.44(6)	C(12)-C(11)-H(11)	121.0(4)
C(11)-C(12)-F(1)	118.86(6)	C(13)-C(12)-F(1)	117.95(6)
C(13)-C(12)-C(11)	123.19(7)	H(13)-C(13)-C(12)	119.7(4)
C(14)-C(13)-C(12)	118.37(6)	C(14)-C(13)-H(13)	122.0(4)
C(13)-C(14)-C(9)	120.22(6)	H(14)-C(14)-C(9)	120.5(4)
H(14)-C(14)-C(13)	119.3(4)	O(5)-C(15)-O(4)	125.40(6)
N(1)-C(15)-O(4)	124.13(6)	N(1)-C(15)-O(5)	110.44(5)
C(17)-C(16)-O(5)	102.40(6)	C(18)-C(16)-O(5)	110.93(6)
C(18)-C(16)-C(17)	110.40(7)	C(19)-C(16)-O(5)	109.53(6)
C(19)-C(16)-C(17)	110.84(7)	C(19)-C(16)-C(18)	112.30(6)
H(17a)-C(17)-C(16)	109.8(6)	H(17b)-C(17)-C(16)	110.7(5)
H(17b)-C(17)-H(17a)	107.7(8)	H(17c)-C(17)-C(16)	110.3(5)
H(17c)-C(17)-H(17a)	108.8(8)	H(17c)-C(17)-H(17b)	109.5(8)
H(18a)-C(18)-C(16)	110.7(5)	H(18b)-C(18)-C(16)	110.2(5)
H(18b)-C(18)-H(18a)	112.2(7)	H(18c)-C(18)-C(16)	109.0(5)
H(18c)-C(18)-H(18a)	109.8(8)	H(18c)-C(18)-H(18b)	104.8(8)
H(19a)-C(19)-C(16)	110.5(5)	H(19b)-C(19)-C(16)	109.7(5)
H(19b)-C(19)-H(19a)	107.8(7)	H(19c)-C(19)-C(16)	111.6(5)
H(19c)-C(19)-H(19a)	107.7(7)	H(19c)-C(19)-H(19b)	109.4(7)
H(20)-C(20)-P(1)	101.2(4)	C(21)-C(20)-P(1)	113.71(4)
C(21)-C(20)-H(20)	104.8(4)	C(25)-C(20)-P(1)	117.56(4)
C(25)-C(20)-H(20)	107.8(4)	C(25)-C(20)-C(21)	110.38(6)
H(21a)-C(21)-C(20)	110.1(4)	H(21b)-C(21)-C(20)	108.8(4)
H(21b)-C(21)-H(21a)	107.3(6)	C(22)-C(21)-C(20)	110.43(6)
C(22)-C(21)-H(21a)	111.1(4)	C(22)-C(21)-H(21b)	109.0(5)
H(22a)-C(22)-C(21)	109.1(5)	H(22b)-C(22)-C(21)	109.9(5)
H(22b)-C(22)-H(22a)	106.0(6)	C(23)-C(22)-C(21)	111.74(6)

C(23)-C(22)-H(22a)	109.7(5)	C(23)-C(22)-H(22b)	110.2(5)
H(23a)-C(23)-C(22)	110.2(5)	H(23b)-C(23)-C(22)	108.4(5)
H(23b)-C(23)-H(23a)	106.1(7)	C(24)-C(23)-C(22)	111.22(6)
C(24)-C(23)-H(23a)	111.2(5)	C(24)-C(23)-H(23b)	109.6(5)
H(24a)-C(24)-C(23)	107.6(5)	H(24b)-C(24)-C(23)	110.7(5)
H(24b)-C(24)-H(24a)	106.4(7)	C(25)-C(24)-C(23)	111.28(7)
C(25)-C(24)-H(24a)	109.3(5)	C(25)-C(24)-H(24b)	111.4(5)
C(24)-C(25)-C(20)	110.08(6)	H(25a)-C(25)-C(20)	109.9(4)
H(25a)-C(25)-C(24)	109.9(4)	H(25b)-C(25)-C(20)	110.1(5)
H(25b)-C(25)-C(24)	110.6(4)	H(25b)-C(25)-H(25a)	106.1(6)
H(26)-C(26)-P(1)	102.3(4)	C(27)-C(26)-P(1)	112.08(4)
C(27)-C(26)-H(26)	106.0(4)	C(31)-C(26)-P(1)	118.17(4)
C(31)-C(26)-H(26)	106.7(4)	C(31)-C(26)-C(27)	110.36(6)
H(27a)-C(27)-C(26)	109.8(4)	H(27b)-C(27)-C(26)	108.9(4)
H(27b)-C(27)-H(27a)	109.4(6)	C(28)-C(27)-C(26)	110.19(6)
C(28)-C(27)-H(27a)	109.5(4)	C(28)-C(27)-H(27b)	108.9(4)
H(28a)-C(28)-C(27)	109.4(5)	H(28b)-C(28)-C(27)	109.3(5)
H(28b)-C(28)-H(28a)	107.1(6)	C(29)-C(28)-C(27)	111.52(6)
C(29)-C(28)-H(28a)	110.7(5)	C(29)-C(28)-H(28b)	108.8(4)
H(29a)-C(29)-C(28)	109.1(4)	H(29b)-C(29)-C(28)	110.8(4)
H(29b)-C(29)-H(29a)	107.6(6)	C(30)-C(29)-C(28)	110.88(6)
C(30)-C(29)-H(29a)	108.8(4)	C(30)-C(29)-H(29b)	109.4(4)
H(30a)-C(30)-C(29)	110.0(4)	H(30b)-C(30)-C(29)	109.8(4)
H(30b)-C(30)-H(30a)	107.1(6)	C(31)-C(30)-C(29)	111.51(6)
C(31)-C(30)-H(30a)	109.8(4)	C(31)-C(30)-H(30b)	108.6(4)
C(30)-C(31)-C(26)	110.41(6)	H(31a)-C(31)-C(26)	110.0(4)
H(31a)-C(31)-C(30)	108.7(4)	H(31b)-C(31)-C(26)	109.9(4)
H(31b)-C(31)-C(30)	109.9(4)	H(31b)-C(31)-H(31a)	107.9(6)
H(32)-C(32)-P(1)	107.0(4)	C(33)-C(32)-P(1)	110.44(4)
C(33)-C(32)-H(32)	107.6(4)	C(37)-C(32)-P(1)	112.23(4)
C(37)-C(32)-H(32)	109.6(4)	C(37)-C(32)-C(33)	109.80(5)
H(33a)-C(33)-C(32)	110.4(4)	H(33b)-C(33)-C(32)	109.7(4)
H(33b)-C(33)-H(33a)	106.7(6)	C(34)-C(33)-C(32)	111.43(5)
C(34)-C(33)-H(33a)	109.0(4)	C(34)-C(33)-H(33b)	109.5(4)
H(34a)-C(34)-C(33)	107.8(4)	H(34b)-C(34)-C(33)	109.8(4)
H(34b)-C(34)-H(34a)	106.3(6)	C(35)-C(34)-C(33)	111.22(6)
C(35)-C(34)-H(34a)	109.9(4)	C(35)-C(34)-H(34b)	111.7(5)
H(35a)-C(35)-C(34)	109.5(4)	H(35b)-C(35)-C(34)	109.5(4)
H(35b)-C(35)-H(35a)	108.2(6)	C(36)-C(35)-C(34)	111.31(6)
C(36)-C(35)-H(35a)	110.0(4)	C(36)-C(35)-H(35b)	108.4(4)
H(36a)-C(36)-C(35)	108.9(4)	H(36b)-C(36)-C(35)	109.9(4)
H(36b)-C(36)-H(36a)	107.2(6)	C(37)-C(36)-C(35)	111.95(5)
C(37)-C(36)-H(36a)	109.0(4)	C(37)-C(36)-H(36b)	109.8(4)
C(36)-C(37)-C(32)	110.70(5)	H(37a)-C(37)-C(32)	109.4(4)

H(37a)-C(37)-C(36)	110.5(4)	H(37b)-C(37)-C(32)	110.7(4)
H(37b)-C(37)-C(36)	110.6(4)	H(37b)-C(37)-H(37a)	104.8(6)
H(93b)-C(93B)-H(93a)	104.3(18)	C(92)-C(93B)-H(93a)	114.6(13)
C(92)-C(93B)-H(93b)	118.1(13)	C(94)-C(93B)-H(93a)	104.0(13)
C(94)-C(93B)-H(93b)	101.3(13)	C(94)-C(93B)-C(92)	112.80(7)
H(91b)-C(91)-H(91a)	107.2(9)	H(91c)-C(91)-H(91a)	109.2(9)
H(91c)-C(91)-H(91b)	110.6(9)	C(92)-C(91)-H(91a)	109.8(7)
C(92)-C(91)-H(91b)	109.5(7)	C(92)-C(91)-H(91c)	110.5(7)
C(91)-C(92)-C(93B)	110.52(8)	H(92c)-C(92)-C(93B)	99.8(15)
H(92c)-C(92)-C(91)	108.8(16)	H(92d)-C(92)-C(93B)	104.8(19)
H(92d)-C(92)-C(91)	102.4(18)	H(92d)-C(92)-H(92c)	130(2)
H(92a)-C(92)-C(93B)	110.7(11)	H(92a)-C(92)-C(91)	115.9(10)
H(92a)-C(92)-H(92c)	109.9(17)	H(92a)-C(92)-H(92d)	20.1(18)
H(92b)-C(92)-C(93B)	115.9(10)	H(92b)-C(92)-C(91)	108.7(12)
H(92b)-C(92)-H(92c)	18(2)	H(92b)-C(92)-H(92d)	113.8(19)
H(92b)-C(92)-H(92a)	94.5(13)	O(93A)-C(92)-C(93B)	0.0
O(93A)-C(92)-C(91)	110.52(8)	O(93A)-C(92)-H(92c)	99.8(15)
O(93A)-C(92)-H(92d)	104.8(19)	O(93A)-C(92)-H(92a)	110.7(11)
O(93A)-C(92)-H(92b)	115.9(10)	H(94c)-C(94)-C(93B)	108(2)
H(94d)-C(94)-C(93B)	96.7(14)	H(94d)-C(94)-H(94c)	129(2)
H(94a)-C(94)-C(93B)	115.4(9)	H(94a)-C(94)-H(94c)	111(2)
H(94a)-C(94)-H(94d)	21.2(17)	H(94b)-C(94)-C(93B)	108.5(12)
H(94b)-C(94)-H(94c)	18(2)	H(94b)-C(94)-H(94d)	111.8(18)
H(94b)-C(94)-H(94a)	95.1(13)	C(95)-C(94)-C(93B)	111.11(8)
C(95)-C(94)-H(94c)	101.0(17)	C(95)-C(94)-H(94d)	110.6(17)
C(95)-C(94)-H(94a)	109.6(11)	C(95)-C(94)-H(94b)	116.4(10)
O(93A)-C(94)-C(93B)	0.0	O(93A)-C(94)-H(94c)	108(2)
O(93A)-C(94)-H(94d)	96.7(14)	O(93A)-C(94)-H(94a)	115.4(9)
O(93A)-C(94)-H(94b)	108.5(12)	O(93A)-C(94)-C(95)	111.11(8)
H(95a)-C(95)-C(94)	111.4(7)	H(95b)-C(95)-C(94)	111.1(7)
H(95b)-C(95)-H(95a)	108.3(9)	H(95c)-C(95)-C(94)	109.2(7)
H(95c)-C(95)-H(95a)	107.3(9)	H(95c)-C(95)-H(95b)	109.4(10)
C(94)-O(93A)-C(92)	112.80(7)		

General Information

Unless stated otherwise, all reactions were carried out under argon atmosphere in flame-dried Schlenk glassware.

Solvents were purified by distillation over the indicated drying agents under argon: toluene ($NaAlEt_4$), THF (Mg/anthracene), Et_2O (Mg/anthracene), *tert*-butyl methyl ether (CaH_2), pentane (Na/K), CH_2Cl_2 (CaH_2), $CHCl_3$ ($CaCl_2$), methanol (Mg). DMF and NEt_3 were dried by an absorption solvent purification system based on molecular sieves.

Flash chromatography: Merck silica gel 60 (40-63 µm or 15-40 µm). Thin layer chromatography (TLC): Macherey-Nagel precoated plates (POLYGRAM®SIL/UV254); visualization by UV light (254 nm) and/or by staining with solutions of *p*-anisaldeyhde, ninhydrin, ceric ammonium nitrate, or $KMnO_4$.

NMR: Spectra were recorded on Bruker Avance III 300, 400 MHz or Avance Neo 600 MHz (equipped with a Bruker BBO CryoProbe) NMR spectrometers in the solvents indicated; chemical shifts are given in ppm relative to TMS, coupling constants (*J*) in Hz. The solvent signals were used as references and the chemical shifts converted to the TMS scale ($CDCl_3$: δ_C = 77.16 ppm; residual $CHCl_3$ in $CDCl_3$: δ_H = 7.26 ppm; [D_6]-DMSO: δ_C = 39.52 ppm; residual D_2HSOCD_3 in [D_6]-DMSO: δ_H = 2.50 ppm; CD_2Cl_2: δ_C = 53.84 ppm; residual $CDHCl_2$ in CD_2Cl_2: δ_H = 5.32 ppm; residual CHD_2OD in CD_3OD: δ_H = 3.31 ppm; CD_3OD: δ_C = 49.0 ppm). All spectra were recorded at 298 K unless stated otherwise. Peak multiplicities are indicated by the following abbreviations: s: singlet, d: doublet, t: triplet, q: quartet, p: pentet, h: hextet, hept: heptet, m: multiplet, dd: double doublet, dt: double triplet, and so on; "br" indicates a broad peak, where the width is large enough to eclipse small coupling constants, which might otherwise be expected.

IR: Spectra were recorded on an Alpha Platinum ATR instrument (Bruker); wavenumbers ($\tilde{\nu}$) are reported in cm^{-1}.

MS (EI): Finnigan MAT 8200 (70 eV), ESI-MS: ESQ3000 (Bruker), Thermo Scientific LTQ-FT, or Thermo Scientific Exactive; HRMS: Bruker APEX III FT-MS (7T magnet), MAT 95 (Finnigan), Thermo Scientific LTQ-FT, or Thermo Scientific Exactive; GC-MS: Shimadzu GCMS-QP2010 Ultra instrument, GC-MS (CI): Q Exactive GC Orbitrap.

HPLC analyses for the determination of the enantiomeric excess (ee) of the individual compounds were conducted on a Shimadzu LC 20 instrument equipped with a Shimadzu SPD-M20A UV/VIS detector, or on a Shimadzu LC 40xR instrument equipped with a Shimadzu SPD-40 UV/VIS detector. Solvents (HPLC grade) were purchased and used as received. The exact conditions are specified for each compound.

Optical rotations were measured with an A-Krüss Otronic Model P8000-t polarimeter at a wavelength of 589 nm. The values are given as specific optical rotation with exact temperature, concentration (c (1.0 = 10 mg/mL)) and solvent.

Melting points were determined using a BÜCHI B-540.

(Ni(*t*Bu-stb)$_3$) was purchased from STREM chemicals, (*S*)-AntPhos and *rac*-AntPhos from BLDpharm. Triethylborane (1.0 M in THF) was prepared from neat triethylborane and freshly distilled THF. Diethylzinc (1.0 M in hexane) was purchased from Sigma Aldrich. Unless stated otherwise, all other commercially available compounds (TCI Chemicals, Fisher Scientific, Sigma Aldrich, abcr, BLDpharm) were used as received.

The amino-pentadienoates **1a** and **1b** required for the substrate synthesis were prepared on multi-gram scale following our previously established procedure.[1]

Building Blocks

2-Methyl-4-(phenylsulfonyl)benzonitrile (S1).

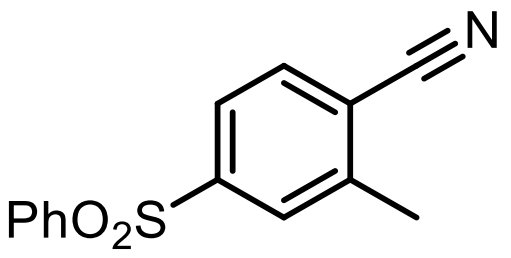

A 2-neck flask was charged with 4-bromo-2-methylbenzonitrile (1.37 g, 7.00 mmol), sodium benzenesulfonate (1.38 g, 8.40 mmol), tris(dibenzylideneacetone)dipalladium(0) (160 mg, 0.18 mmol), XantPhos (203 mg, 0.35 mmol), caesium carbonate (3.42 g, 10.5 mmol) and toluene (40 mL). The mixture was stirred at reflux temperature overnight. The mixture was allowed to cool to room temperature before it was diluted with water (50 mL) and ethyl acetate (100 mL). The organic phase was separated, washed with water (50 mL) and brine (50 mL), dried over $MgSO_4$ and filtered, and the solvent was removed under reduced pressure. The residue was purified by flash chromatography (SiO_2, *n*-hexane/ethyl acetate, 4:1) to give the title compound as a red-brown solid (1.45 g, 80%). ^1H NMR (600 MHz, $CDCl_3$): δ = 7.97 – 7.93 (m, 2H), 7.90 (dt, *J* = 1.9, 0.7 Hz, 1H), 7.85 – 7.82 (m, 1H), 7.74 – 7.70 (m, 1H), 7.67 – 7.59 (m, 1H), 7.58 – 7.52 (m, 2H), 2.61 (s, 3H); ^{13}C NMR (151 MHz, $CDCl_3$): δ = 145.6, 143.8, 140.4, 134.1, 133.6, 129.7, 129.1, 128.1, 125.5, 117.5, 116.6, 20.8; IR (ATR): $\tilde{\nu}$ = 2230, 1447, 1323, 1288, 1155, 1095, 733 cm^{-1}; HRMS (EI): *m/z*: calcd. for $C_{14}H_{11}NO_2S$ $[M]^{+\cdot}$: 257.05050, found: 257.05052.

2-(Bromomethyl)-4-(phenylsulfonyl)benzonitrile (S2)

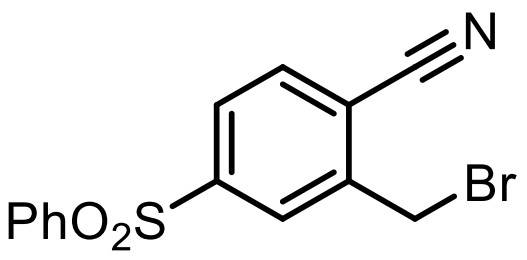

A flame-dried Schlenk flask was charged with 2-methyl-4-(phenylsulfonyl)-benzonitrile (1.34 g, 5.20 mmol), N-bromosuccinimide (1.85 g, 10.4 mmol), benzoyl peroxide (420 mg (75 wt%), 1.30 mmol) and chloroform (8 mL). The mixture was stirred at reflux temperature overnight before it was allowed to cool to room temperature and all precipitates were filtered off. The solvent was removed under reduced pressure and the residue was purified by flash chromatography (SiO_2, *n*-hexane/ethyl acetate, 4:1) which also allowed for recovery of unconverted starting material. The title compound was obtained as a colorless solid (745 mg, 43% (93% brsm)). ^1H NMR (600 MHz, $CDCl_3$): δ = 8.13 (dd, *J* = 1.8, 0.5 Hz, 1H), 7.98 – 7.92 (m, 3H), 7.79 (dd, *J* = 8.1, 0.5 Hz, 1H), 7.67 – 7.61 (m, 1H), 7.58 – 7.53 (m, 2H), 4.64 (s, 2H); ^{13}C NMR (151 MHz, $CDCl_3$): δ = 146.5, 143.0, 140.0, 134.3(4), 134.3(2), 129.9, 129.3, 128.2, 127.9, 116.9, 115.5, 28.1; IR (ATR): $\tilde{\nu}$ = 2232, 1447, 1324, 1155, 1084, 722 cm^{-1}; HRMS (EI): *m/z*: calcd. for $C_{14}H_{10}NO_2BrS$ $[M]^{+\cdot}$: 334.96103, found: 334.96147.

2-(Bromomethyl)-4-(4,4,5,5-tetramethyl-1,3,2-dioxaborolan-2-yl)benzonitrile (S3)

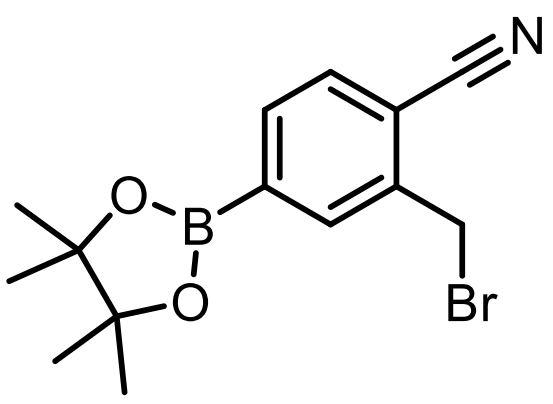

A flame-dried Schlenk flask charged with 2-methyl-4-(4,4,5,5-tetramethyl-1,3,2-dioxaborolan-2-yl)benzonitrile (2.98 g, 11.1 mmol), N-bromosuccinimide (2.29 g, 12.9 mmol), benzoyl peroxide (152 mg (75wt%), 0.63 mmol) and chloroform (18 mL). The mixture was stirred at reflux temperature for 24 h before it was allowed to cool to room temperature and all precipitates were filtered off. The filtrate was washed with water (2 x 10 mL) and brine (10 mL), dried over $MgSO_4$ and filtered, and the solvent was removed under reduced pressure. Recrystallization of the crude product from *n*-hexane/ethyl acetate gave the title compound as a pale yellow solid (2.02 g, 51%). ^1H NMR (600 MHz, $CDCl_3$): δ = 10.29 (s, 1H), 7.93 (dd, *J* = 7.5, 1.1 Hz, 1H), 7.88 – 7.86 (m, 1H), 7.83 (d, *J* = 7.5 Hz, 1H), 4.94 (s, 2H), 1.36 (s, 11H); ^{13}C NMR (151 MHz, $CDCl_3$): δ = 192.3, 138.2, 137.9, 135.5, 135.0, 132.7, 84.7, 29.7, 25.0 (the signal of C-Bpin was not detected); IR (ATR): $\tilde{\nu}$ = 2979, 2229, 1405, 1357, 1276, 1141, 965, 851 cm^{-1}; HRMS (EI): *m/z*: calcd. for $C_{14}H_{17}NO_2BrB$ $[M]^{+\cdot}$: 321.05303, found: 321.05330.

2-(Bromomethyl)-5-(trifluoromethoxy)benzonitrile (S4)

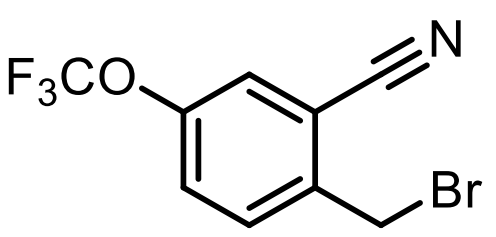

A flame-dried Schlenk flask was charged with 2-methyl-5-(trifluoromethoxy)-benzonitrile (1.00 g, 4.97mmol), N-bromosuccinimide (0.93 g, 5.23 mmol), benzoyl peroxide (122 mg (75wt%), 0.50 mmol) and chloroform (10 mL). The mixture was stirred at reflux temperature overnight before it was allowed to

cool to room temperature and all precipitates were filtered off. The solvent was removed under reduced pressure and the residue was purified by flash chromatography (SiO_2, *n*-hexane/ethyl acetate, 99:1 → 97:3) to give the title compound as a colorless solid (712 mg, 51%). ^{1}H NMR (600 MHz, $CDCl_3$): δ = 7.62 (dd, *J* = 8.6, 0.5 Hz, 1H), 7.51 (dd, *J* = 2.5, 1.0 Hz, 1H), 7.45 (ddd, *J* = 8.6, 2.6, 1.0 Hz, 1H), 4.63 (s, 2H); ^{13}C NMR (151 MHz, $CDCl_3$): δ = 148.9 (q, *J* = 2.0 Hz), 140.0, 132.5, 125.9, 125.2, 120.3 (q, *J* = 259.8 Hz), 115.6, 114.2, 28.2; ^{19}F NMR (565 MHz, $CDCl_3$): δ = –58.1; IR (ATR): ṽ = 2235, 1611, 1581, 1496, 1254, 1215, 1173, 881, 613 cm^{-1}; HRMS (ESI$^+$): *m/z*: calcd. for $C_9H_5BrF_3NONa$ [M+Na]$^+$: 301.93988, found: 301.93960.

4-(Bromomethyl)thiophene-3-carbonitrile (S5)

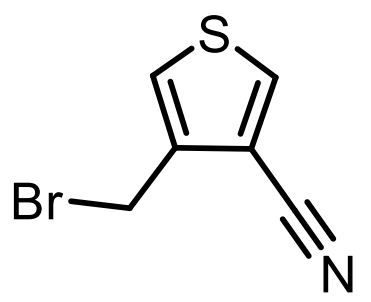

A flame-dried Schlenk flask was charged with 4-methylthiophene-3-carbonitrile (421 mg, 3.42 mmol), N-bromosuccinimide (639 mg, 3.59 mmol), benzoyl peroxide (82.9 mg (75wt%), 0.34 mmol) and chloroform (7 mL). The mixture was stirred at 85 °C (bath temperature) overnight before it was cooled to room temperature and all precipitates were filtered off. The solvent was removed under reduced pressure and the residue was purified by flash chromatography (SiO_2, *n*-hexane/ethyl acetate, 96:4 → 80:20). The resulting product was further purified by recrystallization from *n*-hexane/*tert*-butyl methyl ether to yield the title compound as a colorless solid (573 mg, 2.21 mmol, 65%). ^{1}H NMR (400 MHz, $CDCl_3$): δ = 7.95 (d, *J* = 3.1 Hz, 1H), 7.44 (dt, *J* = 3.2, 0.6 Hz, 1H), 4.55 (s, 2H); ^{13}C NMR (101 MHz, $CDCl_3$): δ = 139.4, 136.9, 126.6, 113.8, 111.8, 23.7; IR (ATR): ṽ = 3099, 2924, 2225, 1452, 1427, 1218, 1178, 870, 824 cm^{-1}; HRMS (EI): *m/z*: calcd. for C_6H_4NBrS [M]$^{+\cdot}$: 200.92425, found: 200.92440.

2-(Bromomethyl)benzaldehyde (S6). Representative Procedure A

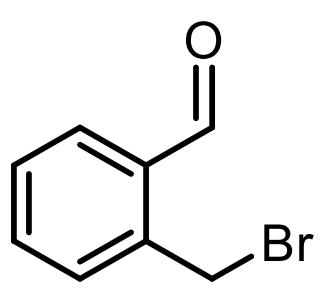

A solution of diisobutylaluminum hydride (1.0 M in hexane, 16.5 mL, 16.5 mmol) was added dropwise over 30 min to a solution of 2-cyanobenzylbromide (2.94 g, 15.0 mmol) in dichloromethane (60 mL) at 0 °C (bath temperature). The mixture was warmed to room temperature and stirred for another 2 h. The mixture was cooled to 0 °C before it was carefully poured into an ice-cold aq. HBr (6.0 M, 55 mL). The mixture was vigorously stirred for 1 h at 0 °C and was then extracted with dichloromethane (3 x 30 mL). The combined organic phases were washed with sat. aq. $NaHCO_3$ (2 x 30 mL) and brine (30 mL), dried over $MgSO_4$ and filtered, and the solvent was removed under reduced pressure to give the product as a dark brown oil which solidified over time (2.65 g, 89%). The product was used without further purification. ^{1}H NMR (400 MHz, $CDCl_3$): δ = 10.26 (s, 1H), 7.85 (dd, *J* = 7.4, 1.6 Hz, 1H), 7.61 – 7.44 (m, 3H), 4.95 (s, 2H). The data matched the literature.[2]

2-(Bromomethyl)-3-fluorobenzaldehyde (S7)

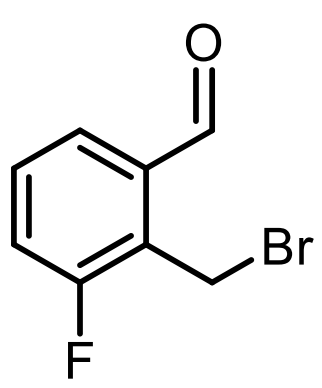

The title compound was prepared by following the representative procedure **A** using 2-(bromomethyl)-3-fluorobenzonitrile (2.99 g, 14.0 mmol). The product was obtained as a dark brown oil (2.74 g, 90%), which was used without further purification. ^{1}H NMR (600 MHz, $CDCl_3$): δ = 10.23 (d, *J* = 1.4 Hz, 1H), 7.66 (ddd, *J* = 7.6, 1.2, 0.6 Hz, 1H), 7.52 (ddd, *J* = 8.3, 7.6, 5.2 Hz, 1H), 7.34 (ddd, *J* = 9.5, 8.3, 1.3 Hz, 1H), 5.00 (d, *J* = 1.6 Hz, 2H); ^{13}C NMR (151 MHz, $CDCl_3$): δ = 191.2 (d, *J* = 3.3 Hz), 161.1 (d, *J* = 252.1 Hz), 134.7 (d, *J* = 2.5 Hz), 130.5 (d, *J* = 8.7 Hz), 129.8 (d, *J* = 3.2 Hz), 126.6 (d, *J* = 15.0 Hz), 121.3 (d, *J* = 22.9 Hz), 20.0 (d, *J* = 7.1 Hz); ^{19}F NMR (565 MHz, $CDCl_3$): δ = –116.5; IR (ATR): ṽ = 1706, 1691, 1607, 1582, 1463, 1252, 1224, 793 cm^{-1}; HRMS (EI): *m/z*: calcd. for C_8H_6OFBr [M]$^{+\cdot}$: 215.95807, found: 215.95809.

2-(Bromomethyl)-4-fluorobenzaldehyde (S8)

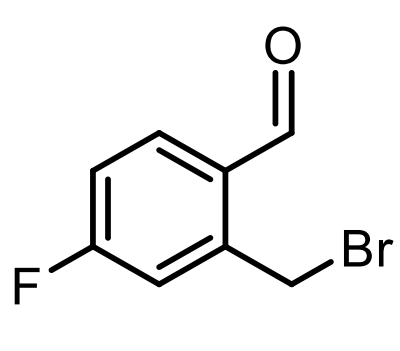

The title compound was prepared by following the representative procedure **A** using 2-(bromomethyl)-4-fluorobenzonitrile (1.15 g, 5.37 mmol). The product was obtained as a dark brown solid (1.03 g, 88%), which was used without further

purification. ^{1}H NMR (400 MHz, $CDCl_3$): δ = 10.18 (s, 1H), 7.87 (dd, *J* = 8.5, 5.7 Hz, 1H), 7.25 – 7.12 (m, 3H), 4.92 (s, 2H). The data matched the literature.[3]

2-(Bromomethyl)-5-fluorobenzaldehyde (S9)

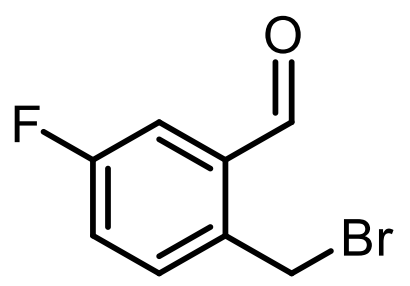

The title compound was prepared by following the representative procedure **A** using 2-(bromomethyl)-5-fluorobenzonitrile (1.14 g, 5.34 mmol). The product was obtained as a dark brown solid (1.01 g, 87%), which was used without further purification. ^{1}H NMR (400 MHz, $CDCl_3$): δ = 10.25 (d, *J* = 1.3 Hz, 1H), 7.55 (dd, *J* = 8.4, 2.8 Hz, 1H), 7.47 (dd, *J* = 8.5, 5.1 Hz, 1H), 7.30 – 7.23 (m, 1H), 4.89 (s, 2H); ^{13}C NMR (101 MHz, $CDCl_3$): δ = 190.3 (d, *J* = 1.8 Hz), 162.9 (d, *J* = 251.5 Hz), 135.4 (d, *J* = 3.7 Hz), 135.0 (d, *J* = 6.0 Hz), 133.8 (d, *J* = 7.6 Hz), 121.2 (d, *J* = 21.6 Hz), 119.3 (d, *J* = 22.5 Hz), 28.4; ^{19}F NMR (282 MHz, $CDCl_3$): δ = –110.7; IR (ATR): ṽ = 1693, 1607, 1584, 1494, 1421, 1245, 1150, 877, 605 cm^{-1}; HRMS (EI): *m/z*: calcd. for C_8H_6OFBr $[M]^{+\cdot}$: 215.95807, found: 215.95806.

2-(Bromomethyl)-6-fluorobenzaldehyde (S10)

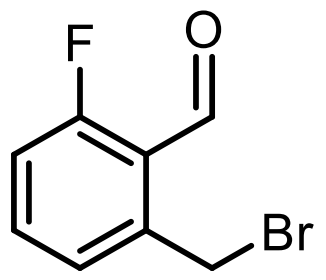

The title compound was prepared by following the representative procedure **A** using 2-(bromomethyl)-6-fluorobenzonitrile (1.06 g, 4.95 mmol). The product was obtained as a dark brown solid (0.61 g, 57%), which was used without further purification. ^{1}H NMR (400 MHz, $CDCl_3$): δ = 10.54 (d, *J* = 0.6 Hz, 1H), 7.55 (ddd, *J* = 8.4, 7.7, 5.7 Hz, 1H), 7.28 (dd, *J* = 7.7, 1.0 Hz, 1H), 7.16 (ddd, *J* = 10.6, 8.4, 1.1 Hz, 1H), 4.93 (s, 2H); ^{13}C NMR (101 MHz, $CDCl_3$): δ = 188.5 (d, *J* = 11.6 Hz), 166.4 (d, *J* = 258.6 Hz), 140.9, 135.8 (d, *J* = 10.2 Hz), 127.8 (d, *J* = 3.3 Hz), 121.4 (d, *J* = 6.4 Hz), 116.9 (d, *J* = 22.0 Hz), 29.8 (d, *J* = 2.7 Hz); ^{19}F NMR (282 MHz, $CDCl_3$): δ = –120.0; IR (ATR): ṽ = 1687, 1606, 1571, 1471, 1450, 1254, 1186, 1154, 804 cm^{-1} HRMS (EI): *m/z*: calcd. for C_8H_6OFBr $[M]^{+\cdot}$: 215.95807, found: 215.95810.

2-(Bromomethyl)-4-methoxybenzaldehyde (S11)

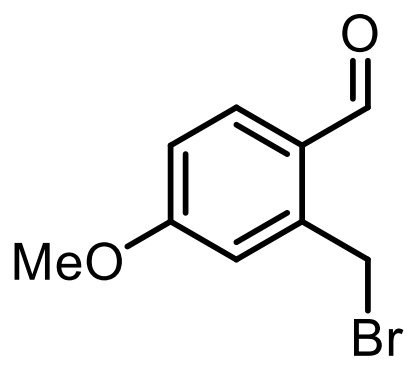

The title compound was prepared by following the representative procedure **A** using 2-(bromomethyl)-4-methoxybenzonitrile (1.29 g, 5.71 mmol). The product was obtained as a dark brown solid (1.14 g, 87%), which was used without further purification. ^{1}H NMR (400 MHz, $CDCl_3$): δ = 10.09 (s, 1H), 7.84 – 7.72 (m, 1H), 7.02 – 6.90 (m, 2H), 4.93 (s, 2H), 3.90 (s, 3H). The data matched the literature.[3]

3-(Bromomethyl)-[1,1'-biphenyl]-4-carbaldehyde (S12)

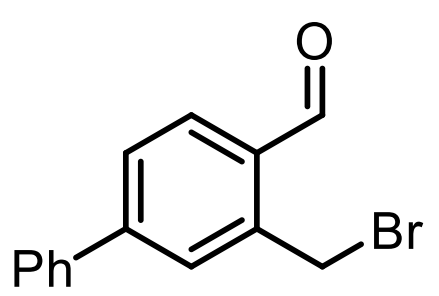

The title compound was prepared by following the representative procedure **A** using 3-(bromomethyl)-[1,1'-biphenyl]-4-carbonitrile (600 mg, 2.20 mmol).[4] The product was obtained as a dark brown oil (523 mg, 86%), which was used without further purification. ^{1}H NMR (400 MHz, $CDCl_3$): δ = 10.28 (s, 1H), 7.90 (d, *J* = 8.0 Hz, 1H), 7.73-7.70 (m, 2H), 7.65-7.62 (m, 2H), 7.51-7.41 (m, 3H), 5.01 (s, 2H). The data matched the literature.[3]

4-Bromo-2-(bromomethyl)benzaldehyde (S13)

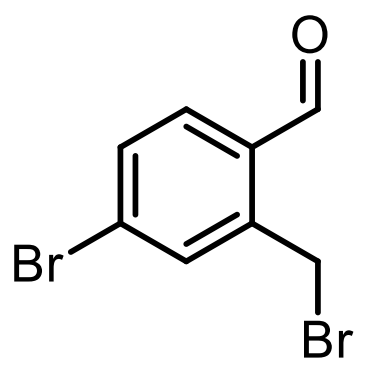

The title compound was prepared by following the representative procedure **A** using 4-bromo-2-(bromomethyl)benzonitrile (1.00 g, 3.64 mmol). The product was obtained as a dark brown solid (650 mg, 64%), which was used without further purification. ^{1}H NMR (400 MHz, $CDCl_3$): δ = 10.20 (s, 1H), 7.95 – 7.57 (m, 3H), 4.88 (s, 2H). The data matched the literature.[3]

2-(Bromomethyl)-4-(phenylsulfonyl)benzaldehyde (S14)

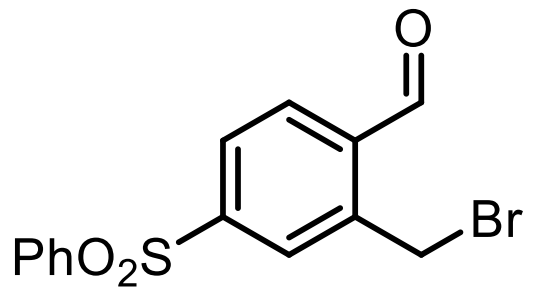

The title compound was prepared by following the representative procedure **A** using 2-(bromomethyl)-4-(phenylsulfonyl)benzonitrile **S2** (915 mg, 2.72 mmol). The product was obtained as a dark red-brown solid (754 mg, 82%), which was used without further purification. ^{1}H NMR (400 MHz, $CDCl_3$): δ = 10.31 (s, 1H), 8.10 – 8.01 (m, 2H), 8.00 – 7.91 (m, 3H), 7.68 – 7.60 (m, 1H), 7.59 – 7.50 (m, 2H), 4.91 (s, 2H); ^{13}C NMR (101 MHz, $CDCl_3$): δ = 190.6, 146.4, 140.8, 140.4, 136.1, 134.1, 133.9, 130.5, 129.8, 128.2, 128.1, 28.0; IR (ATR): ṽ = 1706, 1570, 1446, 1332, 1302, 1187, 1153, 1088, 728 cm^{-1}; HRMS (EI): *m/z*: calcd. for $C_{14}H_{11}O_3BrS$ $[M]^{+\cdot}$: 337.96069, found: 337.96087.

2-(Bromomethyl)-4-(4,4,5,5-tetramethyl-1,3,2-dioxaborolan-2-yl)benzaldehyde (S15)

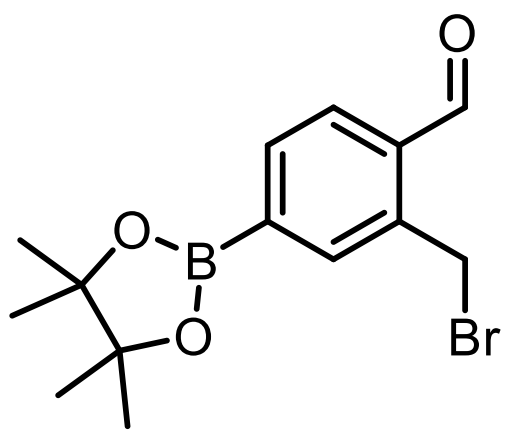

A solution of diisobutylaluminum hydride (1.0 M in hexane, 3.40 mL, 3.40 mmol) was added dropwise over 30 min to a solution of 2-(bromomethyl)-4-(4,4,5,5-tetramethyl-1,3,2-dioxaborolan-2-yl)benzonitrile **S3** (1.00 g, 3.11 mmol) in dichloromethane (10 mL) at 0 °C (bath temperature). The mixture was allowed to warm to room temperature and was stirred for another 2 h. The mixture was cooled to 0 °C and carefully poured into an ice-cold aqueous formic acid solution (8 M, 6 mL). The mixture was vigorously stirred for 2 h at 0 °C before it was extracted with dichloromethane (3 x 10 mL). The combined organic phases were washed with sat. aq. $NaHCO_3$ (2 x 10 mL) and brine (10 mL), dried over $MgSO_4$ and filtered, and the solvent was removed under reduced pressure to give the product as a dark brown solid, which was used without further purification (887 mg, 88%). ^{1}H NMR (600 MHz, $CDCl_3$): δ = 10.29 (s, 1H), 7.93 (dd, *J* = 7.5, 1.1 Hz, 1H), 7.88 – 7.86 (m, 1H), 7.83 (d, *J* = 7.5 Hz, 1H), 4.94 (s, 2H), 1.36 (s, 12H); ^{13}C NMR (151 MHz, $CDCl_3$): δ = 192.3, 138.2, 137.9, 135.5, 135.0, 132.7, 84.7, 29.7, 25.0 (the signal of C-Bpin was not detected); IR (ATR): ṽ = 2978, 1699, 1390, 1356, 1331, 1288, 1213, 1142, 1084, 852 cm^{-1}; HRMS (ESI$^+$): *m/z*: calcd. for $C_{14}H_{18}O_3BrBNa$ $[M+Na]^+$: 347.04247, found: 347.04248.

2-(Bromomethyl)-5-chlorobenzaldehyde (S16)

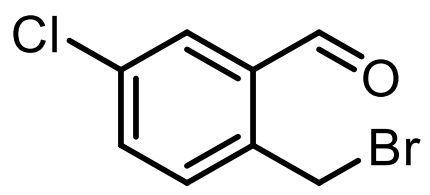

The title compound was prepared by following the representative procedure **A** using 2-(bromomethyl)-5-chlorobenzonitrile (950 mg, 4.12 mmol). The product was obtained as a dark brown solid (745 mg, 77%), which was used without further purification. ^{1}H NMR (600 MHz, $CDCl_3$): δ = 10.22 (d, *J* = 0.5 Hz, 1H), 7.82 (dd, *J* = 2.2, 0.4 Hz, 1H), 7.54 (dd, *J* = 8.2, 2.3 Hz, 1H), 7.45 – 7.39 (m, 1H), 4.88 (s, 2H); ^{13}C NMR (151 MHz, $CDCl_3$): δ = 190.5, 137.7, 135.5, 134.4, 134.1, 133.2, 133.1, 28.5; IR (ATR): ṽ = 1736, 1447, 1372, 1233, 1043 cm^{-1}; HRMS (EI): *m/z*: calcd. for C_8H_6OClBr $[M]^{+\cdot}$: 231.92852, found: 231.92847.

2-(Bromomethyl)-5-(trifluoromethoxy)benzaldehyde (S17)

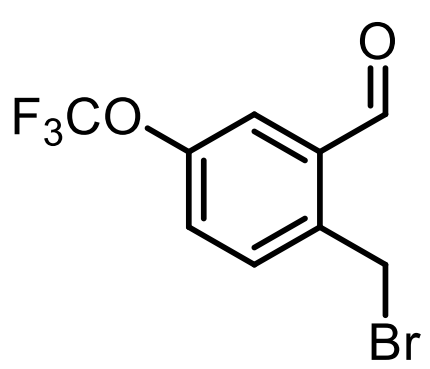

The title compound was prepared by following the representative procedure **A** using 2-(bromomethyl)-5-(trifluoromethoxy)benzonitrile **S4** (709 mg, 2.53 mmol). The product was obtained as an orange-brown liquid (638 mg, 89%), which was used without further purification. ^{1}H NMR (600 MHz, $CDCl_3$): δ = 10.26 (s, 1H), 7.69 (dd, *J* = 2.7, 1.0 Hz, 1H), 7.54 (dt, *J* = 8.4, 0.5 Hz, 1H), 7.46 – 7.37 (m, 1H), 4.90 (s, 2H); ^{13}C NMR (151 MHz, $CDCl_3$): δ = 190.2, 149.7 (q, *J* = 2.0 Hz), 137.9, 134.7, 133.5, 126.2, 124.7, 120.4 (q, *J* = 259.0 Hz), 28.1; ^{19}F NMR (585 MHz, $CDCl_3$): δ = –58.0; IR (ATR): ṽ = 1699, 1580, 1494, 1254, 1211, 1151, 885, 841, 809 cm^{-1}; HRMS (EI): *m/z*: calcd. for $C_9H_6O_2BrF_3$ $[M]^{+\cdot}$: 281.94979, found: 281.94976.

4-(Bromomethyl)thiophene-3-carbaldehyde (S18)

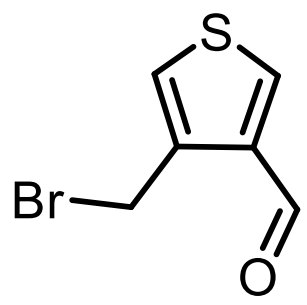

A solution of diisobutylaluminum hydride (1.0 M in hexane, 4.60 mL, 4.60 mmol) was added dropwise over 30 min to a solution of 4-(bromomethyl)thiophene-3-carbonitrile **S5** (845 mg, 4.18 mmol) in dichloromethane (12 mL) at 0 °C (bath temperature). The mixture was allowed to reach room temperature and was stirred for another 2 h. The mixture was cooled to 0 °C before it was carefully poured into an ice-cold aqueous formic acid solution (8 M, 9 mL). The mixture was vigorously stirred for 2 h at 0 °C before it was extracted with dichloromethane (3 x 10 mL). The combined organic phases were washed with sat. aq. $NaHCO_3$ (2 x 10 mL) and brine (10 mL), dried over $MgSO_4$ and filtered, and the solvent was removed under reduced pressure to give the product as a dark brown solid, which was used without further purification (808 mg, 94%). ^{1}H NMR (400 MHz, $CDCl_3$): δ = 10.01 (d, *J* = 0.8 Hz, 1H), 8.14 (d, *J* = 3.2 Hz, 1H), 7.41 (dq, *J* = 3.2, 0.8 Hz, 1H), 4.81 (s, 2H); ^{13}C NMR (101 MHz, $CDCl_3$): δ = 185.2, 140.7, 139.1, 137.8, 128.0, 26.0; IR (ATR): ṽ = 3093, 1681, 1451, 1420, 1218, 1174, 815 cm^{-1}; HRMS (EI): *m/z*: calcd. for C_6H_5OSBr $[M]^{+\cdot}$: 203.92391, found: 203.92381.

Methyl 3-(bromomethyl)furan-2-carboxylate (S19)

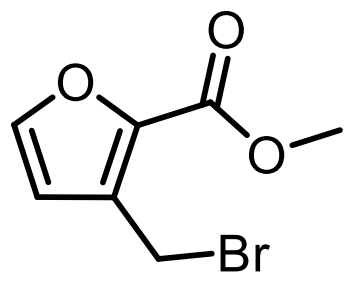

A flame-dried Schlenk flask was charged with methyl 3-methyl-2-furoate (1.50 g, 10.7 mmol), N-bromosuccinimide (2.00 g, 1.05 mmol), benzoyl peroxide (129 mg (75wt%), 0.53 mmol) and chloroform (22 mL). The mixture was stirred at reflux temperature overnight before it was allowed to cool to room temperature and all precipitates were filtered off. The solvent was removed under reduced pressure and the residue was purified by flash chromatography (SiO_2, *n*-hexane/ethyl acetate, 98:2 → 96:4) to give the title compound as a pale yellow liquid (1.33 g, 57%). ^{1}H NMR (400 MHz, $CDCl_3$): δ = 7.49 (d, *J* = 1.8 Hz, 1H), 6.60 (d, *J* = 1.8 Hz, 1H), 4.70 (s, 2H), 3.93 (s, 3H); ^{13}C NMR (101 MHz, $CDCl_3$): δ = 159.3, 145.5, 140.1, 131.3, 114.2, 52.2, 22.4; IR (ATR): ṽ = 1713, 1600, 1493, 1440, 1410, 1305, 1199, 1136, 1063, 894 cm^{-1}; HRMS (ESI$^+$): *m/z*: calcd. for $C_7H_7BrO_3Na$ $[M+Na]^+$: 240.94708, found: 240.94705.

3-(Bromomethyl)furan-2-carbaldehyde (S20)

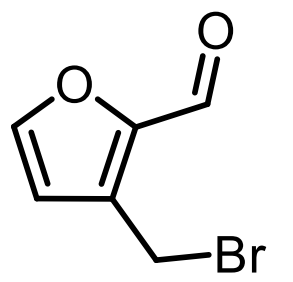

A solution of diisobutylaluminum hydride (1.0 M in hexane, 7.70 mL, 7.70 mmol) was added dropwise over 10 min to a solution of methyl 3-(bromomethyl)furan-2-carboxylate **S19** (844 mg, 3.85 mmol) in dichloromethane (17 mL) at –95 °C (bath temperature). The resulting mixture was then stirred at this temperature for another 2 h. The cooling bath was removed and the mixture was diluted with dichloromethane (5 mL) and aq. HBr (1 M, 10 mL). After vigorous stirring for 10 min, the organic phase was separated and the aqueous phase was extracted with dichloromethane (3 x 20 mL). The combined organic phases were washed with water (20 mL) and brine (20 mL), dried over $MgSO_4$ and filtered into a round bottom flask.

Dess-Martin periodinane (4.93 g, 11.6 mmol) and $NaHCO_3$ (1.62 g, 19.2 mmol) were added and the resulting mixture was stirred at room temperature for 2 h. The reaction was diluted with sat. aq. $Na_2S_2O_3$ solution and vigorously stirred for 20 min. The organic phase was separated and the aqueous phase extracted with dichloromethane (3 x 15 mL). The combined organic phases were washed with water (30 mL) and brine (30 mL), dried over $MgSO_4$ and filtered, and the solvent was removed under reduced pressure (***Note***: *do not dry completely! The product quickly starts decomposing once concentrated but is stable in solution even at high concentrations*). The crude mixture was purified by flash chromatography (SiO_2, *n*-pentane/diethyl ether, 95:5). Before removing the solvent, a small amount of C_6D_6 (~2 mL) and trimethoxybenzene (18.1 mg, 0.11 mmol) were added to the product solution. The volatile components were carefully removed under reduced pressure. The product was obtained as a stock solution in C_6D_6 (250 mg, 34% / determined with internal standard via ^{1}H NMR). (***Note:*** *NMR analysis of the isolated product was possible by removing the solvent and quickly redissolving the product in $CDCl_3$ to avoid decomposition. The obtained stock solution in C_6D_6 was frozen and the glass stored in the freezer; no signs of decomposition were noticed after >3 months*). ^{1}H NMR (600 MHz, $CDCl_3$): δ = 9.86 (d, *J* = 0.6 Hz, 1H), 7.59 (d, *J* = 1.8 Hz, 1H), 6.68 (dd, *J* = 1.8, 0.6 Hz, 1H), 4.68

(s, 2H); ^{13}C NMR (151 MHz, $CDCl_3$): δ = 179.4, 148.0, 147.3, 132.0, 115.0, 21.0; IR (ATR): ṽ = 1678, 1588, 1479, 1428, 1376, 1234, 1153, 764 cm^{-1}; HRMS (EI): *m/z*: calcd. for $C_6H_5O_2Br$ $[M]^{+\cdot}$: 187.94676, found: 187.94688.

3-Bromo-2,2-dimethylpropanal (S21)

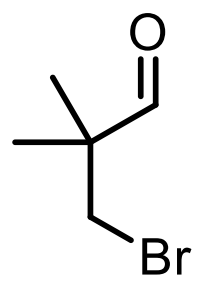

A mixture of pyridinium chlorochromate (2.59 g, 12.0 mmol) and silica gel (2.50 g) was suspended in dichloromethane (20.0 mL). 3-Bromo-2,2-dimethyl-1-propanol (1.00 g, 6.00 mmol) was added and the mixture was stirred at room temperature for 16 h. The solid was filtered off and carefully rinsed with diethylether (80 mL). The solvent was removed under reduced pressure to give the product as a light brown oil, which was used without further purification (941 mg, 95%). ^{1}H NMR (400 MHz, $CDCl_3$): δ = 9.49 (s, 1H), 3.46 (s, 2H), 1.22 (s, 6H). The data matched the literature.[5]

3-(Bromomethyl)oxetane-3-carbaldehyde (S22)

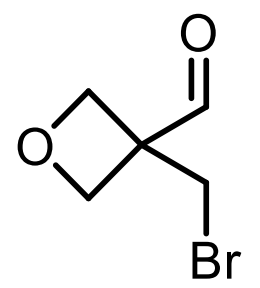

(3-(Bromomethyl)oxetan-3-yl)methanol (1.09 g, 6.00 mmol) was added to a suspension of Dess-Martin periodinane (5.09 g, 12.0 mmol) in dichloromethane (40 mL) at 0 °C (bath temperature). The mixture was allowed to warm to room temperature and stirred for 2 h. Sat. aq. $NaHCO_3$ (20 mL) and sat. aq. $Na_2S_2O_3$ (20 mL) were added and the resulting mixture was vigorously stirred for 30 min. The organic phase was separated and the aqueous phase was extracted with dichloromethane (3 x 10 mL). The combined organic phases were washed with sat. aq. $Na_2S_2O_3$ (10 mL) and brine (10 mL), dried over $MgSO_4$ and filtered, and the solvent was removed under reduced pressure. The residue was purified by flash chromatography (SiO_2, *n*-hexane/ethyl acetate, 1.5:1) to give the title compound as a colorless oil (510 mg, 47%). ^{1}H NMR (400 MHz, $CDCl_3$): δ = 9.90 (s, 1H), 4.83 (d, *J* = 6.8 Hz, 2H), 4.56 (d, *J* = 6.8 Hz, 2H), 3.87 (s, 2H). The data matched the literature.[6]

Methyl (2*E*,4*E*)-5-((*tert*-butoxycarbonyl)(4-((*tert*-butyldimethylsilyl)oxy)butyl)amino)penta-2,4-dienoate (S23)

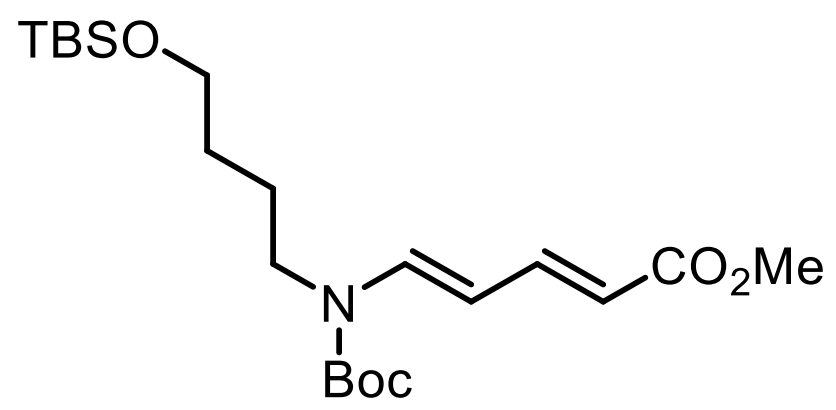

Methyl (2*E*,4*E*)-5-((*tert*-butoxycarbonyl)amino)penta-2,4-dienoate **1a** (352 mg, 1.55 mmol) was added in portions to a suspension of sodium hydride (56.7 mg, 2.36 mmol) in THF (9.0 mL) at 0 °C (bath temperature). After complete addition, the mixture was stirred at 0 °C for 10 min before (4-bromobutoxy)(*tert*-butyl)dimethylsilane (828 mg, 3.10 mmol) and tetrabutylammonium iodide (57.2 mg, 0.15 mmol) were added. The mixture was stirred at 65 °C (bath temperature) for 3 h. After cooling to 0 °C, sat. aq. $NaHCO_3$ (5 mL) was carefully added, followed by ethyl acetate (20 mL) and water (10 mL). The mixture was vigorously stirred for 5 min, the organic phase was separated and the aqueous layer was extracted with ethyl acetate (3 x 20 mL). The combined organic phases were washed with brine (20 mL), dried over $MgSO_4$ and filtered, and the solvent was removed under reduced pressure. The residue was purified by flash chromatography (SiO_2, *n*-hexane/ethyl acetate, 20:1 → 4:1) to give the title compound as a yellow oil (517 mg, 81%). ^{1}H NMR (400 MHz, $CDCl_3$): δ = 7.42 (d, *J* = 13.0 Hz, 1H), 7.33 (ddd, *J* = 15.2, 11.2, 0.7 Hz, 1H), 5.72 (d, *J* = 15.2 Hz, 1H), 5.60 (dd, *J* = 14.0, 11.2 Hz, 1H), 3.73 (s, 3H), 3.63 (t, *J* = 6.1 Hz, 2H), 3.56 (t, *J* = 7.5 Hz, 2H), 1.69 – 1.52 (m, 4H), 1.51 (s, 9H), 0.88 (s, 9H), 0.04 (s, 6H); ^{13}C NMR (101 MHz, $CDCl_3$): δ = 168.2, 152.4, 145.5, 138.6, 115.4, 106.2, 82.6, 62.8, 51.4, 44.2, 30.2, 28.3, 26.1, 23.9, 18.4, -5.1; IR (ATR): ṽ = 2951, 1712, 1620, 1388, 1333, 1237, 1129, 996, 836 cm^{-1} HRMS (ESI^+): *m/z*: calcd. for $C_{21}H_{39}NO_5SiNa$ $[M+Na]^+$: 436.24897, found: 436.24945.

Methyl (2*E*,4*E*)-5-((*tert*-butoxycarbonyl)(4-hydroxybutyl)amino)penta-2,4-dienoate (S24)

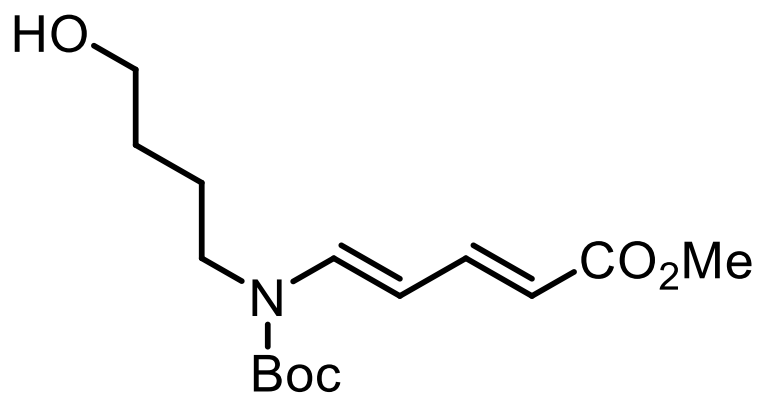

A solution of TBAF in THF (1.0 M, 1.60 mL, 1.60 mmol) was added dropwise to a solution of methyl (2*E*,4*E*)-5-((*tert*-butoxycarbonyl)(4-((*tert*-butyldimethylsilyl)oxy)butyl)amino)penta-2,4-dienoate **S23** (517 mg, 1.25 mmol) in THF (5.0 mL) at 0 °C (bath temperature). The mixture was stirred at room temperature for 30 min before water (10 mL) and *tert*-butyl methyl ether (20 mL) were added. The organic phase was separated and the aqueous layer extracted with *tert*-butyl methyl ether (2 x 20 mL). The combined organic phases were washed with brine (20 mL), dried over $MgSO_4$ and filtered, and the solvent was removed under reduced pressure. The residue was purified by flash chromatography (SiO_2, *n*-hexane/ethyl acetate, 4:1 → 1:1) to give the title compound as a pale yellow oil (315 mg, 84%). ^{1}H NMR (600 MHz, $CDCl_3$): δ = 7.40 (s, 1H), 7.33 (dd, *J* = 15.1, 11.2 Hz, 1H), 5.73 (d, *J* = 15.2 Hz, 1H), 5.61 (dd, *J* = 14.0, 11.2 Hz, 1H), 3.72 (s, 3H), 3.68 (t, *J* = 6.3 Hz, 2H), 3.58 (s, 2H), 1.70 – 1.63 (m, 2H), 1.61 – 1.56 (m, 2H), 1.51 (s, 9H); ^{13}C NMR (151 MHz, $CDCl_3$): δ = 168.2, 152.4, 145.3, 138.5, 115.6, 106.2, 82.7, 62.5, 51.5, 43.8, 29.8, 28.3, 23.7; IR (ATR): ṽ = 3464, 2947, 1711, 1619, 1390, 1242, 1134, 1071 cm^{-1} HRMS (ESI$^+$): *m/z*: calcd. for $C_{15}H_{25}NO_5Na$ [M+Na]$^+$: 322.16249, found: 322.16220.

Methyl (2*E*,4*E*)-5-((*tert*-butoxycarbonyl)(3-((*tert*-butyldimethylsilyl)oxy)propyl)amino)penta-2,4-dienoate (S25).

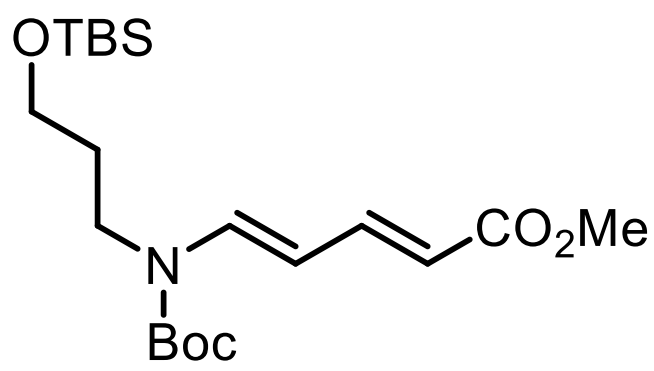

Methyl (2*E*,4*E*)-5-((*tert*-butoxycarbonyl)amino)penta-2,4-dienoate **1a** (217 mg, 1.00 mmol) was added in portions to a suspension of sodium hydride (36.0 mg, 1.50 mmol) in THF (5.0 mL) at 0 °C (bath temperature). After complete addition, the mixture was stirred at 0 °C for another 10 min. (3-Bromopropoxy)-*tert*-butyldimethylsilane (508 mg, 2.00 mmol) and tetrabutylammonium iodide (36.9 mg, 0.10 mmol) were added and the mixture was stirred at 65 °C (bath temperature) for 3 h. After cooling to 0 °C, sat. aq. $NaHCO_3$ (5 mL) was carefully added, followed by addition of ethyl acetate (20 mL) and water (10 mL). The mixture was vigorously stirred for 5 min, the organic phase was separated and the aqueous layer extracted with ethyl acetate (3 x 20 mL). The combined organic phases were washed with brine (20 mL), dried over $MgSO_4$ and filtered, and the solvent was removed under reduced pressure. The residue was purified by flash chromatography (SiO_2, *n*-hexane/ *tert*-butyl methyl ether, 20:1 → 10:1) to give the title compound as a pale yellow oil (307 mg, 77%). ^{1}H NMR (400 MHz, $CDCl_3$): δ = 7.42 (d, *J* = 13.5 Hz, 1H), 7.34 (ddd, *J* = 15.1, 11.3, 0.6 Hz, 1H), 5.79 – 5.63 (m, 2H), 3.73 (s, 3H), 3.67 – 3.59 (m, 4H), 1.82 – 1.72 (m, 2H), 1.51 (s, 9H), 0.91 (s, 9H), 0.06 (s, 6H); ^{13}C NMR (101 MHz, $CDCl_3$): δ = 168.2, 152.4, 145.5, 138.7, 115.4, 106.3, 82.6, 60.6, 51.4, 41.6, 30.5, 28.3, 26.1, 18.4, -5.2; IR (ATR): ṽ = 2930, 1713, 1621, 1388, 1335, 1239, 1131, 836 cm^{-1} HRMS (ESI$^+$): *m/z*: calcd. for $C_{20}H_{37}NO_5SiNa$ [M+Na]$^+$: 422.23332, found: 422.23283.

Methyl (2*E*,4*E*)-5-((*tert*-butoxycarbonyl)(3-hydroxypropyl)amino)penta-2,4-dienoate (S26).

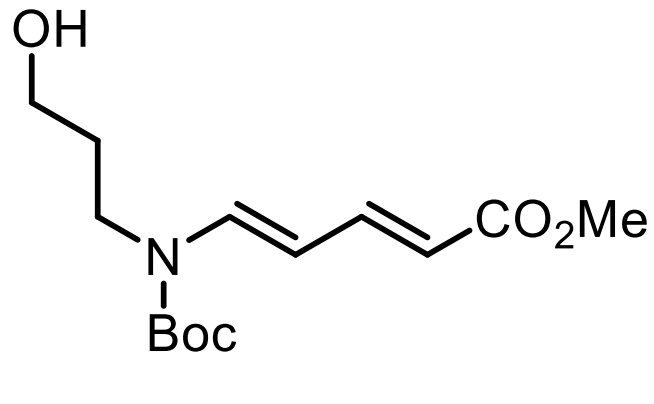

A solution of TBAF in THF (1.0 M, 1.15 mL, 1.15 mmol) was added dropwise to a solution of methyl (2*E*,4*E*)-5-((*tert*-butoxycarbonyl)(3-((*tert*- butyldimethylsilyl)oxy)propyl)amino)penta-2,4-dienoate **S25** in THF (4.0 mL) at 0 °C (bath temperature). The mixture was stirred at room temperature for 30 min before water (10 mL) and *tert*-butyl methyl ether (20 mL) were added. The organic phase was separated and the aqueous layer extracted with *tert*-butyl methyl ether (2 x 20 mL). The combined organic phases were washed with brine (20 mL), dried over $MgSO_4$ and filtered, and the solvent was removed under reduced pressure. The residue was purified by flash chromatography (SiO_2, *n*-hexane/ethyl acetate, 2:1) to give the title compound as an orange-yellow oil (198 mg, 79%). ^{1}H NMR (400 MHz, $CDCl_3$): δ = 7.43 – 7.28 (m, 2H), 5.75 (d, *J* = 15.1 Hz, 1H), 5.67 (dd, *J* = 14.0, 11.2 Hz, 1H), 3.73 (s, 3H), 3.71 (br, 2H), 3.57 (br, 2H), 2.88 (br, 1H), 1.78 (p, *J* = 6.1 Hz, 2H), 1.52 (s, 9H); ^{13}C NMR (101 MHz, $CDCl_3$): δ = 168.1, 153.2,

145.0, 138.0, 116.1, 106.7, 83.4, 58.8, 51.5, 40.0, 29.7, 28.2; IR (ATR): ṽ = 3476, 2950, 1709, 1615, 1435, 1387, 1334, 1239, 1127, 1060, 995 cm^{-1} HRMS (ESI$^+$): *m/z*: calcd. for $C_{14}H_{23}NO_5Na$ [M+Na]$^+$: 308.14684, found: 308.14692.

***tert*-Butyl ((1*E*,3*E*)-4-phenylbuta-1,3-dien-1-yl)carbamate (S27)**

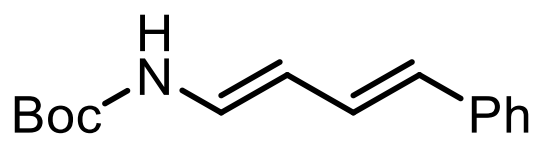

A flame-dried Schlenk flask was charged with (2*E*,4*E*)-5-phenylpenta-2,4-dienoic acid (580 mg, 3.33 mmol) and $SOCl_2$ (2.42 mL, 33.3 mmol) and the resulting mixture was stirred at 60 °C (bath temperature) for 3 h. The excess $SOCl_2$ was removed *in vacuo* to leave the acid chloride as a colorless solid, which was directly used in the next step.

This crude material was dissolved in toluene (8 mL) and a solution of NaN_3 (650 mg, 10.0 mmol) and tetrabutylammonium bromide (53.7 mg, 0.17 mmol) in water (8 mL) was added. The mixture was vigorously stirred for 30 min at room temperature. The organic phase was separated and the aqueous layer was extracted with toluene (3 x 15 mL). The combined organic phases were washed with sat. aq. $NaHCO_3$ (20 mL) and brine (20 mL) and were then dried over $MgSO_4$ and filtered.

The volume of the filtrate was reduced to ca. 20 mL under reduced pressure (***Caution:*** *do not dry completely!*). Dry *tert*-butanol (3.12 mL, 33.3 mmol) was added and the mixture stirred at 100 °C (bath temperature) for 2 h. The solvent was removed under reduced pressure and the residue was purified by recrystallization from *n*-hexane/ethyl acetate to give title compound as a pale yellow solid (566 mg, 69%). ^{1}H NMR (400 MHz, CD_2Cl_2): δ = 7.37 – 7.33 (m, 2H), 7.31 – 7.24 (m, 2H), 7.22 – 7.13 (m, 1H), 6.84 (t, *J* = 12.6 Hz, 1H), 6.76 (dd, *J* = 15.6, 10.7 Hz, 1H), 6.55 (d, *J* = 12.5 Hz, 1H), 6.37 (d, *J* = 15.6 Hz, 1H), 5.87 – 5.73 (m, 1H), 1.48 (s, 9H); ^{13}C NMR (101 MHz, CD_2Cl_2): δ = 152.7, 138.3, 128.9, 128.2(4), 128.1(6), 127.6, 127.1, 126.1, 110.9, 81.2, 28.4; IR (ATR): ṽ = 3311, 2979, 1669, 1648, 1503, 1368, 1252, 1148, 975 cm^{-1}; HRMS (EI): *m/z*: calcd. for $C_{15}H_{19}NO_2$ [M]$^{+\cdot}$: 245.14103, found: 245.14113.

Substrates

Representative Procedure B: Methyl (2*E*,4*E*)-5-((*tert*-butoxycarbonyl)(2-formylbenzyl)amino)-penta-2,4-dienoate (7a).

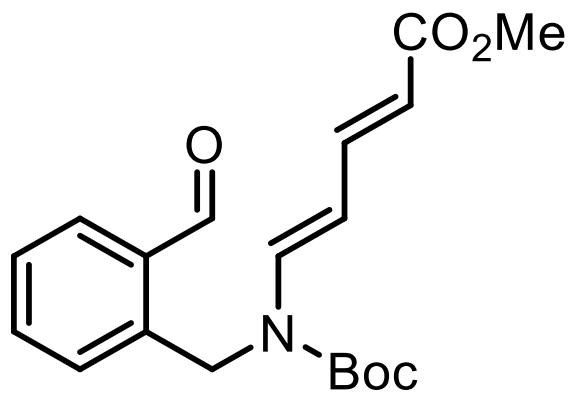

Methyl (2*E*,4*E*)-5-((*tert*-butoxycarbonyl)amino)penta-2,4-dienoate **1a** (682 mg, 3.00 mmol) was added in portions to a suspension of sodium hydride (108 mg, 4.50 mmol) in N,N-dimethylformamide (22 mL) at 0 °C (bath temperature). After complete addition, the mixture was stirred at 0 °C for another 10 min. 2-(Bromomethyl)benzaldehyde **S6** (1.19 g, 6.00 mmol) and tetrabutylammonium iodide (110.8 mg, 0.30 mmol) were added and the mixture was stirred at room temperature overnight. The mixture was cooled to 0 °C and sat. aq. $NaHCO_3$ (15 mL) was carefully added, followed by addition of ethyl acetate (20 mL) and water (10 mL). The mixture was vigorously stirred for 5 min, the organic phase was separated, and the aqueous phase was extracted with ethyl acetate (3 x 20 mL). The combined organic phases were washed with aq. sat. LiCl (5 x 10 mL), dried over $MgSO_4$ and filtered, and the solvent was removed under reduced pressure. The residue was purified by flash chromatography (SiO_2, *n*-hexane/ethyl acetate, 4:1) to afford the title compound as a pale brown solid (940 mg, 91%). ^1H NMR (400 MHz, $CDCl_3$): δ = 10.16 (d, *J* = 0.7 Hz, 1H), 7.86 (dd, *J* = 7.4, 1.7 Hz, 1H), 7.61 (br, 1H), 7.55 (td, *J* = 7.5, 1.6 Hz, 1H), 7.49 (td, *J* = 7.4, 1.3 Hz, 1H), 7.30 (dd, *J* = 15.3, 11.3 Hz, 1H), 7.10 (d, *J* = 7.6 Hz, 1H), 5.58 (d, *J* = 15.2 Hz, 1H), 5.37 (dd, *J* = 13.8, 11.3 Hz, 1H), 5.25 (s, 2H), 3.68 (s, 3H), 1.47 (br, 9H); ^{13}C NMR (101 MHz, $CDCl_3$): δ = 193.9, 167.9, 152.5, 144.6, 138.5, 138.3, 136.0, 134.3, 133.2, 127.6, 125.7, 116.3, 107.5, 83.1, 51.4, 46.4, 28.2; IR (ATR): ṽ = 2979, 1710, 1694, 1622, 1434, 1384, 1331, 1241, 1135, 996 cm^{-1} HRMS (ESI$^+$): *m/z*: calcd. for $C_{19}H_{23}NO_5Na$ $[M+Na]^+$: 368.14684, found: 368.14710.

Representative Procedure C: Methyl (2*E*,4*E*)-5-((*tert*-butoxycarbonyl)(4-fluoro-2-formylbenzyl)-amino)penta-2,4-dienoate (7e).

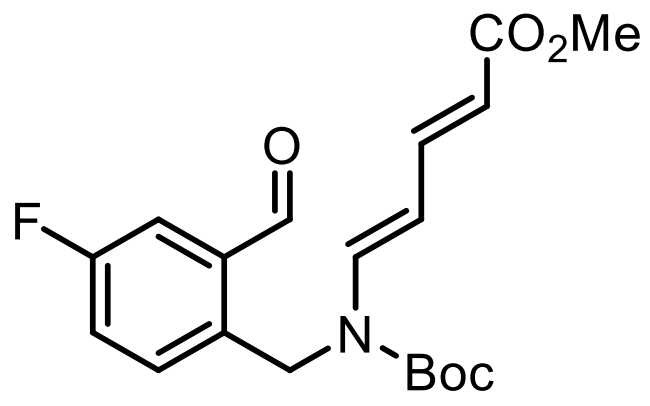

Methyl (2*E*,4*E*)-5-((*tert*-butoxycarbonyl)amino)penta-2,4-dienoate **1a** (273 mg, 1.20 mmol) was added in portions to a suspension of sodium hydride (43.2 mg, 1.80 mmol) in N,N-dimethylformamide (9.0 mL) at 0 °C (bath temperature). After complete addition, the mixture was stirred at 0 °C for another 10 min. 2-(Bromomethyl)-5-fluorobenzaldehyde **S9** (521 mg, 2.40 mmol) and tetrabutylammonium iodide (44.3 mg, 0.12 mmol) were added and the mixture was stirred at room temperature overnight. The reaction was cooled to 0 °C and more sodium hydride (14.4 mg, 0.6 mmol) was added. After stirring at 0 °C for another 10 min, additional (bromomethyl)-5-fluorobenzaldehyde **S9** (130 mg, 0.60 mmol) was introduced. The mixture was stirred at room temperature for 5 h before the reaction was carefully quenched at 0 °C with sat. aq. $NaHCO_3$ (10 mL), followed by addition of ethyl acetate (10 mL) and water (5 mL). The mixture was vigorously stirred for 5 min, the organic phase was separated, and the aqueous layer was extracted with ethyl acetate (3 x 10 mL). The combined organic phases were washed with aq. sat. LiCl (5 x 5 mL), dried over $MgSO_4$ and filtered, and the solvent was removed under reduced pressure. The residue was purified by flash chromatography (SiO_2, *n*-hexane/ethyl acetate, 4:1) to give the title compound as a pale yellow solid (376 mg, 86%). ^1H NMR (600 MHz, $CDCl_3$): δ = 10.12 (s, 1H), 7.60 (br, 1H), 7.56 (dd, *J* = 8.2, 2.8 Hz, 1H), 7.33 – 7.23 (m, 2H), 7.08 (dd, *J* = 8.7, 5.0 Hz, 1H), 5.60 (d, *J* = 15.3 Hz, 1H), 5.36 (t, *J* = 12.5 Hz, 1H), 5.19 (s, 2H), 3.69 (s, 3H), 1.49 (br, 9H); ^{13}C NMR (151 MHz, $CDCl_3$): δ = 192.2, 167.9, 162.0 (d, *J* = 248.9 Hz), 152.4, 144.4, 138.1, 134.6, 134.2, 127.9 (d, *J* = 7.2 Hz), 121.6 (d, *J* = 21.9 Hz), 121.3 (d, *J* = 21.1 Hz), 116.6, 107.5, 83.4, 51.5, 45.8, 28.2; ^{19}F NMR (585 MHz, $CDCl_3$): δ = –114.4; IR (ATR): ṽ = 2980, 1669, 1621, 1495, 1384, 1240, 1132, 995, 857 cm^{-1} HRMS (ESI$^+$): *m/z*: calcd. for $C_{19}H_{22}NO_5FNa$ $[M+Na]^+$: 386.13742, found: 386.13736.

Methyl (2*E*,4*E*)-5-(((benzyloxy)carbonyl)(2-formylbenzyl)amino)penta-2,4-dienoate (7b).

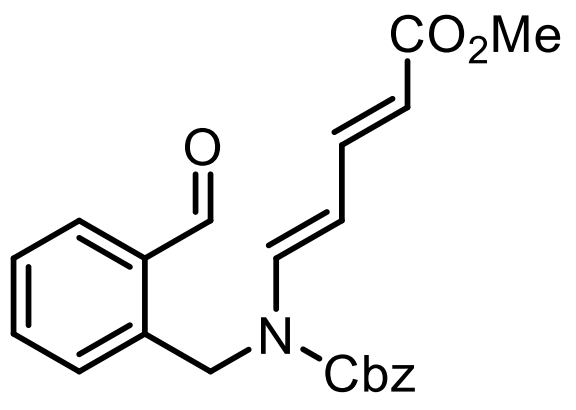

The title compound was prepared according to the representative procedure **B** using methyl (2*E*,4*E*)-5-(((benzyloxy)carbonyl)amino)penta-2,4-dienoate **1b** (261 mg, 1.00 mmol) and 2-(bromomethyl)benzaldehyde **S6** (398 mg, 2.00 mmol). The crude material was purified by flash chromatography (SiO_2, *n*-hexane/ethyl acetate, 4:1) to give the product as an off-white solid (325 mg, 86%). ^{1}H NMR (600 MHz, [D$_6$]-DMSO, 383 K): δ = 10.23 (s, 1H), 7.95 (dd, *J* = 7.5, 1.5 Hz, 1H), 7.68 (d, *J* = 14.0 Hz, 1H), 7.59 (td, *J* = 7.6, 1.5 Hz, 1H), 7.52 (td, *J* = 7.5, 1.2 Hz, 1H), 7.47 – 7.27 (m, 6H), 7.13 (dd, *J* = 7.8, 1.4 Hz, 1H), 5.83 – 5.75 (m, 1H), 5.69 (d, *J* = 15.2 Hz, 1H), 5.32 (s, 2H), 5.26 (s, 2H), 3.63 (s, 3H); ^{13}C NMR (151 MHz, [D$_6$]-DMSO, 383 K): δ = 192.9, 166.0, 152.8, 143.5, 137.2, 135.2, 133.2, 133.0, 132.9, 127.8, 127.5, 127.1, 126.9, 125.1, 116.1, 108.1, 67.6, 50.1, 45.1; IR (ATR): ṽ = 2950, 1713, 1696, 1626, 1399, 1225, 1139, 994 cm^{-1}; HRMS (ESI$^+$): *m/z*: calcd. for $C_{22}H_{21}NO_5Na$ [M+Na]$^+$: 402.13119, found: 402.13104.

Methyl (2*E*,4*E*)-5-((*tert*-butoxycarbonyl)(2-fluoro-6-formylbenzyl)amino)penta-2,4-dienoate (7c).

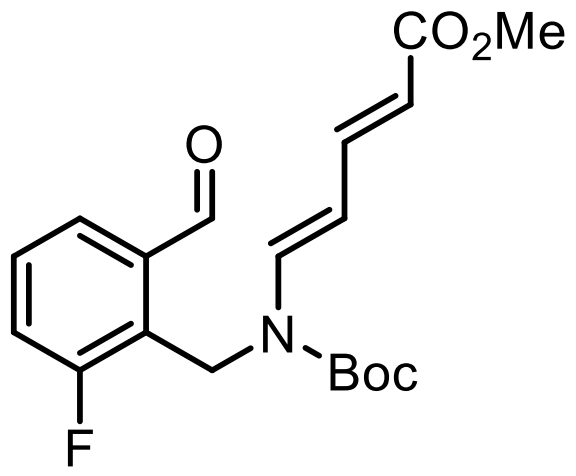

The title compound was prepared according to the representative procedure **C** using methyl (2*E*,4*E*)-5-((*tert*-butoxycarbonyl)amino)penta-2,4-dienoate **1a** (182 mg, 0.80 mmol) and 2-(bromomethyl)-3-fluorobenzaldehyde **S7** (434 mg, 2.00 mmol). The crude material was purified by flash chromatography (SiO_2, *n*-hexane/ethyl acetate, 4:1) to give the product as a yellow liquid (246 mg, 85%). ^{1}H NMR (600 MHz, $CDCl_3$): δ = 10.20 (d, *J* = 1.7 Hz, 1H), 7.66 (dd, *J* = 7.6, 1.3 Hz, 1H), 7.46 (td, *J* = 7.9, 4.9 Hz, 1H), 7.38 (d, *J* = 14.0 Hz, 1H), 7.29 – 7.26 (m, 1H), 7.23 (ddd, *J* = 15.2, 11.1, 0.7 Hz, 1H), 5.60 (dt, *J* = 15.2, 0.6 Hz, 1H), 5.55 (dd, *J* = 14.0, 11.1 Hz, 1H), 5.34 (s, 2H), 3.69 (s, 3H), 1.49 (s, 9H); ^{13}C NMR (151 MHz, $CDCl_3$): δ = 192.4 (d, *J* = 3.3 Hz), 168.0, 161.7 (d, *J* = 250.7 Hz), 152.4, 144.8, 138.0, 136.2 (d, *J* = 3.0 Hz), 129.5, 129.5, 125.5 (d, *J* = 11.7 Hz), 121.6 (d, *J* = 24.2 Hz), 116.2, 107.4 (d, *J* = 1.8 Hz), 83.3, 51.4, 39.4, 28.2; ^{19}F NMR (282 MHz, $CDCl_3$): δ = –115.1; IR (ATR): ṽ = 2980, 1698, 1621, 1465, 1385, 1330, 1242, 1132, 995 cm^{-1}; HRMS (EI): *m/z*: calcd. for $C_{19}H_{22}NO_5F$ [M]$^{+\cdot}$: 363.14765, found: 363.14788.

Methyl (2*E*,4*E*)-5-((*tert*-butoxycarbonyl)(5-fluoro-2-formylbenzyl)amino)penta-2,4-dienoate (7d).

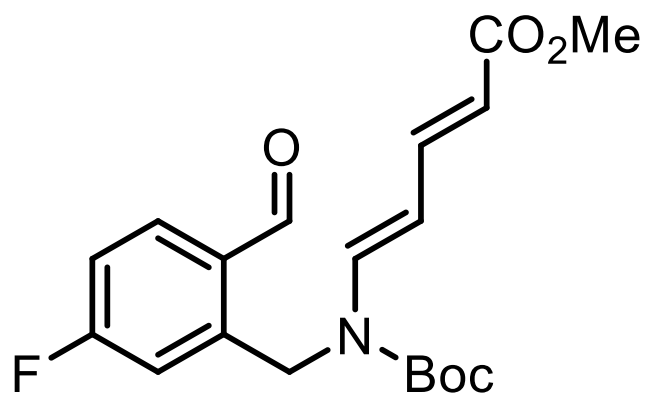

The title compound was prepared according to the representative procedure **C** using methyl (2*E*,4*E*)-5-((*tert*-butoxycarbonyl)amino)penta-2,4-dienoate **1a** (227 mg, 1.00 mmol) and 2-(bromomethyl)-4-fluorobenzaldehyde **S8** (563 mg, 2.50 mmol). The crude material was purified by flash chromatography (SiO_2, toluene/*tert*-butyl methyl ether, 30:1) to give the product as an off-white solid (187 mg, 51%). ^{1}H NMR (600 MHz, $CDCl_3$): δ = 10.10 (d, *J* = 0.7 Hz, 1H), 7.88 (dd, *J* = 8.4, 5.6 Hz, 1H), 7.58 (br, 1H), 7.30 (dd, *J* = 15.2, 11.1 Hz, 1H), 7.16 (td, *J* = 8.0, 2.5 Hz, 1H), 6.79 (d, *J* = 9.8 Hz, 1H), 5.61 (d, *J* = 15.2 Hz, 1H), 5.35 (t, *J* = 12.5 Hz, 1H), 5.25 (s, 2H), 3.70 (s, 3H), 1.50 (br, 9H); ^{13}C NMR (151 MHz, $CDCl_3$): δ = 192.1, 167.9, 166.5 (d, *J* = 257.8 Hz), 152.3, 144.3, 142.6, 138.7 (d, *J* = 9.9 Hz), 137.8, 129.8, 116.8, 114.7 (d, *J* = 22.0 Hz), 113.7 (d, *J* = 24.0 Hz), 107.6, 83.5, 51.5, 46.2, 28.2; ^{19}F NMR (585 MHz, $CDCl_3$): δ = –102.0; IR (ATR): ṽ = 2981, 1700, 1625, 1584, 1435, 1384, 1242, 1135, 966 cm^{-1}; HRMS (ESI$^+$): *m/z*: calcd. for $C_{19}H_{22}NO_5FNa$ [M+Na]$^+$: 386.13742, found: 386.13716.

Methyl (2*E*,4*E*)-5-((*tert*-butoxycarbonyl)(3-fluoro-2-formylbenzyl)amino)penta-2,4-dienoate (7f).

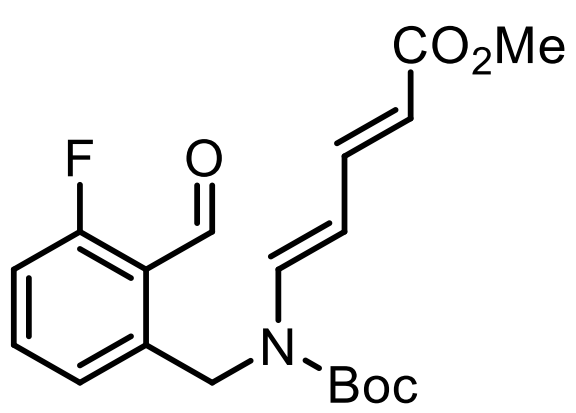

The title compound was prepared according to the representative procedure **C** using methyl (2*E*,4*E*)-5-((*tert*-butoxycarbonyl)amino)penta-2,4-dienoate **1a** (69.0 mg, 0.30 mmol) and 2-(bromomethyl)-6-fluorobenzaldehyde **S10** (165 mg, 0.75 mmol). The crude material was purified by flash chromatography (SiO_2, *n*-hexane/ethyl acetate, 5:1) to give the product as a pale brown solid (70.0 mg, 63%). ^{1}H NMR (600 MHz, $CDCl_3$):

δ = 10.59 (d, *J* = 0.7 Hz, 1H), 7.59 (br, 1H), 7.53 (td, *J* = 8.1, 5.6 Hz, 1H), 7.30 (dd, *J* = 15.2, 11.1 Hz, 1H), 7.12 (t, *J* = 9.4 Hz, 1H), 6.87 (d, *J* = 7.9 Hz, 1H), 5.60 (d, *J* = 15.2 Hz, 1H), 5.35 (t, *J* = 12.6 Hz, 1H), 5.18 (s, 2H), 3.69 (s, 3H), 1.48 (br, 9H); ^{13}C NMR (151 MHz, $CDCl_3$): δ = 189.4 (d, *J* = 11.6 Hz), 167.9, 167.0 (d, *J* = 258.7 Hz), 152.4, 144.5, 140.8, 138.1, 136.2 (d, *J* = 10.4 Hz), 121.4, 121.3 (d, *J* = 3.2 Hz), 116.6, 115.3 (d, *J* = 21.6 Hz), 107.5, 83.3, 51.5, 46.8, 28.2; ^{19}F NMR (585 MHz, $CDCl_3$): δ = –120.1; IR (ATR): ṽ = 2980, 1712, 1694, 1624, 1474, 1384, 1328, 1238, 1134, 995 cm^{-1}; HRMS (ESI$^+$): *m/z*: calcd. for $C_{19}H_{22}NO_5FNa$ [M+Na]$^+$: 386.13742, found: 386.13770.

Methyl (2*E*,4*E*)-5-((*tert*-butoxycarbonyl)(2-formyl-5-methoxybenzyl)amino)penta-2,4-dienoate (7g).

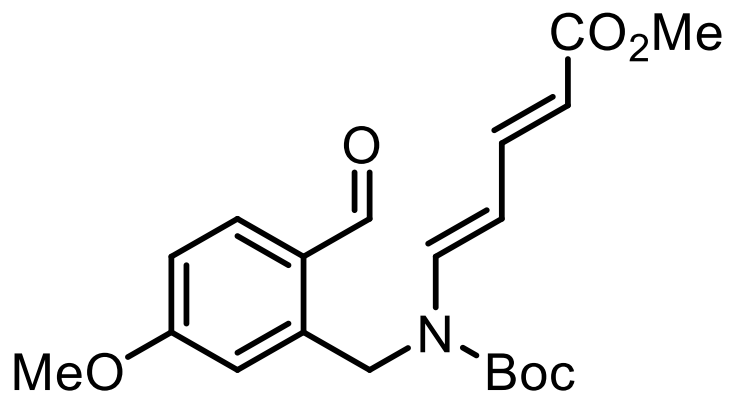

The title compound was prepared according to the representative procedure **B** using methyl (2*E*,4*E*)-5-((*tert*-butoxycarbonyl)amino)-penta-2,4-dienoate **1a** (182 mg, 0.80 mmol) and 2-(bromomethyl)-4-methoxybenzaldehyde **S11** (367 mg, 1.60 mmol). The crude material was purified by flash chromatography (SiO_2, *n*-hexane/ethyl acetate, 4:1) to give the product as a yellow oil which slowly solidified upon standing (220 mg, 73%). ^{1}H NMR (600 MHz, $CDCl_3$): δ = 10.00 (d, *J* = 0.7 Hz, 1H), 7.80 (d, *J* = 8.5 Hz, 1H), 7.60 (br, 1H), 7.35 – 7.27 (m, 1H), 6.92 (dd, *J* = 8.5, 2.5 Hz, 1H), 6.58 (s, 1H), 5.59 (d, *J* = 15.2 Hz, 1H), 5.37 (t, *J* = 12.6 Hz, 1H), 5.24 (s, 2H), 3.83 (s, 3H), 3.69 (s, 3H), 1.49 (s, 9H); ^{13}C NMR (151 MHz, $CDCl_3$): δ = 192.3, 168.0, 164.5, 152.5, 144.7, 141.1, 139.0, 138.1, 126.7, 116.3, 112.5, 111.4, 107.5, 83.2, 55.7, 51.4, 46.3, 28.2; IR (ATR): ṽ = 2978, 1711, 1688, 1623, 1568, 1384, 1288, 1240, 1135, 969 cm^{-1}; HRMS (ESI$^+$): *m/z*: calcd. for $C_{20}H_{25}NO_6Na$ [M+Na]$^+$: 398.15741, found: 398.15744.

Methyl (2*E*,4*E*)-5-((*tert*-butoxycarbonyl)((4-formyl-[1,1'-biphenyl]-3-yl)methyl)amino)penta-2,4-dienoate (7h).

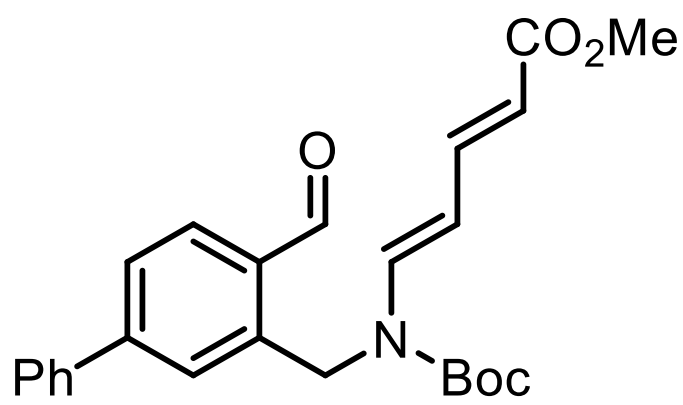

The title compound was prepared according to the representative procedure **C** using methyl (2*E*,4*E*)-5-((*tert*-butoxycarbonyl)amino)-penta-2,4-dienoate **1a** (136 mg, 0.60 mmol) and 3-(bromomethyl)-[1,1'-biphenyl]-4-carbaldehyde **S12** (413 mg, 1.50 mmol). The crude material was purified by flash chromatography (SiO_2, *n*-hexane/ethyl acetate, 5:1) to give the product as a pale brown solid (185 mg, 73%). ^{1}H NMR (600 MHz, $CDCl_3$): δ = 10.19 (d, *J* = 0.6 Hz, 1H), 7.93 (d, *J* = 7.9 Hz, 1H), 7.70 (d, *J* = 7.8 Hz, 1H), 7.63 (br, 1H), 7.54 – 7.51 (m, 2H), 7.48 – 7.44 (m, 2H), 7.43 – 7.39 (m, 1H), 7.36 – 7.27 (m, 2H), 5.60 (d, *J* = 15.2 Hz, 1H), 5.48 – 5.41 (m, 1H), 5.32 (s, 2H), 3.68 (s, 3H), 1.48 (s, 9H); ^{13}C NMR (151 MHz, $CDCl_3$): δ = 193.4, 168.0, 152.6, 147.0, 144.6, 139.6, 139.0, 138.2, 136.6, 132.0, 129.2, 128.8, 127.5, 126.2, 124.5, 116.5, 107.6, 83.2, 51.4, 46.5, 28.2; IR (ATR): ṽ = 2966, 1711, 1692, 1624, 1384, 1260, 1241, 1135, 1097, 1020, 799 cm^{-1}; HRMS (ESI$^+$): *m/z*: calcd. for $C_{25}H_{27}NO_5Na$ [M+Na]$^+$: 444.17814, found: 444.17800.

Methyl (2*E*,4*E*)-5-((5-bromo-2-formylbenzyl)(*tert*-butoxycarbonyl)amino)penta-2,4-dienoate (7i).

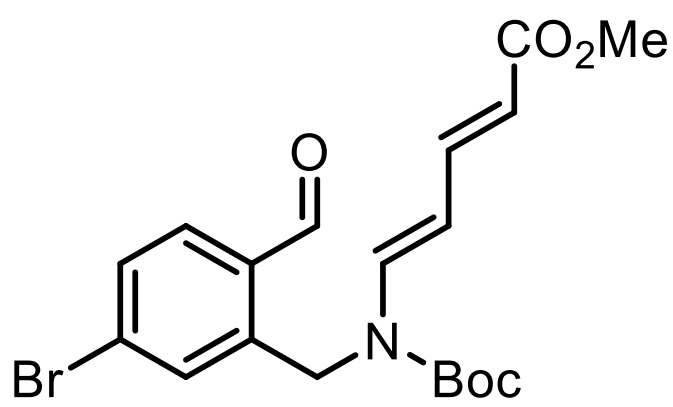

The title compound was synthesized according to the representative procedure **B** using methyl (2*E*,4*E*)-5-((*tert*-butoxycarbonyl)amino)-penta-2,4-dienoate **1a** (136 mg, 0.60 mmol) and 4-bromo-2-(bromomethyl)-benzaldehyde **S13** (334 mg, 1.20 mmol). The crude material was purified by flash chromatography (SiO_2, *n*-hexane/ethyl acetate, 5:1) to give the product as a brown solid (190 mg, 75%). ^{1}H NMR (400 MHz, $CDCl_3$): δ = 10.10 (s, 1H), 7.71 (d, *J* = 8.1 Hz, 1H), 7.64 (dd, *J* = 8.1, 1.8 Hz, 1H), 7.60 (br, 1H), 7.34 – 7.26 (m, 1H), 7.20 (s, 1H), 5.61 (d, *J* = 15.2 Hz, 1H), 5.36 (t, *J* = 12.6 Hz, 1H), 5.22 (s, 2H), 3.69 (s, 3H), 1.49 (s, 9H); ^{13}C NMR (101 MHz, $CDCl_3$): δ = 192.7, 167.8, 152.3, 144.3, 140.5, 137.8, 137.0, 131.9, 131.0, 130.1, 129.2, 116.8, 107.5, 83.5, 51.4, 45.9, 28.2; IR (ATR): ṽ = 2979, 1707, 1624, 1590, 1384, 1241, 1135, 970 cm^{-1}; HRMS (ESI$^+$): *m/z*: calcd. for $C_{19}H_{22}NO_5BrNa$ [M+Na]$^+$: 446.05736, found: 446.05727.

Methyl (2*E*,4*E*)-5-((*tert*-butoxycarbonyl)(2-formyl-5-(phenylsulfonyl)benzyl)amino)penta-2,4-dienoate (7j).

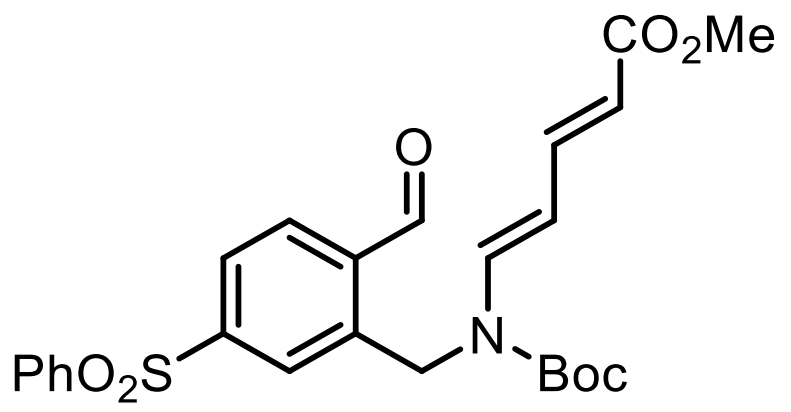

The title compound was prepared according to the representative procedure **B** using methyl (2*E*,4*E*)-5-((*tert*-butoxycarbonyl)amino)-penta-2,4-dienoate **1a** (136 mg, 0.60 mmol) and 2-(bromomethyl)-4-(phenylsulfonyl)benzaldehyde **S14** (407 mg, 1.20 mmol). The crude material was purified by flash chromatography (SiO_2, *n*-hexane/ethyl acetate, 2:1) to give the product as a yellow solid (82.5 mg, 28%). ^1H NMR (400 MHz, $CDCl_3$): δ = 10.21 (s, 1H), 8.07 (d, *J* = 8.0 Hz, 1H), 8.01 (d, *J* = 7.9 Hz, 1H), 7.88 – 7.81 (m, 2H), 7.64 – 7.52 (m, 3H), 7.51 – 7.40 (m, 2H), 7.32 – 7.24 (m, 1H), 5.49 (d, *J* = 15.2 Hz, 1H), 5.31 – 5.08 (m, 3H), 3.73 (s, 3H), 1.51 (s, 9H); ^{13}C NMR (101 MHz, $CDCl_3$): δ = 192.5, 167.8, 152.2, 146.6, 144.1, 140.5, 140.2, 137.7, 136.1, 136.0, 133.9, 129.7, 128.0, 126.7, 125.4, 116.9, 107.6, 83.8, 51.5, 46.2, 28.2; IR (ATR): ṽ = 2977, 1707, 1625, 1385, 1326, 1243, 1138, 728 cm^{-1}; HRMS (ESI$^+$): *m/z*: calcd. for $C_{25}H_{27}NO_7SNa$ $[M+Na]^+$: 508.14004, found: 508.14029.

Methyl (2*E*,4*E*)-5-((*tert*-butoxycarbonyl)(2-formyl-5-(4,4,5,5-tetramethyl-1,3,2-dioxaborolan-2-yl)benzyl)amino)penta-2,4-dienoate (7k).

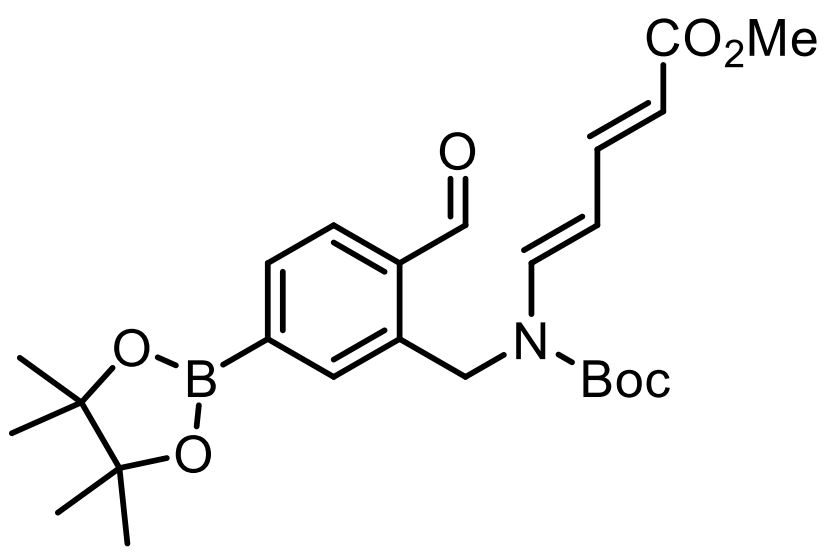

The title compound was prepared according to the representative procedure **C** using methyl (2*E*,4*E*)-5-((*tert*-butoxycarbonyl)-amino)penta-2,4-dienoate **1a** (136 mg, 0.60 mmol) and 2-(bromomethyl)-4-(4,4,5,5-tetramethyl-1,3,2-dioxaborolan-2-yl)-benzaldehyde **S15** (488 mg, 1.50 mmol). The crude material was purified by flash chromatography (SiO_2, *n*-hexane/ethyl acetate, 4:1) and subsequently recrystallized from pentane/*tert*-butyl methyl ether. ***Note:*** *The silica used for flash chromatography was treated with boric acid prior to use following a literature procedure*.[7] The product was isolated as an off-white solid (179 mg, 63%). ^1H NMR (600 MHz, $CDCl_3$): δ = 10.18 (d, *J* = 0.7 Hz, 1H), 7.90 (d, *J* = 7.5 Hz, 1H), 7.84 (d, *J* = 7.5 Hz, 1H), 7.64 (br, 1H), 7.49 (br, 1H), 7.31 (dd, *J* = 15.2, 11.2 Hz, 1H), 5.58 (d, *J* = 15.2 Hz, 1H), 5.39 (t, *J* = 12.5 Hz, 1H), 5.24 (s, 2H), 3.68 (s, 3H), 1.48 (br, 9H), 1.31 (s, 12H); ^{13}C NMR (151 MHz, $CDCl_3$): δ = 194.3, 168.1, 152.8, 145.0, 138.5, 137.1, 135.7, 134.9, 134.8, 133.8, 131.9, 116.1, 107.3, 84.5, 83.1, 51.4, 46.2, 28.1, 25.0; IR (ATR): ṽ = 2979, 1712, 1699, 1624, 1385, 1368, 1240, 1137, 966, 854 cm^{-1}; HRMS (ESI$^+$): *m/z*: calcd. for $C_{25}H_{34}NO_7BNa$ $[M+Na]^+$: 494.23205, found: 494.23218.

Methyl (2*E*,4*E*)-5-((*tert*-butoxycarbonyl)(4-chloro-2-formylbenzyl)amino)penta-2,4-dienoate (7m).

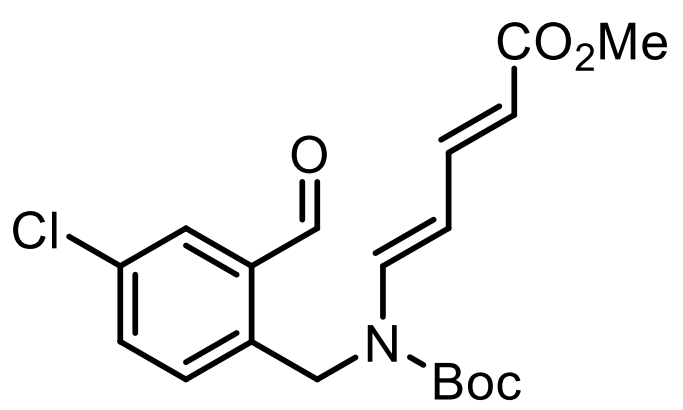

The title compound was prepared according to the representative procedure **B** using methyl (2*E*,4*E*)-5-((*tert*-butoxycarbonyl)amino)-penta-2,4-dienoate **1a** (136 mg, 0.60 mmol) and 2-(bromomethyl)-5-chloro-benzaldehyde **S16** (280 mg, 1.20 mmol). The crude material was purified by flash chromatography (SiO_2, *n*-hexane/ethyl acetate, 5:1) and subsequently recrystallized from *n*-hexane/ethyl acetate. The product was obtained as a pale yellow solid (100 mg, 44%). ^1H NMR (600 MHz, $CDCl_3$): δ = 10.11 (d, *J* = 0.7 Hz, 1H), 7.83 (d, *J* = 2.3 Hz, 1H), 7.59 (br, 1H), 7.52 (dd, *J* = 8.4, 2.3 Hz, 1H), 7.29 (dd, *J* = 15.2, 11.1 Hz, 1H), 7.05 (d, *J* = 8.3 Hz, 1H), 5.60 (d, *J* = 15.2 Hz, 1H), 5.39 – 5.30 (m, 1H), 5.20 (s, 2H), 3.69 (s, 3H), 1.48 (br, 9H); ^{13}C NMR (151 MHz, $CDCl_3$): δ = 192.3, 167.9, 152.4, 144.3, 138.0, 136.9, 135.2, 134.4, 134.2, 133.8, 127.5, 116.7, 107.5, 83.4, 51.5, 46.0, 28.2; IR (ATR): ṽ =2980, 1700, 1620, 1434, 1383, 1330, 1238, 1129, 995, 906 cm^{-1}; HRMS (ESI$^+$): *m/z*: calcd. for $C_{19}H_{22}NO_5ClNa$ $[M+Na]^+$: 402.10787, found: 402.10793.

Methyl (2*E*,4*E*)-5-((*tert*-butoxycarbonyl)(2-formyl-4-(trifluoromethoxy)benzyl)amino)penta-2,4-dienoate (7n).

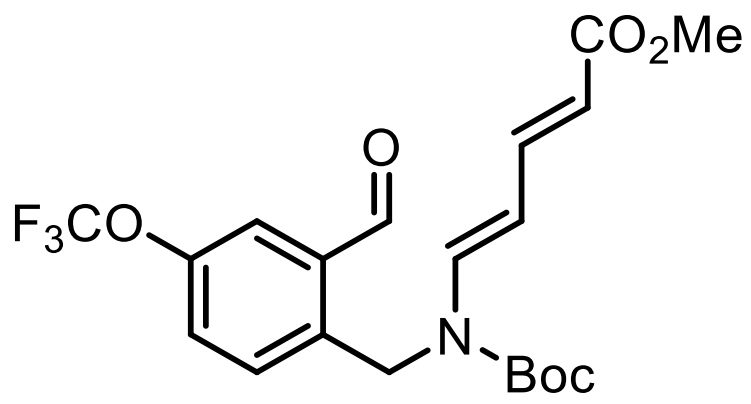

The title compound was prepared according to the representative procedure **B** using methyl (2*E*,4*E*)-5-((*tert*-butoxycarbonyl)amino)-penta-2,4-dienoate **1a** (182 mg, 0.80 mmol) and 2-(bromo-methyl)-5-(trifluoromethoxy)benzaldehyde **S17** (453 mg, 1.60 mmol). The crude material was purified by flash chromatography (SiO_2, *n*-hexane/ethyl acetate, 6:1) to give the product as an off-white solid (239 mg, 67%). ^1H NMR (400 MHz, $CDCl_3$): δ = 10.15 (d, *J* = 0.7 Hz, 1H), 7.71 (dd, *J* = 2.6, 1.1 Hz, 1H), 7.60 (br, 1H), 7.45 – 7.38 (m, 1H), 7.30 (dd, *J* = 15.2, 11.2 Hz, 1H), 7.14 (d, *J* = 8.5 Hz, 1H), 5.61 (d, *J* = 15.2 Hz, 1H), 5.36 (dd, *J* = 14.0, 11.2 Hz, 1H), 5.23 (s, 2H), 3.70 (s, 3H), 1.49 (br, 9H); ^{13}C NMR (101 MHz, $CDCl_3$): δ = 192.0, 167.9, 152.4, 148.7 (q, *J* = 2.2 Hz), 144.3, 137.9, 137.1, 134.5, 127.8, 127.2, 126.5, 120.5 (q, *J* = 258.7 Hz), 116.8, 107.5, 83.5, 51.5, 45.9, 28.2; ^{19}F NMR (282 MHz, $CDCl_3$): δ = –58.0; IR (ATR): ṽ = 2982, 1709, 1626, 1386, 1246, 1136, 995 cm^{-1}; HRMS (ESI$^+$): *m/z*: calcd. for $C_{20}H_{22}NO_6F_3Na$ $[M+Na]^+$: 452.12914, found: 452.12900.

Methyl (2*E*,4*E*)-5-((*tert*-butoxycarbonyl)((4-formylthiophen-3-yl)methyl)amino)penta-2,4-dienoate (S28).

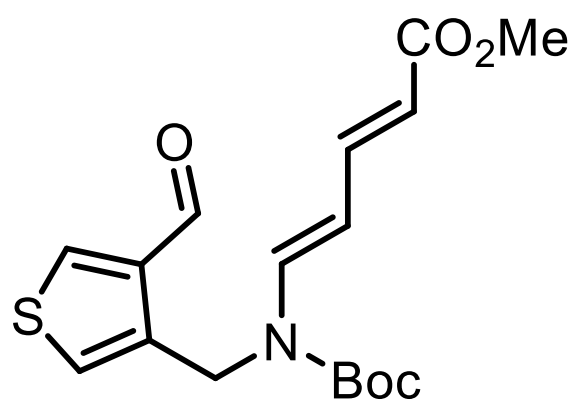

The title compound was prepared according to the representative procedure **B** using methyl (2*E*,4*E*)-5-((*tert*-butoxycarbonyl)amino)penta-2,4-dienoate **1a** (182 mg, 0.80 mmol) and 3-(bromomethyl)thiophene-2-carbaldehyde **S18** (327 mg, 1.60 mmol). The crude material was purified by flash chromatography (SiO_2, *n*-hexane/ethyl acetate, 4:1) to give the product as an off-white solid (212 mg, 76%). ^1H NMR (400 MHz, $CDCl_3$): δ = 10.00 (d, *J* = 1.0 Hz, 1H), 8.15 (d, *J* = 3.2 Hz, 1H), 7.55 (br, 1H), 7.30 (dd, *J* = 15.2, 11.2 Hz, 1H), 6.90 (dd, *J* = 3.1, 1.2 Hz, 1H), 5.63 (d, *J* = 15.2 Hz, 1H), 5.49 (dd, *J* = 14.0, 11.2 Hz, 1H), 5.07 (s, 2H), 3.70 (s, 3H), 1.49 (br, 9H); ^{13}C NMR (101 MHz, $CDCl_3$): δ = 186.0, 168.0, 152.3, 144.7, 141.4, 139.7, 137.9, 137.2, 123.1, 116.4, 107.4, 83.2, 51.4, 44.3, 28.2; IR (ATR): ṽ = 2979, 1709, 1682, 1620, 1434, 1383, 1327, 1239, 1131, 995, 966 cm^{-1}; HRMS (ESI$^+$): *m/z*: calcd. for $C_{17}H_{21}NO_5SNa$ $[M+Na]^+$: 374.10326, found: 374.10352.

Methyl (2*E*,4*E*)-5-((*tert*-butoxycarbonyl)((2-formylfuran-3-yl)methyl)amino)penta-2,4-dienoate (S29)

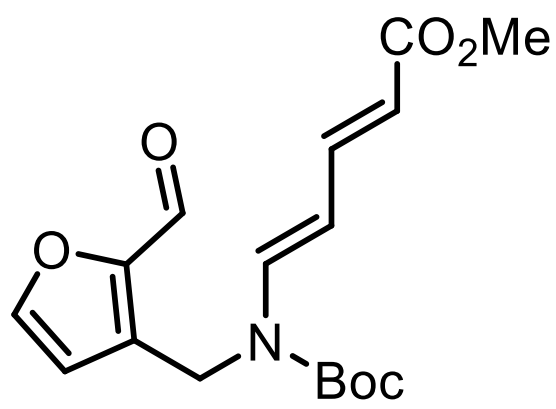

The title compound was prepared according to the representative procedure **B** using methyl (2*E*,4*E*)-5-((*tert*-butoxycarbonyl)amino)penta-2,4-dienoate **1a** (102 mg, 0.45 mmol) and a stock solution of 3-(bromomethyl)furan-2-carbaldehyde **S20** (170 mg, 0.90 mmol) in C_6D_6. The crude material was purified by flash chromatography (SiO_2, pentane/ethyl acetate, 5:1) to give the product as a pale yellow oil (93.5 mg, 62%). ^1H NMR (400 MHz, $CDCl_3$): δ = 9.87 (d, *J* = 0.7 Hz, 1H), 7.56 (d, *J* = 1.7 Hz, 1H), 7.43 (br, 1H), 7.33 – 7.22 (m, 1H), 6.43 (d, *J* = 1.7 Hz, 1H), 5.68 (d, *J* = 15.2 Hz, 1H), 5.60 (dd, *J* = 13.9, 11.0 Hz, 1H), 5.01 (s, 2H), 3.70 (s, 3H), 1.52 (s, 9H); ^{13}C NMR (101 MHz, $CDCl_3$): δ = 180.4, 168.0, 152.3, 148.4, 147.7, 144.4, 137.5, 131.8, 116.8, 113.2, 107.5, 83.5, 51.5, 39.2, 28.2; IR (ATR): ṽ = 2979, 1709, 1677, 1621, 1475, 1385, 1330, 1240, 1131, 996, 787 cm^{-1}; HRMS (ESI$^+$): *m/z*: calcd. for $C_{17}H_{21}NO_6Na$ $[M+Na]^+$: 358.12611, found: 358.12597.

Methyl (2*E*,4*E*)-5-((*tert*-butoxycarbonyl)(4-oxobutyl)amino)penta-2,4-dienoate (S30)

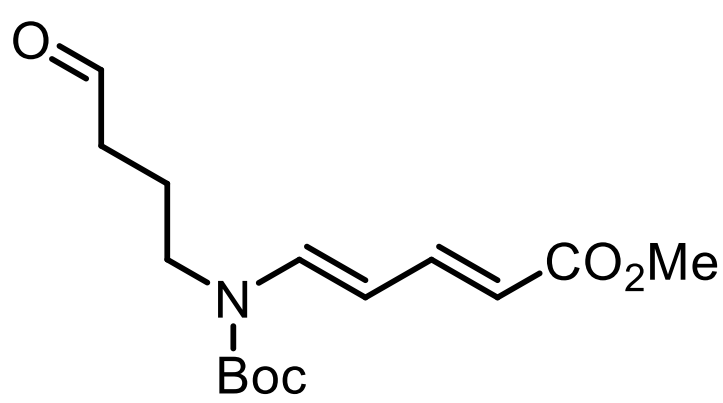

A solution of methyl (2*E*,4*E*)-5-((*tert*-butoxycarbonyl)(4-hydroxybutyl)amino)penta-2,4-dienoate **S24** (1.23 g, 0.97 mmol) in dichloromethane (5.0 mL) and water (19.2 µL, 1.07 mmol) was added to a suspension of Dess-Martin periodinane (357 mg, 2.90 mmol) and $NaHCO_3$ (488 mg, 5.81 mmol) in dichloromethane (5.0 mL) at 0 °C (bath temperature). The mixture was stirred at room temperature for

3 h. Dichloromethane (5 mL), sat. aq. $NaHCO_3$ (5 mL) and sat. aq. $Na_2S_2O_3$ (5 mL) were added and the mixture was vigorously stirred for 20 min. The organic phase was separated and the aqueous layer extracted with dichloromethane (3 x 10 mL). The combined organic phases were washed with sat. aq. $Na_2S_2O_3$ (10 mL) and brine (10 mL), dried over $MgSO_4$ and filtered, and the solvent was removed under reduced pressure. The residue was purified by flash chromatography (SiO_2, *n*-pentane/ethyl acetate, 5:1 → 3:1) to give the title compound as a pale yellow oil (99 mg, 34%). ^{1}H NMR (400 MHz, $CDCl_3$): δ = 9.79 (s, 1H), 7.38 (br, 1H), 7.34– 7.29 (m, 1H), 5.77 (d, *J* = 15.1 Hz, 1H), 5.70 (dd, *J* = 14.1, 11.2 Hz, 1H), 3.73 (s, 3H), 3.58 (t, *J* = 7.4 Hz, 2H), 2.51 (td, *J* = 7.0, 1.0 Hz, 2H), 1.96 – 1.79 (m, 2H), 1.51 (s, 9H); ^{13}C NMR (101 MHz, $CDCl_3$): δ = 201.2, 168.1, 152.3, 145.1, 138.1, 115.9, 106.5, 82.9, 51.5, 43.0, 40.9, 28.3, 19.6; IR (ATR): ṽ = 2951, 1711, 1621, 1390, 1312, 1241, 1134, 998 cm^{-1} HRMS (ESI$^+$): *m/z*: calcd. for $C_{15}H_{23}NO_5Na$ [M+Na]$^+$: 320.14684, found: 320.14712.

Methyl (2*E*,4*E*)-5-((*tert*-butoxycarbonyl)(3-oxopropyl)amino)penta-2,4-dienoate (S31)

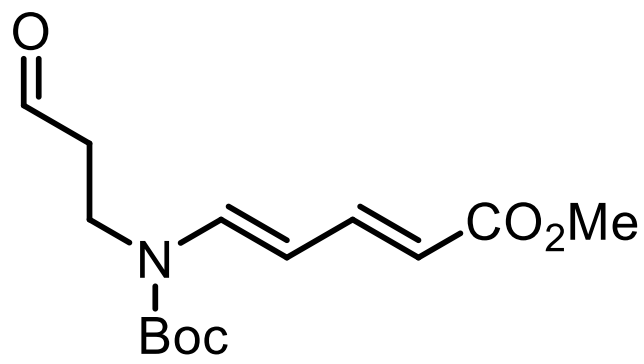

A solution of methyl (2*E*,4*E*)-5-((*tert*-butoxycarbonyl)(3-hydroxypropyl)-amino)penta-2,4-dienoate **S26** (120 mg, 0.42 mmol) in dichloromethane (2.0 mL) was added to a suspension of Dess-Martin periodinane (357 mg, 0.84 mmol) and $NaHCO_3$ (177 mg, 2.10 mmol) in dichloromethane (3.0 mL) at 0 °C (bath temperature). The mixture was stirred at room temperature for 1 h before dichloromethane (5 mL), sat. aq. $NaHCO_3$ (5 mL) and sat. aq. $Na_2S_2O_3$ (5 mL) were added. The resulting mixture was vigorously stirred for 20 min, the organic phase was separated and the aqueous layer was extracted with dichloromethane (3 x 10 mL). The combined organic phases were washed with sat. aq. $Na_2S_2O_3$ (10 mL) and brine (10 mL), dried over $MgSO_4$ and filtered, and the solvent was removed under reduced pressure. The residue was purified by flash chromatography (SiO_2, *n*-pentane/ethyl acetate, 4:1) to give the title compound as a colorless oil (63 mg, 53%). ^{1}H NMR (400 MHz, $CDCl_3$): δ = 9.81 (s, 1H), 7.41 – 7.37 (m, 1H), 7.31 (ddd, *J* = 15.2, 11.1, 0.7 Hz, 1H), 5.75 (dd, *J* = 15.2, 0.6 Hz, 1H), 5.67 – 5.49 (m, 1H), 3.89 (t, *J* = 7.2 Hz, 2H), 3.73 (s, 3H), 2.73 (td, *J* = 7.2, 1.4 Hz, 2H), 1.51 (s, 9H); ^{13}C NMR (101 MHz, $CDCl_3$): δ = 199.8, 168.0, 152.0, 144.7, 137.7, 116.4, 106.3, 83.3, 51.5, 41.7, 37.7, 28.2; IR (ATR): ṽ = 2980, 1710, 1622, 1387, 1334, 1241, 1134, 1059, 966 cm^{-1} HRMS (ESI$^+$): *m/z*: calcd. for $C_{14}H_{21}NO_5Na$ [M+Na]$^+$: 306.13119, found: 306.13084.

Methyl (2*E*,4*E*)-5-((*tert*-butoxycarbonyl)(2,2-dimethyl-3-oxopropyl)amino)penta-2,4-dienoate (S32)

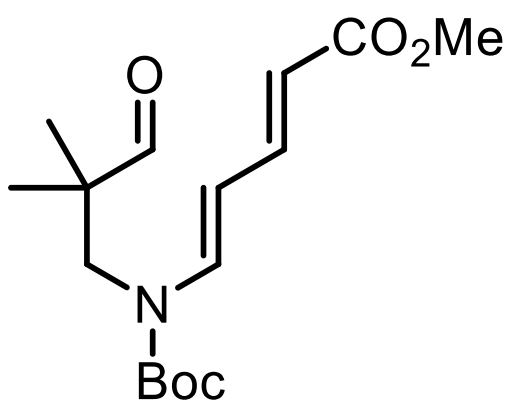

Methyl (2*E*,4*E*)-5-((*tert*-butoxycarbonyl)amino)penta-2,4-dienoate **1a** (182 mg, 0.80 mmol) was added in portions to a suspension of sodium hydride (25.0 mg, 1.04 mmol) in N,N-dimethylformamide (4.0 mL) at 0 °C (bath temperature). After complete addition, the mixture was stirred at 0 °C for another 10 min. 3-Bromo-2,2-dimethylpropanal **S21** (264 mg, 1.60 mmol) and tetrabutyl-ammonium iodide (29.5 mg, 0.08 mmol) were added. The mixture was stirred at 80 °C (bath temperature) for 16 h. After cooling to 0 °C, sat. aq. $NaHCO_3$ (5 mL) was carefully added, followed by addition of ethyl acetate (20 mL) and water (10 mL). The mixture was vigorously stirred for 5 min, the organic phase was separated, and the aqueous layer was extracted with ethyl acetate (3 x 20 mL). The combined organic phases were washed with aq. sat. LiCl (5 x 10 mL), dried over $MgSO_4$ and filtered, and the solvent was removed under reduced pressure. The residue was purified by flash chromatography (SiO_2, *n*-hexane/ethyl acetate, 4:1) to give the title compound as a pale yellow oil (192 mg, 77%). ^{1}H NMR (400 MHz, $CDCl_3$): δ = 9.50 (s, 1H), 7.42 (d, *J* = 14.3 Hz, 1H), 7.34 – 7.26 (m, 1H), 5.74 (dt, *J* = 15.2, 0.7 Hz, 1H), 5.63 (dd, *J* = 14.1, 11.0 Hz, 1H), 3.72 (s, 3H), 3.67 (s, 2H), 1.49 (s, 9H), 1.09 (s, 6H); ^{13}C NMR (101 MHz, $CDCl_3$): δ = 203.3, 168.0, 152.6, 144.7, 139.3, 116.4, 107.7, 83.7, 51.5, 49.5, 47.6, 28.1, 20.4; IR (ATR): ṽ = 2977, 1712, 1622, 1435, 1387, 1241, 1133, 997 cm^{-1} HRMS (ESI$^+$): *m/z*: calcd. for $C_{16}H_{25}NO_5Na$ [M+Na]$^+$: 334.16249, found: 334.16237.

Methyl (2*E*,4*E*)-5-((*tert*-butoxycarbonyl)((3-formyloxetan-3-yl)methyl)amino)penta-2,4-dienoate (S33)

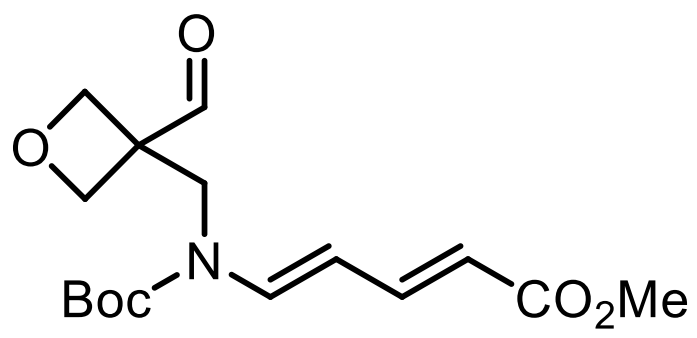

Methyl (2*E*,4*E*)-5-((*tert*-butoxycarbonyl)amino)penta-2,4-dienoate **1a** (159 mg, 0.70 mmol) was added in portions to a suspension of sodium hydride (25.2 mg, 1.05 mmol) in THF (4.0 mL) at 0 °C (bath temperature). After complete addition, the mixture was stirred at 0 °C for another 10 min. 3-(Bromomethyl)oxetane-3-carbaldehyde **S22** (251 mg, 1.40 mmol) and tetrabutylammonium iodide (25.9 mg, 0.07 mmol) were added and the mixture was stirred at 80 °C (bath temperature) for 6 h. After cooling to to 0 °C, sat. aq. $NaHCO_3$ solution (5 mL) was carefully added, followed by addition of ethyl acetate (20 mL) and water (10 mL). The mixture was vigorously stirred for 5 min, the organic phase was separated and the aqueous layer extracted with ethyl acetate (3 x 20 mL). The combined organic phases were washed with brine (20 mL), dried over $MgSO_4$ and filtered, and the solvent was removed under reduced pressure. The residue was purified by flash chromatography (SiO_2, *n*-hexane/ethyl acetate, 1.5:1 → 1:1) to give the title compound as a pale yellow oil (160 mg, 70%). ^{1}H NMR (400 MHz, $CDCl_3$): δ = 9.98 (s, 1H), 7.51 – 7.43 (m, 1H), 7.34 – 7.27 (m, 1H), 5.76 (d, *J* = 15.2 Hz, 1H), 5.58 (ddd, *J* = 14.1, 11.0, 0.6 Hz, 1H), 4.73 (d, *J* = 6.8 Hz, 2H), 4.68 (d, *J* = 6.8 Hz, 2H), 3.99 (s, 2H), 3.73 (s, 3H), 1.52 (s, 9H); ^{13}C NMR (101 MHz, $CDCl_3$): δ = 198.7, 167.8, 152.5, 144.0, 138.7, 117.2, 107.1, 84.1, 73.6, 54.0, 51.5, 43.8, 28.2; IR (ATR): ṽ = 2977, 1706, 1619, 1435, 1385, 1332, 1240, 1127, 992 cm^{-1} HRMS (ESI$^+$): *m/z*: calcd. for $C_{16}H_{23}NO_6Na$ [M+Na]$^+$: 348.14176, found: 348.14165.

***tert*-Butyl (*E*)-buta-1,3-dien-1-yl(2-formylbenzyl)carbamate (S34)**

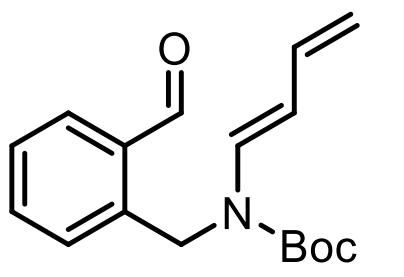

tert-Butyl (*E*)-buta-1,3-dien-1-ylcarbamate (300 mg, 1.77 mmol) was added in portions to a suspension of sodium hydride (72.0 mg, 3.00 mmol) in THF (18 mL) at 0 °C (bath temperature). After complete addition, the mixture was stirred at 0 °C for 10 min before 2-(bromomethyl)benzaldehyde **S6** was introduced, The mixture was stirred at 50 °C (bath temperature) for 16 h. After cooling to 0 °C, sat. aq. $NaHCO_3$ solution (5 mL) was carefully added, followed by ethyl acetate (20 mL) and water (10 mL). The mixture was vigorously stirred for 5 min, the organic phase was separated and the aqueous layer was extracted with ethyl acetate (3 x 20 mL). The combined organic phases were washed with brine (20 mL), dried over $MgSO_4$ and filtered, and the solvent was removed under reduced pressure.

In order to remove remaining 2-(bromomethyl)benzaldehyde, the crude product was dissolved in dichloromethane, pyridine (0.16 mL, 1.95 mmol) was added, and the resulting mixture was stirred at room temperature for 24 h. The mixture was washed with aq. $CuSO_4$ solution (10% *w/w*, 2 x 10 mL), water (2 x 10 mL) and brine (20 mL) before it was dried over $MgSO_4$ and filtered. The solvent was removed under reduced pressure and the residue purified by flash chromatography (SiO_2, toluene/*tert*-butyl methyl ether, 150:1) to give the title compound as a pale yellow oil (326 mg, 64%). ^{1}H NMR (400 MHz, CD_2Cl_2): δ = 10.18 (s, 1H), 7.88 (dd, *J* = 7.5, 1.5 Hz, 1H), 7.57 (td, *J* = 7.6, 1.5 Hz, 1H), 7.49 (td, *J* = 7.5, 1.2 Hz, 1H), 7.39 – 7.20 (m, 1H), 7.16 (d, *J* = 7.7 Hz, 1H), 6.35 – 6.25 (m, 1H), 5.33 (br, 1H), 5.19 (s, 2H), 4.88 – 4.84 (m, 1H), 4.82 – 4.79 (m, 1H), 1.51 (br, 9H); ^{13}C NMR (101 MHz, CD_2Cl_2): δ = 193.9, 153.2, 139.7, 135.8, 135.6, 134.3, 133.6, 131.7, 127.6, 126.2, 112.9, 111.0, 82.2, 46.4, 28.2; IR (ATR): ṽ = 2978, 1706, 1643, 1426, 1373, 1323, 1254, 1154 cm^{-1} HRMS (ESI$^+$): *m/z*: calcd. for $C_{17}H_{21}NO_3Na$ [M+Na]$^+$: 310.14136, found: 310.14121.

***tert*-Butyl (2-formylbenzyl)((1*E*,3*E*)-4-phenylbuta-1,3-dien-1-yl)carbamate (S35)**

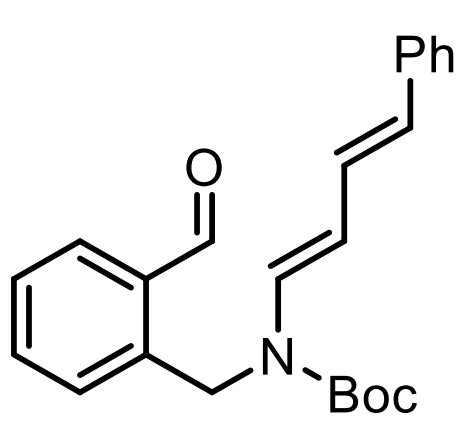

The title compound was prepared according to the representative procedure **B** using *tert*-butyl ((1*E*,3*E*)-4-phenylbuta-1,3-dien-1-yl)carbamate **S27** (245 mg, 1.00 mmol) and 2-(bromomethyl)benzaldehyde **S6** (398 mg, 2.00 mmol). The crude material was purified by flash chromatography (SiO_2, *n*-hexane/ethyl acetate, 10:1 → 4:1) to give the product as a pale yellow solid (288 mg, 79%). ^{1}H

NMR (600 MHz, [D$_6$]-DMSO, 383 K): δ = 10.29 (s, 1H), 7.97 (dd, *J* = 7.6, 1.5 Hz, 1H), 7.64 (td, *J* = 7.6, 1.5 Hz, 1H), 7.56 – 7.49 (m, 1H), 7.38 – 7.31 (m, 3H), 7.30 – 7.22 (m, 2H), 7.19 – 7.11 (m, 2H), 6.90 – 6.75 (m, 1H), 6.31 (d, *J* = 15.6 Hz, 1H), 5.68 (ddd, *J* = 14.2, 10.3, 0.7 Hz, 1H), 5.24 (s, 2H), 1.45 (s, 9H); ^{13}C NMR (151 MHz, [D$_6$]-DMSO, 383 K): δ = 192.9, 151.8, 138.4, 137.2, 133.2, 132.9, 132.8, 130.9, 127.8, 127.6, 127.4, 126.7, 125.9, 125.2, 125.0, 110.4, 81.1, 44.7, 27.3; IR (ATR): ṽ = 2977, 1695, 1635, 1390, 1333, 1238, 1147, 973, 749 cm^{-1}; HRMS (ESI$^+$): *m/z*: calcd. for $C_{23}H_{25}NO_3Na$ [M+Na]$^+$: 386.17266, found: 386.17281.

Methyl (2*E*,4*E*)-6-((*tert*-butoxycarbonyl)(2-formylphenyl)amino)hexa-2,4-dienoate (S36).

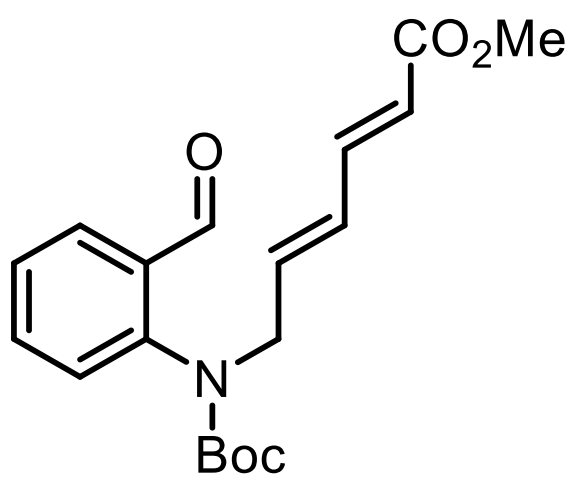

Caesium carbonate (135 mg, 0.41 mmol) was added to a solution of 2-(Boc-amino)-benzaldehyde (45.8 mg, 0.21 mmol) in N,N-dimethylformamide (2.0 mL) and the resulting mixture was stirred for 5 min. Tetrabutylammonium iodide (115 mg, 0.31 mmol) and methyl (2*E*,4*E*)-6-bromohexa-2,4-dienoate (63.7 mg, 0.31 mmol) were introduced and the mixture was stirred at 50 °C (bath temperature) for 30 min. After cooling to room temperature, water (10 mL) and ethyl acetate (10 mL) were added, the organic phase was separated and the aqueous layer extracted with ethyl acetate (2 x 10 mL). The combined organic phases were washed with aq. sat. LiCl (3 x 10 mL), dried over $MgSO_4$ and filtered, and the solvent was removed under reduced pressure. The residue was purified by flash chromatography (SiO_2, *n*-pentane/ethyl acetate, 5:1) to give the title compound as a yellow oil (43.8 mg, 61%). ^{1}H NMR (400 MHz, CD_2Cl_2): δ = 10.05 (s, 1H), 7.85 (dd, *J* = 7.7, 1.7 Hz, 1H), 7.61 (td, *J* = 7.7, 1.7 Hz, 1H), 7.42 (tt, *J* = 7.6, 0.9 Hz, 1H), 7.30 – 7.15 (m, 2H), 6.30 – 6.11 (m, 2H), 5.83 (d, *J* = 15.4 Hz, 1H), 4.35 (s, 2H), 3.70 (s, 3H), 1.32 (s, 9H); ^{13}C NMR (101 MHz, CD_2Cl_2): δ = 190.2, 167.3, 154.6, 144.5, 143.7, 137.6, 135.0, 133.1, 131.1, 129.4, 128.5, 127.8, 121.9, 81.7, 52.6, 51.8, 28.2; IR (ATR): ṽ = 2978, 1695, 1598, 1457, 1390, 1367, 1269, 1158, 1001 cm^{-1} HRMS (ESI$^+$): *m/z*: calcd. for $C_{19}H_{23}NO_5Na$ [M+Na]$^+$: 368.14684, found: 368.14655.

Methyl (2*E*,4*E*)-6-(2-formylphenoxy)hexa-2,4-dienoate (S37).

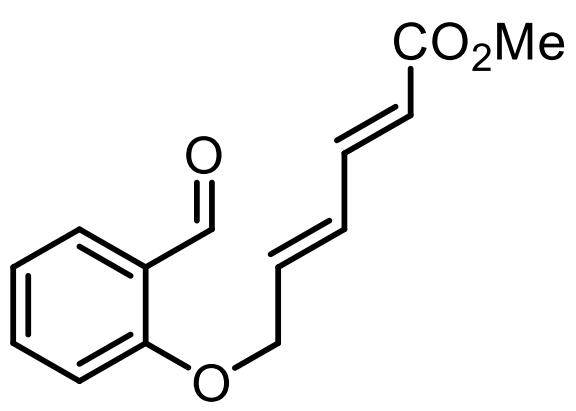

Potassium carbonate (415 mg, 3.00 mmol) and salicylaldehyde (272 µL, 2.60 mmol) were added to a solution of methyl (2*E*,4*E*)-6-bromohexa-2,4-dienoate (410 mg, 2.00 mmol) in acetonitrile (10 mL) and the resulting mixture was stirred at 65 °C (bath temperature) for 2 h. The solvent was removed under reduced pressure and the residue was dissolved in ethyl acetate (20 mL) and water (20 mL). The organic phase was separated and the aqueous layer was extracted with ethyl acetate (3 x 20 mL). The combined organic phases were washed with brine (20 mL), dried over $MgSO_4$ and filtered, and the solvent was removed under reduced pressure. The residue was purified by flash chromatography (SiO_2, *n*-hexane/ethyl acetate, 5:1) to give the title compound as an off-white solid (323 mg, 66%). ^{1}H NMR (400 MHz, $CDCl_3$): δ = 10.54 (d, *J* = 0.8 Hz, 1H), 7.86 (dd, *J* = 7.7, 1.8 Hz, 1H), 7.54 (ddd, *J* = 8.5, 7.3, 1.9 Hz, 1H), 7.33 (ddd, *J* = 15.4, 11.0, 0.7 Hz, 1H), 7.06 (tt, *J* = 7.5, 0.9 Hz, 1H), 6.96 (dd, *J* = 8.5, 0.9 Hz, 1H), 6.54 (ddtd, *J* = 15.2, 11.1, 1.7, 0.7 Hz, 1H), 6.30 (dtt, *J* = 15.5, 5.1, 0.8 Hz, 1H), 5.95 (dt, *J* = 15.5, 0.8 Hz, 1H), 4.78 (dd, *J* = 5.1, 1.7 Hz, 2H), 3.76 (s, 3H). The data matched the literature.[8]

Ni-Catalyzed Reductive Coupling Reactions

Reaction Optimization

The Ni-catalyzed reductive coupling reaction was optimized using compound **7a** as the model substrate. Reactions were run with different achiral and chiral ligands and in different solvents. Enantiomeric excesses (ee) could reliably be determined from the crude mixtures (***Note***: *in cases where the product was isolated, no difference was observed between the ee determined from the crude material or the isolated product*). Diastereomeric ratios and conversions were determined by ^{1}H NMR analysis of the crude product after aqueous workup. The product was isolated as a single diastereomer.

The results are compiled in Table 1 of the main text.

Rotamer Formation

While the products of the nickel-catalyzed reductive coupling reactions could typically be isolated in high purity, many signals in the ^{1}H, ^{13}C and ^{19}F NMR spectra recorded at room temperature were significantly broadened or even doubled. In the ^{1}H NMR spectra, many characteristic signals and ^{n}J-couplings could not be resolved and the obtained spectra were rather complex (see below). Therefore, it was decided to characterize compounds affected by massive rotamer formation at elevated temperatures, which resulted in significantly simpler spectra.

^{1}H NMR (600 MHz, $CDCl_3$, 298 K ; top) of compound 8d

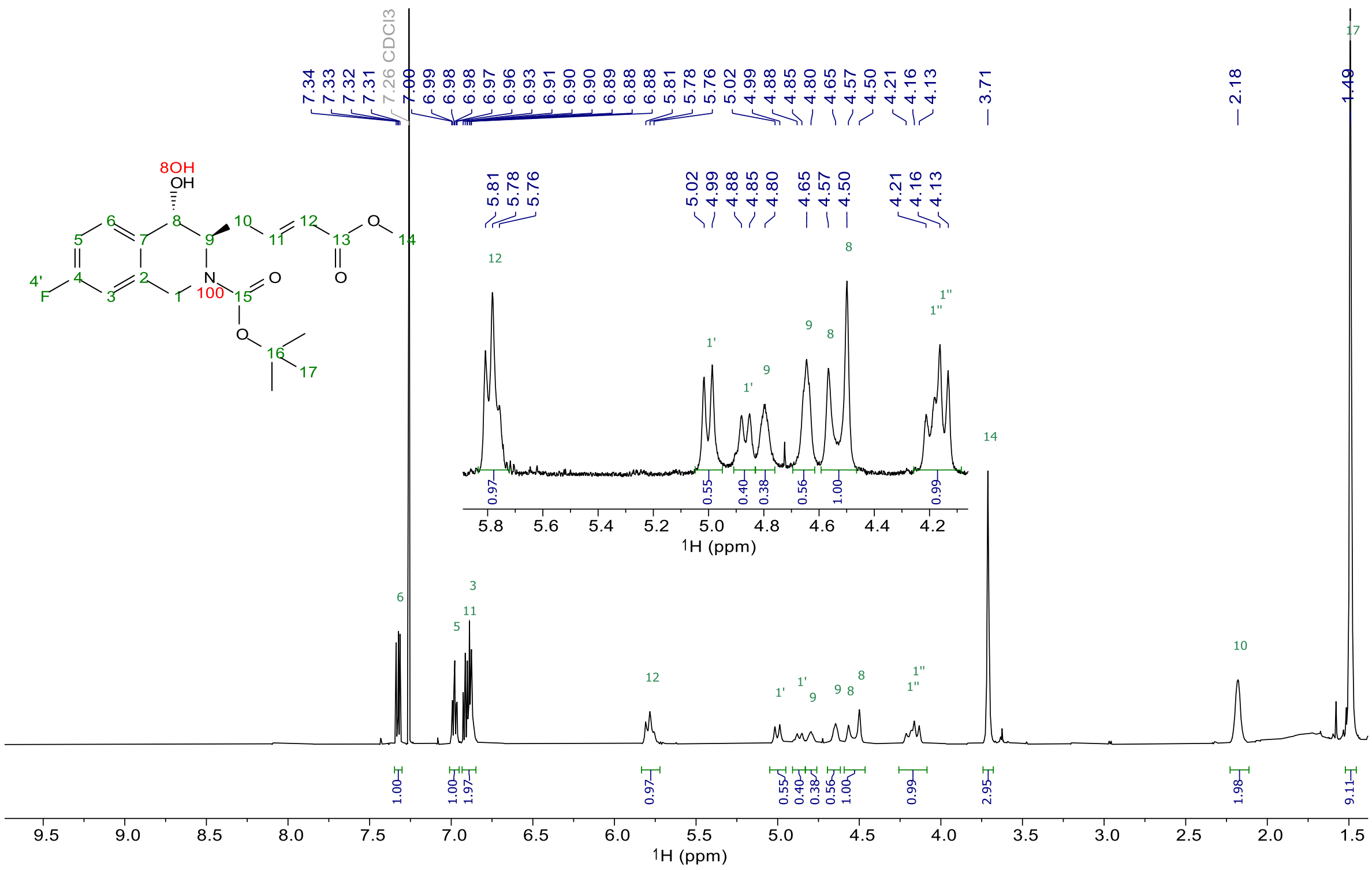

^{13}C NMR (151 MHz, $CDCl_3$, 298 K; bottom) of compound 8d

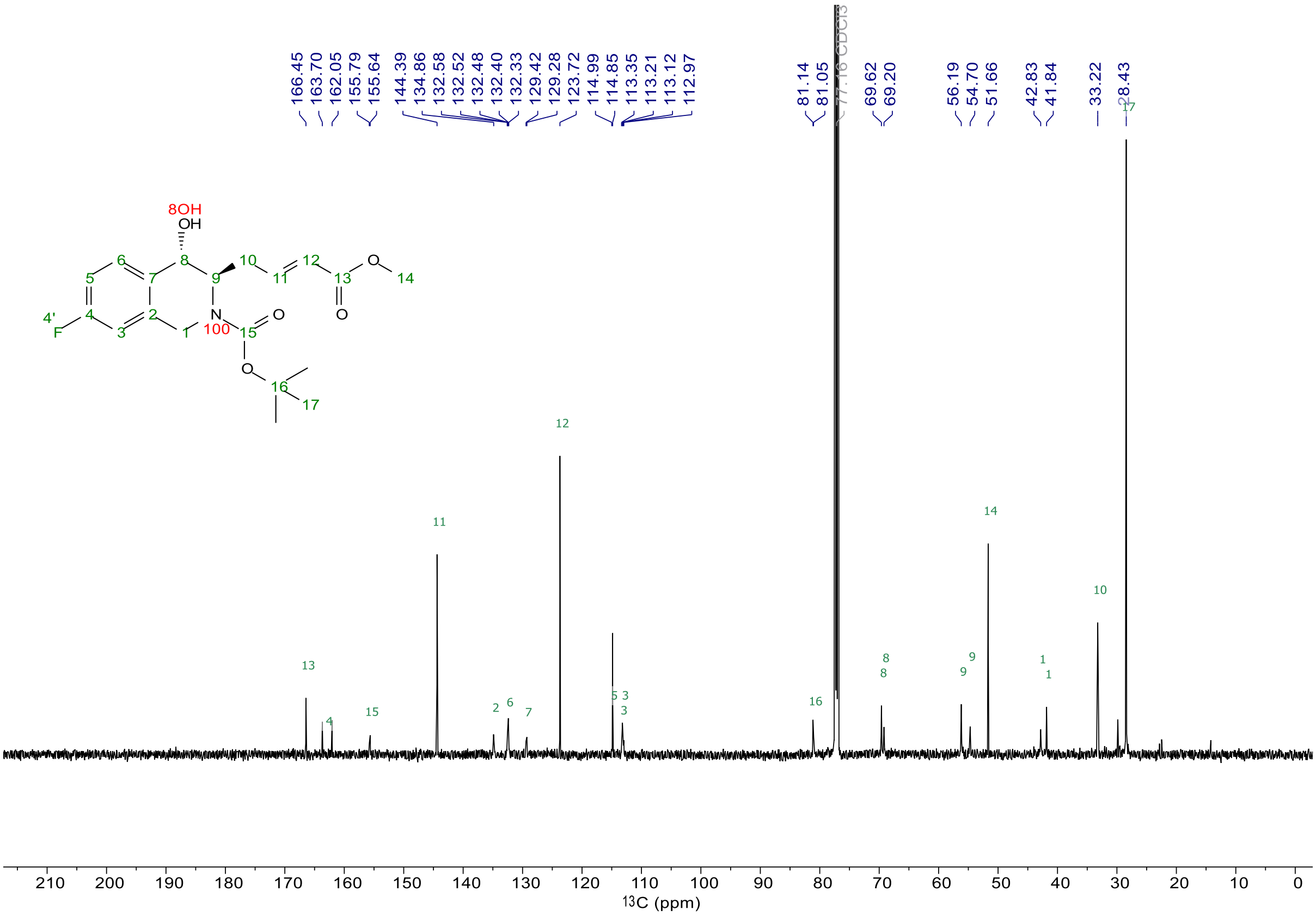

^{19}F NMR (585 MHz, $CDCl_3$, 298K) of compound 8d

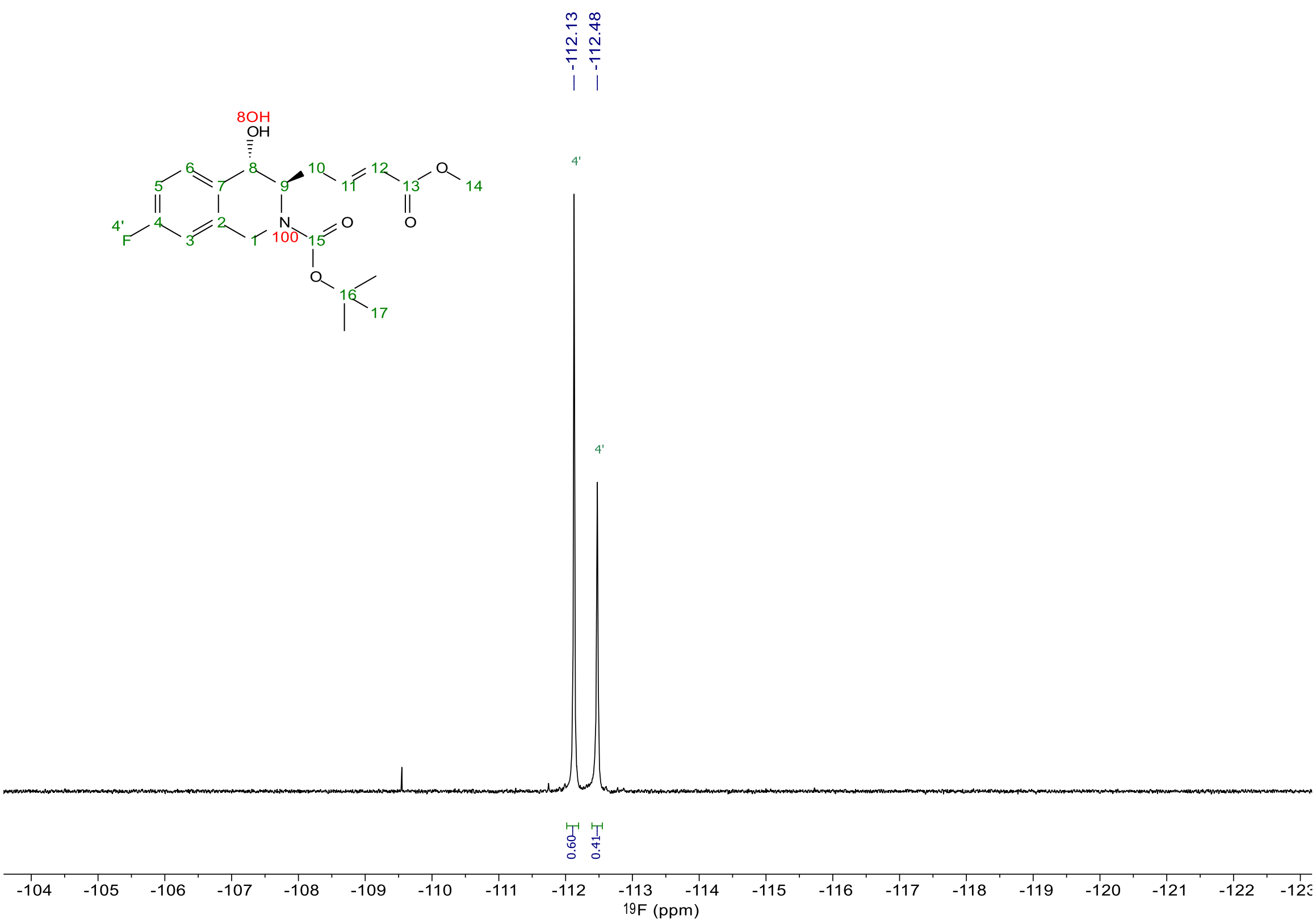

Temperature effect on rotamer formation as observed in the ^{1}H NMR spectrum of compound 8a ([D$_6$]-DMSO, 600 MHz)

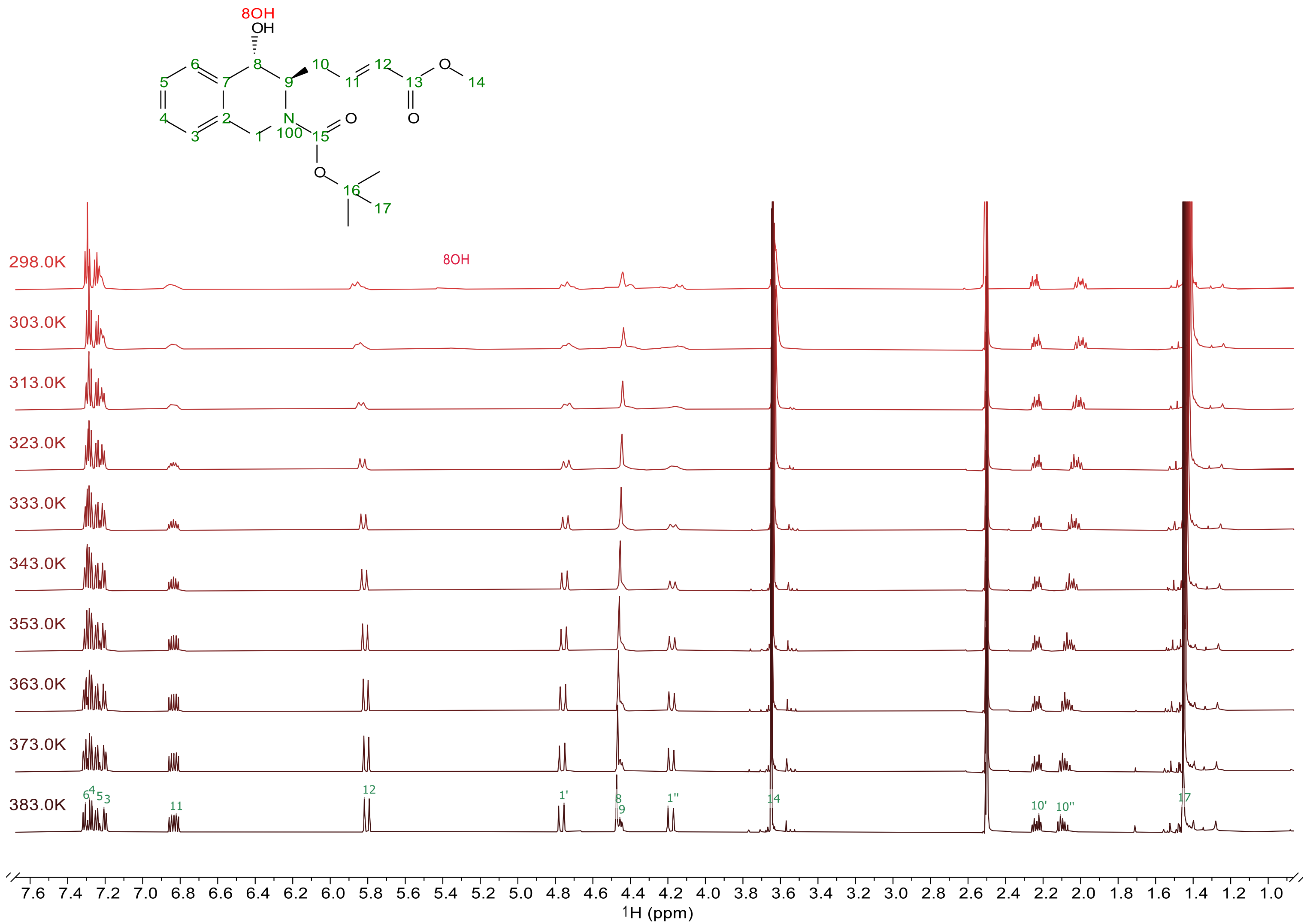

Procedures and Characterization Data

General Procedure D (for solid aldehydes)

A flame-dried Schlenk flask was charged under argon with tris(*trans*-1,2-bis(4-*tert*-butylphenyl)ethene)nickel(0) (Ni(*t*Bu-stb)$_3$) (9.36 mg, 10.0 µmol) and (*S*)-AntPhos (4.07 mg, 11.0 µmol). The flask was evacuated and refilled with argon (3 cycles). THF (0.75 mL) and triethylborane (1.0 M in THF, 0.25 mL, 0.25 mmol) were added and the resulting solution was cooled to 0 °C and stirred for 2 min before the respective substrate (0.10 mmol) was introduced under an argon counterflow. The flask was sealed and the mixture stirred at 0 °C (bath temperature) for the indicated time. The reaction was quenched via the work-up procedure **I** or **II** as specified in each case.

General Procedure E (for liquid aldehydes)

A flame-dried Schlenk flask was charged under argon with tris(*trans*-1,2-bis(4-*tert*-butylphenyl)ethene)nickel(0) (Ni(*t*Bu-stb)$_3$) (9.36 mg, 10.0 µmol) and (*S*)-AntPhos (4.07 mg, 11.0 µmol). The flask was evacuated and refilled with argon (3 cycles). THF (0.25 mL) and triethylborane (1.0 M in THF, 0.25 mL, 0.25 mmol) were added and the resulting solution was cooled to 0 °C and stirred for 2 min. A solution of the respective substrate (0.10 mmol) in THF (0.50 mL) was introduced under an argon counterflow, the flask was sealed and the mixture stirred at 0 °C (bath temperature) for the indicated time. The reaction was quenched via the work-up procedure **I** or **II** as specified in each case.

General Procedure F (using Et_2Zn)

A flame-dried Schlenk flask under argon was charged with tris(*trans*-1,2-bis(4-*tert*-butylphenyl)ethene)nickel(0) (Ni(*t*Bu-stb)$_3$) (9.36 mg, 10.0 µmol) and (*S*)-AntPhos (4.07 mg, 11.0 µmol). The flask was evacuated and refilled with argon (3 cycles) before THF (0.25 mL) and diethylzinc (1.0 M in hexane, 0.25 mL, 0.25 mmol) were added, and the resulting solution was cooled to 0 °C and stirred for 2 min. A solution of the respective substrate (0.10 mmol) in THF (0.50 mL) was introduced under an argon counterflow, the flask was sealed and the mixture stirred at 0 °C (bath temperature) for the indicated time. The reaction was quenched via the work-up procedure **I** or **II** as specified in each case.

Work-up Procedures

Oxidative Work-up Procedure (I): The reaction mixture was cooled to 0 °C and pH 7 phosphate buffer solution (1.0 mL), aq. H_2O_2 (30% *w/w*, ≈0.1 mL) and ethyl acetate (2.0 mL) were added. The mixture was vigorously stirred at room temperature for 30 min. Additional ethyl acetate (10 mL) and water (5 mL) were introduced, the organic phase was separated, and the aqueous layer was extracted with ethyl acetate (2 × 10 mL). The combined organic phases were washed with sat. aq. $Na_2S_2O_3$ (10 mL) and brine (10 mL), dried over $MgSO_4$, filtered and concentrated *in vacuo*. The residue was purified as specified for each compound.

Basic Work-up Procedure (II): The reaction mixture was cooled to 0 °C and sat. aq. $NaHCO_3$ solution (2.0 mL) and ethyl acetate (2.0 mL) were added. The mixture was vigorously stirred at room temperature for 30 min. The mixture was diluted with ethyl acetate (10 mL) and water (5 mL) and the aqueous layer was extracted with ethyl acetate (2 × 10 mL). The combined organic layers were washed with brine (15 mL), dried over $MgSO_4$, filtered and concentrated in vacuo. The residue was purified as specified for each compound.

Scale-Up Experiment: *tert*-Butyl (3*R*,4*S*)-4-hydroxy-3-((*E*)-4-methoxy-4-oxobut-2-en-1-yl)-3,4-dihydroisoquinoline-2(1*H*) carboxylate (8a).

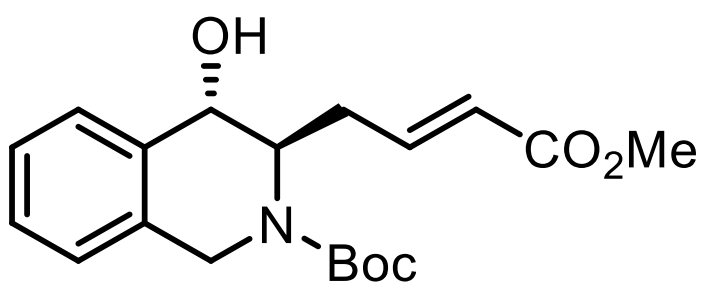

A flame-dried Schlenk flask was charged under argon with tris(*trans*-1,2-bis(4-*tert*-butylphenyl)ethene)nickel(0) (Ni(*t*Bu-stb)$_3$) (93.6 mg, 0.10 mmol) and (*S*)-AntPhos (40.7 mg, 0.11 mmol). The flask was evacuated and refilled with argon (3 cycles) before THF (7.5 mL) and triethylborane (1.0 M in THF, 2.50 mL, 2.50 mmol) were added. The resulting solution was cooled to 0 °C (bath temperature) and stirred for 2 min before methyl (2*E*,4*E*)-5-((*tert*-butoxycarbonyl)(2-formylbenzyl)amino)penta-2,4-dienoate **7a** (345 mg, 1.00 mmol) was introduced under an argon counterflow. A gas bubbler was connected to the Schlenk flask and the mixture was stirred under a very gentle flow of argon at 0 °C (bath temperature) for 16 h. pH 7 Phosphate buffer solution (10 mL), aq. H_2O_2 (30% *w/w* in water, ≈1.0 mL) and ethyl acetate (20 mL) were successively added at 0 °C and the mixture was allowed to reach room temperature while vigorously stirring for 30 min. Additional ethyl acetate (20 mL) and water (10 mL) were introduced, the organic phase was separated, and the aqueous layer was extracted with ethyl acetate (2 × 20 mL). The combined organic layers were washed with sat. aq. $Na_2S_2O_3$ (20 mL) and brine (20 mL), dried over $MgSO_4$, filtered and concentrated *in vacuo*. The residue was purified by flash chromatography (SiO_2, *n*-pentane/*tert*-butyl methyl ether, 1:1) to give the title compound as a colorless oil (302 mg, 87%, 92% ee).

$[\alpha]_D^{20}$ = +60.6 (c = 0.50, $CHCl_3$); ^{1}H NMR (600 MHz, [D$_6$]-DMSO, 383K): δ = 7.31 (dd, *J* = 7.3, 1.6 Hz, 1H), 7.28 (td, *J* = 7.4, 1.6 Hz, 1H), 7.27 – 7.21 (m, 1H), 7.20 (d, *J* = 7.4 Hz, 1H), 6.83 (dt, *J* = 15.6, 7.8 Hz, 1H), 5.81 (dt, *J* = 15.6, 1.5 Hz, 1H), 4.77 (d, *J* = 17.1 Hz, 1H), 4.47 (d, *J* = 2.7 Hz, 1H), 4.46 (ddd, *J* = 8.9, 5.4, 2.7 Hz, 1H), 4.19 (d, *J* = 17.1 Hz, 1H), 3.65 (s, 3H), 2.24 (dddd, *J* = 14.6, 6.9, 5.4, 1.6 Hz, 1H), 2.10 (dddd, *J* = 14.6, 8.8, 7.8, 1.5 Hz, 1H), 1.45 (s, 9H); ^{13}C NMR (151 MHz, [D$_6$]-DMSO, 383K): δ = 165.1, 154.3, 144.9, 134.3, 132.3, 128.9, 127.1, 125.9, 125.4, 121.9, 78.5, 68.3, 55.4, 50.2, 41.8, 32.9, 27.5; IR (ATR): ṽ = 3433, 2925, 1722, 1688, 1406, 1365, 1241, 1160, 1123, 1031, 732 cm^{-1}; HRMS (ESI$^+$): *m/z*: calcd. for $C_{19}H_{25}NO_5Na$ [M+Na]$^+$: 370.16249, found: 370.16251.

The ee was determined by HPLC analysis: Chiralpak 150 mm IB-N-3, 3 µm, 4.6 mm ∅, acetonitrile/water = 30:70, v = 1.0 mL/min, λ = 220 nm, t(minor) = 20.00 min, t(major) = 25.06 min (enantioenriched sample: left, racemic sample: right).

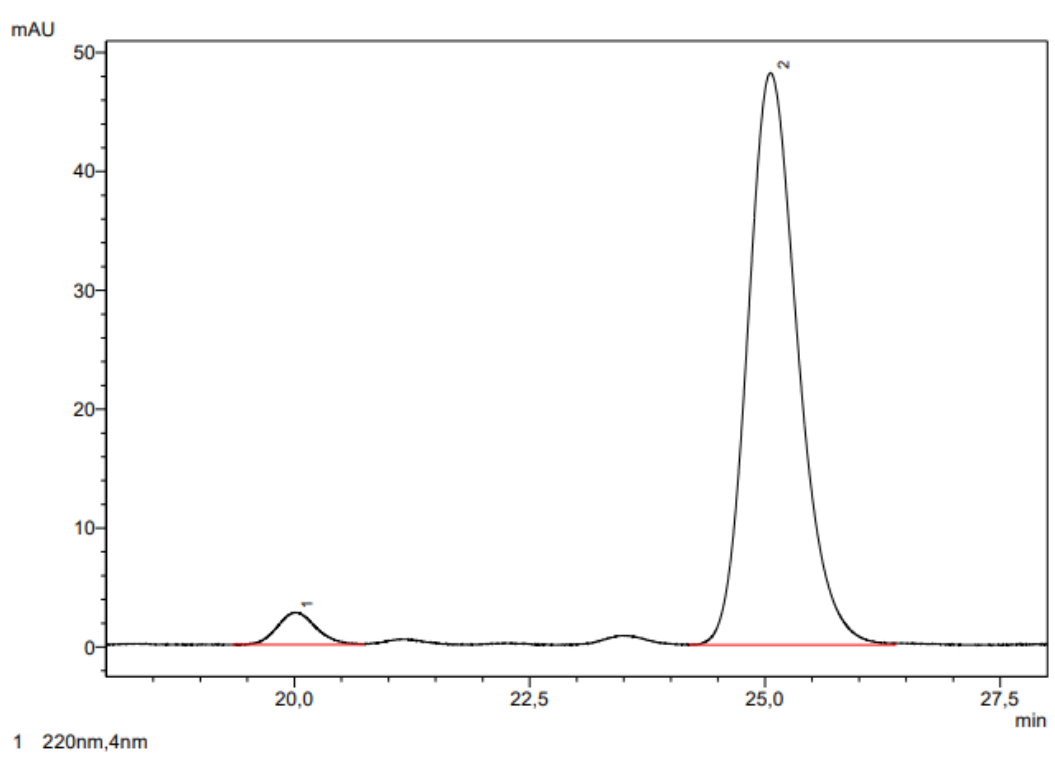

PDA Ch1 220nm

Peak #	Ret. Time	Area %	Name
1	20,00	4,20	1. Enantiomer
2	25,06	95,80	2. Enantiomer 91.6 % ee
Total		100,00	

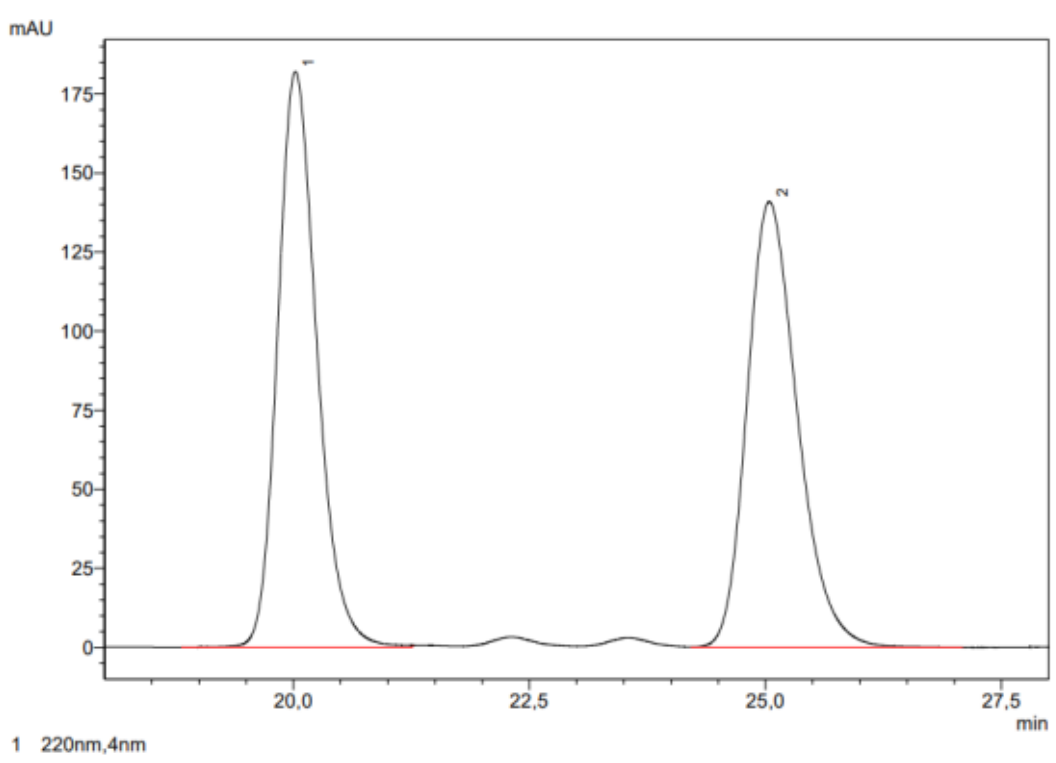

PDA Ch1 220nm

Peak #	Ret. Time	Area %	Name
1	20,02	50,21	1. Enantiomer
2	25,04	49,79	2. Enantiomer
Total		100,00	

Benzyl (3*R*,4*S*)-4-hydroxy-3-((*E*)-4-methoxy-4-oxobut-2-en-1-yl)-3,4-dihydroisoquinoline-2(1*H*)-carboxylate (8b).

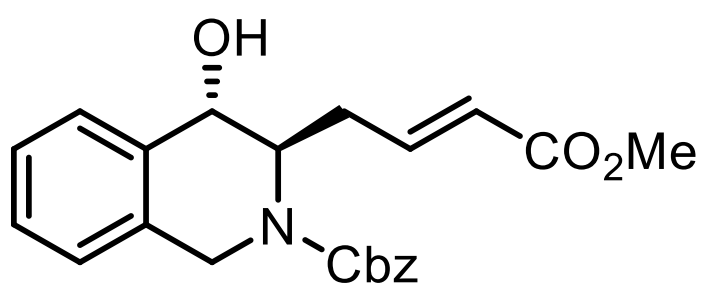

The title compound was prepared according to the general procedure **D** followed by oxidative workup **I**, using methyl (2*E*,4*E*)-5-(((benzyloxy)carbonyl)(2-formylbenzyl)amino)penta-2,4-dienoate **7b** (75.9 mg, 0.20 mmol); the reaction time was 16 h. The crude material was purified by flash chromatography (SiO_2, *n*-pentane/ethyl acetate, 1.5:1) to give the product as a colorless oil (68.3 mg, 90%, 93% ee).

$[\alpha]_D^{20}$ = +22.5 (c = 0.48, $CHCl_3$); ^1H NMR (600 MHz, [D_6]-DMSO, 383K): δ = 7.41 – 7.35 (m, 4H), 7.34 – 7.30 (m, 2H), 7.29 (dd, *J* = 7.4, 1.6 Hz, 1H), 7.27 – 7.24 (m, 1H), 7.23 – 7.21 (m, 1H), 6.82 (ddd, *J* = 15.6, 7.7, 7.0 Hz, 1H), 5.81 (dt, *J* = 15.7, 1.5 Hz, 1H), 5.17 (d, *J* = 12.7 Hz, 1H), 5.16 – 5.11 (m, 1H), 5.06 (br, 1H), 4.87 (d, *J* = 17.3 Hz, 1H), 4.55 (ddd, *J* = 8.5, 6.0, 2.6 Hz, 1H), 4.50 (d, *J* = 2.6 Hz, 1H), 4.30 (d, *J* = 17.0 Hz, 1H), 3.65 (s, 3H), 2.25 (dddd, *J* = 14.7, 6.9, 6.0, 1.6 Hz, 1H), 2.17 (dddd, *J* = 14.8, 8.4, 7.6, 1.5 Hz, 1H); ^{13}C NMR (151 MHz, [D_6]-DMSO, 383K): δ = 165.1, 155.0, 144.4, 136.5, 134.2, 131.9, 129.0, 127.7, 127.2, 127.0, 126.7, 126.1, 125.4, 122.1, 68.1, 65.9, 55.7, 50.3, 42.0, 32.7; IR (ATR): ṽ = 3410, 1699, 1420, 1322, 1272, 1227, 1123, 1032, 742 cm^{-1}; HRMS (ESI^+): *m/z*: calcd. for $C_{22}H_{23}NO_5Na$ $[M+Na]^+$: 404.14684, found: 404.14694.

The ee was determined by HPLC analysis: Chiralpak 150 mm IG-N-3, 3 µm, 4.6 mm ∅, acetonitrile/water = 45:55, v = 1.0 mL/min, λ = 215 nm, t(minor) = 17.66 min, t(major) = 22.89 min (enantioenriched sample: left, racemic sample: right).

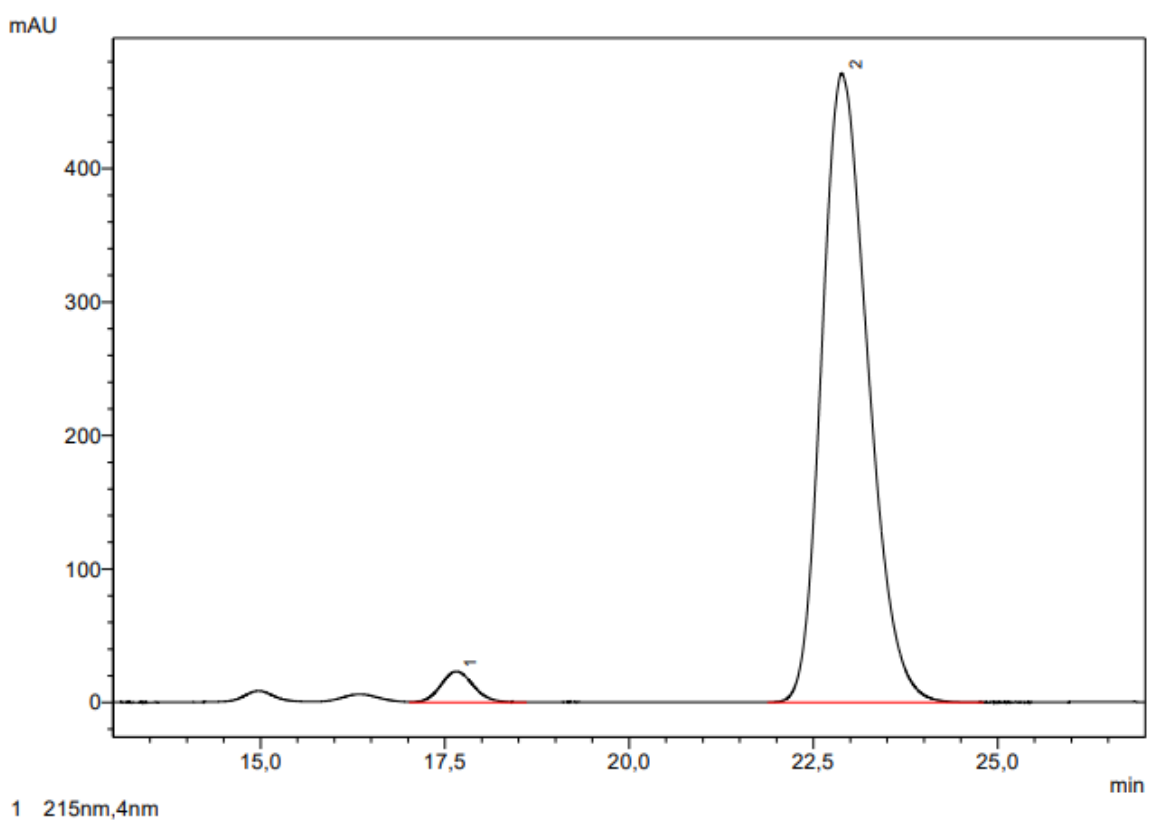

PDA Ch1 215nm

Peak #	Ret. Time	Area %	Name
1	17,66	3,37	1. Enantiomer
2	22,89	96,63	2. Enantiomer 93.3 % ee
Total		100,00	

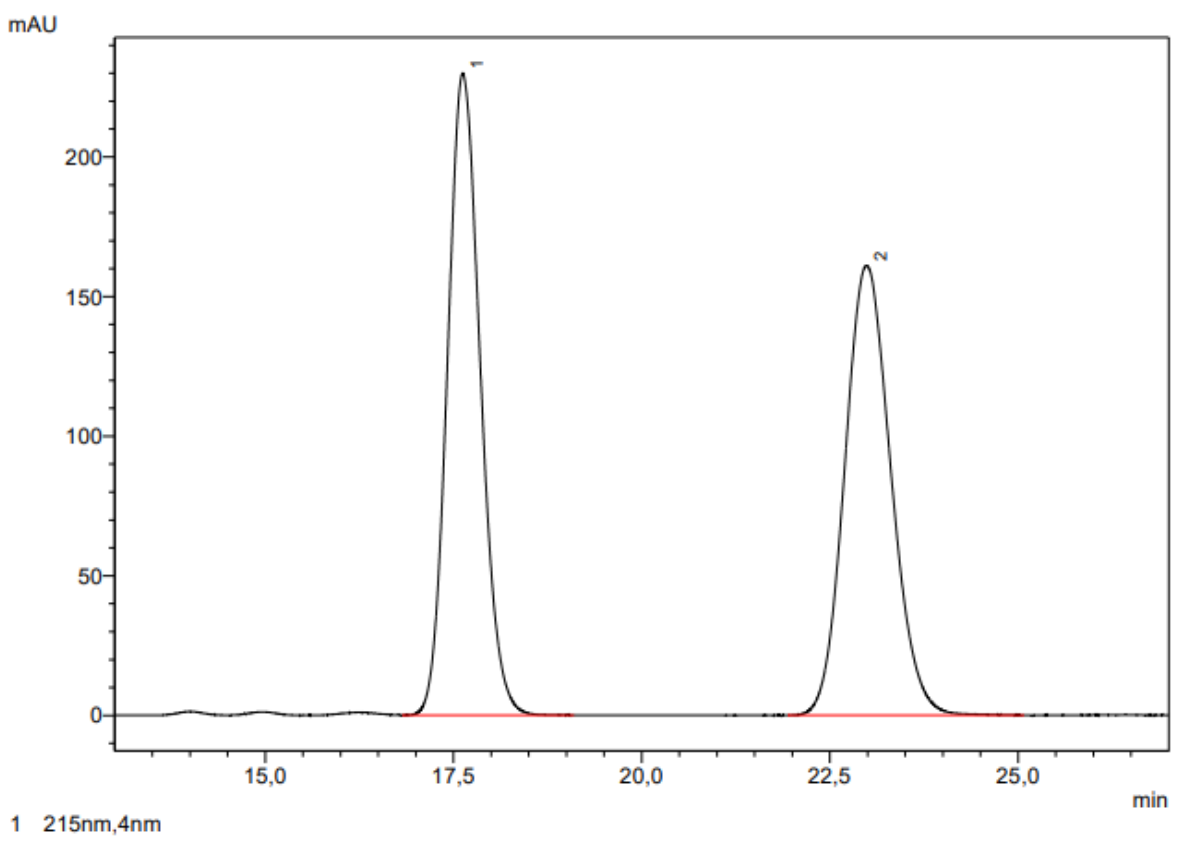

PDA Ch1 215nm

Peak #	Ret. Time	Area %	Name
1	17,62	50,42	1. Enantiomer
2	22,99	49,58	2. Enantiomer
Total		100,00	

tert-Butyl (3*R*,4*S*)-8-fluoro-4-hydroxy-3-((*E*)-4-methoxy-4-oxobut-2-en-1-yl)-3,4-dihydroisoquinoline-2(1*H*)-carboxylate (8c).

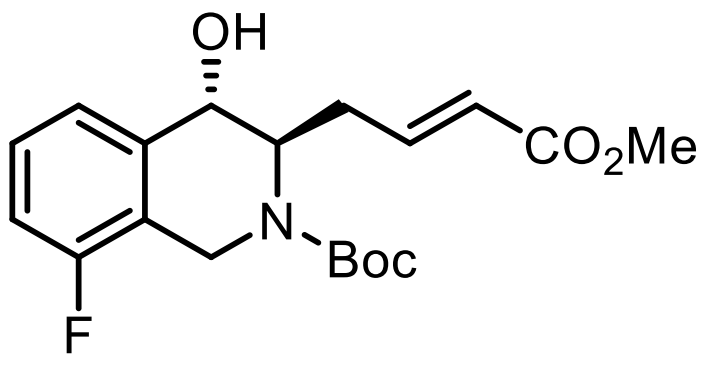

The title compound was prepared according to the general procedure **E** followed by oxidative workup **I**, using methyl (2*E*,4*E*)-5-((*tert*-butoxycarbonyl)(2-fluoro-6-formylbenzyl)amino)penta-2,4-dienoate **7c** (36.3 mg, 0.10 mmol); the reaction time was 16 h. The crude material was purified by flash chromatography (SiO_2, *n*-pentane/ethyl acetate, 1.5:1) to give the product as a colorless oil (27.6 mg, 76%, 92% ee).

$[\alpha]_D^{20}$ = +46.3 (c = 0.54, $CHCl_3$); ^{1}H NMR (600 MHz, [D_6]-DMSO, 383K): δ = 7.35 – 7.27 (m, 1H), 7.20 – 7.15 (m, 1H), 7.10 (ddd, *J* = 10.1, 8.2, 1.1 Hz, 1H), 6.83 (ddd, *J* = 15.6, 7.9, 6.8 Hz, 1H), 5.84 (dt, *J* = 15.6, 1.5 Hz, 1H), 5.10 (d, *J* = 4.5 Hz, 1H), 4.80 (d, *J* = 17.6 Hz, 1H), 4.51 (d, *J* = 4.0 Hz, 1H), 4.49 (dd, *J* = 5.3, 2.5 Hz, 1H), 4.14 (d, *J* = 17.8 Hz, 1H), 3.65 (s, 3H), 2.26 (dddd, *J* = 14.7, 6.8, 5.3, 1.6 Hz, 1H), 2.12 (dddd, *J* = 14.8, 9.2, 7.9, 1.4 Hz, 1H), 1.46 (s, 9H); ^{13}C NMR (151 MHz, [D_6]-DMSO, 383K): δ = 165.1, 157.9 (d, *J* = 244.4 Hz), 154.2, 144.6, 136.9 (d, *J* = 3.5 Hz), 127.4 (d, *J* = 8.3 Hz), 125.0 (d, *J* = 3.2 Hz), 122.0, 119.6 (d, *J* = 15.5 Hz), 113.3 (d, *J* = 20.7 Hz), 78.8, 67.6 (d, *J* = 2.7 Hz), 55.1, 50.2, 36.1 (d, *J* = 6.1 Hz), 32.3, 27.5; ^{19}F NMR (585 MHz, [D_6]-DMSO, 383K): δ = –120.4 (dd, *J* = 10.3, 5.8 Hz); IR (ATR): ṽ = 3433, 2977, 1722, 1692, 1409, 1366, 1319, 1244, 1162, 1120, 1039, 978 cm^{-1}; HRMS (ESI$^+$): *m/z*: calcd. for $C_{19}H_{24}FNO_5Na$ $[M+Na]^+$: 388.15307, found: 388.15309.

The ee was determined by HPLC analysis: Chiralpak 150 mm IG-3, 3 µm, 4.6 mm ∅, acetonitrile/water = 45:55, v = 1.0 mL/min, λ = 220 nm, t(minor) = 7.18 min, t(major) = 12.56 min (enantioenriched sample: left, racemic sample: right).

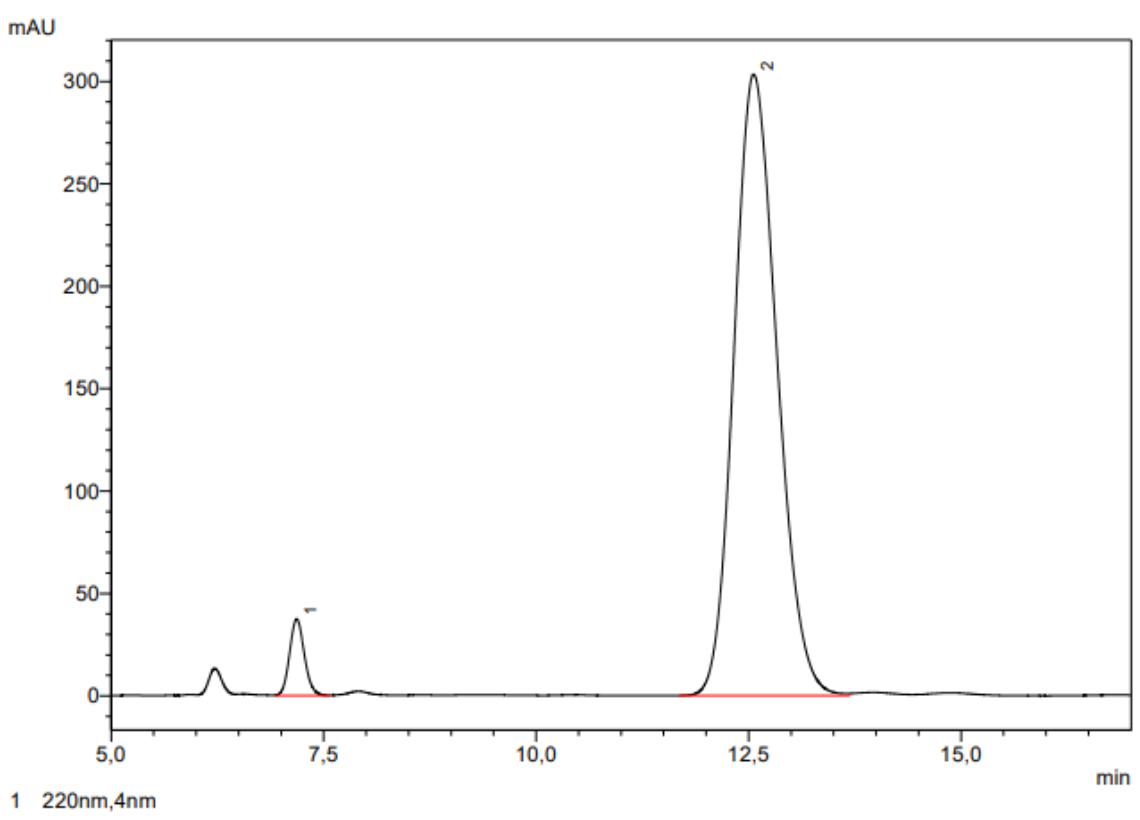

PDA Ch1 220nm

Peak #	Ret. Time	Area %	Name
1	7,18	4,09	1. Enantiomer
2	12,56	95,91	2. Enantiomer 91.8 % ee
Total		100,00	

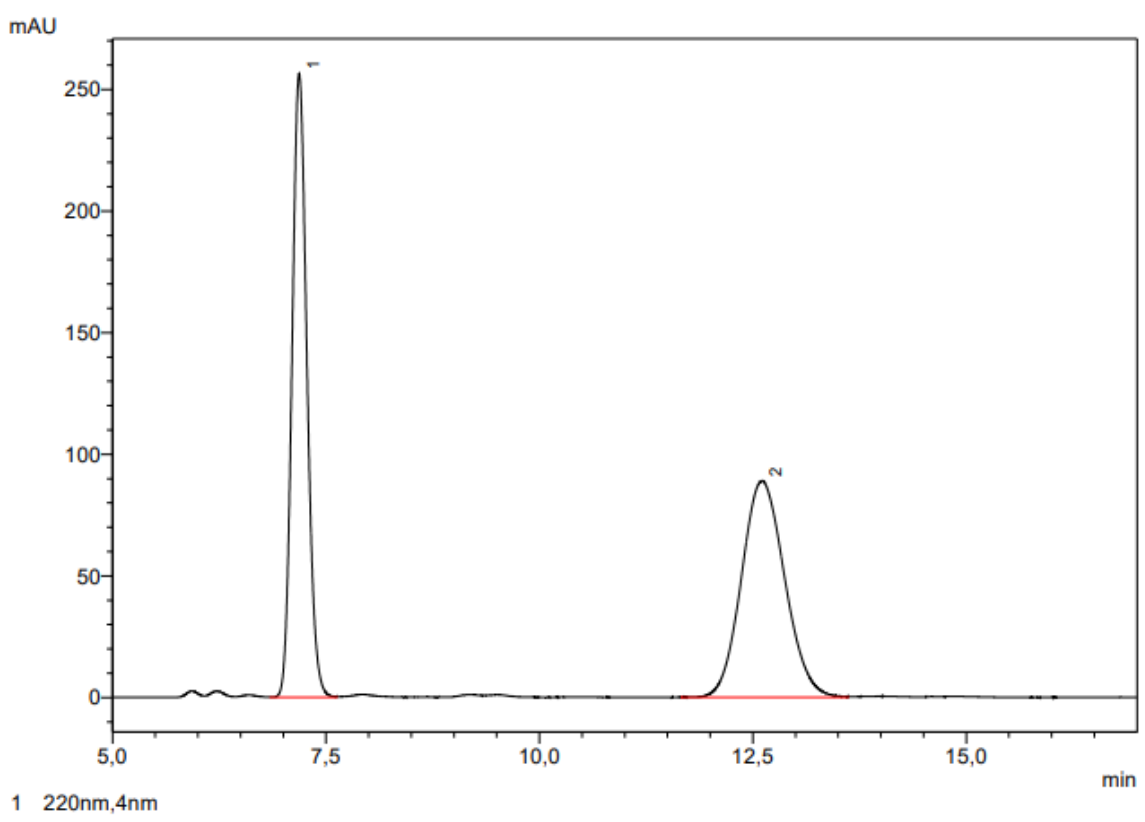

PDA Ch1 220nm

Peak #	Ret. Time	Area %	Name
1	7,19	49,81	1. Enantiomer
2	12,61	50,19	2. Enantiomer
Total		100,00	

tert-Butyl (3*R*,4*S*)-7-fluoro-4-hydroxy-3-((*E*)-4-methoxy-4-oxobut-2-en-1-yl)-3,4-dihydroisoquinoline-2(1*H*)-carboxylate (8d).

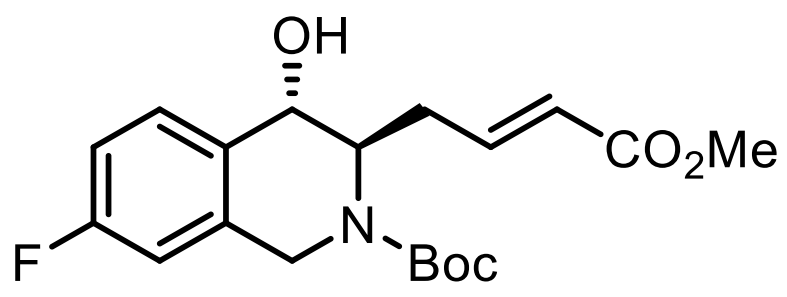

The title compound was prepared according to the general procedure **D** followed by oxidative workup **I**, using methyl (2*E*,4*E*)-5-((*tert*-butoxycarbonyl)(5-fluoro-2-formylbenzyl)amino)penta-2,4-dienoate **7d** (36.3 mg, 0.10 mmol); the reaction time was 16 h. The crude material was purified by flash chromatography (SiO_2, *n*-pentane/ethyl acetate, 2:1) to give the product as a colorless oil (28.0 mg, 77%, 93% ee).

$[\alpha]_D^{20}$ = +42.2 (c = 0.40, $CHCl_3$); ^{1}H NMR (600 MHz, [D$_6$]-DMSO, 383K): δ = 7.38 – 7.32 (m, 1H), 7.05 – 7.01 (m, 2H), 6.82 (ddd, *J* = 15.6, 7.8, 6.8 Hz, 1H), 5.82 (dt, *J* = 15.7, 1.5 Hz, 1H), 4.99 (br, 1H), 4.78 (d, *J* = 17.4 Hz, 1H), 4.47 (d, *J* = 1.8 Hz, 1H), 4.47 – 4.44 (m, 1H), 4.18 (d, *J* = 17.6 Hz, 1H), 3.65 (s, 3H), 2.23 (dddd, *J* = 14.7, 6.9, 5.4, 1.6 Hz, 1H), 2.16 – 2.04 (m, 1H), 1.45 (s, 9H); ^{13}C NMR (151 MHz, [D$_6$]-DMSO, 383K): δ = 165.1, 161.1 (d, *J* = 243.8 Hz), 154.2, 144.7, 134.9 (d, *J* = 8.1 Hz), 131.1 (d, *J* = 8.4 Hz), 130.6 (d, *J* = 2.9 Hz), 121.9, 112.9 (d, *J* = 21.4 Hz), 111.9 (d, *J* = 22.1 Hz), 78.6, 67.6, 55.4, 50.3, 41.7, 32.7, 27.5; ^{19}F NMR (585 MHz, [D$_6$]-DMSO, 383K): δ = –114.6 (m); IR (ATR): ṽ = 3422, 2926, 1723, 1694, 1410, 1367, 1251, 1163, 1121, 1042 cm^{-1}; HRMS (ESI$^+$): *m/z*: calcd. for $C_{19}H_{24}FNO_5Na$ [M+Na]$^+$: 388.15307, found: 388.15287.

The ee was determined by HPLC analysis: Chiralpak 150 mm IG-3, 3 µm, 4.6 mm ∅, acetonitrile/water = 40:60, v = 1.0 mL/min, λ = 220 nm, t(minor) = 8.32 min, t(major) = 9.30 min (enantioenriched sample: left, racemic sample: right).

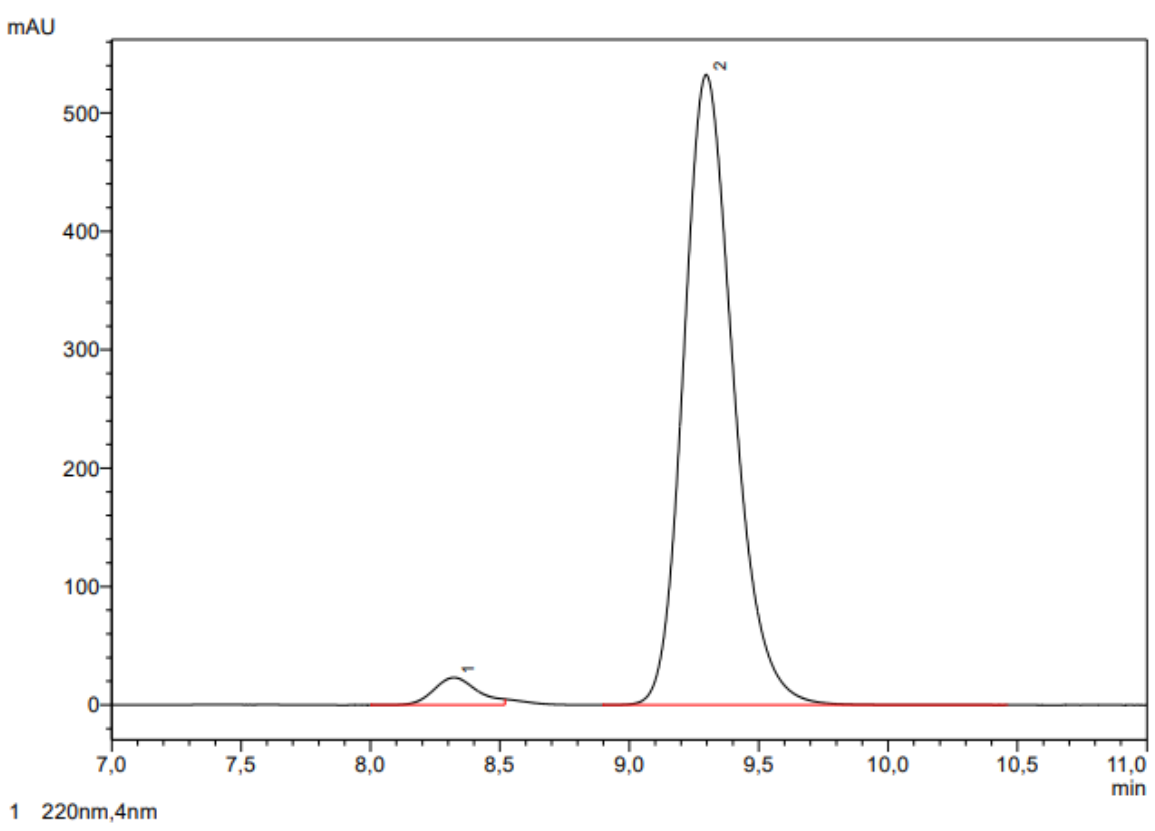

PDA Ch1 220nm

Peak #	Ret. Time	Area %	Name
1	8,32	3,70	1. Enantiomer
2	9,30	96,30	2. Enantiomer 92.6 % ee
Total		100,00	

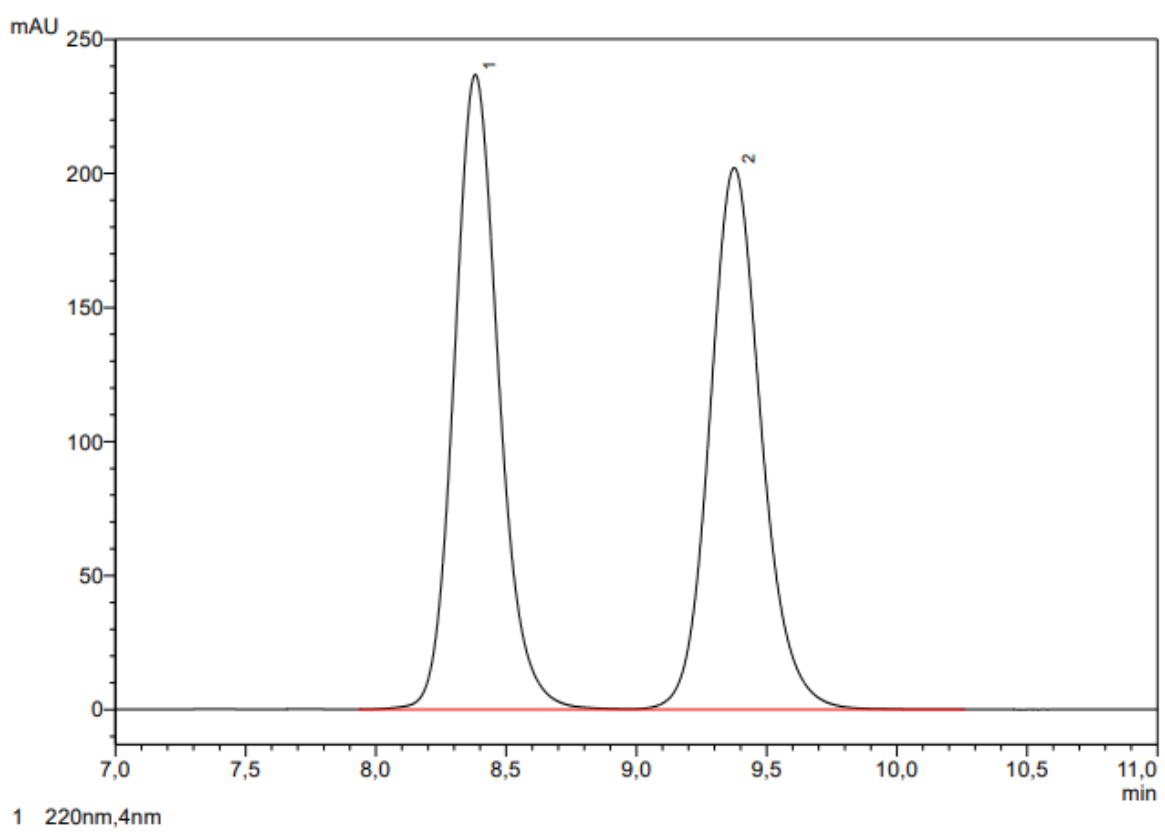

PDA Ch1 220nm

Peak #	Ret. Time	Area %	Name
1	8,38	50,35	1. Enantiomer
2	9,38	49,65	2. Enantiomer
Total		100,00	

***tert*-Butyl (3*R*,4*S*)-6-fluoro-4-hydroxy-3-((*E*)-4-methoxy-4-oxobut-2-en-1-yl)-3,4-dihydroisoquinoline-2(1*H*)-carboxylate (8e).**

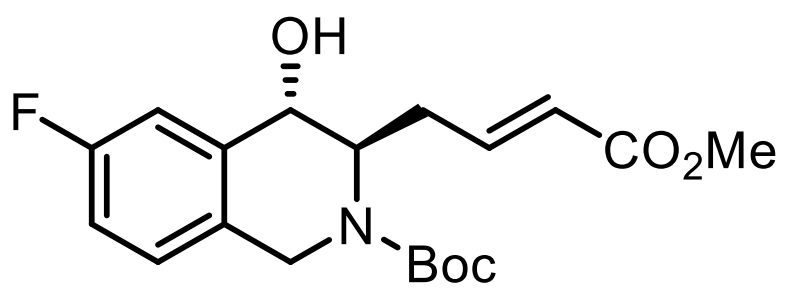

The title compound was prepared according to the general procedure **D** followed by oxidative workup **I**, using methyl (2*E*,4*E*)-5-((*tert* butoxycarbonyl)(4-fluoro-2-formylbenzyl)amino)penta-2,4-dienoate **7e** (36.3 mg, 0.10 mmol); the reaction time was 16 h. The crude material was purified by flash chromatography (SiO_2, *n*-pentane/ethyl acetate, 2:1) to give the product as a colorless oil (32.8 mg, 90%, 97% ee).

$[\alpha]_D^{20}$ = +60.2 (c = 0.56, $CHCl_3$); ^{1}H NMR (600 MHz, [D_6]-DMSO, 383K): δ = 7.25 (ddt, *J* = 8.4, 5.5, 0.4 Hz, 1H), 7.11 (ddt, *J* = 9.4, 2.8, 0.5 Hz, 1H), 7.10 – 7.05 (m, 1H), 6.83 (ddd, *J* = 15.6, 7.9, 6.8 Hz, 1H), 5.83 (dt, *J* = 15.6, 1.5 Hz, 1H), 5.14 (br, 1H), 4.74 (dt, *J* = 16.9, 1.3 Hz, 1H), 4.47 (d, *J* = 2.8 Hz, 1H), 4.42 (ddd, *J* = 8.6, 5.5, 2.8 Hz, 1H), 4.15 (d, *J* = 16.8 Hz, 1H), 3.65 (s, 3H), 2.27 (dddd, *J* = 14.7, 7.0, 5.5, 1.6 Hz, 1H), 2.12 (dddd, *J* = 14.7, 9.1, 7.9, 1.4 Hz, 1H), 1.45 (s, 9H); ^{13}C NMR (151 MHz, [D_6]-DMSO, 383K): δ = 165.1, 160.4 (d, *J* = 242.9 Hz), 154.2, 144.7, 136.9 (d, *J* = 7.0 Hz), 128.5 (d, *J* = 2.9 Hz), 127.4 (d, *J* = 8.0 Hz), 122.0, 114.9 (d, *J* = 21.1 Hz), 114.0 (d, *J* = 21.6 Hz), 78.6, 68.0 (d, *J* = 1.5 Hz), 55.2, 50.3, 41.3, 32.8, 27.5; ^{19}F NMR (585 MHz, [D_6]-DMSO, 383K): δ = –116.4 (m); IR (ATR): ṽ = 3425, 2977, 1722, 1690, 1409, 1367, 1254, 1163, 1041, 873 cm^{-1}; HRMS (ESI$^+$): *m/z*: calcd. for $C_{19}H_{24}FNO_5Na$ [M+Na]$^+$: 388.15307, found: 388.15286.

The ee was determined by HPLC analysis: Chiralpak 150 mm IG-3, 3 µm, 4.6 mm ∅, acetonitrile/water = 45:55, v = 1.0 mL/min, λ = 220 nm, t(minor) = 8.87 min, t(major) = 15.79 min (enantioenriched sample: left, racemic sample: right).

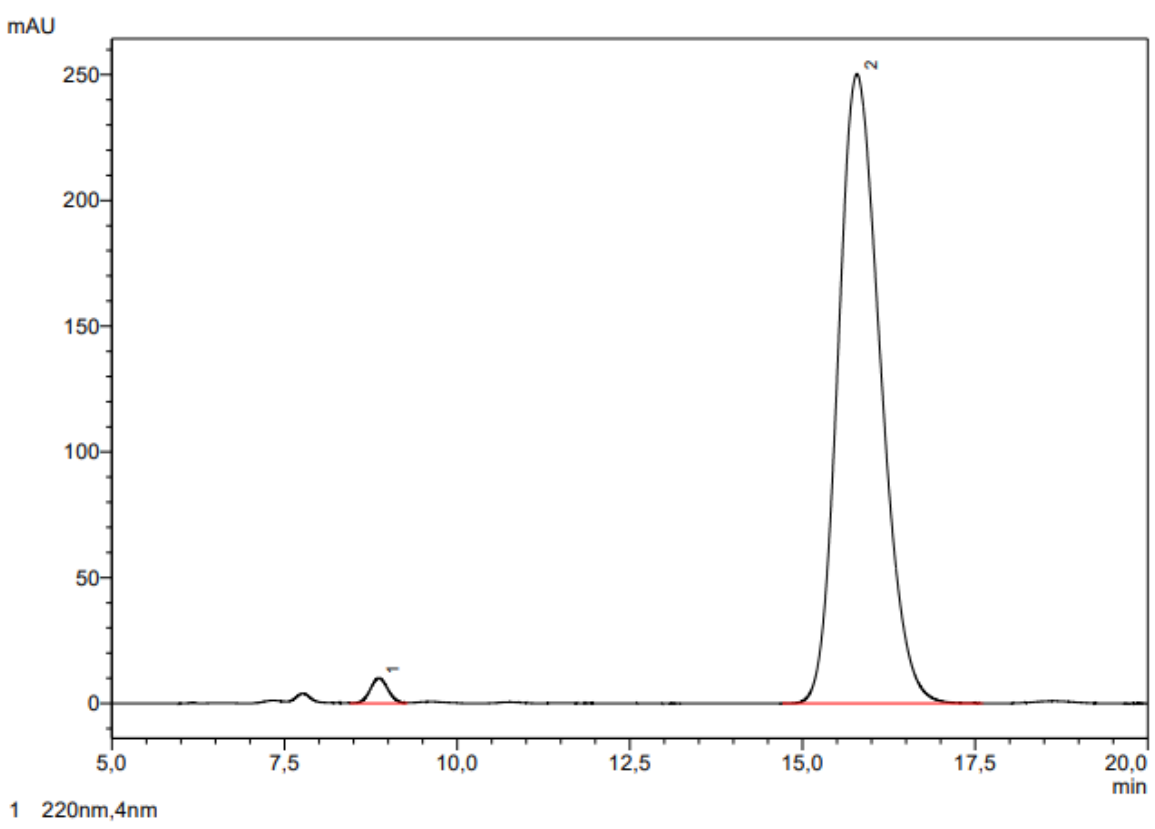

PDA Ch1 220nm

Peak #	Ret. Time	Area %	Name
1	8,87	1,69	1. Enantiomer
2	15,79	98,31	2. Enantiomer 96.6 % ee
Total		100,00	

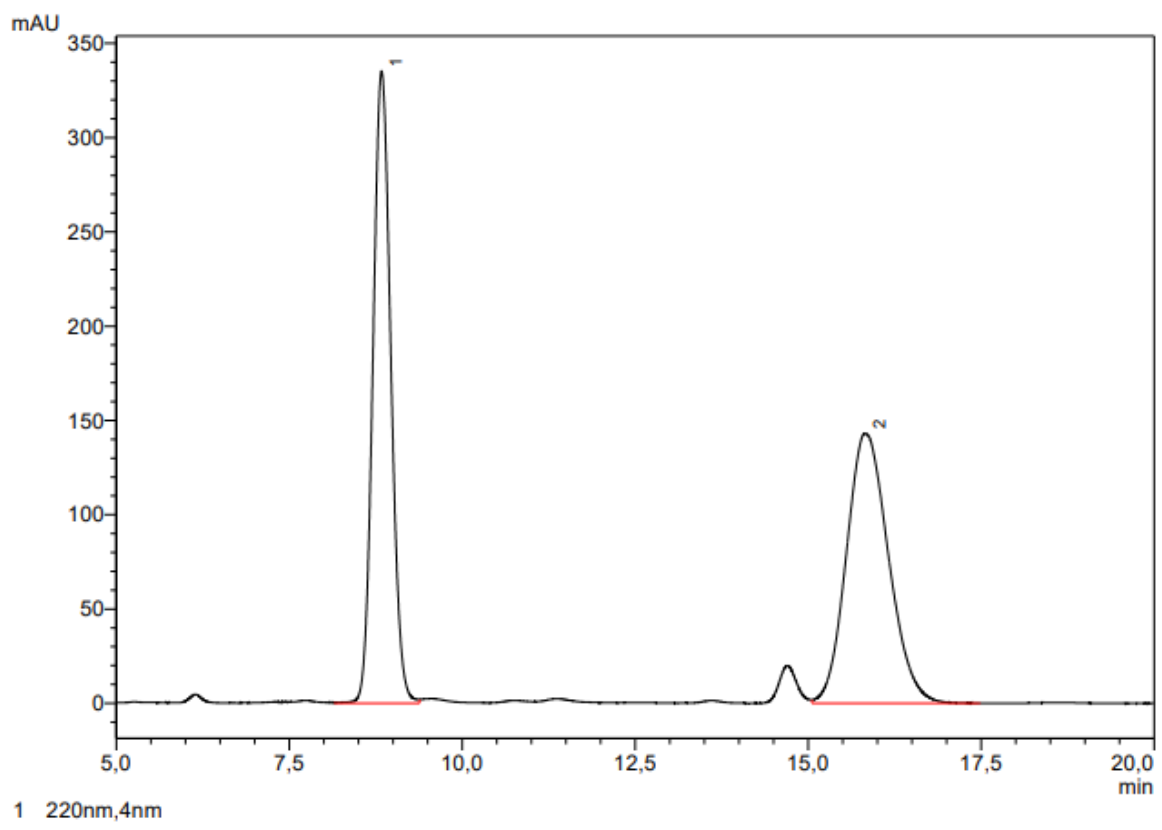

PDA Ch1 220nm

Peak #	Ret. Time	Area %	Name
1	8,84	49,89	1. Enantiomer
2	15,82	50,11	2. Enantiomer
Total		100,00	

tert-Butyl (3*R*,4*S*)-5-fluoro-4-hydroxy-3-((*E*)-4-methoxy-4-oxobut-2-en-1-yl)-3,4-dihydro-isoquinoline-2(1*H*)-carboxylate (8f) and Double Bond Isomer 8f' (rr ≈ 4:1)

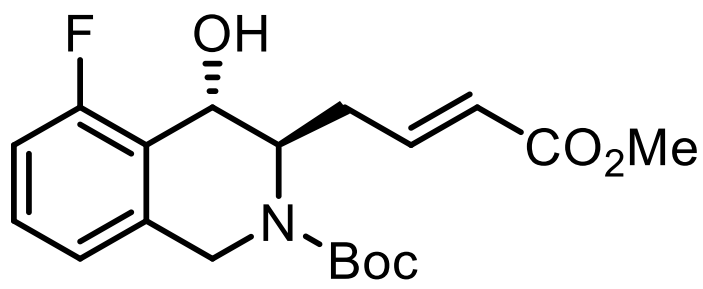

The title compound was prepared according to the general procedure **D** (reaction was performed at room temperature) followed by oxidative workup **I,** using methyl (2*E*,4*E*)-5-((*tert*-butoxycarbonyl)(3-fluoro-2-formylbenzyl)amino)penta-2,4-dienoate **7f** (36.3 mg, 0.10 mmol); the reaction time was 16 h. The crude material was purified by flash chromatography (SiO_2, *n*-pentane/ethyl acetate, 2:1) to give the product as a colorless oil (31.5 mg, 86%, 55% ee, rr ≈ 4:1). ***Note:*** *The product was obtained as a ~4:1 mixture of alkene isomers that could not be separated.*

$[\alpha]_D^{20}$ = +55.0 (c = 0.40, $CHCl_3$); ^1H NMR (600 MHz, [D_6]-DMSO, 383K): δ = 7.32 (ddd, *J* = 8.2, 7.7, 5.8 Hz, 1H), 7.10 – 7.05 (m, 1H), 7.05 – 6.99 (m, 1H), 6.81 (ddd, *J* = 15.7, 7.8, 6.8 Hz, 1H), 5.82 (dt, *J* = 15.6, 1.5 Hz, 1H), 4.81 (d, *J* = 17.8 Hz, 1H), 4.77 (d, *J* = 2.2 Hz, 1H), 4.55 (t, *J* = 7.2 Hz, 1H), 4.22 (d, *J* = 17.8 Hz, 1H), 3.65 (s, 3H), 2.22 (dddd, *J* = 14.7, 6.9, 5.5, 1.6 Hz, 1H), 2.06 (dddd, *J* = 14.7, 9.3, 7.8, 1.4 Hz, 1H), 1.46 (s, 9H); ^{13}C NMR (151 MHz, [D_6]-DMSO, 383K): δ = 165.1, 160.4 (d, *J* = 246.3 Hz), 154.2, 144.5, 135.0 (d, *J* = 4.7 Hz), 128.6 (d, *J* = 8.9 Hz), 122.0, 121.6, 121.6 – 121.4 (m), 112.3 (d, *J* = 21.7 Hz), 78.7, 61.5 (d, *J* = 3.6 Hz), 55.0, 50.3, 41.2, 32.3, 27.5; ^{19}F NMR (585 MHz, [D_6]-DMSO, 383K): δ = –121.6 (dd, *J* = 9.7, 5.8 Hz); IR (ATR): ṽ = 3436, 2976, 1723, 1689, 1408, 1366, 1249, 1165, 1117, 1040, 783 cm^{-1}; HRMS (ESI$^+$): *m/z*: calcd. for $C_{19}H_{24}FNO_5Na$ [M+Na]$^+$: 388.15307, found: 388.15285.

The ee was determined by HPLC analysis: Chiralpak 150 mm IA-3, 3 µm, 4.6 mm ∅, *n*-heptane/ethanol = 90:10, v = 1.0 mL/min, λ = 210 nm, **for major regioisomer:** t(minor) = 8.05 min, t(major) = 12.88 min, **for minor regioisomer:** t(minor) = 6.11 min, t(major) = 9.53 min, (enantioenriched sample: left, racemic sample: right).

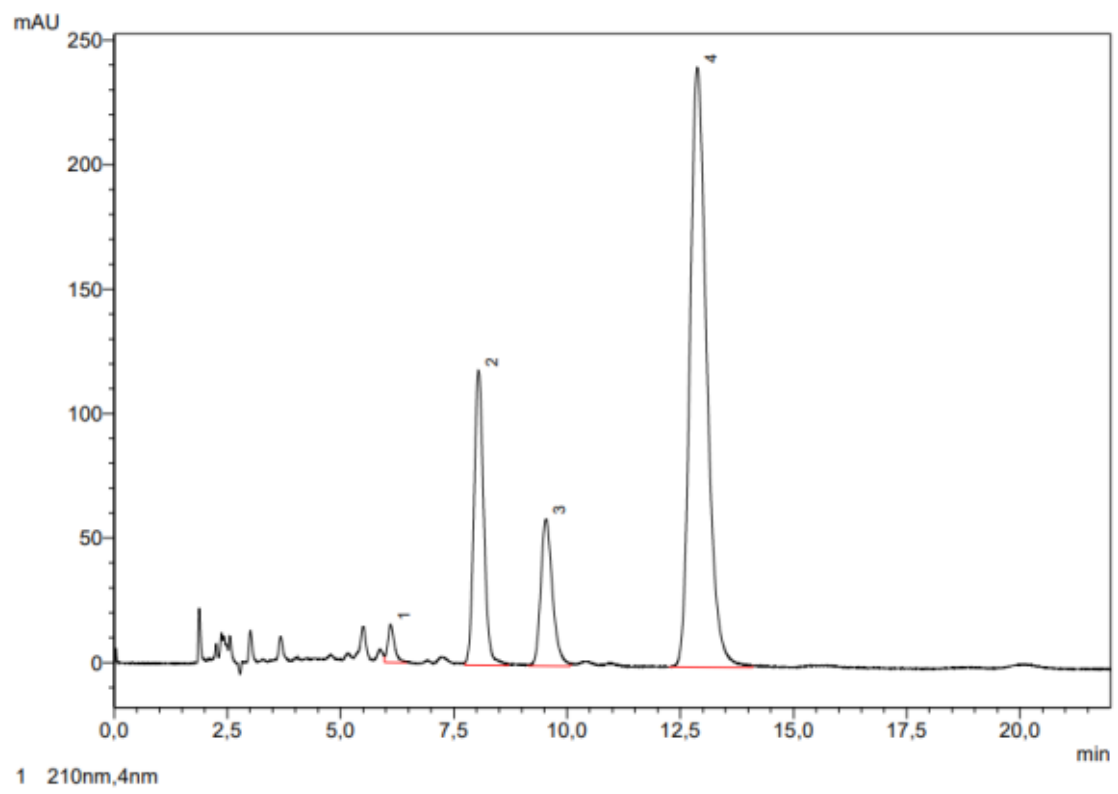

PDA Ch1 210nm

Peak #	Ret. Time	Area %	Name
1	6,11	1,70	1. Enantiomer Isomer 1
2	8,05	19,43	1. Enantiomer Isomer 2
3	9,53	11,57	2. Enantiomer Isomer 1 74.3 % ee
4	12,88	67,30	2. Enantiomer Isomer 2 55.2 % ee
Total		100,00	

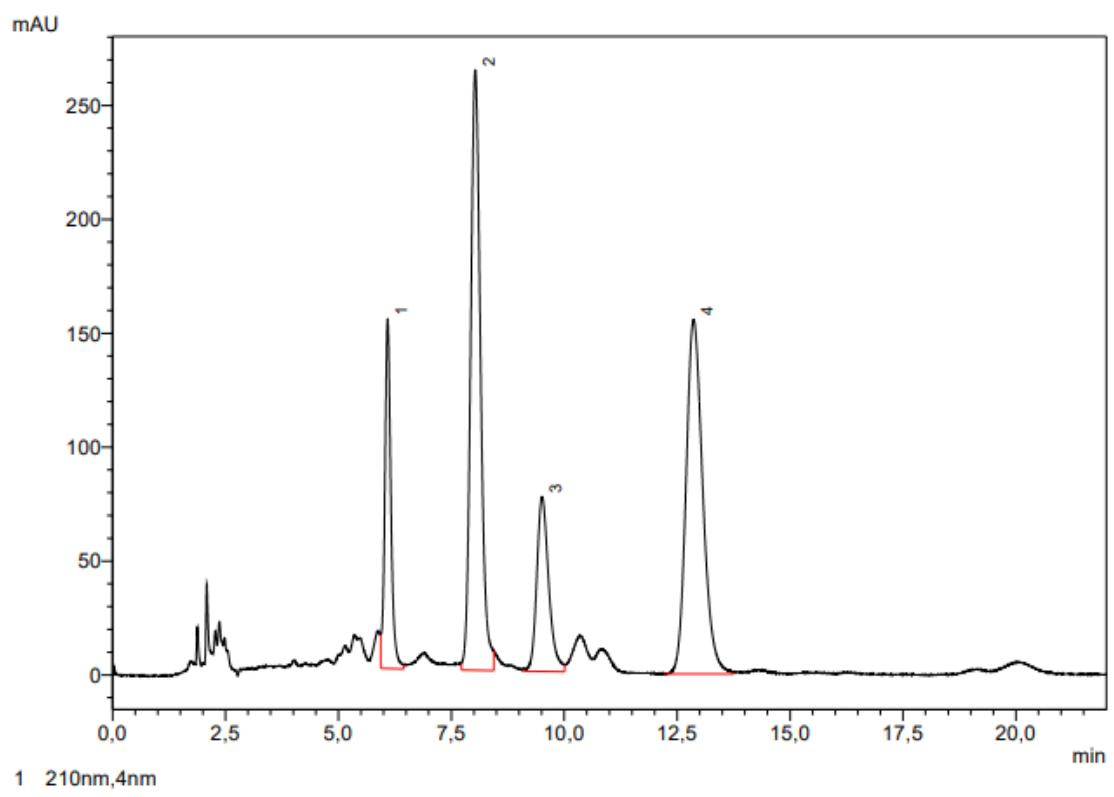

PDA Ch1 210nm

Peak #	Ret. Time	Area %	Name
1	6,09	13,16	1. Enantiomer Isomer 1
2	8,03	36,95	1. Enantiomer Isomer 2
3	9,51	13,06	2. Enantiomer Isomer 1
4	12,87	36,83	2. Enantiomer Isomer 2
Total		100,00	

tert-Butyl (3_R_,4_S_)-4-hydroxy-7-methoxy-3-((_E_)-4-methoxy-4-oxobut-2-en-1-yl)-3,4-dihydro-isoquinoline-2(1_H_)-carboxylate (8g).

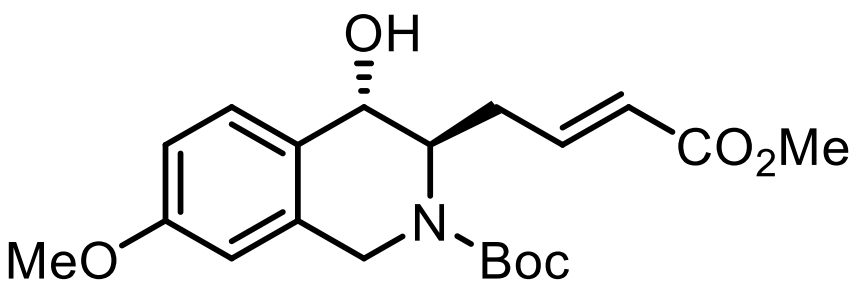

The title compound was prepared according to the general procedure **D** followed by oxidative workup **I**, using methyl (2*E*,4*E*)-5-((*tert*-butoxycarbonyl)(2-formyl-5-methoxybenzyl)-amino)penta-2,4-dienoate **7g** (37.5 mg, 0.10 mmol); the reaction time was 48 h. The crude material was purified by flash chromatography (SiO_2, *n*-pentane/ethyl acetate, 1.5:1) to give the product as a colorless sticky solid (25.3 mg, 67%, 71% ee).

$[\alpha]_D^{20}$ = +33.6 (c = 0.58, $CHCl_3$); ^1H NMR (600 MHz, [D_6]-DMSO, 383K): δ = 7.21 (dt, *J* = 8.3, 0.5 Hz, 1H), 6.86 – 6.75 (m, 3H), 5.80 (dt, *J* = 15.6, 1.5 Hz, 1H), 4.79 (s, 1H), 4.74 (d, *J* = 17.1 Hz, 1H), 4.47 – 4.44 (m, 1H), 4.42 (d, *J* = 2.5 Hz, 1H), 4.15 (d, *J* = 17.3 Hz, 1H), 3.78 (s, 3H), 3.65 (s, 3H), 2.21 (dddd, *J* = 14.7, 7.0, 5.5, 1.6 Hz, 1H), 2.08 (dddd, *J* = 14.7, 9.2, 7.8, 1.4 Hz, 1H), 1.45 (s, 9H); ^{13}C NMR (151 MHz, [D_6]-DMSO, 383K): δ = 165.1, 158.6, 154.3, 144.9, 133.6, 130.3, 126.7, 121.8, 112.5, 110.5, 78.4, 67.9, 55.6, 54.7, 50.2, 42.0, 32.8, 27.6; IR (ATR): ṽ = 3425, 2974, 1723, 1690, 1613, 1406, 1365, 1270, 1162, 1126, 1063 cm^{-1}; HRMS (ESI$^+$): *m/z*: calcd. for $C_{20}H_{27}NO_6Na$ $[M+Na]^+$: 400.17306, found: 400.17307.

The ee was determined by HPLC analysis: Chiralpak 150 mm IG-3, 3 µm, 4.6 mm ∅, acetonitrile/water = 45:55, v = 1.0 mL/min, λ = 220 nm, t(minor) = 11.01 min, t(major) = 16.37 min, (enantioenriched sample: left, racemic sample: right).

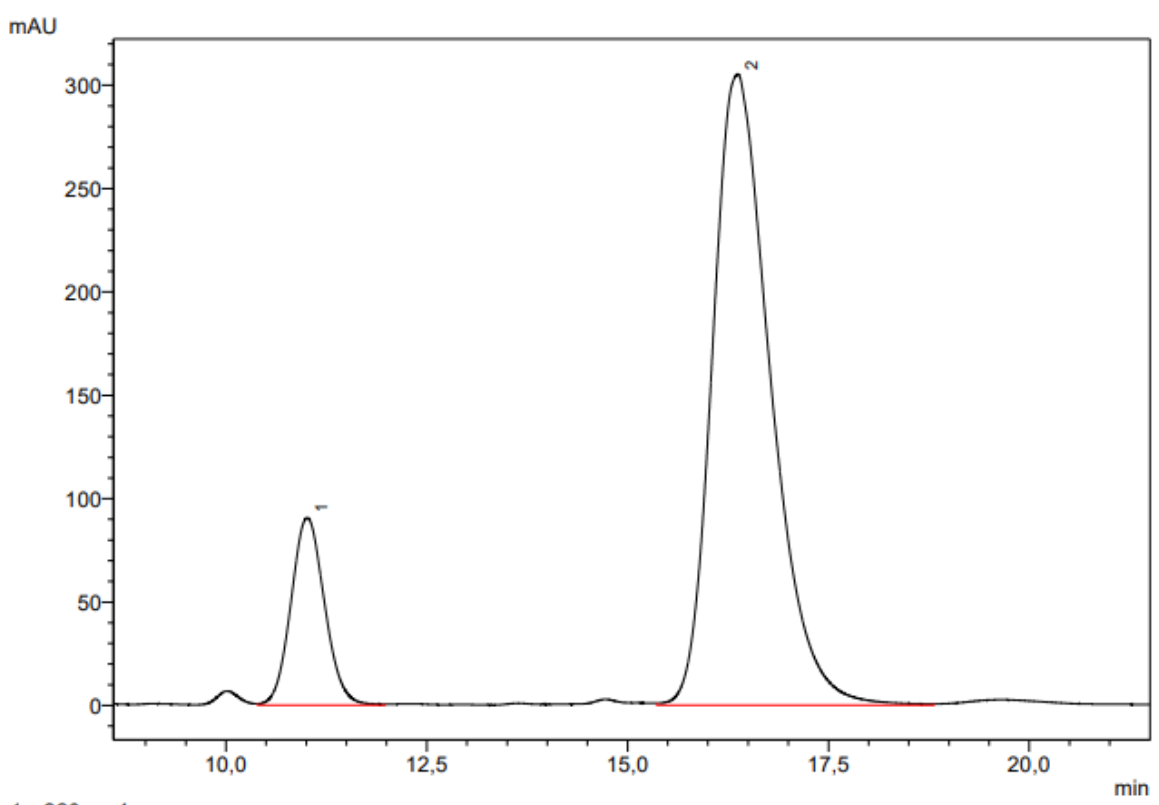

1 220nm,4nm

PDA Ch1 220nm

Peak #	Ret. Time	Area %	Name
1	11,01	14,67	1. Enantiomer
2	16,37	85,33	2. Enantiomer 70.7 % ee
Total		100,00	

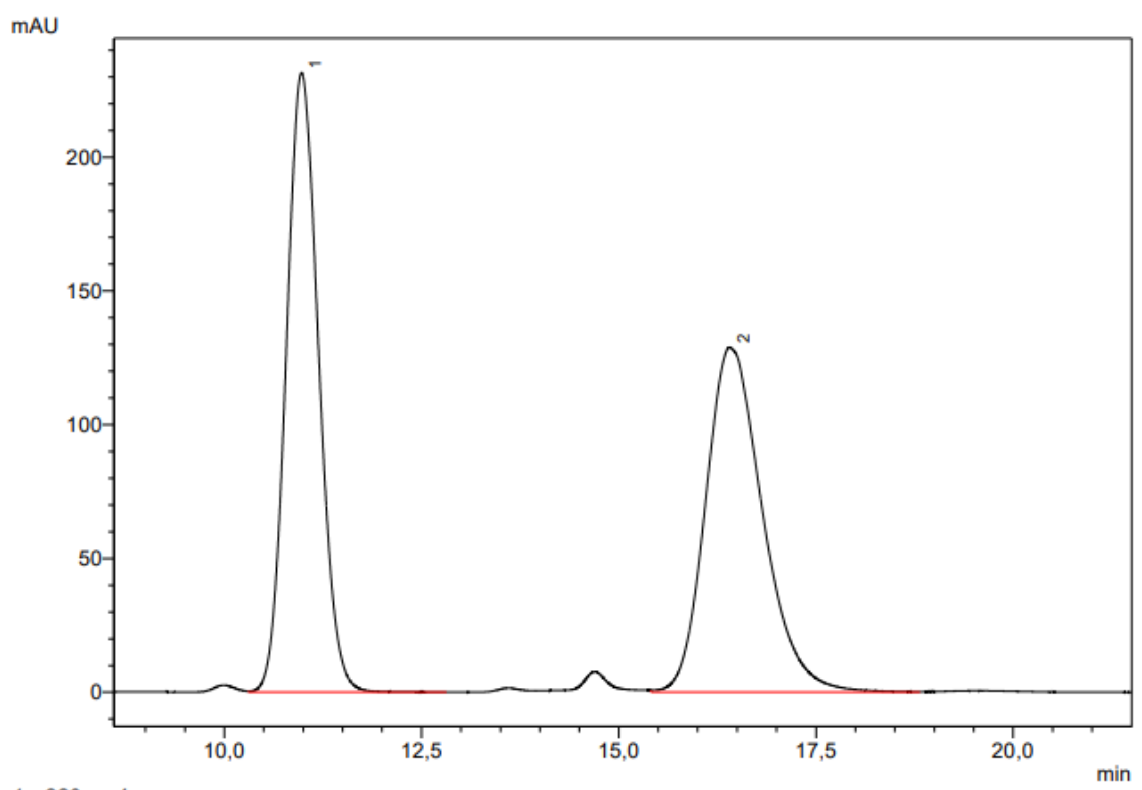

1 220nm,4nm

PDA Ch1 220nm

Peak #	Ret. Time	Area %	Name
1	10,98	50,53	1. Enantiomer
2	16,40	49,47	2. Enantiomer
Total		100,00	

***tert*-Butyl (3*R*,4*S*)-4-hydroxy-3-((*E*)-4-methoxy-4-oxobut-2-en-1-yl)-7-phenyl-3,4-dihydroiso-quinoline-2(1*H*)-carboxylate (8h).**

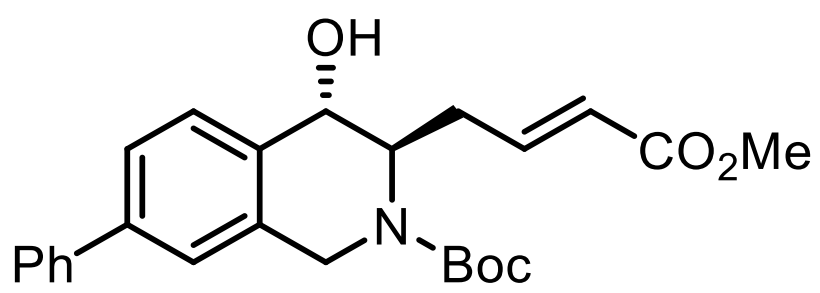

The title compound was prepared according to the general procedure **D** followed by oxidative workup **I**, using methyl (2*E*,4*E*)-5-((*tert*-butoxycarbonyl)((4-formyl-[1,1'-biphenyl]-3-yl)methyl)-amino)penta-2,4-dienoate **7h** (42.1 mg, 0.10 mmol); the reaction time was 16 h. The crude material was purified by flash chromatography (SiO_2, *n*-hexane/ethyl acetate, 1.5:1) to give the product as an off-white solid (38.0 mg, 90%, 91% ee).

$[\alpha]_D^{20}$ = +56.2 (c = 0.50, $CHCl_3$); ^{1}H NMR (600 MHz, [D$_6$]-DMSO, 383K): δ = 7.68 – 7.64 (m, 2H), 7.53 (ddt, *J* = 7.9, 1.6, 0.7 Hz, 1H), 7.50 – 7.49 (m, 1H), 7.46 (dddd, *J* = 7.8, 6.7, 1.4, 0.8 Hz, 1H), 7.40 (d, *J* = 7.8 Hz, 1H), 7.38 – 7.34 (m, 1H), 6.85 (ddd, *J* = 15.6, 7.8, 6.8 Hz, 1H), 5.83 (dt, *J* = 15.6, 1.5 Hz, 1H), 5.01 (d, *J* = 4.9 Hz, 1H), 4.86 (d, *J* = 17.3 Hz, 1H), 4.52 (d, *J* = 2.5 Hz, 1H), 4.50 (dq, *J* = 5.5, 2.7 Hz, 1H), 4.27 (d, *J* = 17.2 Hz, 1H), 3.65 (s, 3H), 2.28 (dddd, *J* = 14.7, 6.9, 5.5, 1.6 Hz, 1H), 2.15 (dddd, *J* = 14.7, 9.1, 7.9, 1.4 Hz, 1H), 1.47 (s, 9H); ^{13}C NMR (151 MHz, [D$_6$]-DMSO, 383K): δ = 165.2, 154.3, 144.9, 139.5, 139.3, 133.5, 132.9, 129.6, 128.2, 126.8, 126.1, 124.4, 123.7, 121.9, 78.5, 68.1, 55.5, 50.3, 42.0, 32.8, 27.6; IR (ATR): $\tilde{\nu}$ = 3428, 2925, 1723, 1691, 1407, 1366, 1270, 1168, 1040, 767 cm^{-1}; HRMS (ESI$^+$): *m/z*: calcd. for $C_{25}H_{29}NO_5Na$ [M+Na]$^+$: 446.19379, found: 446.19370.

The ee was determined by HPLC analysis: Chiralpak 150 mm IA-3, 3 µm, 4.6 mm ∅, *n*-heptane/ethanol = 90:10, v = 1.0 mL/min, λ = 254 nm, t(minor) = 11.35 min, t(major) = 14.03 min, (enantioenriched sample: left, racemic sample: right).

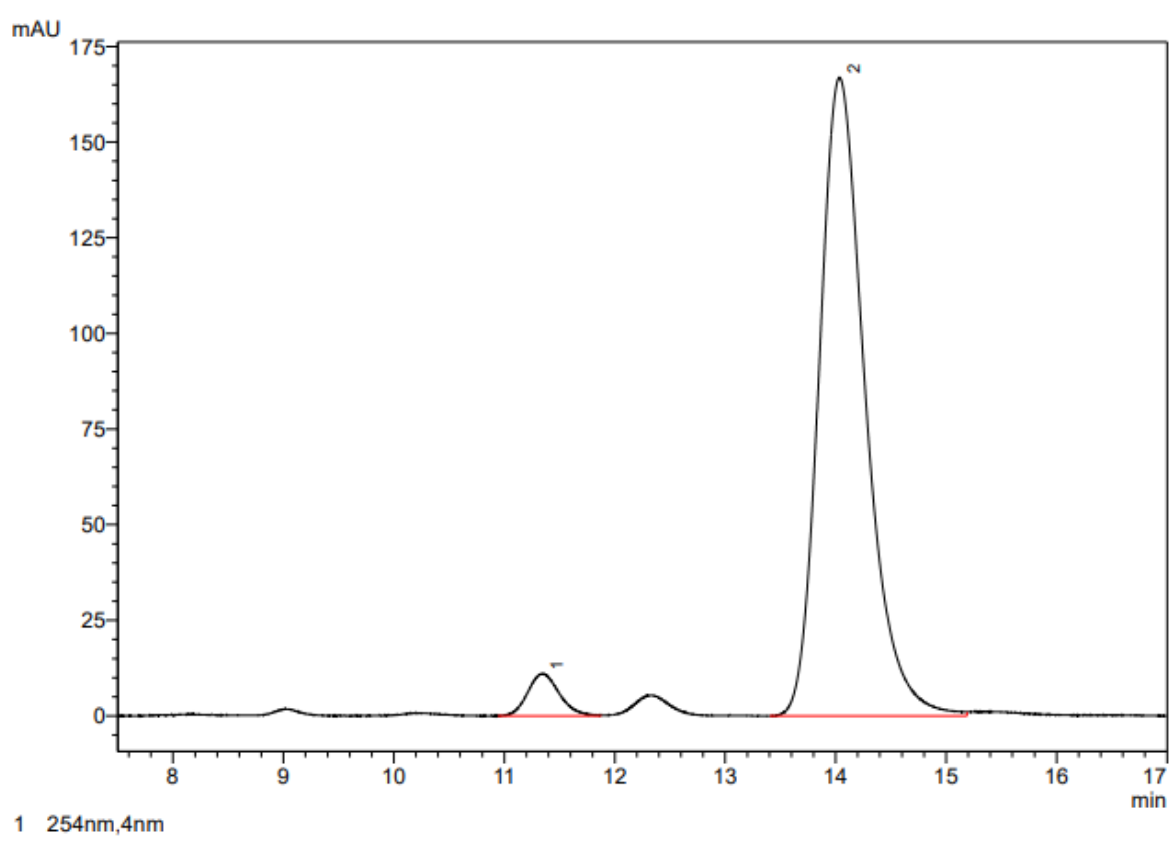

PDA Ch1 254nm

Peak #	Ret. Time	Area %	Name
1	11,35	4,36	1. Enantiomer
2	14,03	95,64	2. Enantiomer 91.3 % ee
Total		100,00	

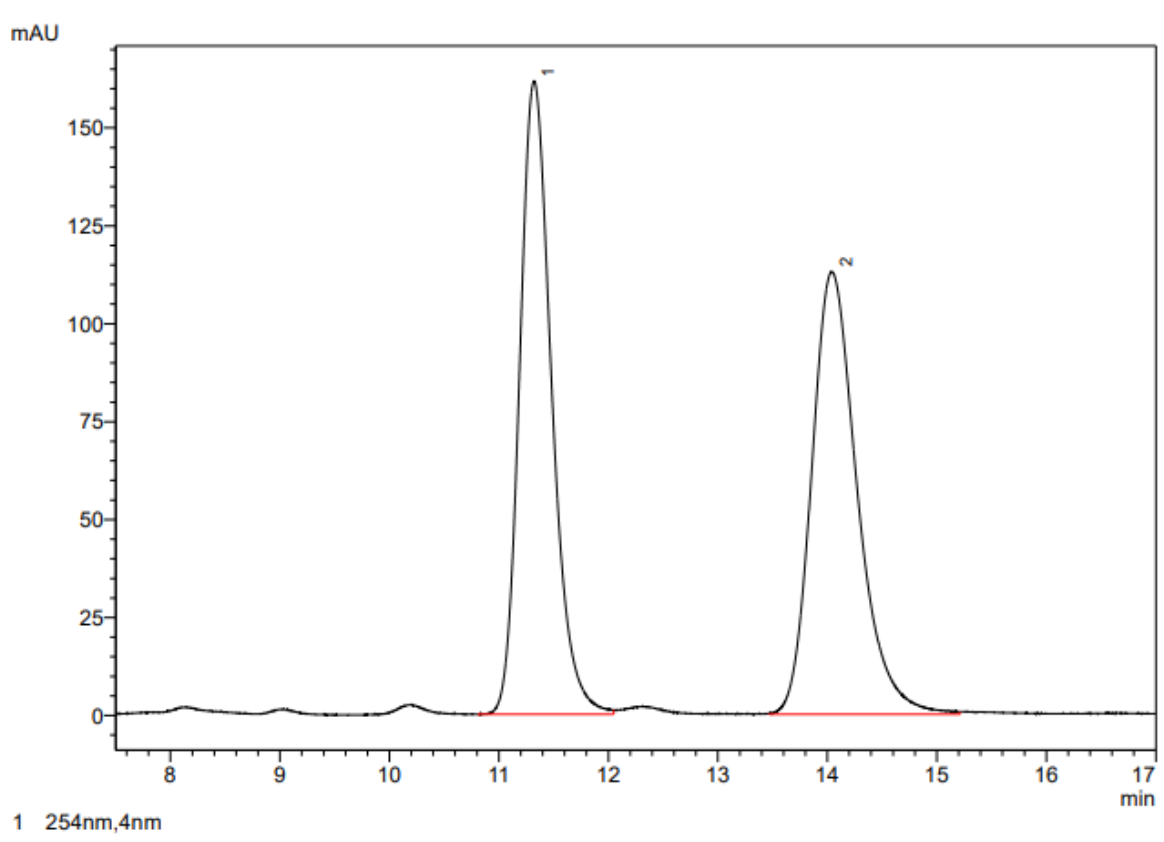

PDA Ch1 254nm

Peak #	Ret. Time	Area %	Name
1	11,32	50,06	1. Enantiomer
2	14,04	49,94	2. Enantiomer
Total		100,00	

tert-Butyl (3_R_,4_S_)-7-bromo-4-hydroxy-3-((_E_)-4-methoxy-4-oxobut-2-en-1-yl)-3,4-dihydro-isoquinoline-2(1_H_)-carboxylate (8i).

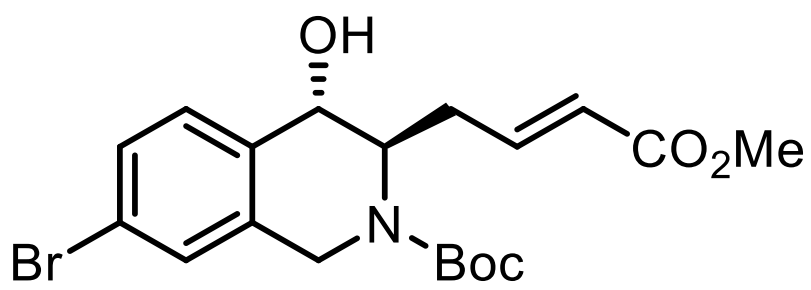

The title compound was prepared according to the general procedure **F** (the reaction was performed at 0 °C) followed by a basic workup **II**, using methyl (2*E*,4*E*)-5-((5-bromo-2-formylbenzyl)(*tert*-butoxycarbonyl)amino)penta-2,4-dienoate **7i** (42.4 mg, 0.10 mmol); the reaction time was 24 h. The crude material was purified by flash chromatography (SiO_2, *n*-pentane/ethyl acetate, 1:1) to give the product as a colorless solid (29.8 mg, 70%, 93% ee).

$[\alpha]_D^{20}$ = +75.3 (c = 0.45, $CHCl_3$); ^{1}H NMR (600 MHz, [D$_6$]-DMSO, 383K): δ = 7.44 – 7.43 (m, 1H), 7.43 – 7.39 (m, 1H), 7.27 (d, *J* = 8.1 Hz, 1H), 6.82 (ddd, *J* = 15.6, 7.9, 6.8 Hz, 1H), 5.82 (dt, *J* = 15.6, 1.5 Hz, 1H), 5.08 (d, *J* = 5.1 Hz, 1H), 4.78 (d, *J* = 17.3 Hz, 1H), 4.45 (d, *J* = 4.7 Hz, 1H), 4.44 (dd, *J* = 5.3, 2.7 Hz, 1H), 4.17 (d, *J* = 17.5 Hz, 1H), 3.65 (s, 3H), 2.25 (dddd, *J* = 14.7, 6.8, 5.3, 1.6 Hz, 1H), 2.09 (dddd, *J* = 14.7, 9.1, 7.9, 1.4 Hz, 1H), 1.45 (s, 9H); ^{13}C NMR (151 MHz, [D$_6$]-DMSO, 383K): δ = 165.1, 154.1, 144.7, 135.1, 133.8, 131.1, 128.9, 128.2, 122.0, 120.2, 78.7, 67.7, 55.3, 50.3, 41.3, 32.6, 27.5; IR (ATR): ṽ = 3425, 2978, 1723, 1693, 1405, 1366, 1321, 1271, 1164, 1041 cm^{-1}; HRMS (ESI$^+$): *m/z*: calcd. for $C_{19}H_{24}NO_5BrNa$ $[M+Na]^+$: 448.07301, found: 448.07288.

The ee was determined by HPLC analysis: Chiralpak 150 mm OJ-3R, 3 µm, 4.6 mm ∅, acetonitrile/water = 35:65, v = 1.0 mL/min, λ = 220 nm, t(major) = 23.90 min, t(minor) = 26.83 min, (enantioenriched sample: left, racemic sample: right).

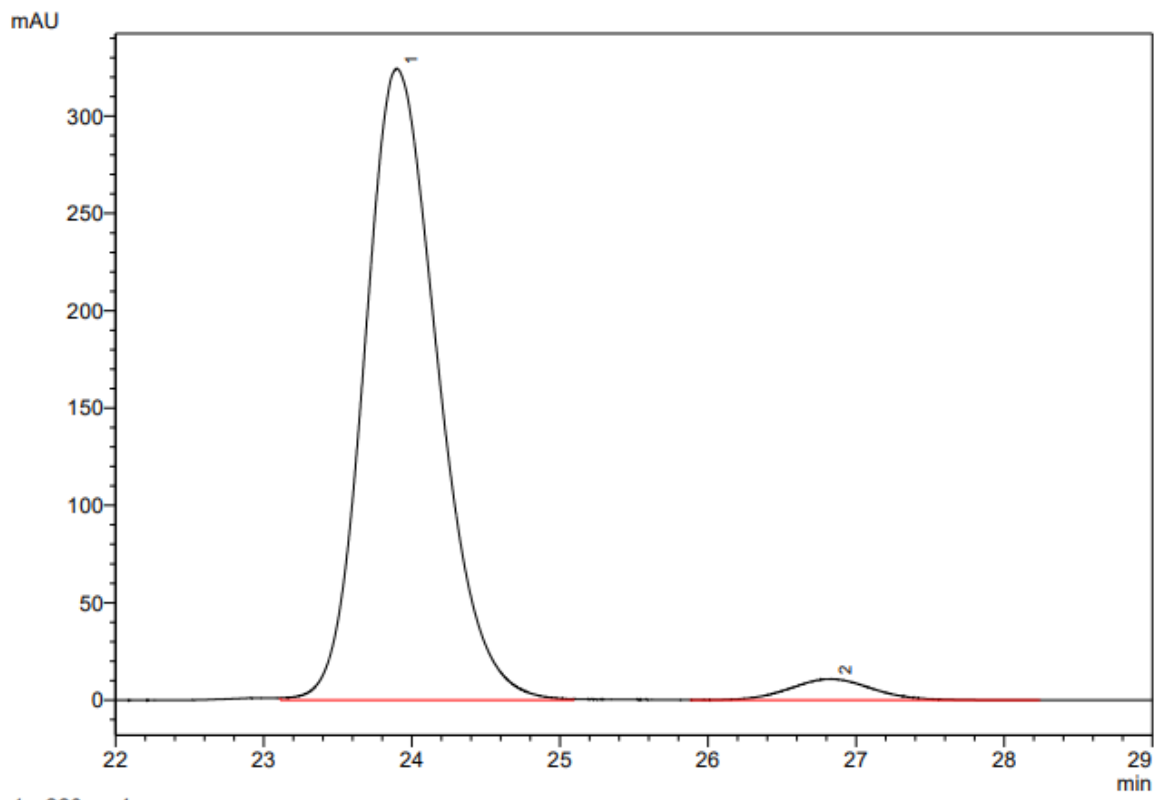

1 220nm,4nm

PDA Ch1 220nm

Peak #	Ret. Time	Area %	Name
1	23,90	96,34	1. Enantiomer 92.7 % ee
2	26,83	3,66	2. Enantiomer
Total		100,00	

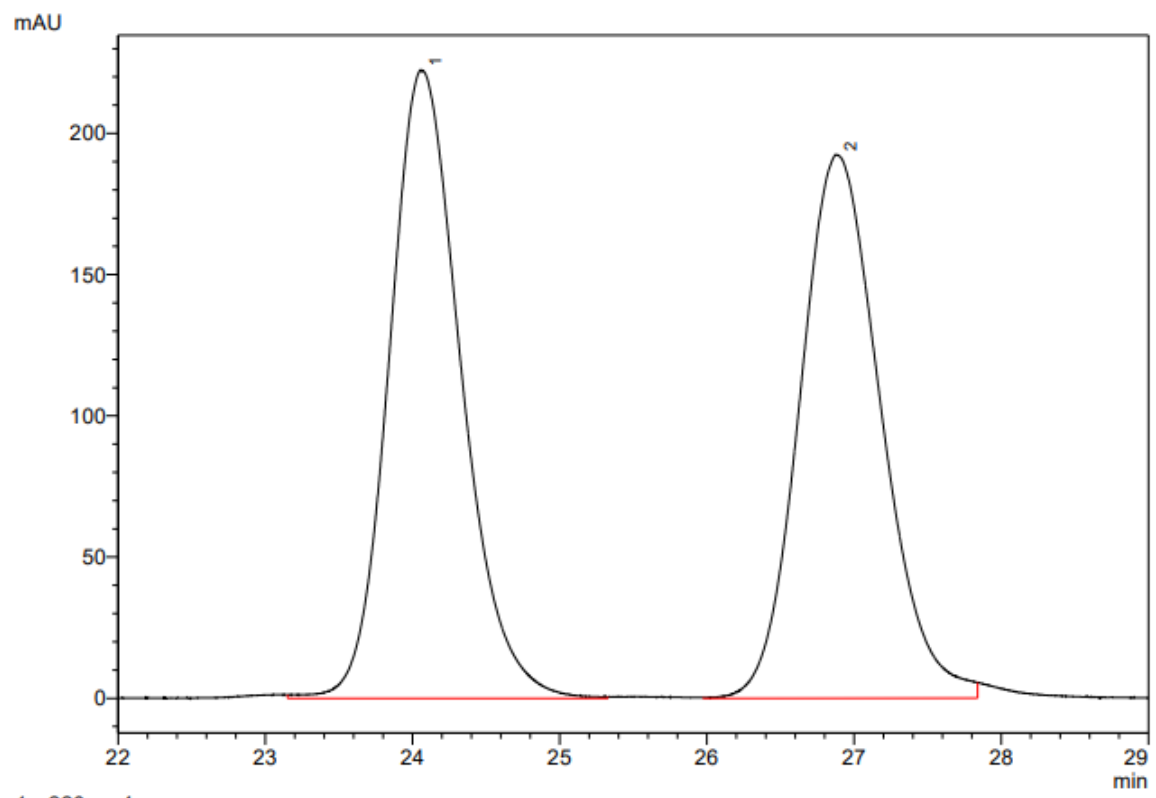

1 220nm,4nm

PDA Ch1 220nm

Peak #	Ret. Time	Area %	Name
1	24,06	50,70	1. Enantiomer
2	26,88	49,30	2. Enantiomer
Total		100,00	

tert-Butyl (3*R*,4*S*)-4-hydroxy-3-((*E*)-4-methoxy-4-oxobut-2-en-1-yl)-7-(phenylsulfonyl)-3,4-dihydro-isoquinoline-2(1*H*)-carboxylate (8j).

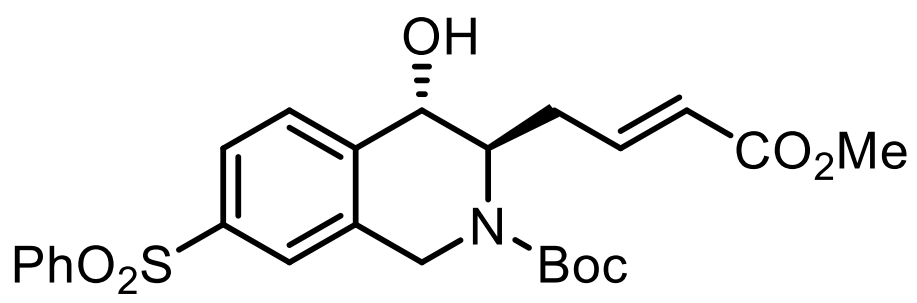

The title compound was prepared according to the general procedure **D** followed by oxidative workup **I**, using methyl (2*E*,4*E*)-5-((*tert*-butoxycarbonyl)(2-formyl-5-(phenylsulfonyl)-benzyl)amino)penta-2,4-dienoate **7j** (48.6 mg, 0.10 mmol); the reaction time was 16 h. The crude material was purified by flash chromatography (SiO_2, *n*-pentane/ethyl acetate, 1:1) to give the product as an off-white solid (41.3 mg, 85%, 97% ee).

Mp = 195 – 196 °C; $[\alpha]_D^{20}$ = +23.1 (c = 0.51, $CHCl_3$); ^{1}H NMR (600 MHz, [D_6]-DMSO, 383K): δ = 7.97 – 7.92 (m, 2H), 7.83 – 7.82 (m, 1H), 7.80 – 7.76 (m, 1H), 7.70 – 7.65 (m, 1H), 7.64 – 7.60 (m, 2H), 7.55 (dt, *J* = 8.0, 0.5 Hz, 1H), 6.81 (ddd, *J* = 15.6, 7.9, 6.8 Hz, 1H), 5.83 (dt, *J* = 15.6, 1.5 Hz, 1H), 5.26 (br, 1H), 4.86 (d, *J* = 17.5 Hz, 1H), 4.52 (d, *J* = 2.8 Hz, 1H), 4.44 (ddd, *J* = 9.1, 5.4, 2.8 Hz, 1H), 4.24 (d, *J* = 17.7 Hz, 1H), 3.64 (s, 3H), 2.26 (dddd, *J* = 14.7, 6.8, 5.4, 1.6 Hz, 1H), 2.11 (dddd, *J* = 14.7, 9.2, 7.9, 1.4 Hz, 1H), 1.44 (s, 9H); ^{13}C NMR (151 MHz, [D_6]-DMSO, 383K): δ = 165.1, 154.1, 144.5, 141.0, 140.2, 140.1, 134.3, 132.8, 130.2, 129.0, 126.7, 124.9, 124.6, 122.1, 78.8, 67.8, 55.2, 50.3, 41.6, 32.5, 27.5; IR (ATR): ṽ = 3459, 2977, 1722, 1693, 1406, 1324, 1154, 1092, 725 cm^{-1}; HRMS (ESI$^+$): *m/z*: calcd. for $C_{25}H_{29}NO_7SNa$ [M+Na]$^+$: 510.15569, found: 510.15598.

Note: Single crystals suitable for X-ray diffraction analysis were obtained in the process of recrystallization *via* vapor diffusion using *n*-pentane and ethyl acetate.

The ee was determined by HPLC analysis: Chiralpak 150 mm IB-N-3, 3 µm, 4.6 mm ∅, acetonitrile/water = 45:55, v = 1.0 mL/min, λ = 220 nm, t(minor) = 9.10 min, t(major) = 10.92 min, (enantioenriched sample: left, racemic sample: right).

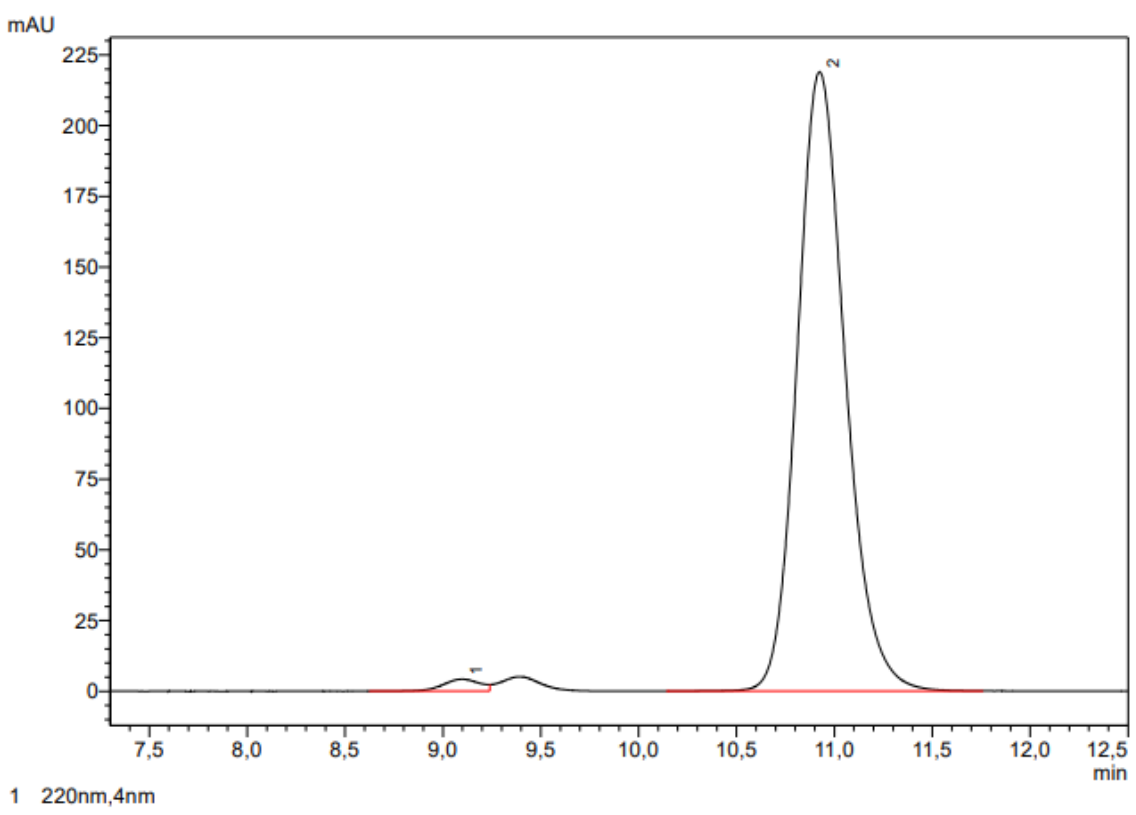

PDA Ch1 220nm

Peak #	Ret. Time	Area %	Name
1	9,10	1,50	1. Enantiomer
2	10,92	98,50	2. Enantiomer 97.0 % ee
Total		100,00	

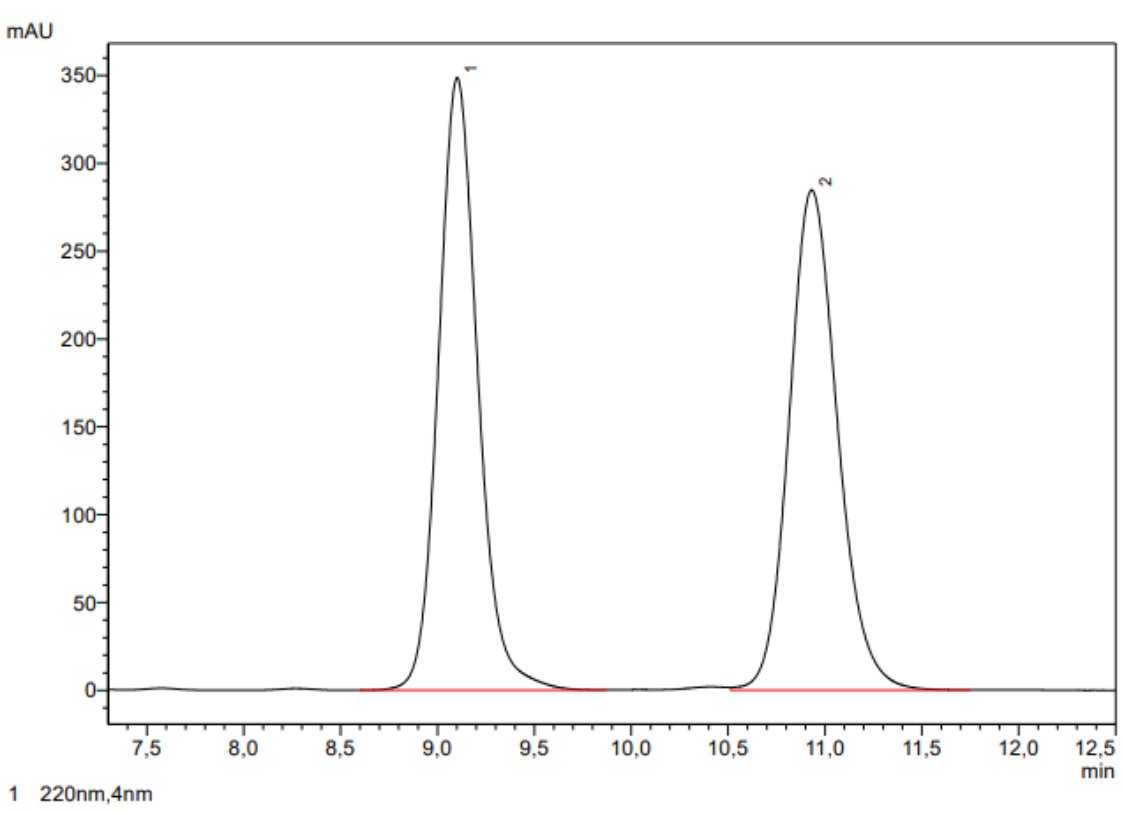

PDA Ch1 220nm

Peak #	Ret. Time	Area %	Name
1	9,10	49,86	1. Enantiomer
2	10,93	50,14	2. Enantiomer
Total		100,00	

tert-Butyl (3_R_,4_S_)-4-hydroxy-3-((_E_)-4-methoxy-4-oxobut-2-en-1-yl)-7-(4,4,5,5-tetramethyl-1,3,2-dioxaborolan-2-yl)-3,4-dihydroisoquinoline-2(1_H_)-carboxylate (8k).

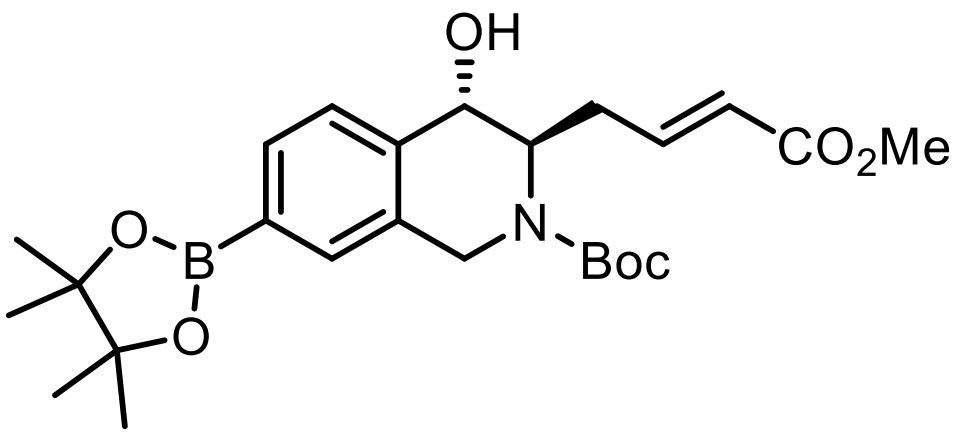

The title compound was prepared according to the general procedure **D** and basic workup **II**, using methyl (2_E_,4_E_)-5-((_tert_-butoxycarbonyl)(2-formyl-5-(4,4,5,5-tetramethyl-1,3,2-dioxaborolan-2-yl)benzyl)amino)penta-2,4-dienoate **7k** (47.1 mg, 0.10 mmol); the reaction time was 16 h. The crude material was purified by flash chromatography (SiO_2, _n_-pentane/ethyl acetate, 2:1) to give the product as a colorless oil (40.4 mg, 85%, 95% ee).

$[\alpha]_D^{20}$ = +48.7 (c = 0.46, $CHCl_3$); ^1H NMR (600 MHz, [D_6]-DMSO, 383K): δ = 7.56 (dd, _J_ = 7.4, 1.2 Hz, 1H), 7.51 (s, 1H), 7.32 (d, _J_ = 7.5 Hz, 1H), 6.82 (ddd, _J_ = 15.7, 7.8, 6.8 Hz, 1H), 5.81 (dt, _J_ = 15.7, 1.5 Hz, 1H), 5.03 (d, _J_ = 5.3 Hz, 1H), 4.78 (d, _J_ = 17.0 Hz, 1H), 4.48 (dd, _J_ = 5.3, 2.8 Hz, 1H), 4.45 (ddd, _J_ = 8.5, 5.5, 2.8 Hz, 1H), 4.19 (d, _J_ = 17.1 Hz, 1H), 3.65 (s, 3H), 2.24 (dddd, _J_ = 14.7, 6.9, 5.4, 1.6 Hz, 1H), 2.10 (dddd, _J_ = 14.7, 9.1, 7.8, 1.4 Hz, 1H), 1.45 (s, 9H), 1.33 (s, 12H); ^{13}C NMR (151 MHz, [D_6]-DMSO, 383K): δ = 165.1, 154.2, 144.8, 137.5, 131.8 (2 x), 131.5, 128.3, 121.9, 83.1, 78.5, 68.3, 55.4, 50.2, 41.7, 32.8, 27.5, 24.1; (the C-Bpin signal is broad and could only be assigned based on the 2D HMBC (~127.7 ppm)); IR (ATR): ṽ = 3436, 2977, 1723, 1692, 1393, 1360, 1270, 1164, 1144, 1105, 1040, 854 cm^{-1}; HRMS (ESI^+): _m/z_: calcd. for $C_{25}H_{36}BNO_7Na$ $[M+Na]^+$: 496.24770, found: 496.24760.

The ee was determined by HPLC analysis: Chiralpak 150 mm IA-3, 3 µm, 4.6 mm ∅, _n_-heptane/_iso_-propanol = 95:5, v = 1.0 mL/min, λ = 225 nm, t(major) = 11.37 min, t(minor) = 15.93 min, (enantioenriched sample: left, racemic sample: right).

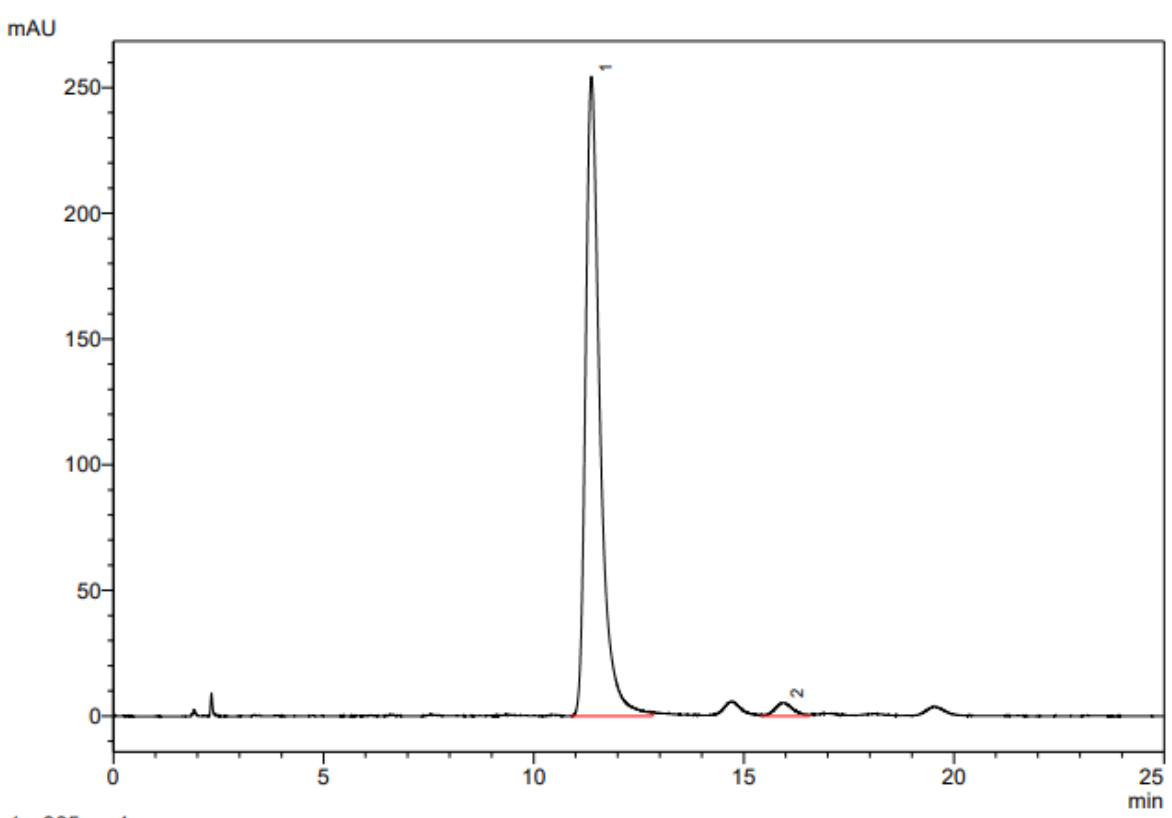

1 225nm,4nm

PDA Ch1 225nm

Peak #	Ret. Time	Area %	Name
1	11,37	97,38	1. Enantiomer 94.8 % ee
2	15,93	2,62	
Total		100,00	

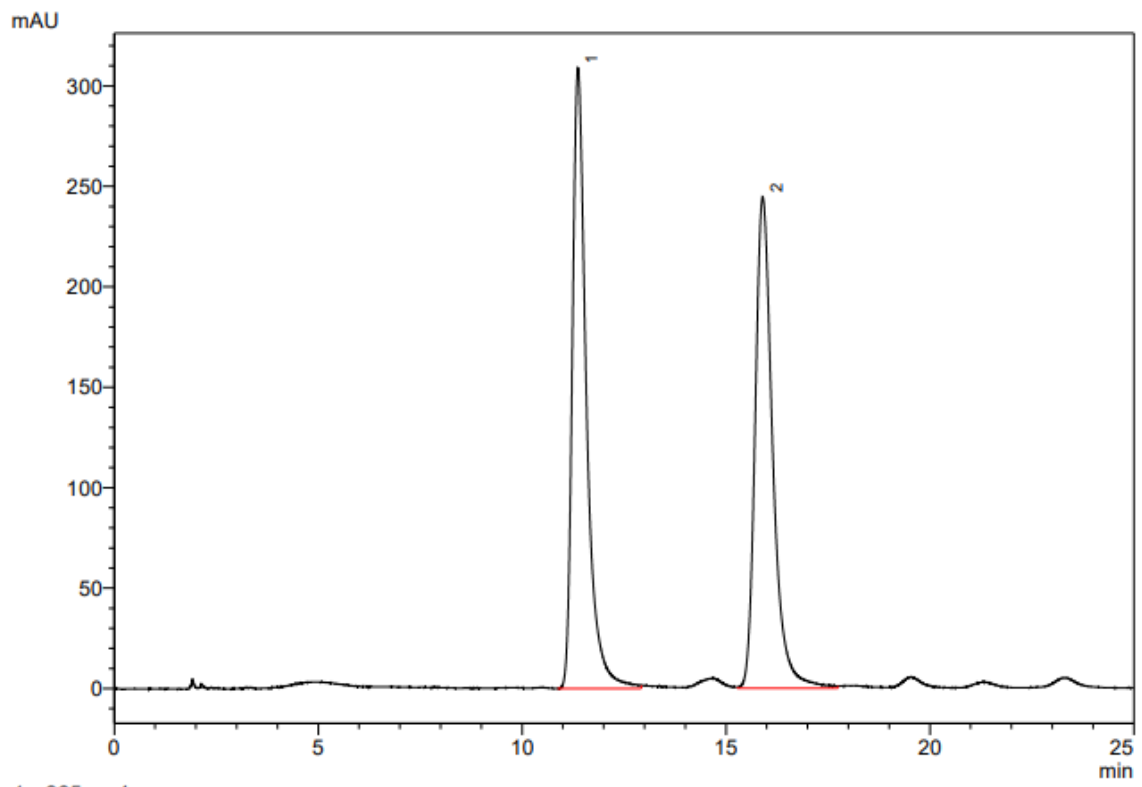

1 225nm,4nm

PDA Ch1 225nm

Peak #	Ret. Time	Area %	Name
1	11,37	50,38	1. Enantiomer
2	15,90	49,62	2. Enantiomer
Total		100,00	

tert-Butyl (3*R*,4*S*)-4,7-dihydroxy-3-((*E*)-4-methoxy-4-oxobut-2-en-1-yl)-3,4-dihydroisoquinoline-2(1*H*)-carboxylate (8l).

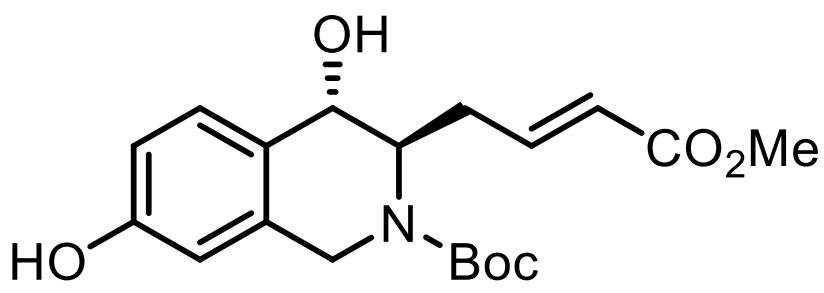

The title compound was prepared according to the general procedure **D** followed by basic workup **I**, using methyl (2*E*,4*E*)-5-((*tert*-butoxycarbonyl)(2-formyl-5-(4,4,5,5-tetramethyl-1,3,2-dioxaborolan-2-yl)benzyl)amino)penta-2,4-dienoate **7k** (47.1 mg, 0.10 mmol); the reaction time was 16 h.

The crude material was then dissolved in THF (1.0 mL). pH 7 Phosphate buffer solution (1.0 mL) and aq. H_2O_2 (30% *w/w*, ≈0.1 mL) were added and the resulting mixture was stirred at room temperature for 18 h. Additional ethyl acetate (10 mL) and water (5 mL) were introduced, the organic phase was separated, and the aqueous layer was extracted with ethyl acetate (2 × 10 mL). The combined organic phases were washed with sat. aq. $Na_2S_2O_3$ (10 mL) and brine (10 mL), dried over $MgSO_4$, filtered and concentrated *in vacuo*. The residue was purified by flash chromatography (SiO_2, toluene/*tert*-butyl methyl ether, 1:2) to give the product as a colorless solid (30.8 mg, 85%, 95% ee).

$[\alpha]_D^{20}$ = +63.8 (c = 0.48, $CHCl_3$); ^1H NMR (600 MHz, [D_6]-DMSO, 383K): δ = 8.96 (s, 1H), 7.09 (d, *J* = 8.2 Hz, 1H), 6.82 (ddd, *J* = 15.6, 7.8, 6.9 Hz, 1H), 6.66 (ddd, *J* = 8.2, 2.0, 1.2 Hz, 1H), 6.61 – 6.54 (m, 1H), 5.80 (dt, *J* = 15.6, 1.5 Hz, 1H), 4.67 (d, *J* = 17.1 Hz, 1H), 4.43 (ddd, *J* = 8.5, 5.5, 2.5 Hz, 1H), 4.37 (d, *J* = 2.5 Hz, 1H), 4.09 (d, *J* = 17.2 Hz, 1H), 3.65 (s, 3H), 2.20 (dddd, *J* = 14.7, 7.0, 5.5, 1.6 Hz, 1H), 2.08 (dddd, *J* = 14.7, 9.1, 7.8, 1.4 Hz, 1H), 1.45 (s, 9H); ^{13}C NMR (151 MHz, [D_6]-DMSO, 383K): δ = 165.2, 156.5, 154.3, 145.0, 133.3, 130.2, 125.0, 121.8, 113.6, 111.7, 78.4, 67.9, 55.7, 50.2, 41.9, 32.8, 27.6; IR (ATR): $\tilde{\nu}$ = 3340, 2977, 1695, 1660, 1410, 1367, 1291, 1163, 1126, 1039, 861 cm^{-1}; HRMS (ESI^+): *m/z*: calcd. for $C_{19}H_{25}NO_6Na$ $[M+Na]^+$: 386.15741, found: 386.15755.

The ee was determined by HPLC analysis: Chiralpak 150 mm IB-N-3, 3 µm, 4.6 mm ∅, *n*-heptane/ethanol = 95:5, v = 1.0 mL/min, λ = 220 nm, t(minor) = 16.13 min, t(major) = 25.65 min, (enantioenriched sample: left, racemic sample: right).

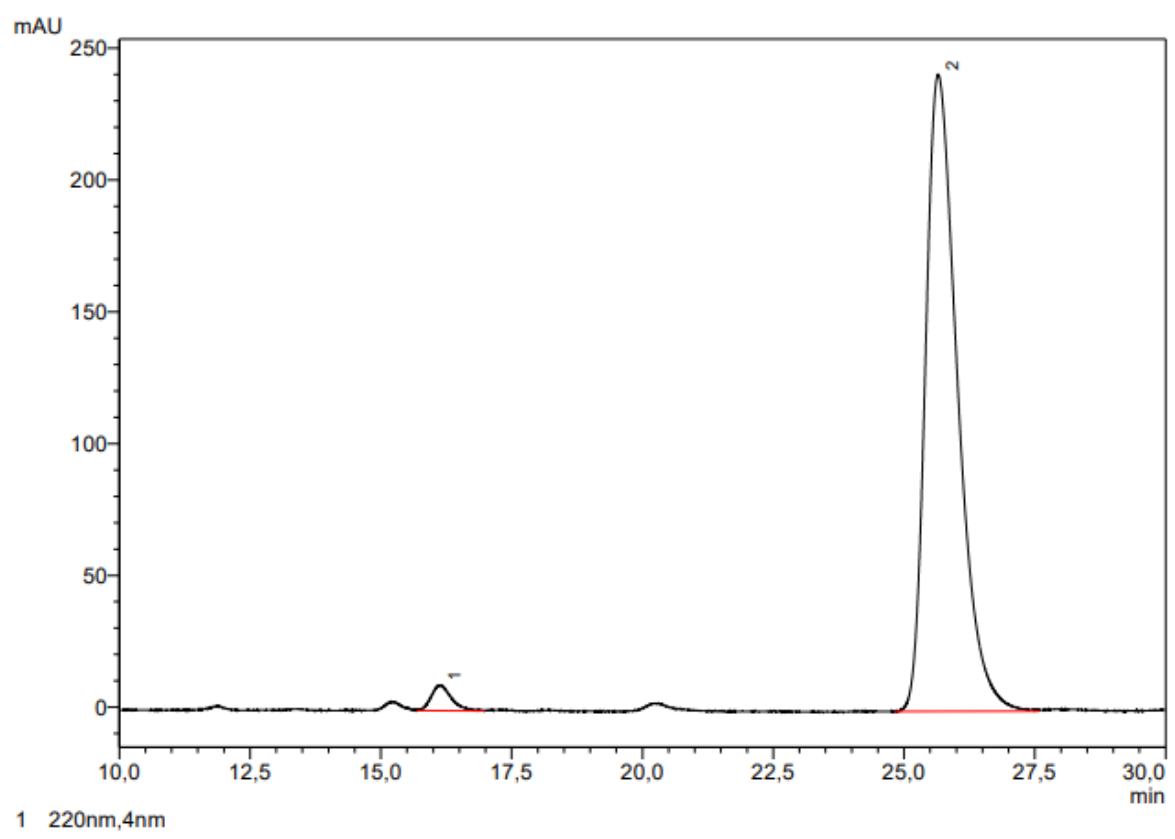

PDA Ch1 220nm

Peak #	Ret. Time	Area %	Name
1	16,13	2,48	1. Enantiomer
2	25,65	97,52	2. Enantiomer 95.0 % ee
Total		100,00	

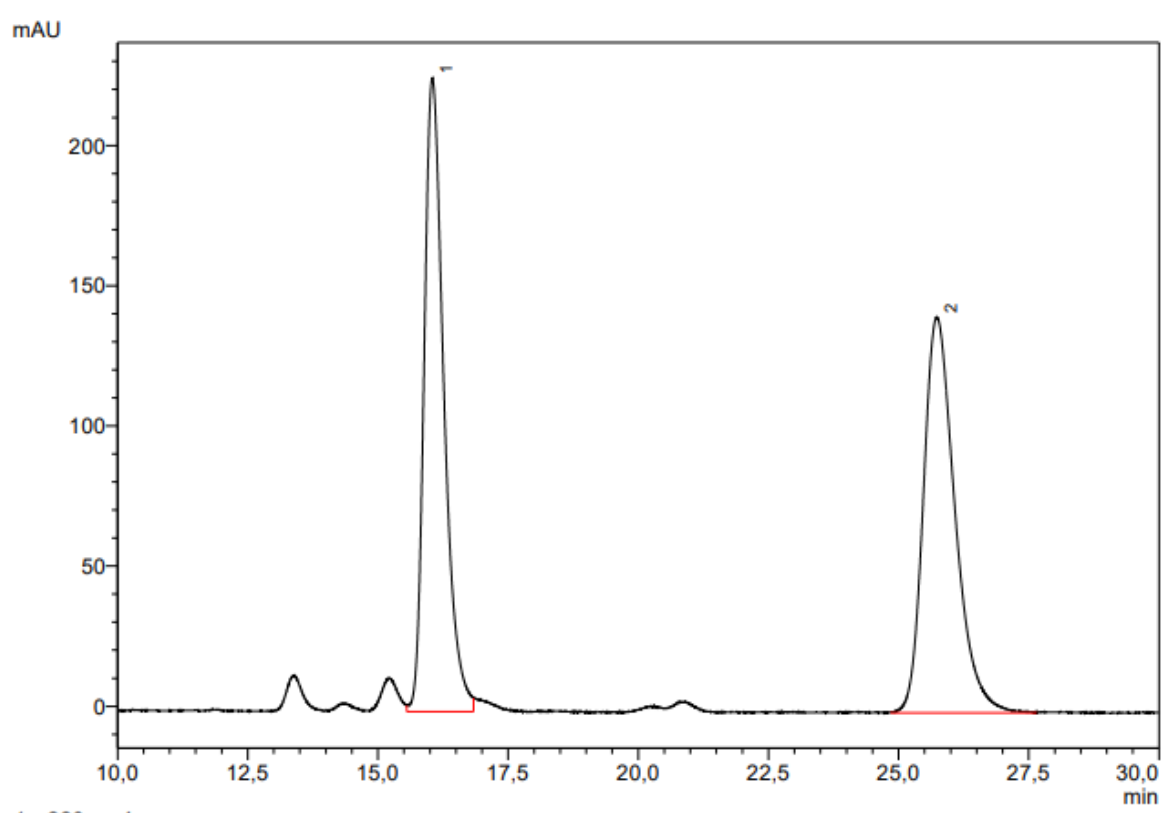

PDA Ch1 220nm

Peak #	Ret. Time	Area %	Name
1	16,05	50,10	1. Enantiomer
2	25,73	49,90	2. Enantiomer
Total		100,00	

***tert*-Butyl (3*R*,4*S*)-6-chloro-4-hydroxy-3-((*E*)-4-methoxy-4-oxobut-2-en-1-yl)-3,4-dihydro-isoquinoline-2(1*H*)-carboxylate (8m).**

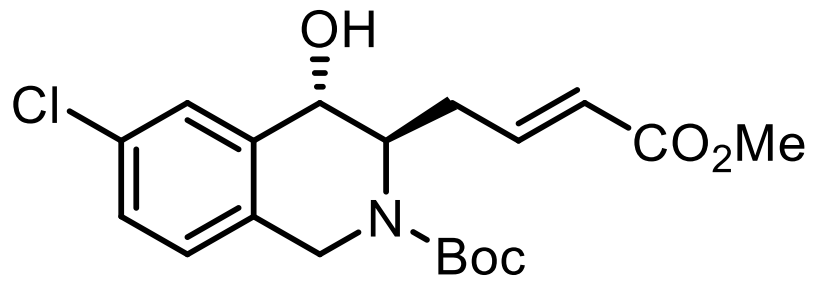

The title compound was prepared according to the general procedure **E** followed by oxidative workup **I**, using methyl (2*E*,4*E*)-5-((*tert*-butoxycarbonyl)(4-chloro-2-formylbenzyl)amino)penta-2,4-dienoate **7m** (38.0 mg, 0.10 mmol); the reaction time was 20 h. The crude material was purified by flash chromatography (SiO_2, *n*-pentane/ethyl acetate, 2:1) to give the product as a colorless oil (30.7 mg, 80%, 98% ee).

$[\alpha]_D^{20}$ = +50.2 (c = 0.56, $CHCl_3$); ^1H NMR (600 MHz, [D$_6$]-DMSO, 383K): δ = 7.38 – 7.35 (m, 1H), 7.31 (dd, *J* = 8.2, 2.3 Hz, 1H), 7.24 (d, *J* = 8.2 Hz, 1H), 6.83 (ddd, *J* = 15.6, 7.9, 6.8 Hz, 1H), 5.83 (dt, *J* = 15.6, 1.5 Hz, 1H), 5.15 (d, *J* = 5.1 Hz, 1H), 4.75 (d, *J* = 17.3 Hz, 1H), 4.47 (br, 1H), 4.43 (ddd, *J* = 8.5, 5.4, 2.8 Hz, 1H), 4.15 (d, *J* = 17.4 Hz, 1H), 3.65 (s, 3H), 2.27 (dddd, *J* = 14.7, 6.9, 5.4, 1.6 Hz, 1H), 2.11 (dddd, *J* = 14.7, 9.1, 7.9, 1.4 Hz, 1H), 1.45 (s, 9H); ^{13}C NMR (151 MHz, [D$_6$]-DMSO, 383K): δ = 165.1, 154.2, 144.7, 136.7, 131.4, 130.5, 128.5, 127.4, 127.0, 122.0, 78.7, 67.9, 55.2, 50.2, 41.4, 32.7, 27.5; IR (ATR): ṽ = 3414, 2977, 1722, 1691, 1408, 1366, 1249, 1164, 1040, 881 cm^{-1}; HRMS (ESI$^+$): *m/z*: calcd. for $C_{19}H_{24}NO_5ClNa$ [M+Na]$^+$: 404.12352, found: 404.12358.

The ee was determined by HPLC analysis: Chiralpak 150 mm IG-3, 3 µm, 4.6 mm ∅, acetonitrile/water = 45:55, v = 1.0 mL/min, λ = 220 nm, t(minor) = 17.46 min, t(major) = 29.16 min, (enantioenriched sample: left, racemic sample: right).

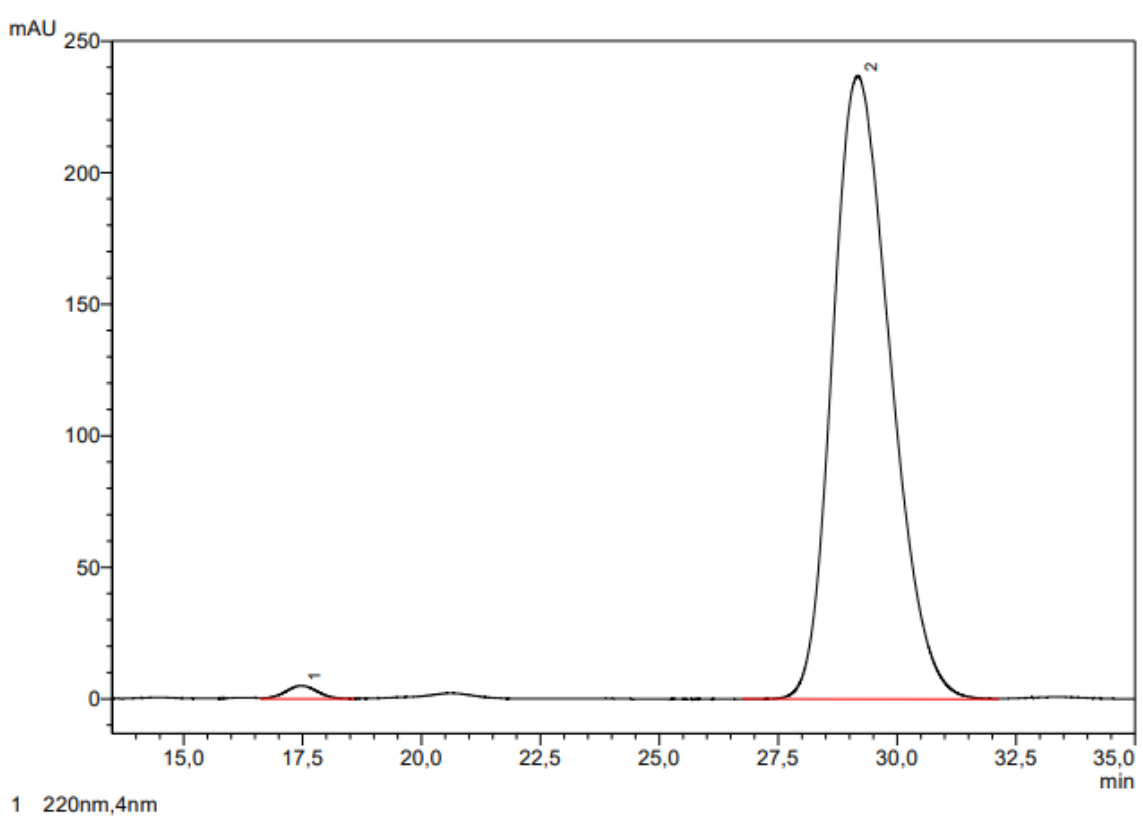

PDA Ch1 220nm

Peak #	Ret. Time	Area %	Name
1	17,46	1,20	1. Enantiomer
2	29,16	98,80	2. Enantiomer 97.6 % ee
Total		100,00	

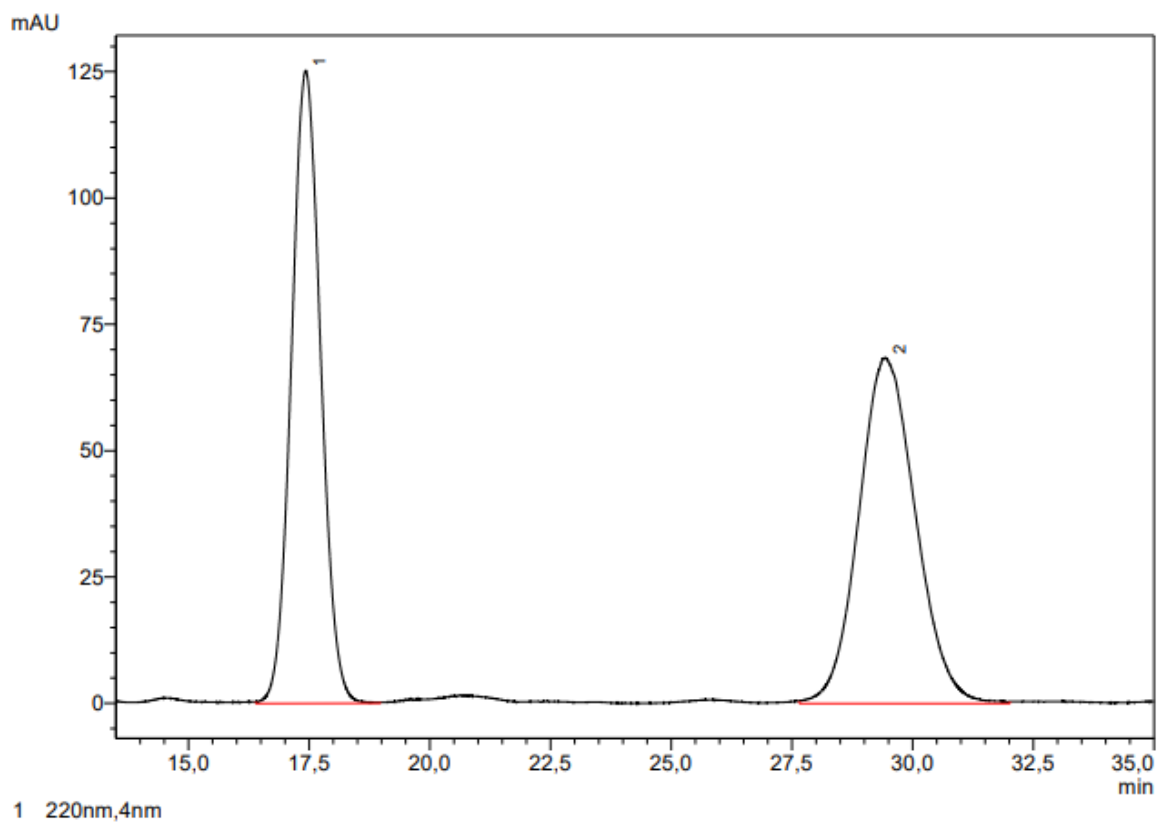

PDA Ch1 220nm

Peak #	Ret. Time	Area %	Name
1	17,43	48,57	1. Enantiomer
2	29,44	51,43	2. Enantiomer
Total		100,00	

tert-Butyl (3_R_,4_S_)-4-hydroxy-3-((_E_)-4-methoxy-4-oxobut-2-en-1-yl)-6-(trifluoromethoxy)-3,4-dihydroisoquinoline-2(1_H_)-carboxylate (8n).

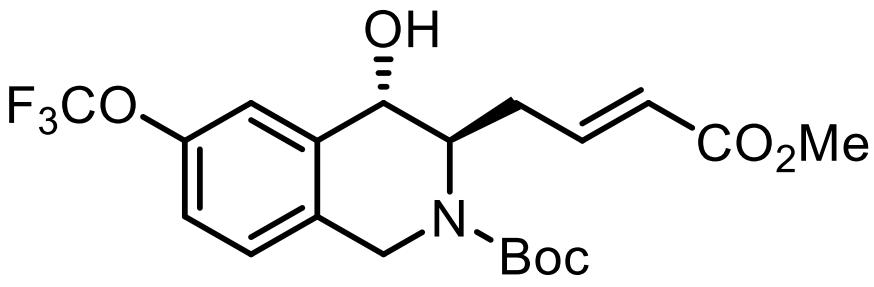

The title compound was prepared according to the general procedure **D** followed by oxidative workup **I**, using methyl methyl (2*E*,4*E*)-5-((*tert*-butoxycarbonyl)(2-formyl-4-(trifluoromethoxy)benzyl)amino)penta-2,4-dienoate **7n** (42.9 mg, 0.10 mmol); the reaction time was 16 h. The crude material was purified by flash chromatography (SiO_2, *n*-pentane/ethyl acetate, 2.5:1) to give the product as a colorless oil (34.3 mg, 80%, 97% ee).

$[\alpha]_D^{20}$ = +53.7 (c = 0.65, $CHCl_3$); ^1H NMR (600 MHz, [D$_6$]-DMSO, 383K): δ = 7.35 (ddt, *J* = 8.6, 1.2, 0.6 Hz, 1H), 7.27 (dq, *J* = 1.6, 0.5 Hz, 1H), 7.23 (ddt, *J* = 8.3, 2.6, 1.0 Hz, 1H), 6.83 (ddd, *J* = 15.6, 7.9, 6.8 Hz, 1H), 5.83 (dt, *J* = 15.7, 1.5 Hz, 1H), 5.23 (d, *J* = 5.4 Hz, 1H), 4.79 (d, *J* = 17.4 Hz, 1H), 4.51 (dd, *J* = 5.3, 2.8 Hz, 1H), 4.44 (ddd, *J* = 9.0, 5.4, 2.8 Hz, 1H), 4.19 (d, *J* = 17.1 Hz, 1H), 3.65 (s, 3H), 2.29 (dddd, *J* = 14.7, 6.9, 5.4, 1.6 Hz, 1H), 2.13 (dddd, *J* = 14.7, 9.2, 7.9, 1.4 Hz, 1H), 1.45 (s, 9H); ^{13}C NMR (151 MHz, [D$_6$]-DMSO, 383K): δ = 165.1, 154.1, 146.6 (q, *J* = 1.9 Hz), 144.6, 136.9, 131.8, 127.4, 122.0, 120.6, 119.6 (q, *J* = 256.5 Hz), 119.4, 78.7, 67.9, 55.2, 50.2, 41.3, 32.7, 27.5; ^{19}F NMR (585 MHz, [D$_6$]-DMSO, 383K): δ = –56.6; IR (ATR): ṽ = 3427, 2978, 1724, 1692, 1410, 1257, 1211, 1161, 1042 cm^{-1}; HRMS (ESI$^+$): *m/z*: calcd. for $C_{20}H_{24}NO_6F_3Na$ $[M+Na]^+$: 454.14479, found: 454.14452.

The ee was determined by HPLC analysis: Chiralpak 150 mm IG-3, 3 µm, 4.6 mm ∅, acetonitrile/water = 45:55, v = 1.0 mL/min, λ = 220 nm, t(minor) = 11.84 min, t(major) = 16.64 min, (enantioenriched sample: left, racemic sample: right).

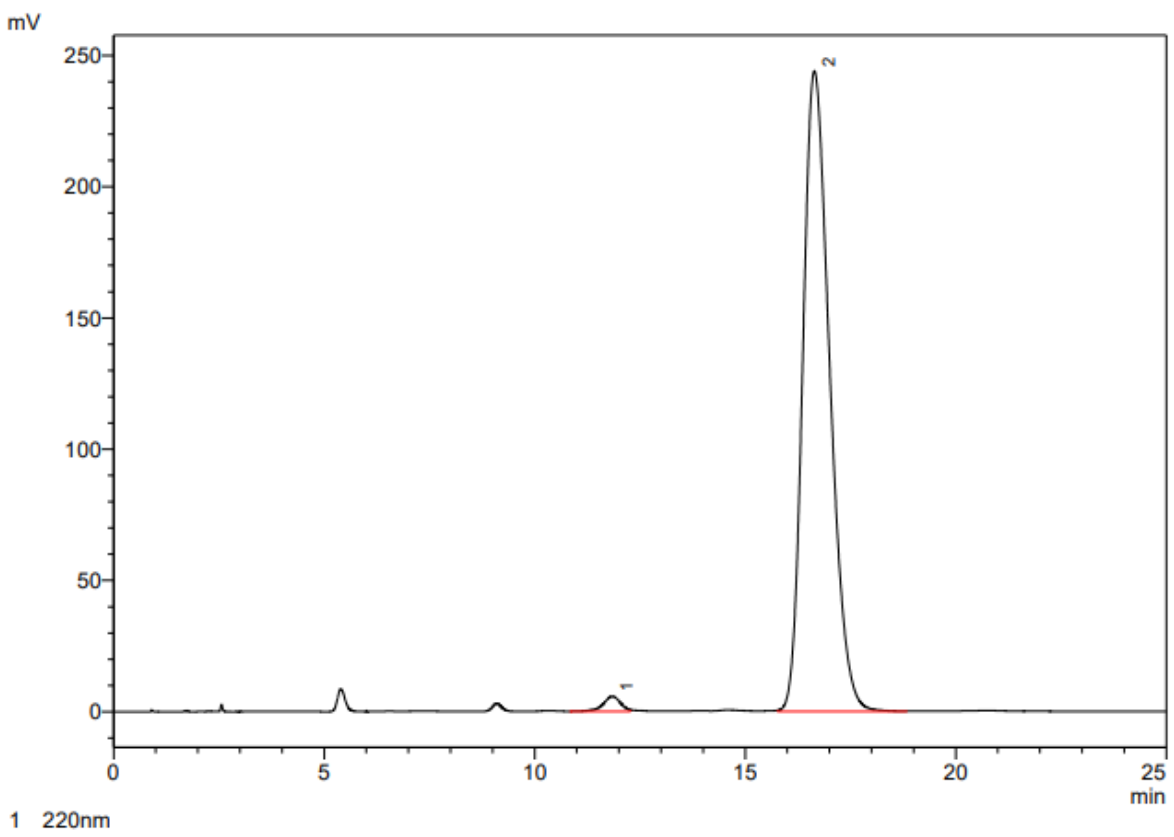

Detector A 220nm

Peak #	Ret. Time	Area %	Name
1	11,84	1,52	1. Enantiomer
2	16,64	98,48	2. Enantiomer 97.0 % ee
Total		100,00	

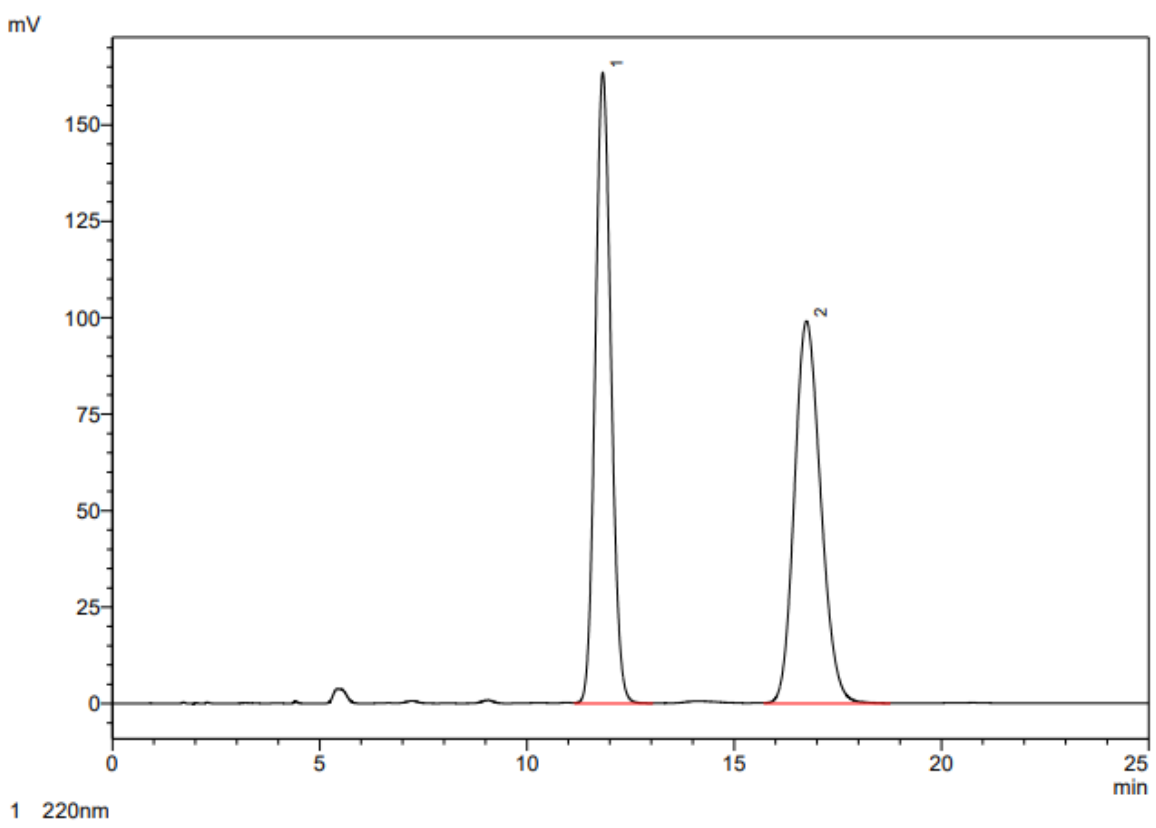

Detector A 220nm

Peak #	Ret. Time	Area %	Name
1	11,83	49,74	1. Enantiomer
2	16,75	50,26	2. Enantiomer
Total		100,00	

tert-Butyl (6_R_,7_S_)-7-hydroxy-6-((_E_)-4-methoxy-4-oxobut-2-en-1-yl)-6,7-dihydrothieno[3,4-c]-pyridine-5(4_H_)-carboxylate (9).

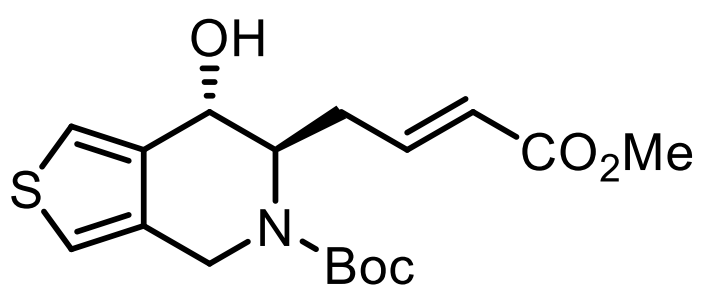

The title compound was prepared according to the general procedure **F** and basic workup **I**, using methyl (2_E_,4_E_)-5-((_tert_-butoxycarbonyl)((4-formylthiophen-3-yl)methyl)amino)penta-2,4-dienoate **S28** (35.1 mg, 0.10 mmol); the reaction time was 16 h. The crude material was purified by flash chromatography (SiO_2, _n_-pentane/ethyl acetate, 1:1) to give the product as a colorless oil (23.7 mg, 67%, 70% ee).

$[\alpha]_D^{20}$ = +36.4 (c = 0.45, $CHCl_3$); ^{1}H NMR (600 MHz, [D$_6$]-DMSO, 383K): δ = 7.42 (d, _J_ = 3.0 Hz, 1H), 7.25 – 7.18 (m, 1H), 6.81 (ddd, _J_ = 15.6, 7.8, 6.8 Hz, 1H), 5.82 (dt, _J_ = 15.7, 1.5 Hz, 1H), 4.86 (br, 1H), 4.80 (dd, _J_ = 16.5, 1.4 Hz, 1H), 4.58 (d, _J_ = 2.5 Hz, 1H), 4.50 (ddd, _J_ = 9.2, 5.5, 2.5 Hz, 1H), 4.16 (d, _J_ = 17.1 Hz, 1H), 3.65 (s, 3H), 2.26 – 2.16 (m, 1H), 2.07 (dddd, _J_ = 14.8, 9.2, 7.8, 1.4 Hz, 1H), 1.44 (s, 9H); ^{13}C NMR (151 MHz, [D$_6$]-DMSO, 383K): δ = 165.1, 154.4, 144.8, 136.8, 132.7, 123.4, 121.9, 118.2, 78.5, 65.1, 55.9, 50.3, 39.9, 32.4, 27.5; IR (ATR): ṽ = 3427, 2976, 1721, 1685, 1395, 1366, 1236, 1163, 1094, 1039, 731 cm^{-1}; HRMS (ESI$^+$): _m/z_: calcd. for $C_{17}H_{23}NO_5SNa$ [M+Na]$^+$: 376.11891, found: 376.11902.

The ee was determined by HPLC analysis: Chiralpak 150 mm IG-3, 3 µm, 4.6 mm ∅, acetonitrile/water = 50:50, v = 1.0 mL/min, λ = 220 nm, t(minor) = 6.43 min, t(major) = 14.45 min, (enantioenriched sample: left, racemic sample: right).

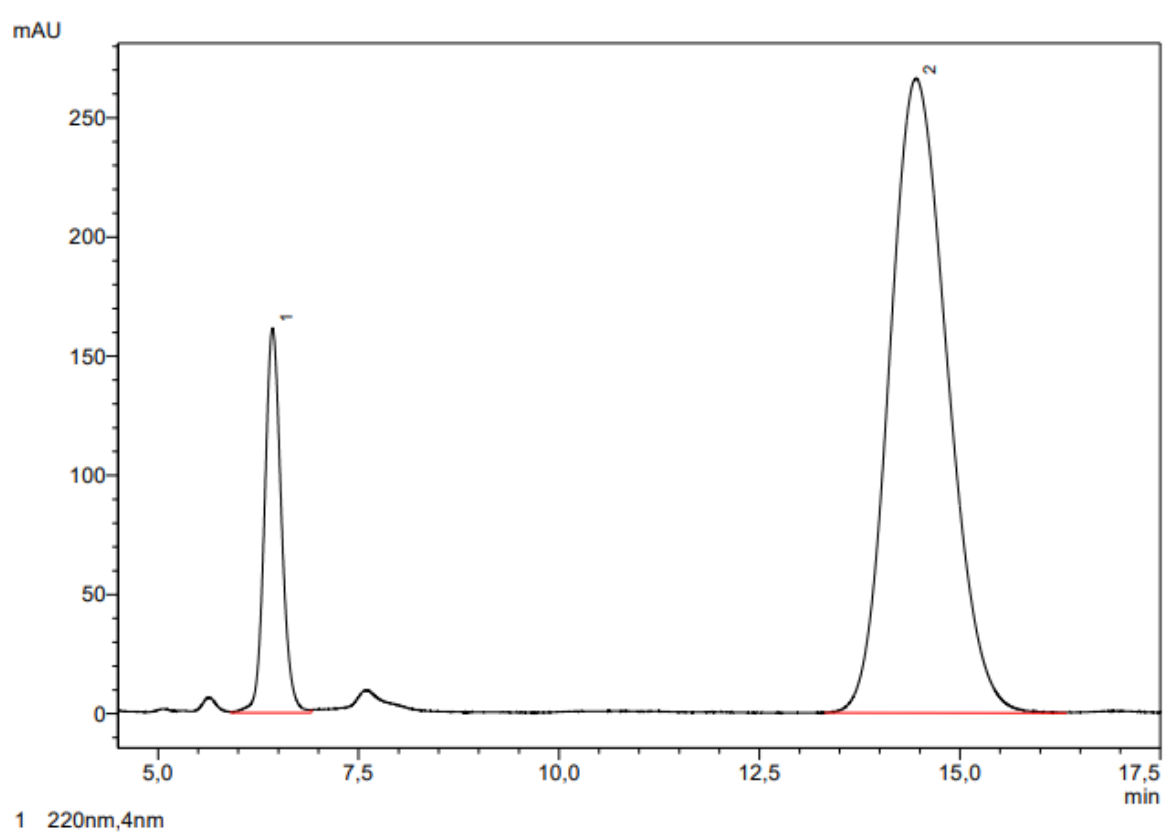

PDA Ch1 220nm

Peak #	Ret. Time	Area %	Name
1	6,43	14,94	1. Enantiomer
2	14,45	85,06	2. Enantiomer 70.1 % ee
Total		100,00	

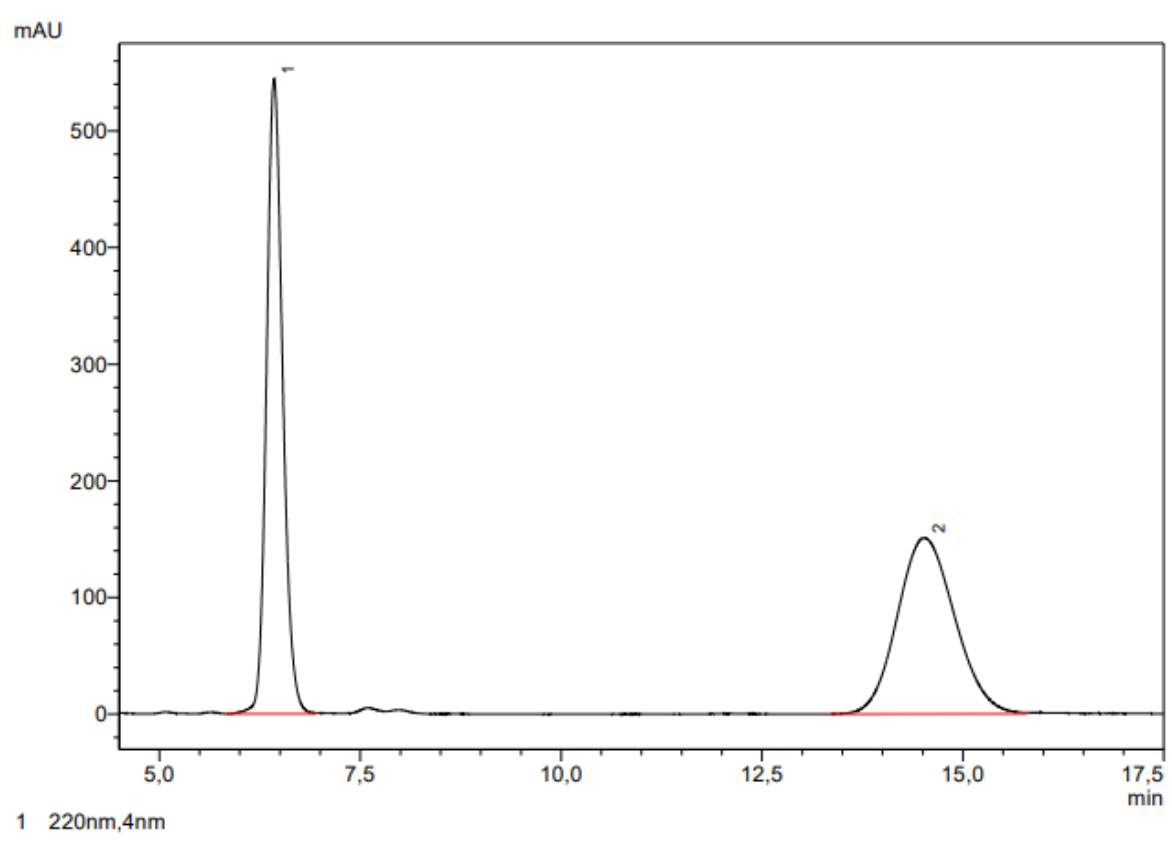

PDA Ch1 220nm

Peak #	Ret. Time	Area %	Name
1	6,43	50,46	1. Enantiomer
2	14,52	49,54	2. Enantiomer
Total		100,00	

tert-Butyl (6_R_,7_S_)-7-hydroxy-6-((_E_)-4-methoxy-4-oxobut-2-en-1-yl)-6,7-dihydrofuro[3,2-c]pyridine-5(4_H_)-carboxylate (10).

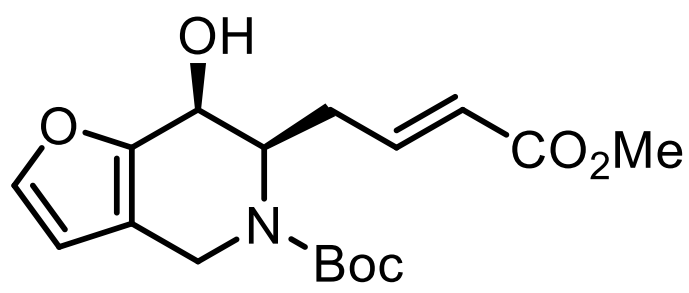

A flame-dried Schlenk flask was charged under argon with tris(_trans_-1,2-bis(4-_tert_-butylphenyl)ethene)nickel(0) (Ni(_t_Bu-stb)$_3$) (9.36 mg, 10.0 µmol) and (_S_)-AntPhos (4.07 mg, 11.0 µmol). The flask was evacuated and refilled with argon (3 cycles) before THF (0.25 mL) and triethylborane (1.0 M in THF, 0.25 mL, 0.25 mmol) were added. The resulting solution was stirred for 2 min before methyl (2_E_,4_E_)-5-((_tert_-butoxycarbonyl)((2-formylfuran-3-yl)methyl)amino)penta-2,4-dienoate **S29** (33.5 mg, 0.10 mmol) was introduced as a solution in THF (0.50 mL) under an argon counterflow. The flask was placed in a preheated metal block at 60 °C. The reaction mixture was stirred at this temperature for 18 h. Reaction control by thin layer chromatography indicated incomplete conversion of the starting material; therefore, more (_S_)-AntPhos (4.07 mg, 11.0 µmol) and tris(_trans_-1,2-bis(4-_tert_-butylphenyl)ethene)nickel(0) (Ni(_t_Bu-stb)$_3$) (9.36 mg, 10.0 µmol) were added. The mixture was stirred at 60 °C for another 18 h before it was cooled to 0 °C and pH 7 phosphate buffer (1.0 mL), aq. H_2O_2 (30% w/w in water, ≈0.1 mL), and ethyl acetate (2.0 mL) were introduced. The mixture was allowed to reach room temperature and was vigorously stirred for 30 min. Additional ethyl acetate (10 mL) and water (5 mL) were added, the organic phase was separated, and the aqueous layer was extracted with ethyl acetate (2 × 10 mL). The combined organic phases were washed with sat. aq. $Na_2S_2O_3$ (10 mL) and brine (10 mL), dried over $MgSO_4$, filtered, and concentrated _in vacuo_. The residue (dr ≈ 2:1) was purified by flash chromatography (SiO_2, _n_-pentane/ethyl acetate, 3:1) to give the product as a colorless oil (14.7 mg, 44%, 86% ee, single diastereomer).

$[\alpha]_D^{20}$ = +25.8 (c = 0.65, $CHCl_3$); ^{1}H NMR (600 MHz, [D$_6$]-DMSO, 383K): δ = 7.52 (dd, _J_ = 1.9, 0.9 Hz, 1H), 6.87 (ddd, _J_ = 15.7, 8.5, 6.1 Hz, 1H), 6.37 – 6.33 (m, 1H), 5.92 (ddd, _J_ = 15.6, 1.8, 1.2 Hz, 1H), 5.52 (d, _J_ = 5.4 Hz, 1H), 4.83 (d, _J_ = 5.6 Hz, 1H), 4.64 (ddd, _J_ = 10.4, 6.3, 3.7 Hz, 1H), 4.55 (dd, _J_ = 16.4, 1.9 Hz, 1H), 3.86 (dd, _J_ = 16.5, 2.4 Hz, 1H), 3.65 (s, 3H), 2.65 (dddd, _J_ = 15.3, 6.0, 3.8, 1.8 Hz, 1H), 2.22 – 2.13 (m, 1H), 1.42 (s, 9H); ^{13}C NMR (151 MHz, [D$_6$]-DMSO, 383K): δ = 165.2, 153.6, 148.4, 146.0, 142.0, 122.0, 114.3, 107.5, 79.0, 62.7, 53.7, 50.2, 37.2, 27.4(3), 27.4(1); IR (ATR): ṽ = 3436, 2926, 1723, 1693, 1398, 1316, 1227, 1168, 1141, 1044, 733 cm^{-1}; HRMS (ESI$^+$): _m/z_: calcd. for $C_{17}H_{23}NO_6Na$ [M+Na]$^+$: 360.14176, found: 360.14163.

The ee was determined by HPLC analysis: Chiralpak 150 mm IG-3, 3 µm, 4.6 mm ∅, acetonitrile/water = 40:60, v = 1.0 mL/min, λ = 220 nm, t(minor) = 7.00 min, t(major) = 8.04 min, (enantioenriched sample: left, racemic sample: right).

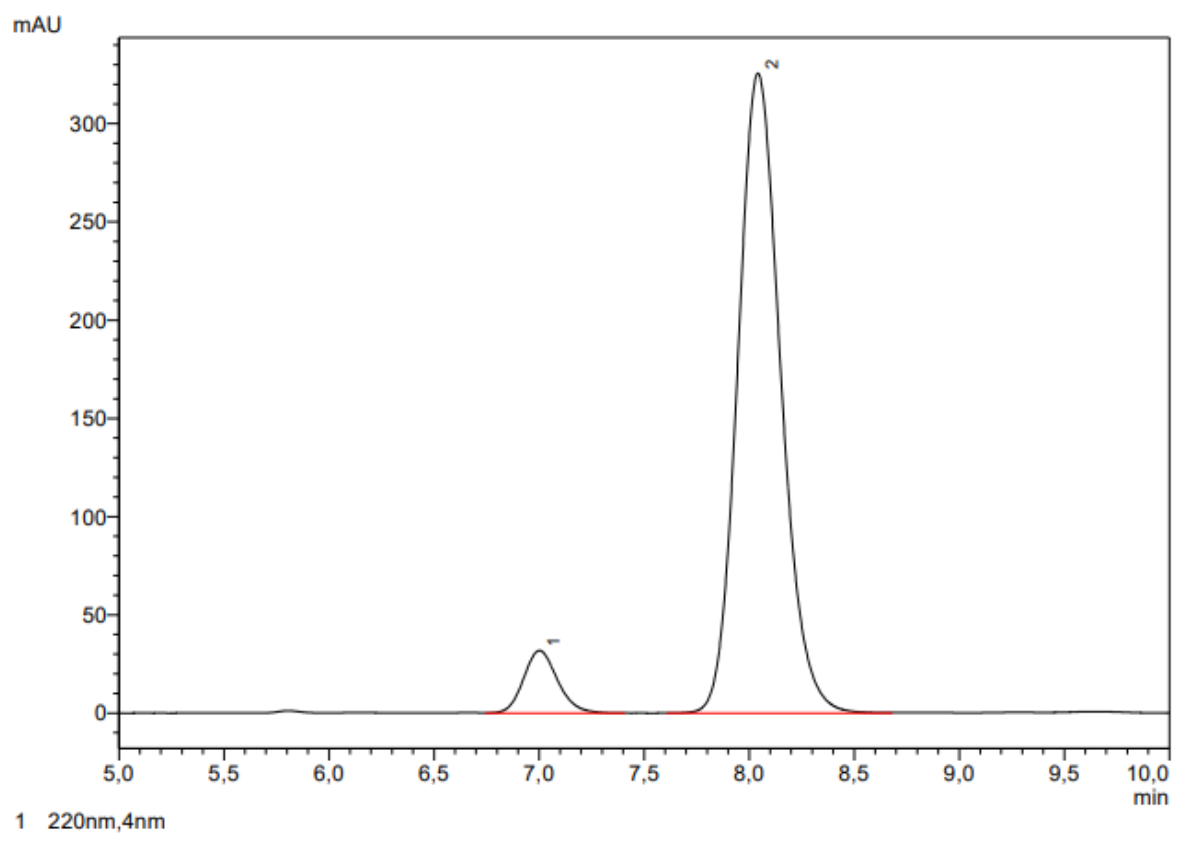

PDA Ch1 220nm

Peak #	Ret. Time	Area %	Name
1	7,00	7,18	1. Enantiomer
2	8,04	92,82	2. Enantiomer 85.6 % ee
Total		100,00	

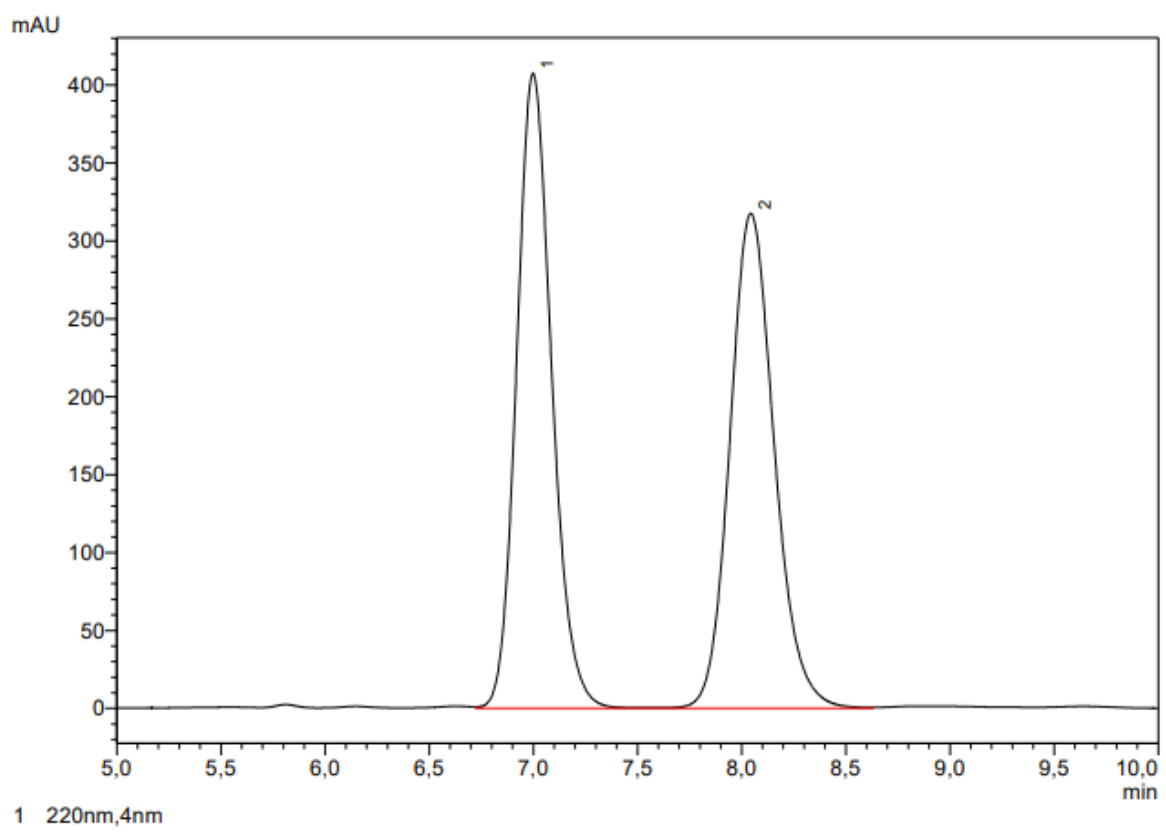

PDA Ch1 220nm

Peak #	Ret. Time	Area %	Name
1	7,00	50,35	1. Enantiomer
2	8,04	49,65	2. Enantiomer
Total		100,00	

tert-Butyl (2_R_,3_R_)-3-hydroxy-2-((_E_)-4-methoxy-4-oxobut-2-en-1-yl)piperidine-1-carboxylate (11).

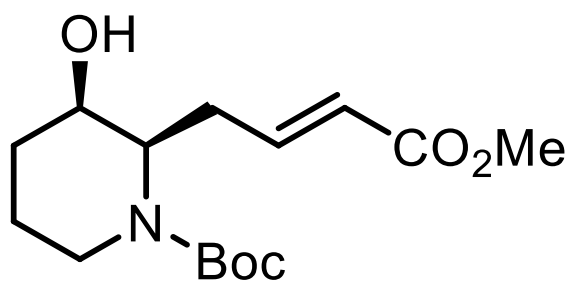

A flame-dried Schlenk flask was charged under argon with tris(_trans_-1,2-bis(4-_tert_-butylphenyl)ethene)nickel(0) (Ni(_t_Bu-stb)$_3$) (9.36 mg, 10.0 µmol) and (_S_)-AntPhos (4.07 mg, 11.0 µmol) and was then evacuated and refilled with argon (3 cycles). THF (0.25 mL) and diethylzinc (1.0 M in hexane, 0.25 mL, 0.25 mmol) were introduced and the resulting solution was stirred for 2 min before a solution of methyl (2_E_,4_E_)-5-((_tert_-butoxycarbonyl)(4-oxobutyl)amino)penta-2,4-dienoate **S30** (29.7 mg, 0.10 mmol) in THF (0.50 mL) was added dropwise over 5 h via syringe pump. After complete addition, the mixture was stirred for another 16 h at room temperature. The reaction was quenched at 0 °C with sat. aq. $NaHCO_3$ (2.0 mL) and ethyl acetate (2.0 mL). The mixture was vigorously stirred at room temperature for 30 min before it was diluted with ethyl acetate (10 mL) and water (5 mL). The aqueous phase was extracted with ethyl acetate (2 × 10 mL), the combined organic layers were washed with brine (15 mL), dried over $MgSO_4$, filtered and concentrated _in vacuo_. The residue (dr ≈ 1.2:1) was purified by flash chromatography (SiO_2, toluene/_tert_-butyl methyl ether, 1:1 → 1:2) to give the product as a colorless oil (10.1 mg, 34%, 90% ee, dr > 20:1).

$[\alpha]_D^{20}$ = −16.7 (c = 0.75, $CHCl_3$); ^1H NMR (600 MHz, [D$_6$]-DMSO, 383K): δ = 6.85 (ddd, _J_ = 15.6, 8.4, 6.4 Hz, 1H), 5.88 (dt, _J_ = 15.6, 1.7 Hz, 1H), 4.67 (br, 1H), 4.32 (dt, _J_ = 10.5, 4.7 Hz, 1H), 3.79 (dd, _J_ = 13.5, 4.9 Hz, 1H), 3.64 (s, 3H), 3.59 (dt, _J_ = 11.5, 5.5 Hz, 1H), 2.67 (td, _J_ = 13.3, 3.1 Hz, 1H), 2.54 (dddd, _J_ = 15.1, 6.4, 4.1, 1.7 Hz, 1H), 2.47 (dddd, _J_ = 15.1, 10.5, 8.4, 1.3 Hz, 1H), 1.66 (dq, _J_ = 11.9, 3.9 Hz, 1H), 1.64 – 1.59 (m, 1H), 1.46 (tdd, _J_ = 13.0, 12.0, 3.6 Hz, 1H), 1.39 (s, 9H), 1.36 (qt, _J_ = 13.0, 4.9 Hz, 1H); ^{13}C NMR (151 MHz, [D$_6$]-DMSO, 383K): δ = 165.3, 153.5, 146.6, 121.3, 78.2, 67.2, 54.5, 50.1, 36.7, 27.4, 26.9, 26.1, 23.2; IR (ATR): ṽ = 3429, 2938, 1723, 1689, 1666, 1415, 1272, 1214, 1154, 750, 688 cm^{-1}; HRMS (ESI$^+$): _m/z_: calcd. for $C_{15}H_{25}NO_5Na$ [M+Na]$^+$: 322.162493, found: 322.16266.

The ee was determined by HPLC analysis: Chiralpak 150 mm IG-3, 3 µm, 4.6 mm ∅, acetonitrile/water = 35:65, v = 1.0 mL/min, λ = 220 nm, t(minor) = 5.71 min, t(major) = 15.11 min, (enantioenriched sample: left, racemic sample: right).

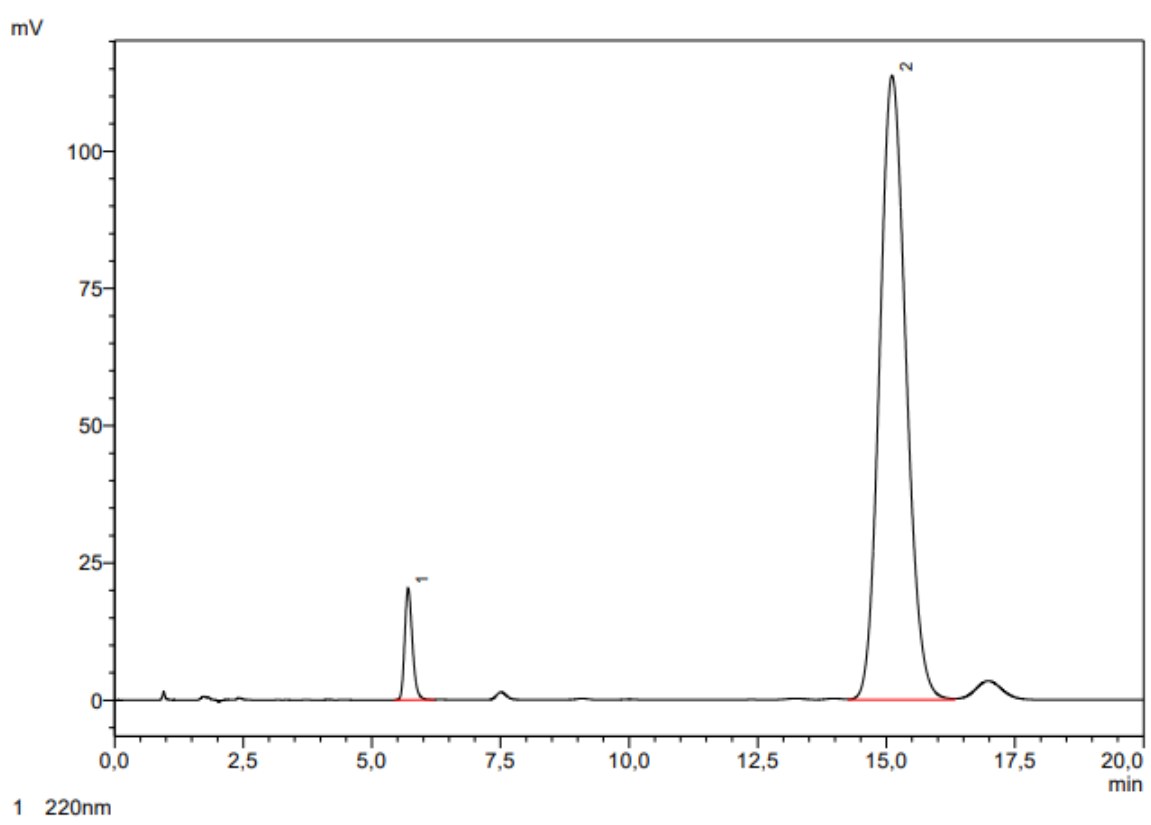

Detector A 220nm

Peak #	Ret. Time	Area %	Name
1	5,71	4,94	1. Enantiomer
2	15,11	95,06	2. Enantiomer 90.1 % ee
Total		100,00	

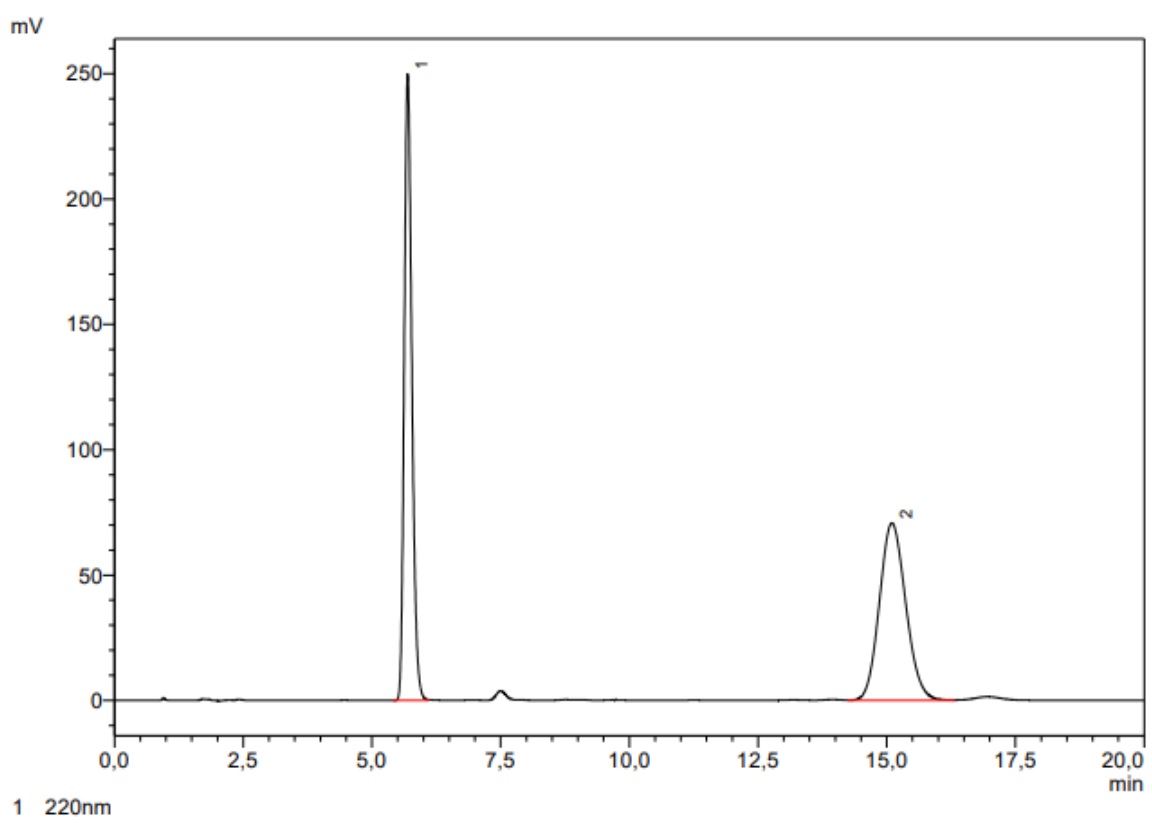

Detector A 220nm

Peak #	Ret. Time	Area %	Name
1	5,69	50,63	1. Enantiomer
2	15,10	49,37	2. Enantiomer
Total		100,00	

tert-Butyl (2_R_,3_R_)-3-hydroxy-2-((_E_)-4-methoxy-4-oxobut-2-en-1-yl)pyrrolidine-1-carboxylate (12a).

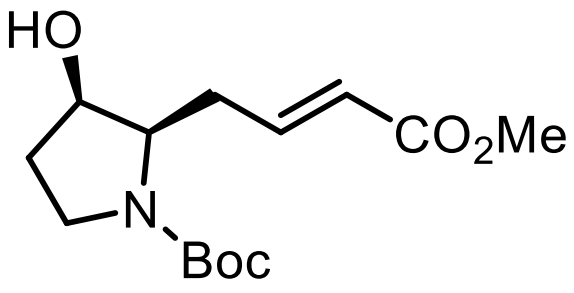

A flame-dried Schlenk flask was charged under argon with tris(*trans*-1,2-bis(4-*tert*-butylphenyl)ethene)nickel(0) (Ni(*t*Bu-stb)$_3$) (9.36 mg, 10.0 µmol) and (*S*)-AntPhos (4.07 mg, 11.0 µmol) and was then evacuated and refilled with argon (3 cycles). THF (0.25 mL) and diethylzinc (1.0 M in hexane, 0.25 mL, 0.25 mmol) were added and the resulting solution was stirred for 2 min before a solution of methyl (2*E*,4*E*)-5-((*tert*-butoxycarbonyl)(3-oxopropyl)amino)penta-2,4-dienoate **S31** (28.3 mg, 0.10 mmol) in THF (0.50 mL) was added dropwise over 5 h via syringe pump. After complete addition, the reaction mixture was stirred for another 16 h at room temperature. The mixture was cooled to 0 °C and sat. aq. $NaHCO_3$ (2.0 mL) and ethyl acetate (2.0 mL) were introduced. The mixture was allowed to warm to room temperature and was vigorously stirred for 30 min before it was diluted with ethyl acetate (10 mL) and water (5 mL). The aqueous phase was extracted with ethyl acetate (2 × 10 mL), the combined organic layers were washed with brine (15 mL), dried over $MgSO_4$ and filtered, and concentrated *in vacuo*. The residue was purified by flash chromatography (SiO_2, *n*-pentane/ethyl acetate, 1:1.5) to give the product as a colorless oil (14.2 mg, 50%, 96% ee).

$[\alpha]_D^{20}$ = –18.3 (c = 0.60, $CHCl_3$); ^{1}H NMR (600 MHz, [D$_6$]-DMSO, 373K): δ = 6.98 (ddd, *J* = 15.7, 7.8, 7.2 Hz, 1H), 5.83 (dt, *J* = 15.6, 1.5 Hz, 1H), 4.86 (br, 1H), 4.24 (dt, *J* = 7.9, 6.4 Hz, 1H), 3.77 (q, *J* = 6.3 Hz, 1H), 3.65 (s, 3H), 3.29 (dt, *J* = 10.8, 7.7 Hz, 1H), 3.24 (ddd, *J* = 10.8, 8.4, 4.9 Hz, 1H), 2.58 (dtd, *J* = 13.9, 6.9, 1.6 Hz, 1H), 2.45 – 2.40 (m, 1H), 1.95 (dddd, *J* = 12.6, 7.9, 6.3, 4.9 Hz, 1H), 1.72 (dq, *J* = 12.4, 7.9 Hz, 1H), 1.41 (s, 9H); ^{13}C NMR (151 MHz, [D$_6$]-DMSO, 373K): δ = 165.5, 153.3, 147.1, 121.2, 77.9, 69.7, 58.8, 50.3, 42.6, 31.1, 30.6, 27.6; IR (ATR): ṽ = 3410, 2973, 1723, 1694, 1688, 1395, 1266, 1170, 1131 cm^{-1}; HRMS (ESI$^+$): *m/z*: calcd. for $C_{14}H_{23}NO_5Na$ [M+Na]$^+$: 308.14684, found: 308.14642.

The ee was determined by HPLC analysis: Chiralpak 150 mm IG-3, 3 µm, 4.6 mm ∅, acetonitrile/water = 20:80 → 30:70 (over 19 min), then 30:70 → 60:30 (over 5 min), v = 1.0 mL/min, λ = 220 nm, t(minor) = 21.66 min, t(major) = 31.28 min, (enantioenriched sample: left, racemic sample: right).

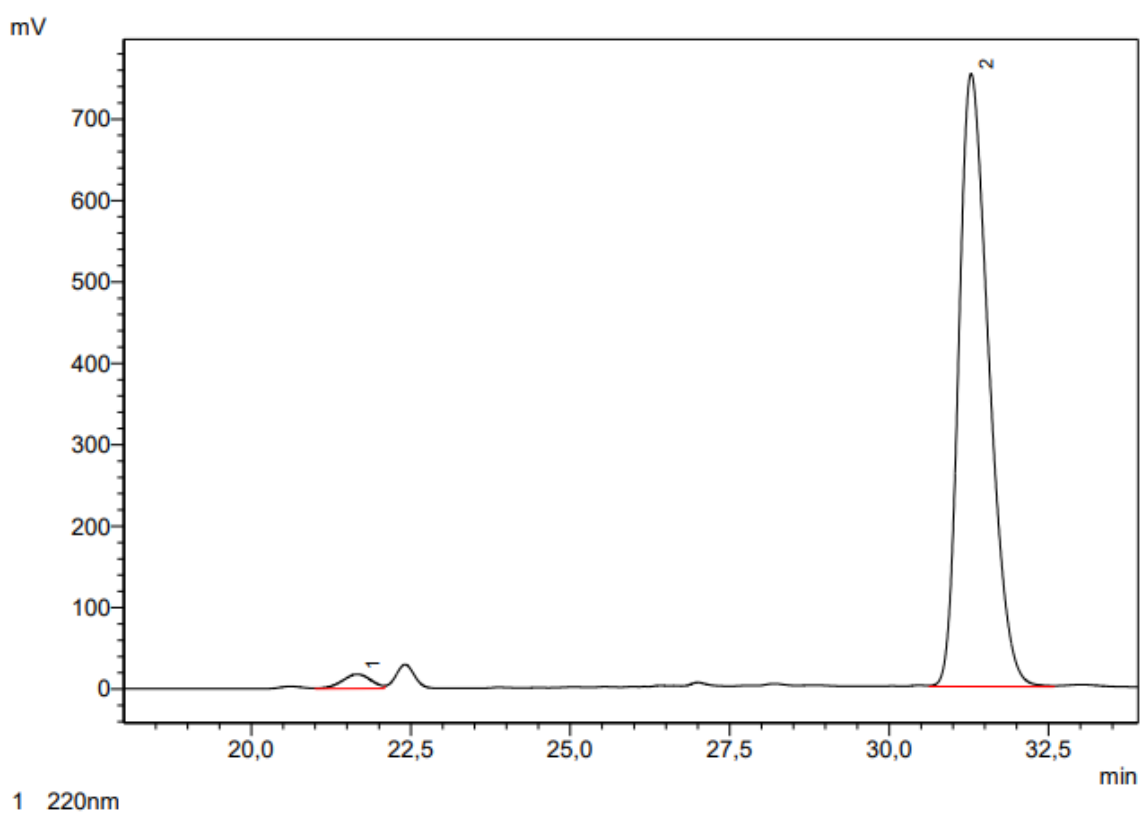

Detector A 220nm

Peak #	Ret. Time	Area %	Name
1	21,66	2,22	1. Enantiomer
2	31,28	97,78	2. Enantiomer 95.6 % ee
Total		100,00	

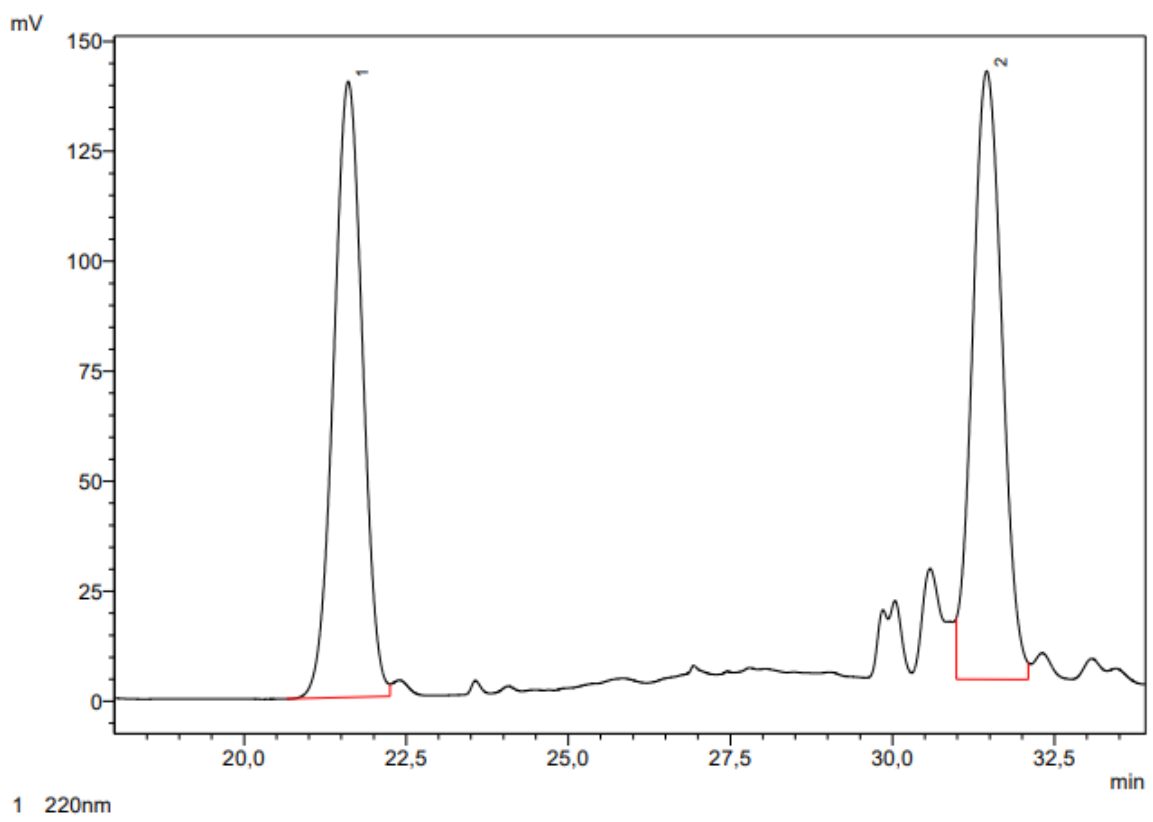

Detector A 220nm

Peak #	Ret. Time	Area %	Name
1	21,61	49,70	1. Enantiomer
2	31,45	50,30	2. Enantiomer
Total		100,00	

tert-Butyl (2*R*,3*R*)-3-hydroxy-2-((*E*)-4-methoxy-4-oxobut-2-en-1-yl)-4,4-dimethylpyrrolidine-1-carboxylate (12b).

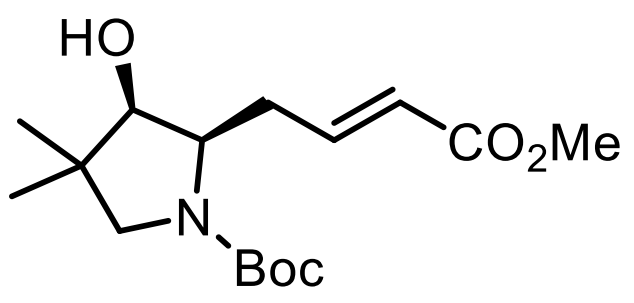

The title compound was prepared according to the general procedure **E** (reaction performed at room temperature) followed by oxidative workup **I**, using methyl (2*E*,4*E*)-5-((*tert*-butoxycarbonyl)(2,2-dimethyl-3-oxo-propyl)amino)penta-2,4-dienoate **S32** (31.1 mg, 0.10 mmol); the reaction time was 18 h. The crude material was purified by flash chromatography (SiO_2, *n*-pentane/ethyl acetate, 3:1) to give the product as a colorless oil (22.3 mg, 71%, 86% ee).

$[\alpha]_D^{20}$ = –29.4 (c = 0.50, $CHCl_3$); ^1H NMR (600 MHz, [D_6]-DMSO, 373K): δ = 6.97 (dt, *J* = 15.7, 7.2 Hz, 1H), 5.85 (dt, *J* = 15.7, 1.5 Hz, 1H), 4.82 (d, *J* = 5.7 Hz, 1H), 3.83 (dt, *J* = 7.7, 5.5 Hz, 1H), 3.66 (s, 3H), 3.63 (t, *J* = 5.3 Hz, 1H), 3.19 (d, *J* = 10.5 Hz, 1H), 3.01 (d, *J* = 10.4 Hz, 1H), 2.69 – 2.58 (m, 2H), 1.41 (s, 9H), 0.99 (s, 3H), 0.88 (s, 3H); ^{13}C NMR (151 MHz, [D_6]-DMSO, 373K): δ = 165.5, 154.0, 147.0, 121.1, 78.0, 76.9, 59.8, 56.1, 50.3, 40.4, 31.0, 27.6, 24.4, 19.5; IR (ATR): ṽ = 3438, 2973, 1725, 1694, 1666, 1401, 1367, 1267, 1162, 1114 cm^{-1}; HRMS (ESI$^+$): *m/z*: calcd. for $C_{16}H_{27}NO_5Na$ $[M+Na]^+$: 336.17814, found: 336.17807.

The ee was determined by HPLC analysis: Chiralpak 150 mm IG-3, 3 µm, 4.6 mm ∅, acetonitrile/water = 40:60, v = 1.0 mL/min, λ = 220 nm, t(minor) = 7.70 min, t(major) = 19.73 min, (enantioenriched sample: left, racemic sample: right).

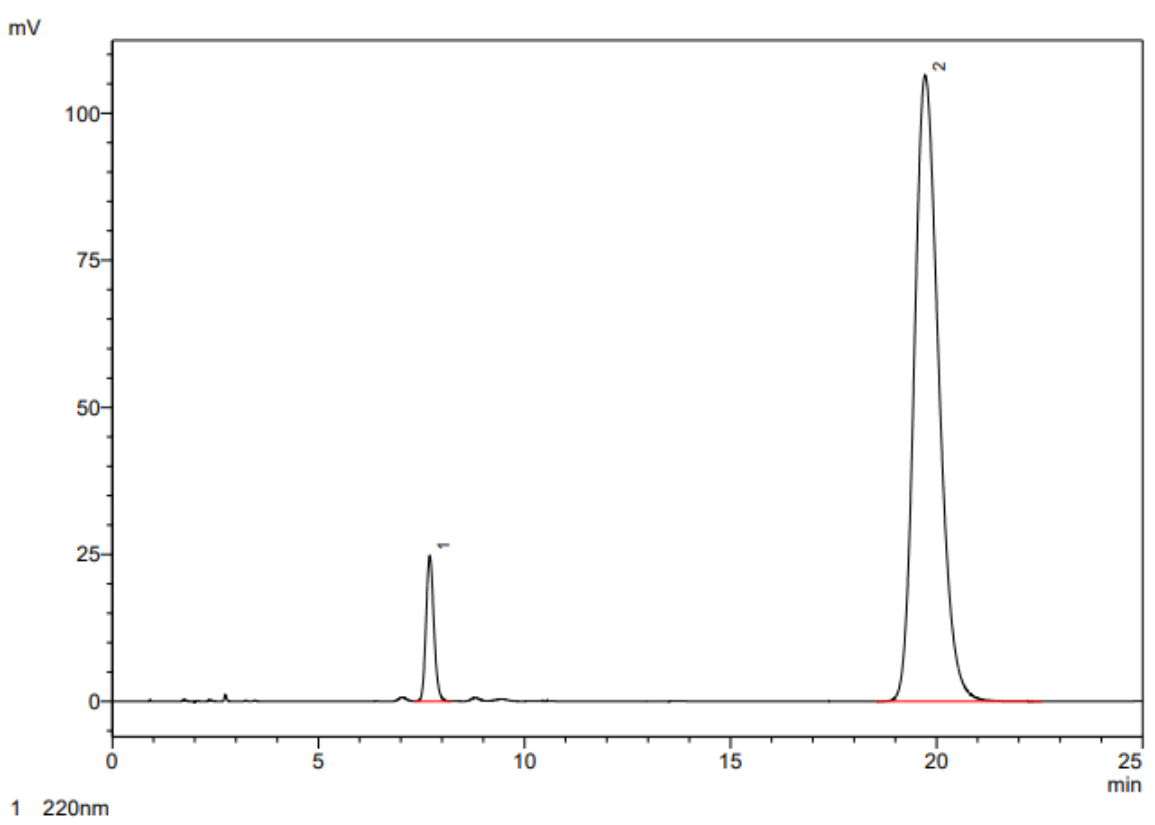

Detector A 220nm

Peak #	Ret. Time	Area %	Name
1	7,70	7,02	1. Enantiomer
2	19,73	92,98	2. Enantiomer 86.0 % ee
Total		100,00	

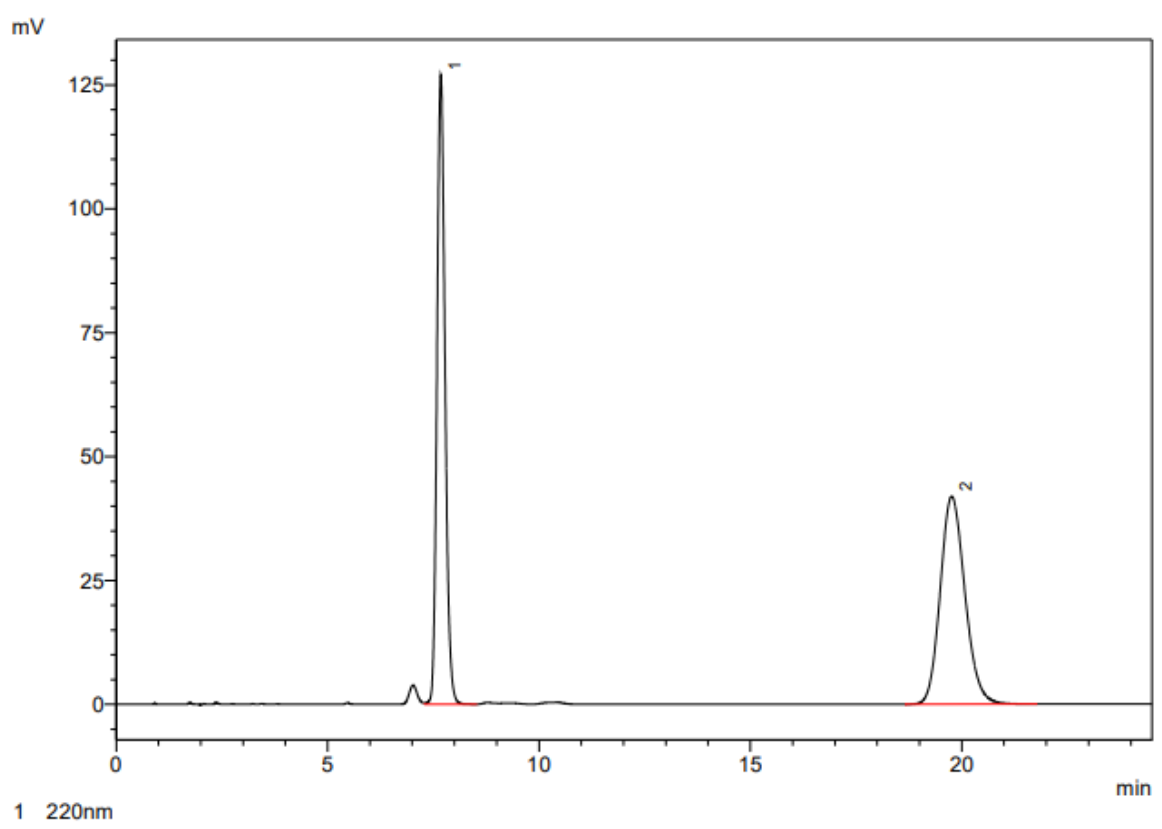

Detector A 220nm

Peak #	Ret. Time	Area %	Name
1	7,68	49,81	1. Enantiomer
2	19,76	50,19	2. Enantiomer
Total		100,00	

tert-Butyl (7R,8R)-8-hydroxy-7-((E)-4-methoxy-4-oxobut-2-en-1-yl)-2-oxa-6-azaspiro[3.4]octane-6-carboxylate (13).

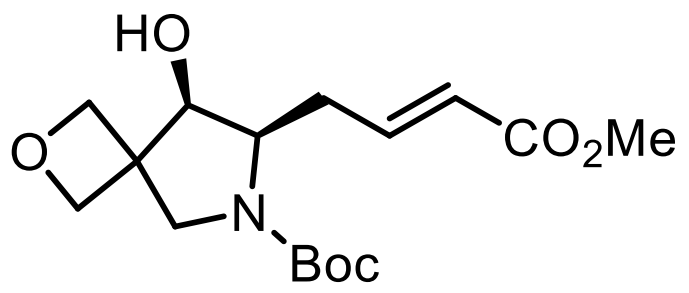

The title compound was prepared according to the general procedure **E** followed by oxidative workup **I**, using methyl (2*E*,4*E*)-5-((*tert*-butoxycarbonyl)((3-formyloxetan-3-yl)methyl)amino)penta-2,4-dienoate **S33** (32.5 mg, 0.10 mmol); the reaction time was 18 h. The crude material was purified by flash chromatography (SiO_2, *n*-pentane/ethyl acetate, 1:2) to give the product as a colorless oil (21.8 mg, 67%, 95% ee).

$[\alpha]_D^{20}$ = +3.8 (c = 0.48, $CHCl_3$); ^{1}H NMR (600 MHz, [D_6]-DMSO, 383K): δ = 6.91 (ddd, *J* = 15.8, 7.7, 7.0 Hz, 1H), 5.82 (dt, *J* = 15.6, 1.5 Hz, 1H), 5.38 (br, 1H), 4.85 (d, *J* = 5.9 Hz, 1H), 4.51 (d, *J* = 5.9 Hz, 1H), 4.35 (d, *J* = 5.9 Hz, 1H), 4.31 (d, *J* = 5.9 Hz, 1H), 4.14 (d, *J* = 6.2 Hz, 1H), 3.77 (q, *J* = 6.3 Hz, 1H), 3.66 (s, 3H), 3.63 (d, *J* = 11.4 Hz, 1H), 3.58 (d, *J* = 11.3 Hz, 1H), 2.55 – 2.51 (m, 1H), 2.32 – 2.21 (m, 1H), 1.41 (s, 9H); ^{13}C NMR (151 MHz, [D_6]-DMSO, 383K): δ = 165.4, 153.3, 146.3, 121.3, 78.3, 76.9, 74.2, 72.7, 58.7, 52.8, 50.2, 46.8, 31.1, 27.5; IR (ATR): ṽ = 3403, 2951, 1722, 1694, 1664, 1395, 1268, 1166, 1100, 979 cm^{-1}; HRMS (ESI$^+$): *m/z*: calcd. for $C_{16}H_{25}NO_6Na$ [M+Na]$^+$: 350.15741, found: 350.15752.

The ee was determined by HPLC analysis: Chiralpak 150 mm IG-3, 3 µm, 4.6 mm ∅, acetonitrile/water = 40:60, v = 1.0 mL/min, λ = 220 nm, t(minor) = 5.92 min, t(major) = 10.87 min, (enantioenriched sample: left, racemic sample: right).

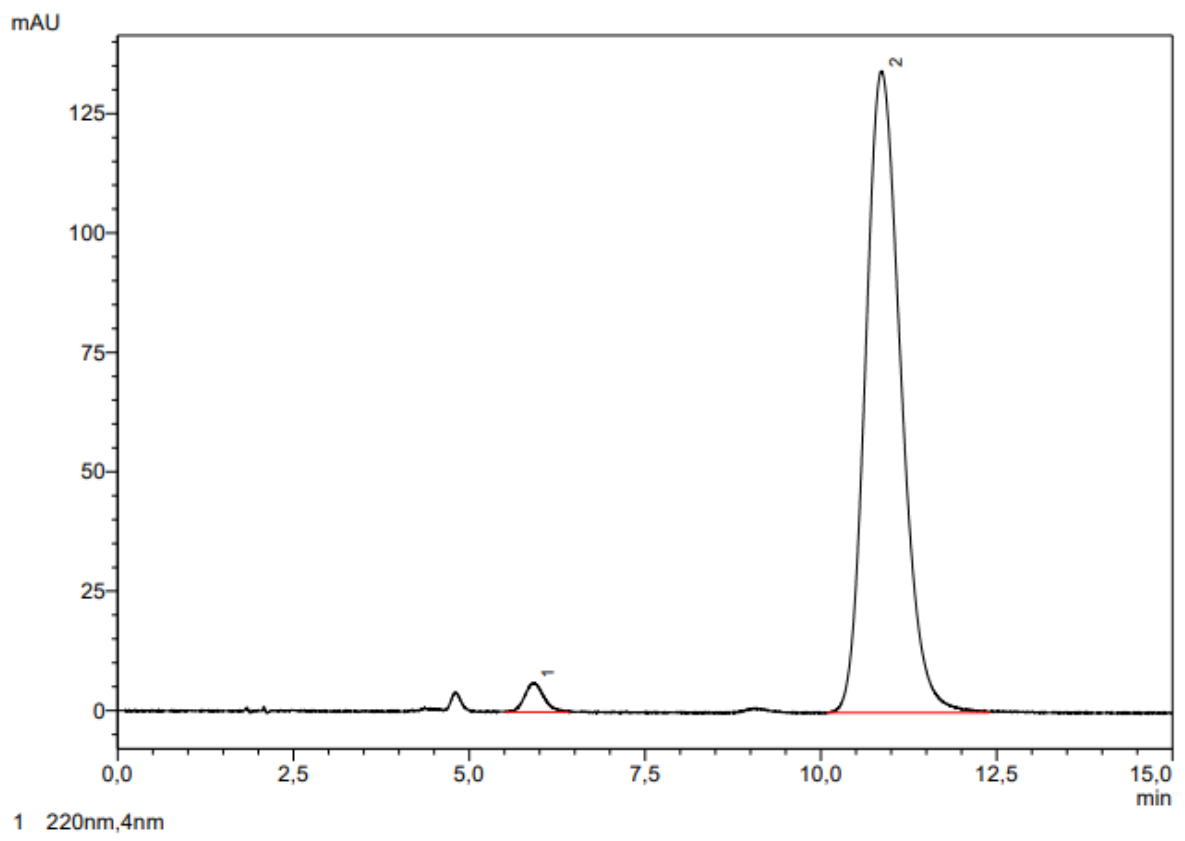

PDA Ch1 220nm

Peak #	Ret. Time	Area %	Name
1	5,92	2,40	1. Enantiomer
2	10,87	97,60	2. Enantiomer 95.2 % ee
Total		100,00	

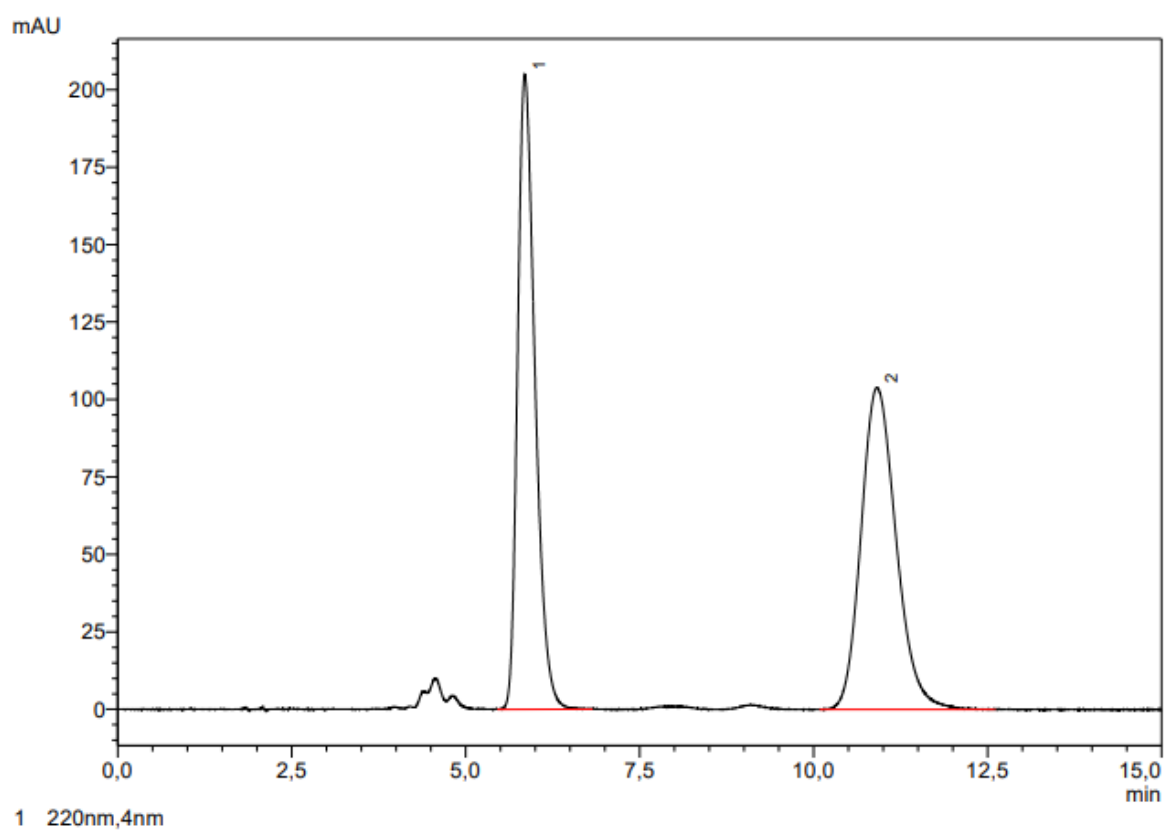

PDA Ch1 220nm

Peak #	Ret. Time	Area %	Name
1	5,85	49,58	1. Enantiomer
2	10,91	50,42	2. Enantiomer
Total		100,00	

tert-Butyl (3_R_,4_S_)-3-allyl-4-hydroxy-3,4-dihydroisoquinoline-2(1_H_)-carboxylate (14).

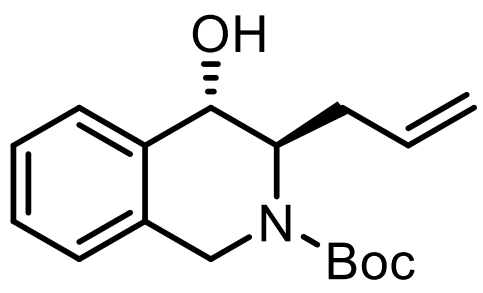

The title compound was prepared according to the general procedure **E** (the reaction was performed at room temperature) followed by oxidative workup **I**, using *tert*-butyl (*E*)-buta-1,3-dien-1-yl(2-formylbenzyl)carbamate **S34** (28.7 mg, 0.10 mmol); the reaction time was 20 h. The crude material was purified by flash chromatography (SiO_2, *n*-pentane/ethyl acetate, 2:1) to give the product as a pale yellow oil (25.1 mg, 87%, 28% ee, rr ≈ 11:1). ***Note:*** *The product was obtained as a ~11:1 mixture of regioisomers that could not be separated.*

^{1}H NMR (600 MHz, [D$_6$]-DMSO, 373K): δ = 7.33 – 7.21 (m, 3H), 7.21 – 7.17 (m, 1H), 5.83 – 5.67 (m, 1H), 5.01 – 4.92 (m, 2H), 4.76 (d, *J* = 17.1 Hz, 1H), 4.48 (d, *J* = 2.6 Hz, 1H), 4.39 (td, *J* = 7.4, 2.5 Hz, 1H), 4.17 (d, *J* = 17.1 Hz, 1H), 2.08 – 1.89 (m, 2H), 1.47 (s, 9H); ^{13}C NMR (151 MHz, [D$_6$]-DMSO, 373K): δ = 154.4, 134.6, 134.6, 132.5, 129.1, 127.0, 125.9, 125.3, 116.0, 78.2, 67.9, 55.8, 41.9, 34.5, 27.7; IR (ATR): ṽ = 3404, 2976, 1690, 1410, 1366, 1244, 1169, 1019, 767 cm^{-1}; HRMS (ESI$^+$): *m/z*: calcd. for $C_{17}H_{23}NO_3Na$ [M+Na]$^+$: 312.15701, found: 312.15658.

The ee was determined by 2D-HPLC analysis. First dimension = 50 mm Nucleodur C18 Gravity SB 1.8 µm, 4.6 mm Ø, methanol/water = 50:50 → 10:90, v = 1.0 mL/min, λ = 220 nm. Second Dimension = 150 mm Chiralpak IG-3, 4.6 mm Ø, acetonitrile/water = 50:50, v = 1.0 ml/min, λ = 220 nm, t(major) = 5.57 min, t(minor) = 8.25 min.

First dimension (left side: enantioenriched sample, right side: racemic sample):

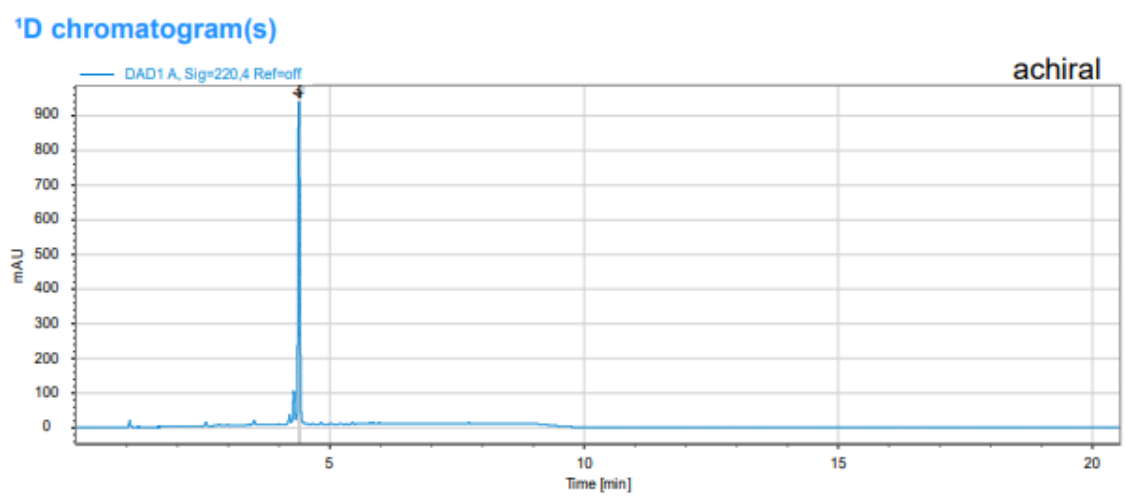

Sampling table (¹D)

Cut group	Cut #	¹D Cut start [min]	¹D Ret. time [min]	¹D Duration [min]	Trigger	²D Run start [min]
	1	4.38	4.400	0.04	Peak	4.42

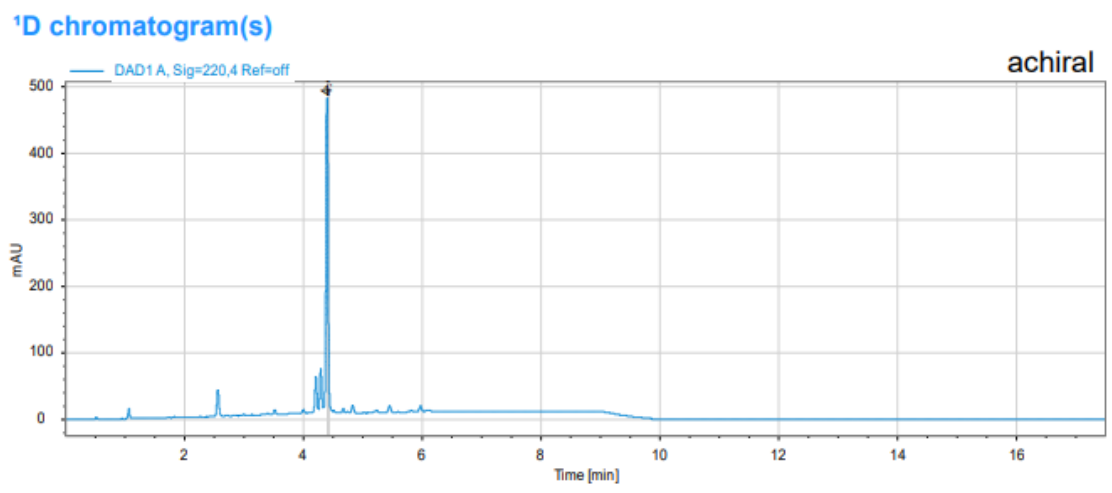

Sampling table (¹D)

Cut group	Cut #	¹D Cut start [min]	¹D Ret. time [min]	¹D Duration [min]	Trigger	²D Run start [min]
	1	4.39	4.406	0.04	Peak	4.43

Second dimension (left side: enantioenriched sample, right side: racemic sample):

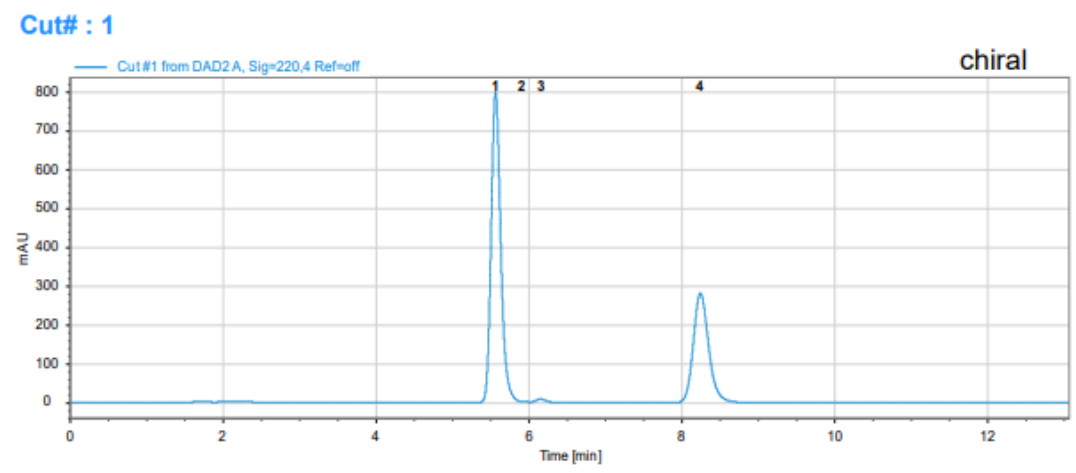

Component table

Signal: DAD2 A, Sig=220,4 Ref=off

ee = 28.4%

Component	¹D Sampling range [min]	Ret.Time ²D [min]	Area	Area%	
1	4.38 - 4.42	5.568	6982.553	63.618	Hauptisomer 1. Enantiomer
2	4.38 - 4.42	5.902	15.398	0.140	
3	4.38 - 4.42	6.155	81.027	0.738	
4	4.38 - 4.42	8.248	3896.843	35.504	Hauptisomer 2. Enantiomer

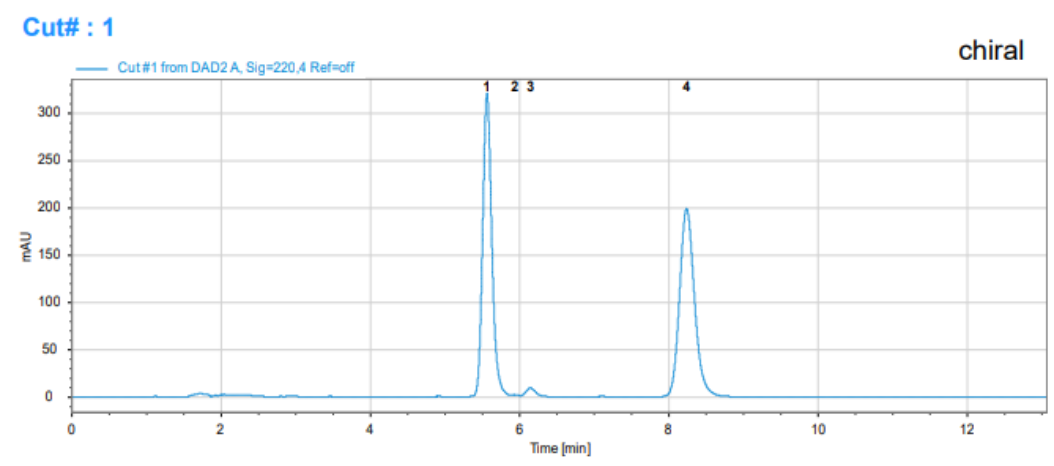

Component table

Signal: DAD2 A, Sig=220,4 Ref=off

Component	¹D Sampling range [min]	Ret.Time ²D [min]	Area	Area%	
1	4.39 - 4.43	5.568	2742.215	49.064	Hauptisomer 1. Enantiomer
2	4.39 - 4.43	5.935	13.831	0.247	
3	4.39 - 4.43	6.149	80.307	1.437	
4	4.39 - 4.43	8.243	2752.680	49.251	Hauptisomer 2. Enantiomer

tert-Butyl (3_R_,4_S_)-3-cinnamyl-4-hydroxy-3,4-dihydroisoquinoline-2(1_H_)-carboxylate (15).

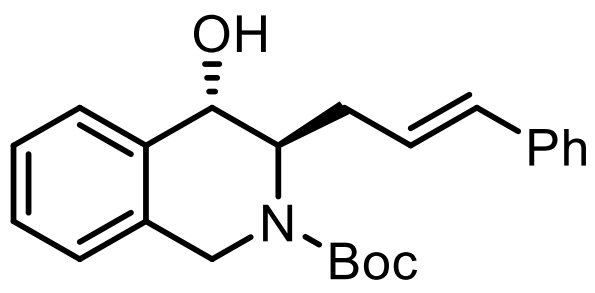

The title compound was prepared according to the general procedure **D** followed by oxidative workup **I**, using _tert_-butyl (2-formylbenzyl)((1_E_,3_E_)-4-phenylbuta-1,3-dien-1-yl)carbamate **S35** (36.3 mg, 0.10 mmol); the reaction time was 72 h. The crude material was purified by flash chromatography (SiO_2, _n_-pentane/ethyl acetate, 2:1) to give the product as colorless solid (28.6 mg, 78%, 14% ee).

^{1}H NMR (600 MHz, [D$_6$]-DMSO, 383K): δ = 7.36 – 7.23 (m, 7H), 7.22 – 7.17 (m, 2H), 6.34 (dt, _J_ = 15.9, 1.5 Hz, 1H), 6.16 (ddd, _J_ = 15.8, 7.5, 6.9 Hz, 1H), 4.88 (br, 1H), 4.78 (d, _J_ = 17.1 Hz, 1H), 4.54 (d, _J_ = 2.6 Hz, 1H), 4.47 (ddd, _J_ = 8.6, 6.4, 2.5 Hz, 1H), 4.24 (d, _J_ = 17.1 Hz, 1H), 2.25 – 2.15 (m, 1H), 2.13 (dddd, _J_ = 14.3, 8.2, 7.5, 1.4 Hz, 1H), 1.42 (s, 9H); ^{13}C NMR (151 MHz, [D$_6$]-DMSO, 383K): δ = 154.4, 136.8, 134.6, 132.5, 130.9, 129.0, 127.7, 127.0, 126.5, 126.3, 125.9, 125.3(1), 125.2(6), 78.2, 68.1, 56.2, 42.0, 33.7, 27.6; IR (ATR): ṽ = 3414, 2976, 1688, 1410, 1365, 1243, 1167, 1125, 1014, 966, 746 cm^{-1}; HRMS (ESI$^+$): _m/z_: calcd. for $C_{23}H_{27}NO_3Na$ [M+Na]$^+$: 388.18831, found: 388.18816.

The ee was determined by HPLC analysis: Chiralpak 150 mm IG-3, 3 µm, 4.6 mm ∅, acetonitrile/water = 55:45, v = 1.0 mL/min, λ = 254 nm, t(minor) = 12.22 min, t(major) = 28.68 min, (enantioenriched sample: left, racemic sample: right).

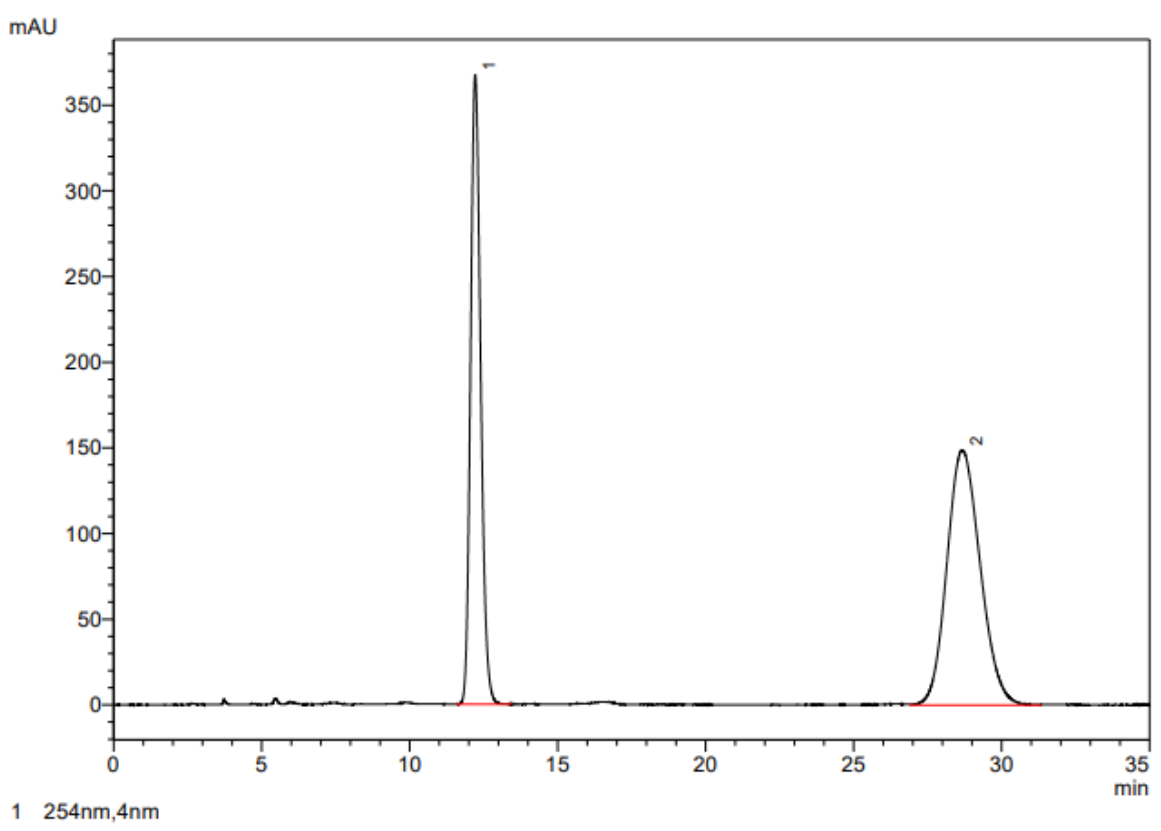

PDA Ch1 254nm

Peak #	Ret. Time	Area %	Name
1	12,22	42,80	1. Enantiomer
2	28,68	57,20	2. Enantiomer 14.4 % ee
Total		100,00	

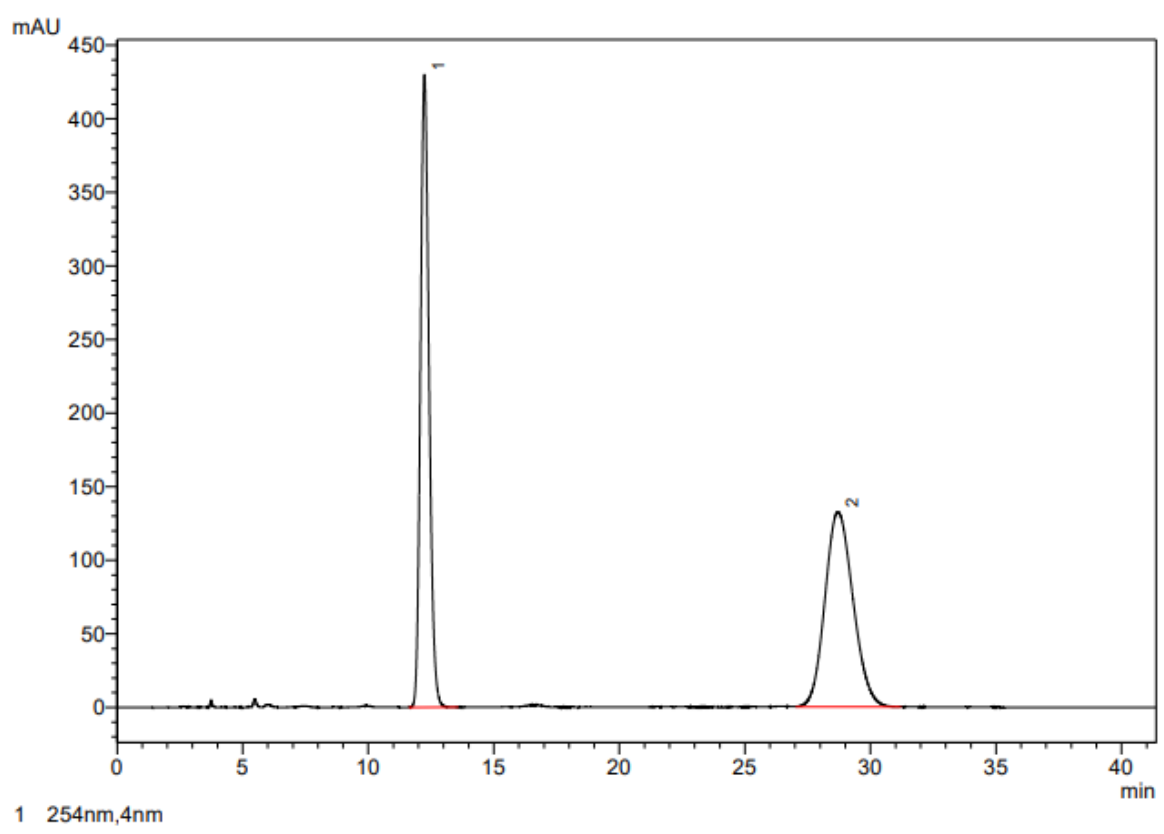

PDA Ch1 254nm

Peak #	Ret. Time	Area %	Name
1	12,24	49,76	1. Enantiomer
2	28,70	50,24	2. Enantiomer
Total		100,00	

tert-Butyl (3_S_,4_R_)-4-hydroxy-3-((_E_)-4-methoxy-4-oxobut-2-en-1-yl)-3,4-dihydroquinoline-1(2_H_)-carboxylate (16a).

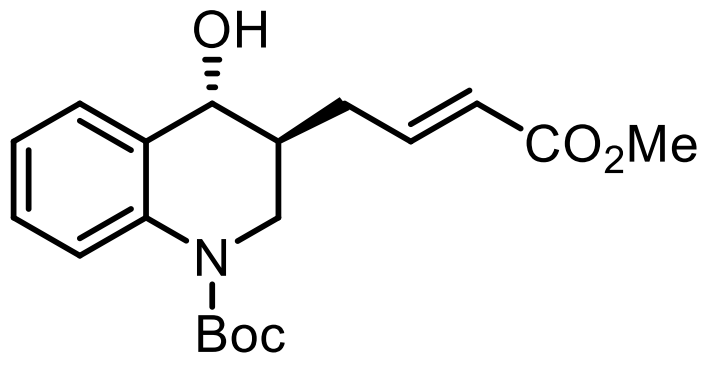

The title compound was prepared according to the general procedure **E** followed by oxidative workup **I**, using methyl (2*E*,4*E*)-6-((*tert*-butoxycarbonyl)(2-formylphenyl)amino)hexa-2,4-dienoate **S36** (34.5 mg, 0.10 mmol); the reaction time was 18 h. The crude material (dr ≈ 3:1) was purified by flash chromatography (SiO_2, *n*-pentane/ethyl acetate, 3:1) to give the product as a colorless oil (20.0 mg, 58%, 5% ee, dr > 20:1).

^{1}H NMR (400 MHz, $CDCl_3$): δ = 7.83 (dd, *J* = 8.5, 1.1 Hz, 1H), 7.38 (dd, *J* = 7.7, 1.7 Hz, 1H), 7.29 – 7.23 (m, 1H), 7.14 – 6.92 (m, 2H), 5.87 (dt, *J* = 15.5, 1.4 Hz, 1H), 4.43 (d, *J* = 4.6 Hz, 1H), 3.82 (dd, *J* = 13.4, 5.6 Hz, 1H), 3.77 (d, *J* = 3.4 Hz, 1H), 3.74 (s, 3H), 2.31 (dddd, *J* = 14.0, 6.8, 5.6, 1.5 Hz, 1H), 2.23 – 2.05 (m, 2H), 1.78 (br, 1H), 1.53 (s, 9H); ^{13}C NMR (101 MHz, $CDCl_3$): δ = 166.8, 153.7, 146.1, 137.7, 129.1, 129.0, 128.4, 123.9, 123.4, 123.3, 81.7, 70.3, 51.7, 44.5, 40.4, 32.2, 28.4; IR (ATR): ṽ = 3450, 2925, 1701, 1660, 1489, 1367, 1248, 1161, 1147, 1020, 757 cm^{-1}; HRMS (ESI$^+$): *m/z*: calcd. for $C_{19}H_{25}NO_5Na$ $[M+Na]^+$: 370.162493, found: 370.16240.

The ee was determined by HPLC analysis: Chiralpak 150 mm IB-N-3, 3 µm, 4.6 mm ∅, acetonitrile/water = 40:60, v = 1.0 mL/min, λ = 240 nm, t(major) = 12.35 min, t(minor) = 13.09 min, (enantioenriched sample: left, racemic sample: right).

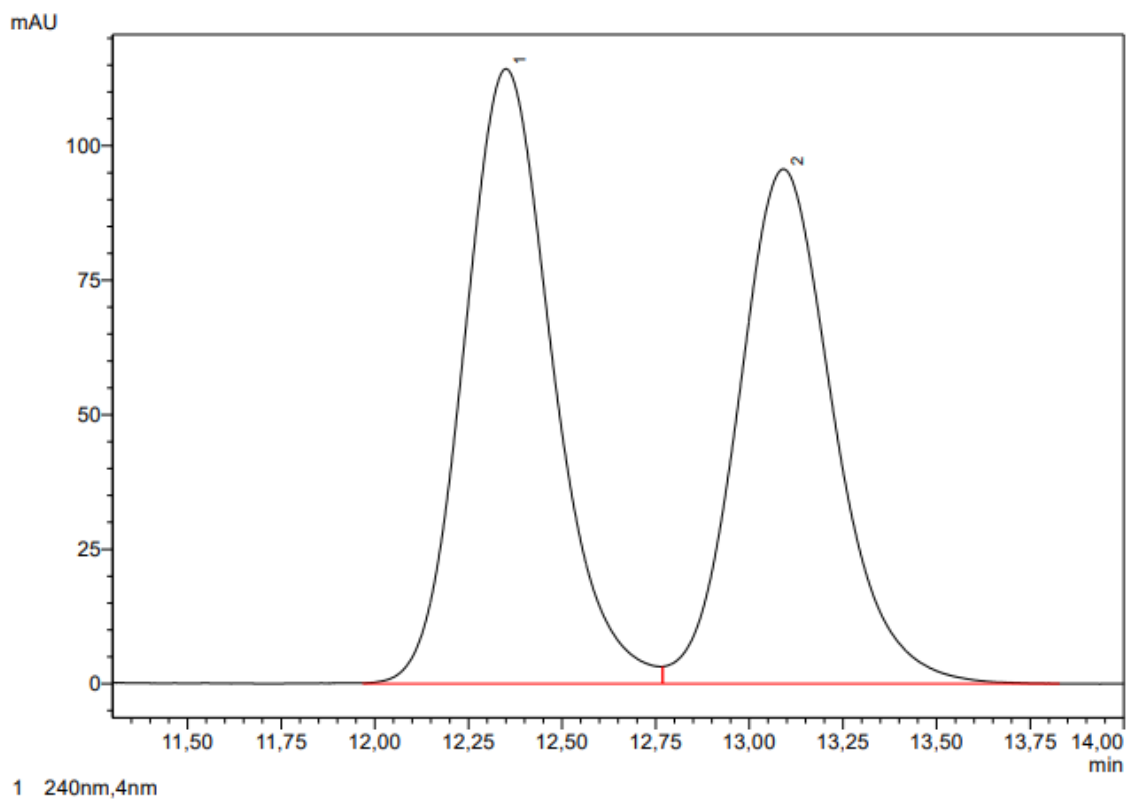

PDA Ch1 240nm

Peak #	Ret. Time	Area %	Name
1	12,35	52,52	1. Enantiomer 5.0 % ee
2	13,09	47,48	2. Enantiomer
Total		100,00	

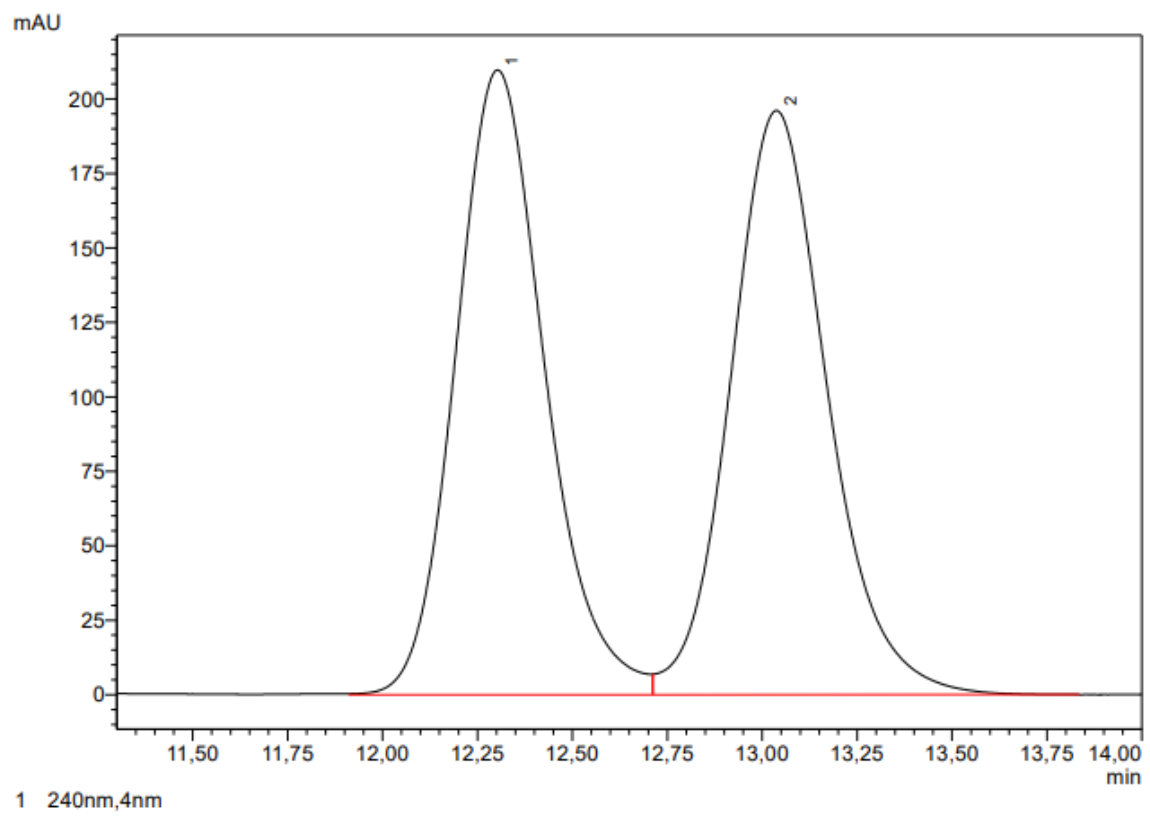

PDA Ch1 240nm

Peak #	Ret. Time	Area %	Name
1	12,30	49,63	1. Enantiomer
2	13,04	50,37	2. Enantiomer
Total		100,00	

Methyl (*E*)-4-((3*S*,4*R*)-4-hydroxychroman-3-yl)but-2-enoate (16b).

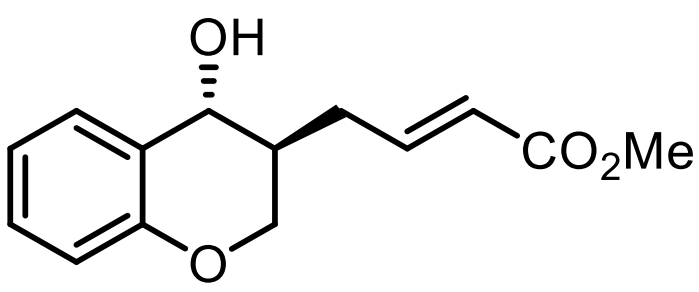

The title compound was prepared according to the general procedure **E** followed by oxidative workup **I**, using methyl (2*E*,4*E*)-6-(2-formylphenoxy)hexa-2,4-dienoate **S37** (17.2 mg, 0.07 mmol); the reaction time was 24 h. The crude material (dr ≈ 5:1) was purified by flash chromatography (SiO_2, *n*-pentane/*tert*-butyl methyl ether, 1.5:1 → 1:1) to give the product as a yellow oil (14.0 mg, 81%, 2% ee, dr > 20:1). ^1H NMR (400 MHz, $CDCl_3$): δ = 7.31 (dd, *J* = 7.6, 1.7 Hz, 1H), 7.23 (ddd, *J* = 8.2, 7.2, 1.7 Hz, 1H), 7.07 – 6.91 (m, 2H), 6.85 (dd, *J* = 8.3, 1.2 Hz, 1H), 5.87 (dt, *J* = 15.6, 1.5 Hz, 1H), 4.47 (d, *J* = 4.1 Hz, 1H), 4.28 (dd, *J* = 11.3, 2.6 Hz, 1H), 4.03 (ddd, *J* = 11.3, 4.5, 1.1 Hz, 1H), 3.74 (s, 3H), 2.34 – 2.16 (m, 2H), 2.12 (dtd, *J* = 9.1, 4.0, 2.5 Hz, 1H); ^{13}C NMR (101 MHz, $CDCl_3$): δ = 166.8, 154.2, 146.0, 130.1, 130.0, 123.4, 123.1, 121.2, 117.1, 67.7, 65.0, 51.7, 39.1, 31.2; IR (ATR): ṽ = 3438, 2926, 1722, 1707, 1654, 1610, 1488, 1456, 1319, 1267, 1225, 1038, 759 cm^{-1}; HRMS (ESI^+): *m/z*: calcd. for $C_{14}H_{16}O_4Na$ $[M+Na]^+$: 271.09408, found: 271.09376.

The ee was determined by HPLC analysis: Chiralpak 150 mm IB-N-3, 3 µm, 4.6 mm ∅, acetonitrile/water = 30:70, v = 1.0 mL/min, λ = 220 nm, t(minor) = 13.75 min, t(major) = 14.47 min (enantioenriched sample: left, racemic sample: right).

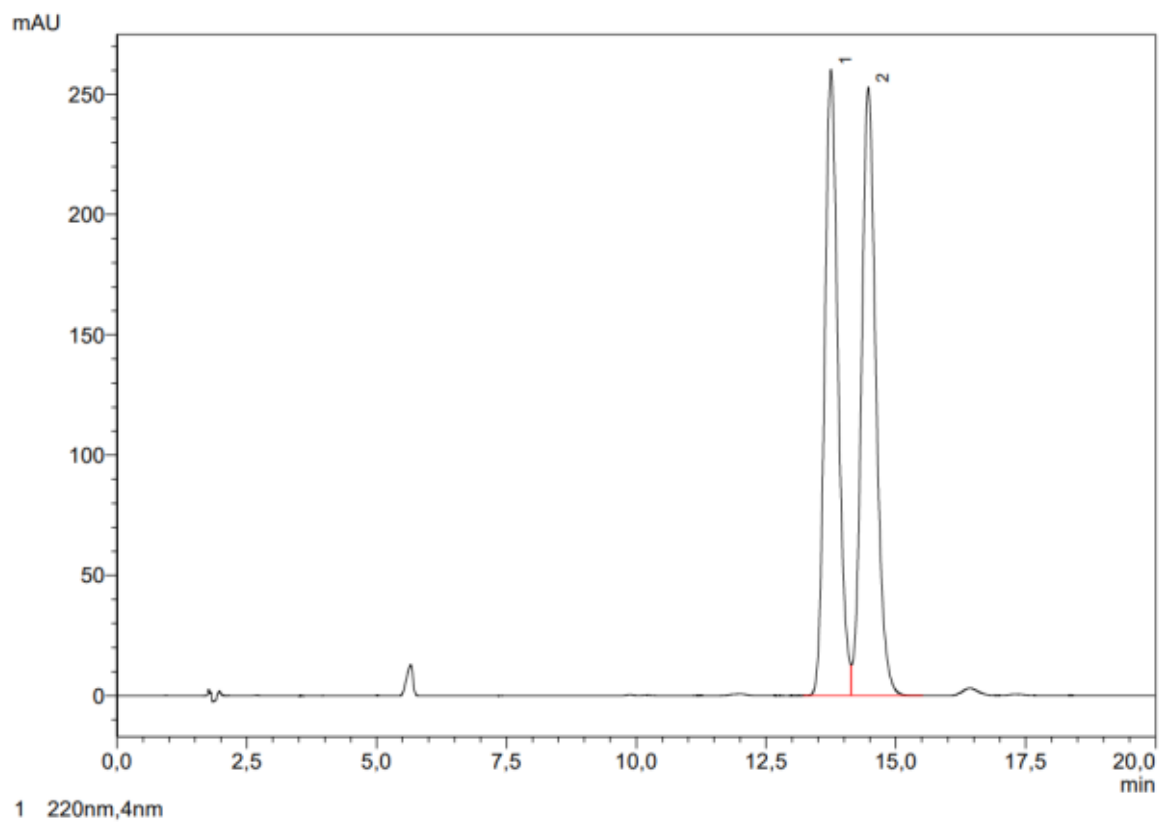

PDA Ch1 220nm

Peak #	Ret. Time	Area %	Name
1	13,75	48,95	1. Enantiomer
2	14,47	51,05	2. Enantiomer 2.1 % ee
Total		100,00	

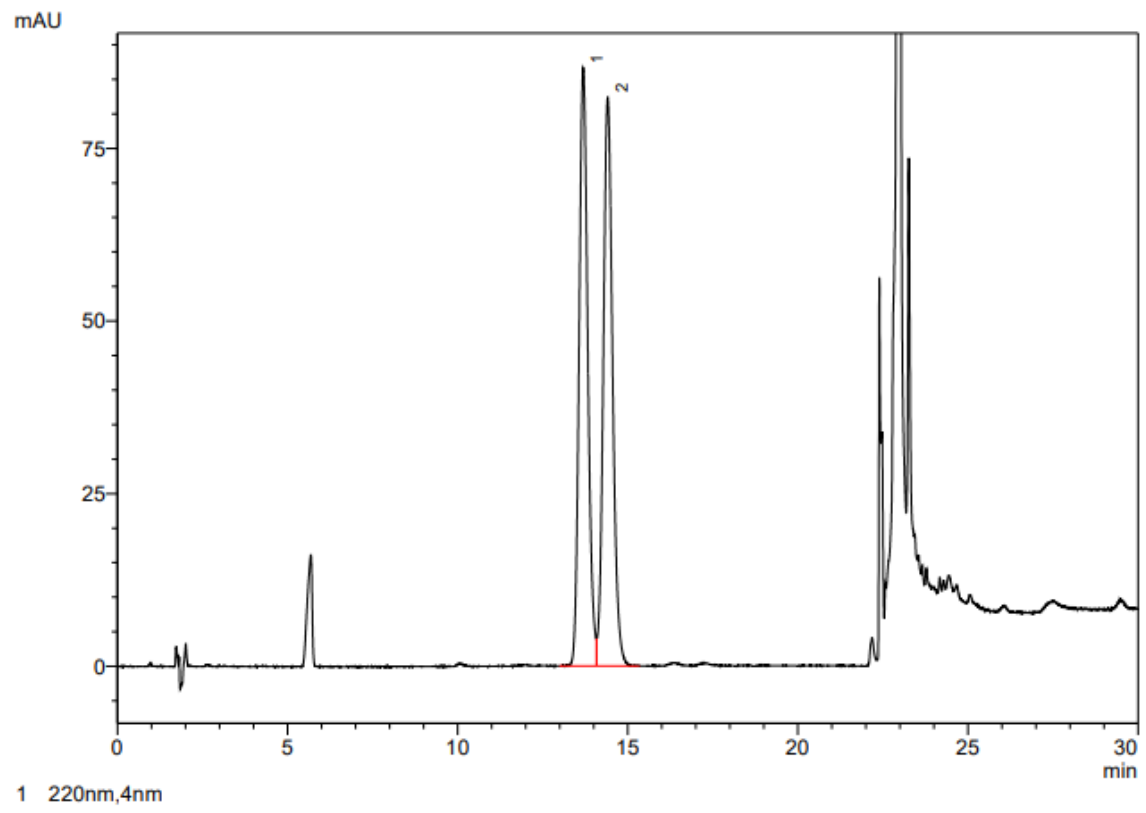

PDA Ch1 220nm

Peak #	Ret. Time	Area %	Name
1	13,69	49,68	1. Enantiomer
2	14,41	50,32	2. Enantiomer
Total		100,00	

Determination of the Relative and Absolute Configuration

The stereostructure of product **8j** determined by X-ray diffraction analysis served as point of reference. The relative configuration of this compound is also clearly reflected in characteristic NOEs as observed by NMR. Moreover, the $^3J_{HH}$ coupling constants of the H-atoms at the 2 stereocenters show a coupling constant of $^3J_{HH} \approx 2.7$ Hz in the ^{1}H NMR, which confirms a *gauche*-orientation, which is again in line with the structure in the solid state.

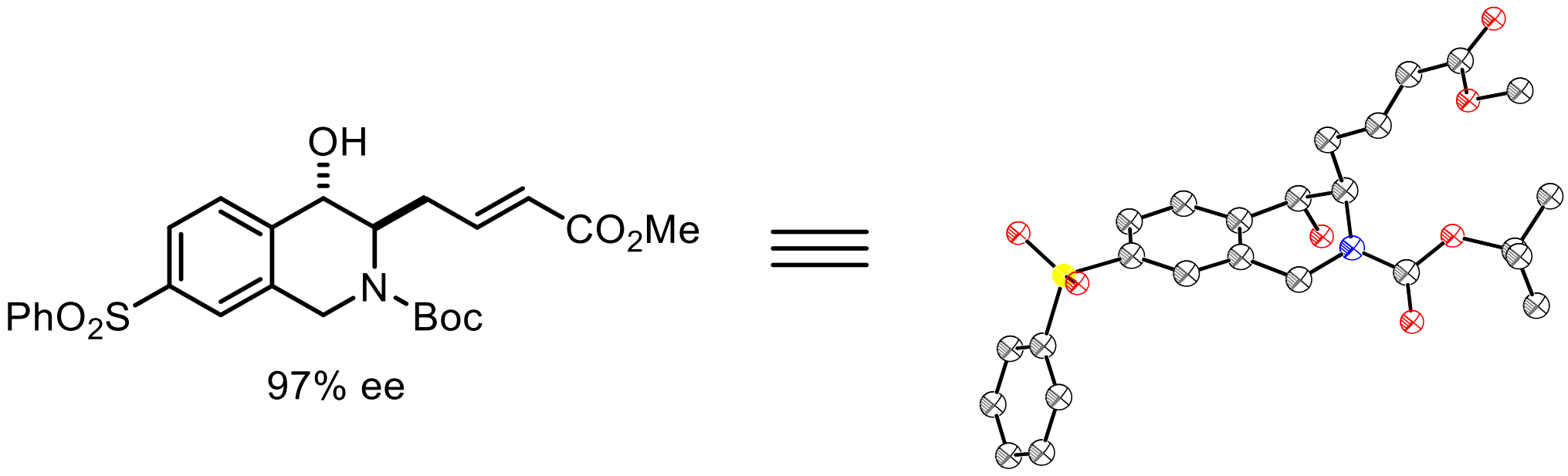

All products such as **8a** showing the same pattern and data were hence assigned in analogy to **8j**.

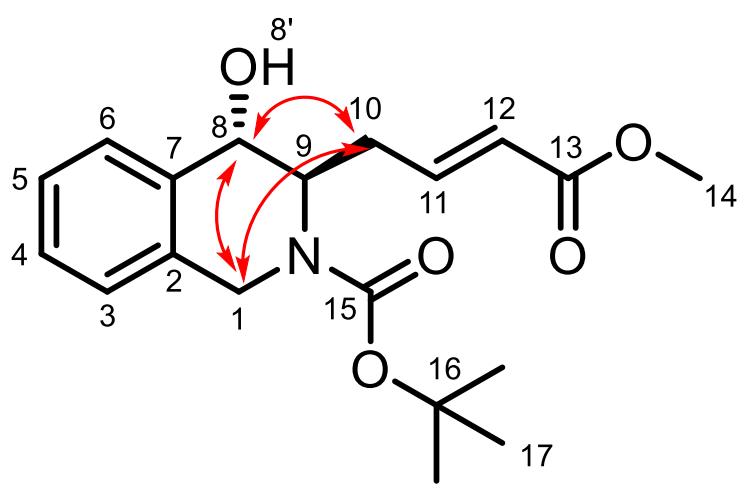

$^3J_{H8,H9}$ = 2.7 Hz

In the cases compiled below, the relative and absolute configuration was apparently different.

NOESY Data and Mosher Ester Analysis of 10

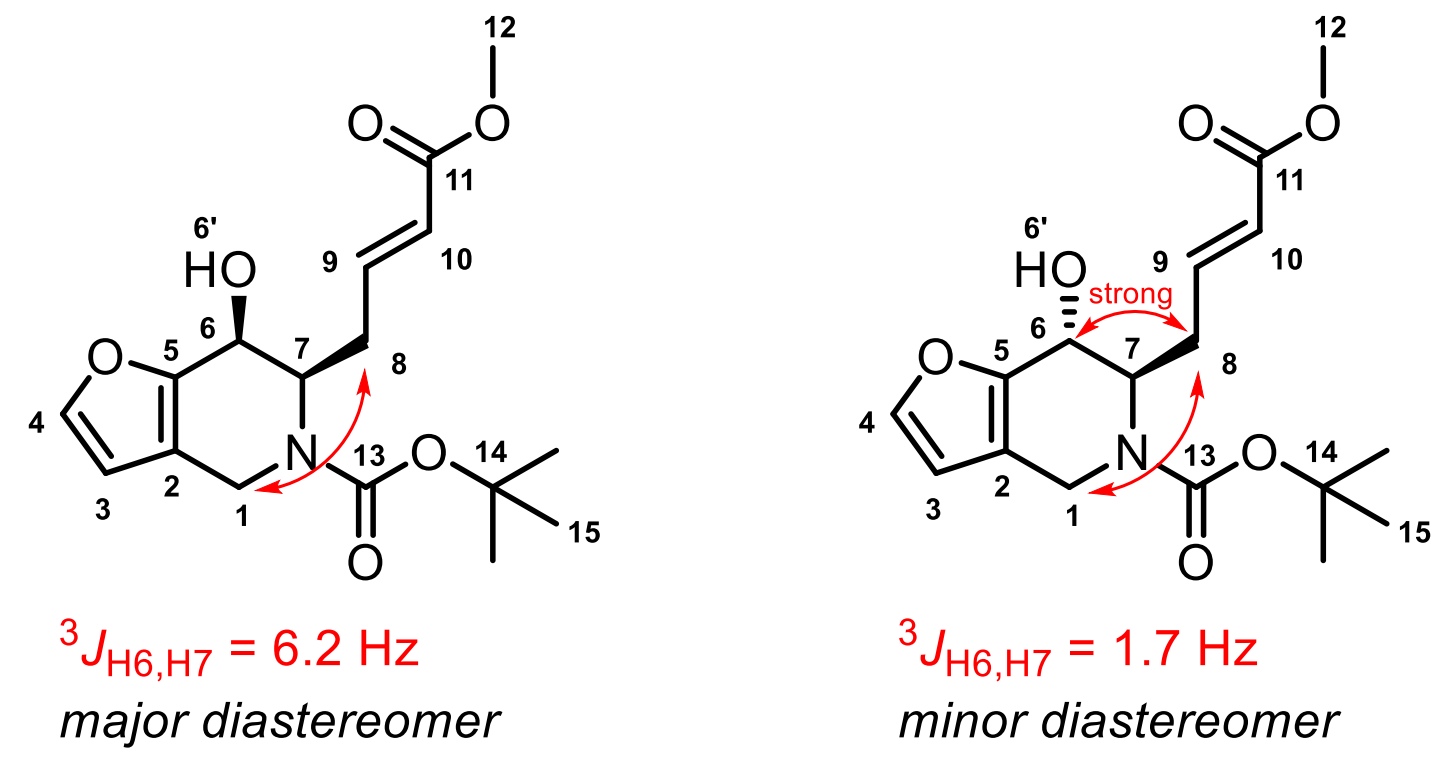

In order to verify the relative configuration of **10**, the NMR data of the major and the minor diastereomer were compared. The minor *trans*-diastereomer shows a strong NOE between H6 and H8'/H8'', while the major diastereomer does not. Moreover, the $^{3}J_{H6,H7}$ coupling constant of the minor diastereomer was 1.7 Hz, which speaks for a *gauche*-orientation of the pertinent protons. The major diastereomer on the other hand showed a $^{3}J_{H6,H7}$ = 6.2 Hz, which corroborates the proposed *cis*-configuration.

The Mosher ester analysis showed that the C6 stereocenter of the major isomer bearing the alcohol group is (*S*)-configured and hence inverted compared to that in the tetrahydroisoquinoline derivative **8j**. The configuration of the second stereocenter of this compound could be inferred as a corollary.

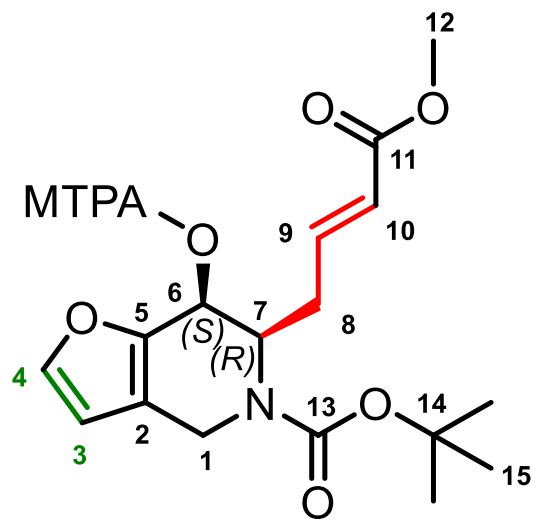

Table S7. Analysis of the Mosher esters according to Hoye and co-workers.[9],[10]

Atom number	(*S*)-MTPA-Ester δ (ppm)	(*R*)-MTPA-Ester δ (ppm)	δ*S* – δ*R* (ppm)
**1'**	3.94	3.95	-0.01
**1''**	4.59	4.60	-0.01
**3**	6.48	6.46	+0.02
**4**	7.67	7.62	+0.05
**6**	6.23	6.22	+0.01
**7**	4.92	5.02	-0.10
**8'**	2.25	2.37	-0.12
**8''**	1.98	2.32	-0.34
**9**	6.69	6.79	-0.10
**10**	5.85	5.94	-0.09

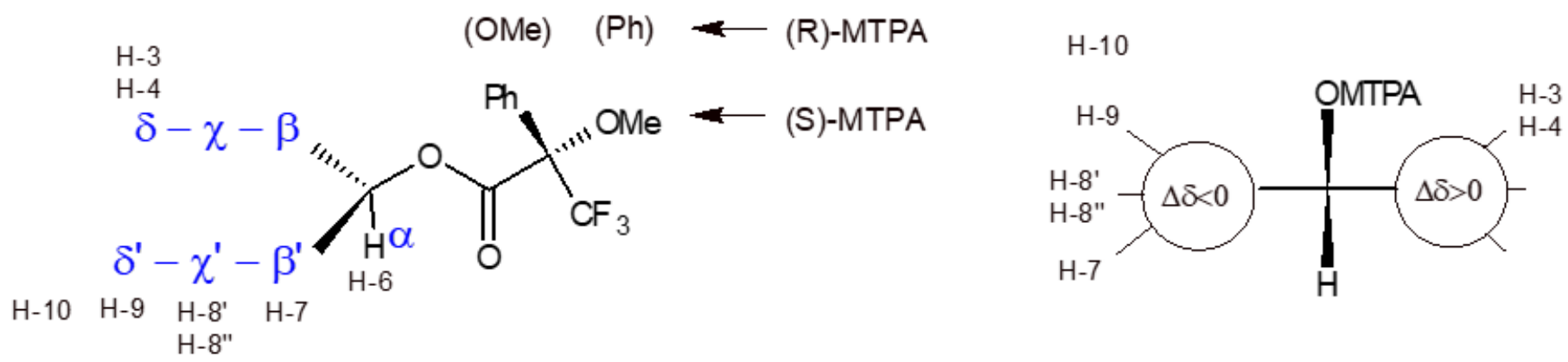

NOESY Data and Mosher Ester Analysis of 12a

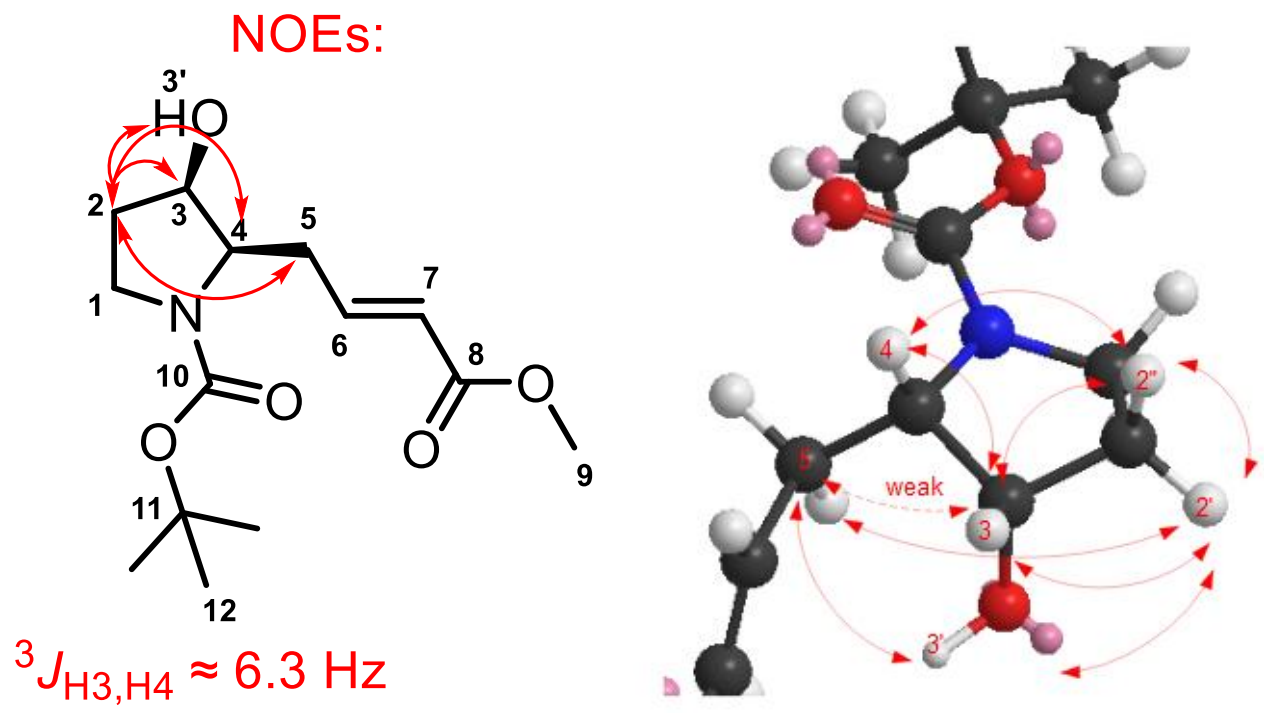

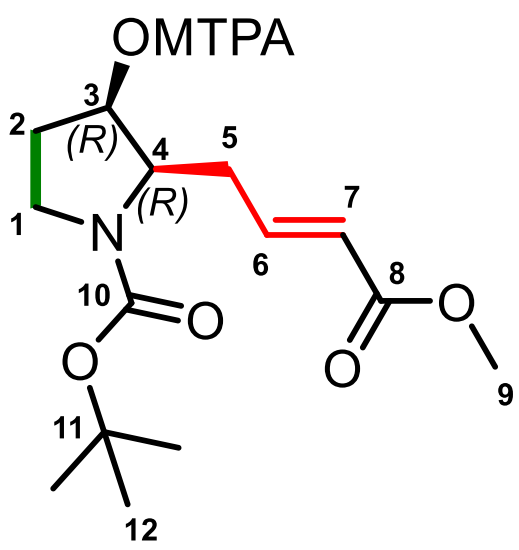

Table S8. Analysis of the Mosher esters according to Hoye and co-workers.[9]

Atom number	(*S*)-MTPA-Ester δ (ppm)	(*R*)-MTPA-Ester δ (ppm)	δ*S* – δ*R* (ppm)
**1'**	3.25	3.14	+0.11
**1''**	3.50	3.46	+0.04
**2'**	1.95	1.84	+0.11
**2''**	2.28	2.26	+0.02
**3**	5.50	5.50	0
**4**	4.13	4.19	-0.06
**5'**	2.24	2.39	-0.15
**5''**	2.38	2.51	-0.13
**6**	6.70	6.79	-0.09
**7**	5.68	5.77	-0.09

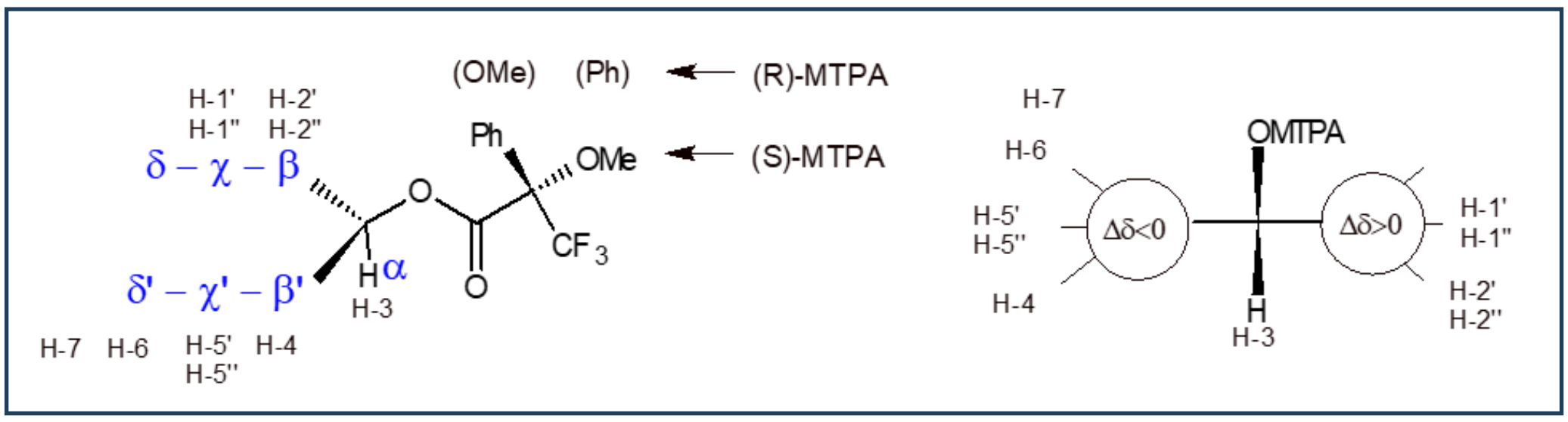

The stereocenter bearing the alcohol group was assigned as (*R)* by the Mosher ester analysis.[9],[11] Due to the *cis*-configuration, the absolute configuration on the second stereocenter could be assigned as (*R*).

NOESY Data and Mosher Ester Analysis of 11

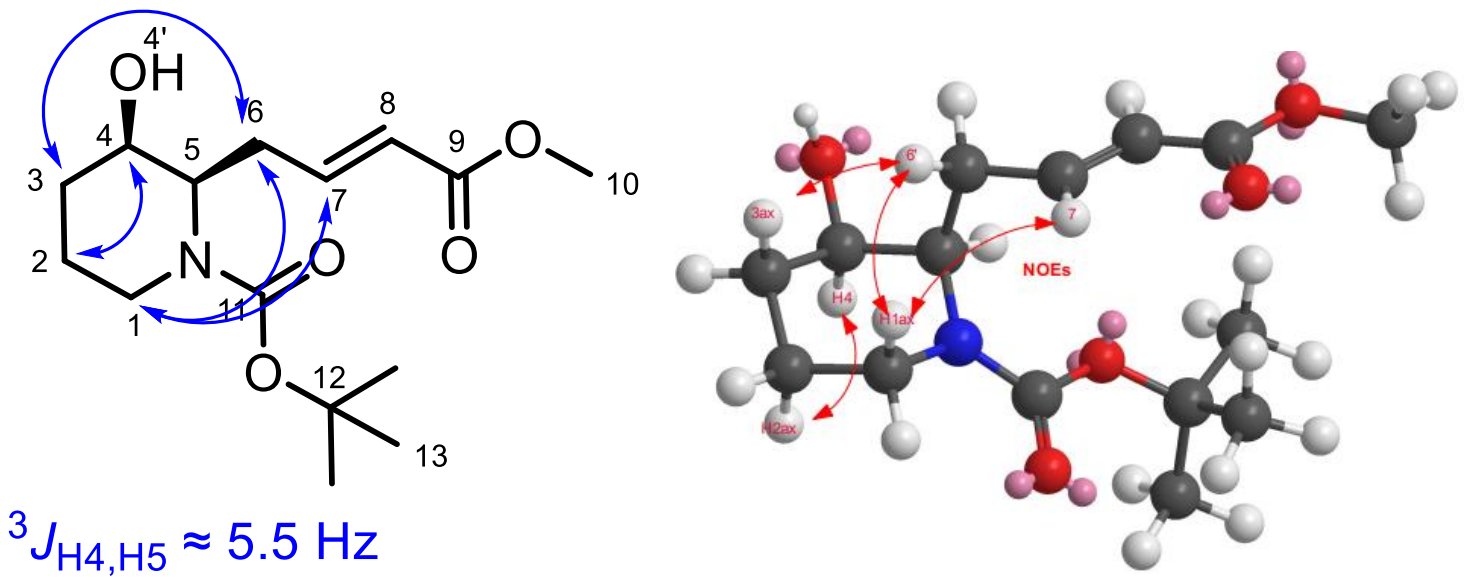

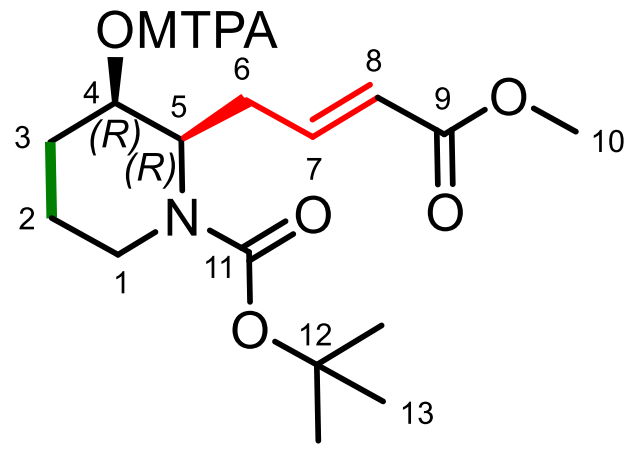

Table S9. Analysis of the Mosher esters according to Hoye and co-workers.[9]

Atom number	(*S*)-MTPA-Ester δ (ppm)	(*R*)-MTPA-Ester δ (ppm)	δ*S* – δ*R* (ppm)
**1'**	2.76	2.78	-0.02
**1''**	3.80	3.80	0
**2'**	1.51	1.50	0.01
**2''**	1.72	1.69	0.03
**3'**	1.76	1.65	0.11
**3''**	1.90	1.83	0.07
**4**	5.04	5.04	0
**5'**	4.49	4.58	-0.09
**6'**	2.64	2.71	-0.07
**6''**	1.87	2.19	-0.32
**7**	6.63	6.74	-0.11
**8**	5.78	5.87	-0.09

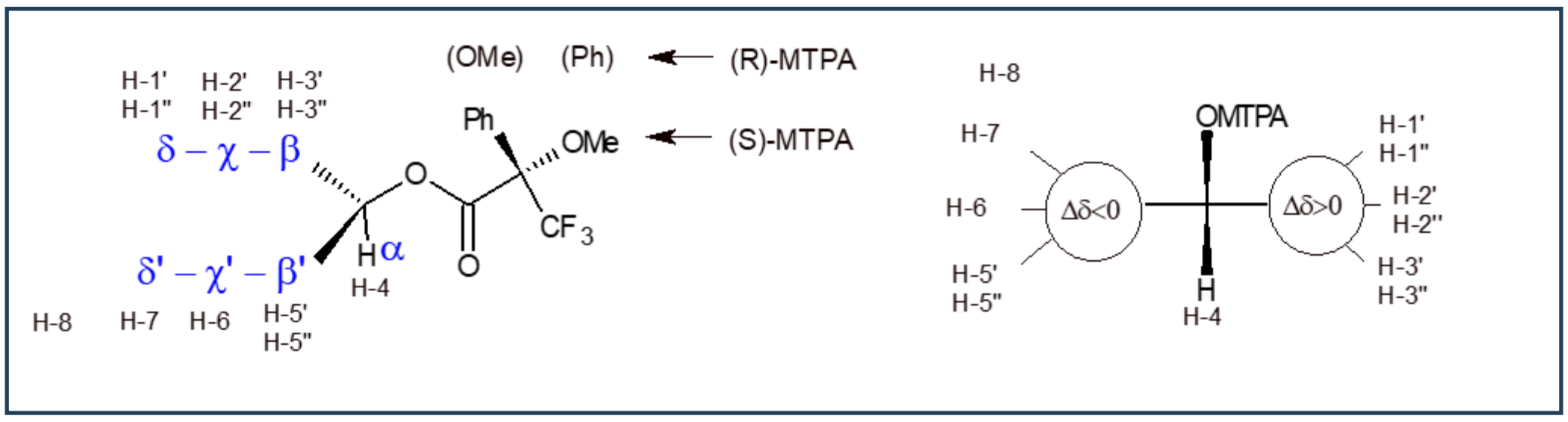

The stereocenter bearing the alcohol group was assigned as (*R)* by the Mosher ester analysis.[9],[11] Due to the *cis*-configuration, the absolute configuration on the second stereocenter could be assigned as (*R*).

The Mosher Ester Derivatives

tert-Butyl (6R,7S)-6-((E)-4-methoxy-4-oxobut-2-en-1-yl)-7-(((S)-3,3,3-trifluoro-2-methoxy-2-phenylpropanoyl)oxy)-6,7-dihydrofuro[3,2-c]pyridine-5(4H)-carboxylate S38

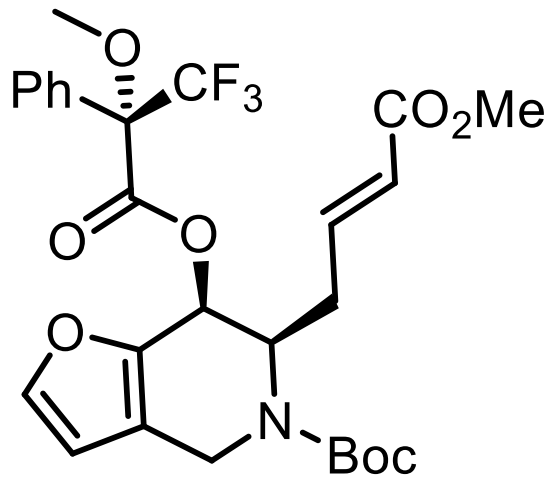

(*R*)-Mosher's acid chloride (4.3 µL, 22.8 µmol), DMAP (0.5 mg, 4.1 µmol) and triethylamine (8.7 µL, 62.2 µmol) were added to a solution of **10** (7.0 mg, 20.7 µmol) in dichloromethane (0.5 mL) and the resulting mixture was stirred at room temperature for 2 h. Dichloromethane (2 mL) and sat. aq. NH_4Cl (2 mL) were added and the resulting mixture was vigorously stirred for 5 min. The organic layer was separated and the aqueous layer was extracted with dichloromethane (2 x 5 mL). The combined organic phases were dried over $MgSO_4$ and filtered, and the solvent was removed under reduced pressure. The residue was purified by flash chromatography (SiO_2, *n*-pentane/ethyl acetate, 4:1) to give the title compound as a colorless oil (5.0 mg, 44%). ^1H NMR (600 MHz, [D_6]-DMSO, 353K): δ = 7.67 (dd, *J* = 1.9, 0.9 Hz, 1H), 7.60 – 7.57 (m, 2H), 7.52 – 7.49 (m, 3H), 6.69 (ddd, *J* = 15.6, 8.6, 5.9 Hz, 1H), 6.48 (d, *J* = 1.9 Hz, 1H), 6.25 – 6.20 (m, 1H), 5.85 (ddd, *J* = 15.6, 1.8, 1.1 Hz, 1H), 4.92 (ddd, *J* = 11.6, 6.4, 3.5 Hz, 1H), 4.59 (dd, *J* = 16.8, 1.9 Hz, 1H), 3.94 (dd, *J* = 16.8, 2.4 Hz, 1H), 3.64 (s, 3H), 3.61 (q, *J* = 1.0 Hz, 3H), 2.25 (dddd, *J* = 15.0, 11.6, 8.6, 1.1 Hz, 1H), 1.98 (dddd, *J* = 15.0, 5.9, 3.6, 1.8 Hz, 1H), 1.41 (s, 9H); IR (ATR): ṽ = 2952, 1756, 1725, 1698, 1396, 1227, 1169, 1122, 1021, 720 cm^{-1}; HRMS (ESI$^+$): *m/z*: calcd. for $C_{27}H_{30}NO_8F_3Na$ $[M+Na]^+$: 576.18157, found: 576.18233.

tert-Butyl (6R,7S)-6-((E)-4-methoxy-4-oxobut-2-en-1-yl)-7-(((R)-3,3,3-trifluoro-2-methoxy-2-phenylpropanoyl)oxy)-6,7-dihydrofuro[3,2-c]pyridine-5(4H)-carboxylate S39

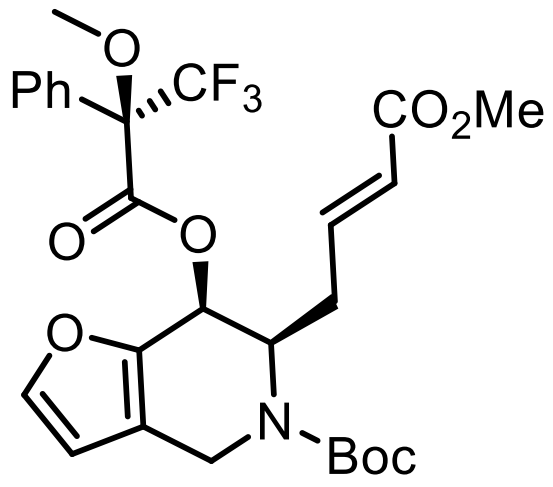

(*S*)-Mosher's acid chloride (3.6 µL, 19.2 µmol), DMAP (0.5 mg, 4.1 µmol) and triethylamine (6.2 µL, 44.4 µmol) were added to a solution of **10** (5.0 mg, 14.8 µmol) in dichloromethane (0.5 mL) and the resulting mixture was stirred at room temperature for 2 h. Dichloromethane (2 mL) and sat. aq. NH_4Cl (2 mL) were added and the resulting mixture was vigorously stirred for 5 min. The organic layer was separated and the aqueous layer was extracted with dichloromethane (2 x 5 mL). The combined organic phases were dried over $MgSO_4$ and filtered, and the solvent was removed under reduced pressure. The residue was purified by flash chromatography (SiO_2, *n*-pentane/ethyl acetate, 4:1) to give the title compound as a colorless oil (4.8 mg, 59%). ^1H NMR (600 MHz, [D_6]-DMSO, 353K): δ = 7.62 (dd, *J* = 1.9, 0.9 Hz, 1H), 7.59 – 7.56 (m, 2H), 7.52 – 7.49 (m, 3H), 6.79 (ddd, *J* = 15.6, 8.5, 6.0 Hz, 1H), 6.46 (d, *J* = 1.9 Hz, 1H), 6.22 (dm, *J* = 6.3 Hz, 1H), 5.94 (ddd, *J* = 15.7, 1.7, 1.2 Hz, 1H), 5.02 (dt, *J* = 10.7, 6.3 Hz, 1H), 4.60 (dd, *J* = 16.7, 1.6 Hz, 1H), 3.95 (dd, *J* = 16.7, 2.3 Hz, 1H), 3.64 (s, 3H), 3.53 (q, *J* = 1.0 Hz, 3H), 2.37 (dddd, *J* = 15.2, 10.7, 8.5, 1.2 Hz, 1H), 2.32 (dddd, *J* = 15.3, 6.0, 4.3, 1.7 Hz, 1H), 1.42 (s, 9H); IR (ATR): ṽ = 2951, 1756, 1726, 1699, 1396, 1228, 1170, 1018, 720 cm^{-1}; HRMS (ESI$^+$): *m/z*: calcd. for $C_{27}H_{30}NO_8F_3Na$ $[M+Na]^+$: 576.18157, found: 576.18179.

tert-Butyl (2R,3R)-2-((E)-4-methoxy-4-oxobut-2-en-1-yl)-3-(((S)-3,3,3-trifluoro-2-methoxy-2-phenyl-propanoyl)oxy)pyrrolidine-1-carboxylate S40

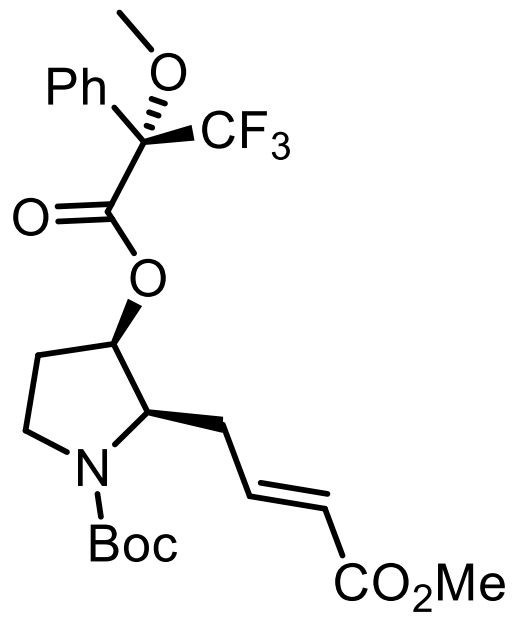

(*R*)-Mosher's acid chloride (5.4 µL, 28.9 µmol), DMAP (0.5 mg, 4.1 µmol) and triethylamine (8.1 µL, 57.8 µmol) were added to a solution of **12a** (5.5 mg, 19.3 µmol) in dichloromethane (0.5 mL) and the resulting mixture was stirred at room temperature for 18 h. Dichloromethane (2 mL) and sat. aq. NH_4Cl (2 mL) were added and the resulting mixture was vigorously stirred for 5 min. The organic layer was separated and the aqueous layer was extracted with dichloromethane (2 x 5 mL). The combined organic phases were dried over $MgSO_4$ and filtered, and the solvent was removed under reduced pressure. The residue was purified by flash chromatography (SiO_2, *n*-pentane/ethyl acetate, 3:1) to give the title compound as a colorless oil (6.5 mg, 67%). ^1H NMR (600 MHz, [D_6]-DMSO, 353K):

δ = 7.50 – 7.46 (m, 5H), 6.70 (dt, *J* = 15.7, 7.2 Hz, 1H), 5.68 (dt, *J* = 15.7, 1.5 Hz, 1H), 5.50 (q, *J* = 6.2 Hz, 1H), 4.13 (q, *J* = 6.3 Hz, 1H), 3.64 (s, 3H), 3.52 (q, *J* = 1.1 Hz, 3H), 3.50 (ddd, *J* = 10.8, 8.2, 5.9 Hz, 1H), 3.25 (ddd, *J* = 10.9, 7.9, 6.6 Hz, 1H), 2.38 (dt, *J* = 15.0, 6.6 Hz, 1H), 2.28 (ddt, *J* = 13.2, 8.2, 6.6 Hz, 1H), 2.23 (dtd, *J* = 15.0, 7.2, 1.5 Hz, 1H), 1.95 (ddt, *J* = 13.8, 7.9, 6.0 Hz, 1H), 1.40 (s, 9H); IR (ATR): ṽ = 2979, 1750, 1724, 1696, 1394, 1257, 1167, 1122, 1023, 720 cm^{-1}; HRMS (ESI^+): *m/z*: calcd. for $C_{24}H_{30}NO_7F_3Na$ $[M+Na]^+$: 524.18666, found: 524.18636.

***tert*-Butyl (2*R*,3*R*)-2-((*E*)-4-methoxy-4-oxobut-2-en-1-yl)-3-(((*R*)-3,3,3-trifluoro-2-methoxy-2-phenyl-propanoyl)oxy)pyrrolidine-1-carboxylate S41**

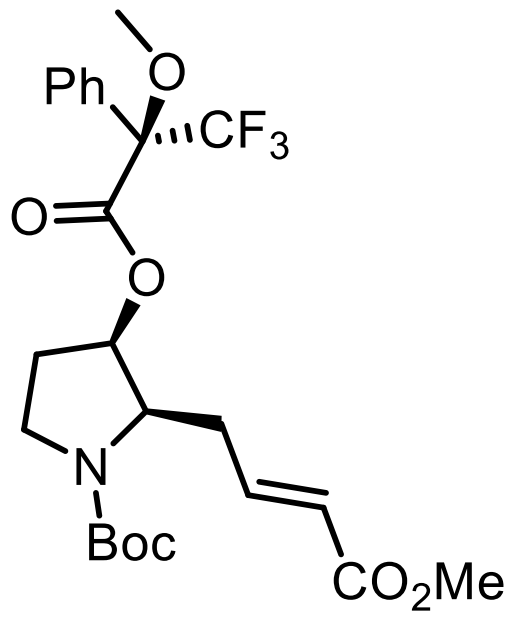

(*S*)-Mosher's acid chloride (7.2 µL, 38.6 µmol), DMAP (0.5 mg, 4.1 µmol) and triethylamine (8.1 µL, 57.8 µmol) were added to a solution of **12a** (5.5 mg, 19.3 µmol) in dichloromethane (0.5 mL) and the resulting mixture was stirred at room temperature for 18 h. Dichloromethane (2 mL) and sat. aq. NH_4Cl (2 mL) were added and the resulting mixture was vigorously stirred for 5 min. The organic layer was separated and the aqueous layer was extracted with dichloromethane (2 x 5 mL). The combined organic phases were dried over $MgSO_4$ and filtered, and the solvent was removed under reduced pressure. The residue was purified by flash chromatography (SiO_2, *n*-pentane/ethyl acetate, 3:1) to give the title compound as a colorless oil (6.6 mg, 68%). ^1H NMR (600 MHz, [D_6]-DMSO, 353K): δ = 7.51 – 7.46 (m, 5H), 6.79 (dt, *J* = 15.7, 7.2 Hz, 1H), 5.77 (dt, *J* = 15.7, 1.5 Hz, 1H), 5.50 (q, *J* = 6.2 Hz, 1H), 4.18 (q, *J* = 6.2 Hz, 1H), 3.65 (s, 3H), 3.47 (q, *J* = 1.1 Hz, 3H), 3.46 (ddd, *J* = 10.8, 8.2, 5.9 Hz, 1H), 3.13 (dt, *J* = 10.8, 7.8 Hz, 1H), 2.51 (dt, *J* = 15.0, 6.6 Hz, 1H), 2.39 (dtd, *J* = 15.0, 7.1, 1.5 Hz, 1H), 2.26 (ddt, *J* = 13.2, 8.2, 6.6 Hz, 1H), 1.84 (ddt, *J* = 13.2, 7.8, 6.2 Hz, 1H), 1.40 (s, 9H); IR (ATR): ṽ = 2979, 1751, 1723, 1695, 1395, 1255, 1168, 1121, 1043, 762 cm^{-1}; HRMS (ESI^+): *m/z*: calcd. for $C_{24}H_{30}NO_7F_3Na$ $[M+Na]^+$: 524.18666, found: 524.18642.

***tert*-Butyl (2*R*,3*R*)-2-((*E*)-4-methoxy-4-oxobut-2-en-1-yl)-3-(((*S*)-3,3,3-trifluoro-2-methoxy-2-phenyl-propanoyl)oxy)piperidine-1-carboxylate S42**

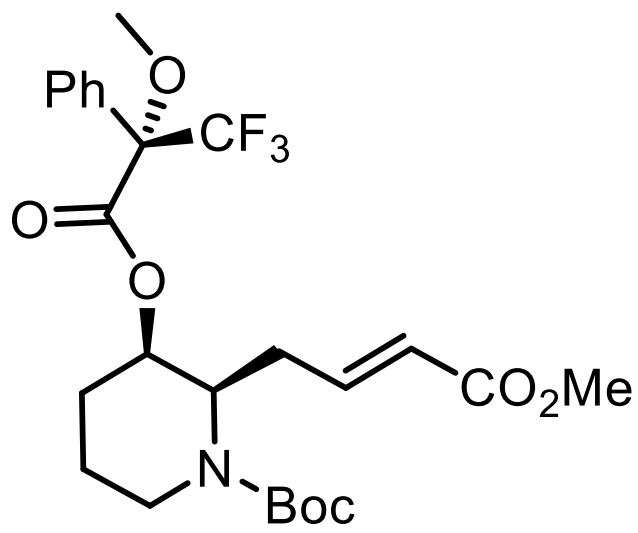

(*R*)-Mosher's acid chloride (4.8 µL, 25.4 µmol), DMAP (0.3 mg, 2.5 µmol) and triethylamine (5.3 µL, 38.1 µmol) were added to a solution of **11** (3.8 mg, 12.7 µmol) in dichloromethane (0.5 mL) and the resulting mixture was stirred at room temperature for 18 h. Dichloromethane (2 mL) and sat. aq. NH_4Cl (2 mL) were added and the resulting mixture was vigorously stirred for 5 min. The organic layer was separated and the aqueous layer was extracted with dichloromethane (2 x 5 mL). The combined organic phases were dried over $MgSO_4$ and filtered, and the solvent was removed under reduced pressure. The residue was purified by flash chromatography (SiO_2, toluene/*tert*-butyl methyl ether, 10:1) to give the title compound as a colorless oil (4.2 mg, 64%). ^1H NMR (600 MHz, [D_6]-DMSO, 353K): δ = 7.52 – 7.47 (m, 5H), 6.63 (ddd, *J* = 15.5, 8.5, 6.2 Hz, 1H), 5.78 (dt, *J* = 15.5, 1.4 Hz, 1H), 5.04 (ddd, *J* = 12.0, 5.5, 4.6 Hz, 1H), 4.49 (ddd, *J* = 12.0, 5.5, 3.7 Hz, 1H), 3.80 (dd, *J* = 13.7, 5.0 Hz, 1H), 3.63 (s, 3H), 3.53 (q, *J* = 1.0 Hz, 3H), 2.76 (td, *J* = 13.3, 2.9 Hz, 1H), 2.64 (dddd, *J* = 14.9, 11.3, 8.5, 1.3 Hz, 1H), 1.93 – 1.83 (m, 2H), 1.81 – 1.68 (m, 2H), 1.51 (qt, *J* = 13.1, 4.5 Hz, 1H), 1.39 (s, 9H); IR (ATR): ṽ = 2925, 1750, 1725, 1693, 1411, 1243, 1156, 1020, 722 cm^{-1}; HRMS (ESI^+): *m/z*: calcd. for $C_{25}H_{32}NO_7F_3Na$ $[M+Na]^+$: 538.20231, found: 538.20272.

tert-Butyl (2R,3R)-2-((E)-4-methoxy-4-oxobut-2-en-1-yl)-3-(((R)-3,3,3-trifluoro-2-methoxy-2-phenyl-propanoyl)oxy)piperidine-1-carboxylate S42

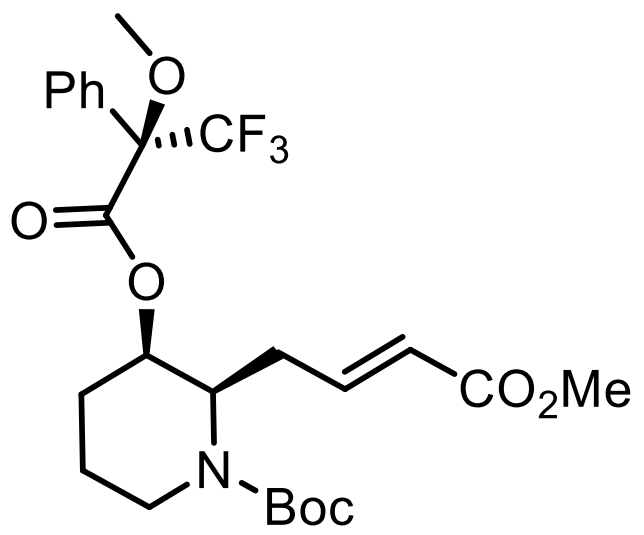

(*S*)-Mosher's acid chloride (4.8 µL, 25.4 µmol), DMAP (0.3 mg, 2.5 µmol) and triethylamine (5.3 µL, 38.1 µmol) were added to a solution of **11** (3.8 mg, 12.7 µmol) in dichloromethane (0.5 mL) and the resulting mixture was stirred at room temperature for 18 h. Dichloromethane (2 mL) and sat. aq. NH_4Cl (2 mL) were added and the resulting mixture was vigorously stirred for 5 min. The organic layer was separated and the aqueous layer was extracted with dichloromethane (2 x 5 mL). The combined organic phases were dried over $MgSO_4$ and filtered, and the solvent was removed under reduced pressure. The residue was purified by flash chromatography (SiO_2, toluene/*tert*-butyl methyl ether, 10:1) to give the title compound as a colorless oil (4.5 mg, 69%). ^{1}H NMR (600 MHz, [D_6]-DMSO, 353K): δ = 7.49 (s, 5H), 6.74 (ddd, *J* = 15.6, 8.5, 6.2 Hz, 1H), 5.87 (dt, *J* = 15.6, 1.4 Hz, 1H), 5.04 (ddd, *J* = 11.6, 5.5, 4.5 Hz, 1H), 4.58 (ddd, *J* = 11.3, 5.5, 3.8 Hz, 1H), 3.80 (dd, *J* = 13.6, 5.0 Hz, 1H), 3.63 (s, 3H), 3.48 (q, *J* = 1.0 Hz, 3H), 2.77 (td, *J* = 13.6, 2.9 Hz, 1H), 2.71 (dddd, *J* = 14.9, 11.3, 8.5, 1.3 Hz, 1H), 2.19 (dddd, *J* = 14.9, 6.2, 3.8, 1.7 Hz, 1H), 1.87 – 1.80 (m, 1H), 1.73 – 1.61 (m, 2H), 1.54 – 1.46 (m, 1H), 1.39 (s, 9H); IR (ATR): ṽ = 2953, 1750, 1726, 1694, 1412, 1272, 1249, 1158, 1022 cm^{-1}; HRMS (ESI$^+$): *m/z*: calcd. for $C_{25}H_{32}NO_7F_3Na$ $[M+Na]^+$: 538.20231, found: 538.20222.

Nickel-Catalyzed Synthesis of Chromanol Derivatives: Preliminary Ligand Screening

Since the nickel-catalyzed cyclization of substrate **S37** in the presence of (*S*)-AntPhos gave almost racemic chromanol **16b** (dr = 5:1, ee = 2%), a range of achiral and chiral ligands was screened.

The reaction proceeded well in the absence of any ligand, even though the dr proved to be solvent-dependent. Addition of PPh_3 as well as a prototypical NHC ligand largely suppressed the reaction. In the presence of most other ligands, including the chiral ligands shown in Figure S7, mixtures of the desired chromanol **16b** and the head-to-tail dimer [**16b**]$_2$ were formed, which are very difficult to separate by flash chromatography. Moreover, the level of asymmetric induction was poor. Among the ligands tested, only the VAPOL-derived phosphoramidite **L1** delivered a promising result, affording the product with an enantiomeric excess of 40%, although still as a mixture **16b**/[**16b**]$_2$. These preliminary data show that a much more extensive ligand- and condition screening is necessary to establish an asymmetric reductive chromanol synthesis.

Table S10. Selected optimization experiments for the enantioselective intramolecular reductive cyclization of **S37**.

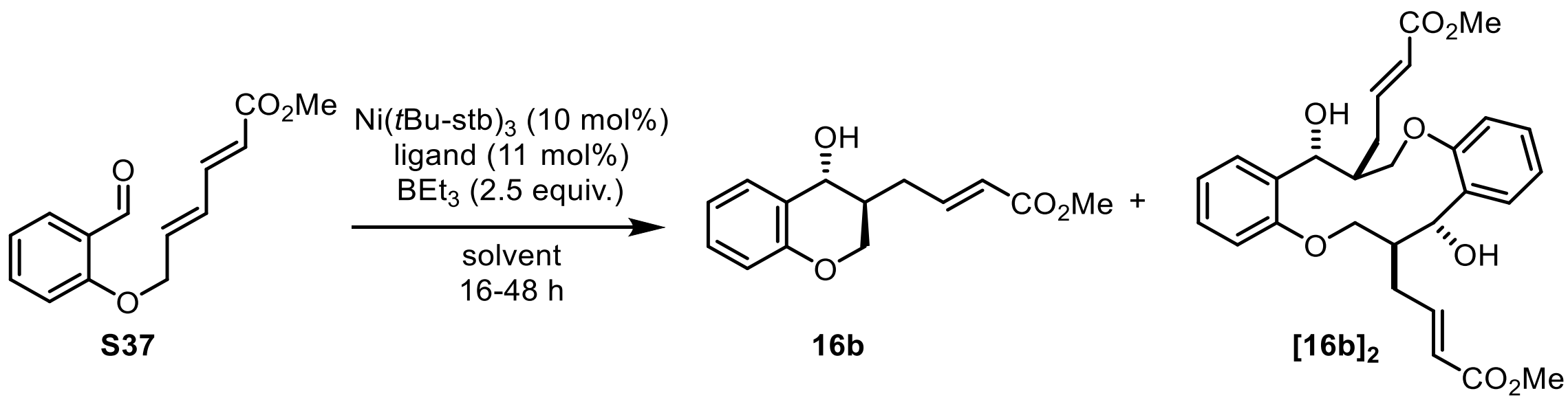

##	Solvent	Ligand	dr	ee	16b/[16b]$_2$	Conversion
1	THF	---	5:1	---	100:0	quant.
2	PhMe	---	2:1	---	100:0	quant.
3	DMA	---	10:1	---	100:0	quant.[a]
4	THF	PPh_3	n.d.	---	n.d.	<10%
5	THF	SIPr·HCl	n.d.	---	n.d.	<10% [a,b]
6	THF	PCy_3	1:2	---	1.3:1	quant.
7	THF	$P(O\text{-}2,4\text{-}t\text{-}Bu_2C_6H_3)_3$	4:1	---	11:1	quant.
8	THF	**L1**	8:1	40%	3:1	quant.
9	THF	**L6**	7:1	2%	19:1	quant.
10	THF	**L12**	8:1	2%	100:0	quant.
11	THF	**L4**	n.d.	n.d.	n.d.	<10%
12	THF	**LS1**	n.d.	n.d.	n.d.	0%
13	THF	**LS2**	n.d.	n.d.	n.d.	0%
14	THF	**LS3**	3:1	13%	6:1	quant.
15	THF	**LS4**	6:1	7%	11:1	quant.
16	THF	(*S*)-AntPhos	5:1	2%	100:0	81%

[a] unknown byproducts observed in crude NMR / messy reaction profile; [b] 1:1 ratio of ligand and LiO^{t}Bu used

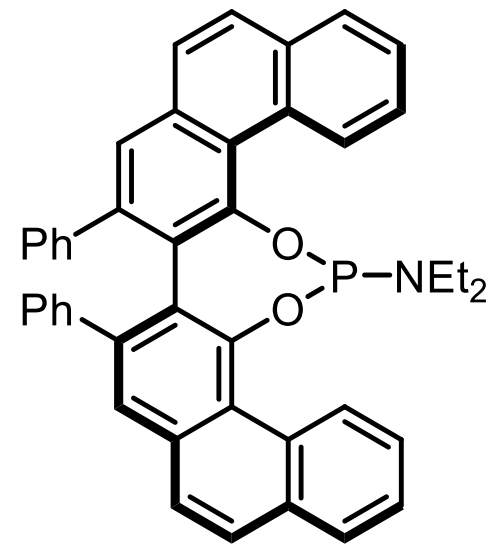

L1

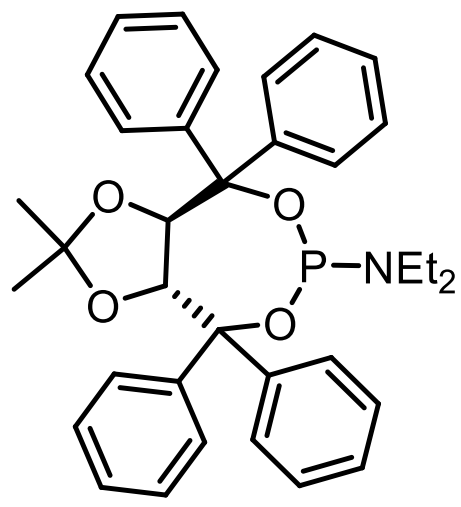

L6

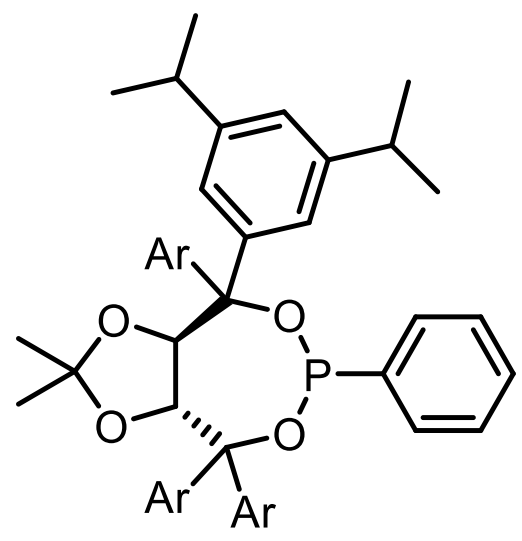

L12

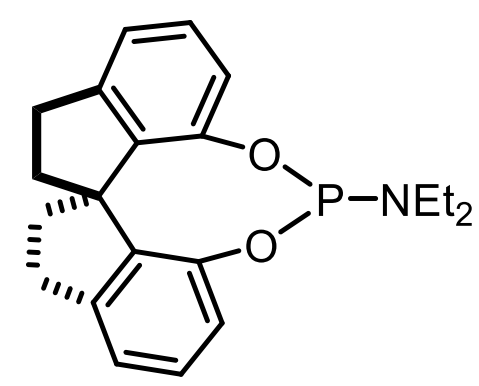

L4

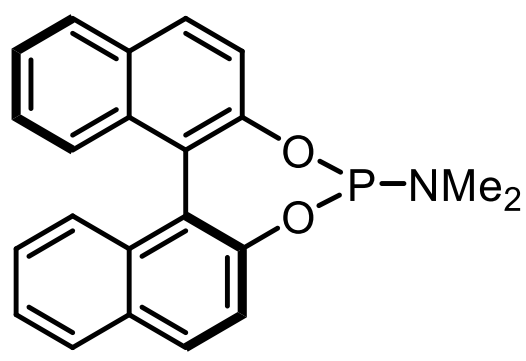

LS1

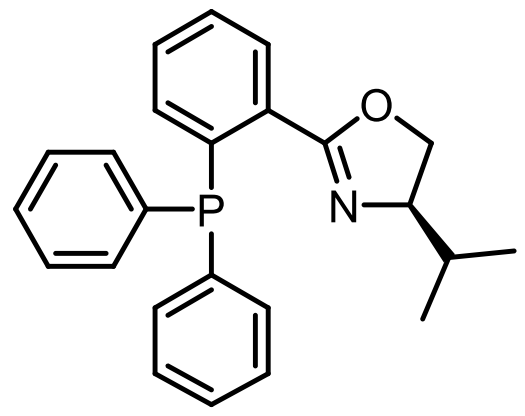

LS2

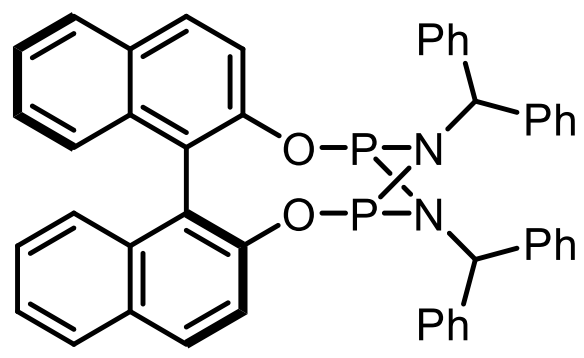

LS3

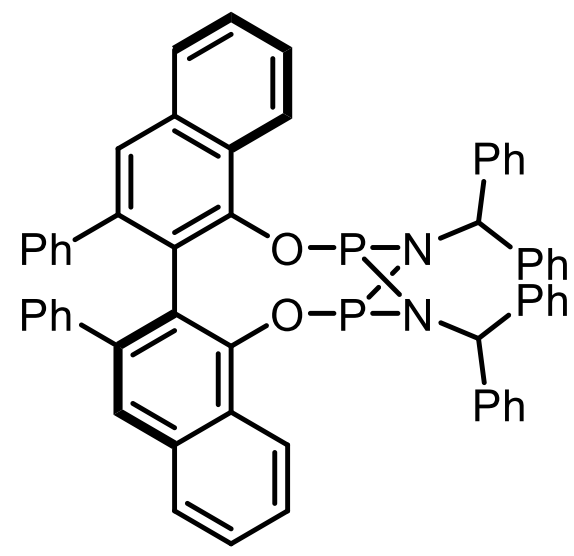

LS4

Figure S7. Chiral ligands tested in the attempted asymmetric reductive cyclization of **S37**.

Applications

tert-Butyl (3*R*,4*S*)-4-fluoro-3-((*E*)-4-methoxy-4-oxobut-2-en-1-yl)-3,4-dihydroisoquinoline-2(1*H*)-carboxylate (17).

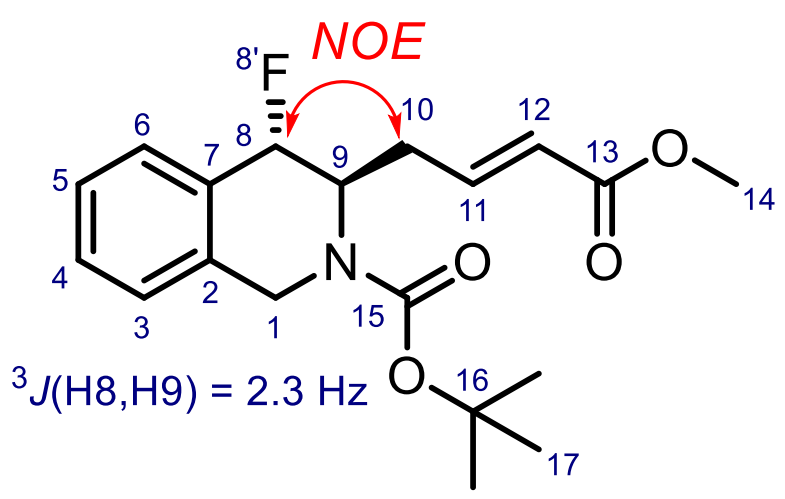

A solution of diethylaminosulfur trifluoride (DAST) (20.0 µL, 0.15 mmol) in dichloromethane (0.1 mL) was added dropwise to a solution of *tert*-butyl (3*R*,4*S*)-4-hydroxy-3-((*E*)-4-methoxy-4-oxobut-2-en-1-yl)-3,4-dihydroisoquinoline-2(1*H*)-carboxylate **8a** (40.0 mg, 0.12 mmol, 92% ee) in dichloromethane (0.3 mL) at –78 °C (bath temperature). After complete addition, the reaction mixture was stirred at this temperature for 2 h. The mixture was warmed to 0 °C before sat. aq. $NaHCO_3$ (0.5 mL) was carefully added. The mixture was vigorously stirred for 5 min, water (5 mL) and dichloromethane (5 mL) were introduced, the organic phase was separated, and the aqueous layer was extracted with dichloromethane (3 x 5 mL). The combined organic phases were washed with brine (10 mL), dried over $MgSO_4$ and filtered, and the solvent was removed under reduced pressure. The residue was purified by flash chromatography (*n*-pentane/ethyl acetate, 5:1) to give the title compound as a colorless oil (25.9 mg, 64%, 90% ee).

The relative configuration was assigned based on NOESY data and the ^3J(H8,H9) coupling constant, which both indicate a *trans*-configuration (reaction under stereoretention, see the Insert).

$[\alpha]_D^{20}$ = +42.0 (c = 0.49, $CHCl_3$); ^{1}H NMR (600 MHz, [D$_6$]-DMSO, 383K): δ = 7.49 – 7.41 (m, 2H), 7.36 – 7.30 (m, 2H), 6.83 (ddd, *J* = 15.6, 8.0, 6.6 Hz, 1H), 5.88 (dt, *J* = 15.6, 1.5 Hz, 1H), 5.48 (dd, *J* = 49.4, 2.3 Hz, 1H), 4.89 (dd, *J* = 17.8, 3.6 Hz, 1H), 4.83 (dddd, *J* = 14.7, 10.0, 5.0, 2.3 Hz, 1H), 4.26 (dd, *J* = 17.8, 5.2 Hz, 1H), 3.66 (s, 3H), 2.34 (dddd, *J* = 14.8, 6.6, 5.0, 1.7 Hz, 1H), 2.10 (dddd, *J* = 14.8, 10.0, 8.0, 1.3 Hz, 1H), 1.46 (s, 9H); ^{13}C NMR (151 MHz, [D$_6$]-DMSO, 383K): δ = 165.0, 153.9, 143.7, 133.3 (d, *J* = 3.3 Hz), 130.3 (d, *J* = 3.4 Hz), 129.3 (d, *J* = 4.2 Hz), 128.1 (d, *J* = 17.6 Hz), 126.4 (d, *J* = 3.3 Hz), 125.8 (d, *J* = 3.0 Hz), 122.5, 87.5 (d, *J* = 170.9 Hz), 79.1, 52.6, 50.3, 41.0, 30.8 (d, *J* = 6.9 Hz), 27.4; ^{19}F NMR (585 MHz, [D$_6$]-DMSO, 383K): δ = –151.5; IR (ATR): ṽ = 2976, 1722, 1689, 1407, 1366, 1326, 1160, 1124, 977, 769, 744 cm^{-1}; HRMS (EI): *m/z*: calcd. for $C_{19}H_{24}NO_4F$ $[M]^{+\cdot}$: 372.15816, found: 372.15829.

The racemic product required for the ee determination was prepared analogously from *rac*-**8a**

The ee was determined by HPLC analysis: Chiralpak 150 mm IG-3, 3 µm, 4.6 mm ∅, acetonitrile/water = 60:40, v = 1.0 mL/min, λ = 210 nm, t(minor) = 4.88 min, t(major) = 5.60 min, (enantioenriched sample: left, racemic sample: right).

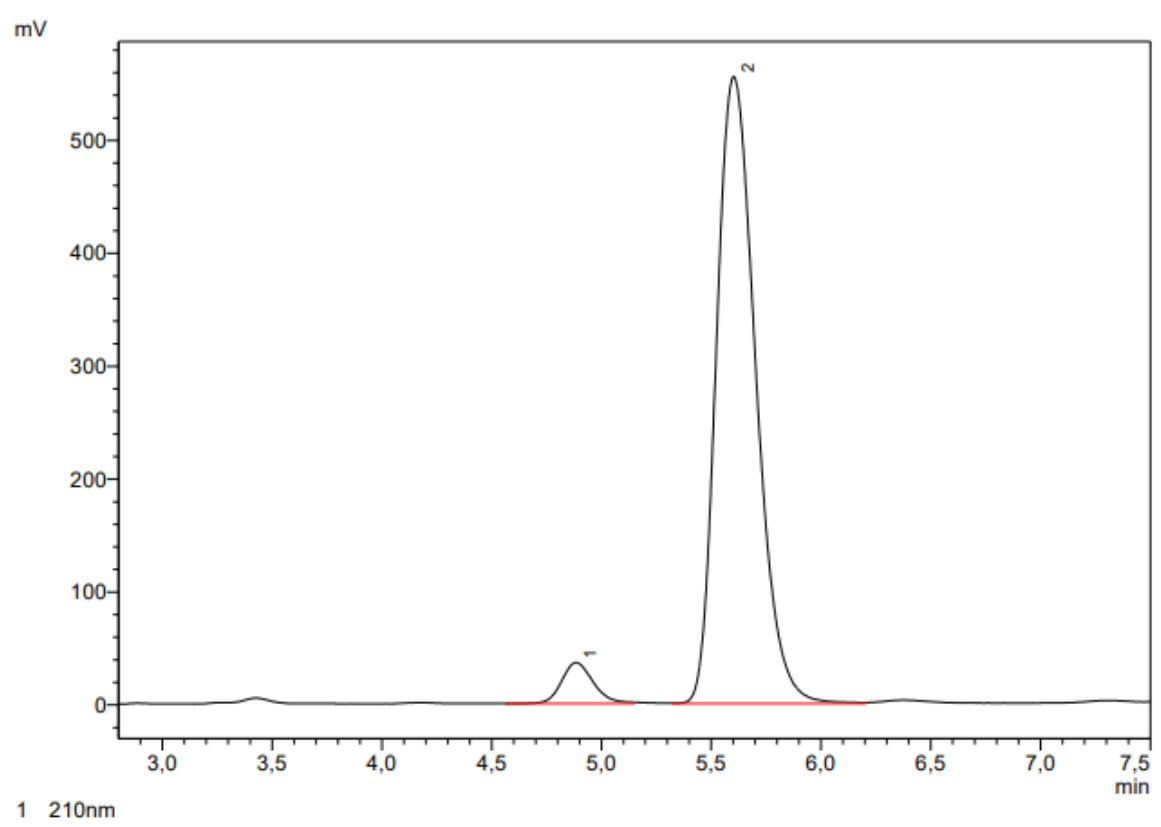

Detector A 210nm

Peak #	Ret. Time	Area %	Name
1	4,88	5,00	1. Enantiomer
2	5,60	95,00	2. Enantiomer 90.0 % ee
Total		100,00	

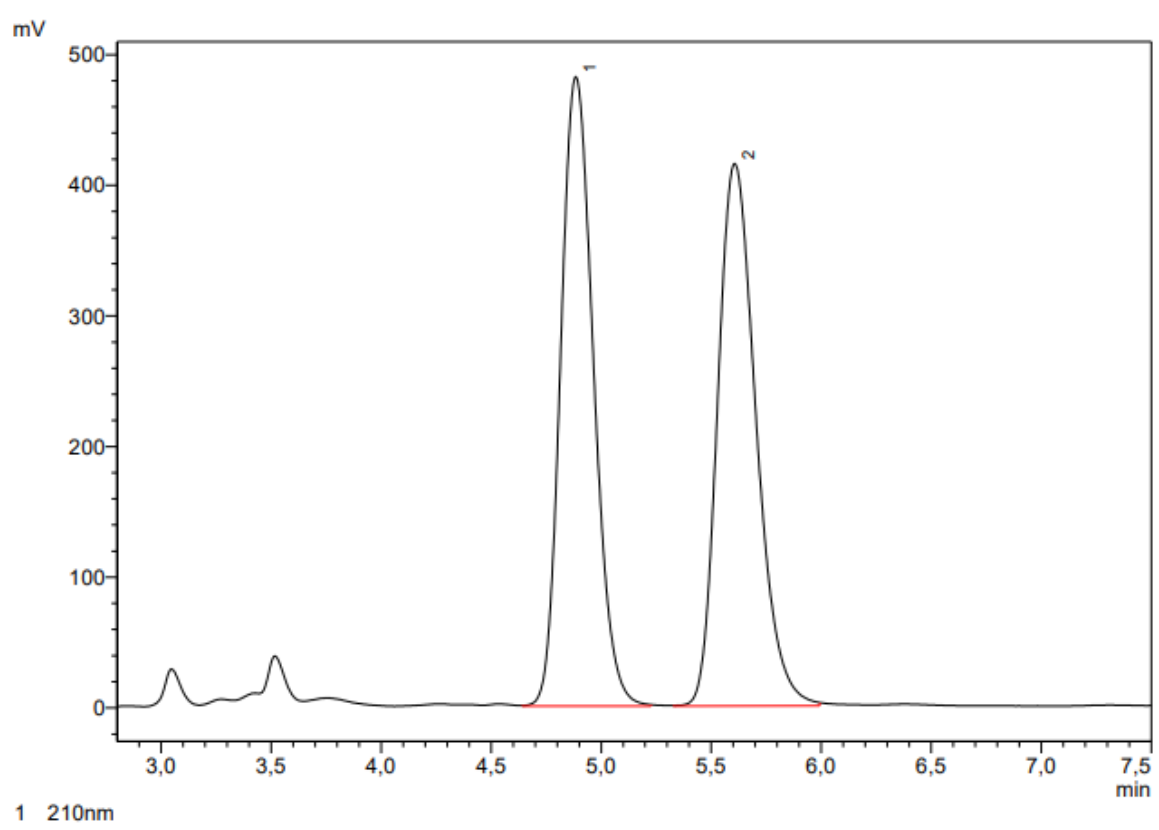

Detector A 210nm

Peak #	Ret. Time	Area %	Name
1	4,88	49,81	1. Enantiomer
2	5,61	50,19	2. Enantiomer
Total		100,00	

tert-Butyl (2_S_,3a_R_,6a_R_)-2-(2-methoxy-2-oxoethyl)tetrahydrospiro[furo[3,2-_b_]pyrrole-6,3'-oxetane]-4(5_H_)-carboxylate (20).

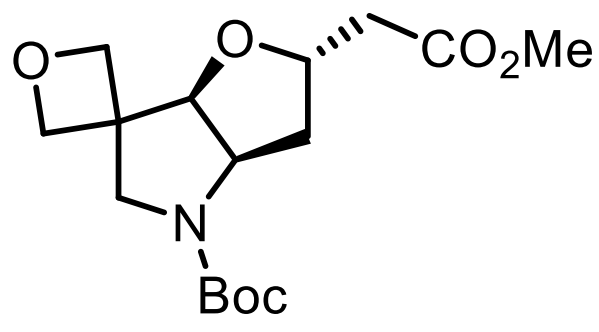

(major isomer)

DBU (2.0 µL, 13.4 µmol) was added to a solution of *tert*-butyl (7*R*,8*R*)-8-hydroxy-7-((*E*)-4-methoxy-4-oxobut-2-en-1-yl)-2-oxa-6-azaspiro[3.4]-octane-6-carboxylate **13** (17.0 mg, 51.9 µmol, 95% ee) in THF (0.5 mL) and the resulting mixture was stirred at room temperature for 16 h. The solvent was evaporated and the residue purified by flash chromatography (SiO_2, *n*-pentane/ethyl acetate, 1:2) to give the title compound as a colorless oil (14.0 mg, 82%, 94% ee, dr ≈ 4:1).

$[\alpha]_D^{20}$ = –56.9 (c = 0.70, $CHCl_3$); ^{1}H NMR (600 MHz, [D$_6$]-DMSO, 373K): δ = 4.70 (d, *J* = 4.8 Hz, 1H), 4.61 (d, *J* = 6.4 Hz, 1H), 4.46 (d, *J* = 6.4 Hz, 1H), 4.41 (d, *J* = 5.7 Hz, 1H), 4.34 (d, *J* = 5.9 Hz, 1H), 4.30 (ddd, *J* = 5.7, 4.8, 1.3 Hz, 1H), 4.15 (dtd, *J* = 9.3, 6.3, 5.6 Hz, 1H), 3.89 (d, *J* = 11.3 Hz, 1H), 3.62 (s, 3H), 3.29 (d, *J* = 11.3 Hz, 1H), 2.61 – 2.51 (m, 2H), 2.27 (ddd, *J* = 13.4, 5.6, 1.3 Hz, 1H), 1.83 (ddd, *J* = 13.4, 9.3, 5.7 Hz, 1H), 1.43 (s, 9H); ^{13}C NMR (151 MHz, [D$_6$]-DMSO, 373K): δ = 170.1, 152.5, 86.0, 79.4, 78.5, 74.9, 72.5, 61.3, 53.1, 50.5, 47.3, 39.0, 38.7, 27.7; IR (ATR): ṽ = 2974, 2872, 1738, 1694, 1398, 1175, 1151, 1069, 905, 730 cm^{-1}; HRMS (ESI$^+$): *m/z*: calcd. for $C_{16}H_{25}NO_6Na$ $[M+Na]^+$: 350.15741, found: 350.15743.

The ee was determined by HPLC analysis: Chiralpak 150 mm IB-N-3, 3 µm, 4.6 mm ∅, *n*-heptane/*iso*-propanol = 95:5, v = 1.0 mL/min, λ = 205 nm, **for major diastereomer:** t(major) = 9.57 min, t(minor) = 12.57 min, **for minor diastereomer:** t(major) = 11.18 min, t(minor) = 13.86 min, (enantioenriched sample: left, racemic sample: right).

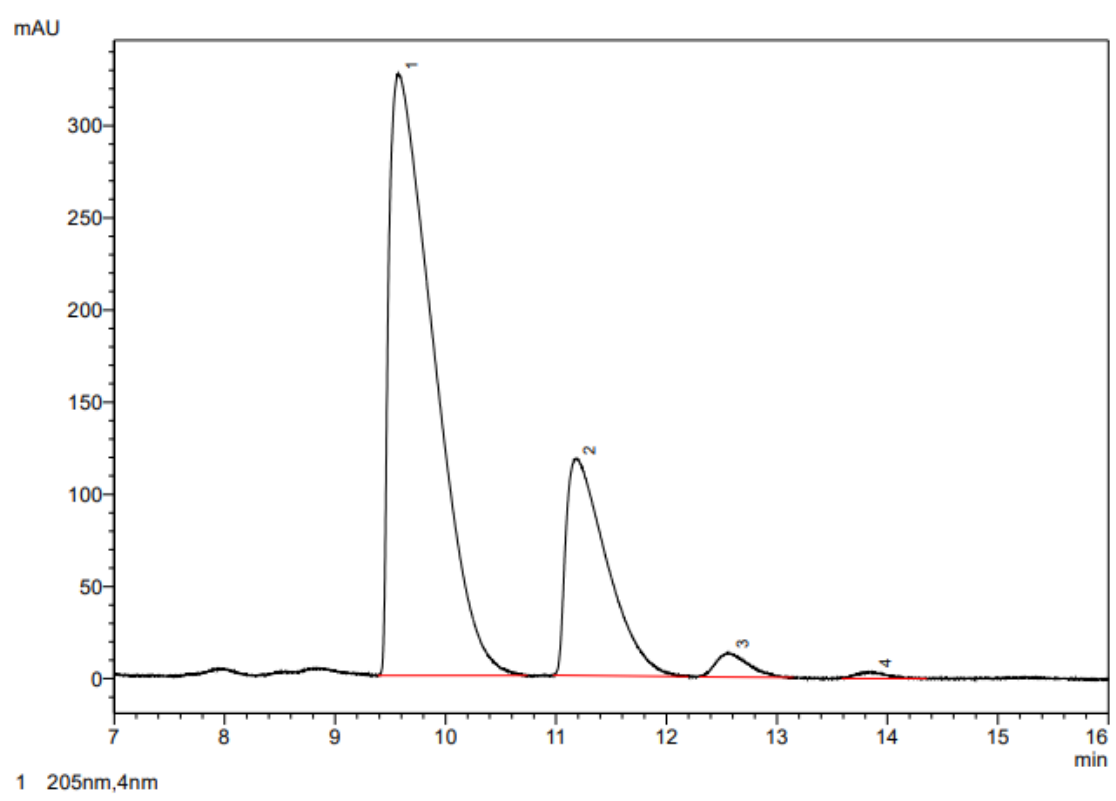

PDA Ch1 205nm

Peak #	Ret. Time	Area %	Name
1	9,57	73,14	1. Enantiomer Diastereomer 1 94.4 % ee
2	11,18	24,19	1. Enantiomer Diastereomer 2 95.5 % ee
3	12,57	2,11	1. Enantiomer Diastereomer 1
4	13,86	0,56	2. Enantiomer Diastereomer 2
Total		100,00	

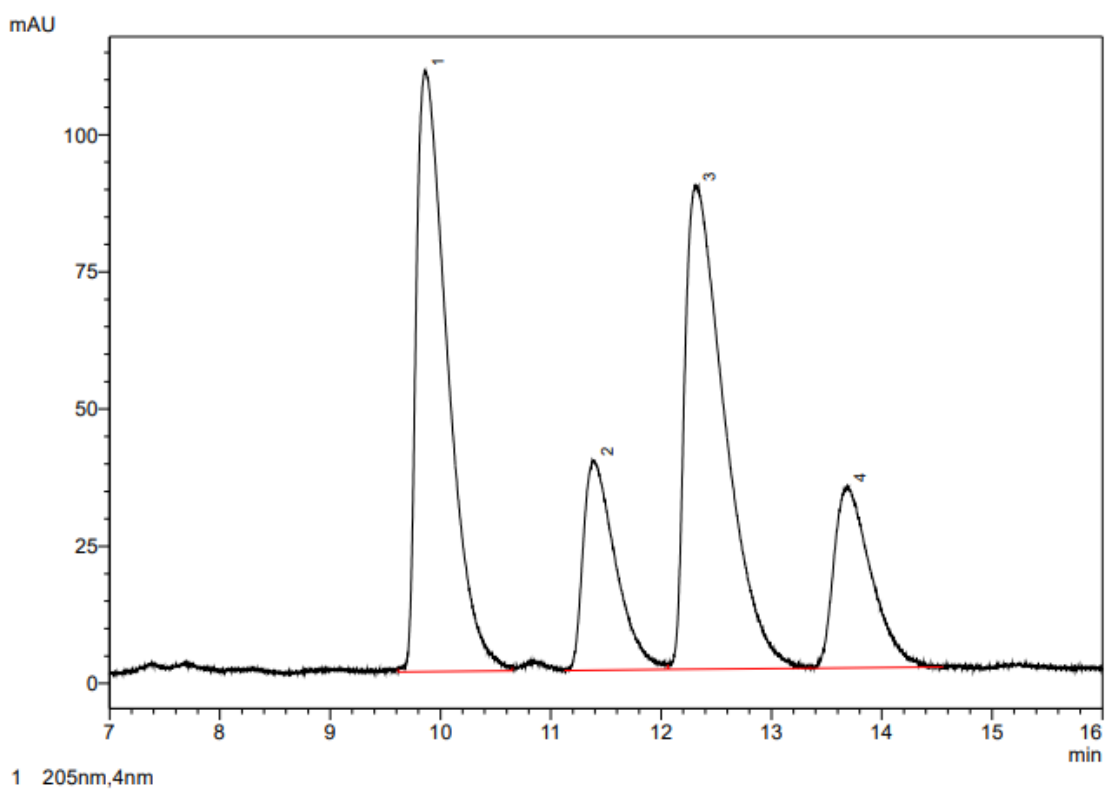

PDA Ch1 205nm

Peak #	Ret. Time	Area %	Name
1	9,86	36,51	1. Enantiomer Diastereomer 1
2	11,39	13,10	1. Enantiomer Diastereomer 2
3	12,31	37,33	2. Enantiomer Diastereomer 1
4	13,68	13,05	2. Enantiomer Diastereomer 2
Total		100,00	

The diastereomers were assigned based on NOESY data:

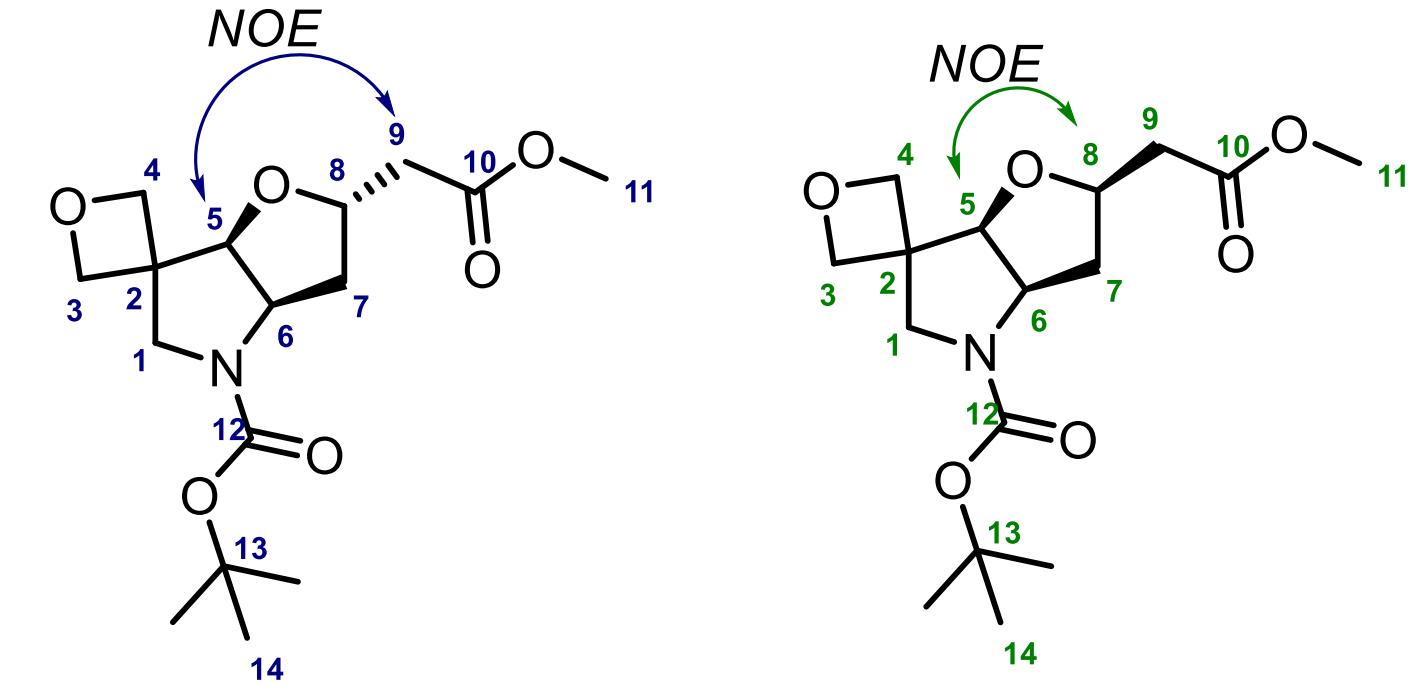

major isomer *minor isomer*

(11*S*,11a*R*)-11-Hydroxy-1,2,3,6,11,11a-hexahydro-4*H*-pyrido[1,2-*b*]isoquinolin-4-one (21).

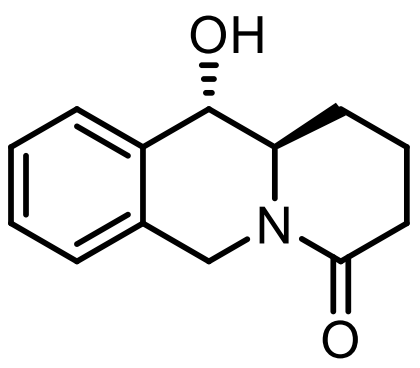

A Schlenk flask was charged under argon with a solution of benzyl (3*R*,4*S*)-4-hydroxy-3-((*E*)-4-methoxy-4-oxobut-2-en-1-yl)-3,4-dihydroisoquinoline-2(1*H*)-carboxylate **8b** (34.5 mg, 90.5 µmol, 93% ee) in ethyl acetate (1.0 mL). Methanol (1.0 mL) and Pd/C (9.6 mg, 9.05 µmol) were added and the mixture was purged with H_2 from a balloon for 1 min. The mixture was then stirred under a H_2-atmosphere for 16 h at room temperature. The suspension was filtered through a plug of Celite, thoroughly rinsing with methanol (20 mL). The combined filtrates were evaporated under reduced pressure to give an off-white solid, which was used in the next step without further purification.

The solid was dissolved in ethanol (3.0 mL) and triethylamine (126 µL, 0.90 mmol) was added. The mixture was stirred at reflux temperature for 24 h. The volatiles were removed under reduced pressure and the residue was purified by flash chromatography (SiO_2, ethyl acetate) to give the title compound as a colorless oil (15.7 mg, 80%, 94% ee). $[\alpha]_D^{20} = -143.7$ (c = 0.75, $CHCl_3$); ^1H NMR (400 MHz, CD_3OD): δ = 7.61 – 7.53 (m, 1H), 7.29 – 7.22 (m, 2H), 7.17 – 7.13 (m, 1H), 5.31 (d, *J* = 17.2 Hz, 1H), 4.57 (d, *J* = 9.8 Hz, 1H), 4.18 (d, *J* = 17.2 Hz, 1H), 3.44 (dt, *J* = 10.1, 5.2 Hz, 1H), 2.42 (t, *J* = 6.4 Hz, 2H), 2.15 (dt, *J* = 7.2, 5.2 Hz, 2H), 2.02 – 1.87 (m, 1H), 1.90 – 1.70 (m, 1H); ^{13}C NMR (101 MHz, CD_3OD): δ = 172.2, 139.4, 133.3, 128.5, 127.9, 127.5, 126.7, 70.1, 60.6, 45.8, 33.2, 25.2, 18.4; IR (ATR): ṽ = 3341, 2947, 2874, 1611, 1580, 1445, 1415, 1251, 1186, 1054, 747 cm^{-1}; HRMS (EI): *m/z*: calcd. for $C_{13}H_{15}NO_2$ $[M]^{+\cdot}$: 217.10973, found: 217.10983.

The racemic product required for the ee determination was prepared analogously from *rac*-**8b**, which in turn had been obtained from **7b** using *rac*-AntPhos as the ligand.

The ee was determined by HPLC analysis: Chiralpak 150 mm IB-N-3, 3 µm, 4.6 mm ∅, acetonitrile/water = 20:80, v = 1.0 mL/min, λ = 220 nm, t(major) = 6.23 min, t(minor) = 7.86 min, (enantioenriched sample: left, racemic sample: right).

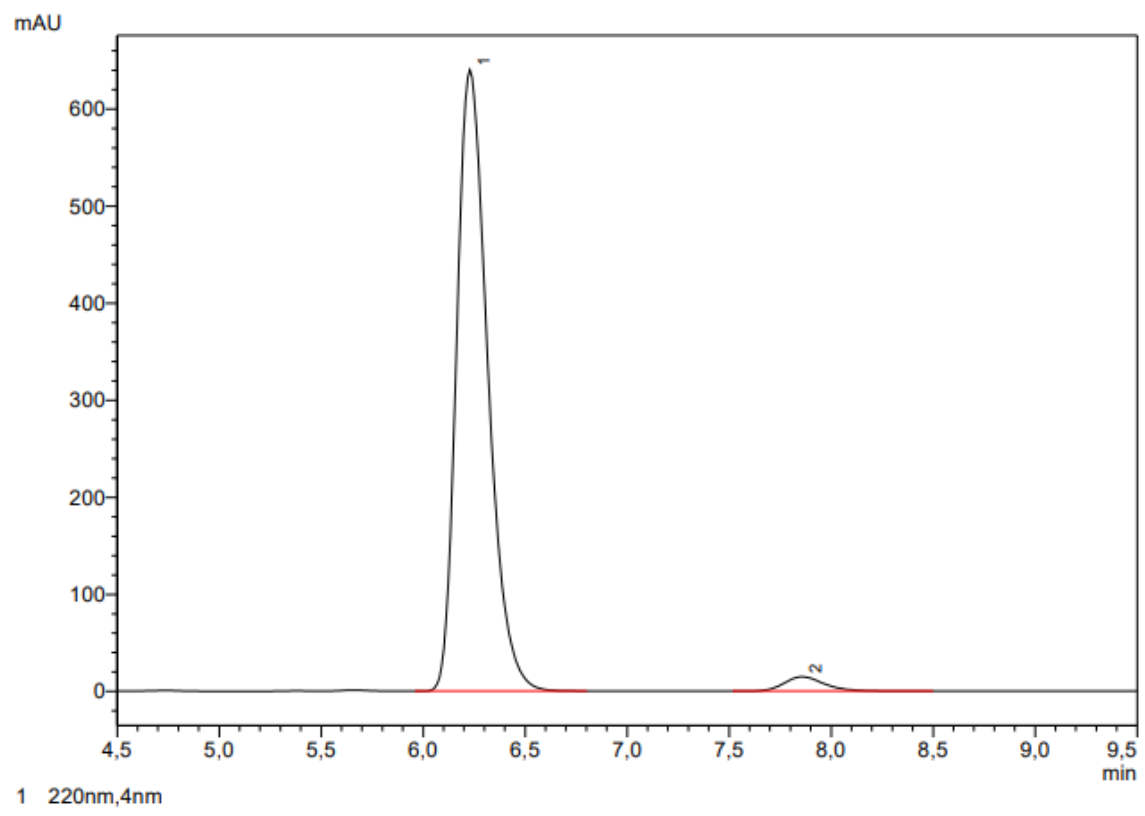

PDA Ch1 220nm

Peak #	Ret. Time	Area %	Name
1	6,23	97,10	1. Enantiomer 94.2 % ee
2	7,86	2,90	2. Enantiomer
Total		100,00	

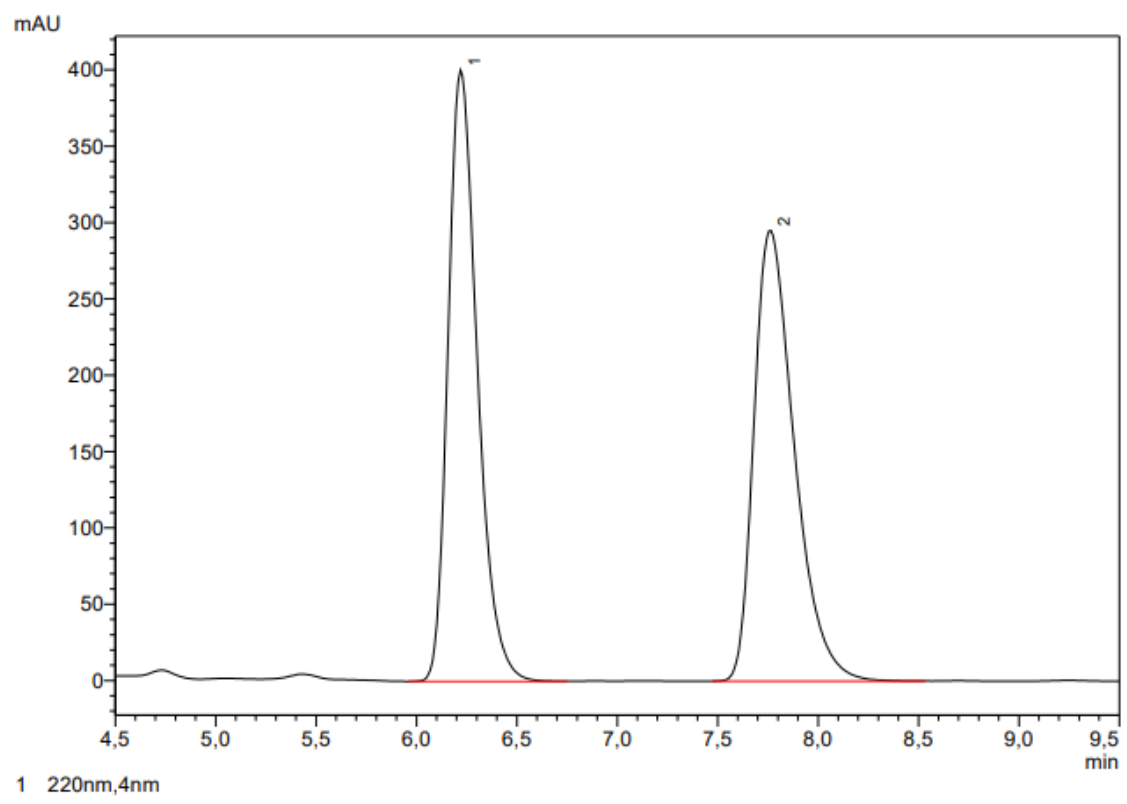

PDA Ch1 220nm

Peak #	Ret. Time	Area %	Name
1	6,22	49,53	1. Enantiomer
2	7,76	50,47	2. Enantiomer
Total		100,00	

(11*S*,11a*R*)-1,3,4,6,11,11a-Hexahydro-2*H*-pyrido[1,2-*b*]isoquinolin-11-ol (22).

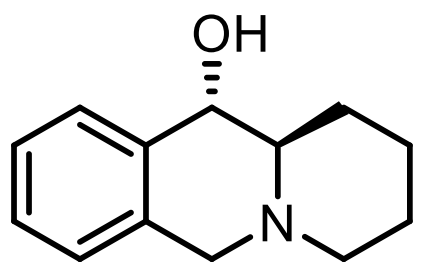

A solution of (11*S*,11a*R*)-11-Hydroxy-1,2,3,6,11,11a-hexahydro-4*H*-pyrido[1,2-*b*]isoquinolin-4-one **21** (15.0 mg, 69.0 µmol) in THF (0.3 mL) was added dropwise to a solution of lithium aluminium hydride (7.9 mg, 0.21 mmol) in THF (0.3 mL) at 0 °C (bath temperature). After complete addition, the mixture was stirred at 50 °C (bath temperature) for 1 h. The mixture was cooled to 0 °C before sat. aq. NH_4Cl solution (1 mL) was added dropwise, followed by sat. aq. Rochelle salt solution (1 mL). The mixture was diluted with dichloromethane (2 mL) and was vigorously stirred for 10 min. The organic phase was separated and the aqueous layer was extracted with dichloromethane (4 x 8 mL). The combined organic phases were washed with brine (5 mL), dried over $MgSO_4$ and filtered, and the solvent was removed under reduced pressure. The residue was purified by flash chromatography (*n*-pentane/ethyl acetate, 1:2 + 3% NEt_3) to give the title compound as an off-white solid (10.3 mg, 73%, 94% ee).

$[\alpha]_D^{20}$ = −23.6 (c = 0.50, MeOH); ^{1}H NMR (400 MHz, CD_3OD): δ = 7.59 – 7.40 (m, 1H), 7.34 – 7.16 (m, 2H), 7.13 – 6.96 (m, 1H), 4.38 (d, *J* = 8.7 Hz, 1H), 3.81 (d, *J* = 15.2 Hz, 1H), 3.52 – 3.38 (m, 1H), 3.19 – 2.94 (m, 1H), 2.40 – 2.32 (m, 1H), 2.23 (ddd, *J* = 12.5, 11.6, 3.1 Hz, 1H), 2.13 (ddd, *J* = 10.1, 8.7, 3.2 Hz, 1H), 1.92 – 1.82 (m, 1H), 1.79 – 1.70 (m, 1H), 1.62 (dtt, *J* = 13.3, 12.4, 3.7 Hz, 1H), 1.47 – 1.21 (m, 2H); ^{13}C NMR (101 MHz, CD_3OD): δ = 138.5, 134.6, 128.2, 128.0, 127.9, 126.4, 73.3, 66.8, 58.9, 57.0, 31.3, 26.2, 24.7; IR (ATR): ṽ = 3340, 2929, 2853, 2811, 1451, 1350, 1124, 1031, 747 cm^{-1}; HRMS (EI): *m/z*: calcd. for $C_{13}H_{17}NO$ $[M]^{+\cdot}$: 203.13046, found: 203.13045.

The racemic product required for the ee determination was prepared analogously from *rac*-**21**

The ee was determined by HPLC analysis: Chiralpak 150 mm OJ-3R, 3 µm, 4.6 mm ∅, acetonitrile/aq. NH_4HCO_3 (pH 9) = 30:70, v = 1.0 mL/min, λ = 220 nm, t(minor) = 3.64 min, t(major) = 3.97 min, (enantioenriched sample: left, racemic sample: right).

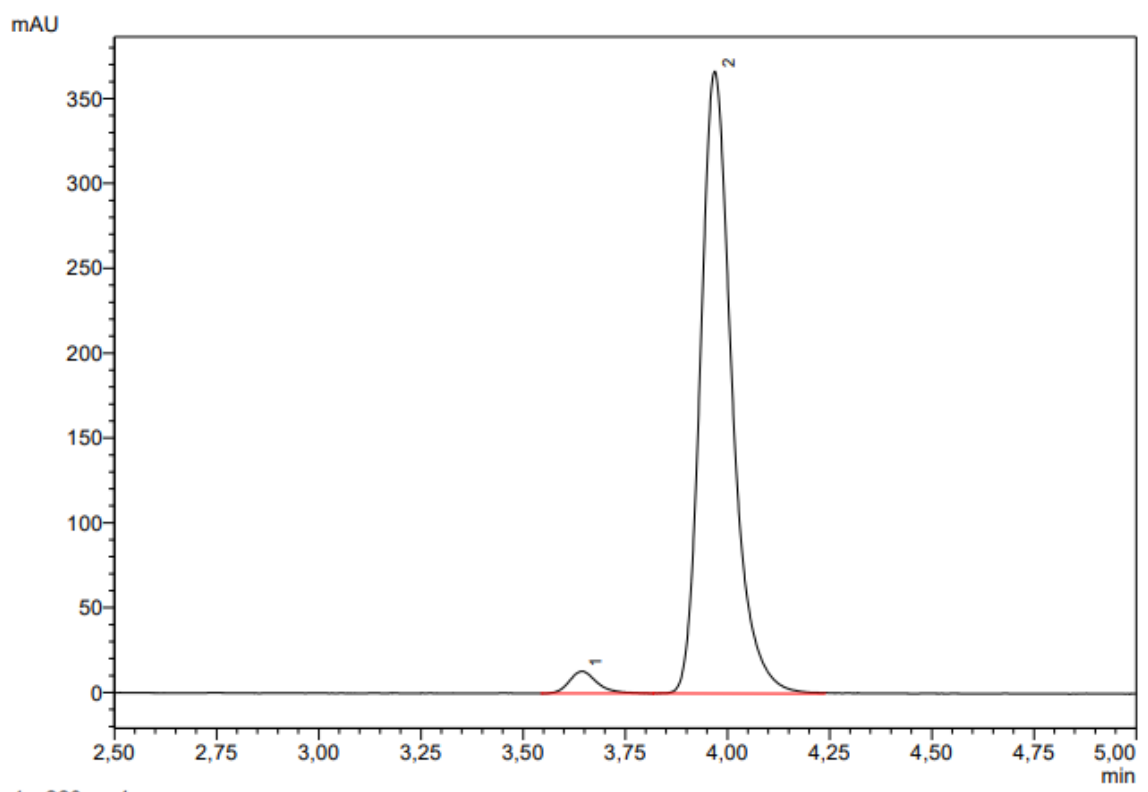

1 220nm,4nm

PDA Ch1 220nm

Peak #	Ret. Time	Area %	Name
1	3,64	3,11	1. Enantiomer
2	3,97	96,89	2. Enantiomer 93.8 % ee
Total		100,00	

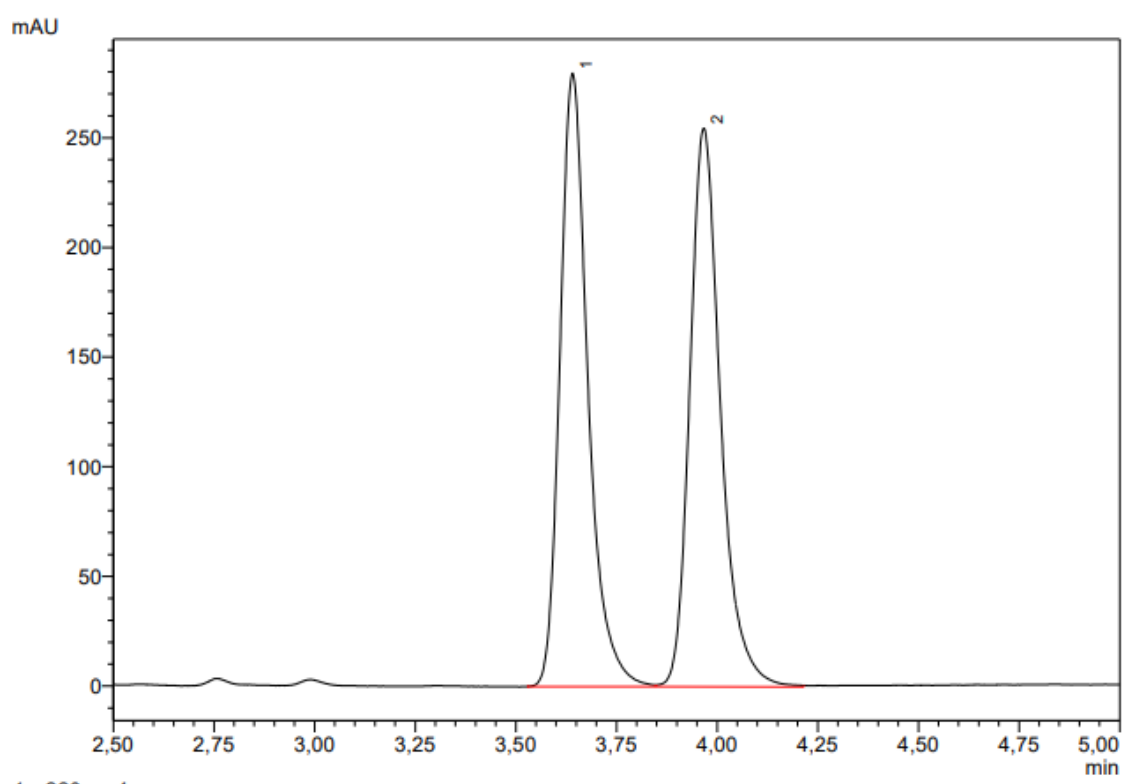

1 220nm,4nm

PDA Ch1 220nm

Peak #	Ret. Time	Area %	Name
1	3,64	50,04	1. Enantiomer
2	3,97	49,96	2. Enantiomer
Total		100,00	

(2*S*,3*R*,11*S*,11a*R*)-2,3,11-Trihydroxy-1,2,3,6,11,11a-hexahydro-4*H*-pyrido[1,2-*b*]isoquinolin-4-one (23).

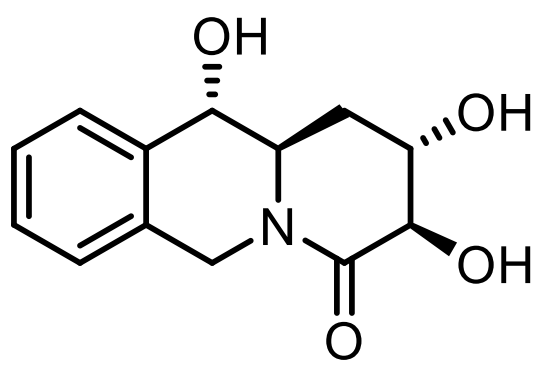

major isomer

Benzyl (3*R*,4*S*)-4-hydroxy-3-((*E*)-4-methoxy-4-oxobut-2-en-1-yl)-3,4-dihydro-isoquinoline-2(1*H*)-carboxylate **8b** (34.3 mg, 0.09 mmol, 93% ee) was added to a solution of $K_3[Fe(CN)_6]$ (89.0 mg, 0.27 mmol), K_2CO_3 (37.3 mg, 0.27 mmol), $K_2OsO_4·2H_2O$ (1.0 mg, 2.7 µmol), $(DHQ)_2PHAL$ (4.2 mg, 5.4 µmol) and methanesulfonamide (17.1 mg, 0.18 mmol) in 1:1 *tert*-butanol/water (2 mL) at 0 °C (bath temperature). The mixture was stirred at 0 °C for 24 h. Sat. aq. $Na_2S_2O_3$ (5 mL) was added and the mixture was vigorously stirred for 10 min before it was diluted with ethyl acetate (10 mL). The organic phase was separated, the aqueous layer was extracted with ethyl acetate (3 x 10 mL), and the combined organic phases were washed with brine (10 mL), dried over $MgSO_4$, filtered, and the solvent was removed under reduced pressure.

The residue was dissolved in methanol (2 mL) and the solution transferred into a Schlenk flask under argon. Pd/C (9.6 mg, 9.0 µmol) was added, the mixture was purged with H_2 from a balloon for 1 min, and was then stirred under H_2 atmosphere for 20 h. The suspension was filtered through a plug of Celite and the filtrate was evaporated under reduced pressure. The residue was purified by flash chromatography (SiO_2, dichloromethane + 5% methanol → dichloromethane + 10% methanol → acetone + 30% methanol) to furnish the title compound as a pale brown solid (18.3 mg, 82% over 2 steps, dr ≈ 10:1). $[\alpha]_D^{20}$ = –11.8 (c = 0.85, MeOH); ^{1}H NMR (600 MHz, CD_3OD): δ = 7.57 (dt, *J* = 7.5, 1.2 Hz, 1H), 7.30 – 7.20 (m, 2H), 7.15 (dd, *J* = 7.3, 1.1 Hz, 1H), 5.30 (d, *J* = 16.9 Hz, 1H), 4.57 (d, *J* = 10.0 Hz, 1H), 4.21 (d, *J* = 16.9 Hz, 1H), 3.98 (ddd, *J* = 10.1, 7.8, 3.9 Hz, 1H), 3.89 (dd, *J* = 7.9, 1.0 Hz, 1H), 3.53 (ddd, *J* = 9.9, 6.1, 3.8 Hz, 1H), 2.54 (dt, *J* = 13.9, 3.9 Hz, 1H), 2.13 (ddd, *J* = 13.9, 10.1, 6.2 Hz, 1H); ^{13}C NMR (151 MHz, CD_3OD): δ = 171.6, 139.2, 133.4, 128.6, 128.0, 127.8, 126.6, 75.0, 70.4, 67.7, 58.1, 45.7, 30.0; IR (ATR): ṽ = 3317, 1636, 1443, 1249, 1053, 753 cm^{-1}; HRMS (ESI$^+$): *m/z*: calcd. for $C_{13}H_{15}NO_4Na$ $[M+Na]^+$: 272.08933, found: 272.08950.

The selectivity of the dihydroxylation was assigned by the established stereochemical model[11] and the assignment confirmed by NOE-data:

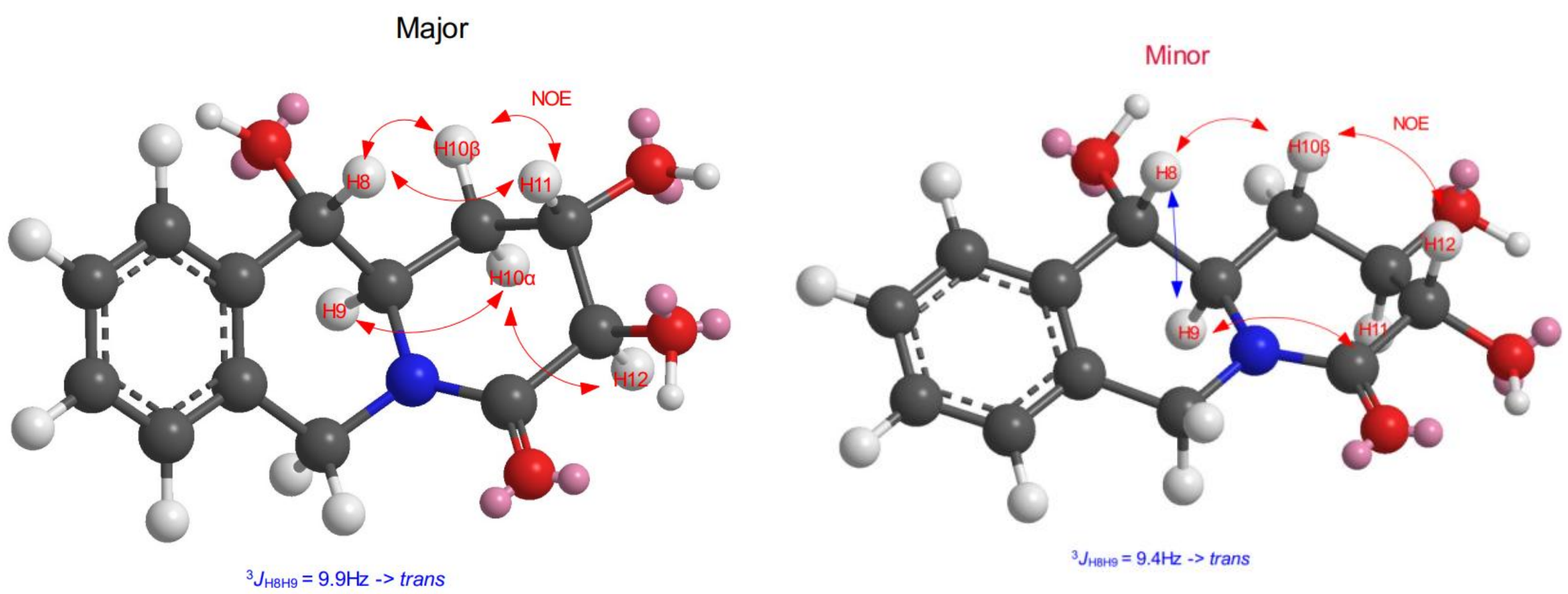

(2*S*,3*S*,11*S*,11a*R*)-1,3,4,6,11,11a-Hexahydro-2H-pyrido[1,2-*b*]isoquinoline-2,3,11-triol (24).

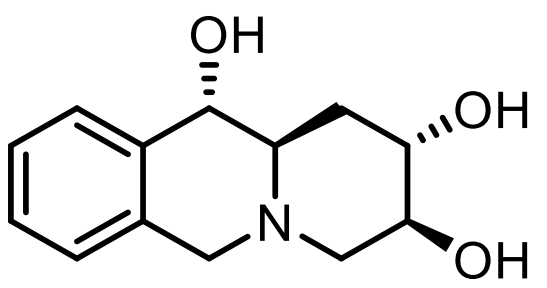

BH_3·SMe_2 (44.1 µL, 0.44 mmol) was added dropwise to a solution of (2*S*,3*R*,11*S*,11a*R*)-2,3,11-trihydroxy-1,2,3,6,11,11a-hexahydro-4*H*-pyrido[1,2-*b*]isoquinolin-4-one **23** (11.0 mg, 44.1 µmol) in THF at 0 °C (bath temperature). After complete addition, the mixture was stirred at reflux temperature for 5 h. The mixture was cooled to 0 °C before ethanol (2 mL) was added dropwise, and the resulting mixture was vigorously stirred for 10 min. The solvent was removed under reduced pressure and the residue was dissolved in ethanol (2 mL). After stirring of the solution at reflux temperature for 2 h, the solvent was removed under reduced pressure and the residue was purified by flash chromatography (SiO_2, ethyl acetate/methanol, 20:3 + 2% NEt_3) to give the title compound as a colorless solid (8.1 mg, 78%). $[\alpha]_D^{20}$ = –17.2 (c = 0.40, MeOH); ^{1}H NMR (400 MHz, CD_3OD): δ = 7.51 (dt, *J* = 7.5, 1.1 Hz, 1H), 7.27 – 7.14 (m, 2H), 7.08 – 7.01 (m, 1H), 4.44 (d, *J* = 8.9 Hz, 1H), 3.87 (q, *J* = 3.5 Hz, 1H), 3.80 (d, *J* = 15.4 Hz, 1H), 3.72 – 3.69 (m, 1H), 3.56 (d, *J* = 15.3 Hz, 1H), 2.89 (dd, *J* = 12.3, 3.1 Hz, 1H), 2.78 (dd, *J* = 12.3, 2.4 Hz, 1H), 2.58 (ddd, *J* = 10.7, 8.9, 3.4 Hz, 1H), 2.26 (dtd, *J* = 14.1, 3.6, 1.3 Hz, 1H), 2.00 – 1.88 (m, 1H); ^{13}C NMR (101 MHz, CD_3OD): δ = 138.6, 134.5, 128.2, 127.8, 126.4, 72.3, 69.5, 68.2, 60.5, 58.5, 56.3, 32.8; IR (ATR): ṽ = 3396, 2923, 1632, 1438, 1353, 1273, 1066, 1033, 746 cm^{-1}; HRMS (ESI$^+$): *m/z*: calcd. for $C_{13}H_{18}NO_3$ $[M+H]^+$: 236.12812, found: 236.12799.

Kinetic Reaction Profile of a Reductive Cyclization Reaction and Speciation of the Crude Reaction Mixture

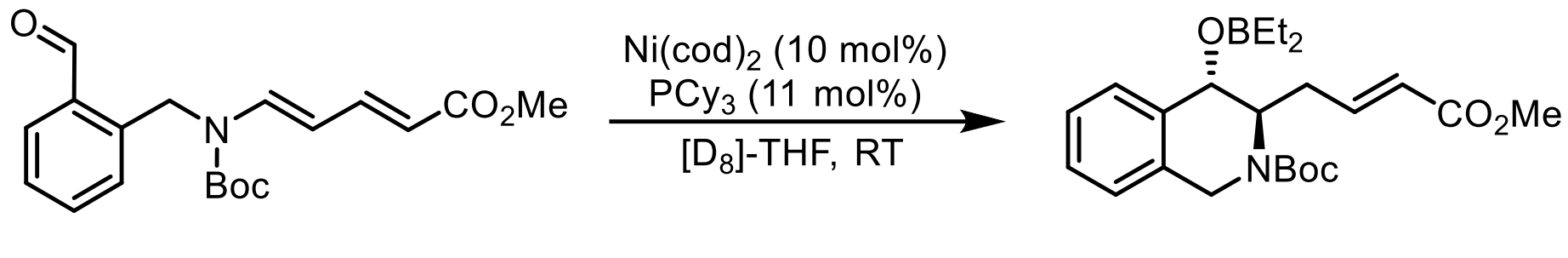

A flame-dried Schlenk flask under argon was charged with solutions of Ni(cod)$_2$ (2.8 mg, 10.0 µmol) in [D$_8$]-THF (0.4 mL) and PCy$_3$ (3.1 mg, 11.0 µmol) in [D$_8$]-THF (0.35 mL). Triethylborane (1.0 M in [D$_8$]-THF, 0.25 mL, 0.25 mmol) and 1,3,5-trimethoxybenzene (5.1 mg, 30.0 µmol) were added and the resulting solution was stirred for 2 min at room temperature before methyl (2*E*,4*E*)-5-((*tert*-butoxycarbonyl)(2-formylbenzyl)amino)penta-2,4-dienoate **7a** (34.5 mg, 0.10 mmol) was introduced under an argon counterflow. The resulting mixture was stirred for ≈30 s until a clear brown solution was obtained and was subsequently transferred into a J-Young tube under argon. The reaction was monitored by NMR spectroscopy (^{1}H and ^{31}P NMR), with the first data point acquired ≈3 min after the reaction was initiated.

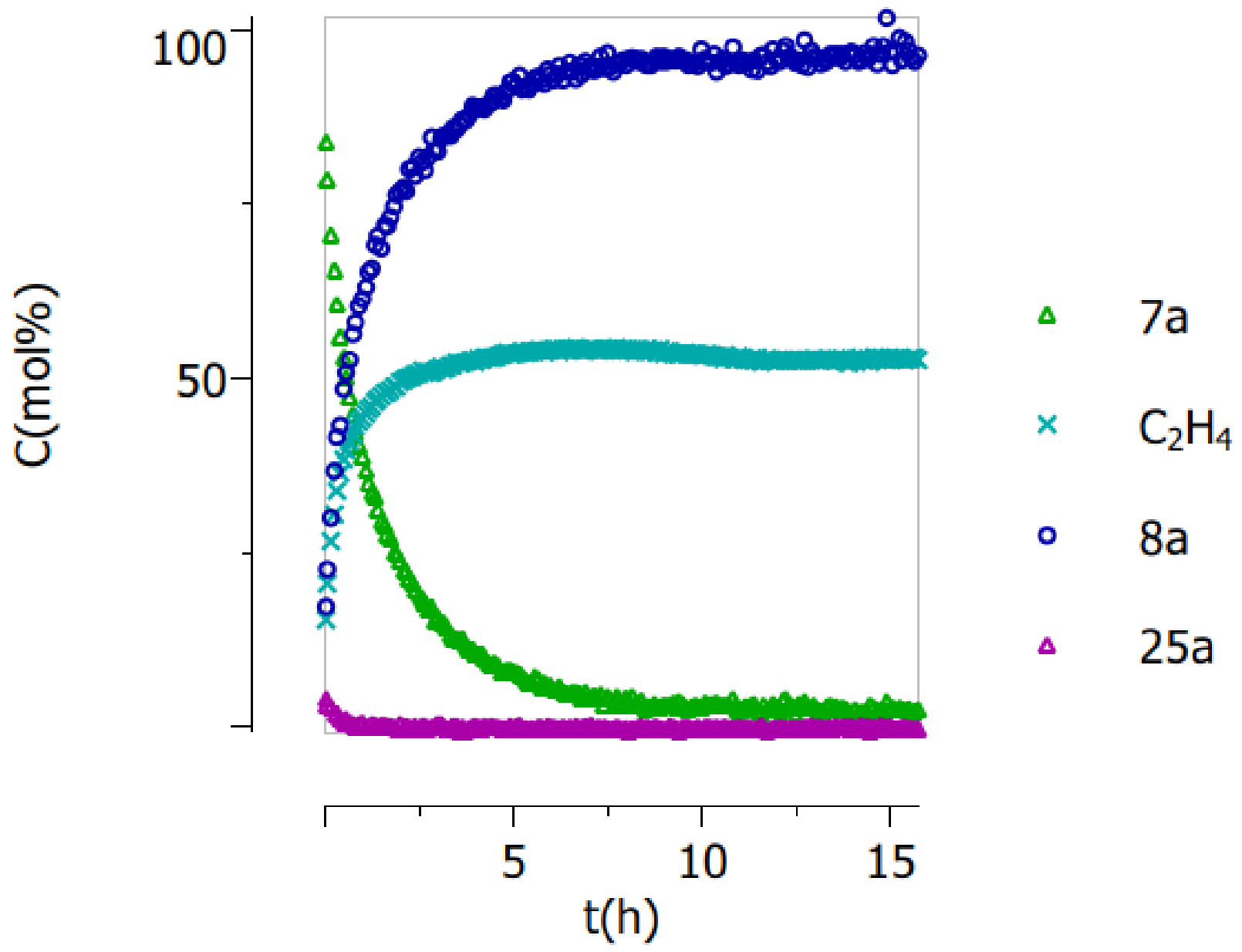

Figure S8. Time-dependent concentration profile of the reductive cyclization reaction; the conversion was determined using 1,3,5-trimethoxybenzene as internal standard.

The reaction profile shown in Figure S8 illustrates the conversion of **7a** and the simultaneous formation of the borinate ester of **8a** as the primary product of the reaction. Likewise, the formation of an equimolar amount of ethylene relative to the converted substrate was observed by ^{1}H NMR spectroscopy, which is formed by β-hydride elimination of the nickel-alkyl intermediate after transmetalation within the catalytic cycle. The ethylene concentration reaches a plateau due to saturation of the THF used as the solvent; there are no signs that the excess ethylene changes the rate or even inhibits the reaction.

After complete conversion of substrate **7a**, the reaction mixture was further analyzed. Specifically, an ^{11}B NMR spectrum showed the signals of residual triethylborane (δ_B = 73.5 ppm, br.), which was used in excess; in addition, the signal of the expected borinate ester of **8a** (δ_B = 53.6 ppm, br.) was detected, which is the primary product of the reaction prior to hydrolytic work-up.

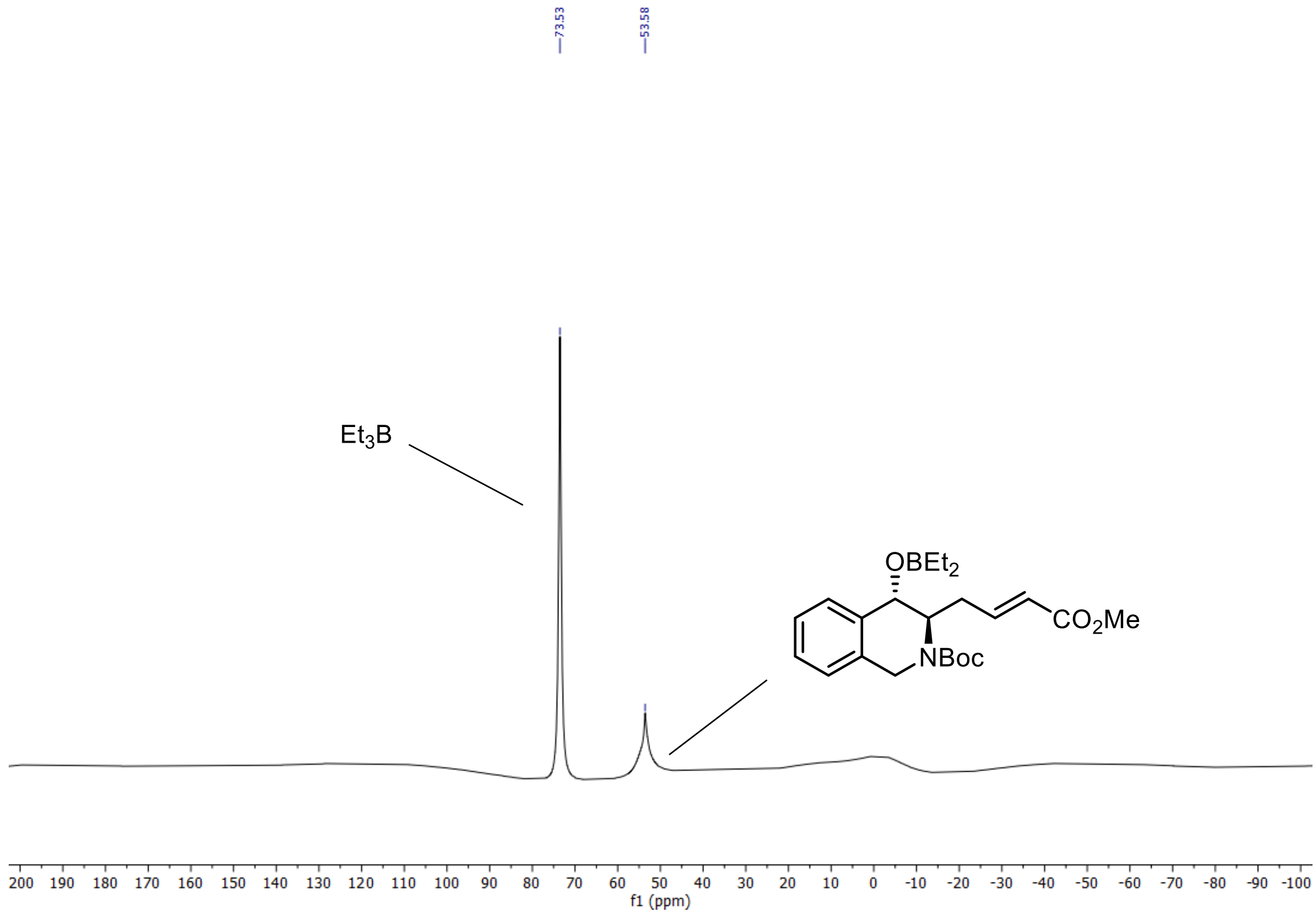

Figure S9. ^{11}B NMR spectrum (193 MHz, [D$_8$]-THF, 298 K) of the reaction mixture after complete conversion of **7a** used as the substrate.

Furthermore, ^{31}P NMR analysis of the reaction mixture was performed both during the catalytic reaction and after its completion. Importantly, the signal of the allyl-oxanickelacycle intermediate **25a** (see below) was clearly detected at the beginning of the reaction but lost intensity; in any case, it was detected during the entire course of the reaction, where it accounts for ca. 1 mol% of the total of 11 mol% of PCy_3 initially added to the reaction mixture.

In addition, several other phosphorous-containing species were observed. Besides $OPCy_3$ ($\delta_P \approx 46.6$ ppm), which is likely formed by oxidation of PCy_3 by trace amounts of oxygen in the NMR tube, several new species were formed during the reductive coupling reaction. The signal at δ_P = 39.4 ppm can be assigned to [Ni(PCy_3)(ethylene)$_2$] by comparison with an authentic sample. This assignment is further supported by 1D and 2D experiments revealing the significantly upfield-shifted resonances of the nickel-bound ethylene (δ_H = 2.41 ppm; δ_C = 50.3 ppm).

A more complex signal pattern consisting of four resonances was observed in the range of δ_P = 33.8–34.5 ppm. Based on fact that these P-containing products were gradually formed during the course of the reaction and the close shift range, these signals were tentatively assigned to a species of the type [Ni(PCy_3)(ethylene)(**8a·BEt$_2$**)] in which the borinate ester primarily formed coordinates to the nickel center by its alkene moiety. The presence of two different signals was attributed to different relative orientations of the coordinated reductive coupling product with respect to the remaining ligands, while additional splitting likely arises from rotamer formation of the N-Boc group, resulting in the observed total of four ^{31}P NMR signals. This assignment is supported by the observation of exchange peaks between two of the four signals (tentatively ascribed to the rotamers) in a ^{31}P-^{1}H EXSY experiment.

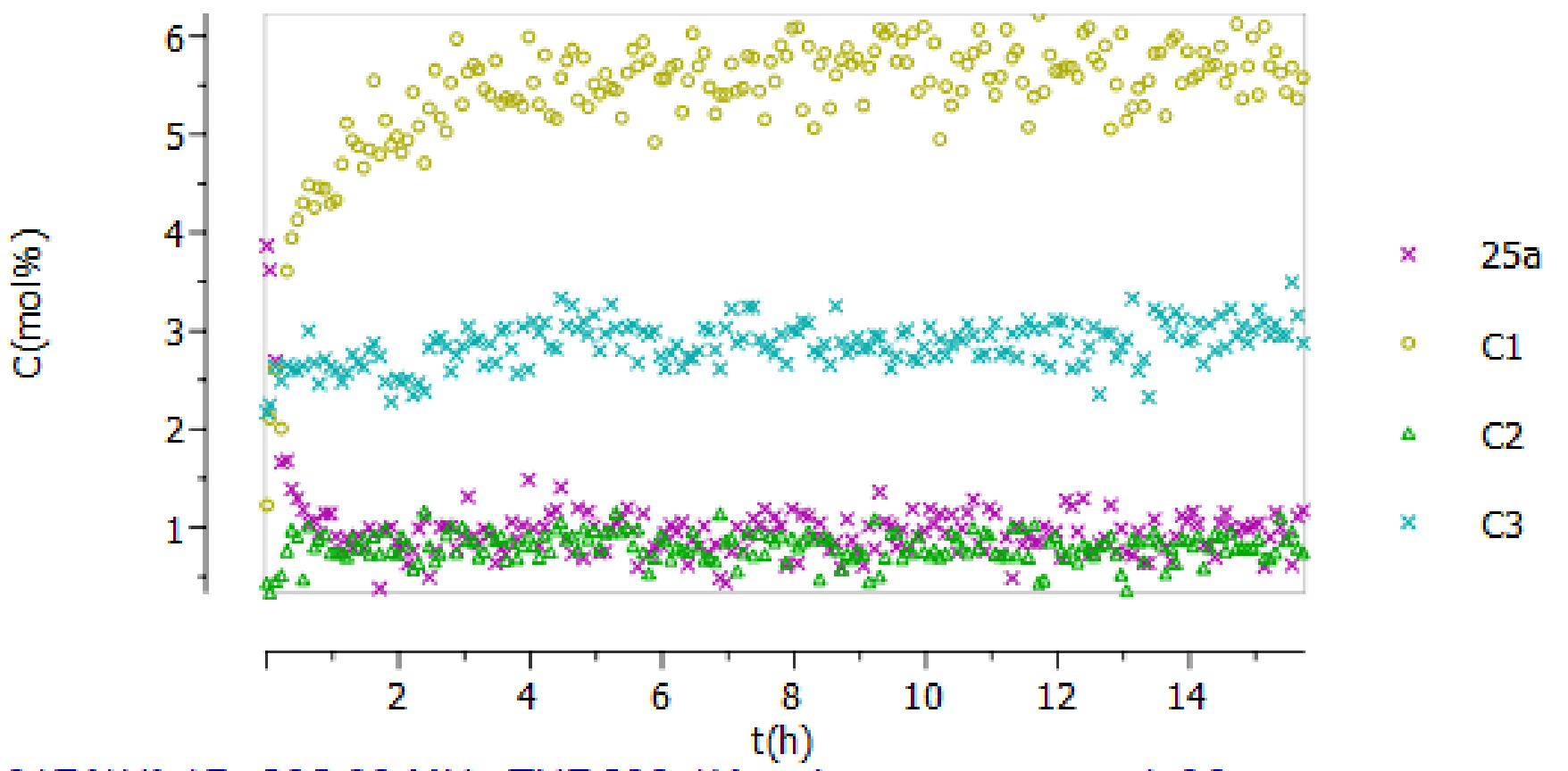

31P{1H},1D, 202.36 MHz,THF,298.1K, pulse sequence: zgig30

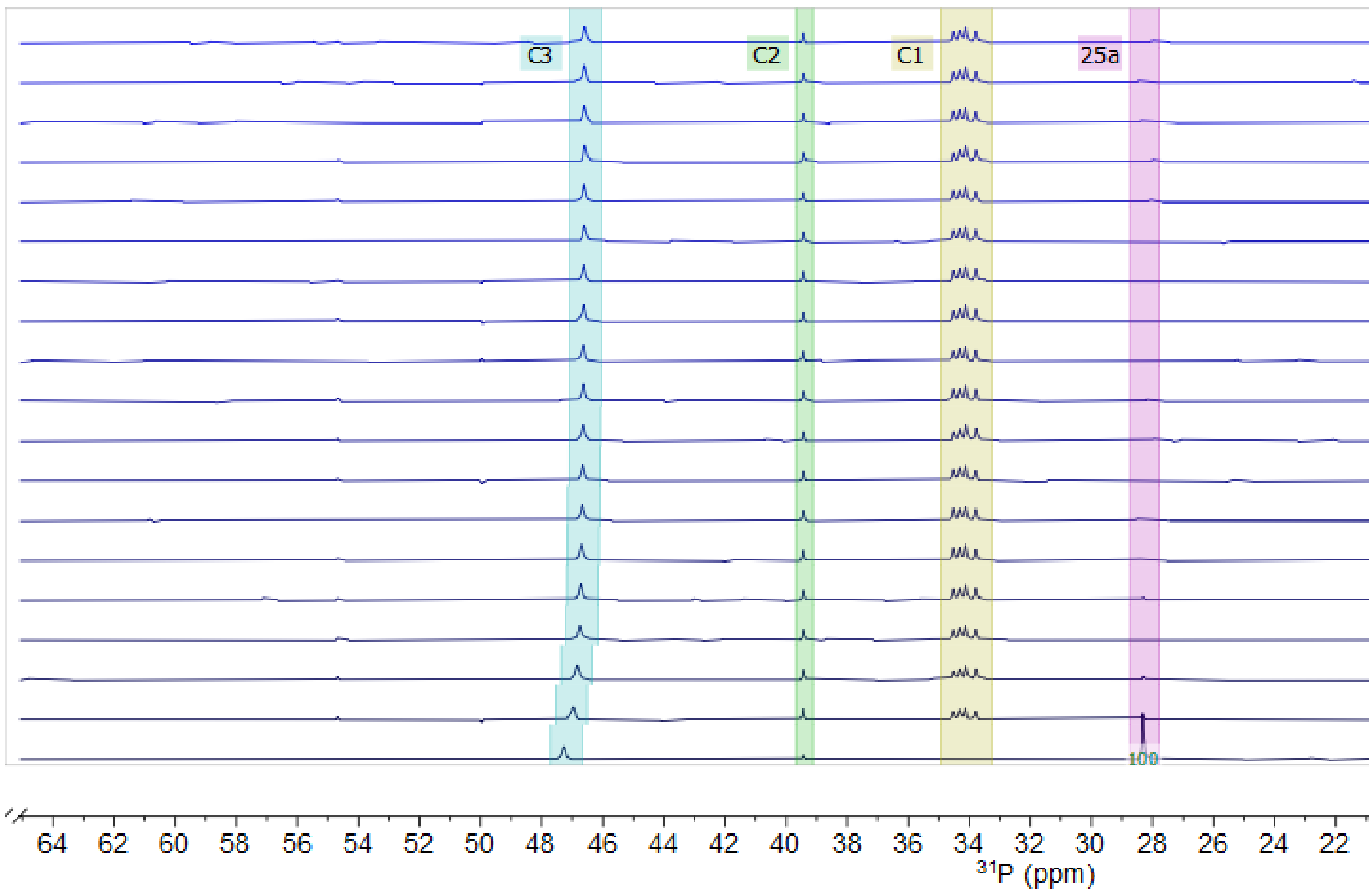

Figure S10. Time-dependent concentration profile of phosphorus-containing species observed during and after the nickel-catalyzed reductive cyclization (202 MHz, [D_8]-THF, 298 K); C1: [Ni(PCy_3)(ethylene)(**8a·BEt₂**)] (see Text); C2: [Ni(PCy_3)(ethylene)$_2$]; C3: Cy_3P=O

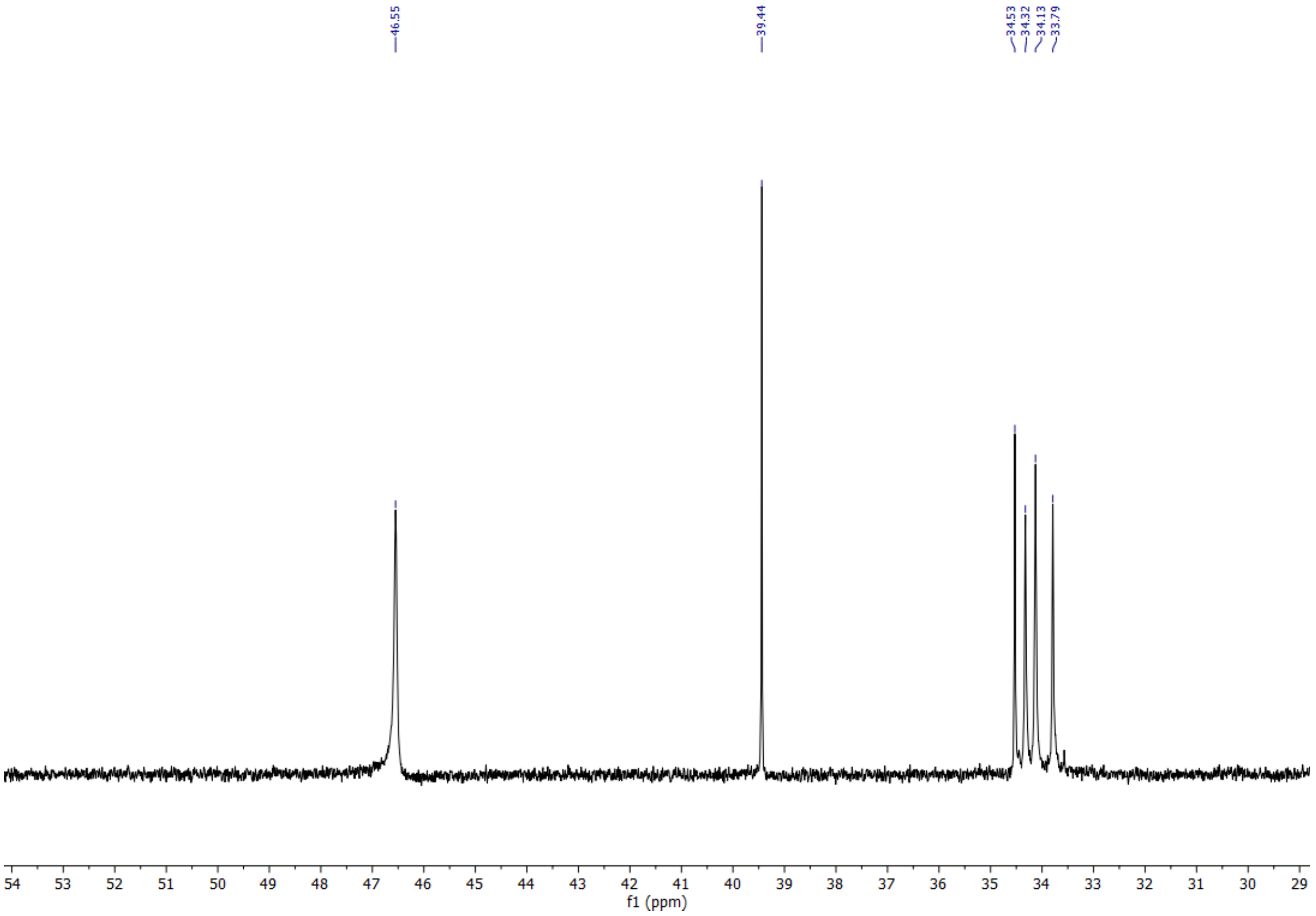

Figure S11. ^{31}P NMR spectrum (243 MHz, [D$_8$]-THF, 298 K) of the reaction mixture after full conversion of substrate **7a**; for the assignment of the Ni(0) speciation, see Figure S10 and the Text

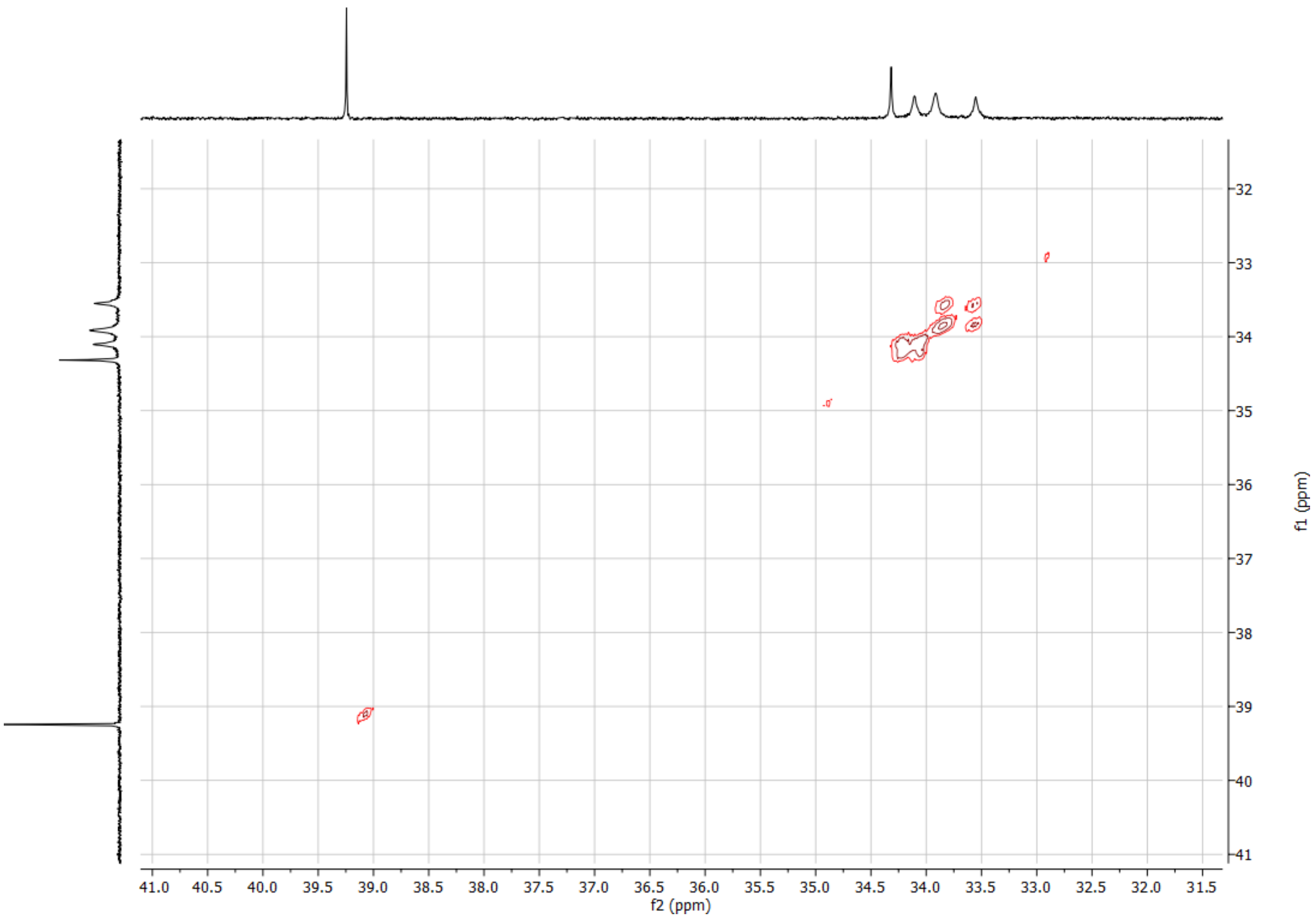

Figure S12. ^{31}P-^{1}H EXSY NMR spectrum (243/600 MHz, [D$_8$]-THF, 323 K) of the reaction mixture after full conversion of substrate **7a**.

Control Experiment with Triethylsilane

A flame-dried Schlenk flask under argon was charged with $Ni(cod)_2$ (15.4 mg, 56.0 µmol) and a solution of PCy_3 (17.3 mg, 61.6 µmol) in [D_8]-THF (0.6 mL). Methyl (2*E*,4*E*)-5-((*tert*-butoxycarbonyl)(2-formylbenzyl)amino)penta-2,4-dienoate **7a** (19.3 mg, 56.0 µmol) was introduced under an argon counterflow and the resulting mixture was stirred at room temperature for 1 h to ensure quantitative formation of allyl-oxanickelacycle **25a** (see below for the preparation and analysis of this complex). Triethylsilane (8.9 µL, 56.0 µmol) was then added and the resulting mixture was stirred at room temperature for 5 min before it was transferred into a J-Young tube under argon.

Analysis of the mixture by ^1H, ^{29}Si and ^{31}P NMR after 30 min and 20 h after addition of the silane showed the allyl-oxanickelacycle **25a** and unreacted Et_3SiH as the virtually only detectable species in solution. The nickelacycle obviously does not react with the silane. These data indicate that a productive turnover *via* σ-bond metathesis, as previously proposed by Mori *et al.*,[12] does not occur to any significant extent under the chosen conditions.

Since substrate **7a** does not succumb to cyclization when exposed to [Ni(*t*Bu-stb)$_3$] (10 mol%), ligand **L17** (11 mol%) and excess Et_3SiH (see main text: Table 1, entry 24), a conceivable alternative pathway involving oxidative addition of the silane to Ni(0), followed by migratory insertion of the resulting Ni(II) hydride complex with formation of an allylnickel species, and subsequent cyclization with formation of a nickelacylce is not operative either; such a mechanism has also been proposed in the literature.[13]

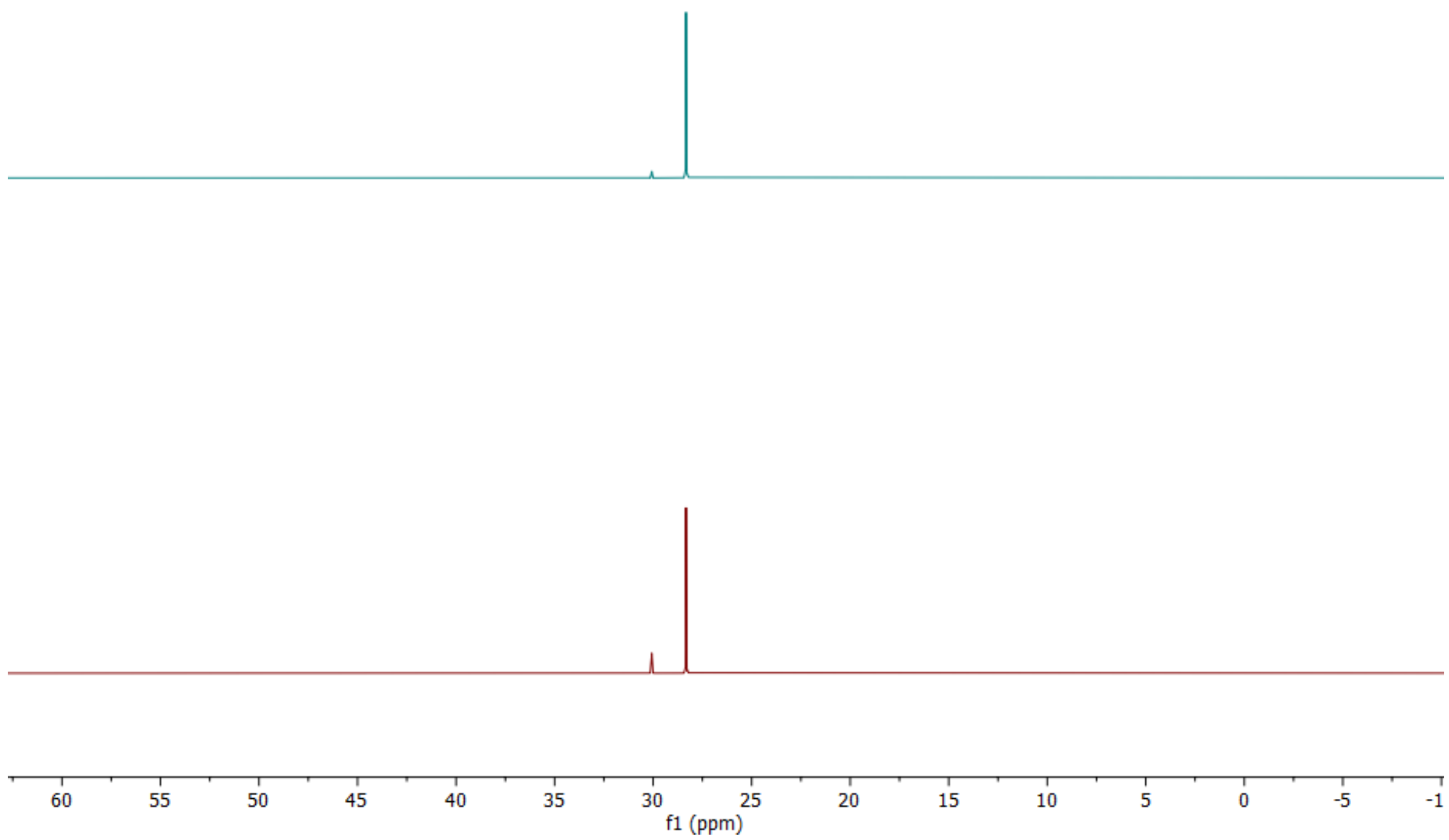

Figure S13. ^{31}P NMR spectrum (243 MHz; [D_8]-THF, 298 K) of the reaction mixture recorded after 30 min (top) and after 20 h (bottom) following the addition of Et_3SiH to the nickelacycle **25a** (δ_P = 28.3 ppm)

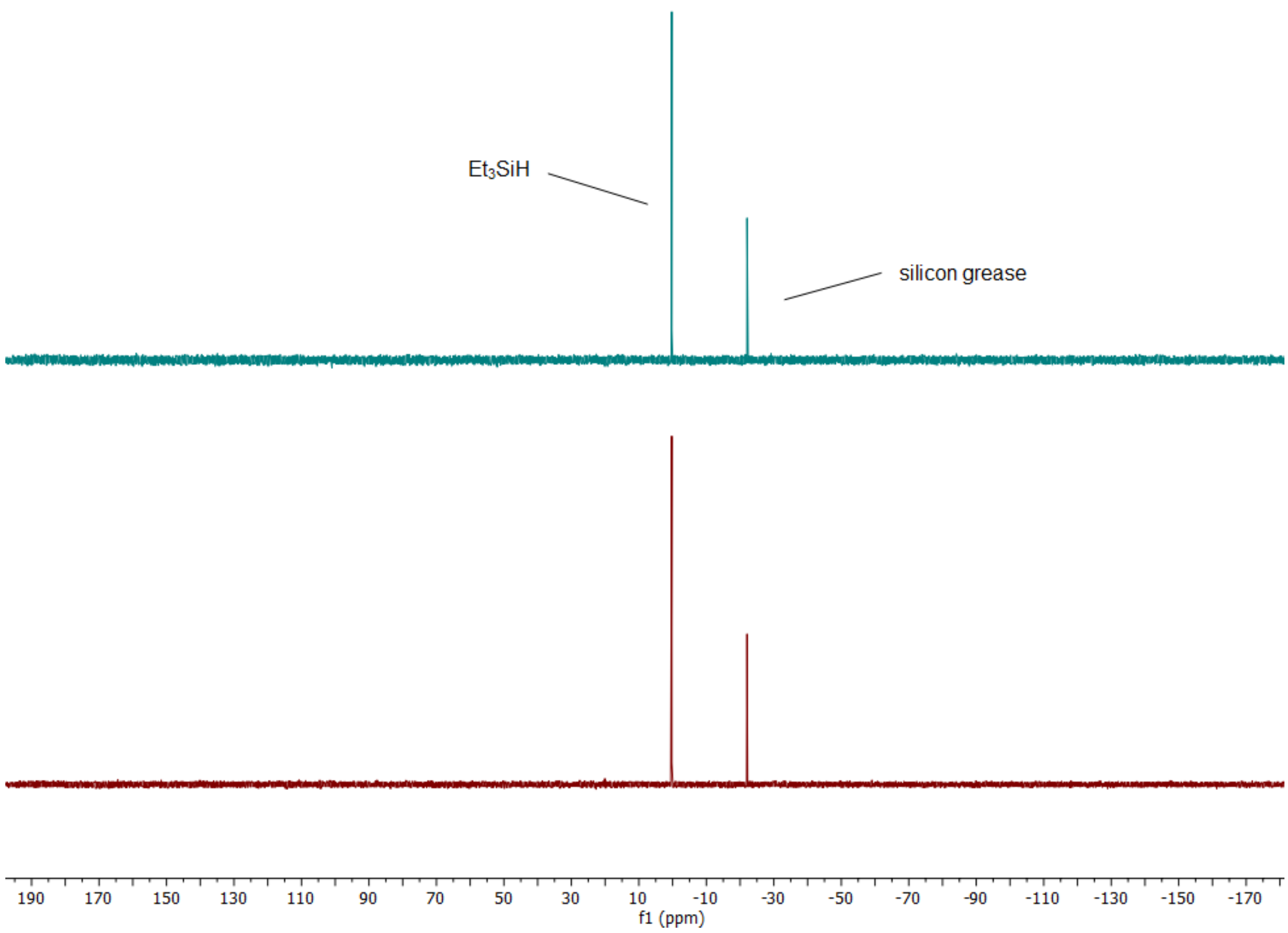

Figure S14. ^{29}Si-refocussed INEPT NMR spectrum (119 MHz, [D$_8$]-THF, 298 K) of the reaction mixture; top = 30 min after addition of Et_3SiH, bottom = 20 h after addition of Et_3SiH to the nickelacycle **25a**.

Preparation and Analysis of the Oxanickelacyclic Complexes

Note: [Ni(cod)$_2$] *was chosen instead of* [Ni(*t*Bu-stb)$_3$] *because the substituted stilbene ligands of the latter are the least soluble component and crystallize before any complex could be obtained. In the NMR studies, they obscure the aromatic/olefinic region of the spectra and hence render the interpretation even more challenging.*

Crystallization of Complex 25e.

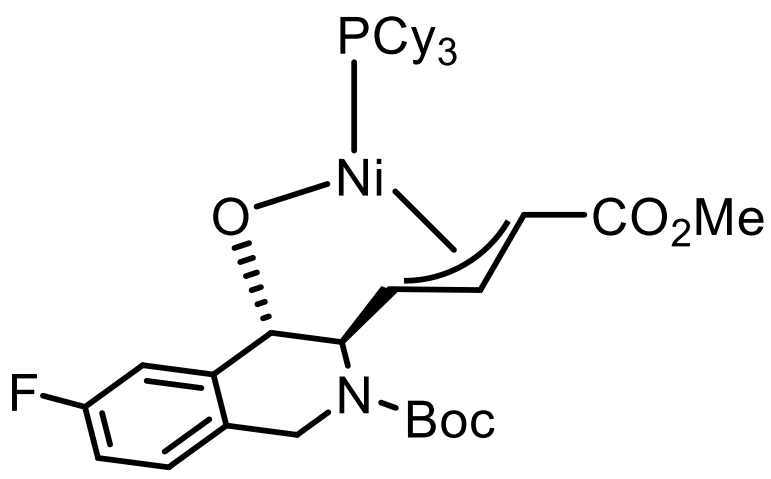

A solution of tricyclohexylphosphine (43.2 mg, 0.15 mmol) in THF (1.5 mL) was added to bis-(1,5-cyclooctadiene)-nickel(0) (38.5 mg, 0.14 mmol) and the resulting yellow-orange solution was stirred at room temperature for 3 min. Methyl (2*E*,4*E*)-5-((*tert*-butoxycarbonyl)(4-fluoro-2-formylbenzyl)amino)penta-2,4-dienoate **7e** (50.9 mg, 0.14 mmol) was added, causing a color change to dark red. The mixture was stirred at room temperature for 1 h before all volatiles were removed *in vacuo* and the residue was suspended in pentane (2.0 mL). After stirring at room temperature for 5 min, the solvent was removed again *in vacuo;* this step was repeated twice. The obtained red solid was dissolved in diethylether (2.0 mL) and pentane (2.0 mL), the solution was filtered *via* cannula, and the filtrate was stored at –20 °C for 2 d, leading to the formation of red-brown crystals suitable for X-ray diffraction analysis.

Crystallization of Complex 25a.

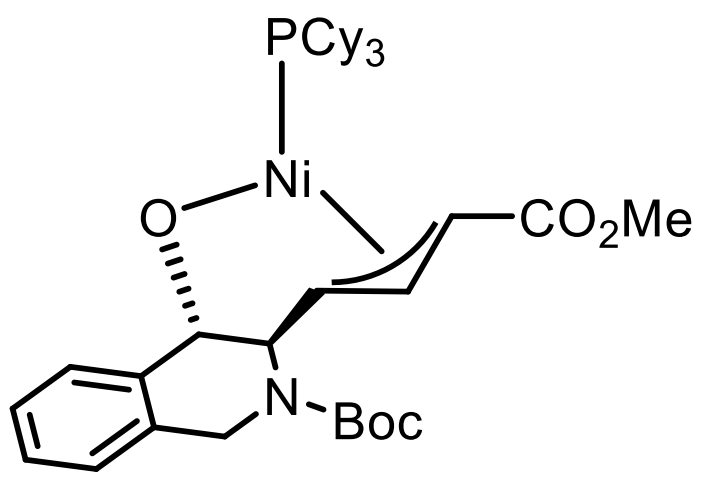

A solution of tricyclohexylphosphine (39.3 mg, 0.14 mmol) in THF (4.5 mL) was added to bis-(1,5-cyclooctadiene)-nickel(0) (35.0 mg, 0.13 mmol) and the resulting yellow-orange solution was stirred at room temperature for 3 min. Methyl (2*E*,4*E*)-5-((*tert*-butoxycarbonyl)-(2-formylbenzyl)amino)penta-2,4-dienoate **7a** (44.0 mg, 0.13 mmol) was added, causing a color change to red-brown. The mixture was stirred at room temperature for 1 h before all volatiles were removed *in vacuo* and the residue was suspended in pentane (2 mL). After stirring at room temperature for 5 min, the solvent was removed *in vacuo*. The resulting red solid was dissolved in diethylether (0.5 mL) and pentane (4.0 mL), the solution was filtered *via* cannula, and the filtrate was stored at –20 °C for 5 weeks, leading to the formation of red-brown crystals suitable for X-ray diffraction analysis.

NMR Analysis of Complex 25a.

A solution of tricyclohexylphosphine (9.2 mg, 32.6 µmol) in [D$_8$]-THF (0.6 mL) was added to bis-(1,5-cyclooctadiene)nickel(0) (8.8 mg, 32.0 µmol) and the resulting yellow-orange mixture was stirred at room temperature for 3 min. Methyl (2*E*,4*E*)-5-((*tert*-butoxycarbonyl)(2-formylbenzyl)amino)penta-2,4-dienoate **7a** (11.1 mg, 32.0 µmol) was added, causing a color change to red-brown. The mixture was stirred at room temperature for 1 h before the solution was transferred into a J-Young NMR tube using a syringe filter. The spectra of the filtrate were recorded at 298 K. ^{1}H NMR (600 MHz, [D$_8$]-THF): δ = 7.33 (d, *J* = 7.4 Hz, 1H), 7.15 (ddd, *J* = 7.4, 6.3, 2.4 Hz, 1H), 7.08 – 7.02 (m, 2H), 6.43 (t, *J* = 12.9 Hz, 1H), 5.33 (d, *J* = 9.5 Hz, 1H), 4.85 (br, 1H), 4.72 (ddd, *J* = 12.9, 9.9, 3.2 Hz, 1H), 4.10 (s, 1H), 3.56 (s, 3H), 3.15 (t, *J* = 9.7 Hz, 1H), 2.06 – 1.99 (m, 9H), 1.88 (dd, *J* = 11.2, 2.3 Hz, 1H), 1.86 – 1.75 (m, 6H), 1.76 – 1.65 (m, 9H), 1.43 (s, 9H), 1.37 – 1.20 (m, 9H); ^{13}C NMR (151 MHz, [D$_8$]-THF): δ = 174.6 (d, *J* = 1.6 Hz), 155.9, 148.2, 132.6, 127.5, 126.1, 125.3, 122.6, 109.5, 103.8 (d, *J* = 11.4 Hz), 92.0 (d, *J* = 8.2 Hz), 79.9, 51.2, 45.7, 36.9, 30.8, 30.7, 28.7, 28.6 (d, *J* = 10.9 Hz), 28.6 (d, *J* = 10.5 Hz), 27.4 (d, *J* = 1.3 Hz); ^{31}P NMR (243 MHz, [D$_8$]-THF): δ = 28.3.

^{1}H NMR (600 MHz, [D$_8$]-THF, 298 K) of complex **25a**

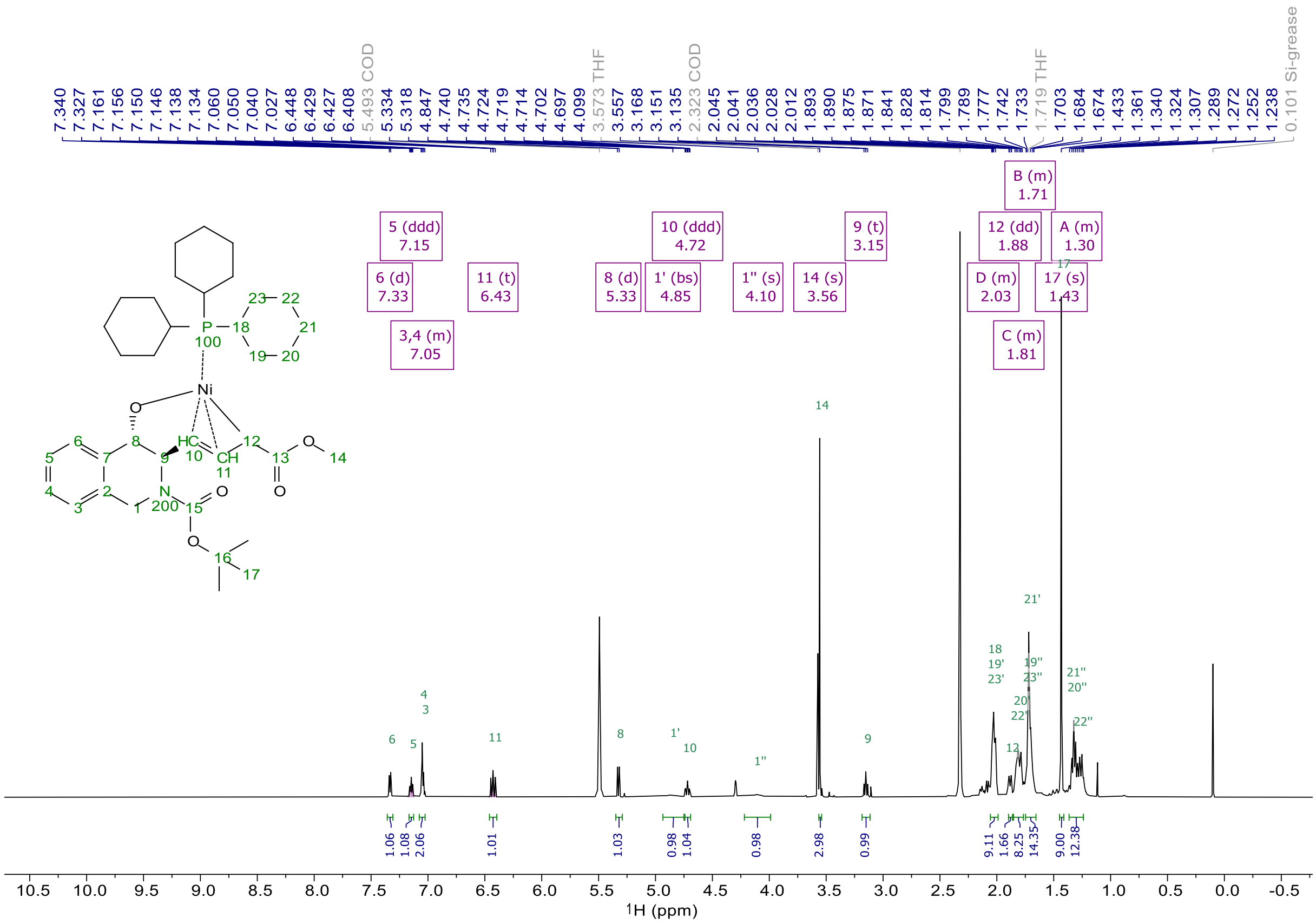

^{13}C NMR (151 MHz, [D$_8$]-THF, 298 K) of complex **25a**

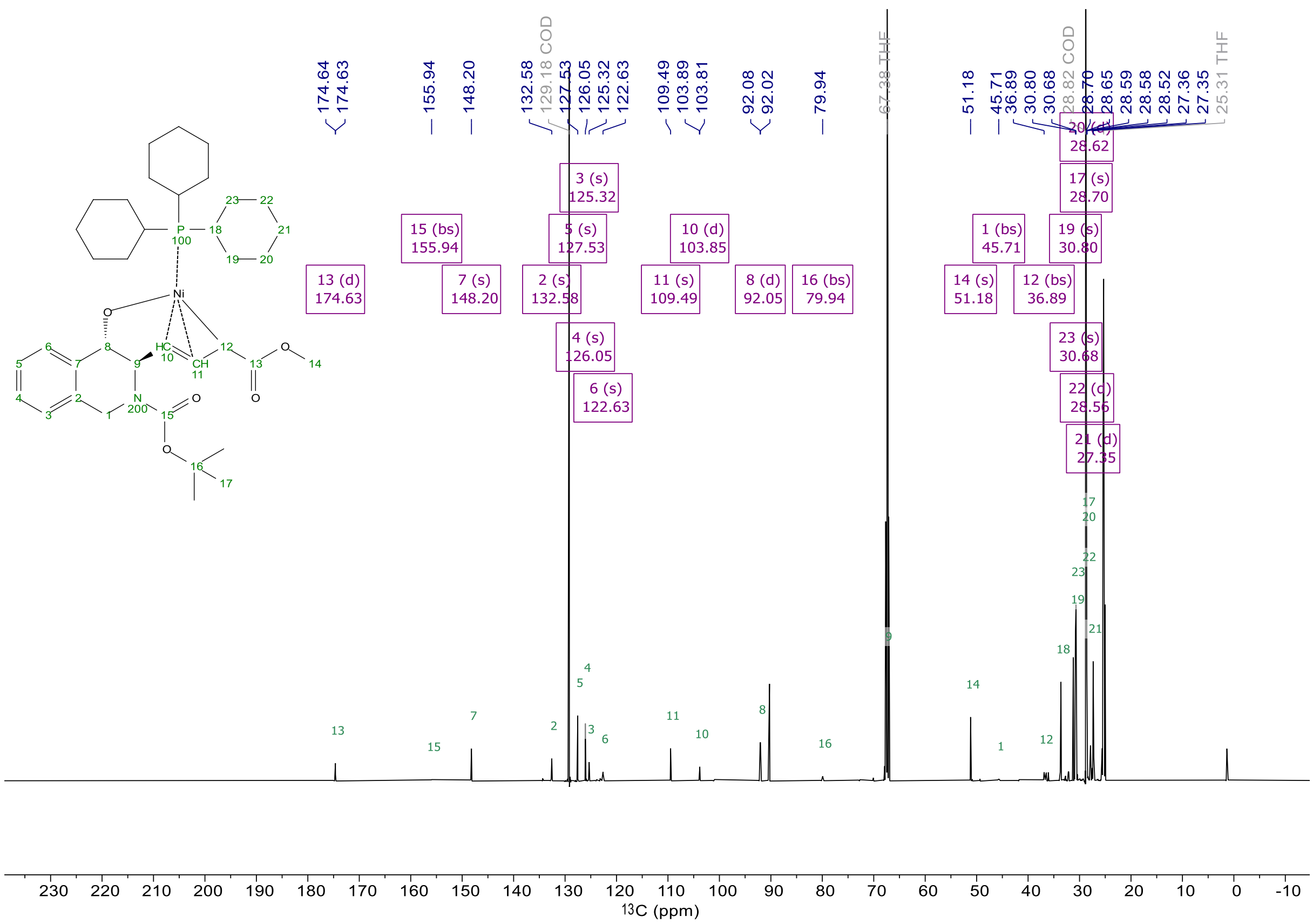

^{1}H-^{13}C HSQC NMR (600 MHz, 151 MHz,[D$_8$]-THF, 298 K) of complex **25a**

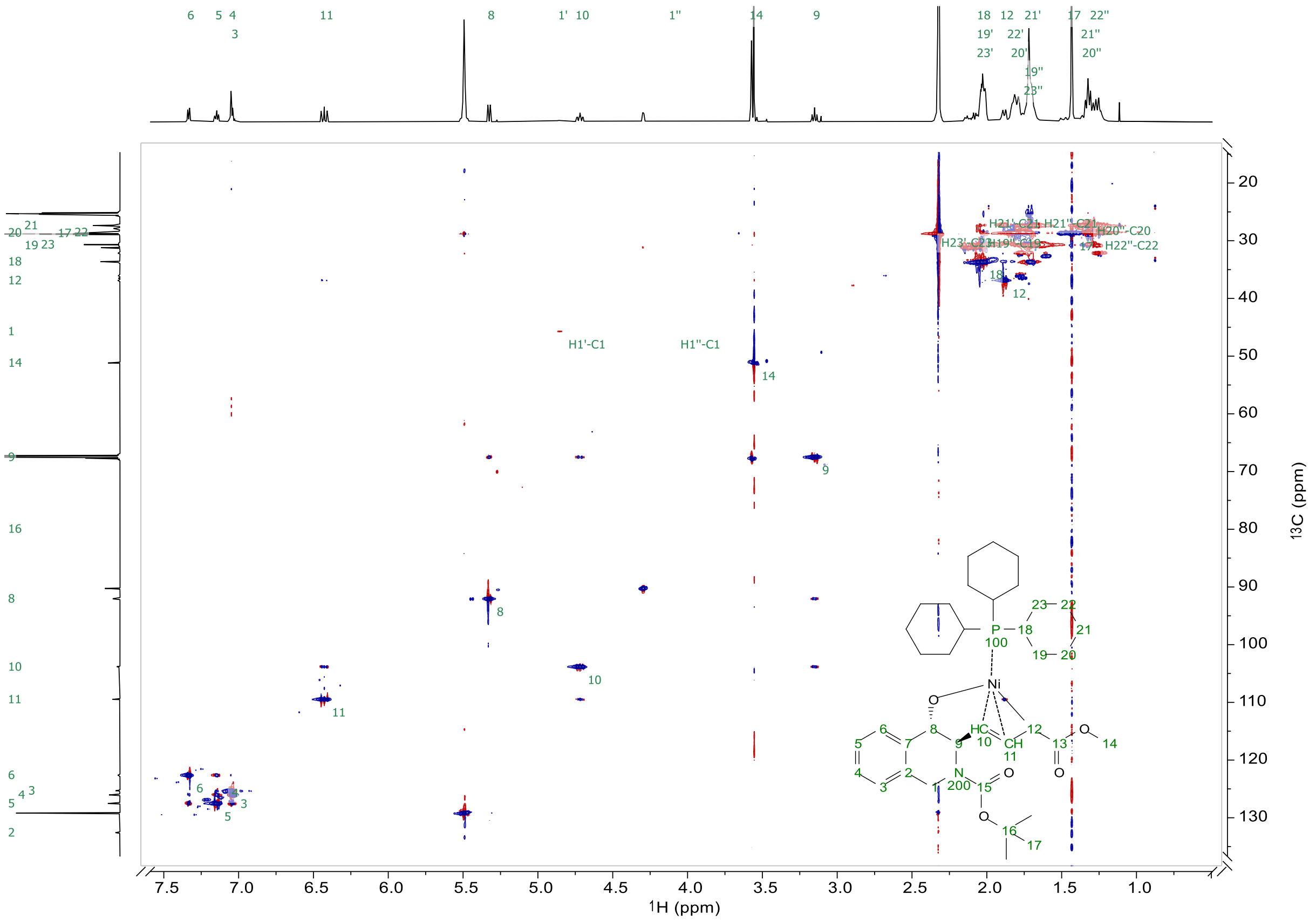

^{1}H-^{13}C HMBC NMR (600 MHz, 151 MHz,[D$_8$]-THF, 298 K) of complex **25a**

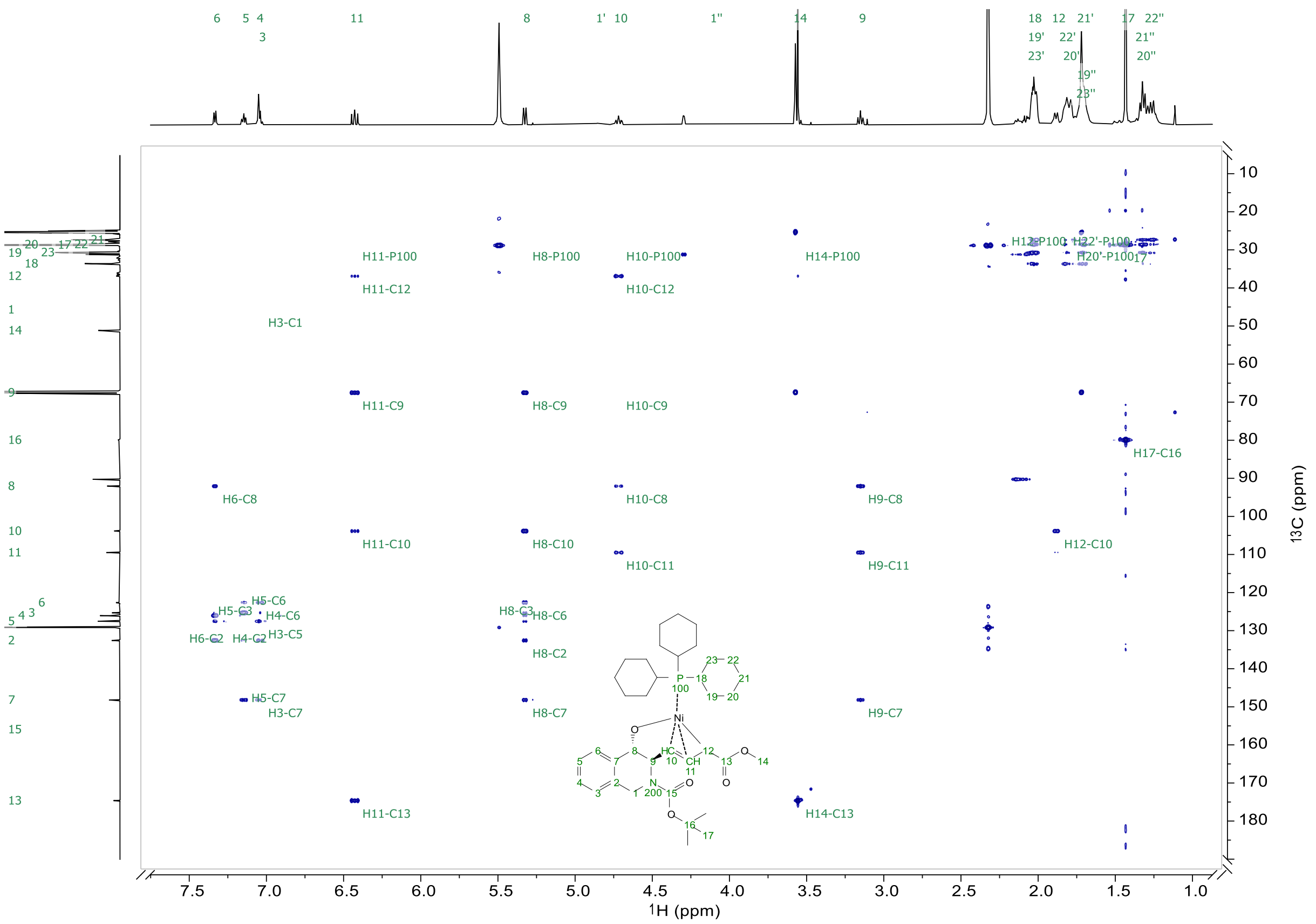

^1H-^1H COSY NMR (600 MHz,[D$_8$]-THF, 298 K) of complex **25a**

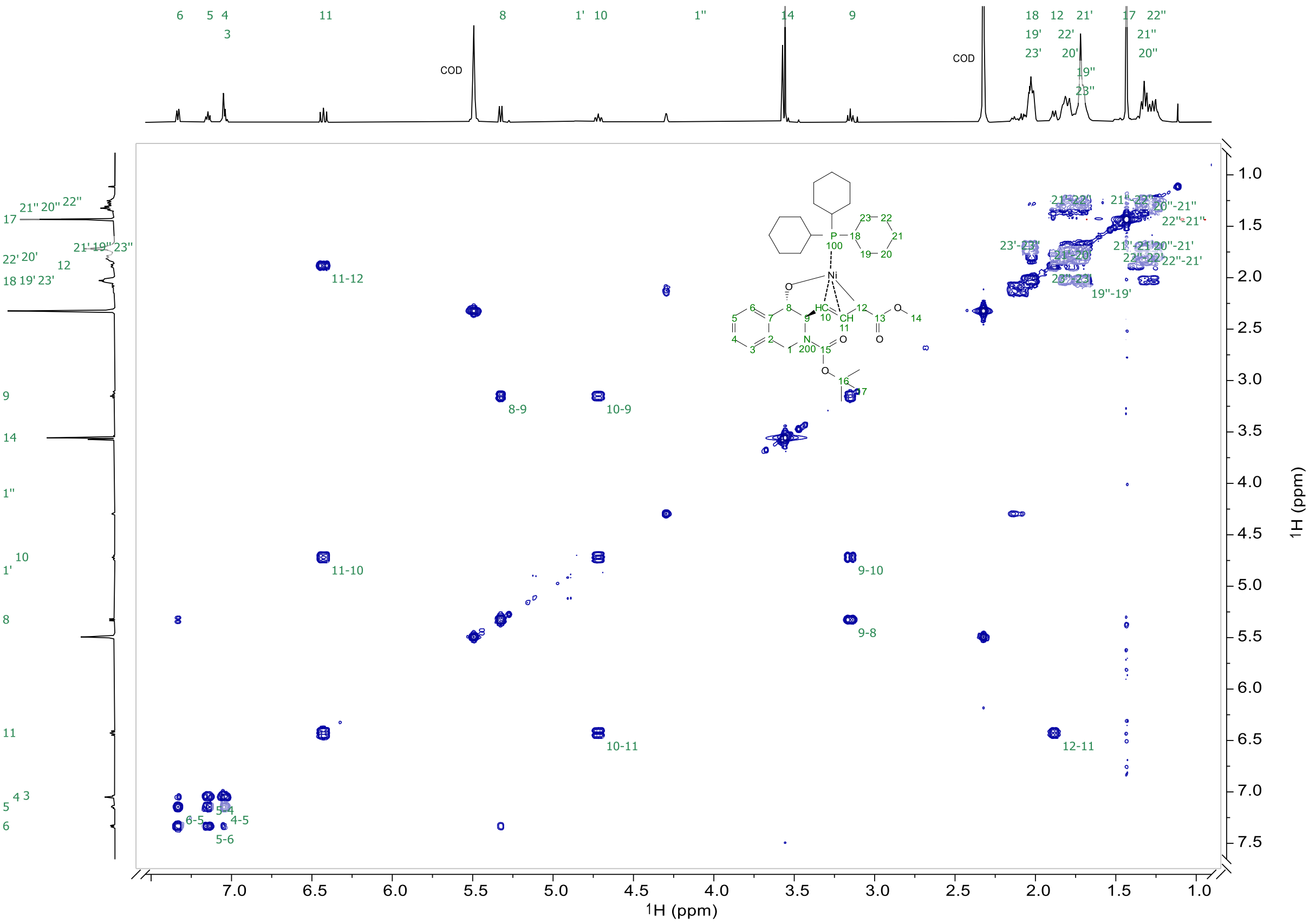

^1H-^1H NOESY NMR (600 MHz,[D$_8$]-THF, 298 K) of complex **25a**

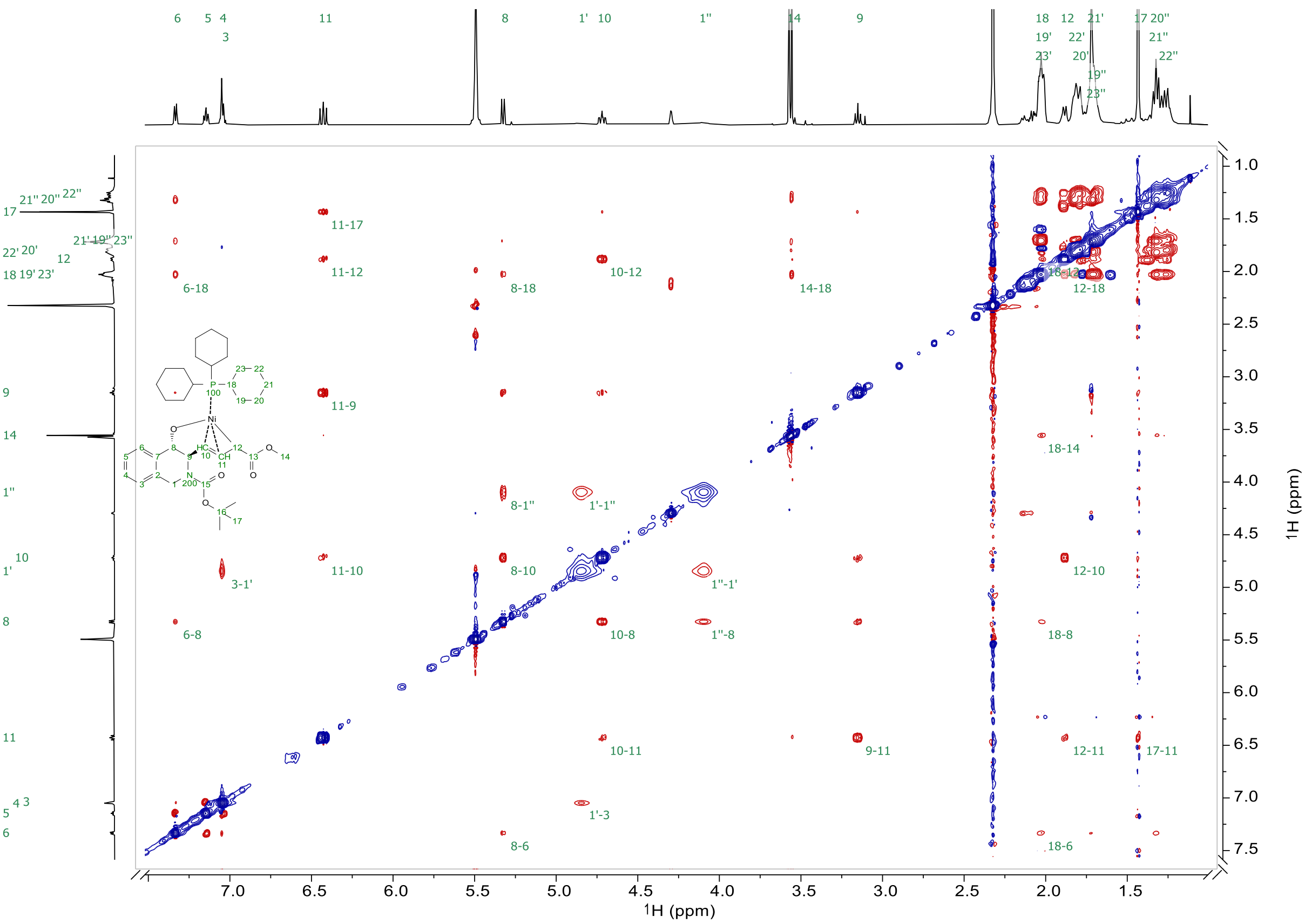

$^{31}P\{^{1}H\}$ NMR (243 MHz, [D$_8$]-THF, 298 K) and ^{1}H-^{31}P HMBC (600 MHz, 243 MHz, [D$_8$]-THF, 298 K) of complex **25a**

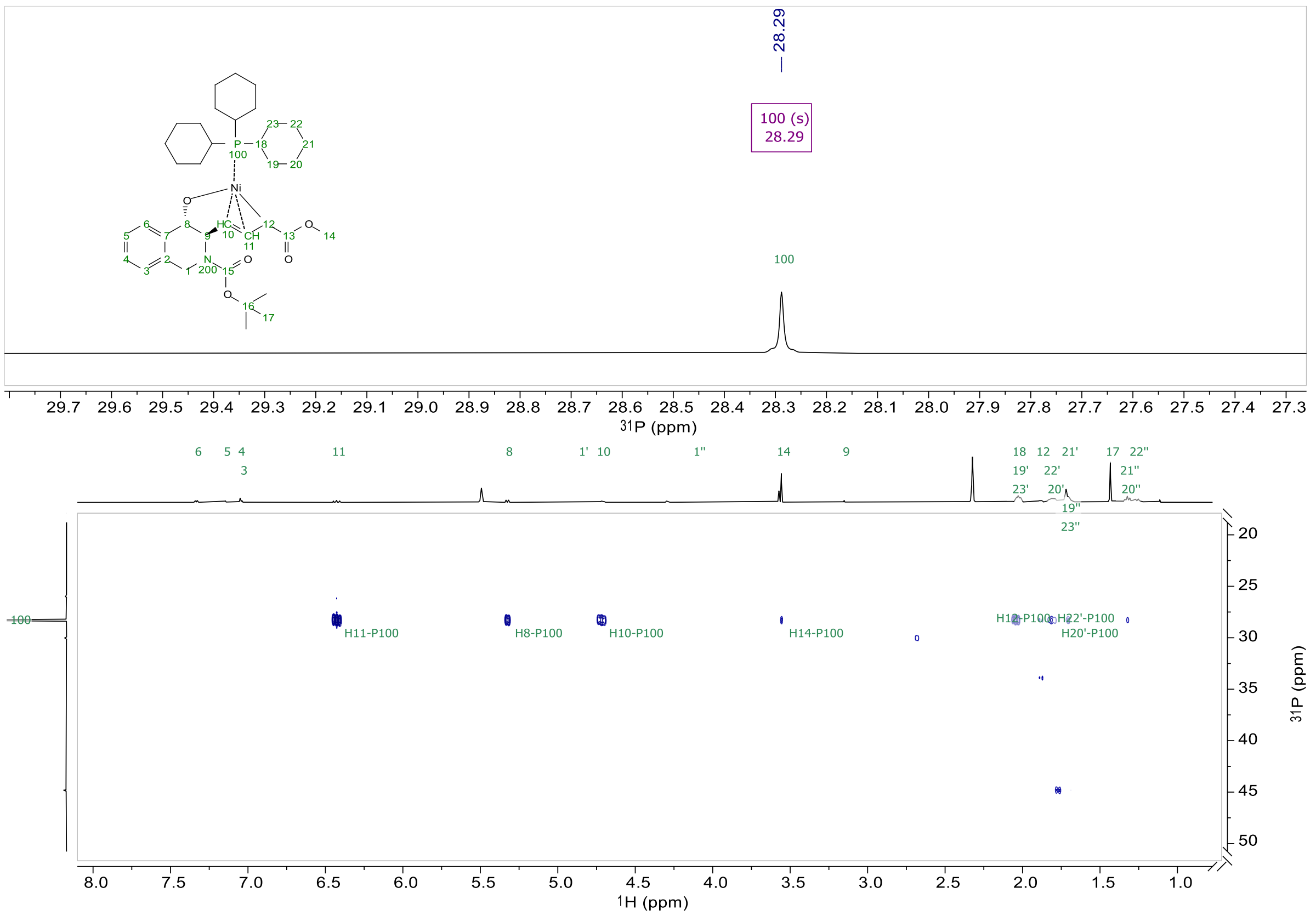

^{1}H-^{15}N HMBC (600 MHz, 62 MHz, [D$_8$]-THF, 298 K) of complex **25a**

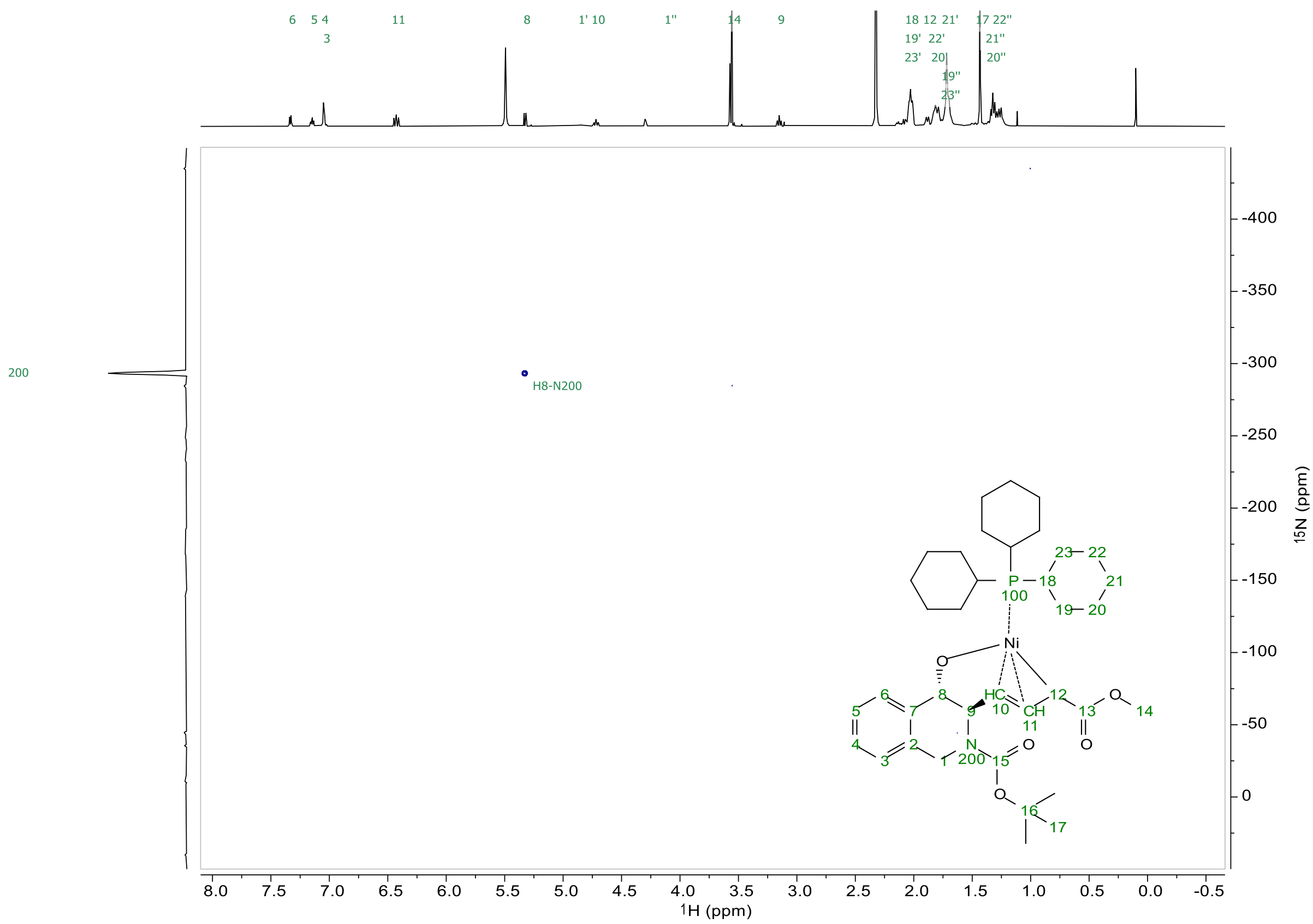

NMR Analysis of Complex 27.

A solution of ligand **L1** (24.0 mg, 65.0 µmol) in [D_8]-THF (0.8 mL) was added to bis-(1,5-cyclooctadiene)nickel(0) (17.0 mg, 61.8 µmol) and the resulting yellow-orange mixture was stirred at room temperature for 3 min. The solution was cooled to –40 °C (bath temperature) before methyl (2*E*,4*E*)-5-((*tert*-butoxycarbonyl)(2-formylbenzyl)amino)penta-2,4-dienoate **7a** (21.3 mg, 61.8 µmol) was added, causing a color change to red-brown. The mixture was stirred for 20 min at –40°C and then transferred into a J-Young NMR tube using a syringe filter and NMR spectra were recorded at 233 K. Pertinent spectra and their analysis are shown below.

The sample also contained free ligand, residual **7a**, and a complex of type [(**L1**)$_2$Ni(0)] in addition to two reactive intermediates **27a** and **27b** (= Int). The concentration of **27a**,**b** increased with time, whereas the amount of [(**L1**)$_2$Ni(0)] as well as of the starting material **7a** decreased.

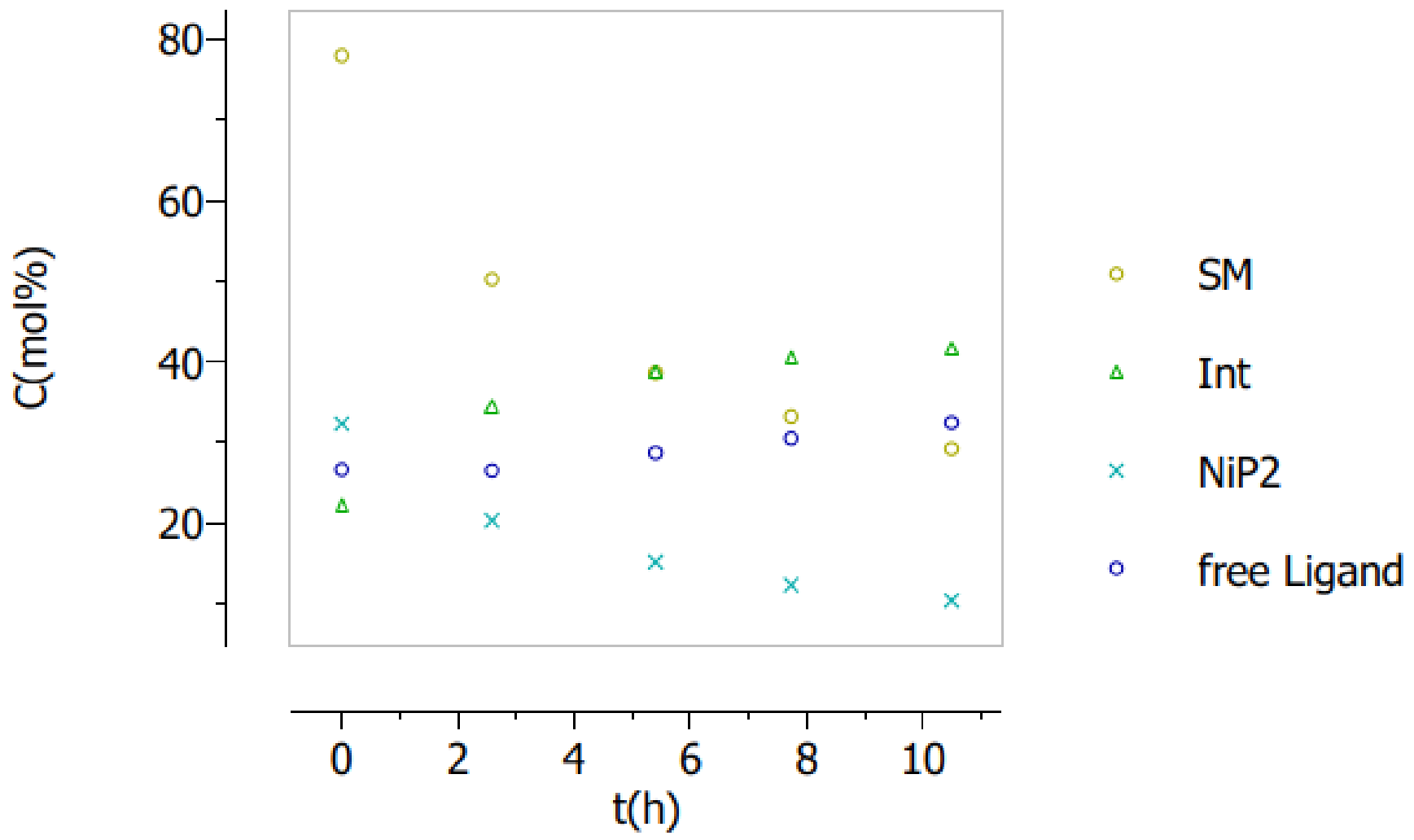

The most relevant signals of the isomeric reactive intermediates could be assigned based on the 1D and 2D NMR data. In regions of complex overlaps, signals were assigned based on 1D selective TOCSYs or the 2D NMR correlation experiments.

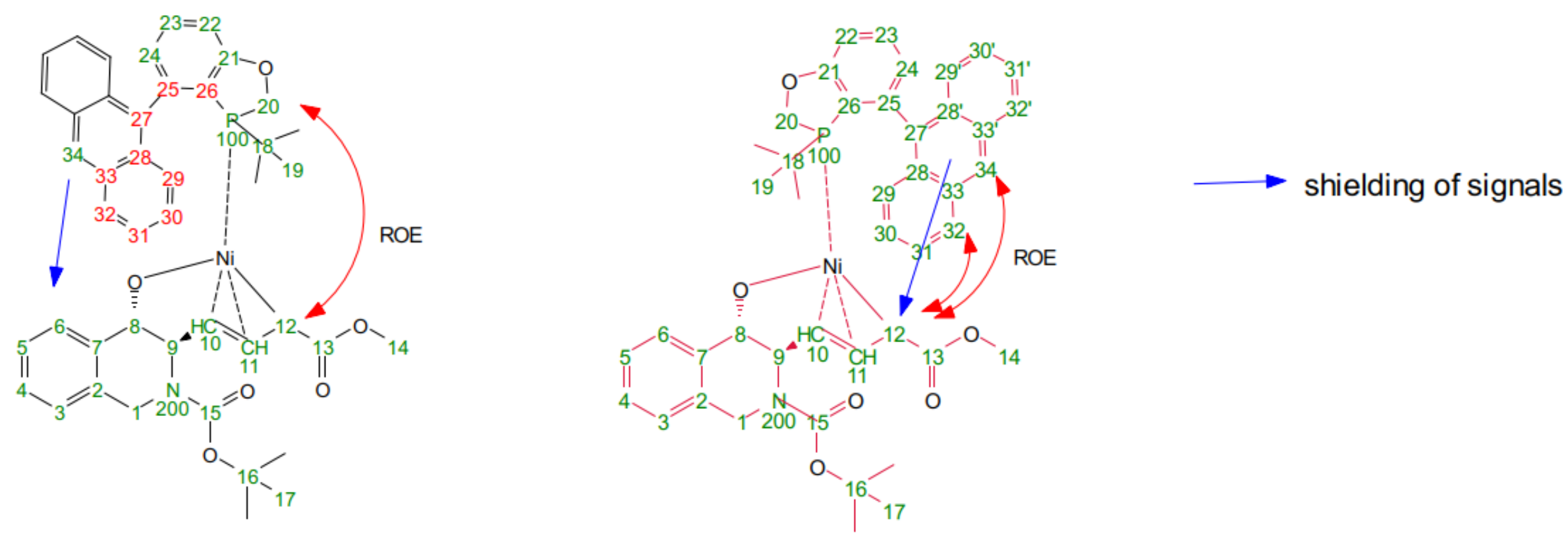

Arbitrary numbering scheme of the minor isomer **27b** (left) and the major isomer **27a** (right); relevant ROEs and shielding effects

The ^{13}C NMR chemical shifts of C8-C12 of both diastereomers were very similar to those observed for complex **25a** carrying a PCy_3 ligand (see above). We therefore conclude that the coordination of the allyl subunit is basically identical. Additionally, the coupling between H8 and H9 is large in both cases (~9.8 Hz), supporting the *trans*-configuration. Two large ^3J couplings between H11 on the one hand and H10 as well as H12a (~12±1 Hz) are also in good agreement with what had been observed for **25a**.

However, certain signals, most notably H12, differ significantly from each other in the two diastereomers **27a** and **27b**. H12 is shifted from 1.90 ppm in the minor isomer to –0.44 ppm in the major isomer. Based on an ROE from H12 to H34 and H32 we attribute this effect to a different spatial positioning of the ligand's anthracene unit. The anisotropy caused by the aromatic ring current results in a drastic shielding of this nucleus in the major isomer **27a**, which must be positioned exactly above the ring. A similar, but less drastic, effect is observed for H6 of the minor isomer **27b** (6.25 ppm vs 7.26 ppm).

Additionally, H10 and H12 show significantly different ^3J couplings to P-100 in the two isomers. In the major diastereomer **27a**, H10 has a large ^{31}P coupling constant of 5.4 Hz; in the minor diastereomer **27b**, it is H12 in which shows a ^{31}P coupling constant of 3.6 Hz.

Major Isomer (27a): Signal Assignments

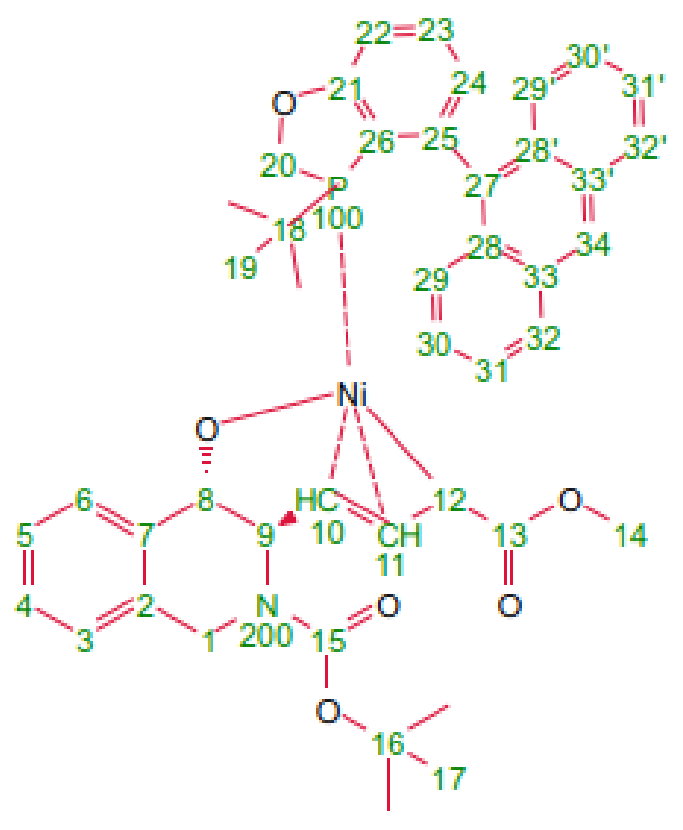

Atom	δ (ppm)	J	COSY	HSQC	HMBC	ROESY
1 C	45.194			1', 1''	3	
H'	4.907	14.50(1'')	1''	1	2, 3, 7, 9, 15	1''
H''	4.153	14.50(1')	1'	1	2, 3, 7, 15	1', 8
2 C	132.549				1', 1'', 4, 6, 8	
3 C	125.573			3	1', 1'', 5	
H	7.127		4	3	1, 5, 7	
4 C	126.381			4	6	
H	7.095		3, 5	4	2, 6	
5 C	127.571			5	3	
H	7.215		4, 6	5	3, 7	
6 C	122.477			6	4, 8	
H	7.257		5	6	2, 4, 8	
7 C	147.314				1', 1'', 3, 5, 8, 9	
8 C	90.780	10.60(100)		8	6, 9, 10	
H	5.436	9.80(9)	9	8	2, 6, 7, 9, 10	1'', 10, 30
9 C	66.645	2.40(100)		9	1', 8, 10, 11	
H	2.988	9.80(8), 10.10(10)	8, 10	9	7, 8, 11, 200	11
10 C	101.012	13.10(100)		10	8, 11, 12	
H	3.544	5.40(100P), 10.10(9), 1.60(12), 12.20(11)	9, 11	10	8, 9, 11, 12, 200	8, 11, 12, 30, 31
11 C	111.576			11	9, 10	
H	6.256	12.20(10), 11.90(12)	10, 12	11	9, 10, 13, 200	9, 10, 17
12 C	40.573	4.20(100)		12	10	
H	-0.442	0.90(100P), 1.60(10), 11.90(11)	11	12	10	10, 32, 34
13 C	174.137	1.40(100)			11, 14	

Atom	δ (ppm)	J	COSY	HSQC	HMBC	ROESY
14 C	50.481			14		
H3	2.794			14	13	
15 C	155.781				1', 1''	
16 C	80.089				17	
17 C	28.489			17	17	
H3	1.345			17	16, 17	11
18 C	33.113	15.00(100)			19, 20', 20''	
19 C	25.585			19	19	
H3	0.537			19	18, 19, 200	20'', 29'
20 C	65.654	13.20(100)		20', 20''		
H'	5.644	3.40(100P), 12.50(20'')	20''	20	18, 200	
H''	4.802	1.30(100P), 12.50(20')	20'	20	18, 21, 200	19
21 C	165.850	5.00(100)			20'', 22, 23	
22 C	112.461			22		
H	7.216		23	22	21, 24, 26, 200	
23 C	134.099			23		
H	7.693		22, 24	23	21, 25, 200	
24 C	126.493	6.10(100)			22	
H	7.110		23		27, 200	
25 C					23	
26 C	119.363	29.70(100)			22	
27 C	135.733	1.90(100)			24, 29, 29'	
28 C	130.903				30, 32	
28' C	131.367				30', 32'	

Atom	δ (ppm)	J	COSY	HSQC	HMBC	ROESY
29 C	127.970			29	31	
H	8.359		30	29	27, 31, 33, 200	
29' C	129.296			29'	31'	
H	7.912		30'	29'	27, 31', 33'	19
30 C	127.613			30		
H	7.859		29, 31	30	28, 32	8, 10
30' C	126.578			30'		
H	7.434		29', 31'	30'	28', 32'	
31 C	126.136			31	29	
H	7.669		30, 32	31	29, 33	10
31' C	125.965			31'	29'	
H	7.490		30', 32'	31'	29', 33'	
32 C	130.146			32	30	
H	8.170		31	32	28, 33	12, 34
32' C	129.663			32'	30'	
H	8.155		31'	32'	28', 33'	34
33 C	132.267				29, 31, 32	
33' C	132.904				29', 31', 32'	
34 C	129.278			34		
H	8.691			34		12, 32, 32'
100 P	27.686	1.40(13), 5.00(21), 1.90(27), 4.20(12), 15.00(18), 13.20(20), 2.40(9), 10.60(8), 29.70(26), 6.10(24), 13.10(10), 0.90(12H), 3.40(20'), 1.30(20''), 5.40(10H)				
200 N	-293.594				9, 10, 11, 19, 20', 20'', 22, 23, 24, 29	

Minor Isomer (27b): Signal Assignments

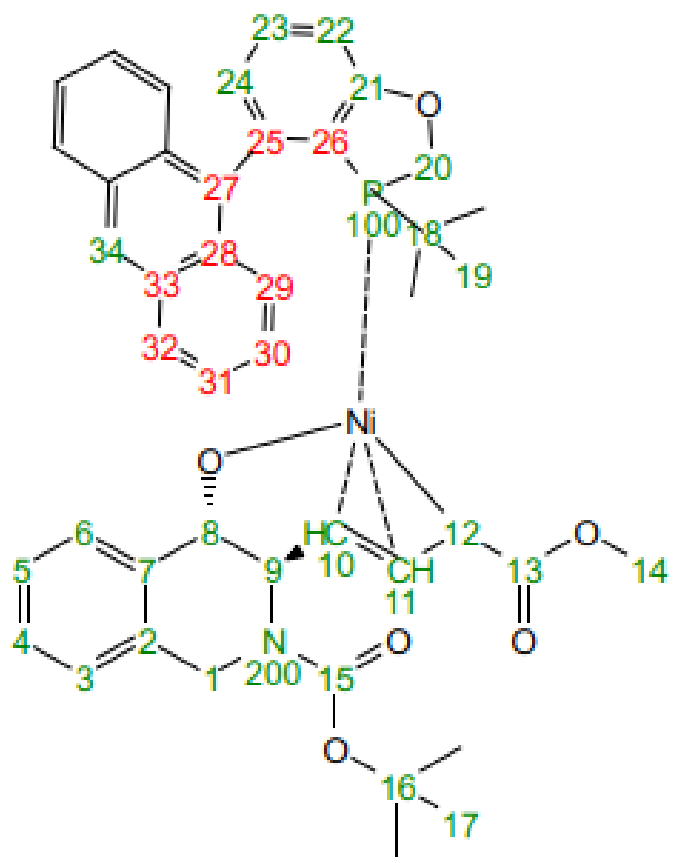

Atom	δ (ppm)	J	COSY	HSQC	HMBC	ROESY
1 C	45.150			1', 1"	3	
H'	4.764	14.80(1")	1"	1	2, 3, 7, 9, 15	1"
H"	3.841	14.80(1')	1'	1	2, 3, 7, 9, 15	1', 8
2 C	131.908				1', 1", 4, 6, 8	
3 C	124.938			3	1', 1", 5	
H	7.011		4	3	1, 5, 7	
4 C	125.952			4	6	
H	7.091		3, 5	4	2, 6	
5 C	127.078			5	3	
H	7.264		4, 6	5	3, 7	
6 C	123.440			6	4, 8	
H	6.253		5	6	2, 4, 8	8
7 C	147.176				1', 1", 3, 5, 8, 9	
8 C	90.844	8.50(100)		8	6, 9, 10	
H	4.754	9.80(9)	9	8	2, 6, 7, 9, 10, 100, 200	1", 6, 10
9 C	65.664	1.30(100)		9	1', 1", 8, 10, 11	
H	2.441	9.80(8), 9.90(10)	8, 10	9	7, 8, 11	11
10 C	103.342	10.70(100)		10	8, 11, 12	
H	4.764	12.90(11), 1.00(12), 9.90(9)	9, 11	10	8, 9, 12	8, 11, 12
11 C	112.878			11	9	
H	6.461	1.60(100P), 12.90(10), 11.20(12)	10, 12	11	9, 10, 13, 100	9, 10, 17
12 C	39.173	4.30(100)		12	10	
H	1.900	3.60(100P), 1.00(10), 11.20(11)	11	12	10, 100	10, 20"
13 C	173.754	1.40(100)			11, 14	
14 C	51.772			14		
H3	3.417			14	13	
15 C	155.839				1', 1"	
16 C	80.013				17	
17 C	28.697			17	17	
H3	1.492			17	16, 17	11

Atom	δ (ppm)	J	COSY	HSQC	HMBC	ROESY
18 C	35.410	12.90(100)			20', 20"	
19 C	25.616			19		
H3	0.687			19		20'
20 C	69.974	16.20(100)		20', 20"		
H'	5.237			20	18, 21, 100	19
H"	5.086			20	18, 100	12
21 C	165.685				20'	
22 C						
H	7.132		23		100	
23 C						
H	7.643		22, 24		100	
24 C						
H	7.074		23		100	
25 C						
26 C						
27 C						
28 C						
29 C						
H						
30 C						
H						
31 C						
H						
32 C						
H						
33 C						
34 C						
H	8.532					
100 P	17.927	1.60(11H), 1.40(13), 4.30(12), 10.70(10), 8.50(8), 16.20(20), 1.30(9), 3.60(12H), 12.90(18)			8, 11, 12, 20', 20", 22, 23, 24	
200 N	-293.594				8	

1H{off},1D, 600.23 MHz,THF,233.0K, pulse sequence: zg30

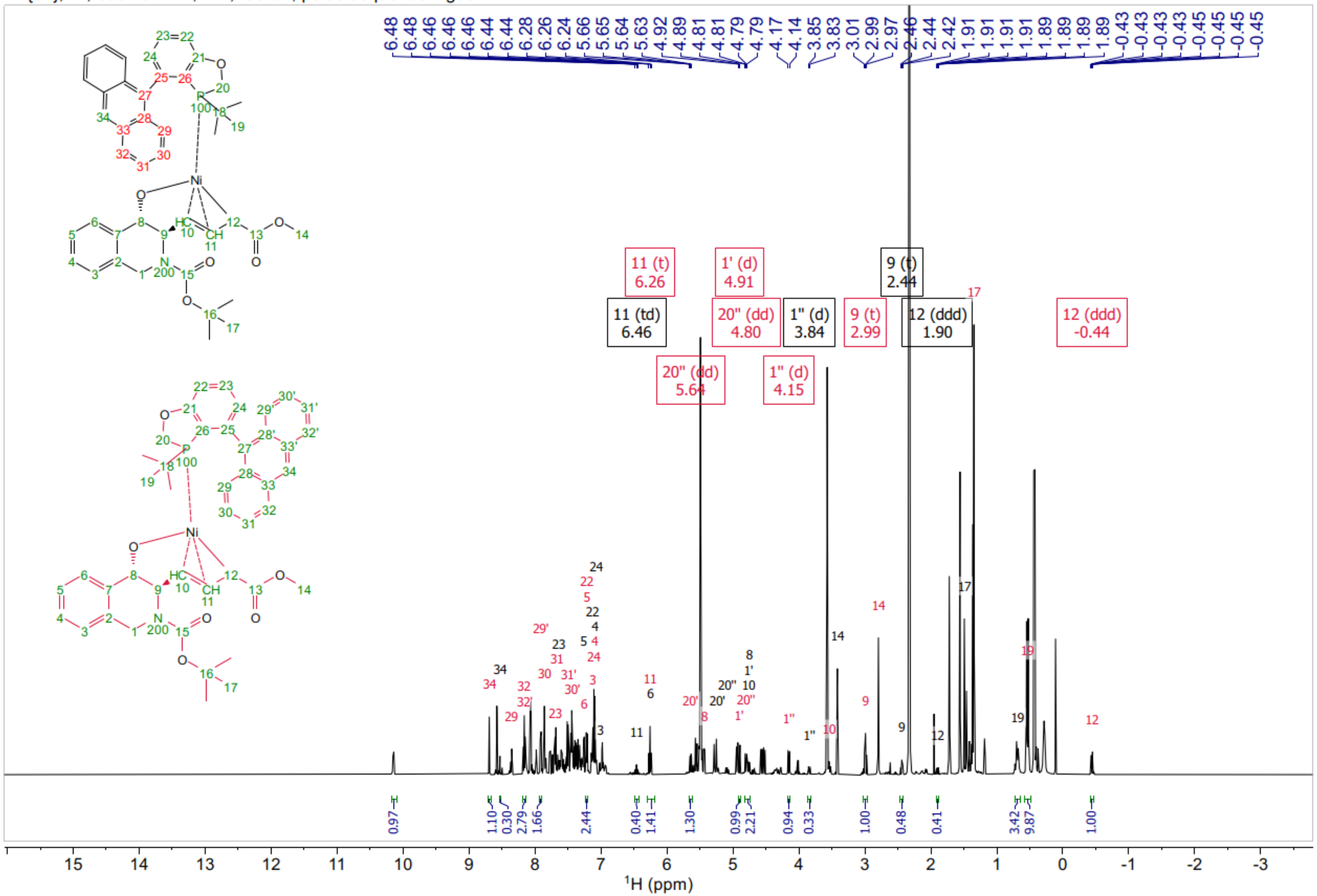

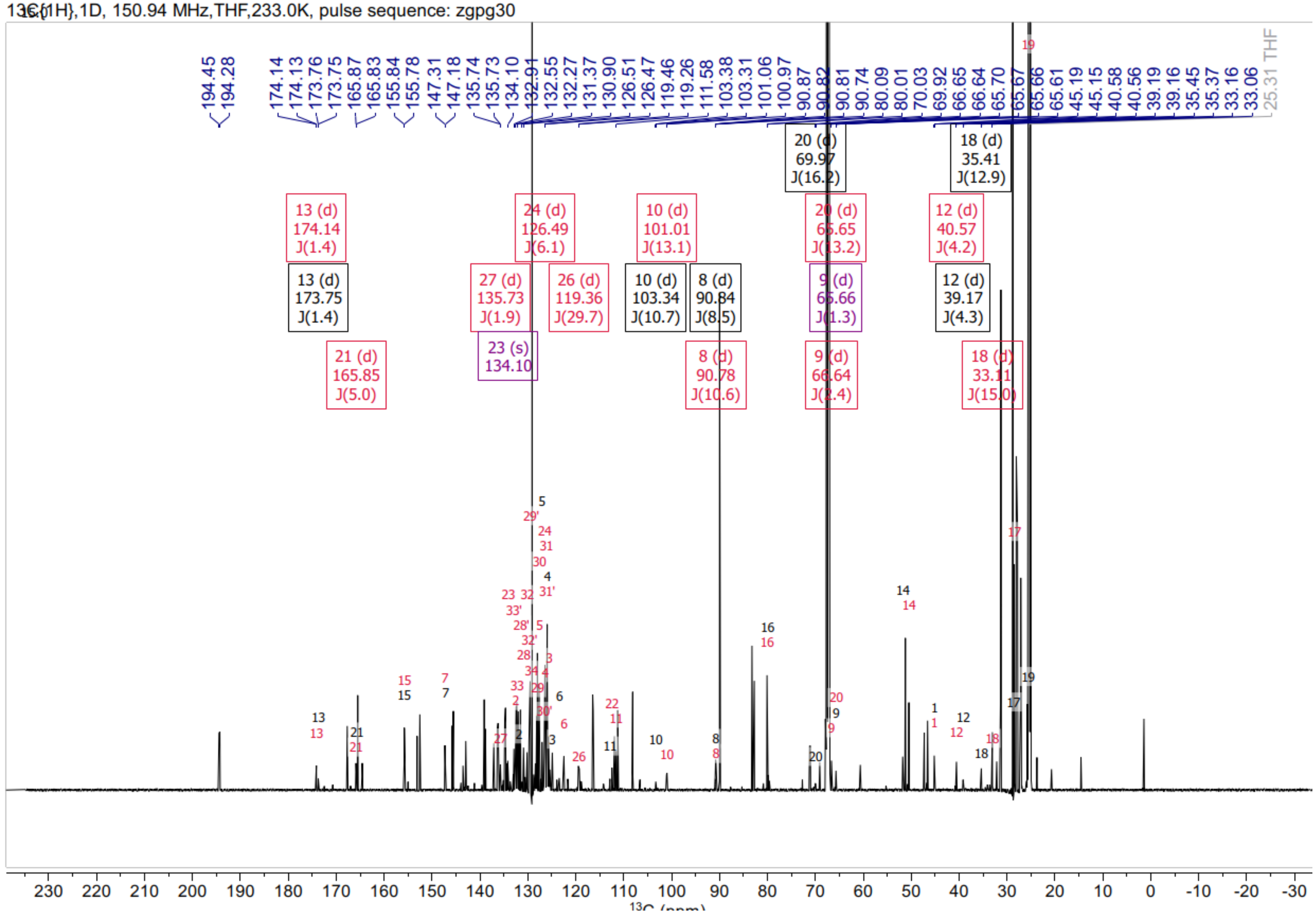
13C{1H},1D, 150.94 MHz,THF,233.0K, pulse sequence: zgpg30
13 (d) 174.14 J(1.4)
13 (d) 173.75 J(1.4)
21 (d) 165.85 J(5.0)
24 (d) 126.49 J(6.1)
27 (d) 135.73 J(1.9)
26 (d) 119.36 J(29.7)
23 (s) 134.10
10 (d) 101.01 J(13.1)
10 (d) 103.34 J(10.7)
8 (d) 90.84 J(8.5)
8 (d) 90.78 J(10.6)
20 (d) 69.97 J(16.2)
20 (d) 65.65 J(13.2)
9 (d) 65.66 J(1.3)
9 (d) 66.64 J(2.4)
18 (d) 35.41 J(12.9)
12 (d) 40.57 J(4.2)
12 (d) 39.17 J(4.3)
18 (d) 33.11 J(15.0)
25.31 THF
13C (ppm)

1H{13C},HSQC-EDITED, 600.23 MHz,THF,233.0K, pulse sequence: hsqcedetgpsisp2.4

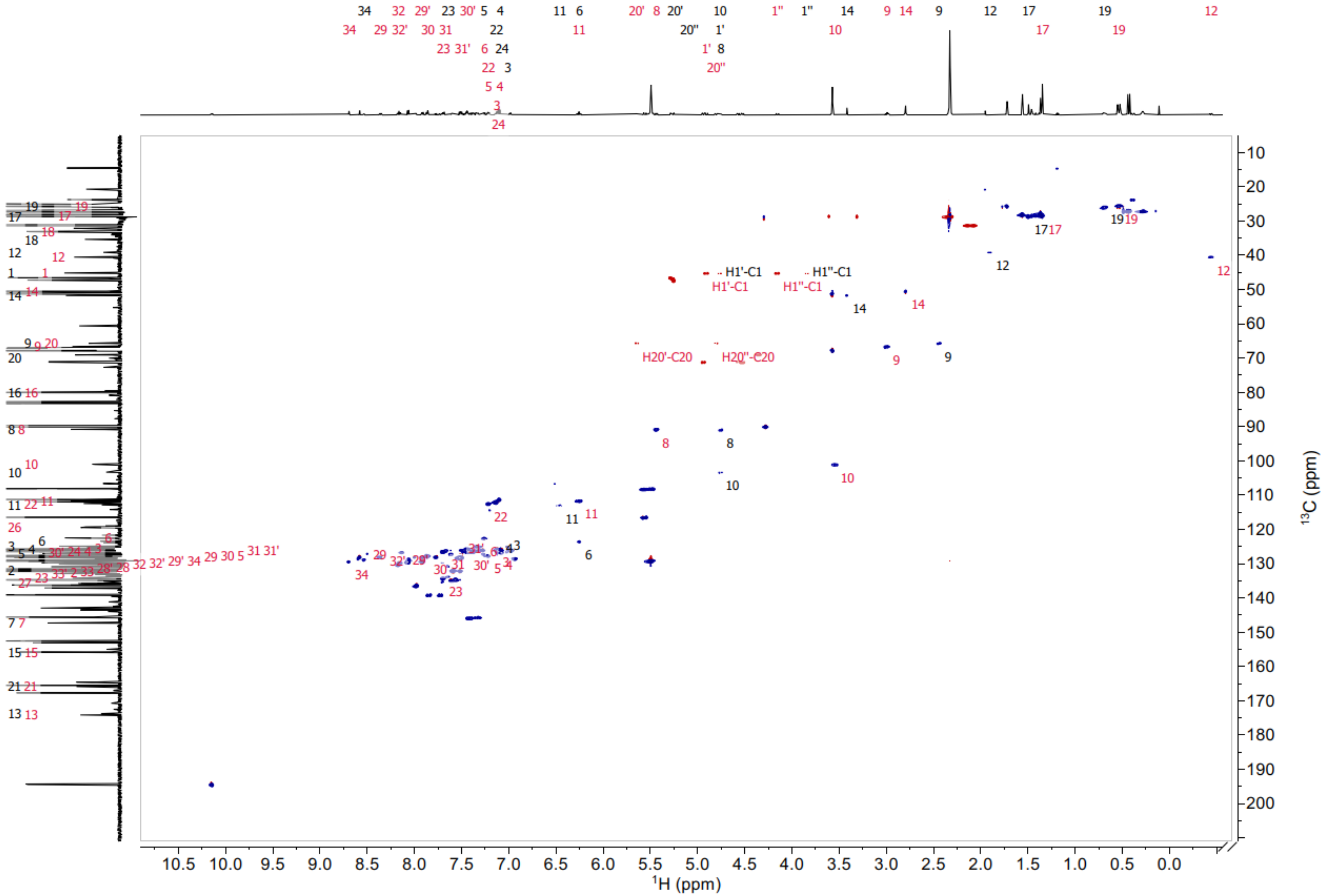

1H{13C},HMBC, 600.23 MHz,THF,233.0K, pulse sequence: hmbcetgpl3nd

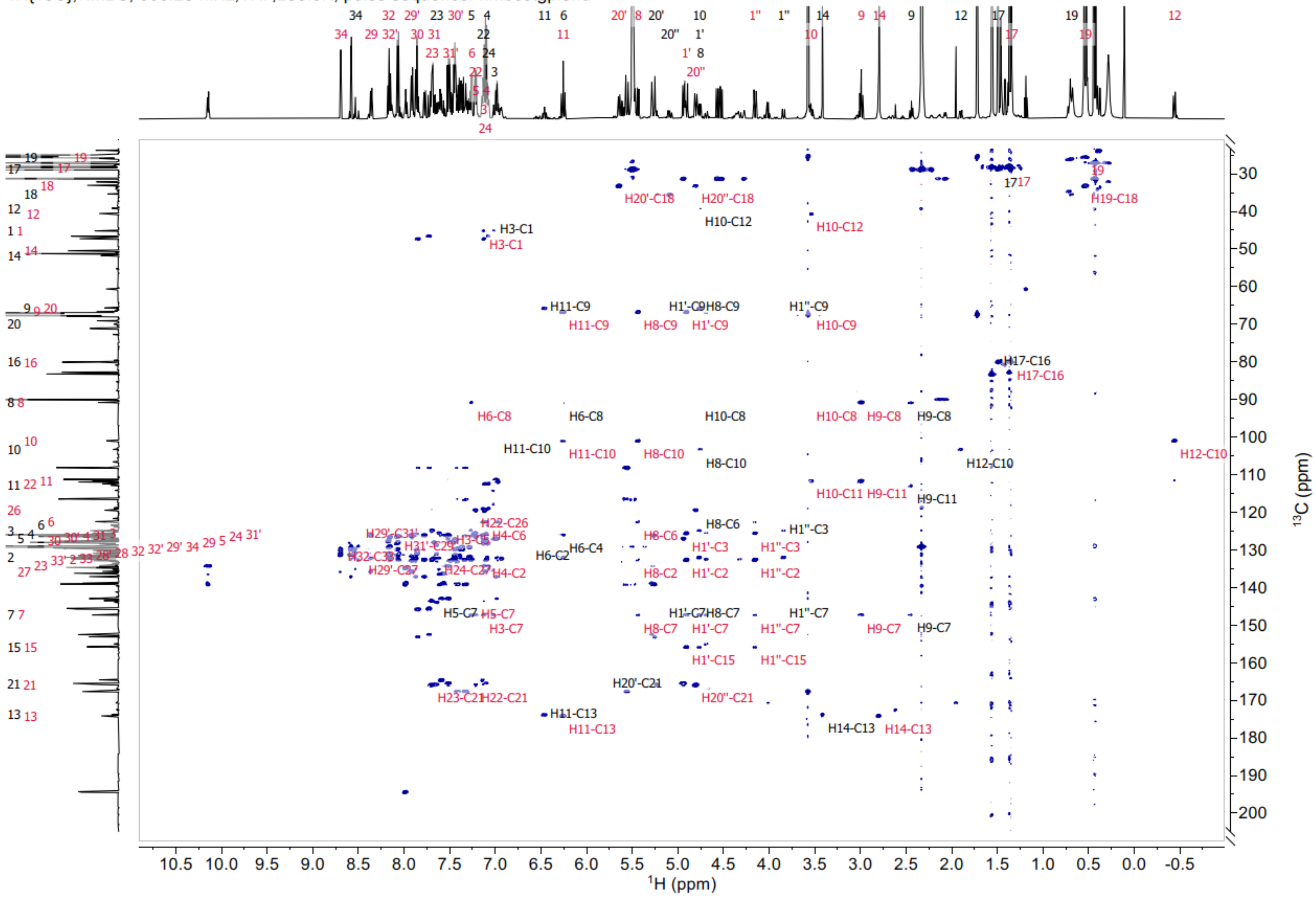

31P{1H},1D, 242.97 MHz,THF,233.0K, pulse sequence: zgpg30

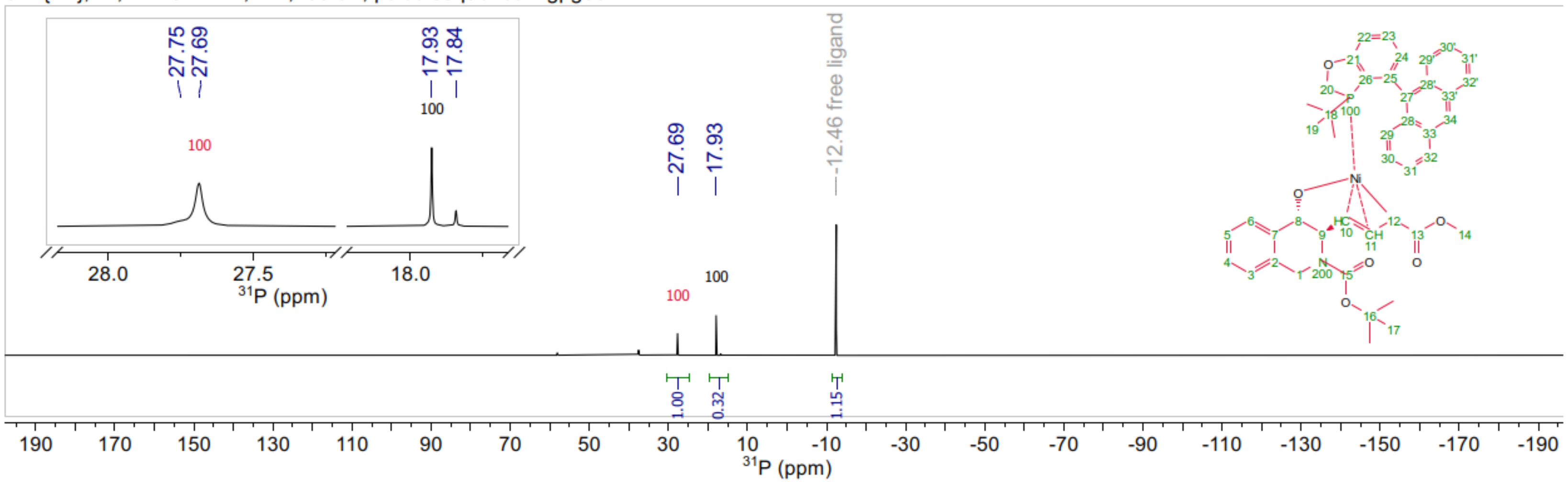

1H{31P},HMBC, 600.23 MHz,THF,233.0K, pulse sequence: hmbcgpndqf

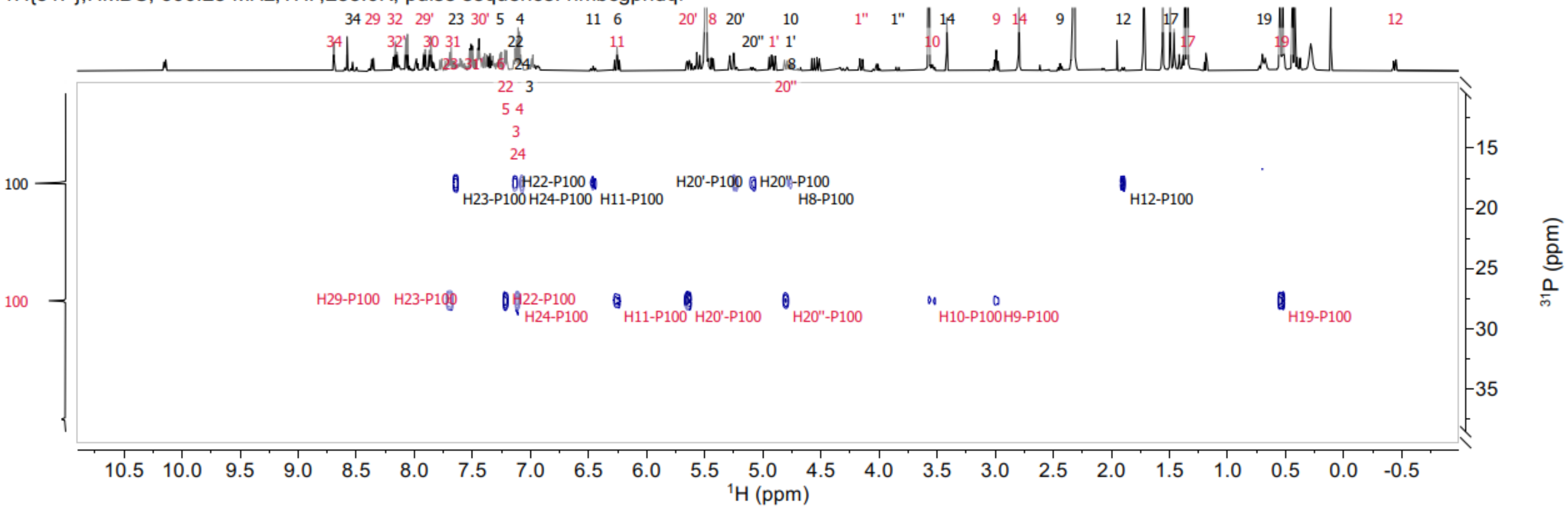

1H{off},COSY, 600.23 MHz,THF,233.0K, pulse sequence: cosygpppqf

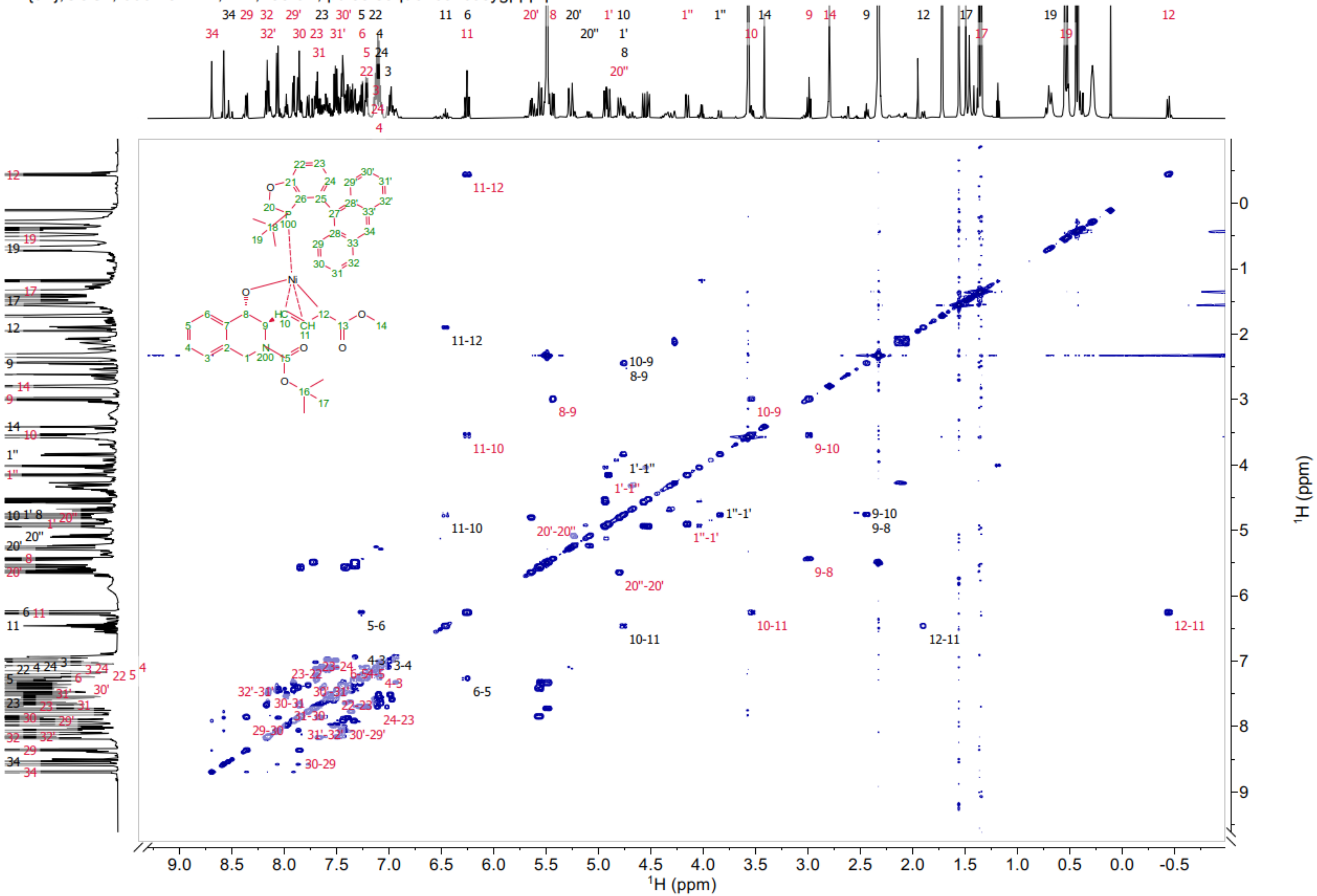

1H{off},ROESY, 600.23 MHz,THF,233.0K, pulse sequence: roesyadjsphpr

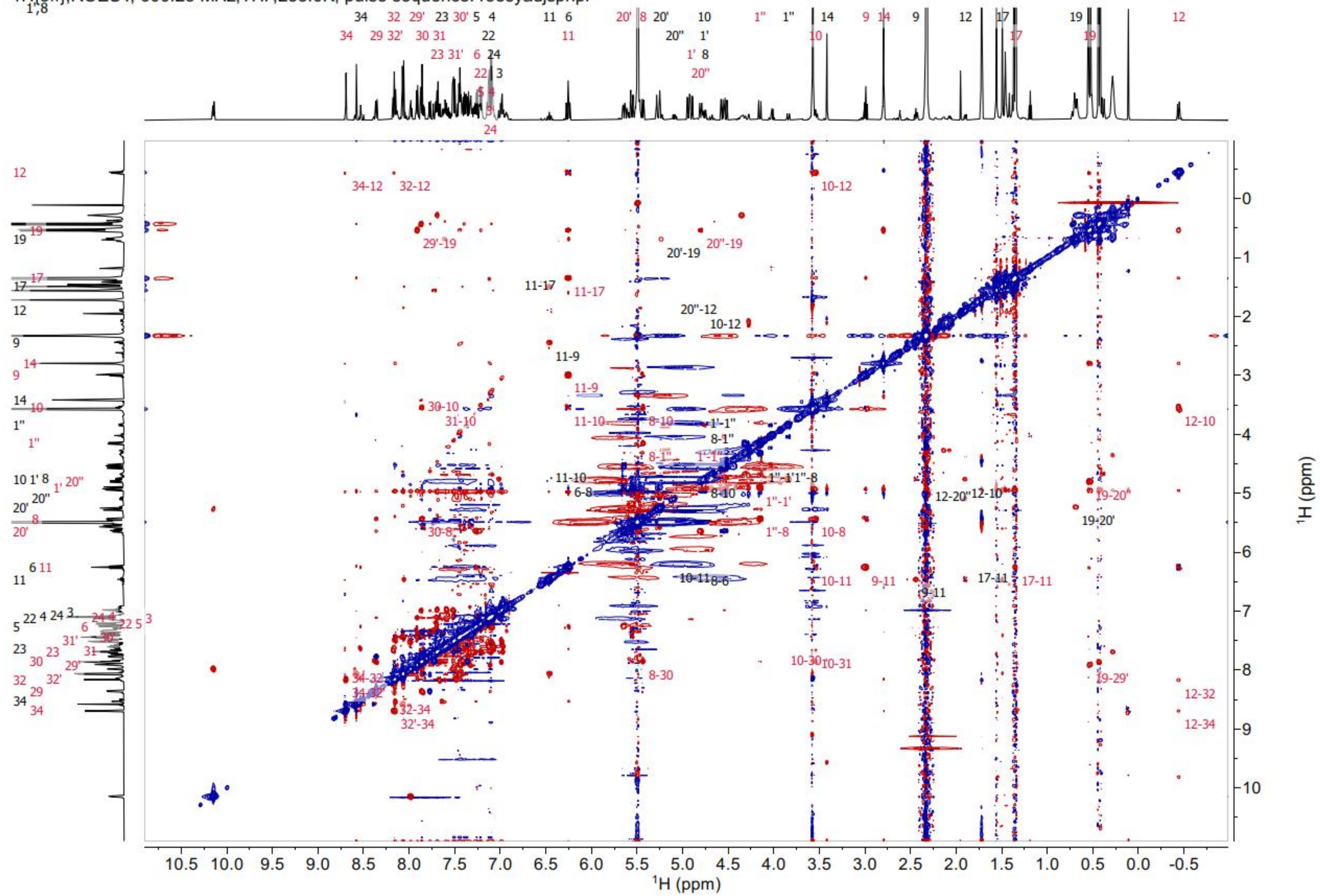

13C{1H},DEPT-135, 150.94 MHz,THF,233.0K, pulse sequence: deptsp135

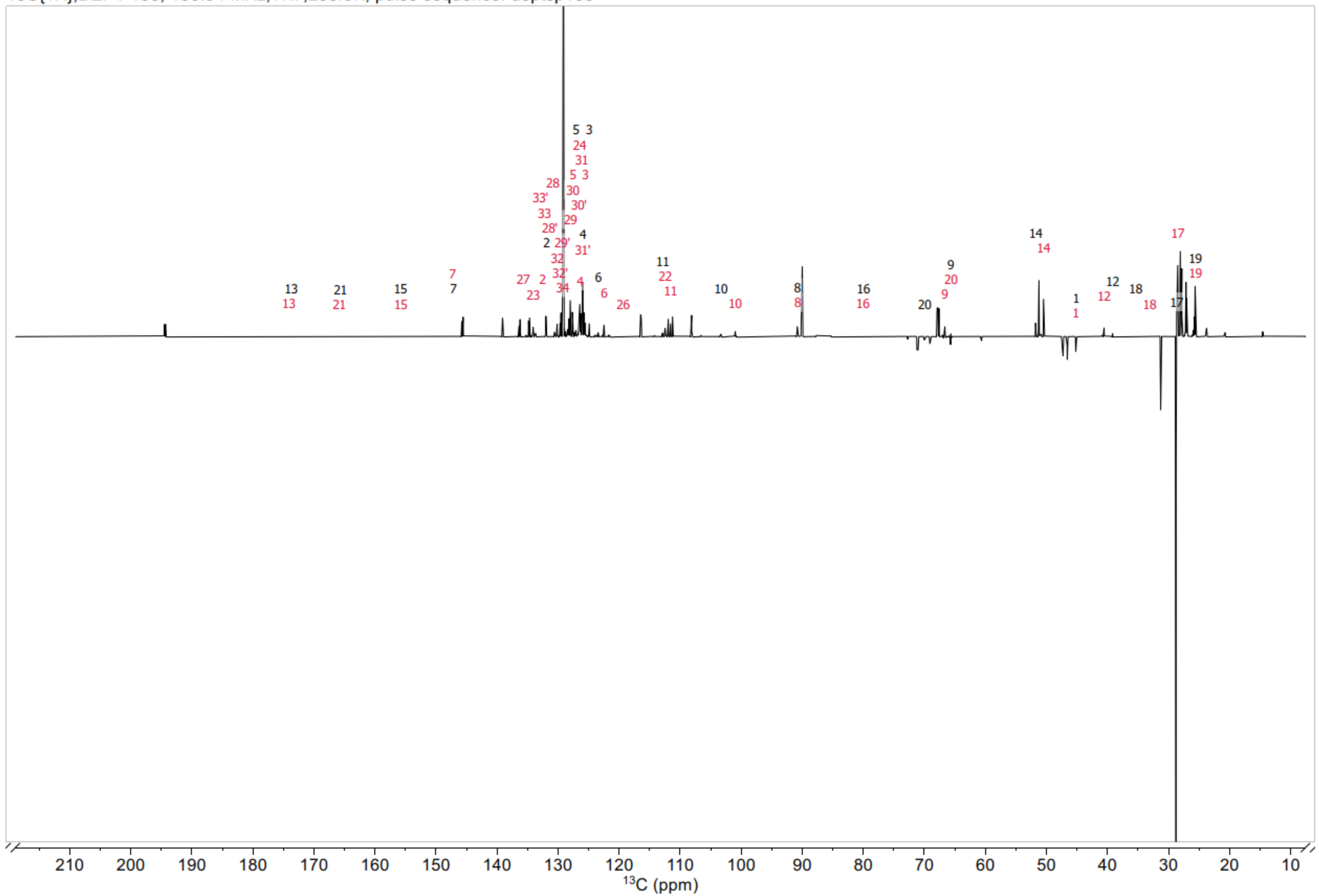

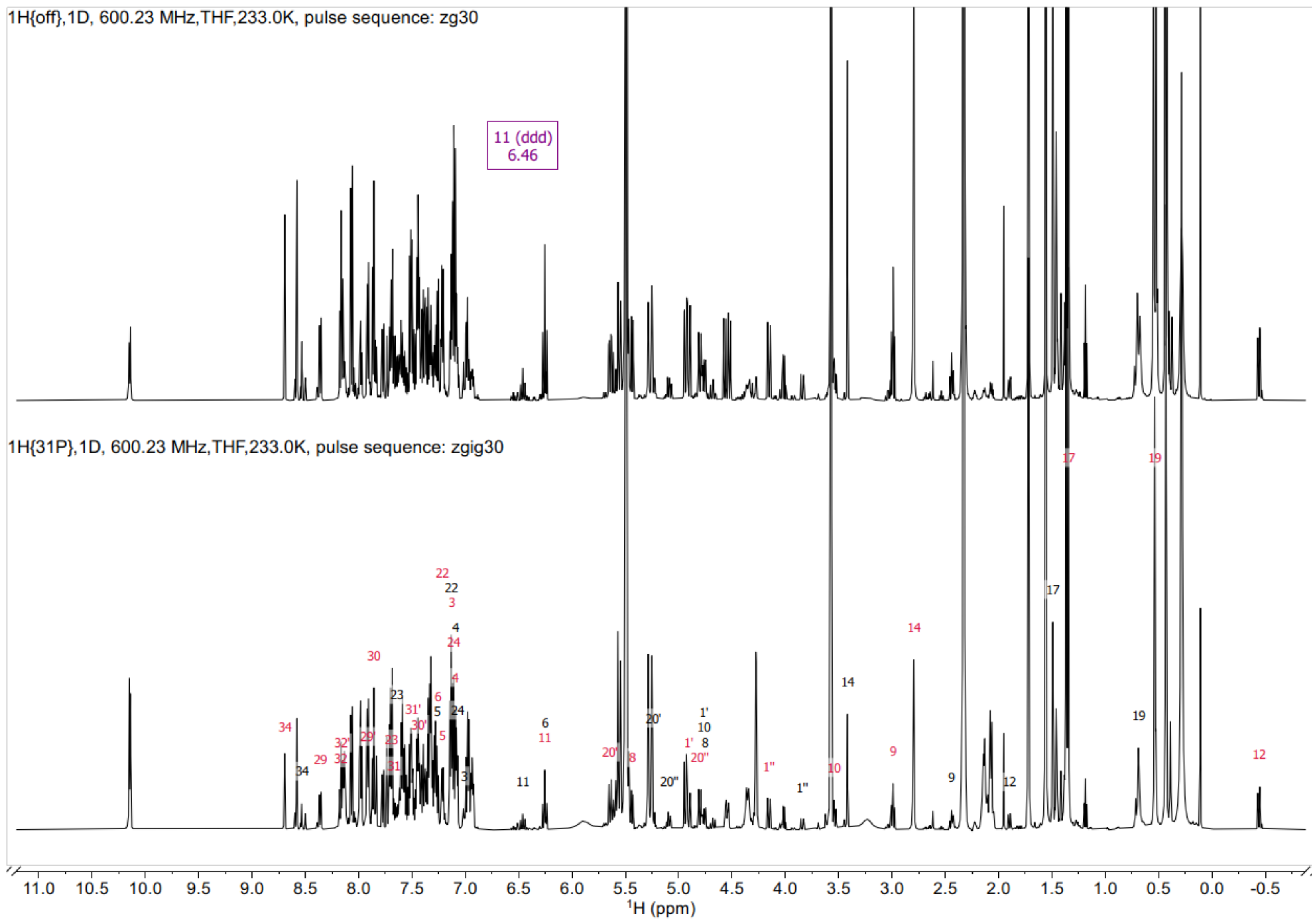
1H{off},1D, 600.23 MHz,THF,233.0K, pulse sequence: zg30
11 (ddd)
6.46
1H{31P},1D, 600.23 MHz,THF,233.0K, pulse sequence: zgig30
^{1}H (ppm)

1H{off},1D, 600.23 MHz,THF,233.0K, pulse sequence: selcssfdizs

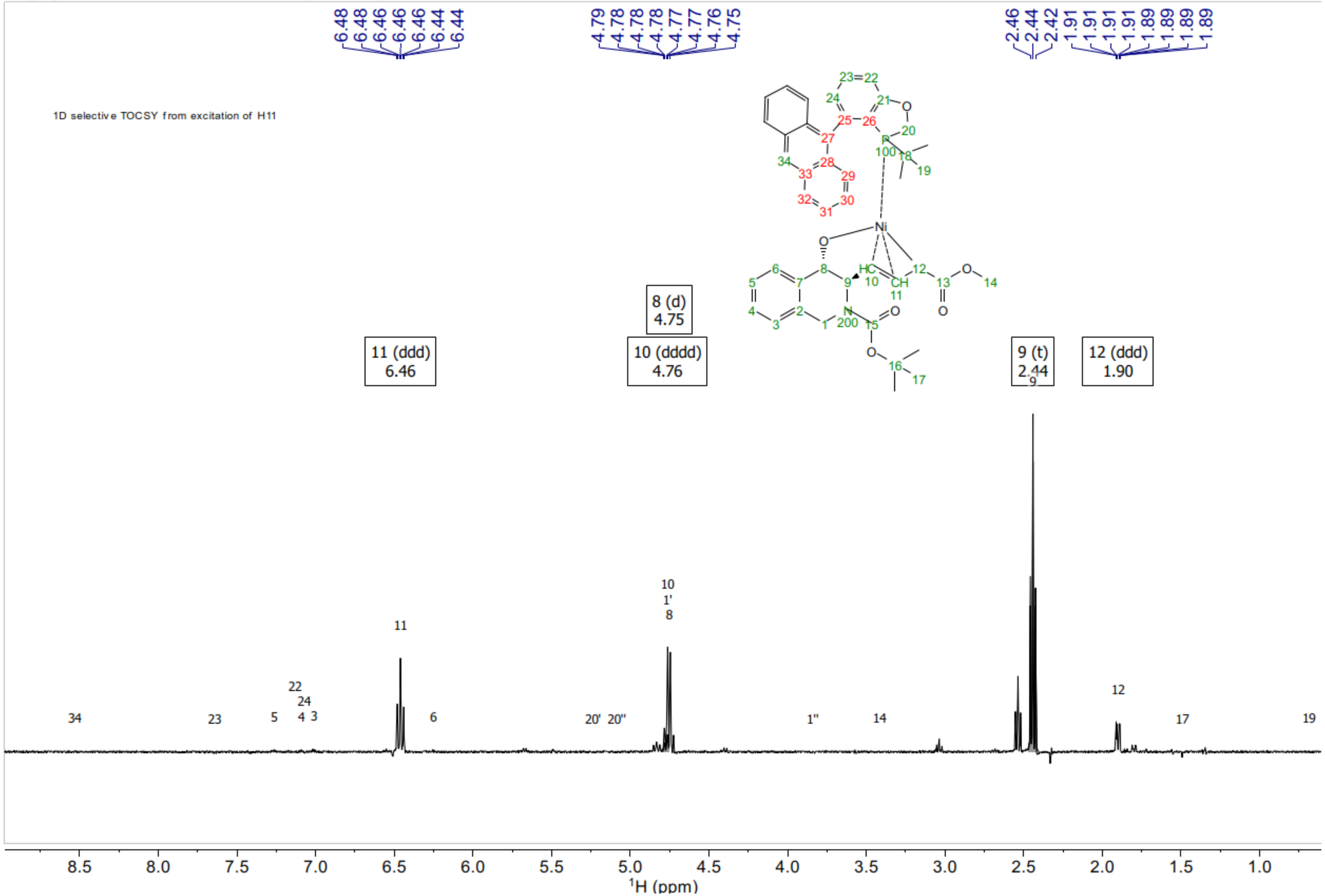

1H{off},1D, 600.23 MHz,THF,233.0K, pulse sequence: selcssfdizs

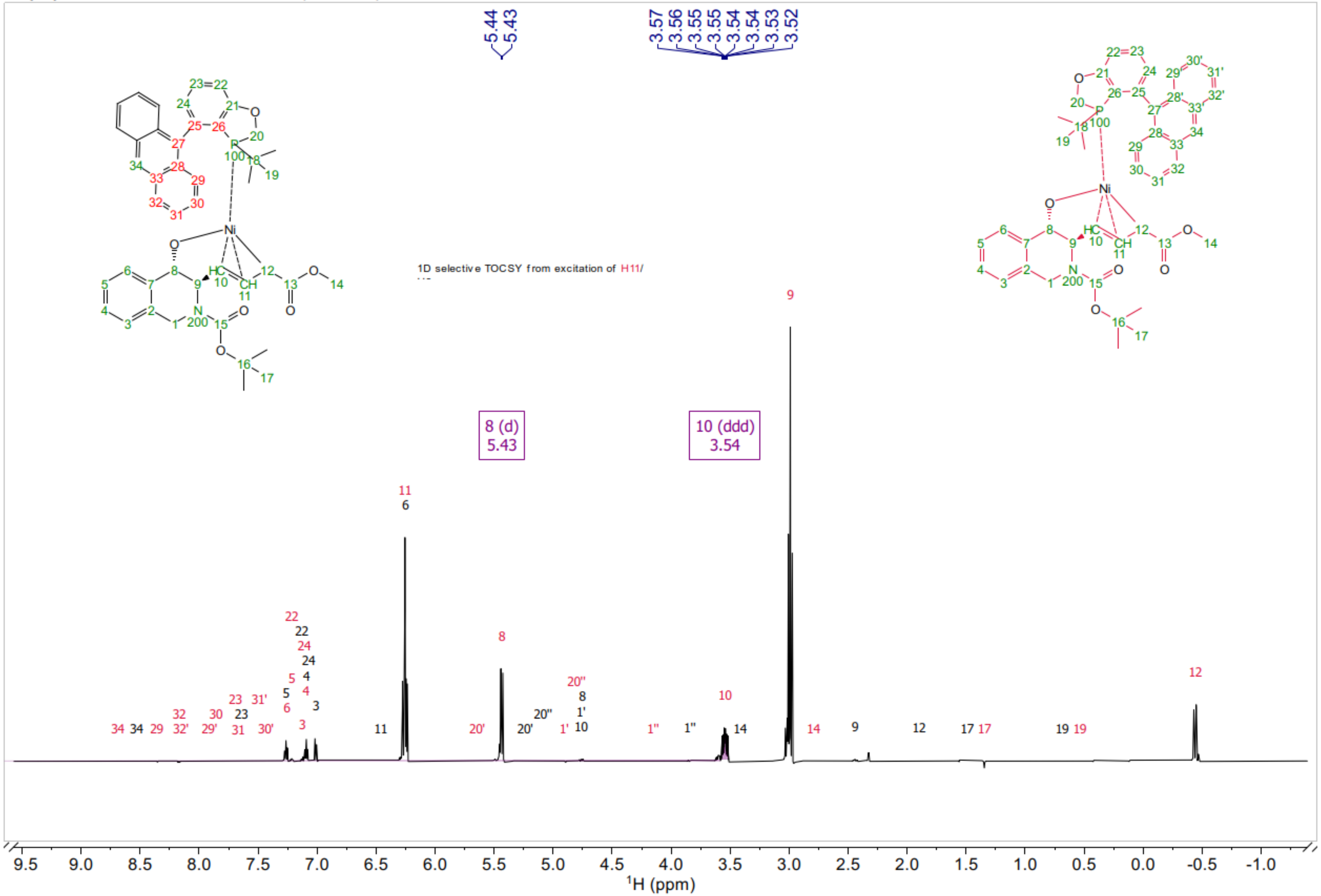

NMR-based DFT Models of Rotamers 27a/b

Initial structures were generated in Avogadro 2.0[14] based on the crystal structures of **25a**/**e** by replacing the PCy_3 unit with (*S*)-AntPhos. The ligand conformation was adjusted to account for the qualitative NOEs observed in the NMR analysis. The resulting two geometries were optimized using the ORCA quantum chemistry package version 6.1 at the B3LYP-D3BJ/def2-TZVP/CPCM(THF) level of theory, employing tight optimization criteria.[15] Harmonic vibrational frequency analyses confirmed that the optimized structures correspond to true minima, with no imaginary frequencies observed.

Cartesian Coordinates

27a

Ni	21.11004338531410	-0.84320853092480	14.80174387148847
P	19.97832923031588	-1.21153203700046	12.91941779039005
O	19.90024634704350	-1.87133549732332	15.79930519767758
O	24.54031948358751	-0.98376213987985	13.26107585340927
O	23.95926879075384	1.09055995020092	12.60405090586610
O	22.85954141705898	-0.48171607932805	20.77163692637586
O	23.76202937660586	-0.87071417890191	18.71505847340921
N	21.55926821533645	-1.05309252593362	18.98345275024492
C	19.98240039813349	-1.60854191312244	17.16247545681451
H	19.48200069509132	-0.65438430190190	17.40532409632200
C	21.46935897734881	-1.40123560781215	17.56239286632832
H	22.00168433346581	-2.33709010102033	17.36701523000418
C	21.93922443900221	-0.34998199777887	16.60196892219646
H	21.51612363760754	0.64097199577911	16.73395733726154
C	22.91138433149402	-0.55976278321720	15.63834409269126
H	23.52043720861926	-1.45624223417843	15.65056448681458
C	22.87543286418071	0.24158911382795	14.47039183217137
H	22.50118692285198	1.25450190190852	14.53795336270795
C	23.86333272997011	0.01618515374874	13.41388580636003
C	24.88198285957402	0.99599322558934	11.50707061611894
H	24.77175351318779	1.92635672557883	10.95729293517496
H	24.63294823526663	0.14719068003049	10.87195676522333
H	25.90125630451961	0.88833494842282	11.87646949335960
C	20.36733068976791	-1.23597853590752	19.83017315925289
H	19.72937344013927	-0.34795816693721	19.75264953351701
H	20.70079649157963	-1.30890357069399	20.86057082416421
C	19.59270394942279	-2.46024459294543	19.42087664801618
C	19.37609112353873	-2.65339968057204	18.05198683437881
C	18.66957339130131	-3.76082145483374	17.60447686261072
H	18.52419348261380	-3.90211300149589	16.54165704216982
C	18.15880051515606	-4.67372050282061	18.52553682911434
H	17.60264293244068	-5.53626961401414	18.17935550624296
C	18.36721936569966	-4.48293504994770	19.88787769702370
H	17.97538055718038	-5.19592108590171	20.60244338546849
C	19.09099035751532	-3.37873593922452	20.33491964057407
H	19.26636436952311	-3.23531562614273	21.39483518159892

C	22.74511608524246	-0.77439878935591	19.58933060546419
C	25.12714841462718	-0.42862835449683	19.04648837491175
C	25.11840844714059	1.04587148174726	19.43393912308316
H	24.63110935668548	1.63590723560300	18.65560169737914
H	26.14762421615291	1.39448889037218	19.53336331841745
H	24.60290520555154	1.20653874686271	20.37762527969091
C	25.71542663040968	-1.31829660243703	20.13584804304311
H	25.19475485946818	-1.18264149340620	21.08026164941349
H	26.76846162375614	-1.06657173277999	20.27371070366481
H	25.65044426207569	-2.36706733830908	19.84071620733160
C	25.87054614784824	-0.62916297231516	17.73110219369648
H	25.81478920649150	-1.67153364682510	17.41479688160519
H	26.91957638577340	-0.35966383316498	17.85821983999320
H	25.44232143019578	-0.00325618974627	16.94788856881049
C	18.71339787599994	-2.54740655468684	13.13119539993330
H	18.44175624296951	-2.58960585676527	14.18532430757146
H	22.47071748076146	-3.04283433285447	10.85753787404285
H	22.62974635448601	-2.33666638332972	12.46901992309504
H	21.44541101868431	-3.61739249444480	12.17439221939239
H	19.05810773091015	-3.52101966766543	12.79470606763991
H	22.27923950497616	-0.72098186841405	10.01684022003868
H	19.47778056202969	-3.19212097043639	10.59159806032179
C	20.87518723621579	-1.67776492382957	11.34008939464568
H	22.06514688682701	0.15254043914062	11.53656622538810
C	19.93522860016476	-2.25519560995570	10.27645997982080
H	20.81831118206469	0.22338888804082	10.30211987503137
H	20.51764274054867	-2.45684181079544	9.37344107050727
H	19.14575076738312	-1.55091770656165	10.01327571373773
O	17.55091821098447	-2.18394965875876	12.34787621127172
C	21.91637741144967	-2.72995475323636	11.74609938681445
C	21.55000551241108	-0.42724938392982	10.77558859367859
C	18.63035712762735	-0.08857796914796	12.44926954613497
C	18.58297075288878	1.30777852614651	12.37489362407769
C	17.48905058154492	-0.84196329631856	12.14230075174894
C	17.41903870724401	1.90681357564030	11.89041847527948
H	17.36739794756799	2.98595979553128	11.83172239658358
C	16.33349130650068	-0.24660085544794	11.65403861447038
H	15.46933431456999	-0.85033518472096	11.41137780028523
C	19.67967419273117	2.16292617074781	12.90929528073132
C	16.32086348688798	1.13574934700432	11.51638096060941
H	15.42871408144922	1.62374505410283	11.14487003720596
C	20.54827740060936	2.85537618029198	12.04971915253947
H	22.48075585255204	4.43681741689249	14.40053441893975
C	19.78437495615865	2.32069086703749	14.30636359454141
C	21.69624197601295	3.81374259514514	13.98672980223890
C	20.80765813121081	3.17492504118859	14.84928008355021
H	19.64769746239410	2.19002418525581	10.19632221076892
C	21.32053684392759	3.43952485271392	9.81882824592293

C	21.59352203274115	3.67544826066077	12.60561350878268
H	21.22185808153809	3.36590279679546	8.74314178794331
H	23.06526071944980	4.72595017817448	9.71192176732403
C	20.44145253684566	2.78240419425143	10.62868500009970
C	22.37169473623015	4.22018988746491	10.37164484148960
C	20.88958348389660	3.34859835439497	16.26213878439131
C	22.49836618732475	4.33605291148254	11.72392001861327
C	18.88435923016061	1.69434461791389	15.22179583779703
C	20.00137645802957	2.73880013731799	17.09858079823626
C	18.98190637141782	1.90626582224587	16.56676912173142
H	18.10521008371779	1.05281842571251	14.83761864454498
H	20.07173471104395	2.88119042574442	18.16934517152873
H	23.29017433216144	4.93547234049320	12.15693418943866
H	21.67446605435486	3.98309405618933	16.65573554638751
H	18.27947434270532	1.43084625740336	17.23878071557558

27b

Ni	20.03110572833202	4.14011299071641	12.68139299598849
P	19.64612360499154	6.06136769261841	11.62872710112303
O	19.50481654501125	4.62696871602544	14.40831267051138
O	19.03427074168933	2.13048305714489	9.72528249352530
O	20.67420888261231	3.43791782925318	8.91141164390432
O	20.70744502232087	-0.80230041224738	16.48730194789634
O	20.01483539450465	-0.23654141824940	14.39189744407516
N	20.54536706522073	1.38240735686364	15.82529273216455
C	19.98552045553155	3.76532995313903	15.38814256479537
H	21.02883977315926	4.01900176903401	15.65720567726776
C	20.03632432934859	2.32158161216600	14.81971137403869
H	19.01357028169930	2.04927956459747	14.55347274157438
C	20.83715904932007	2.47614717415255	13.55816687139411
H	21.86504413851746	2.81483954836728	13.66925636570165
C	20.32031753847077	2.18320392181916	12.30660101696124
H	19.39588285952045	1.63026034108073	12.22527908030531
C	20.76223564401091	2.92272202645702	11.18046049778640
H	21.78836749968104	3.26797637946909	11.11865705745988
C	20.05546607689530	2.76592637199565	9.90413358140183
C	20.02846640828591	3.42095455985903	7.62610014724822
H	20.64924276618545	4.03603152812657	6.98133143571821
H	19.02652312178863	3.84107590252040	7.70061513443519
H	19.97110241438438	2.40267338577940	7.24373629733835
C	20.72122494404536	1.88293891037106	17.20122173657895
H	21.66471966007947	2.43511101580897	17.26026006680531
H	20.79818406637676	1.02333434821007	17.85878811051099
C	19.57116814888380	2.77538944897164	17.58943308992157
C	19.19296152872081	3.75413615477445	16.66299512823680
C	18.12508600456483	4.59789472984488	16.93104746026365
H	17.83172659841094	5.32958597507821	16.19145963200787
C	17.43692683507798	4.47909430101866	18.13650269046788

H	16.60210911743662	5.13625925864817	18.34761947304606
C	17.81287804961872	3.51216944904971	19.06418165314583
H	17.27301612823573	3.41664038115036	19.99806864803387
C	18.87724843740205	2.65507390075547	18.78727476941075
H	19.16088166823849	1.89022856527876	19.50090248609312
C	20.44039586225252	0.03539672919931	15.63790917135363
C	19.81057401093610	-1.61366206160299	13.91369752256757
C	21.12981819116415	-2.37736534151326	13.93003557782861
H	21.89099476492538	-1.82366369004308	13.37748579317956
H	20.98865284542477	-3.34367416378494	13.44299410016028
H	21.47856869597157	-2.54404803453738	14.94600742699349
C	18.72569434253534	-2.29630628488862	14.73933960083656
H	19.05212643898579	-2.46377444456511	15.76268082538519
H	18.48422217868364	-3.25867991754388	14.28505850548492
H	17.82117643396642	-1.68524108375757	14.74869116726756
C	19.33397338114401	-1.40110128718687	12.48178788107911
H	18.41640600009832	-0.81234025593440	12.46727720042305
H	19.13484009738048	-2.36646986133608	12.01542592728026
H	20.09234253469445	-0.88109533083675	11.89579013019057
C	20.76492268488461	6.34880888215494	10.16245628136025
H	21.46742924734712	7.15775941490524	10.34440413059751
H	19.74835686416409	9.77079712924919	12.37528483636727
H	20.34528157300069	8.94886651257979	10.93596116737799
H	18.62300854408377	8.89153556530361	11.33664362056414
H	21.31106184877736	5.44348060734033	9.91733952436875
H	21.45447603259023	8.48855089346827	13.78296989732723
H	17.85193718924789	7.82311421571585	13.47479451684984
C	19.84873410629596	7.63931692555232	12.62837699163560
H	21.43302205681898	6.72015552771881	13.80879337998794
C	18.87096481584315	7.65623893073204	13.80863045870795
H	22.03723061065613	7.58456030332612	12.38607052247645
H	19.14363047577960	8.48453333642802	14.46859191207933
H	18.91639509009980	6.72568956432422	14.37090128185662
O	19.95840581282901	6.70309410236454	9.02074218284267
C	19.62970506433069	8.87787820540528	11.75588329210996
C	21.28462771897863	7.59619863091373	13.17584714512517
C	18.20449830294556	6.19561627533265	10.52724538915525
C	16.83937607465276	5.98868810867761	10.77202732390575
C	18.63057487367422	6.51264112147626	9.23009189775075
C	15.94564711909422	6.10271456220282	9.70673268876400
H	14.89121006501752	5.94396623287271	9.88969445208761
C	17.73781706375389	6.63529827201124	8.17334757837774
H	18.10007999486761	6.88078706206139	7.18414868088862
C	16.30539417781682	5.68668069660499	12.13030929716385
C	16.39036333350467	6.42174305805961	8.42673061308915
H	15.67673625939260	6.50199042257524	7.61664813871273
C	15.70993319387195	6.71629700538040	12.88056997912849
H	14.96343694067664	4.94185187078147	15.71904094799414

C	16.35313267863705	4.37677057468656	12.63695017304247
C	15.31880637027951	5.14461122151652	14.71550455800533
C	15.85196496991429	4.10506993103579	13.95865239643938
H	15.95202792376732	8.27968351750295	11.39959450125018
C	15.04062028727757	9.03922765610403	13.15814733475489
C	15.22661338240515	6.43813536458198	14.20778520090500
H	14.96280062985334	10.04572440585275	12.76671135862580
H	14.15301845592640	9.56209091256820	15.06841684665708
C	15.58843681821896	8.05226954643044	12.39137564712053
C	14.57775898195653	8.76397996129869	14.47292995883019
C	15.91296317743547	2.77339123575501	14.46267918867609
C	14.66983569434578	7.50073260414631	14.97850034866024
C	16.85622840718533	3.28364300513343	11.87359243650854
C	16.39459184545132	1.75483004818335	13.69412655773955
C	16.85552457760646	2.01579764647073	12.37721998862592
H	17.23309878819540	3.46174070286868	10.87773321296763
H	16.43353643176302	0.74514201127276	14.08312485367793
H	14.31960789202926	7.27800535874100	15.97940408870562
H	15.56235028092737	2.58980412570946	15.47093232053268
H	17.22669118588439	1.20179166270363	11.76937289614873

NMR Spectra

^{1}H NMR (600 MHz, $CDCl_3$; top) and ^{13}C NMR (151 MHz, $CDCl_3$; bottom) of compound **S1**

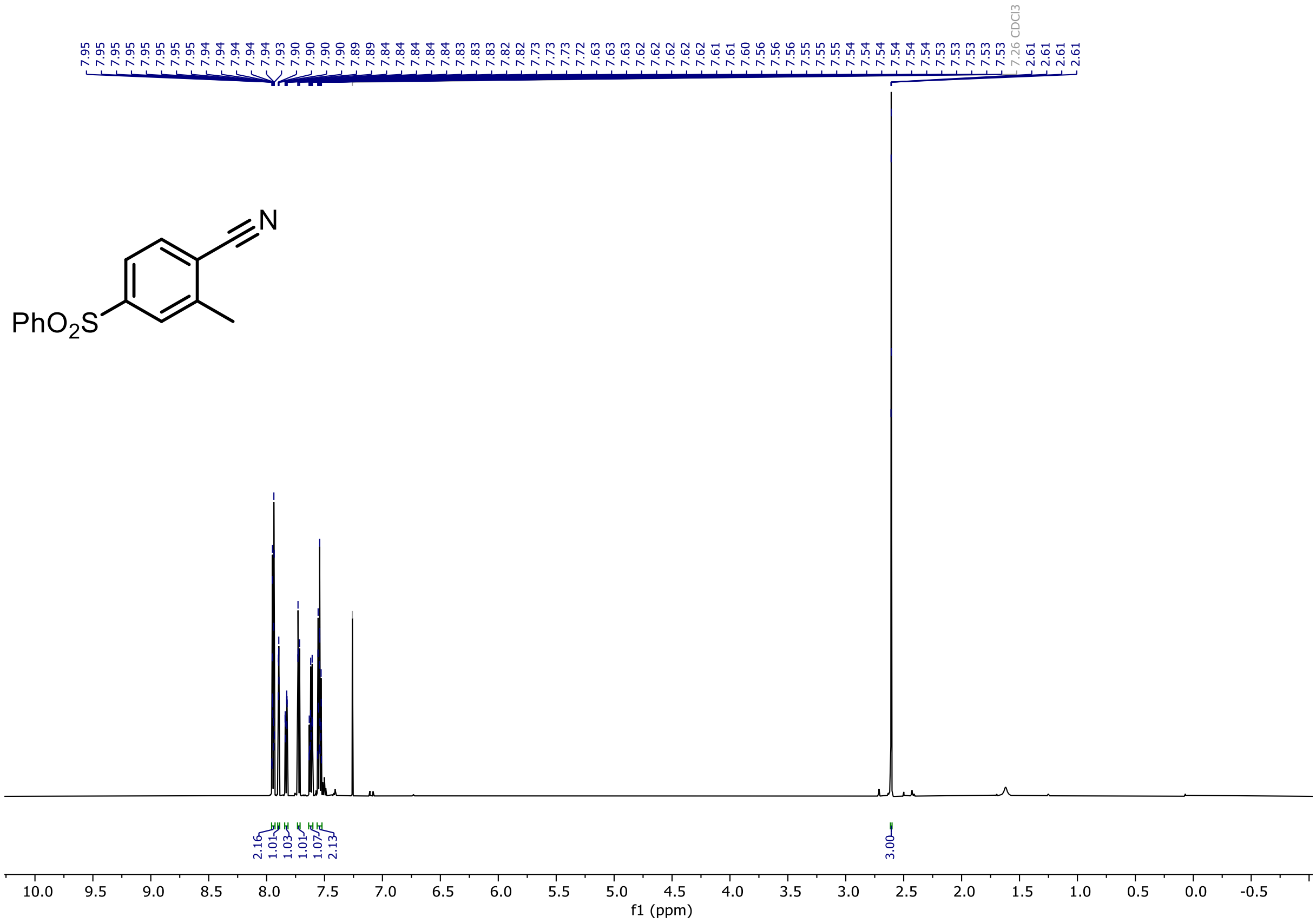

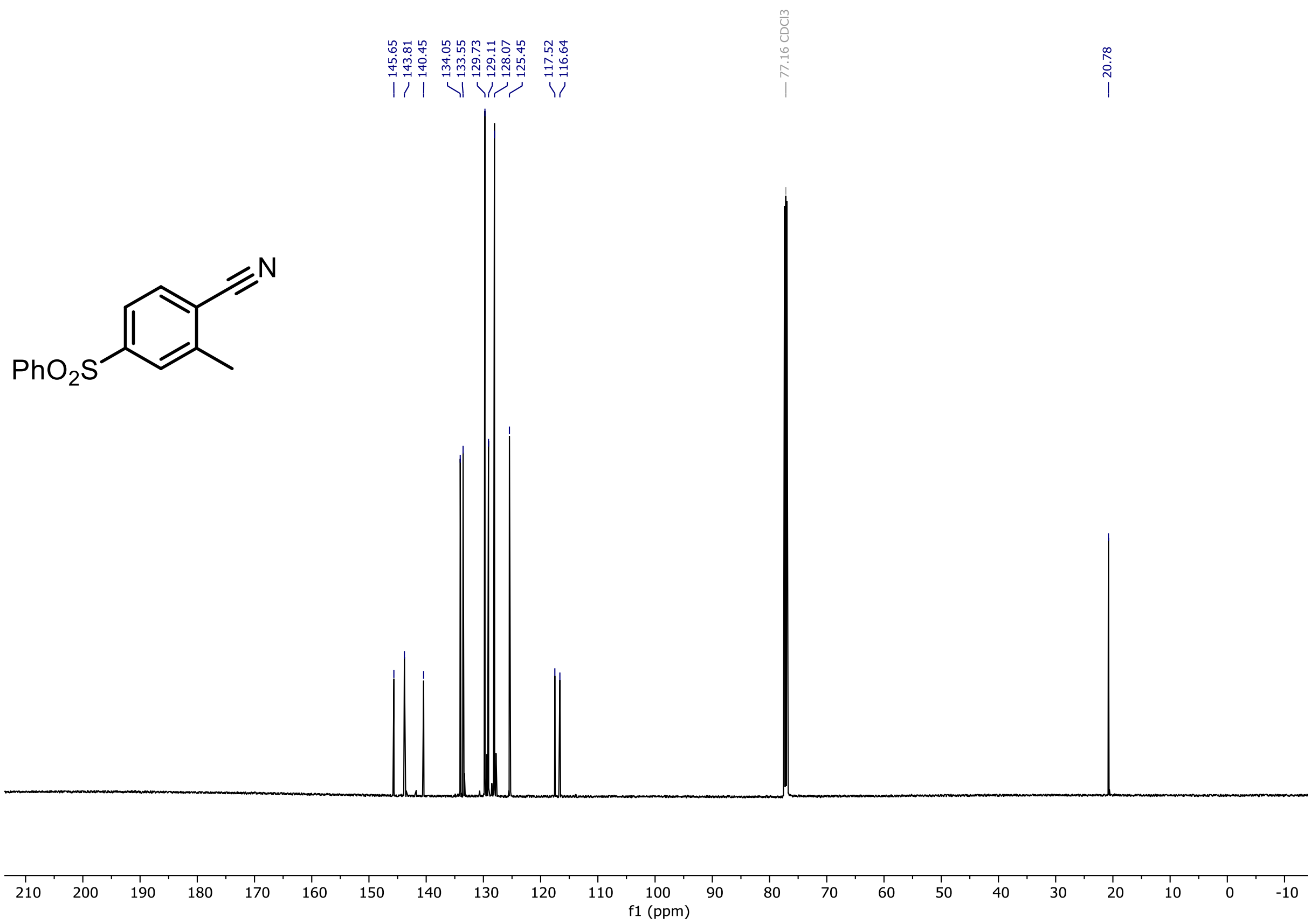

^{1}H NMR (600 MHz, $CDCl_3$; top) and ^{13}C NMR (151 MHz, $CDCl_3$; bottom) of compound **S2**

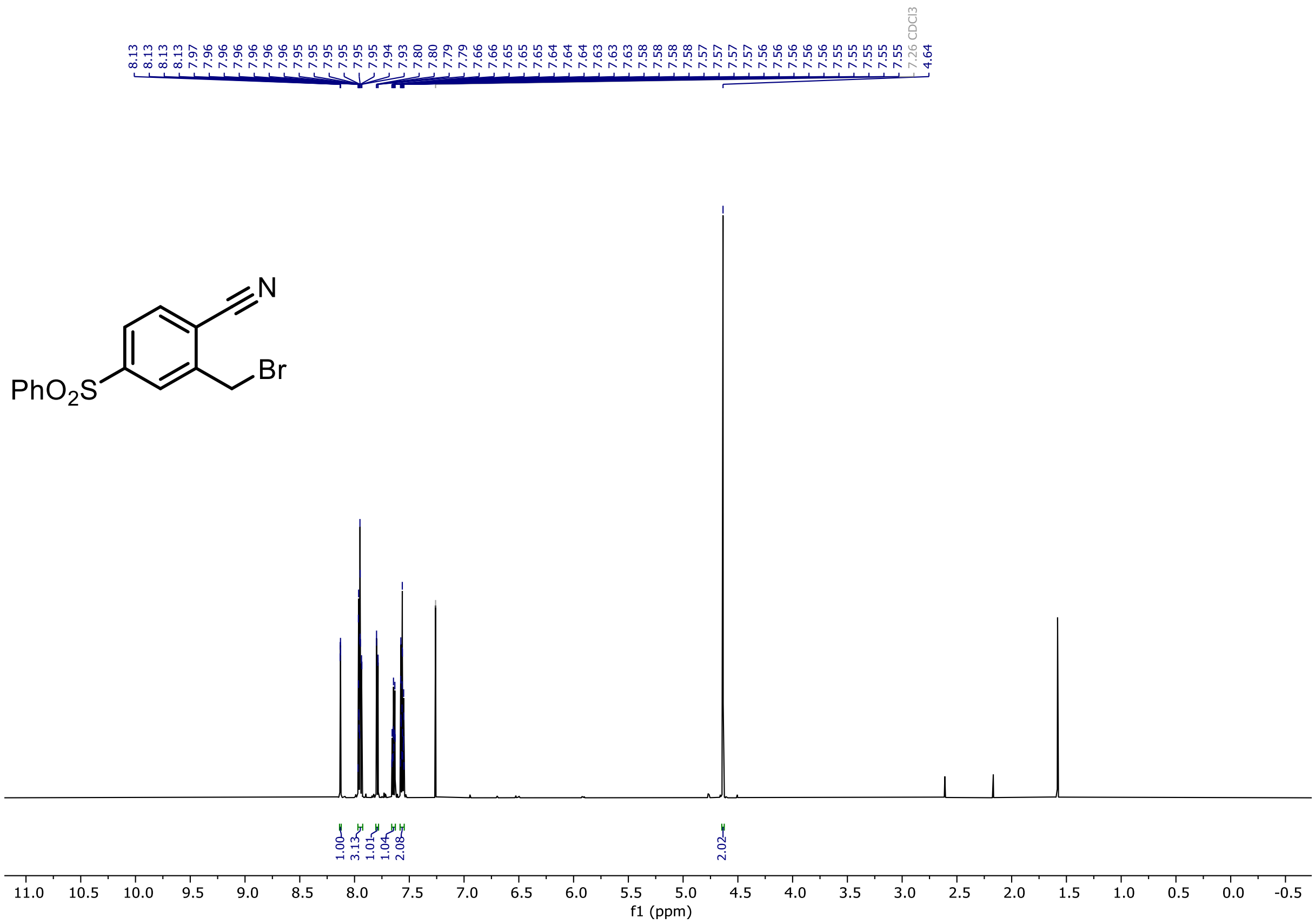

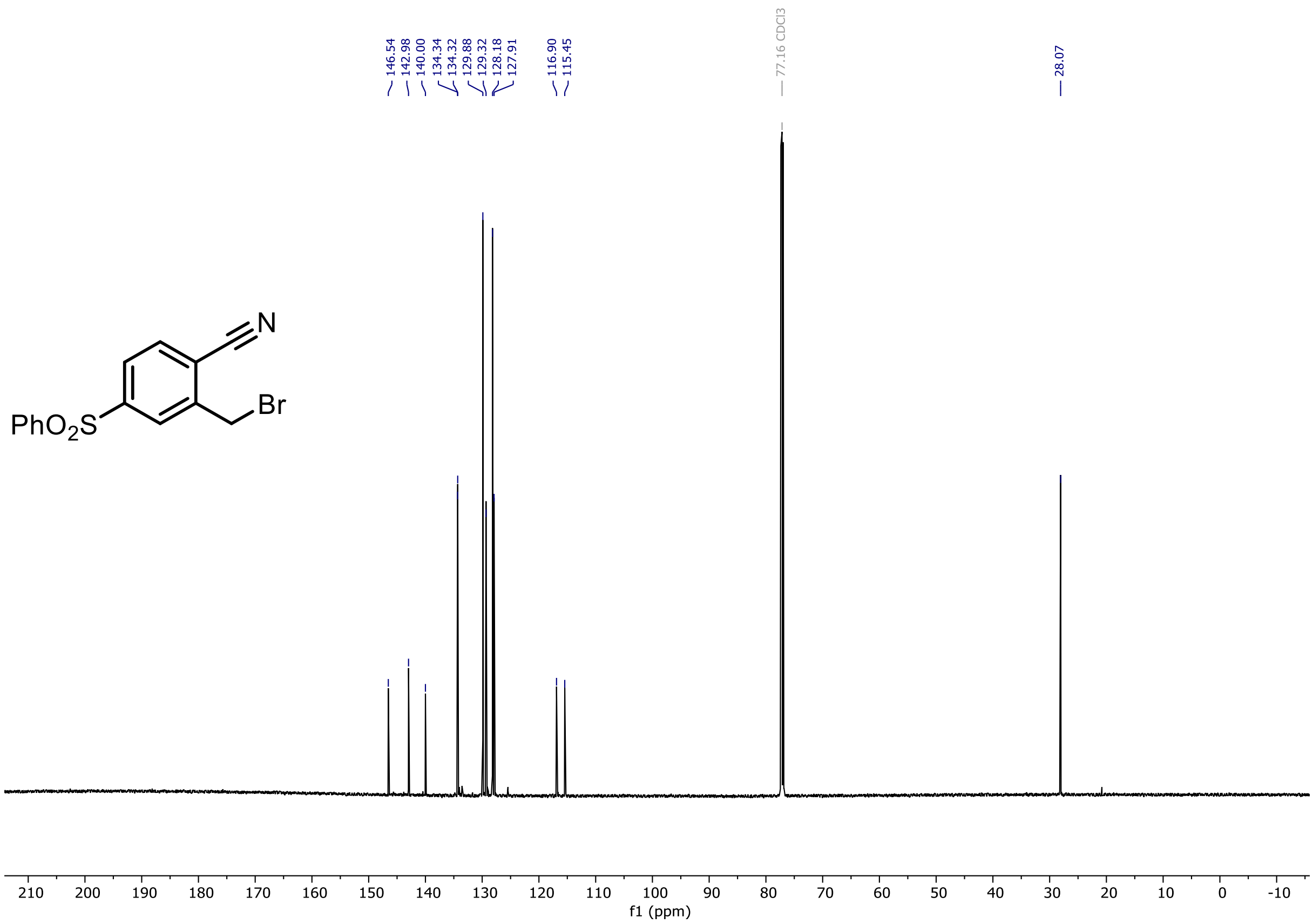

^{1}H NMR (600 MHz, $CDCl_3$; top) and ^{13}C NMR (151 MHz, $CDCl_3$; bottom) of compound **S3**

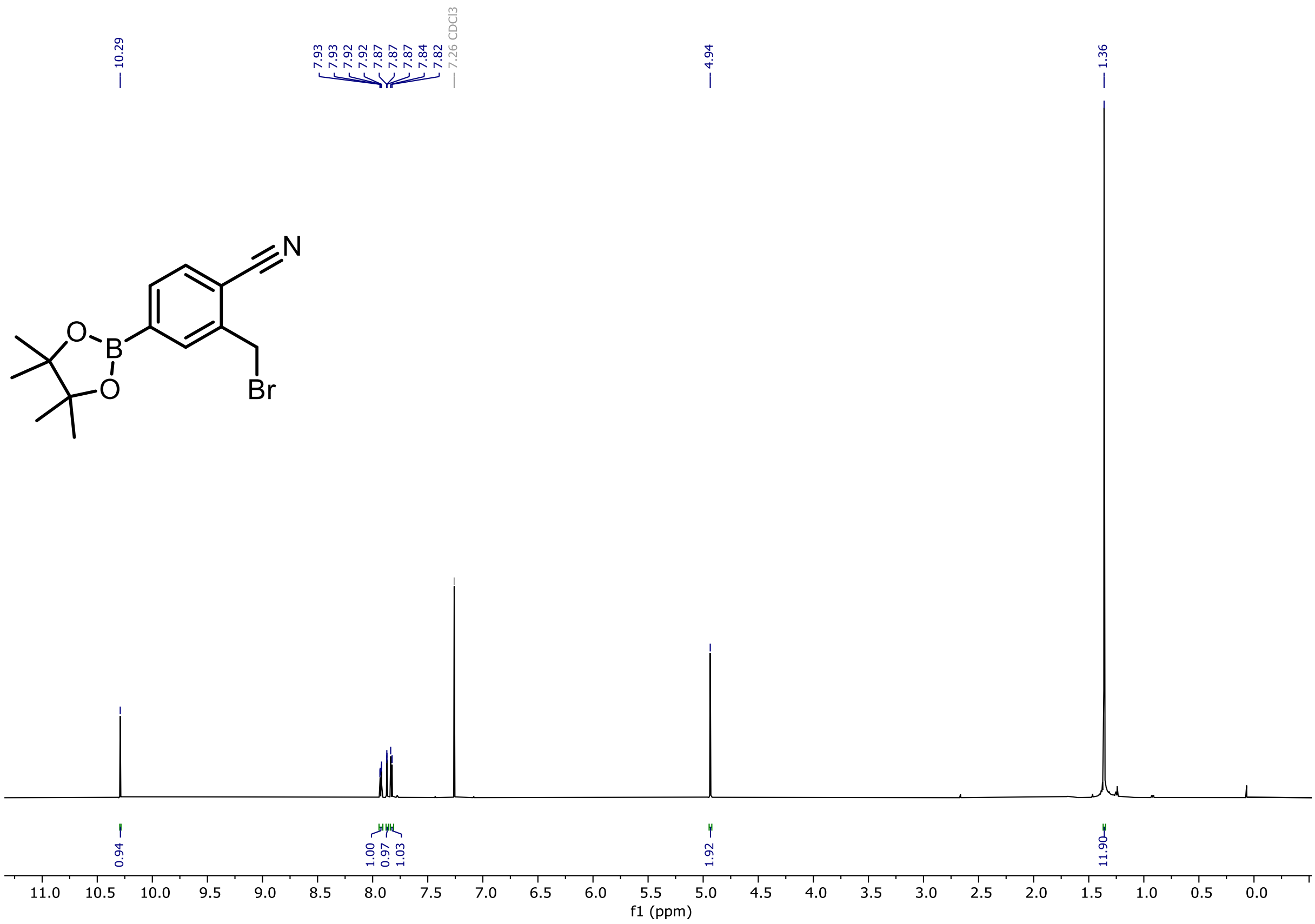

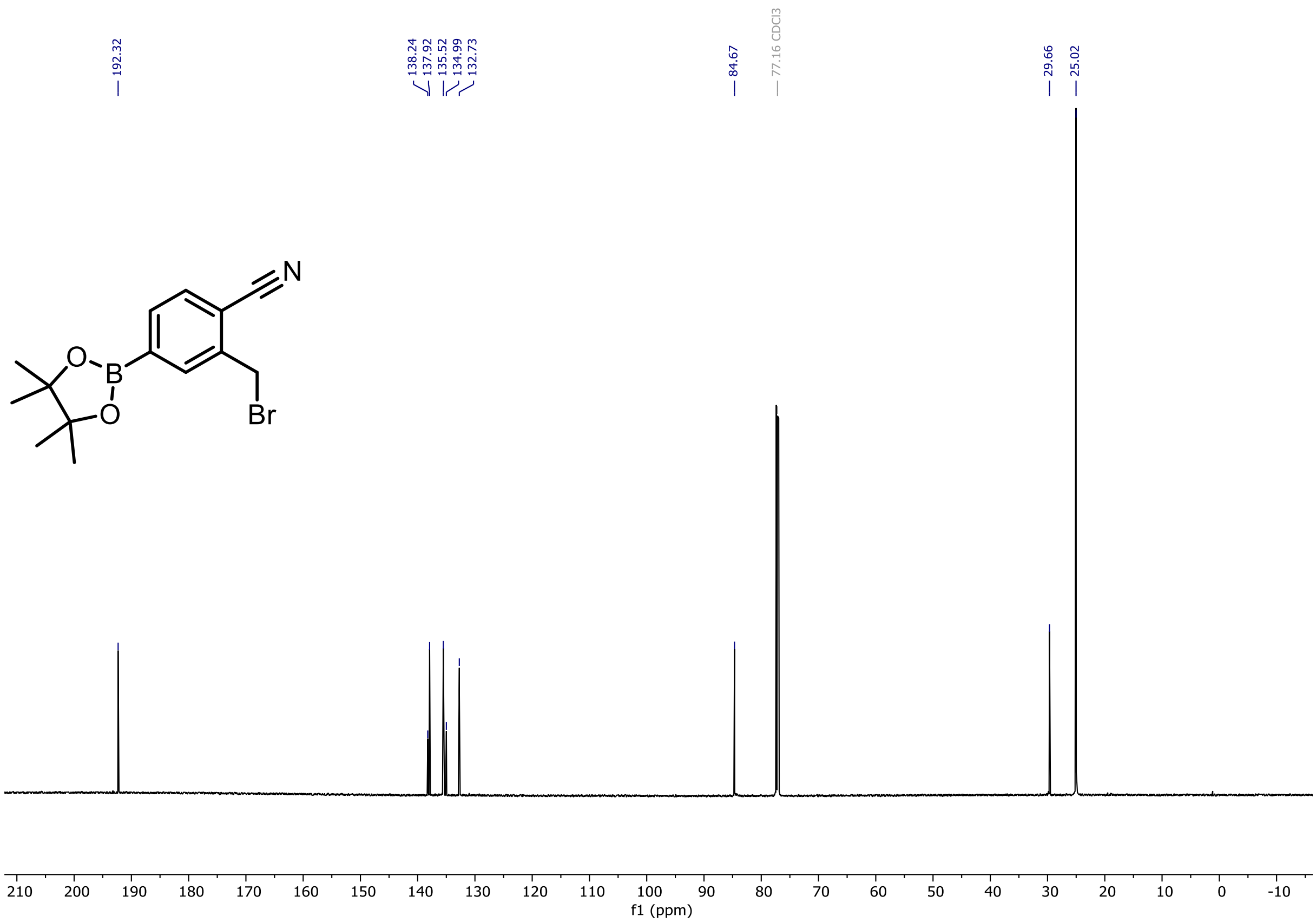

^{1}H NMR (600 MHz, $CDCl_3$; top) and ^{19}F NMR (585 MHz, $CDCl_3$; bottom) of compound **S4**

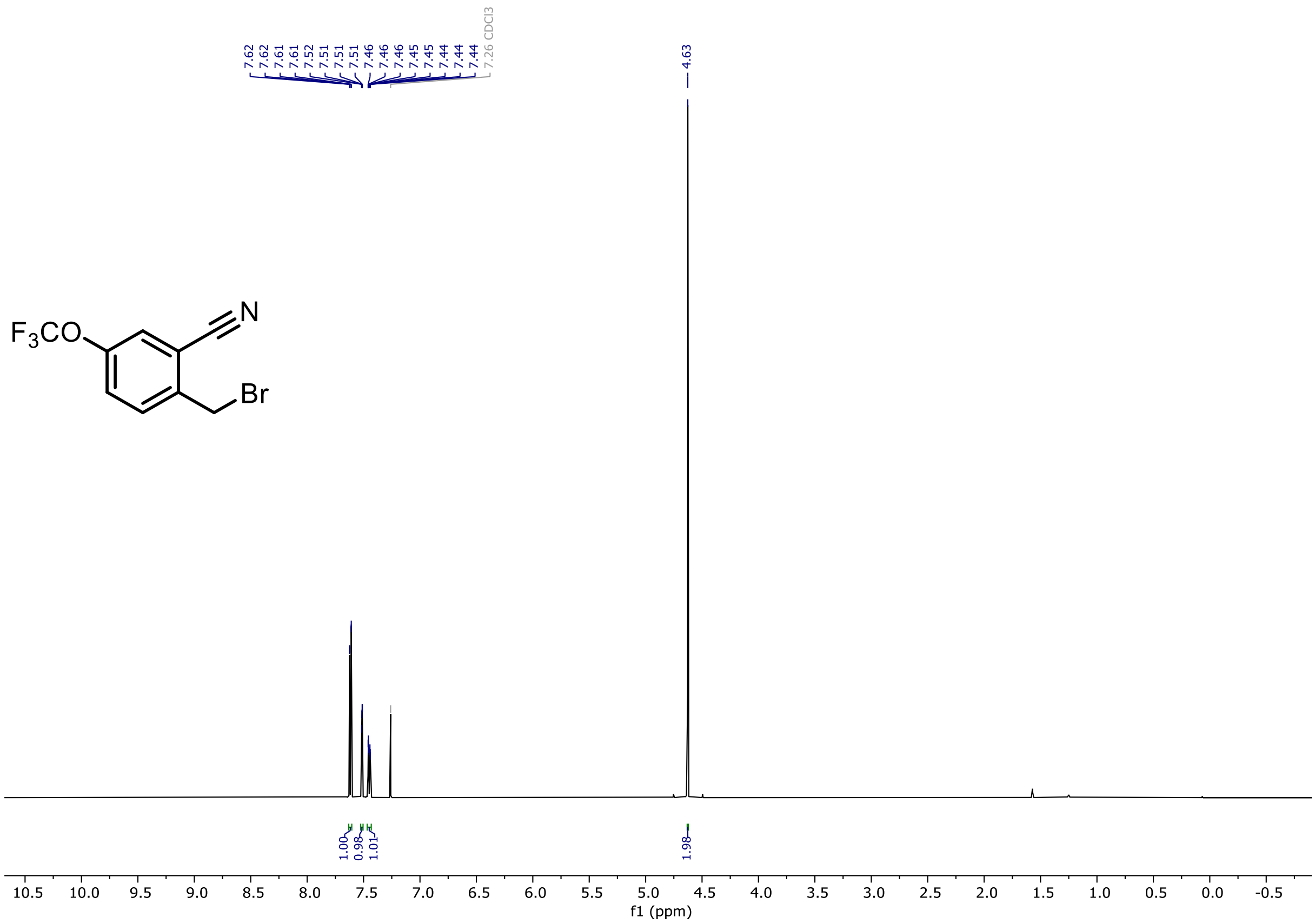

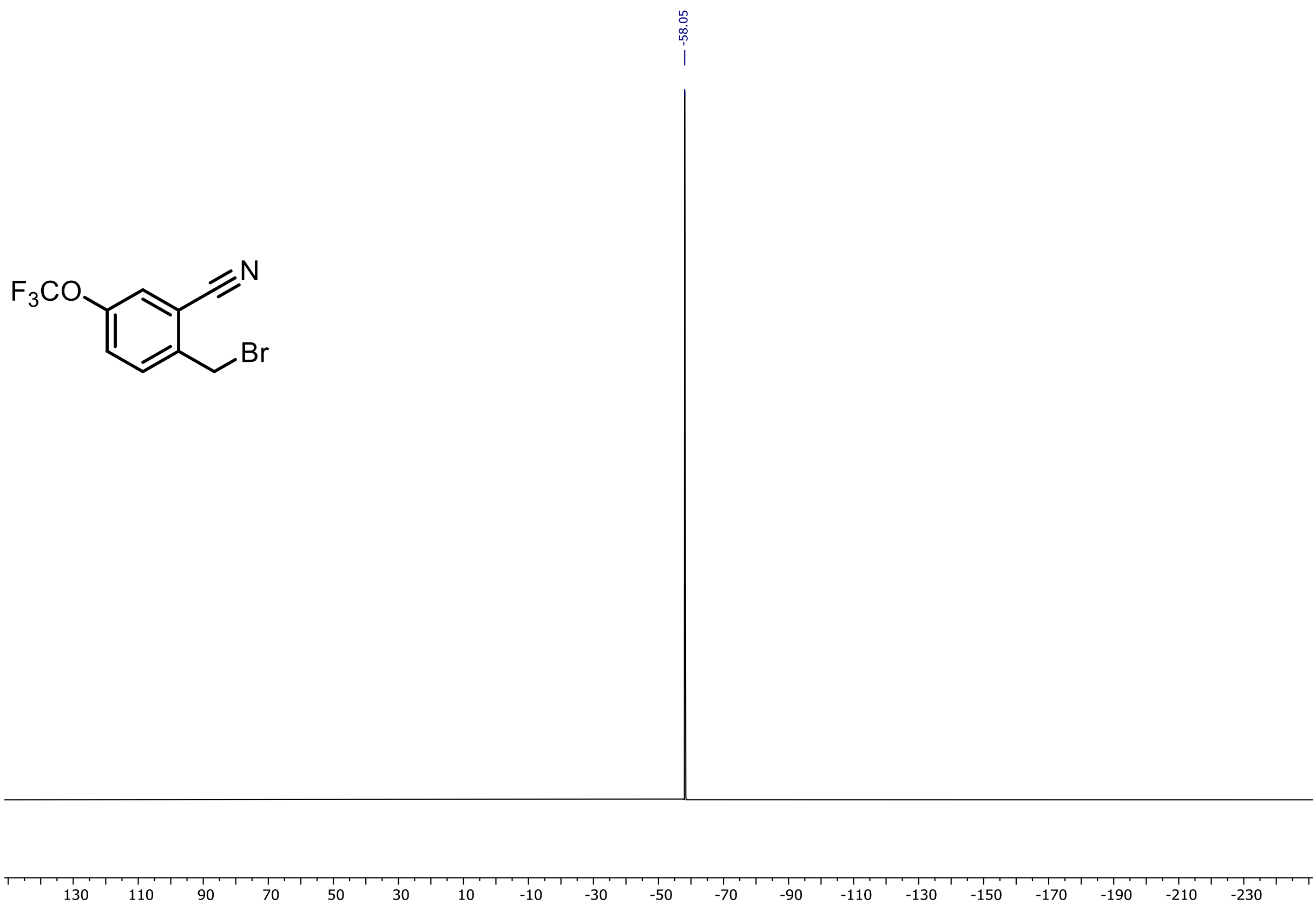

^{13}C NMR (151 MHz, $CDCl_3$) of compound **S4**

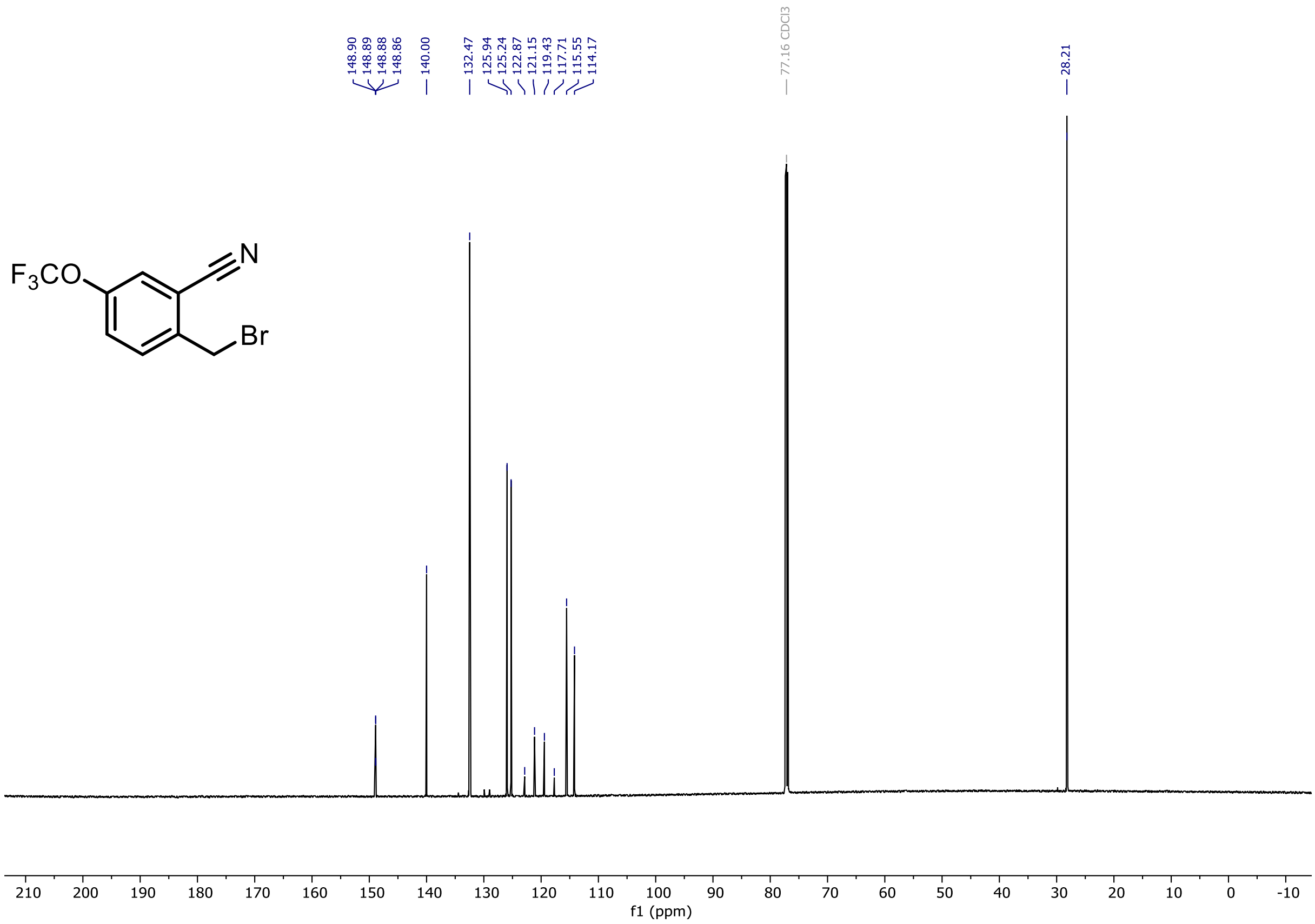

^1H NMR (400 MHz, $CDCl_3$; top) and ^{13}C NMR (101 MHz, $CDCl_3$; bottom) of compound **S5**

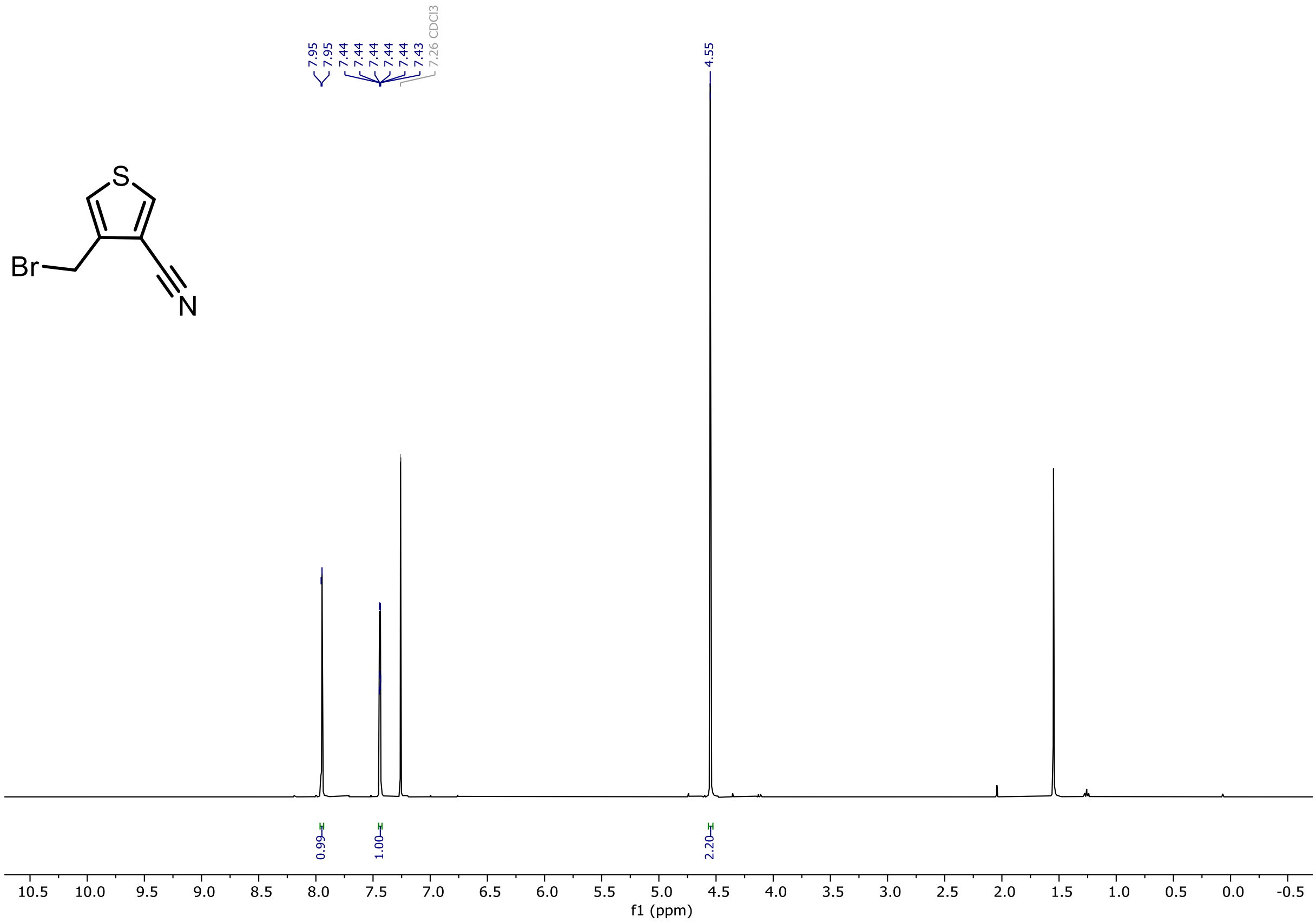

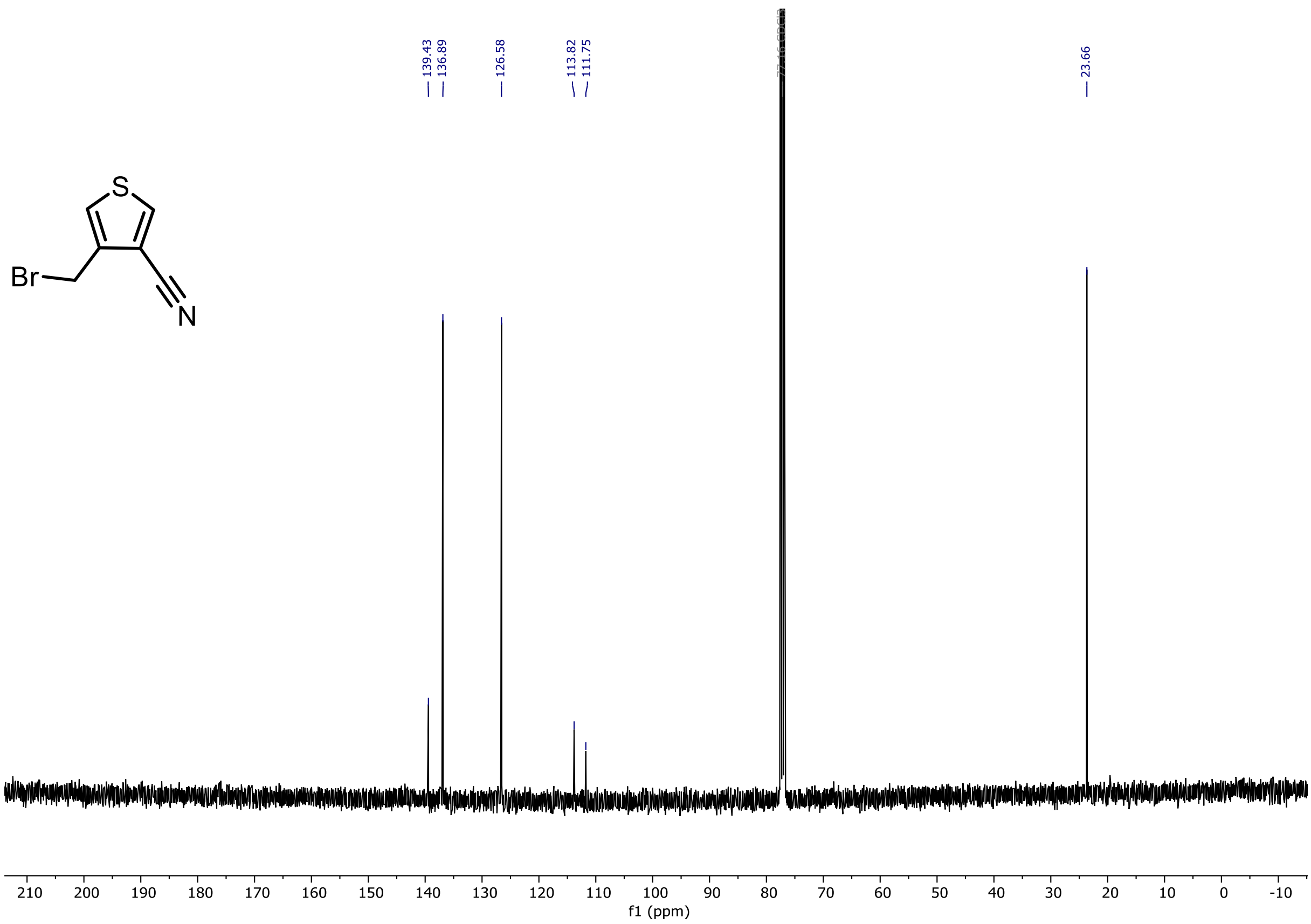

^{1}H NMR (600 MHz, $CDCl_3$; top) and ^{19}F NMR (585 MHz, $CDCl_3$; bottom) of compound **S7**

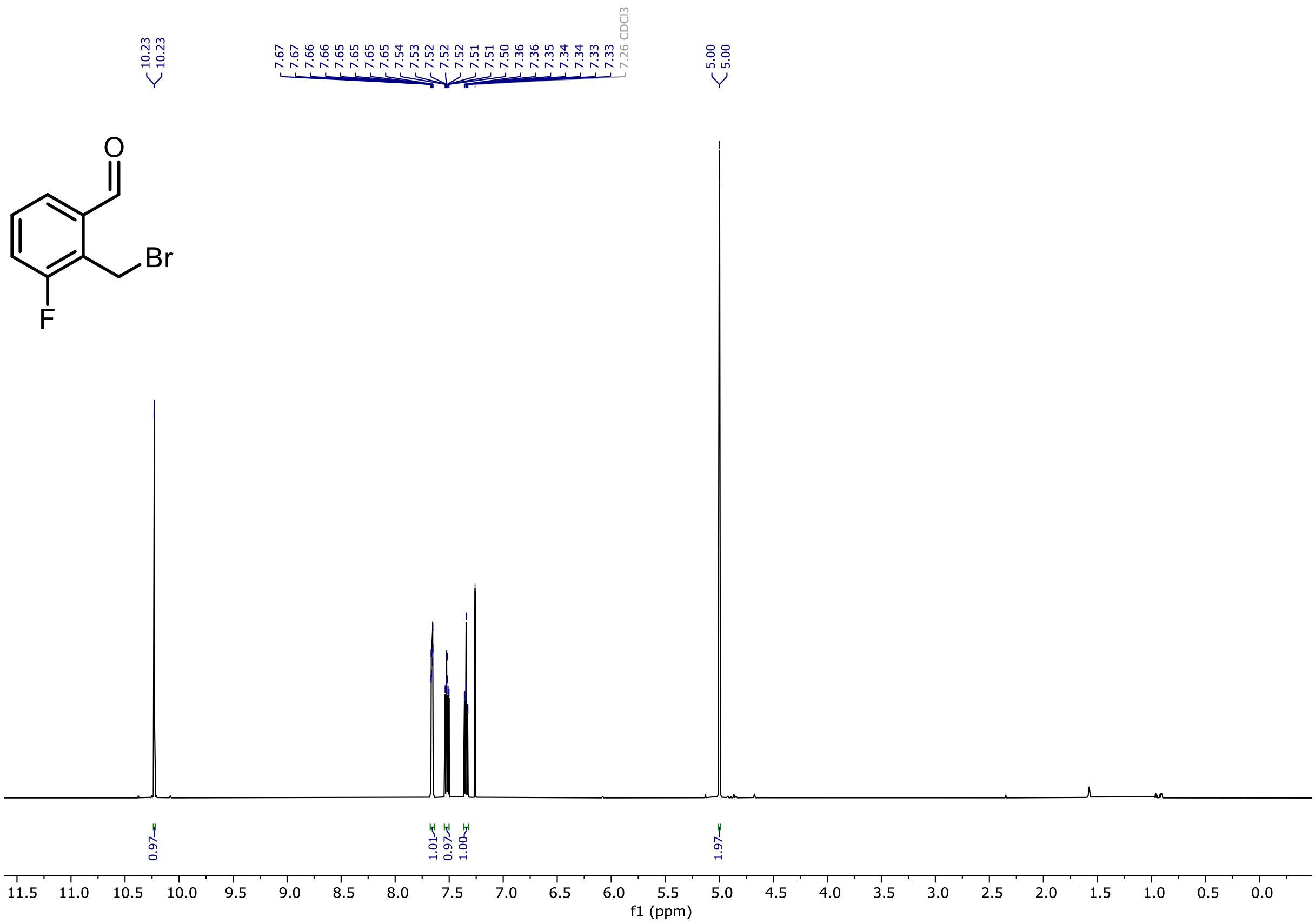

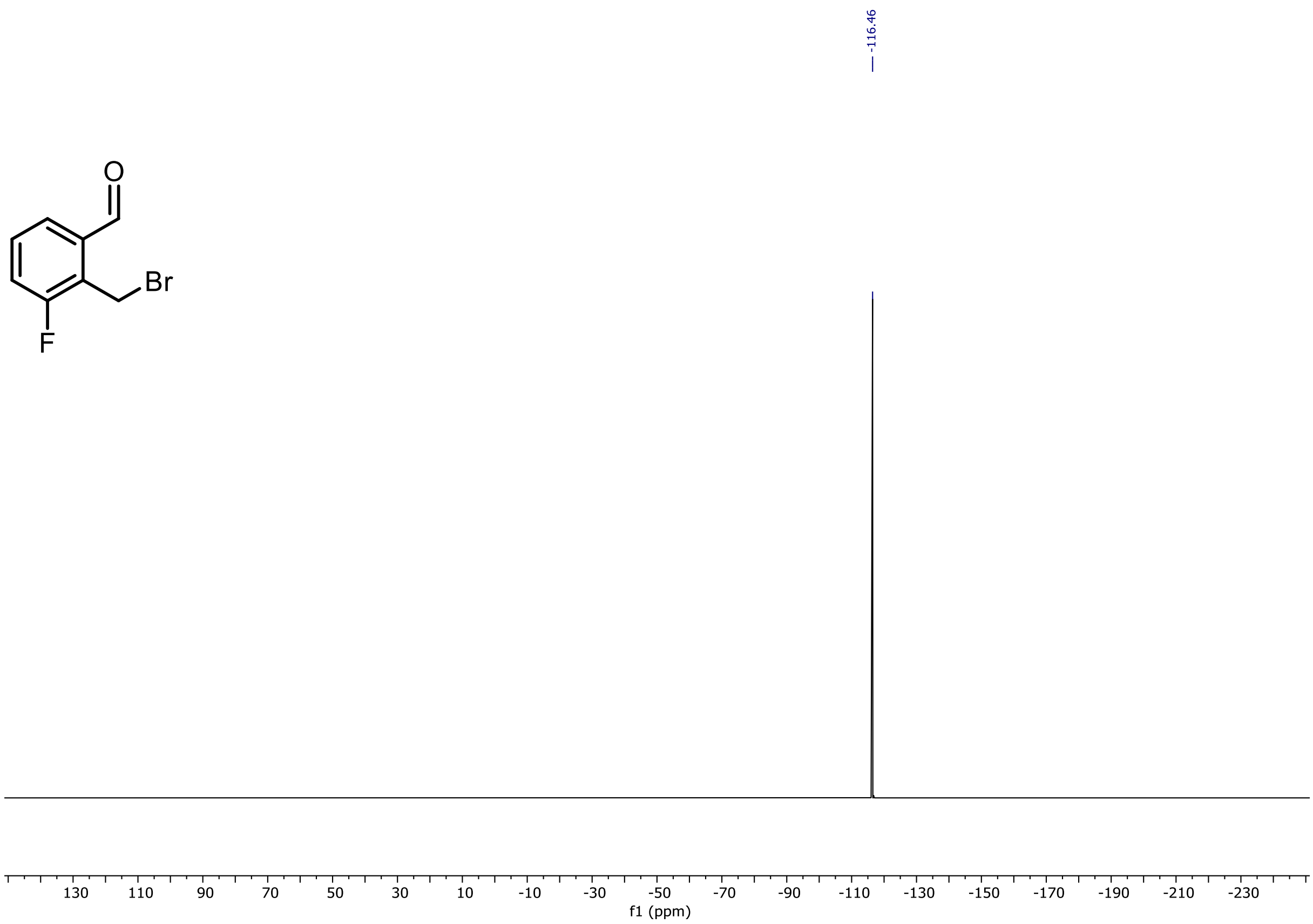

^{13}C NMR (151 MHz, $CDCl_3$) of compound **S7**

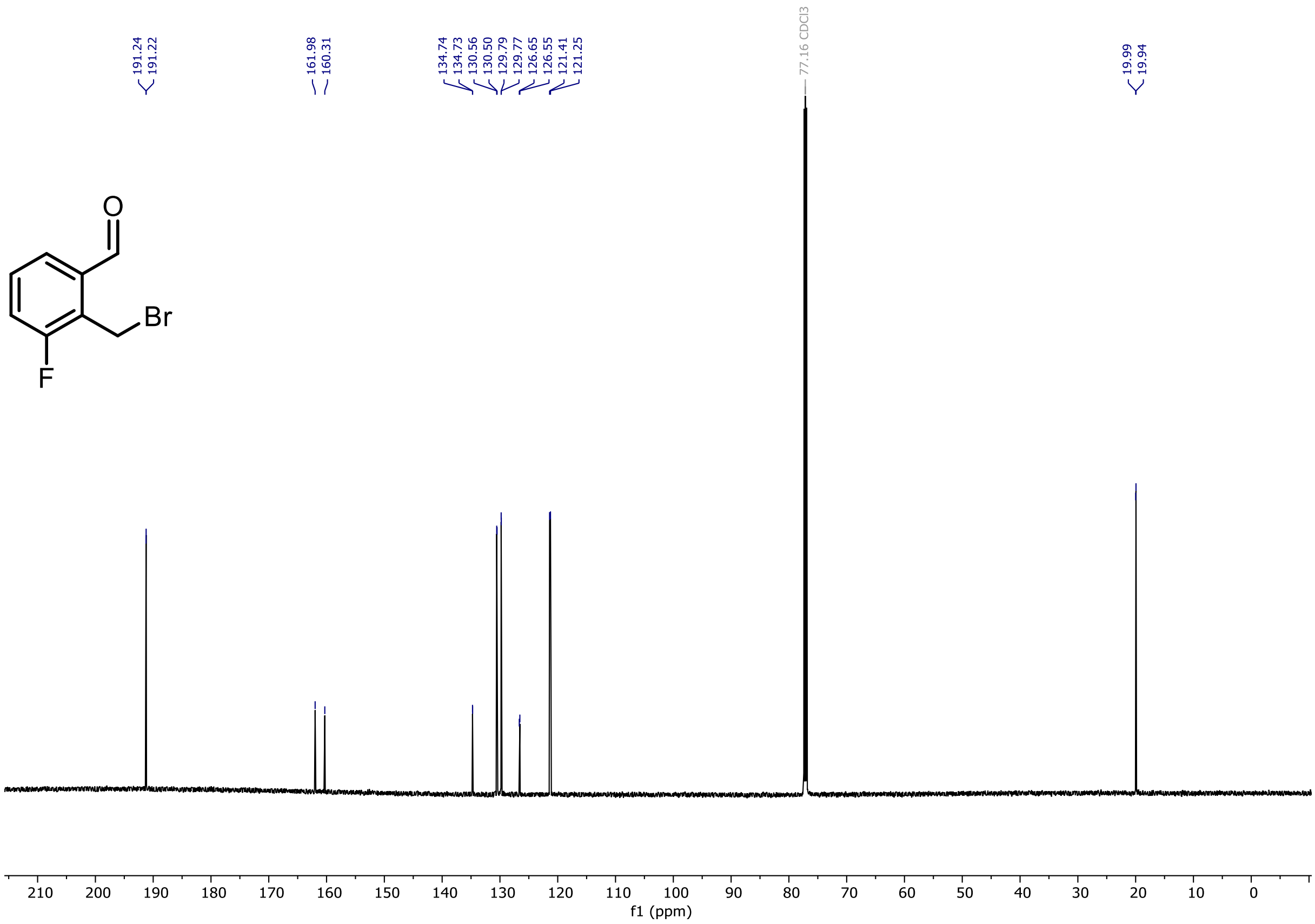

^{1}H NMR (400 MHz, $CDCl_3$; top) and ^{19}F NMR (282 MHz, $CDCl_3$; bottom) of compound **S9**

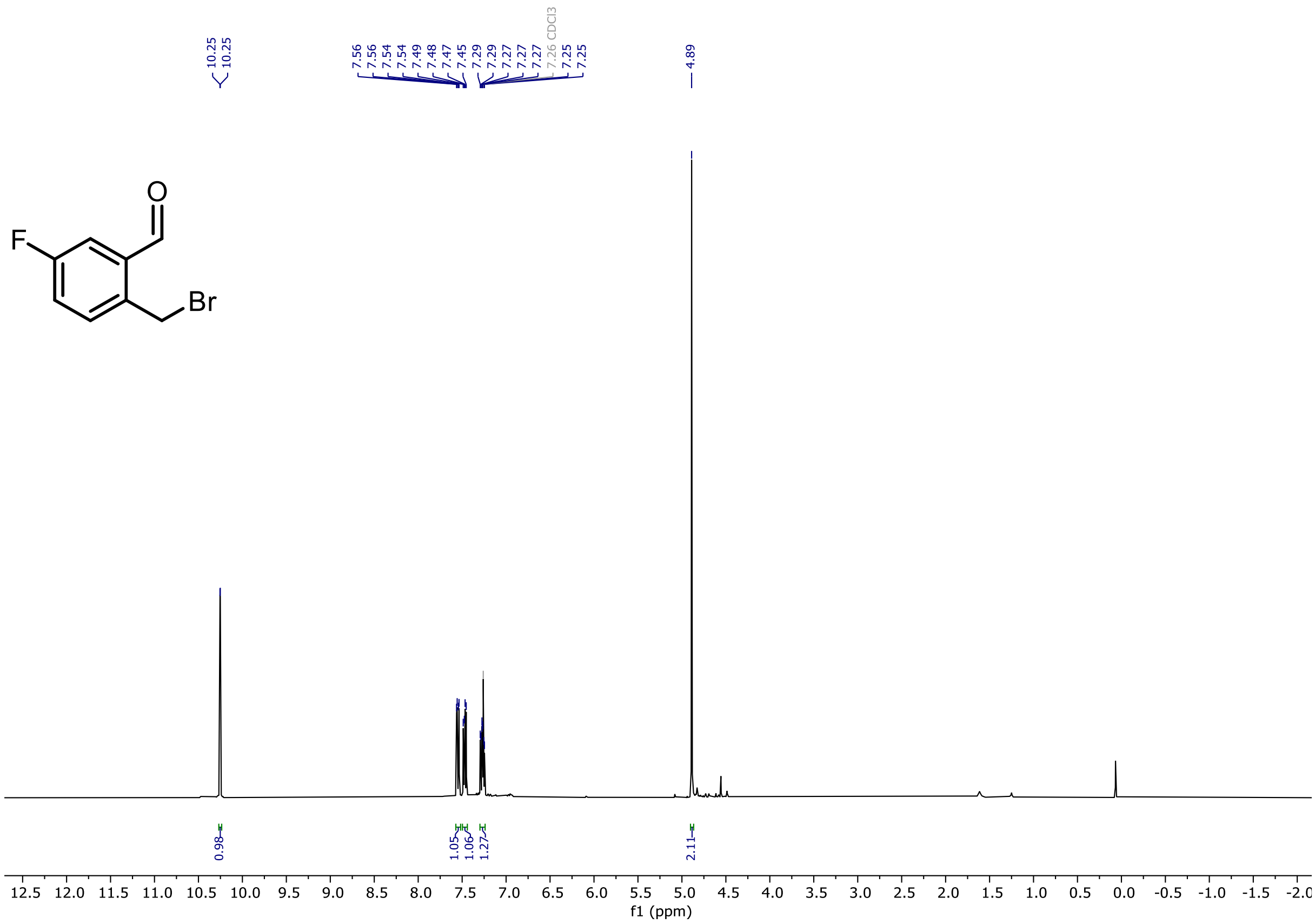

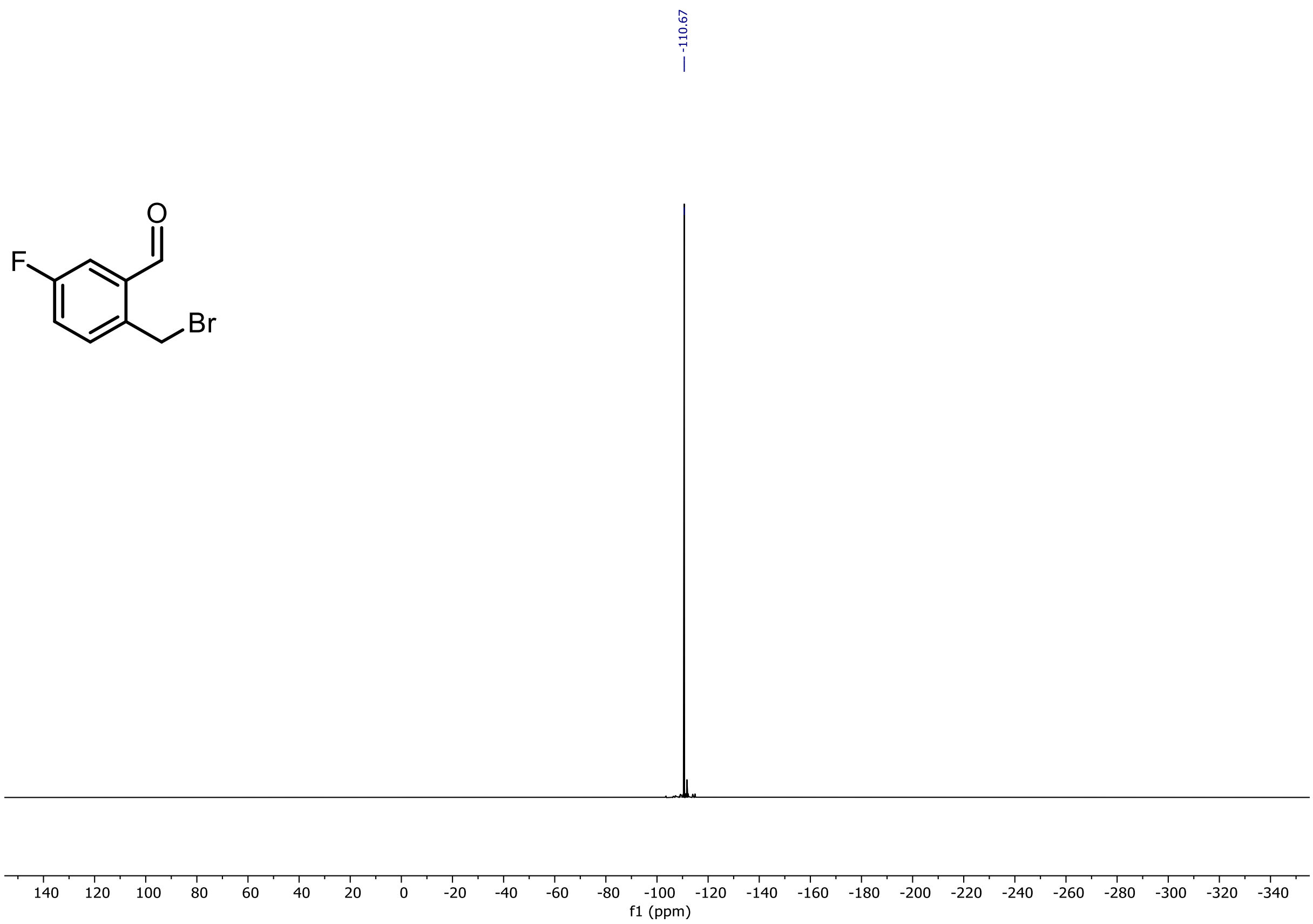

^{13}C NMR (101 MHz, $CDCl_3$) of compound **S9**

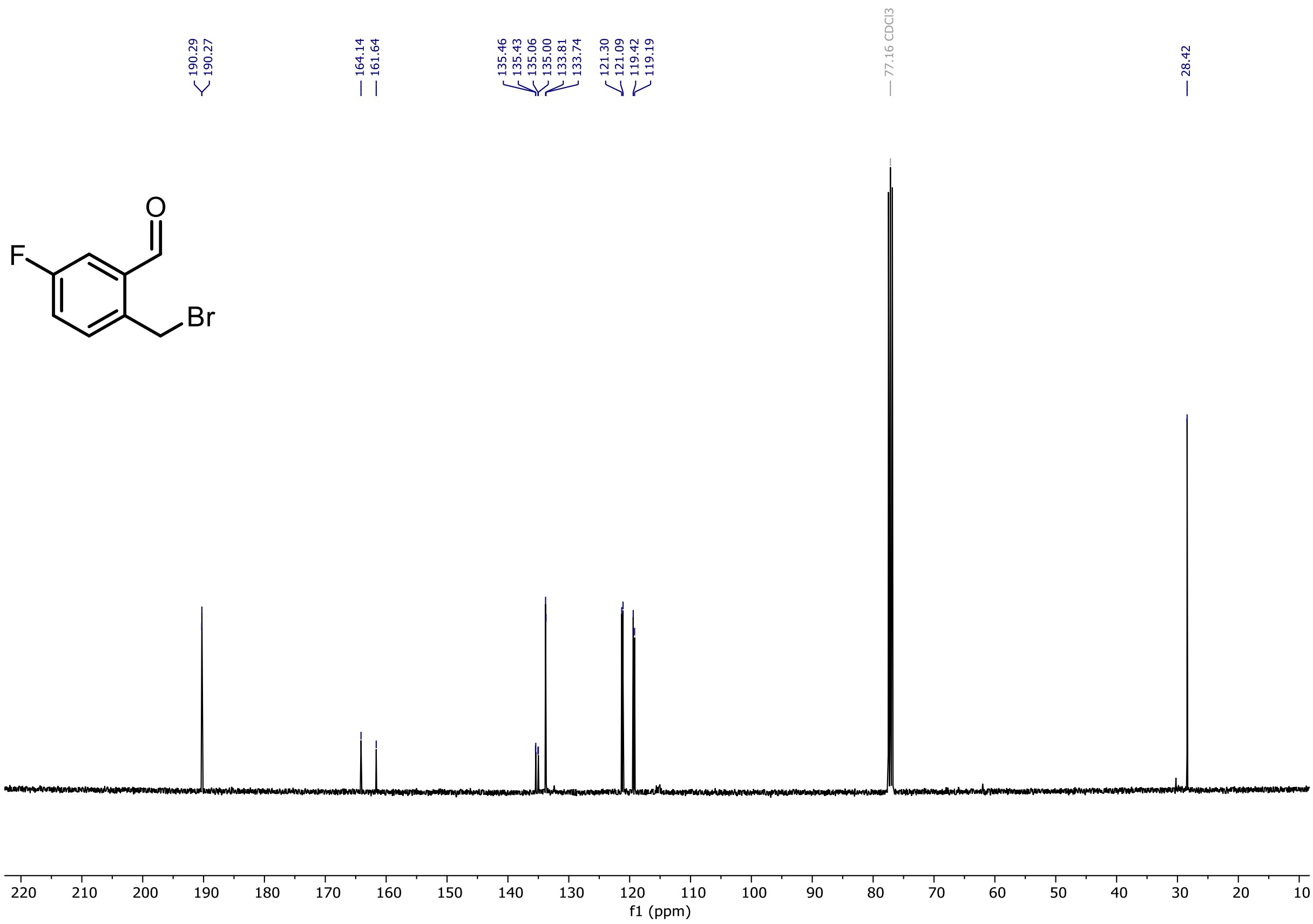

^1H NMR (400 MHz, $CDCl_3$; top) and ^{19}F NMR (282 MHz, $CDCl_3$; bottom) of compound **S10**

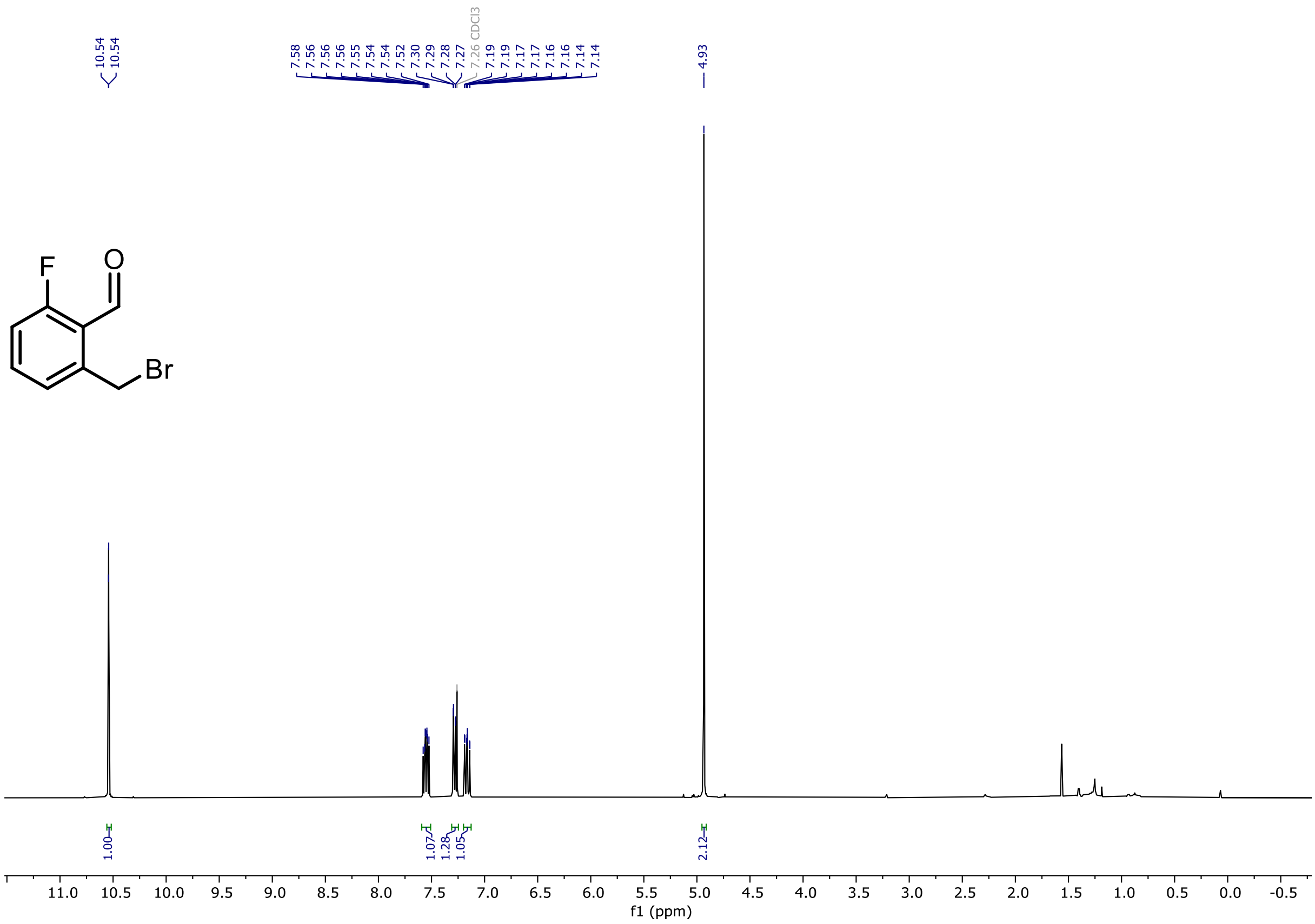

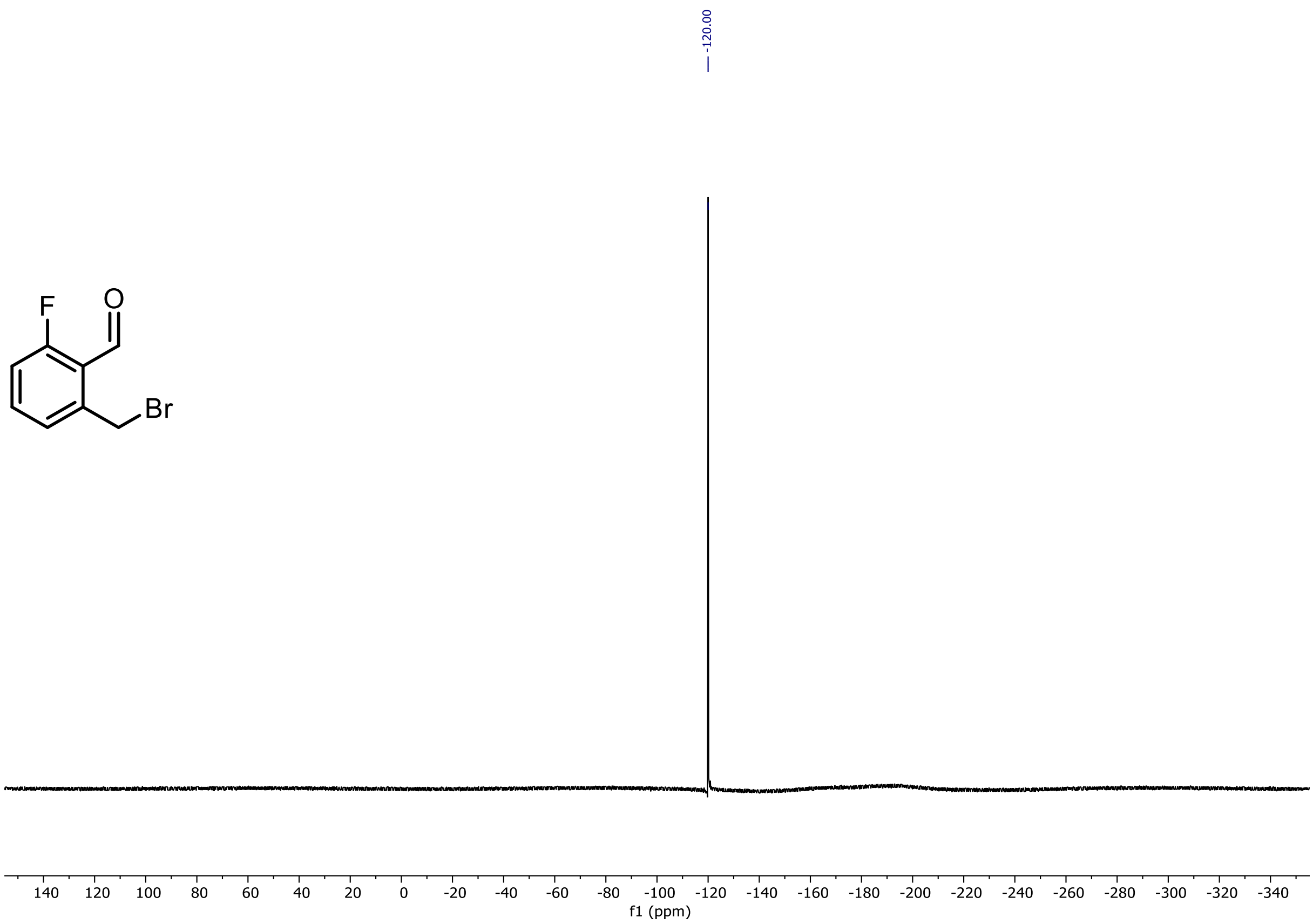

^{13}C NMR (101 MHz, $CDCl_3$) of compound **S10**

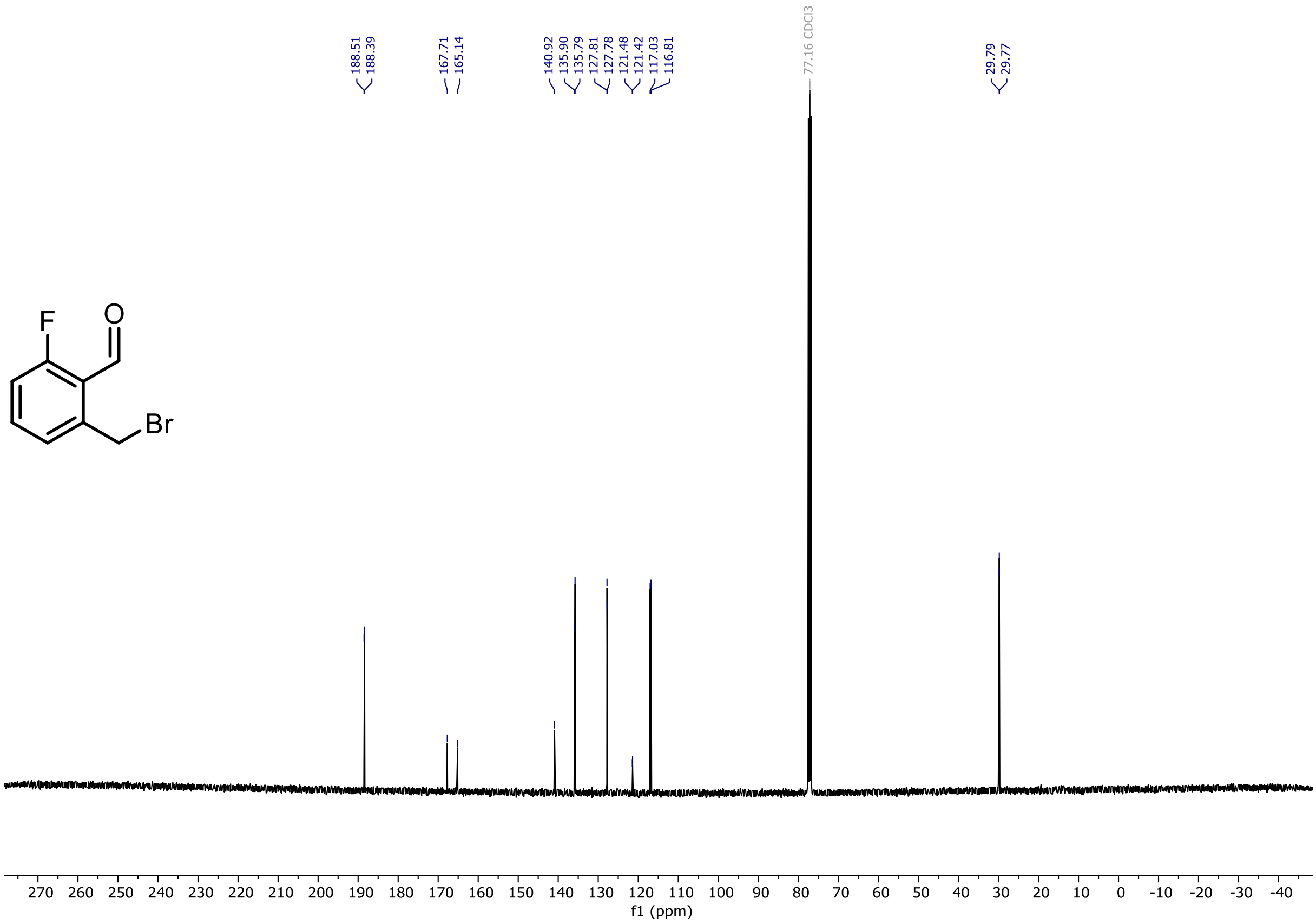

^{1}H NMR (400 MHz, $CDCl_3$; top) and ^{13}C NMR (101 MHz, $CDCl_3$; bottom) of compound **S14**

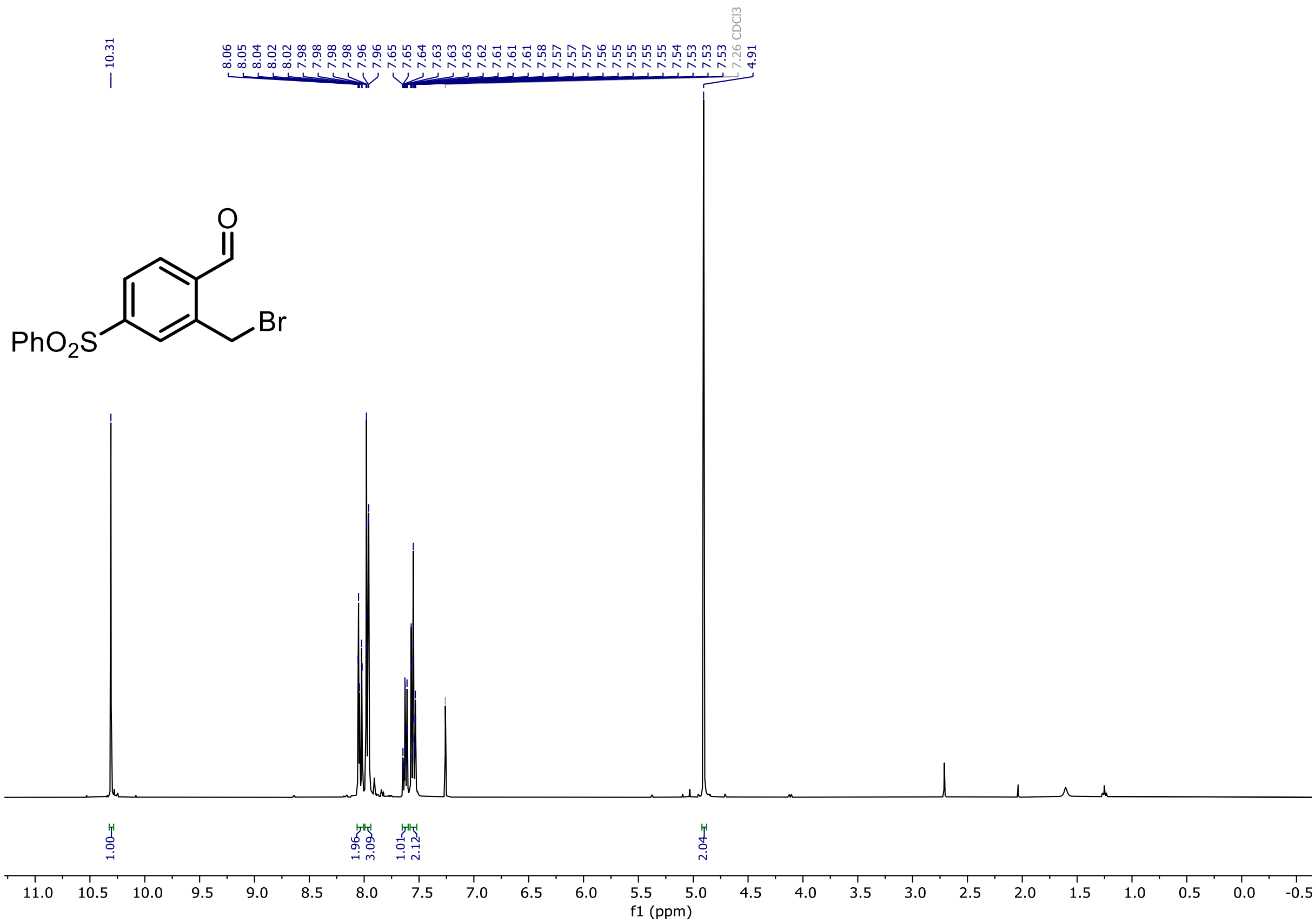

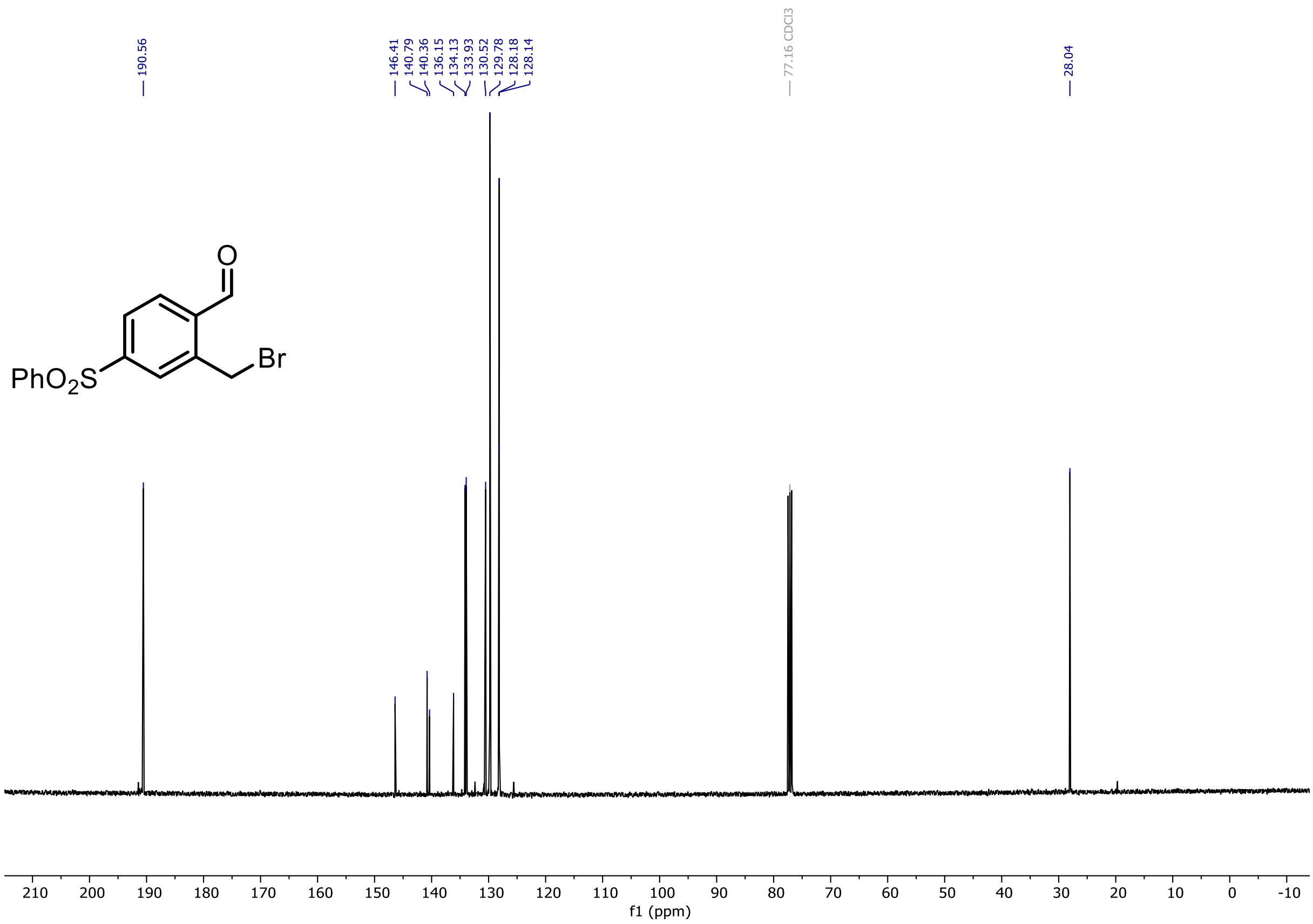

^{1}H NMR (600 MHz, $CDCl_3$; top) and ^{13}C NMR (151 MHz, $CDCl_3$; bottom) of compound **S15**

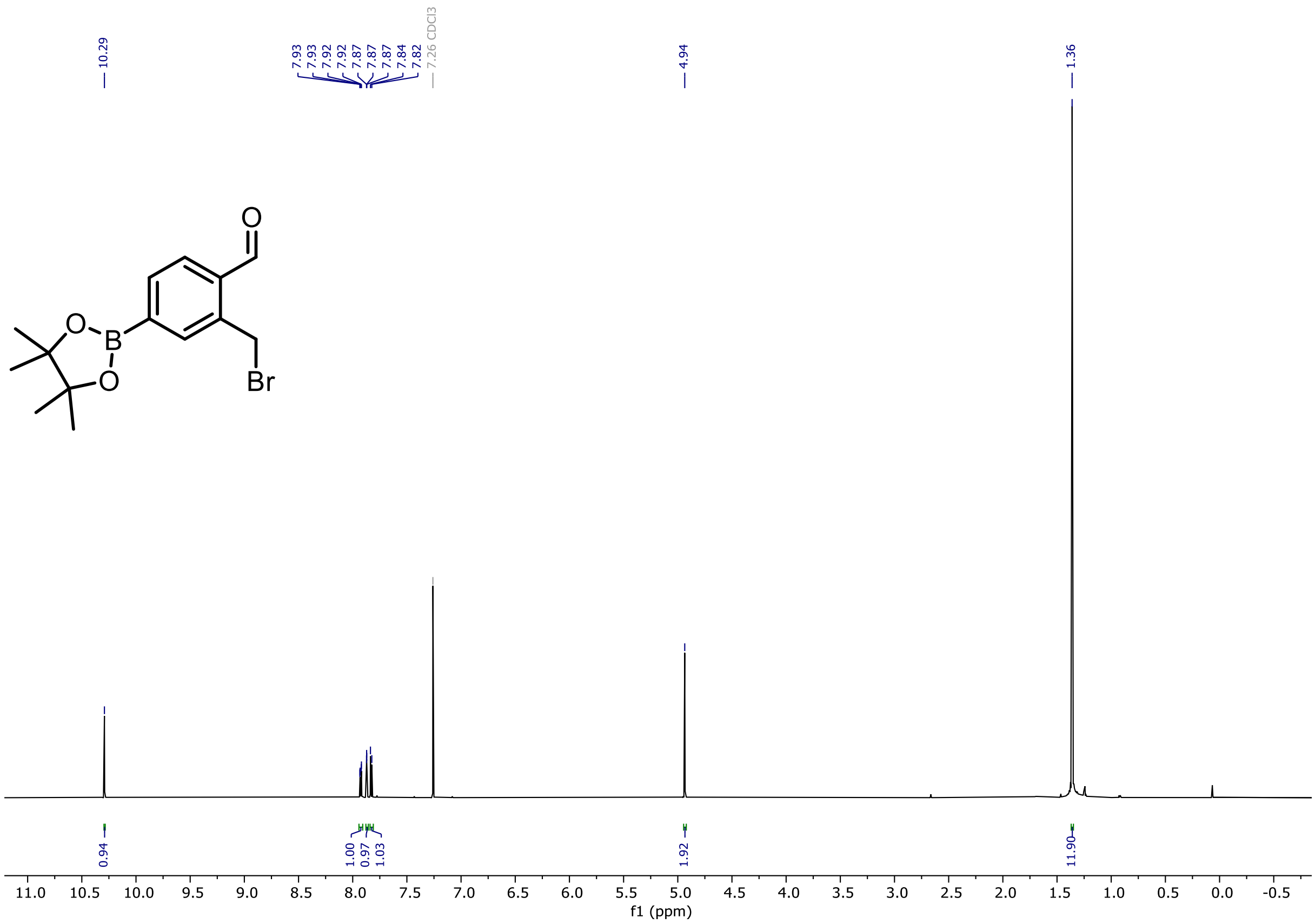

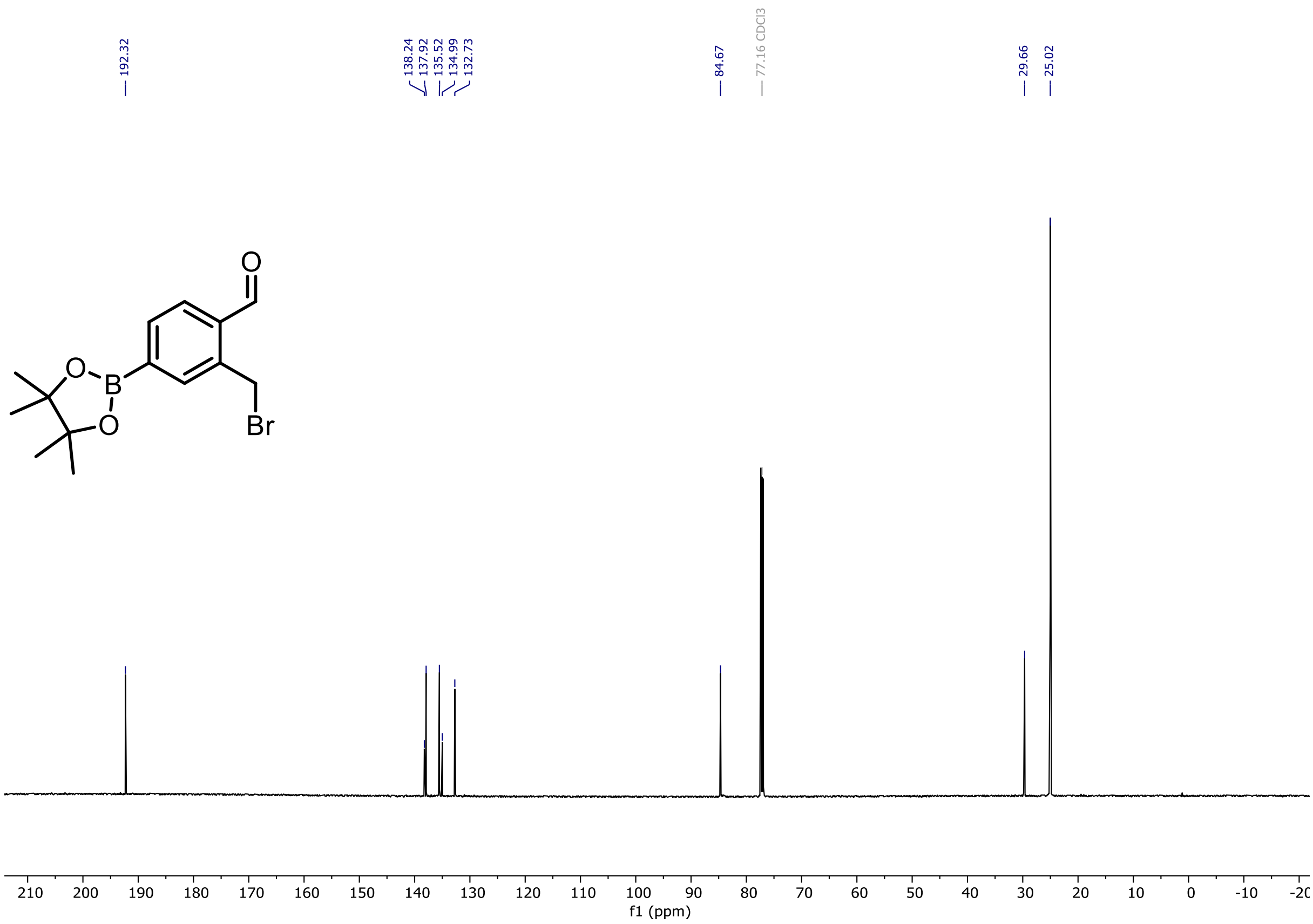

^{1}H NMR (600 MHz, $CDCl_3$; top) and ^{13}C NMR (151 MHz, $CDCl_3$; bottom) of compound **S16**

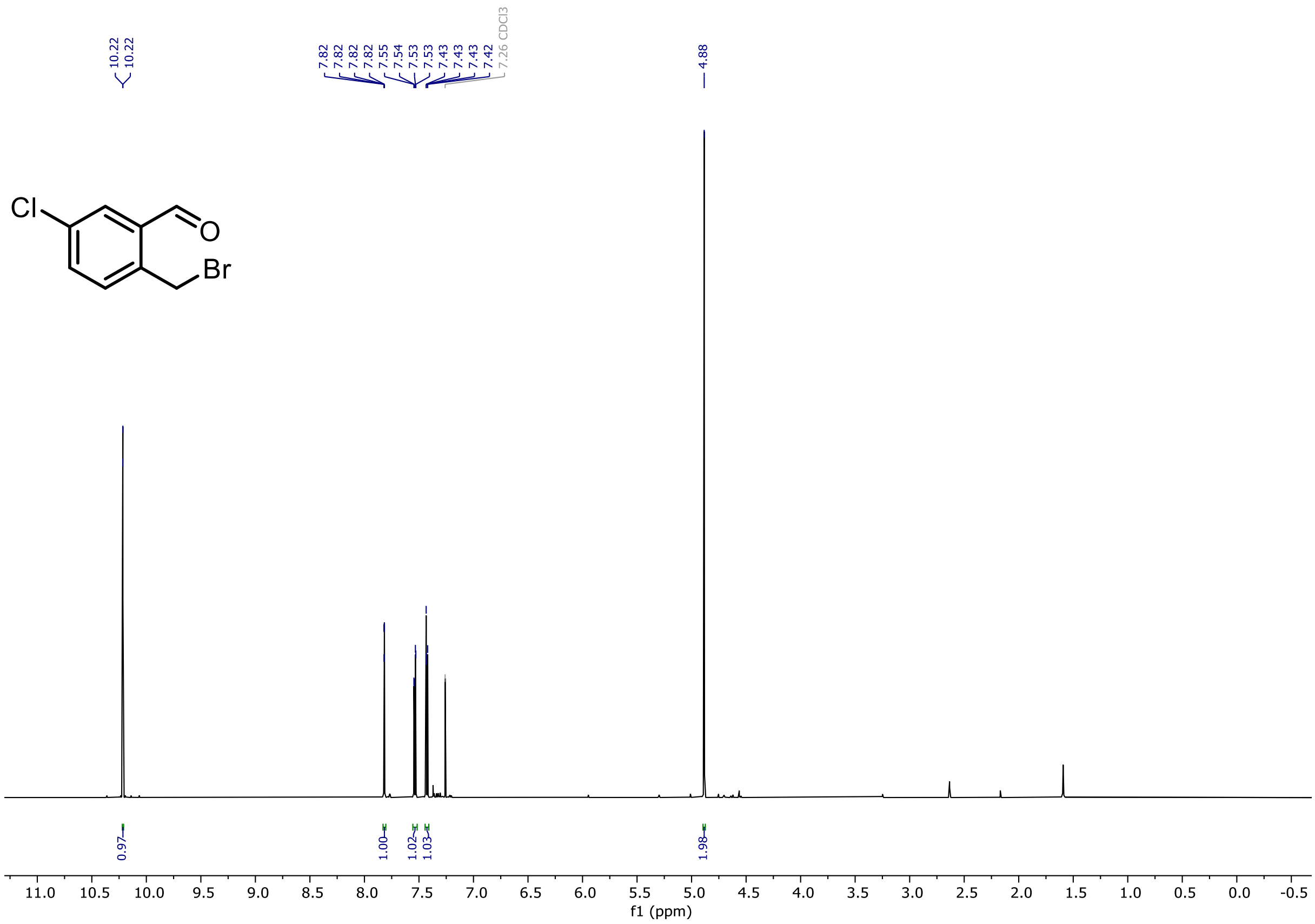

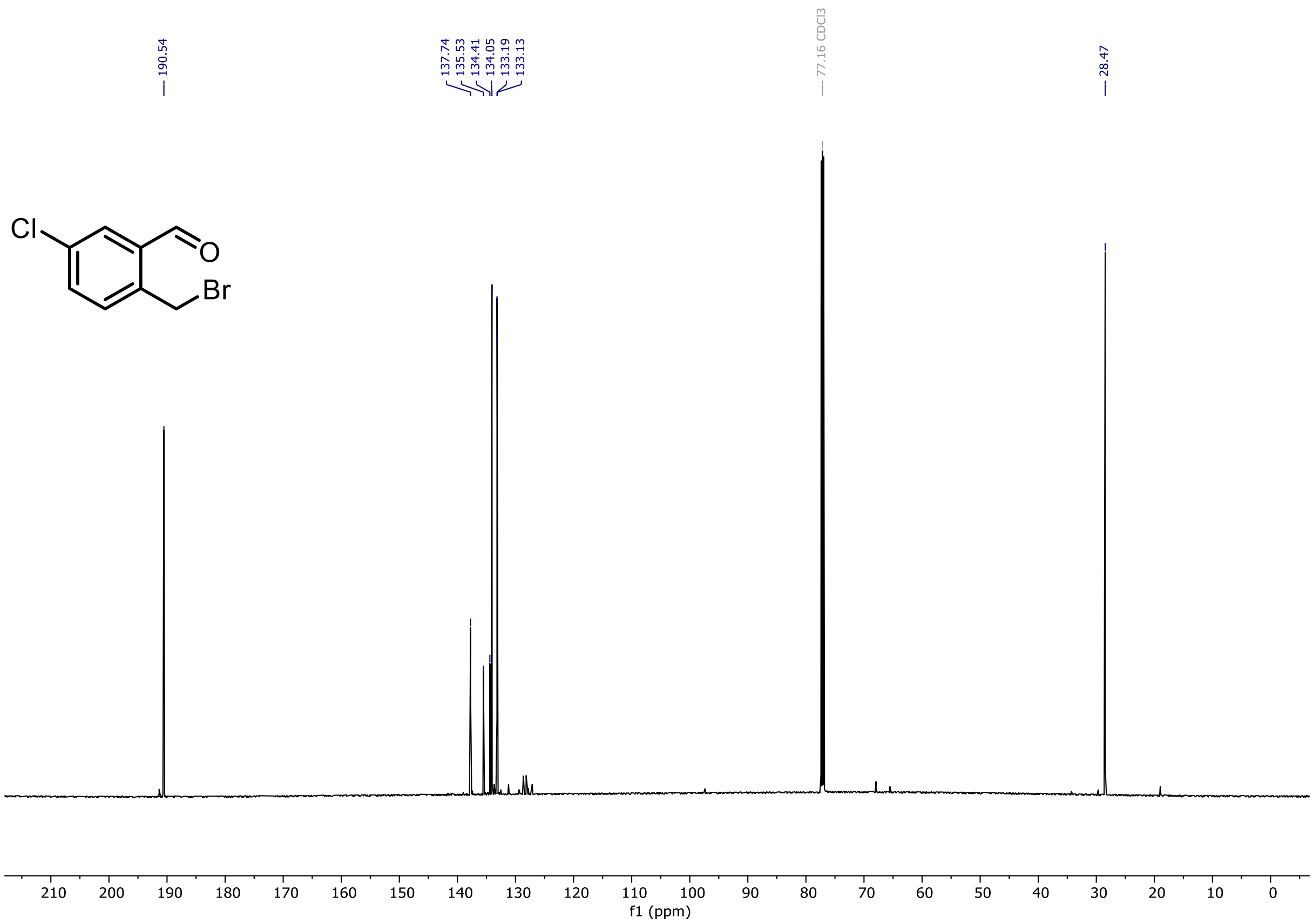

^{1}H NMR (600 MHz, $CDCl_3$; top) and ^{19}F NMR (585 MHz, $CDCl_3$; bottom) of compound **S17**

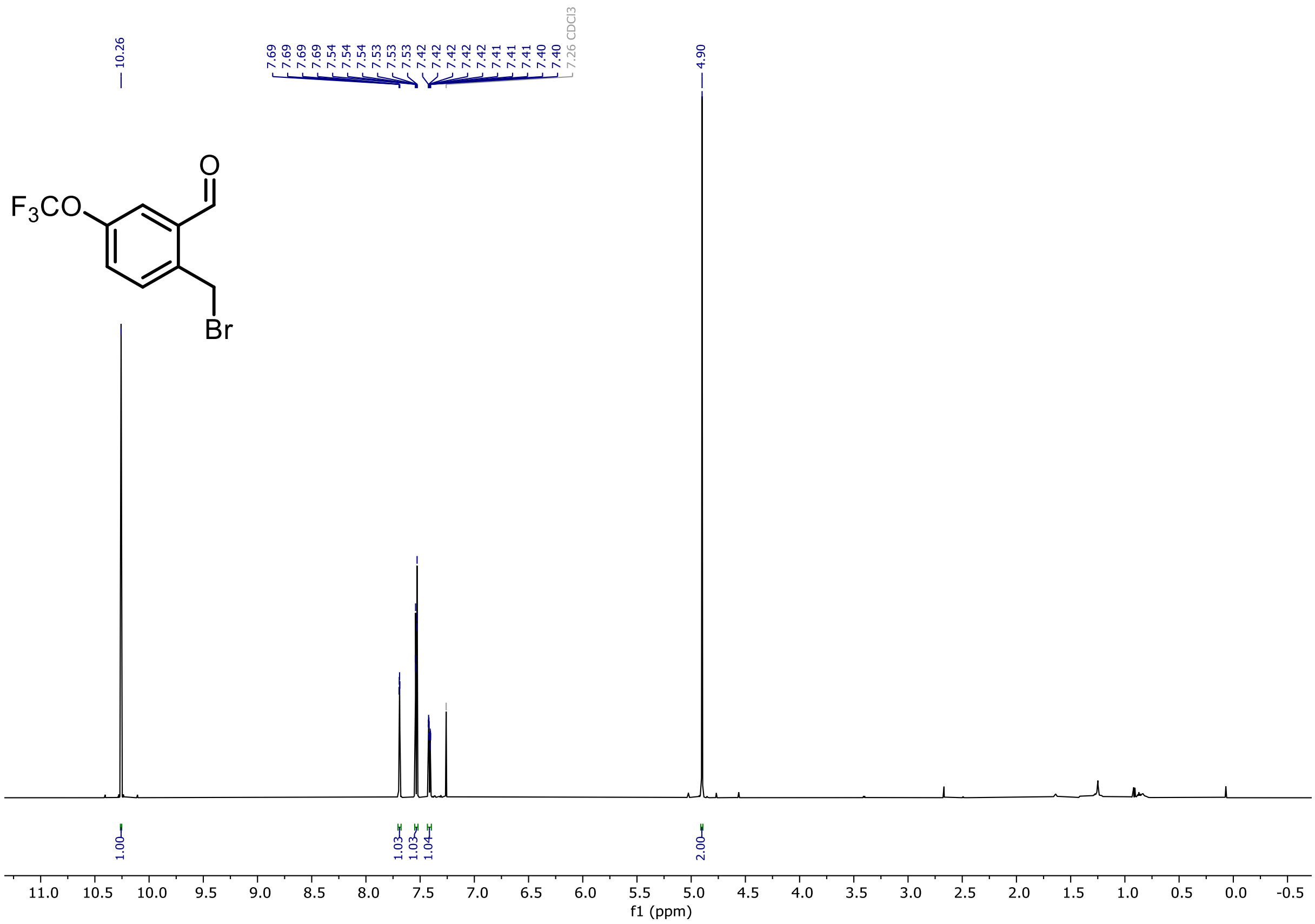

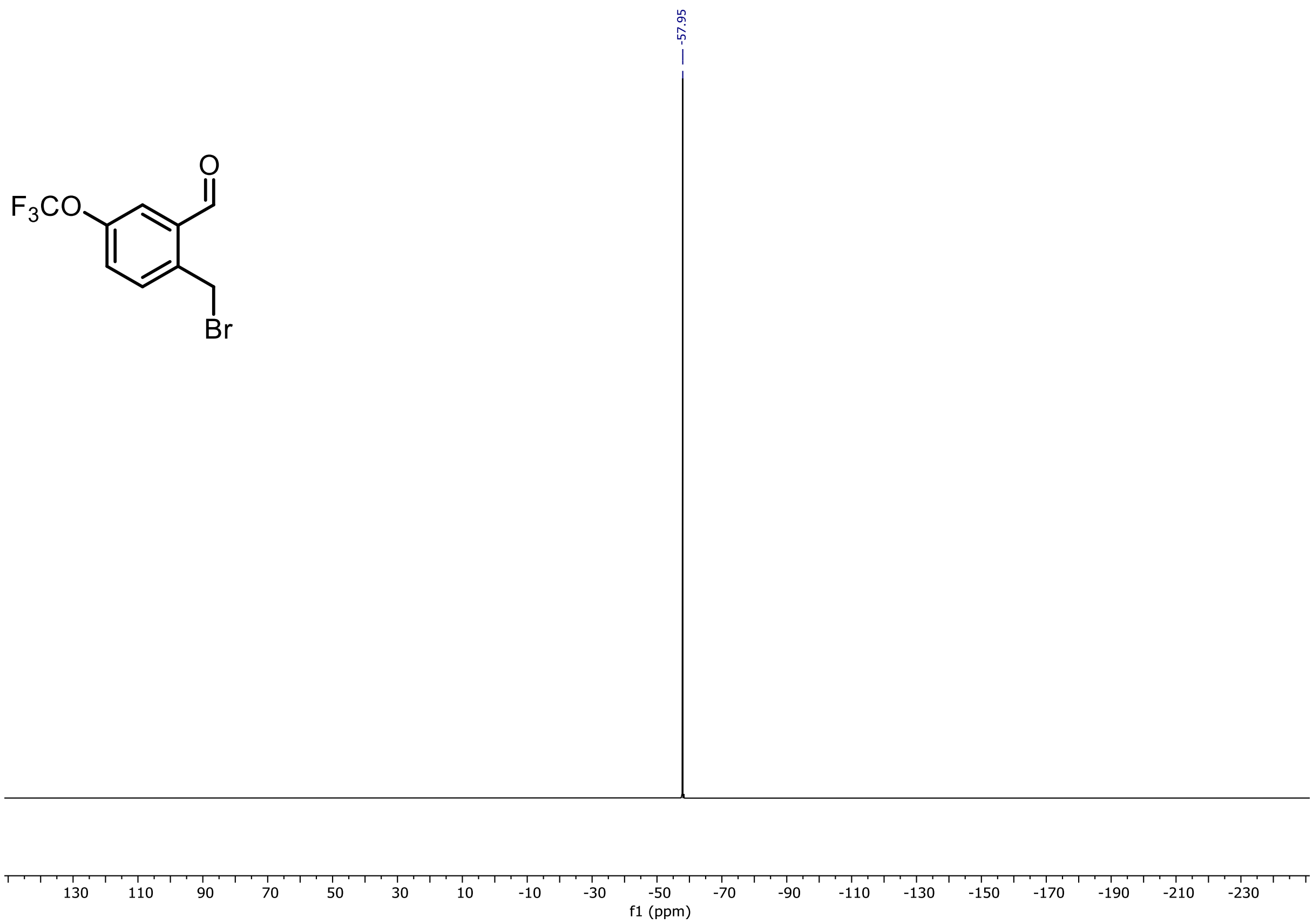

^{13}C NMR (151 MHz, $CDCl_3$) of compound **S17**

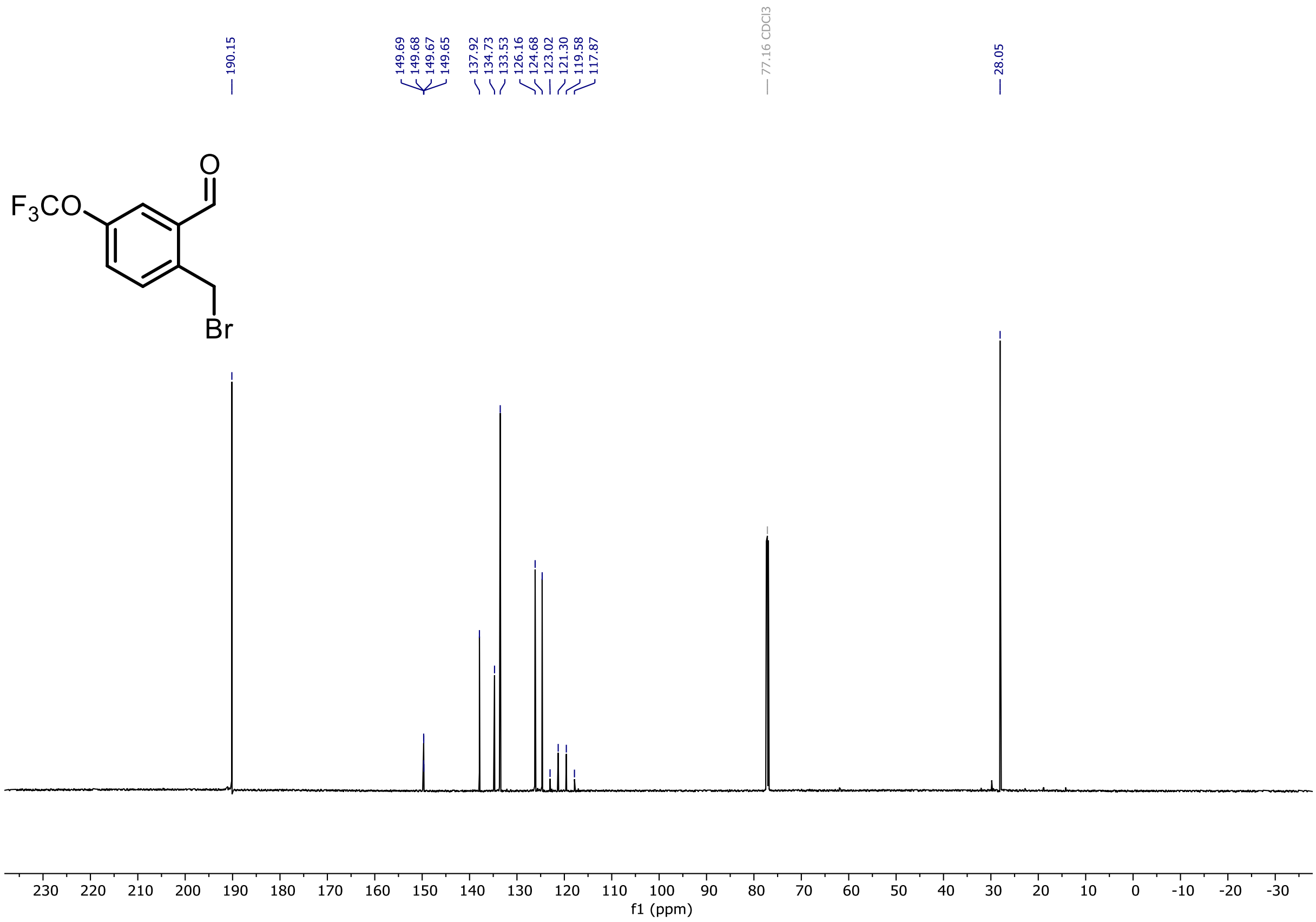

^{1}H NMR (400 MHz, $CDCl_3$; top) and ^{13}C NMR (101 MHz, $CDCl_3$; bottom) of compound **S18**

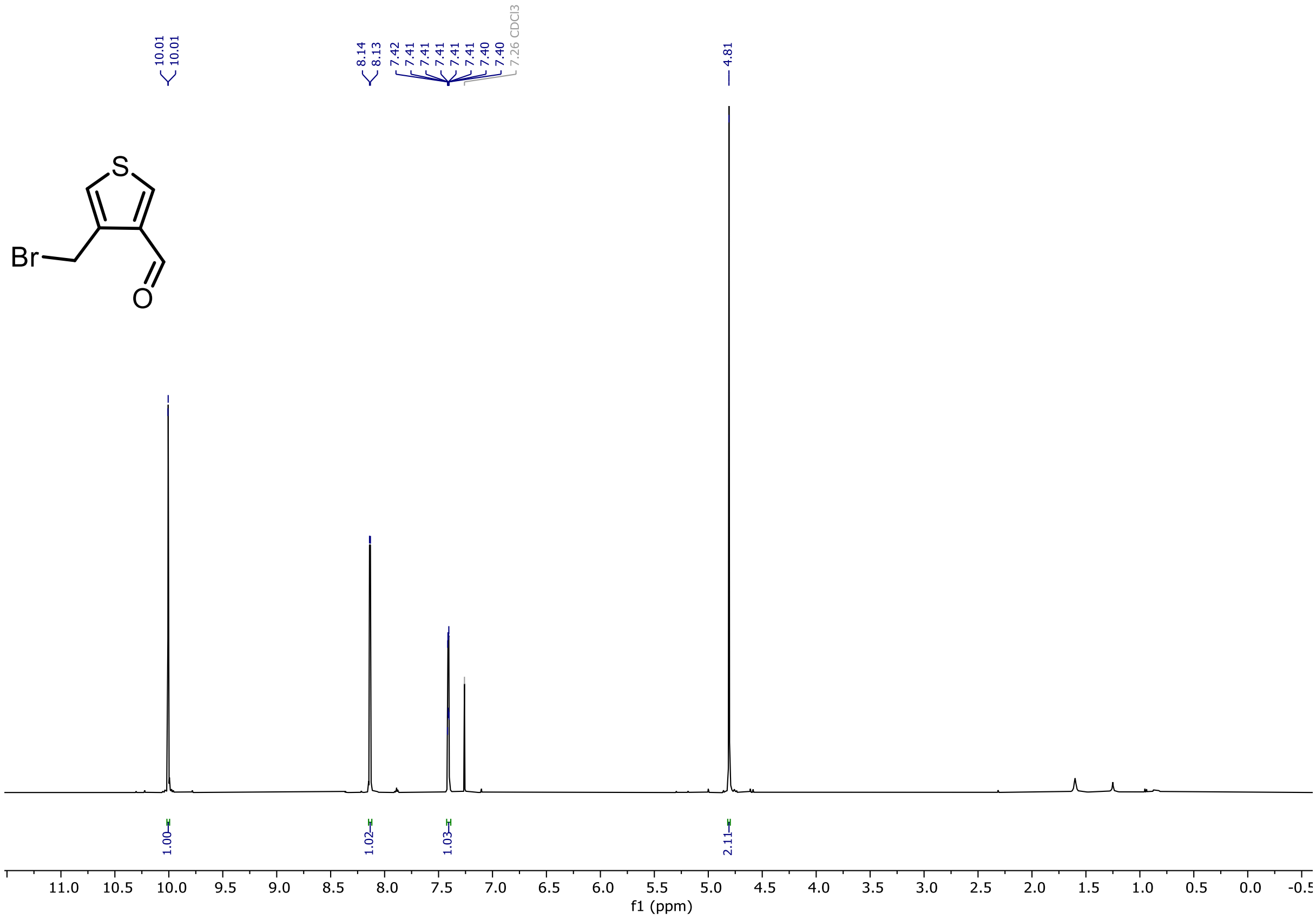

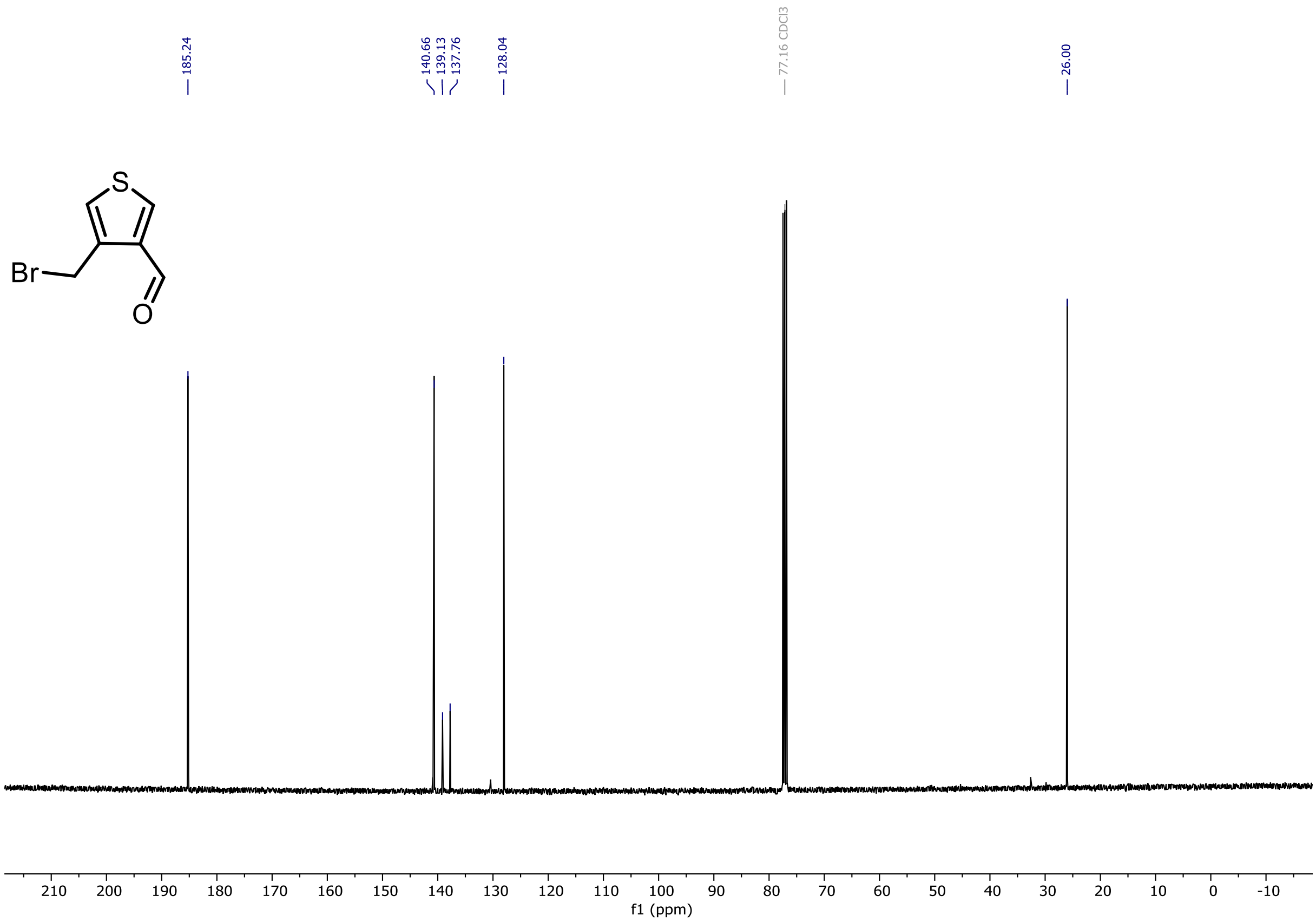

^{1}H NMR (400 MHz, $CDCl_3$; top) and ^{13}C NMR (101 MHz, $CDCl_3$; bottom) of compound **S19**

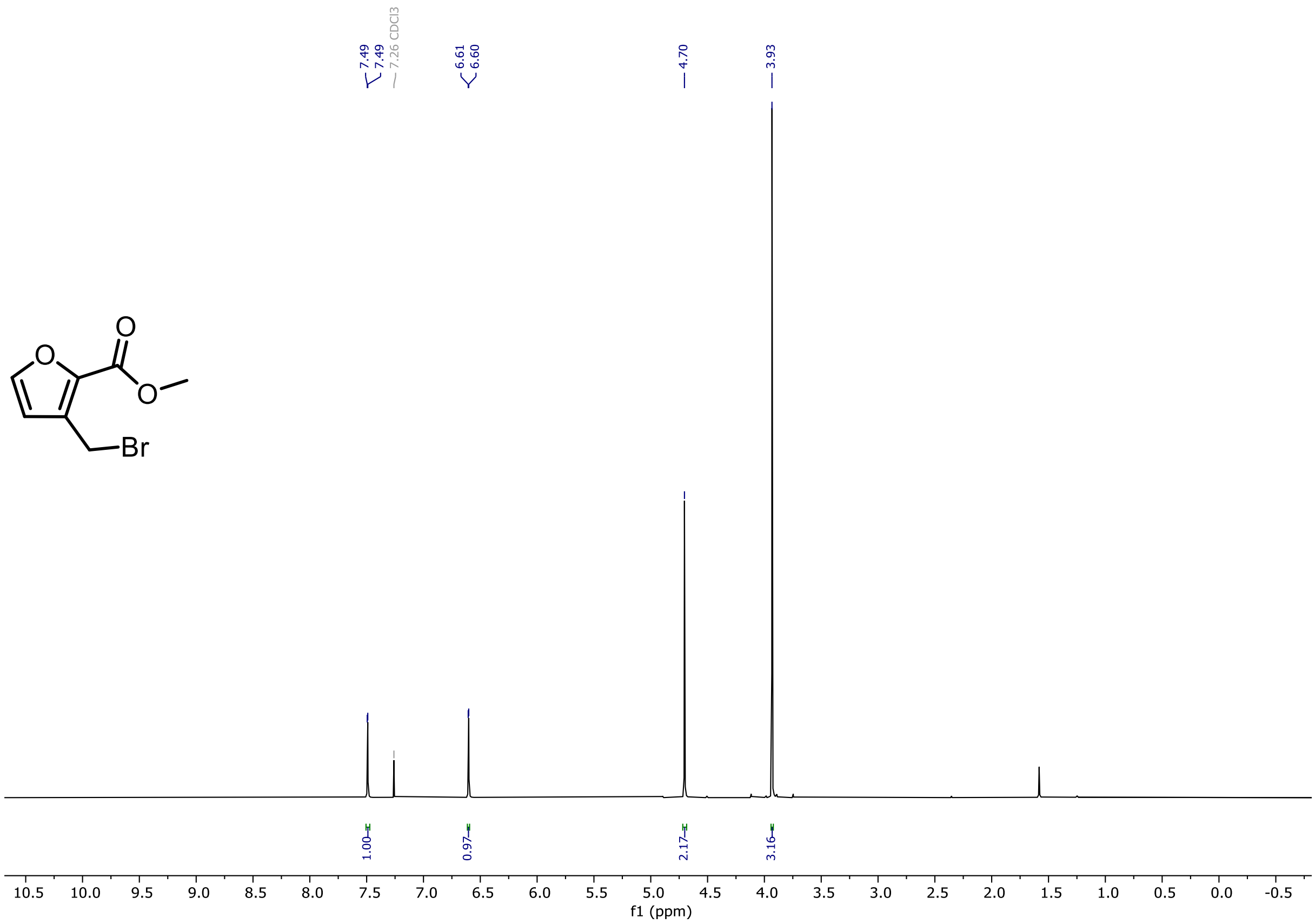

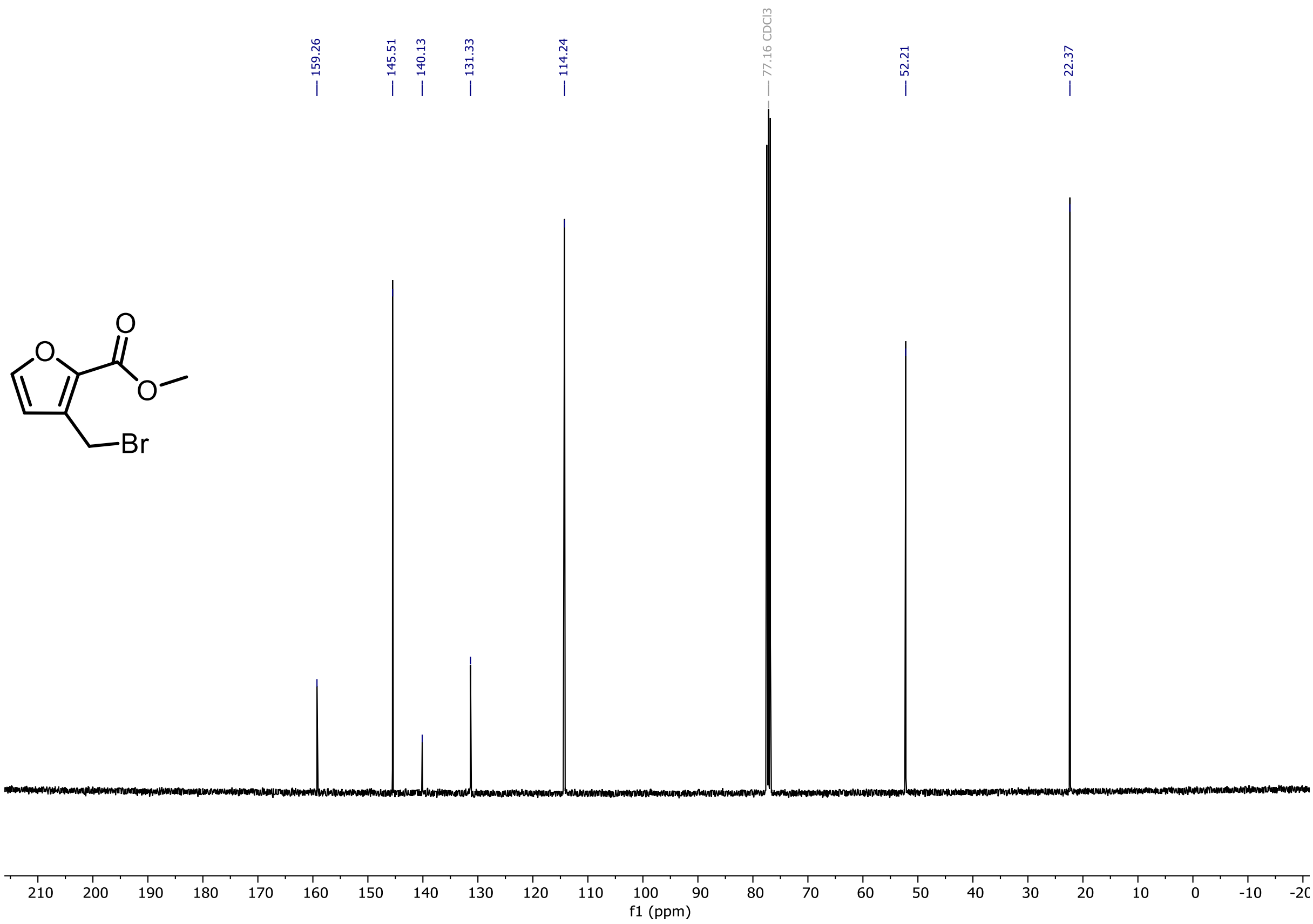

^{1}H NMR (400 MHz, $CDCl_3$; top) and ^{13}C NMR (101 MHz, $CDCl_3$; bottom) of compound **S20**

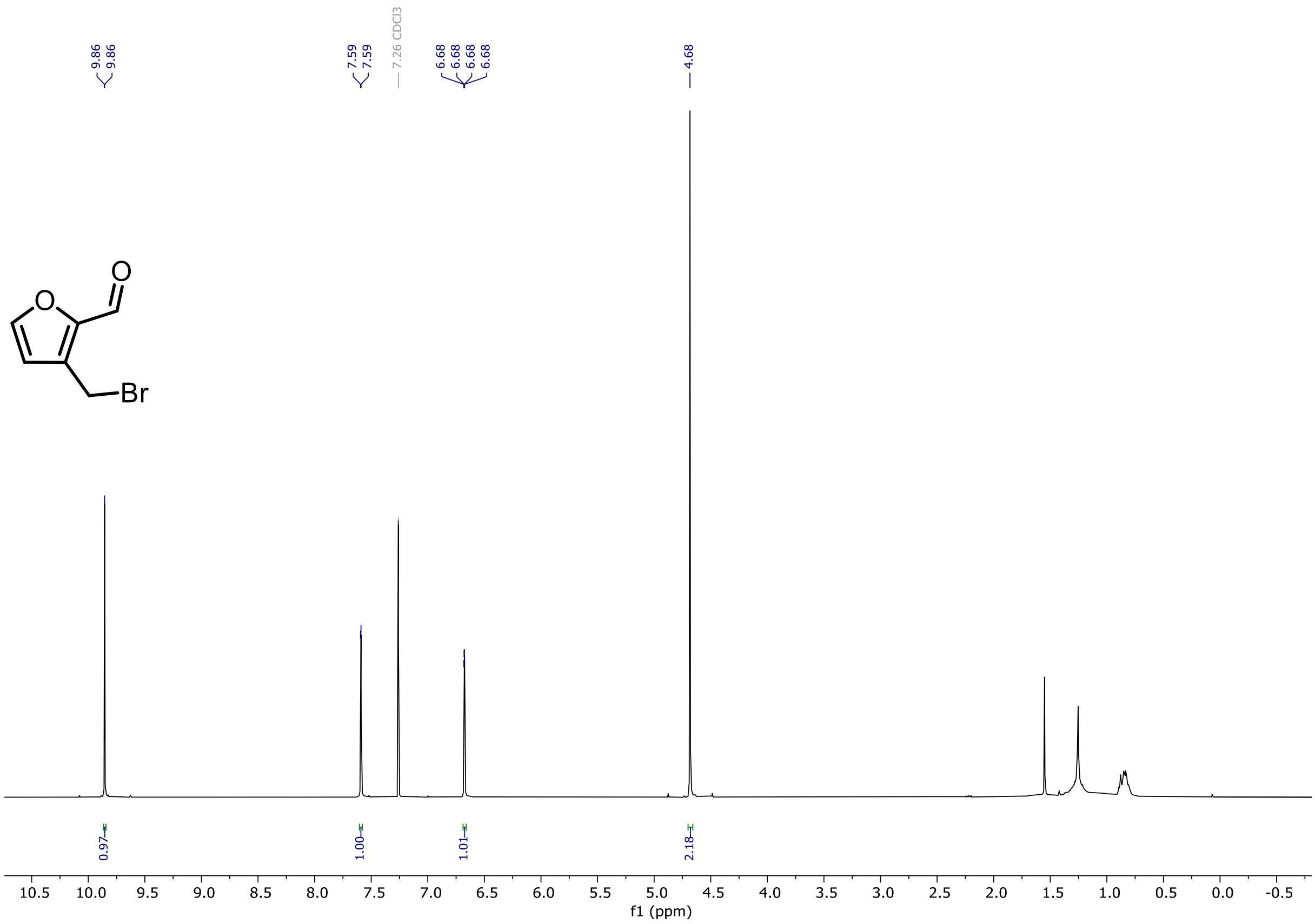

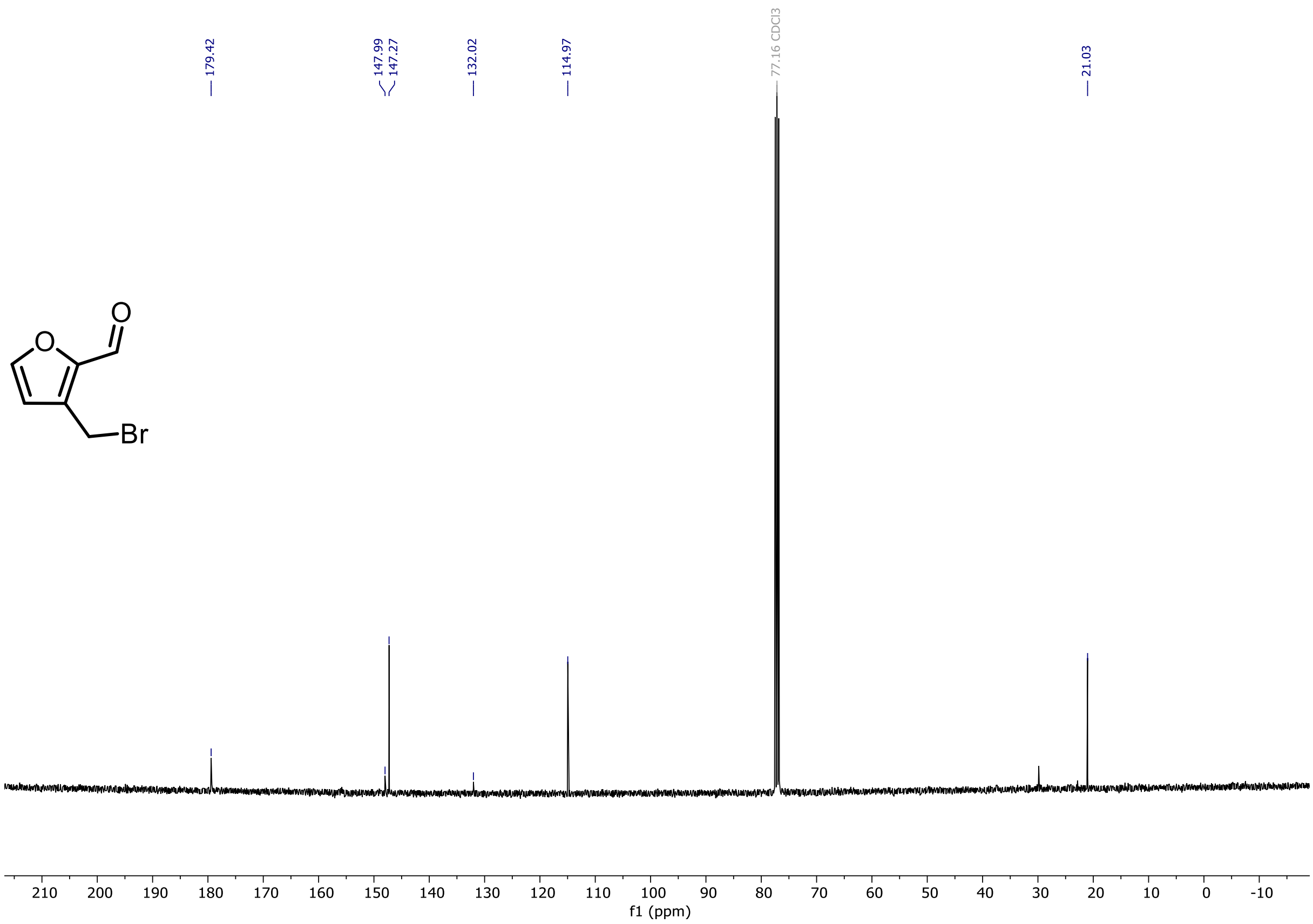

^1H NMR (400 MHz, $CDCl_3$; top) and ^{13}C NMR (101 MHz, $CDCl_3$; bottom) of compound **S23**

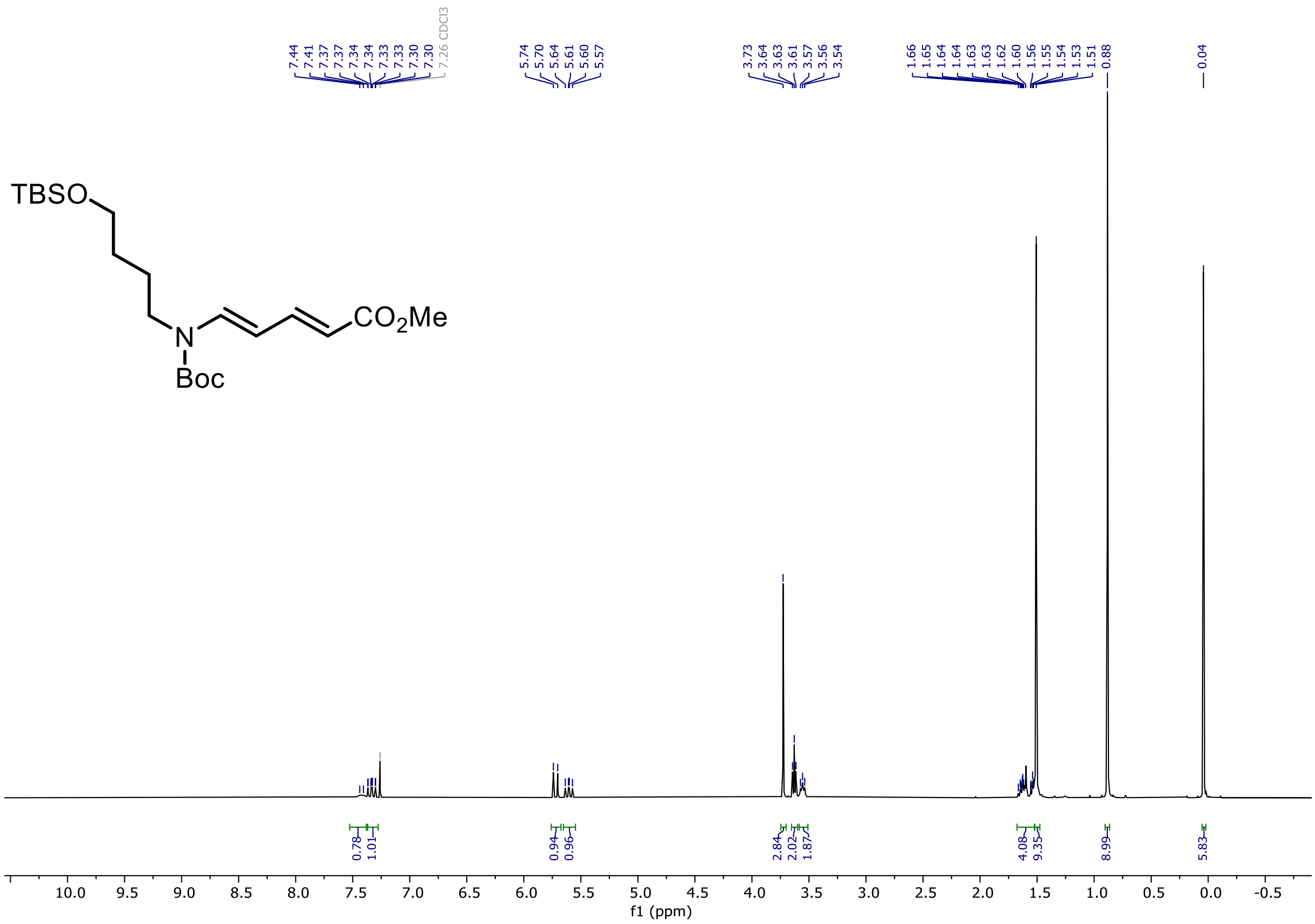

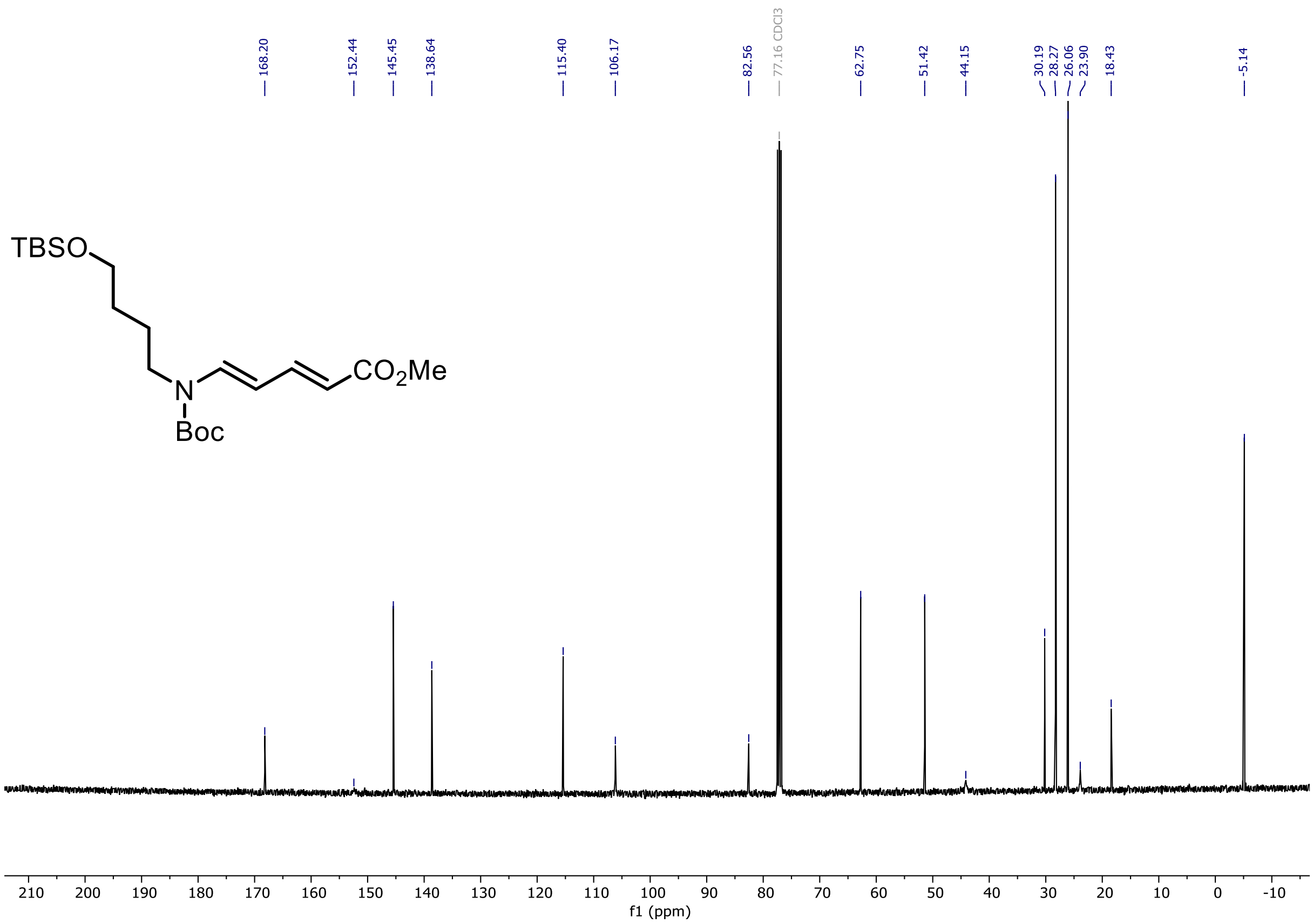

^{1}H NMR (600 MHz, $CDCl_3$; top) and ^{13}C NMR (151 MHz, $CDCl_3$; bottom) of compound **S24**

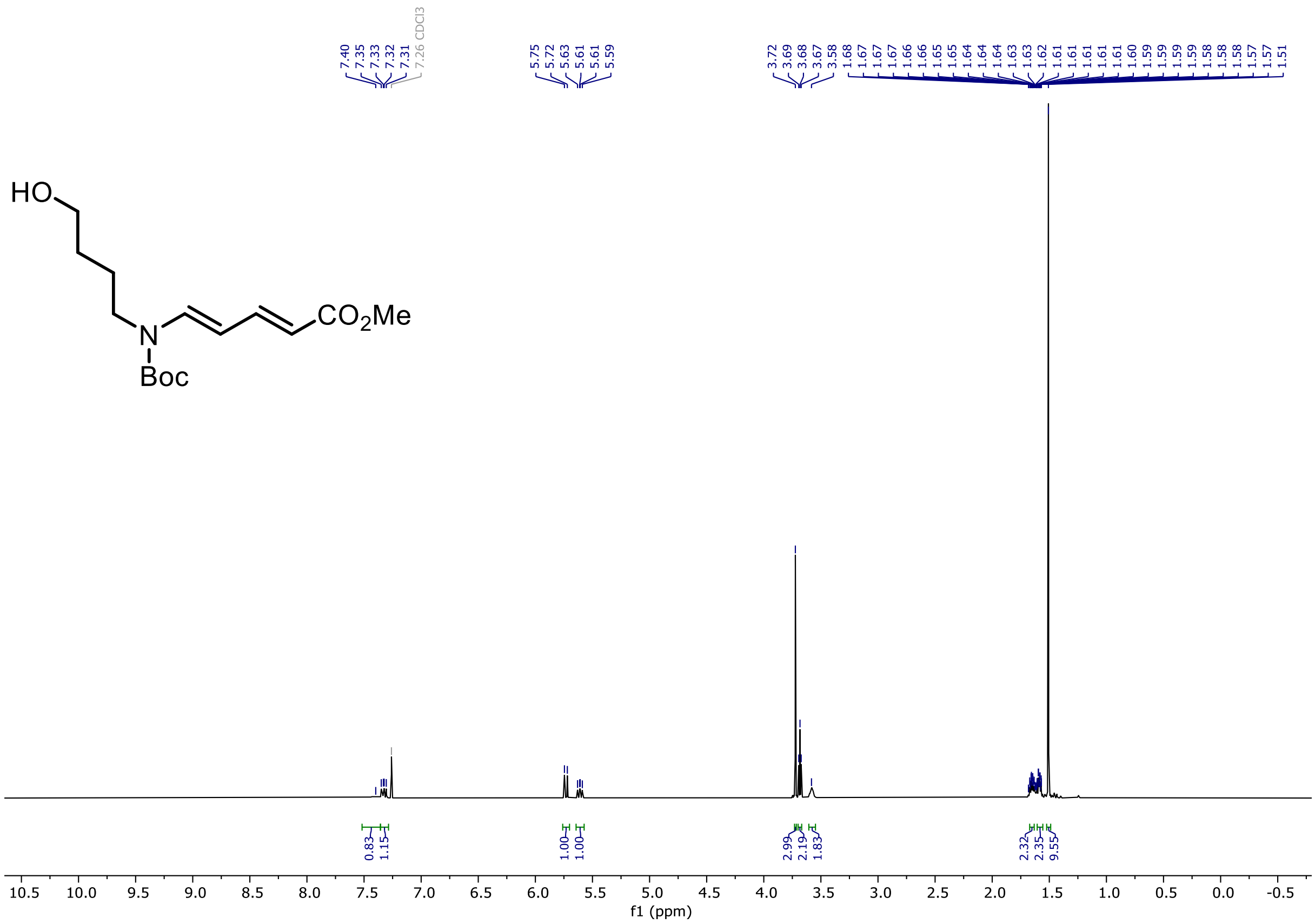

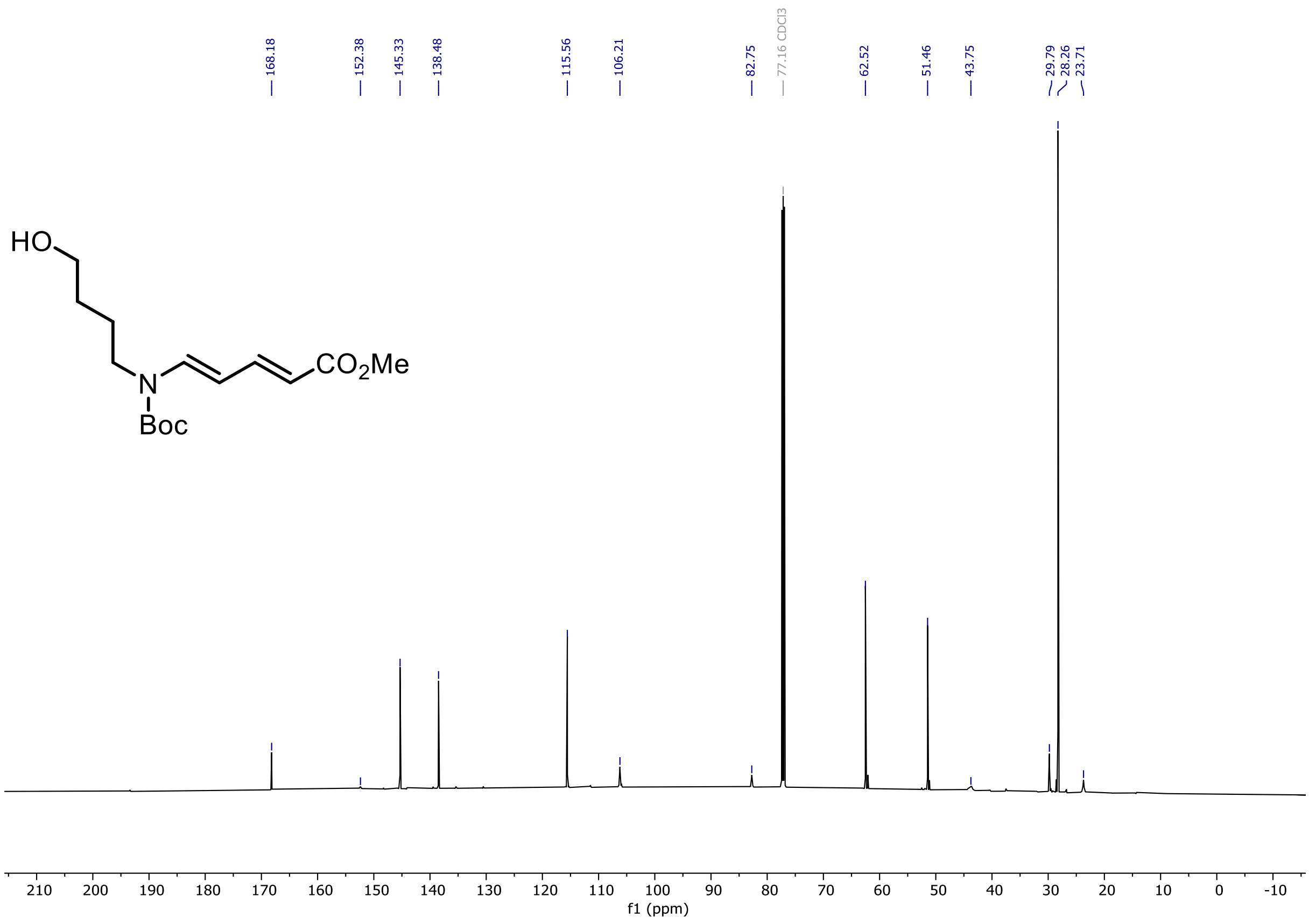

^{1}H NMR (400 MHz, $CDCl_3$; top) and ^{13}C NMR (101 MHz, $CDCl_3$; bottom) of compound **S25**

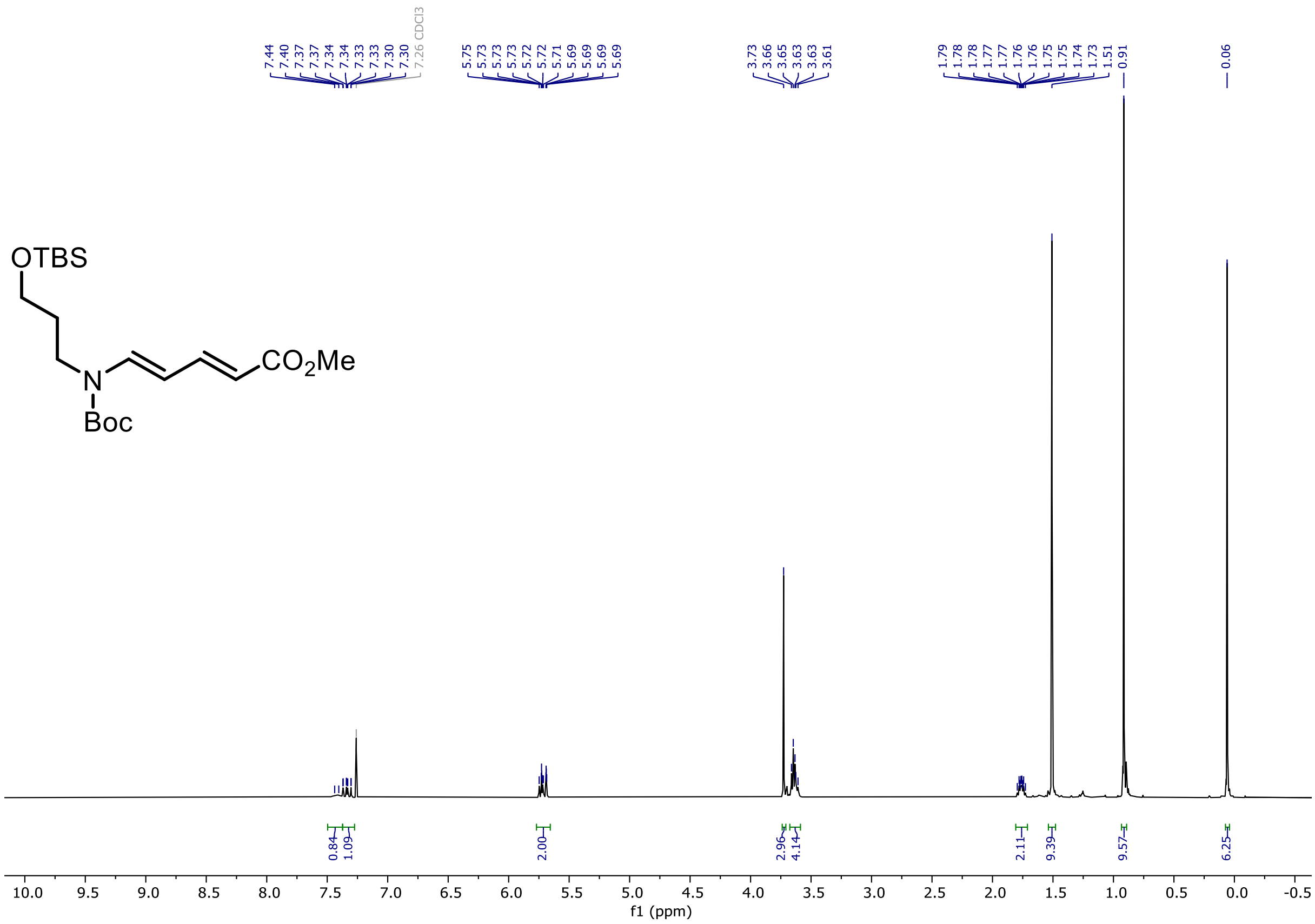

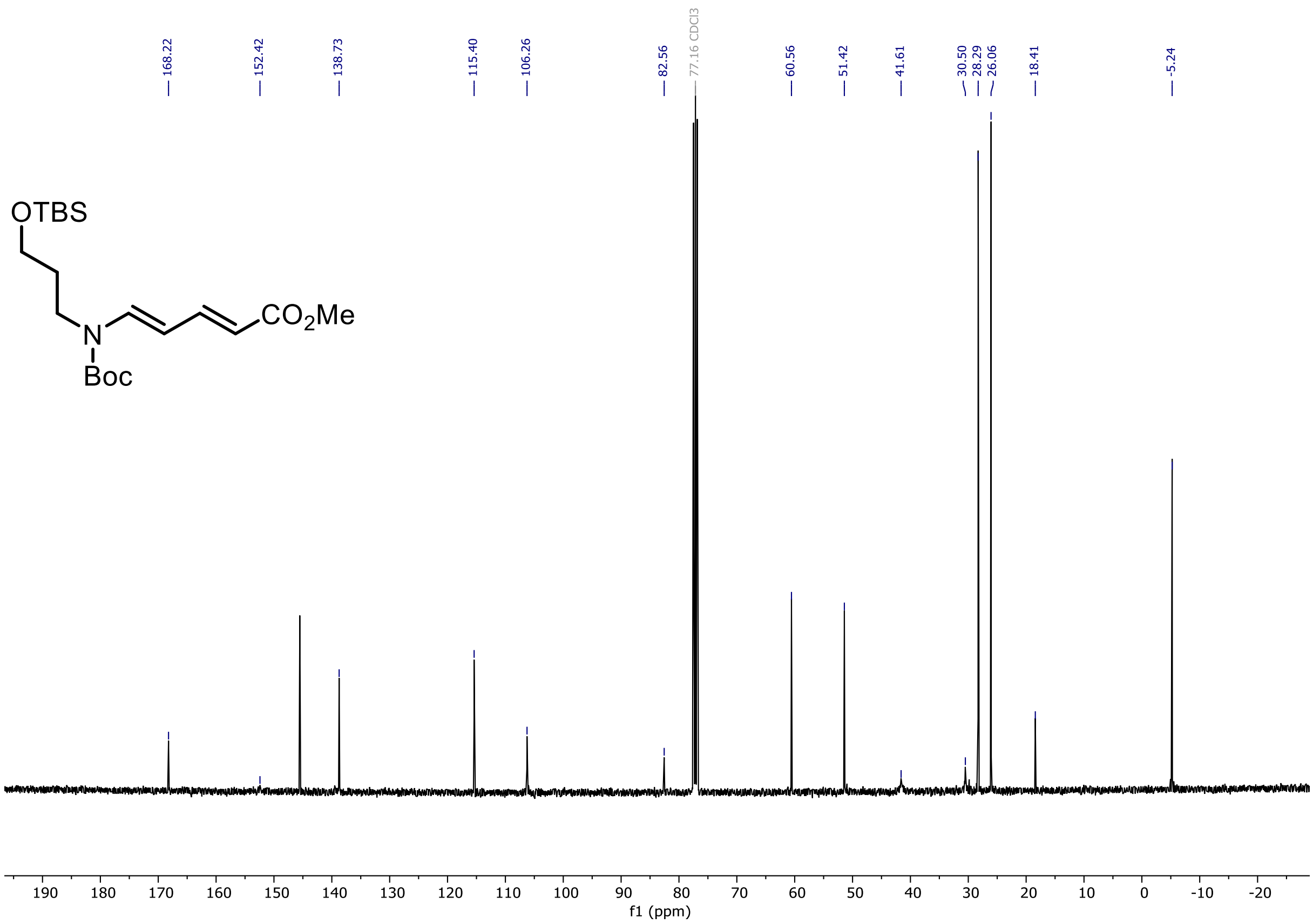

^{1}H NMR (400 MHz, $CDCl_3$; top) and ^{13}C NMR (101 MHz, $CDCl_3$; bottom) of compound **S26**

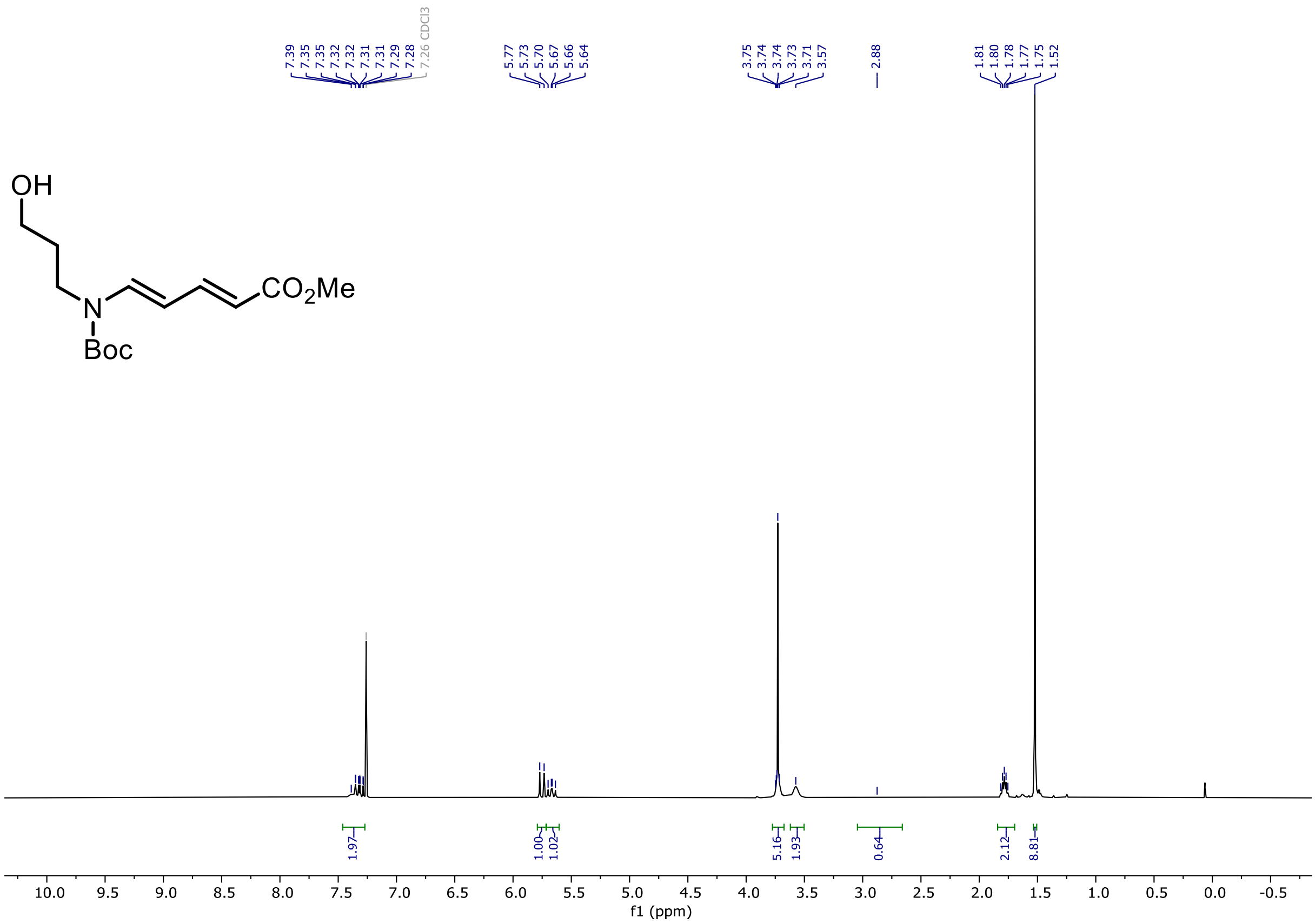

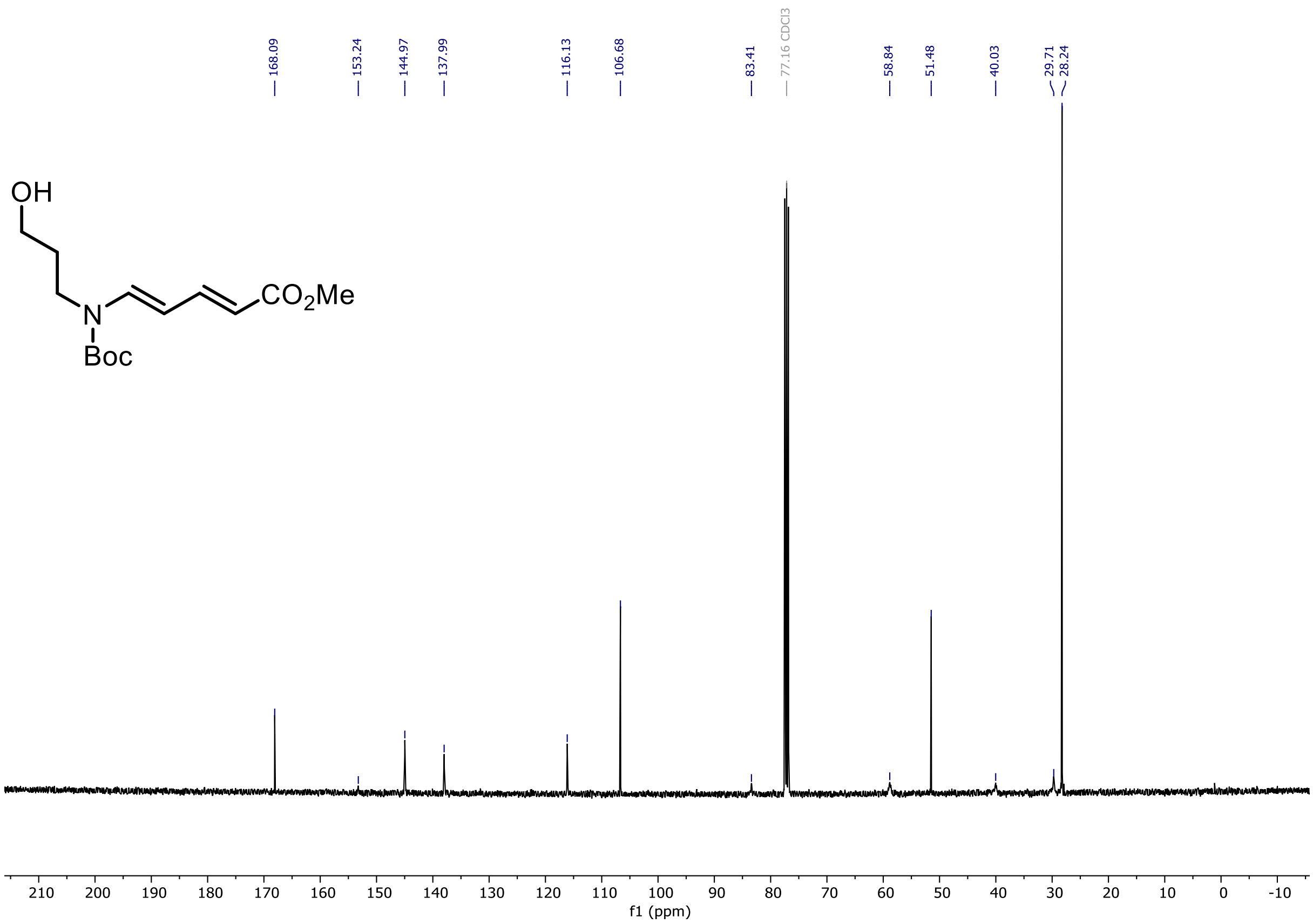

^1H NMR (400 MHz, $CDCl_3$; top) and ^{13}C NMR (101 MHz, $CDCl_3$; bottom) of compound **S27**

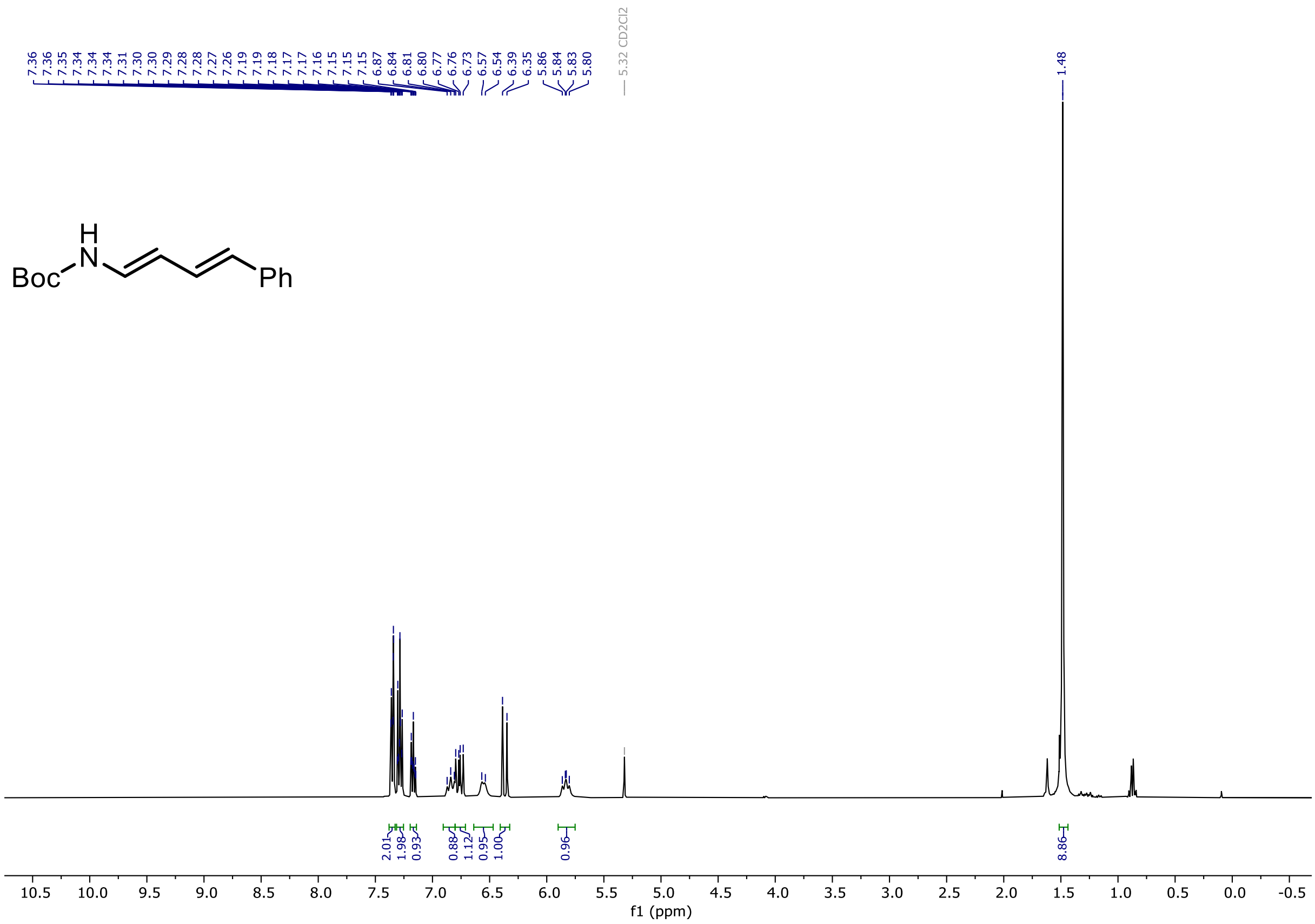

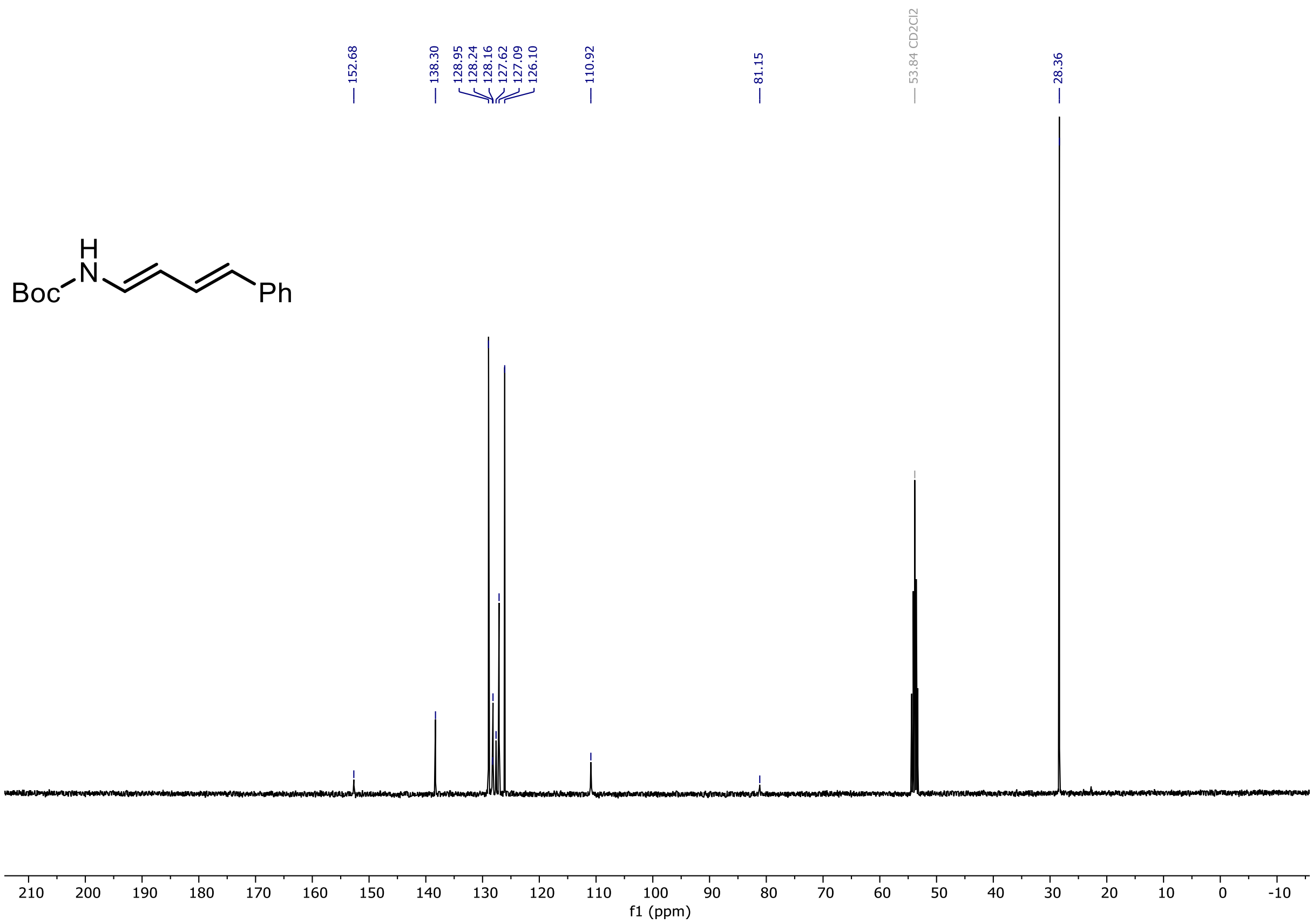

^{1}H NMR (400 MHz, $CDCl_3$; top) and ^{13}C NMR (101 MHz, $CDCl_3$; bottom) of compound **7a**

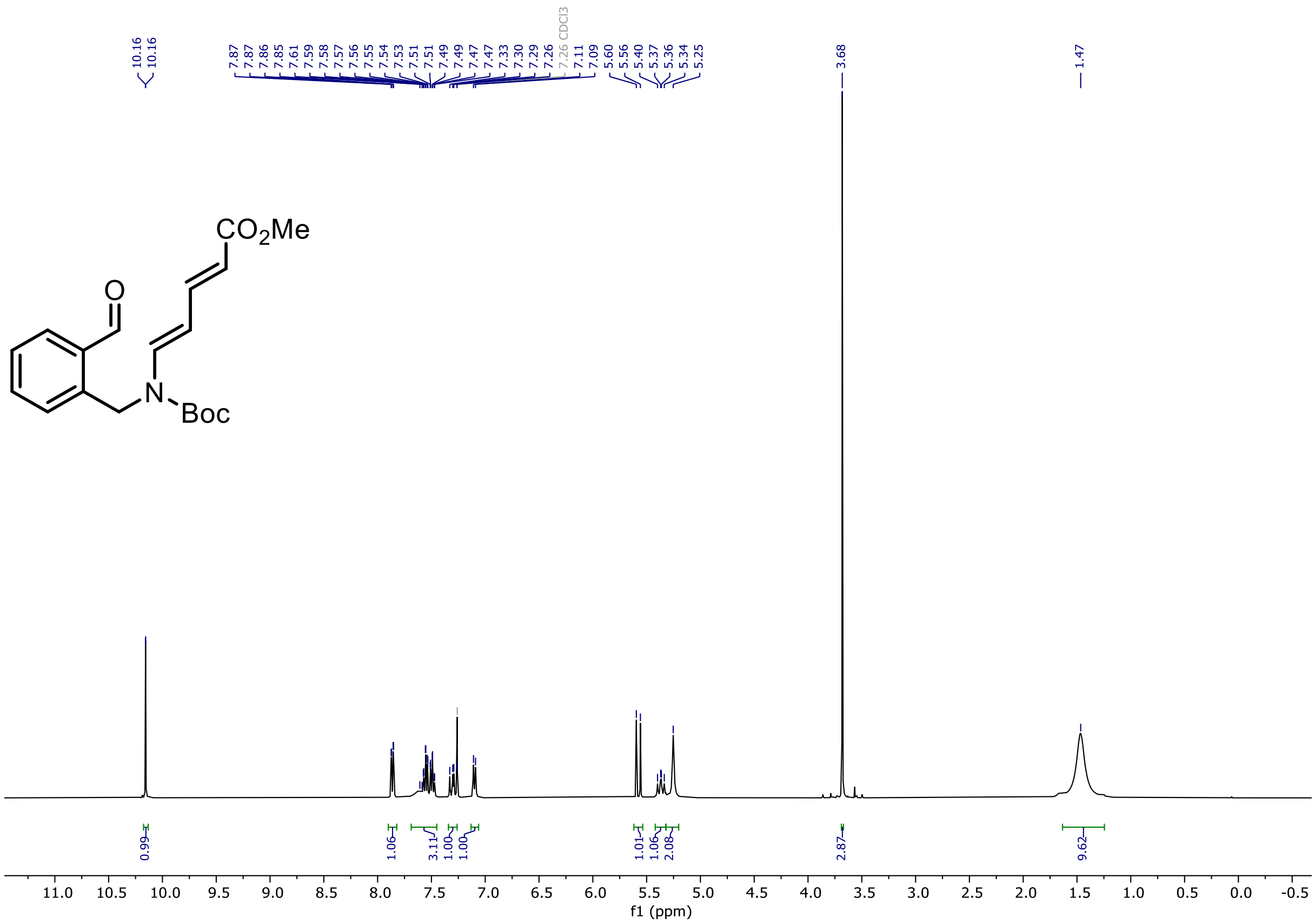

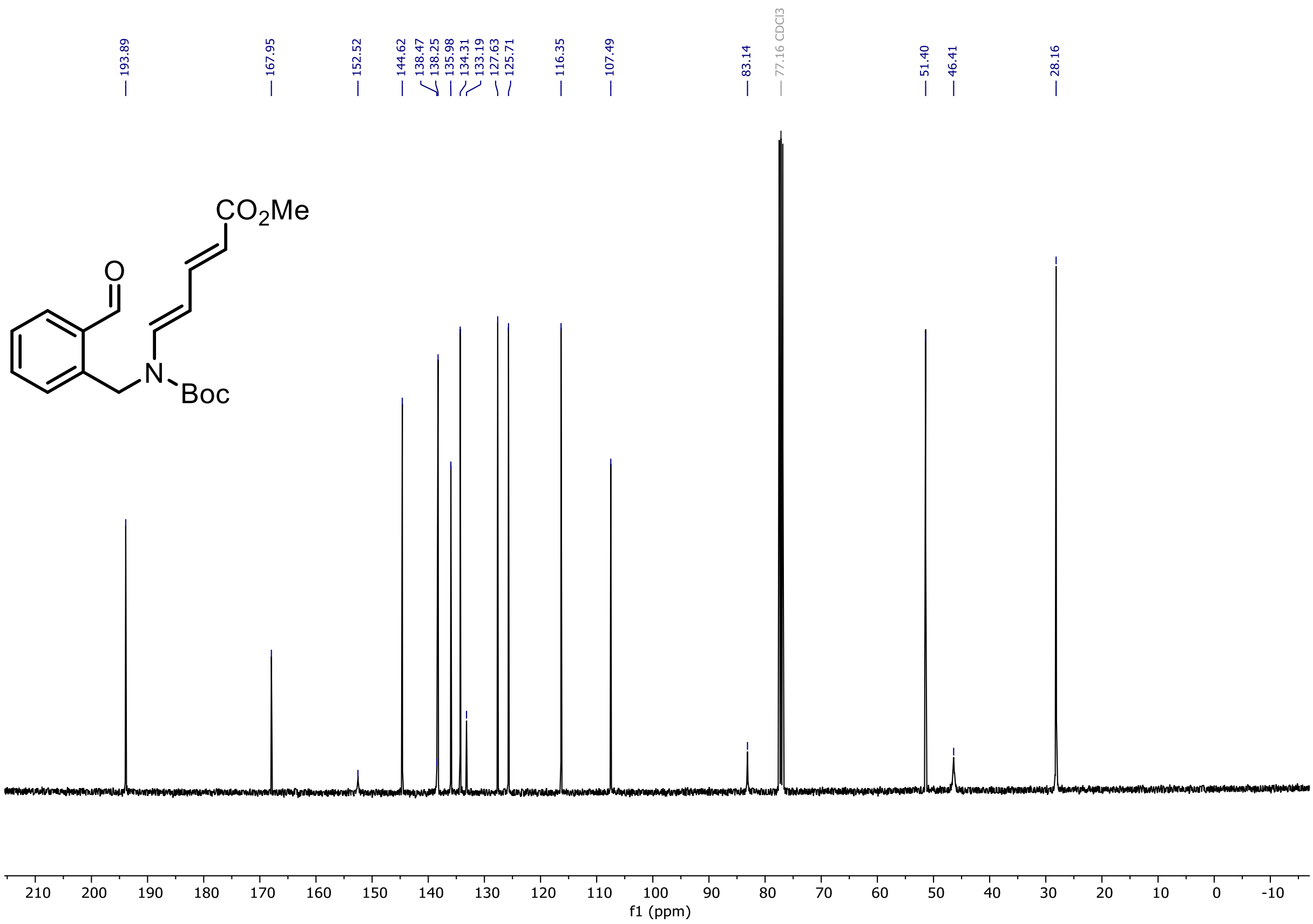

^{1}H NMR (600 MHz, [D$_6$]-DMSO, 383K ; top) and ^{13}C NMR (151 MHz, [D$_6$]-DMSO, 383K; bottom) of compound **7b**

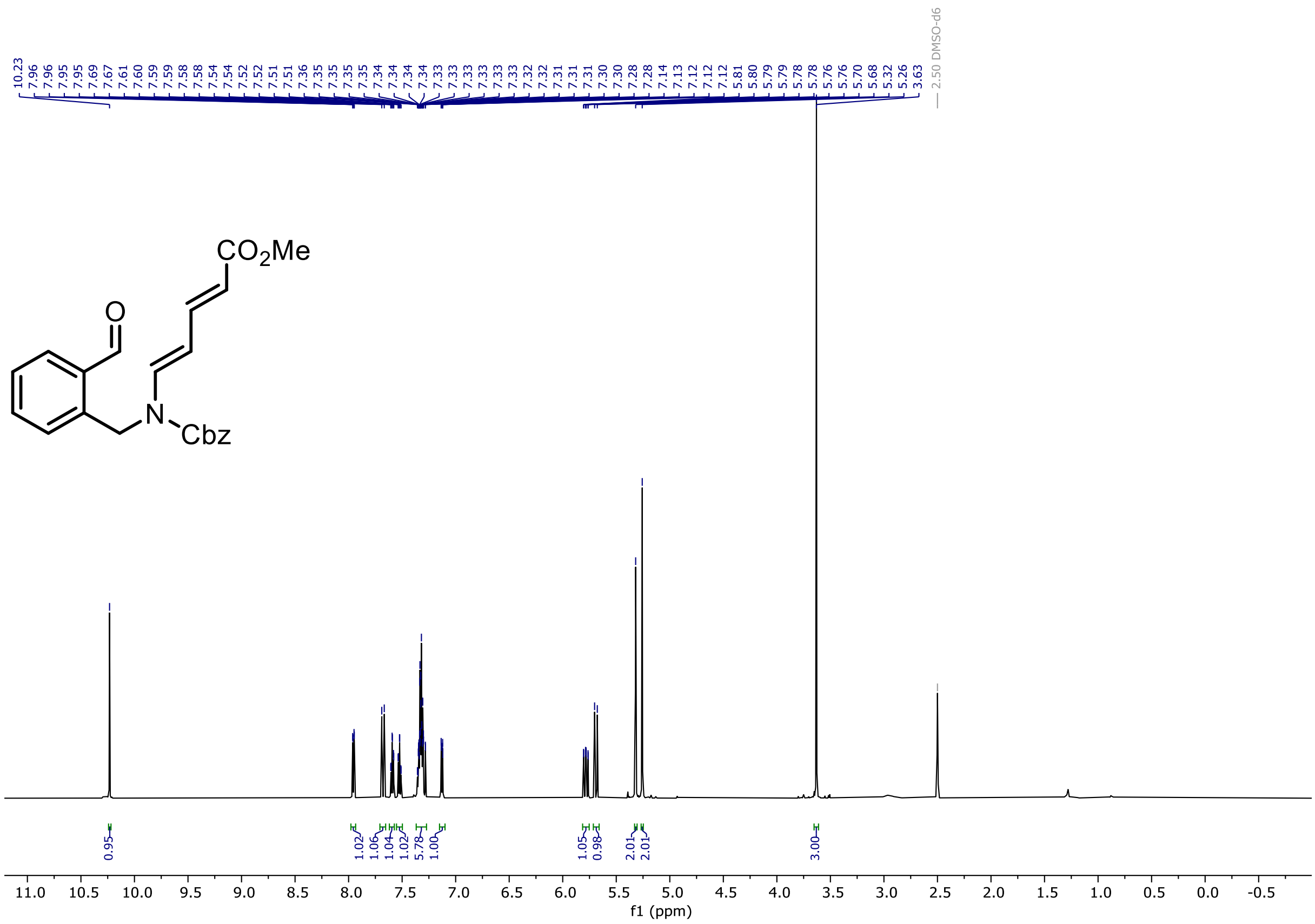

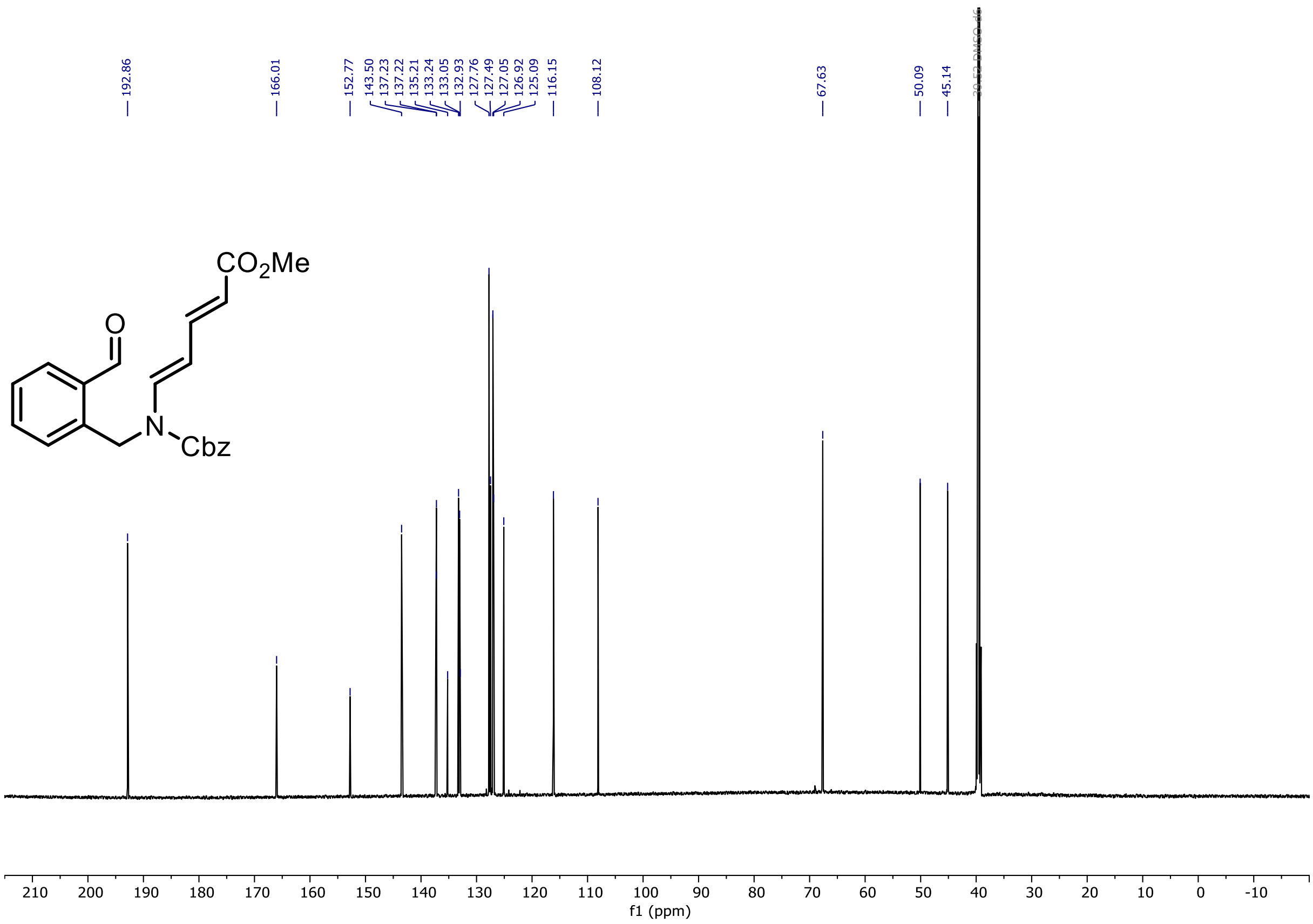

^{1}H NMR (600 MHz, $CDCl_3$; top) and ^{19}F NMR (282 MHz, $CDCl_3$; bottom) of compound **7c**

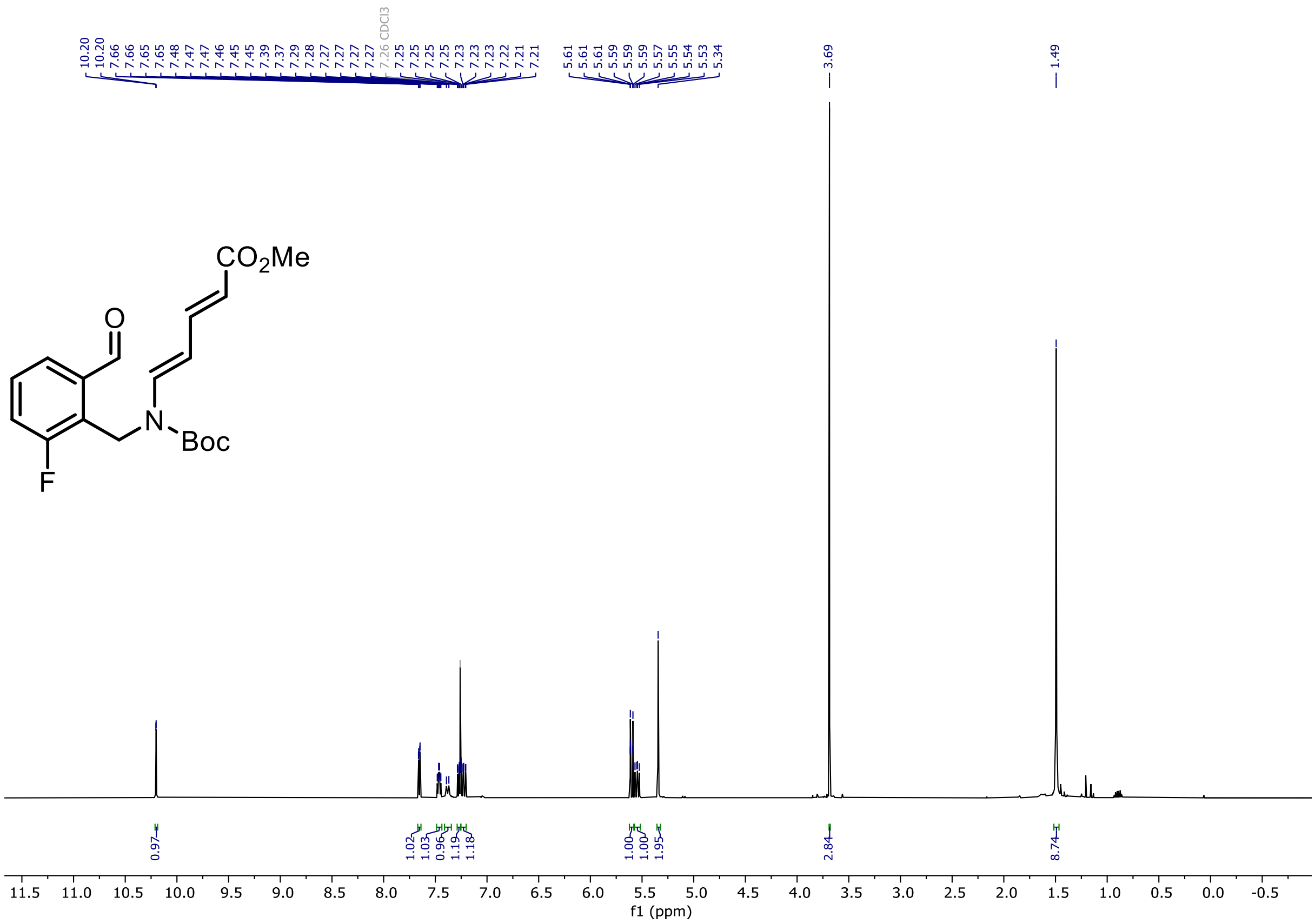

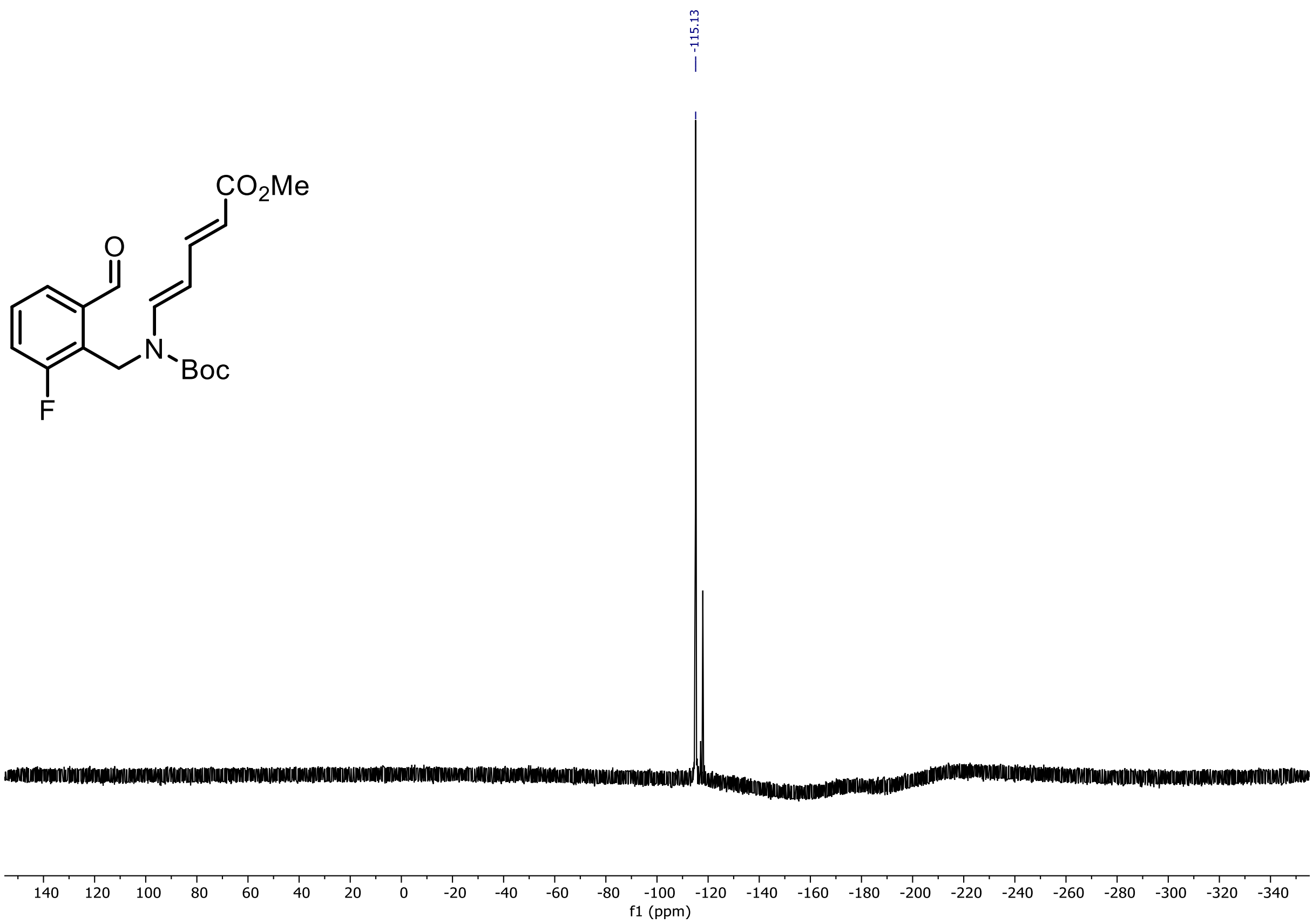

^{13}C NMR (151 MHz, $CDCl_3$) of compound **7c**

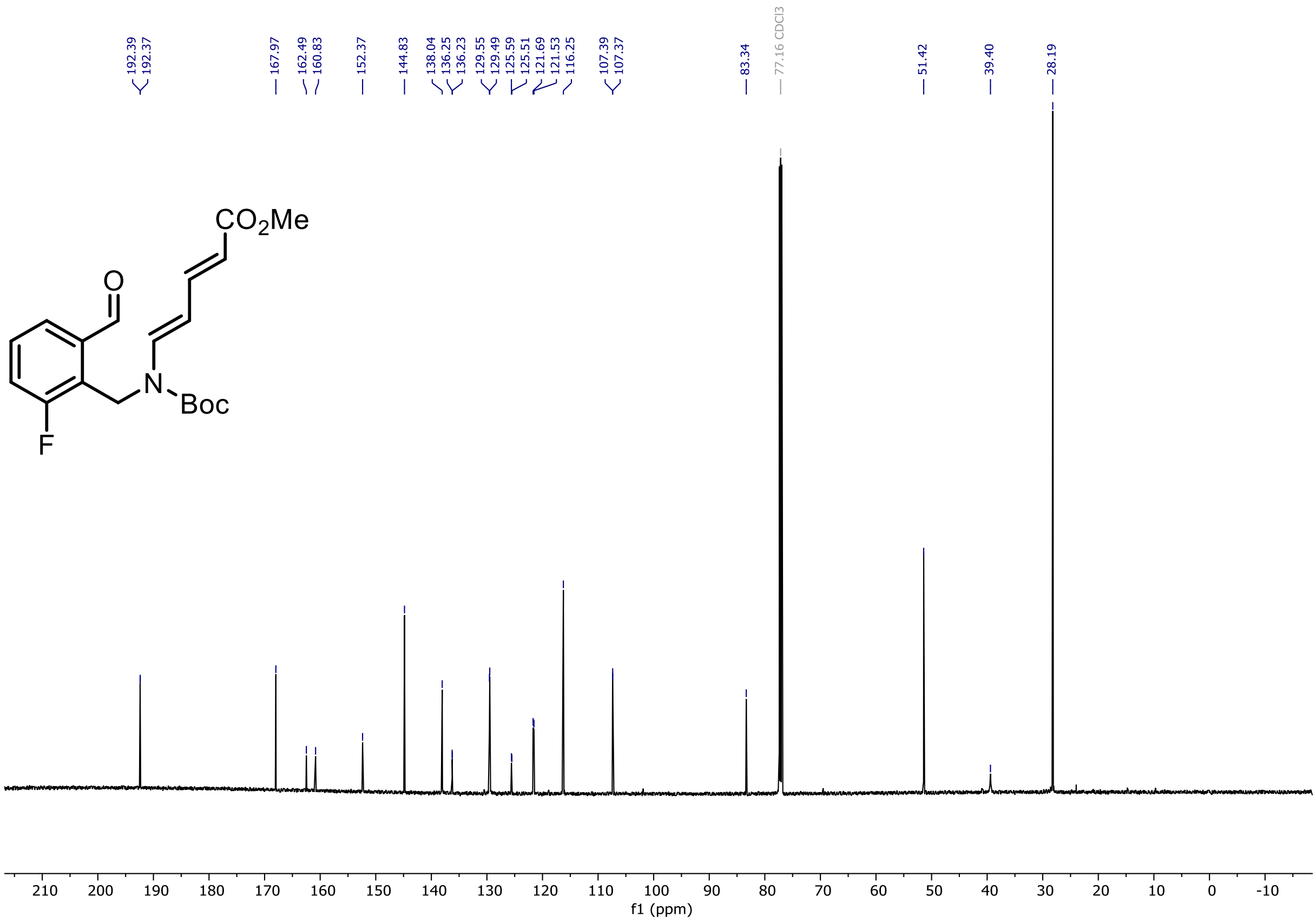

^{1}H NMR (600 MHz, $CDCl_3$; top) and ^{19}F NMR (585 MHz, $CDCl_3$; bottom) of compound **7d**

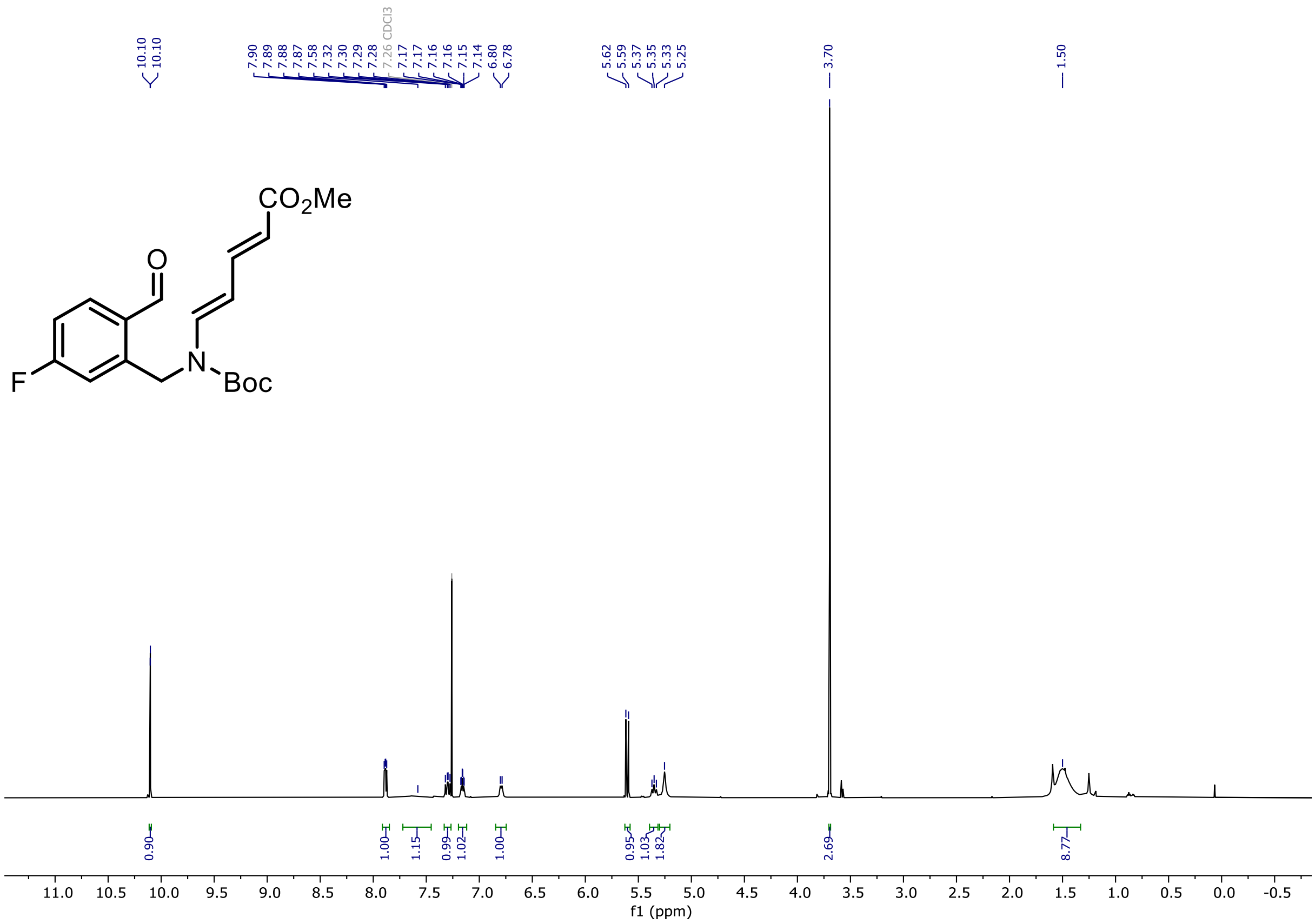

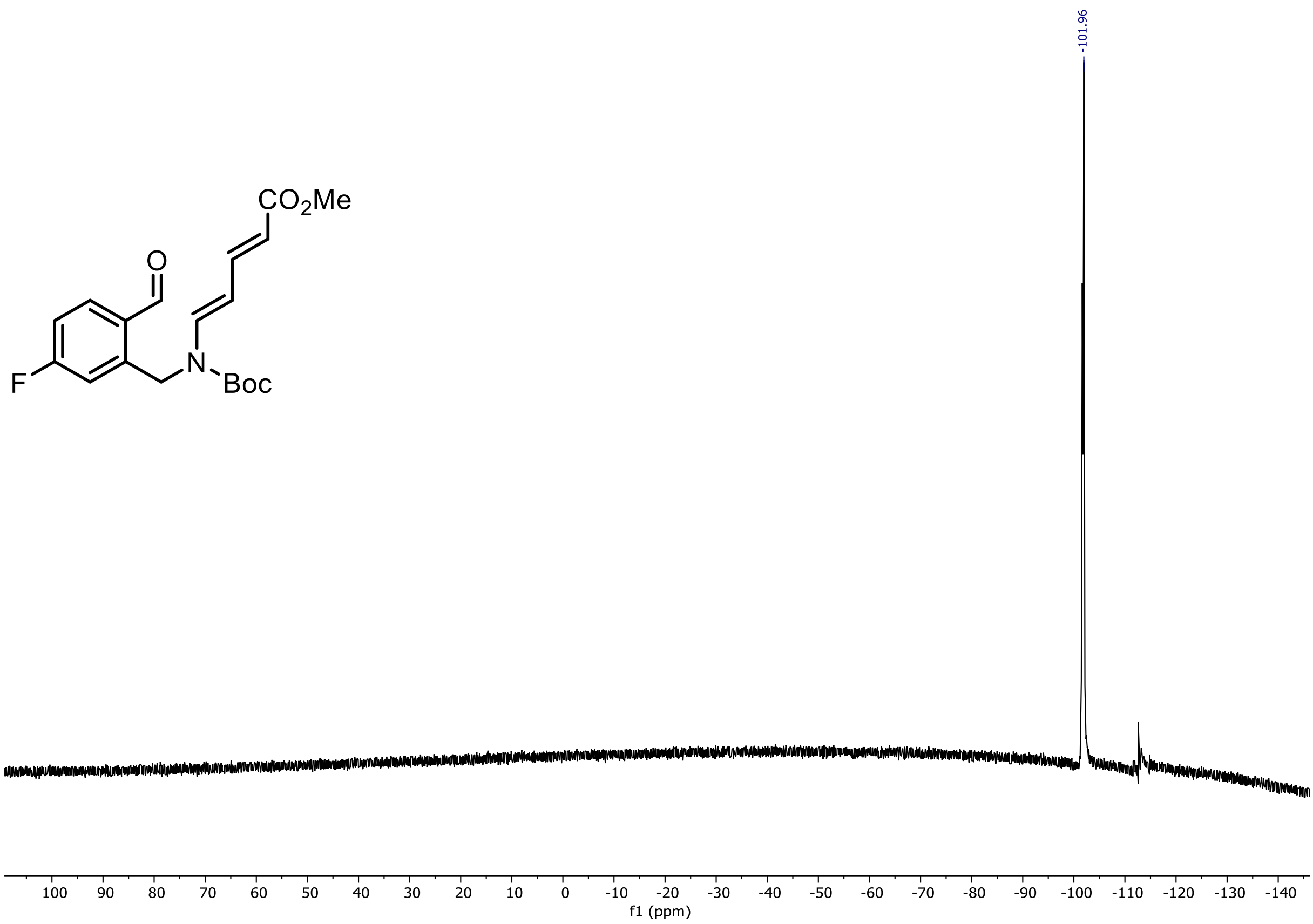

^{13}C NMR (151 MHz, $CDCl_3$) of compound **7d**

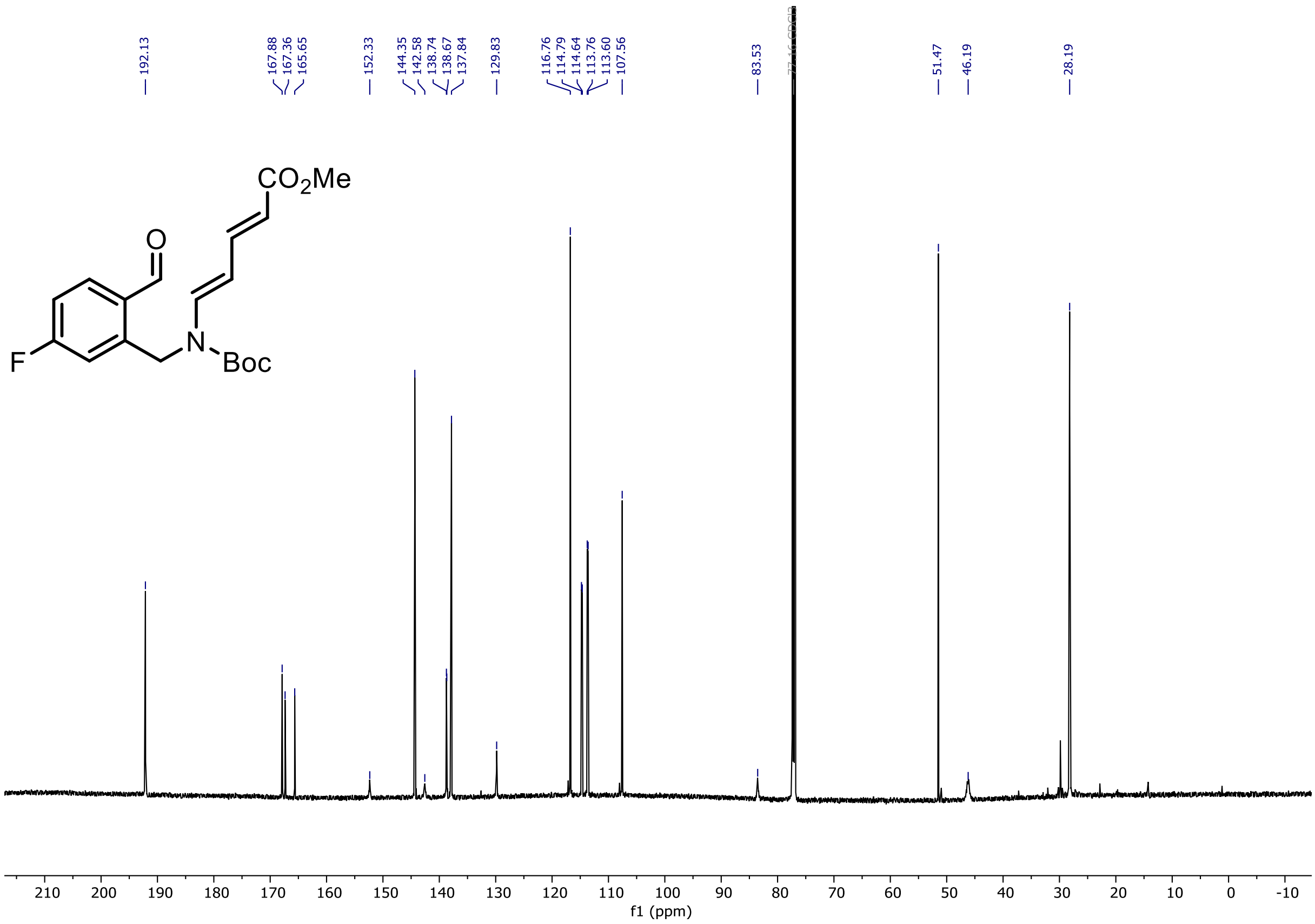

^{1}H NMR (600 MHz, $CDCl_3$; top) and ^{19}F NMR (585 MHz, $CDCl_3$; bottom) of compound **7e**

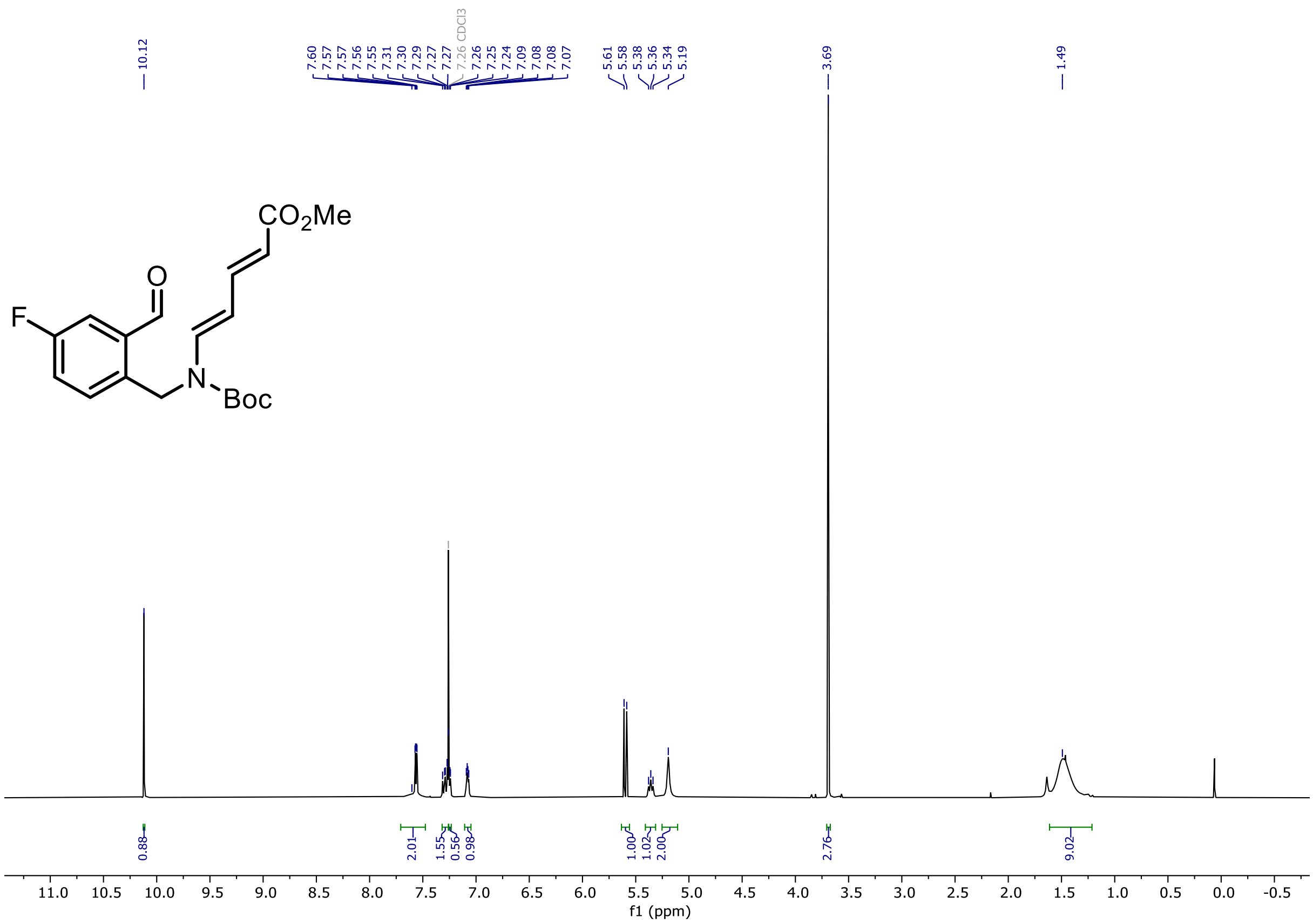

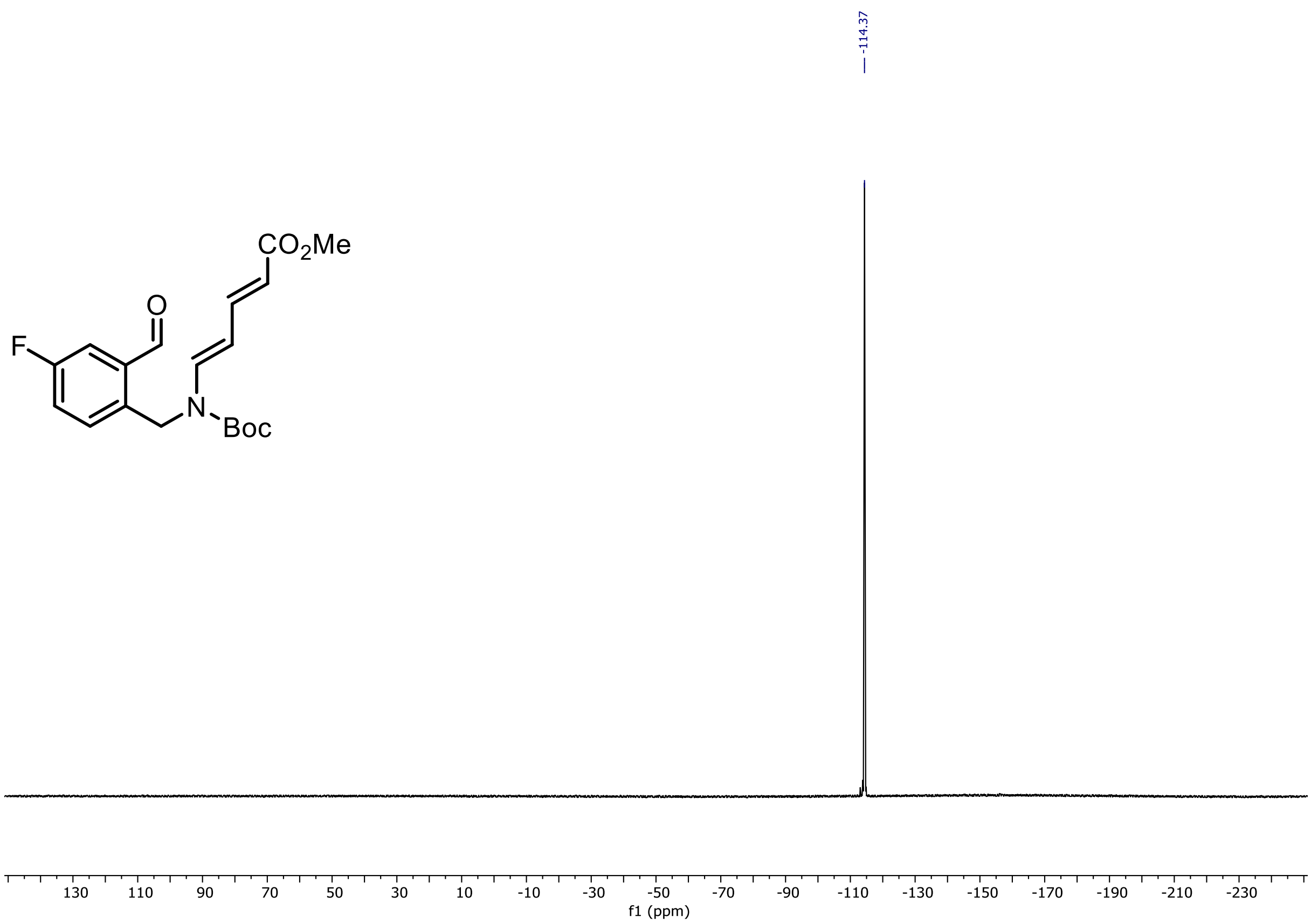

^{13}C NMR (151 MHz, $CDCl_3$) of compound **7e**

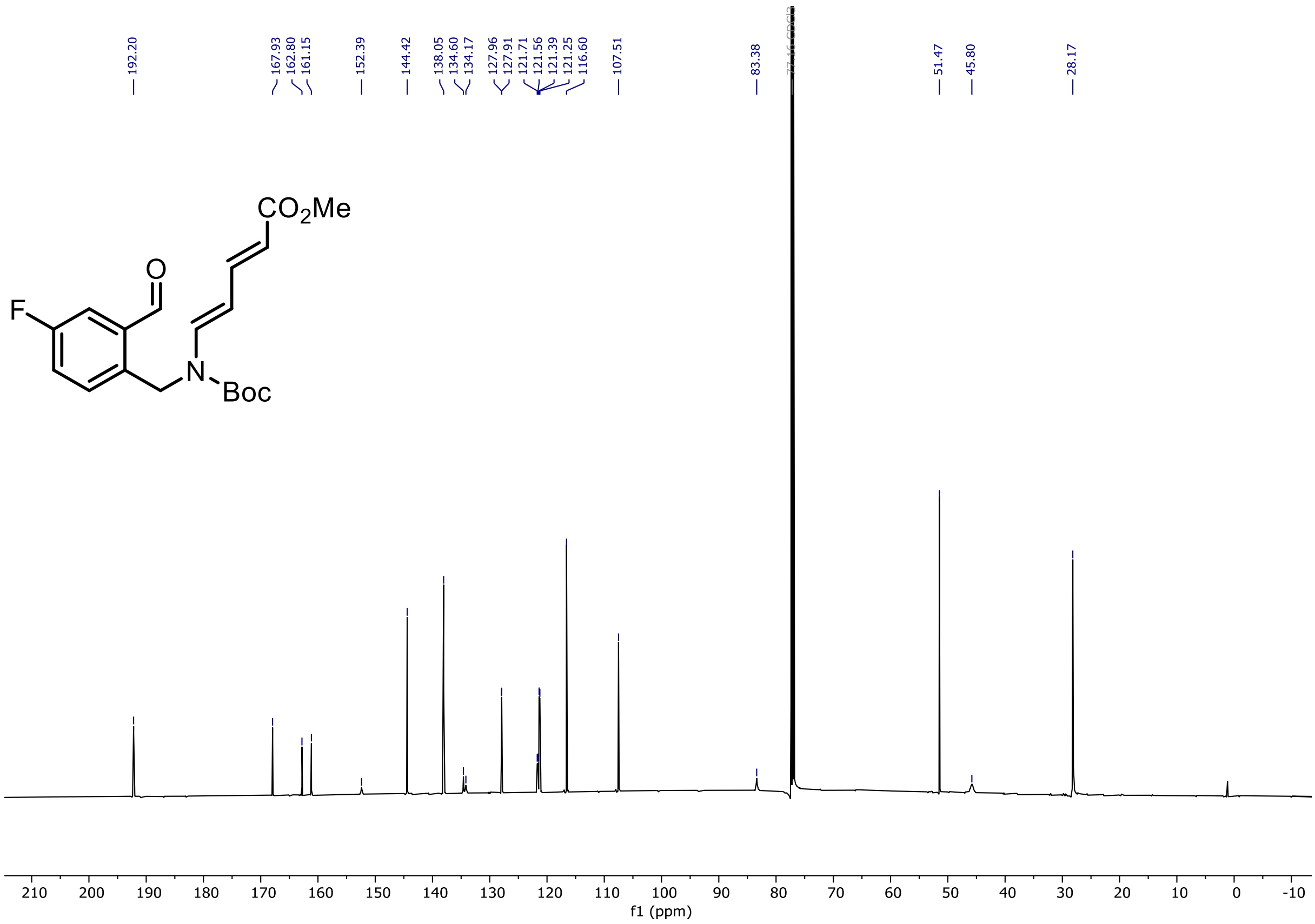

^{1}H NMR (600 MHz, $CDCl_3$; top) and ^{19}F NMR (585 MHz, $CDCl_3$; bottom) of compound **7f**

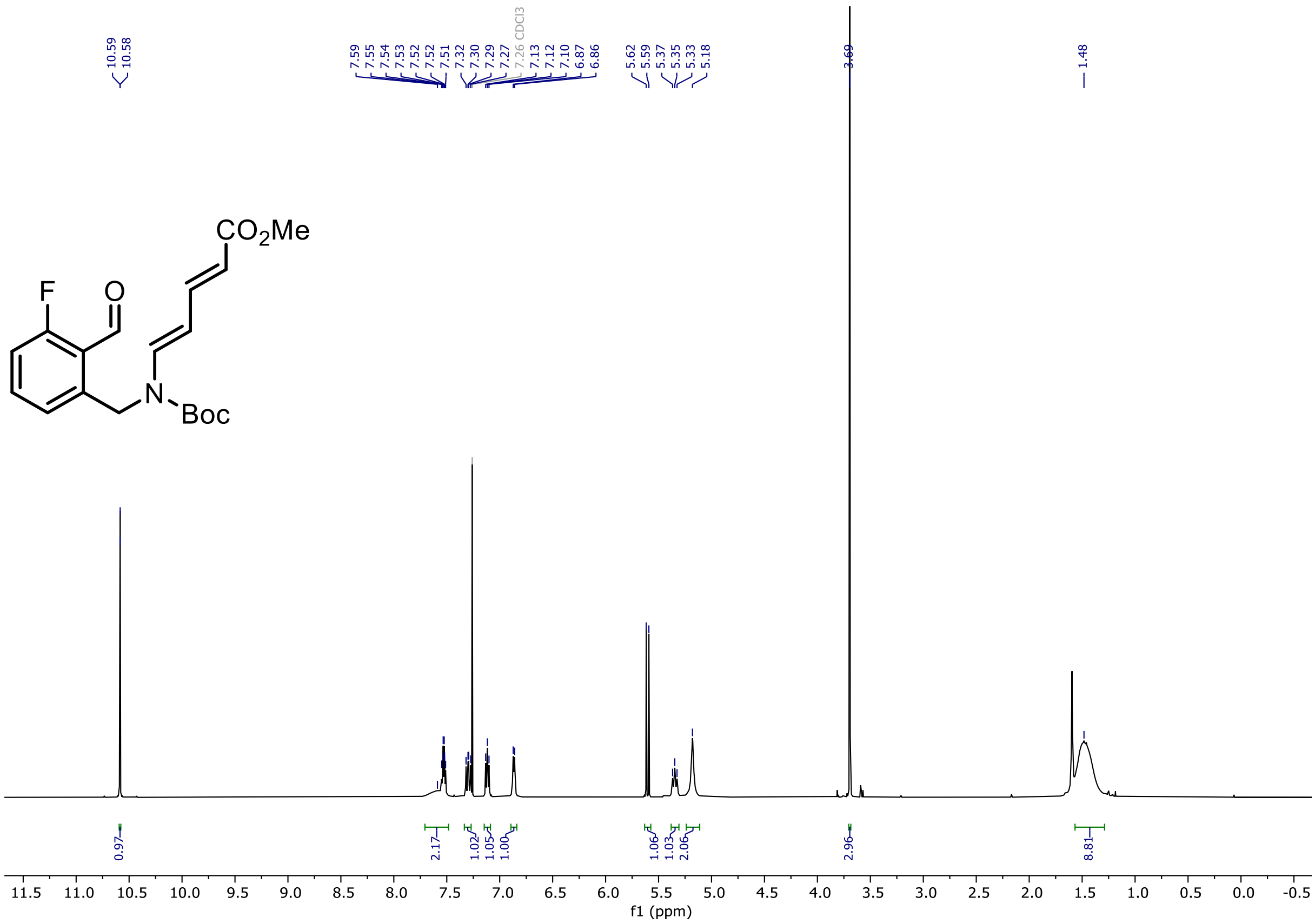

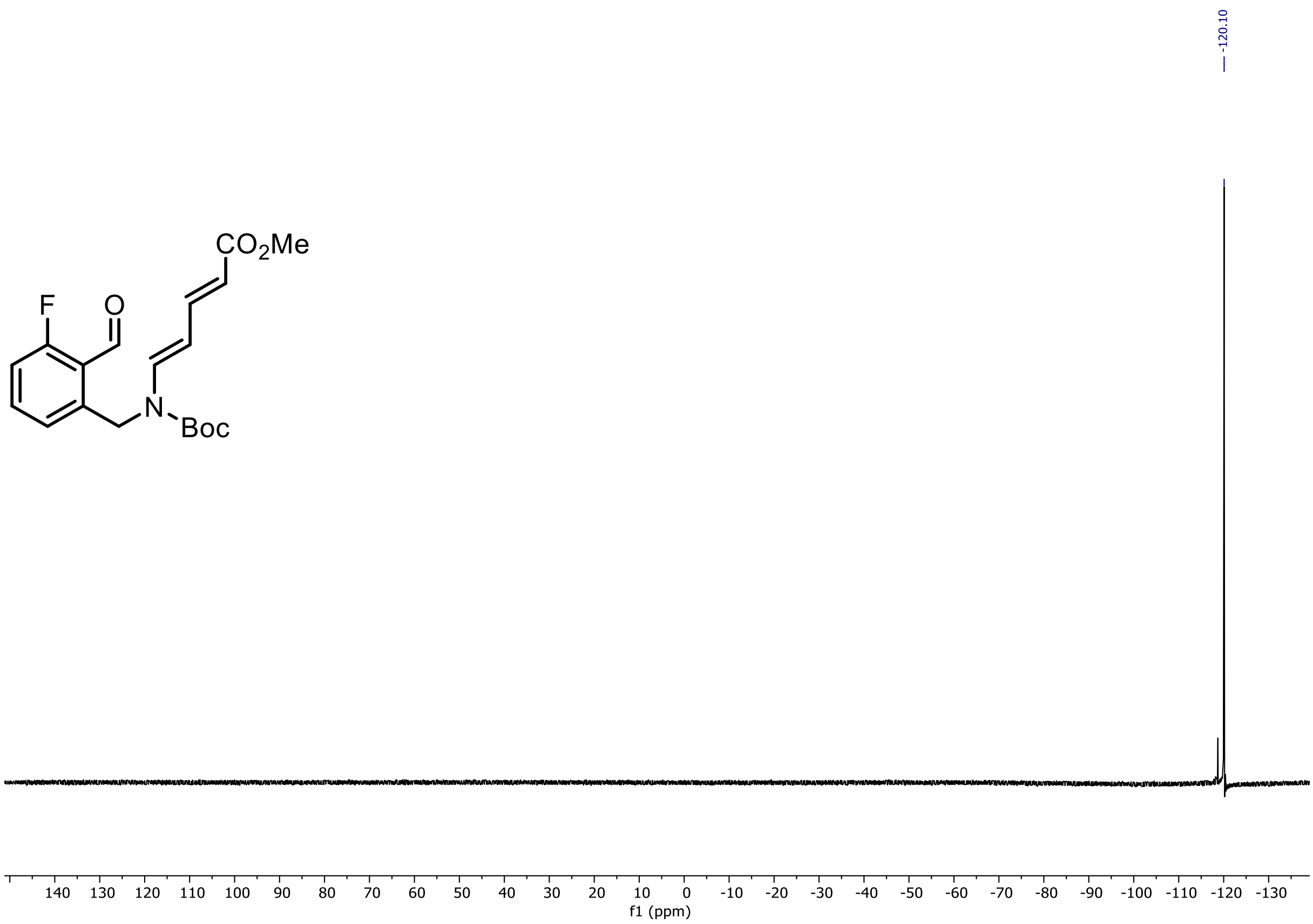

^{13}C NMR (151 MHz, $CDCl_3$) of compound **7f**

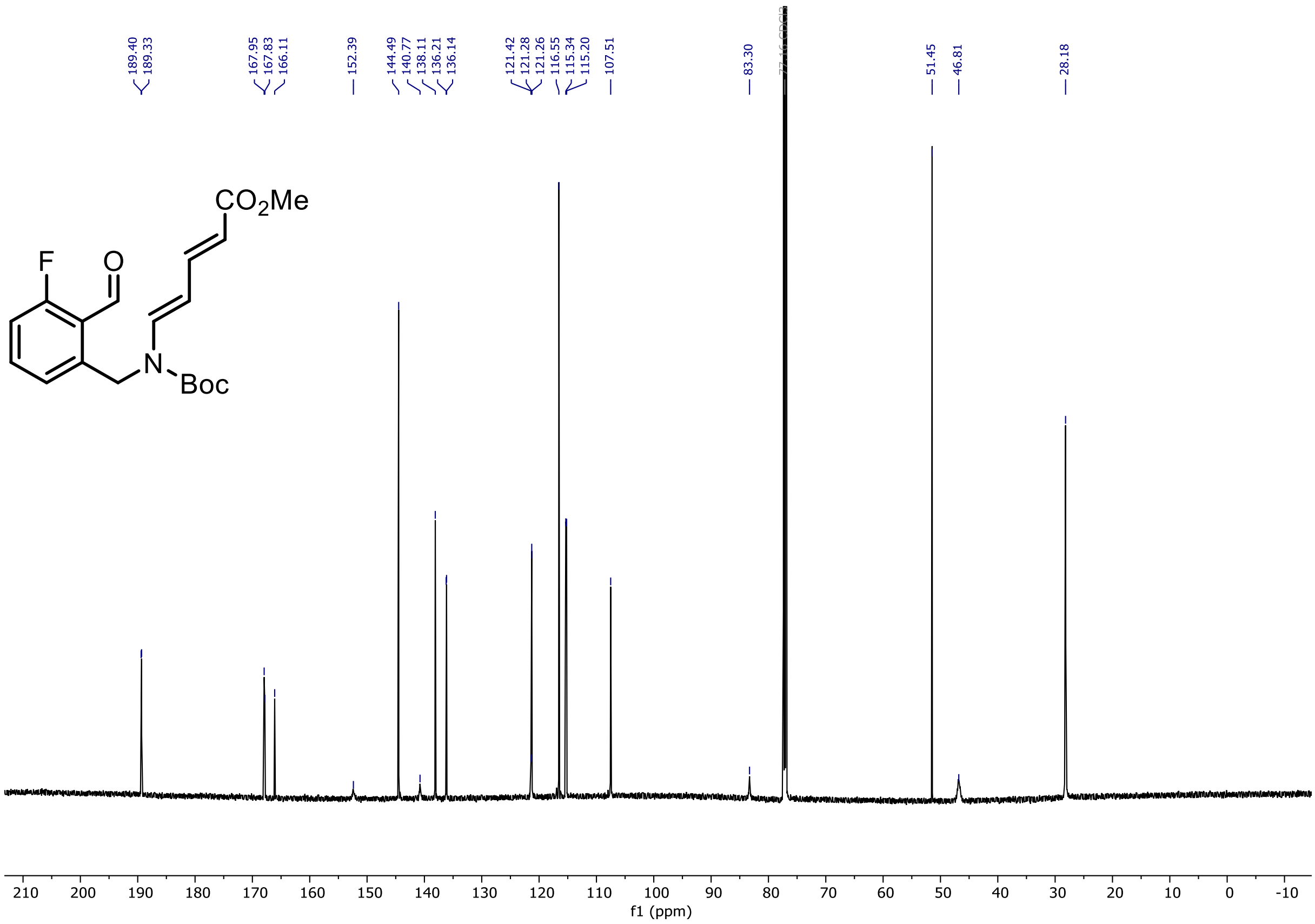

^{1}H NMR (600 MHz, $CDCl_3$; top) and ^{13}C NMR (151 MHz, $CDCl_3$; bottom) of compound **7g**

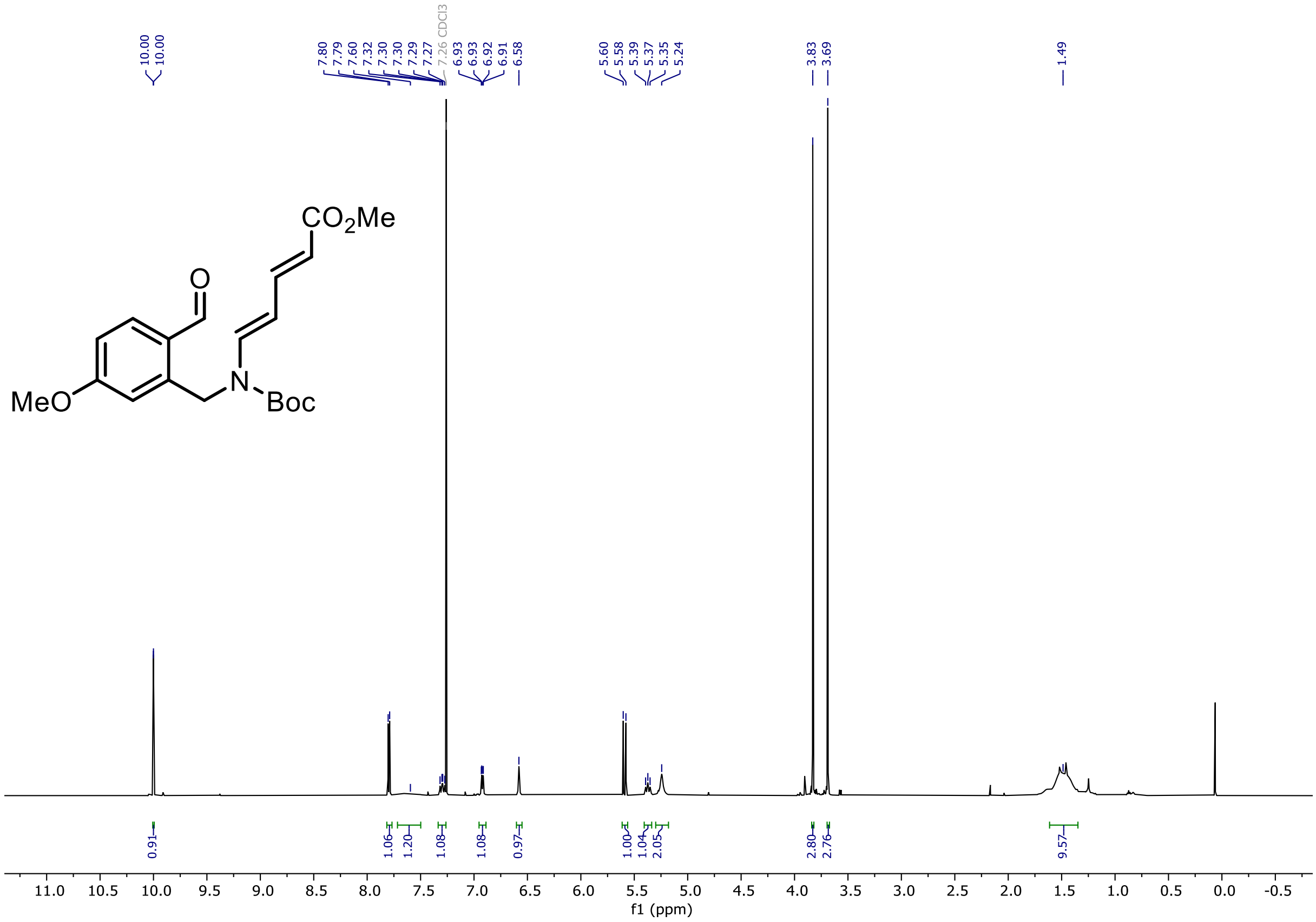

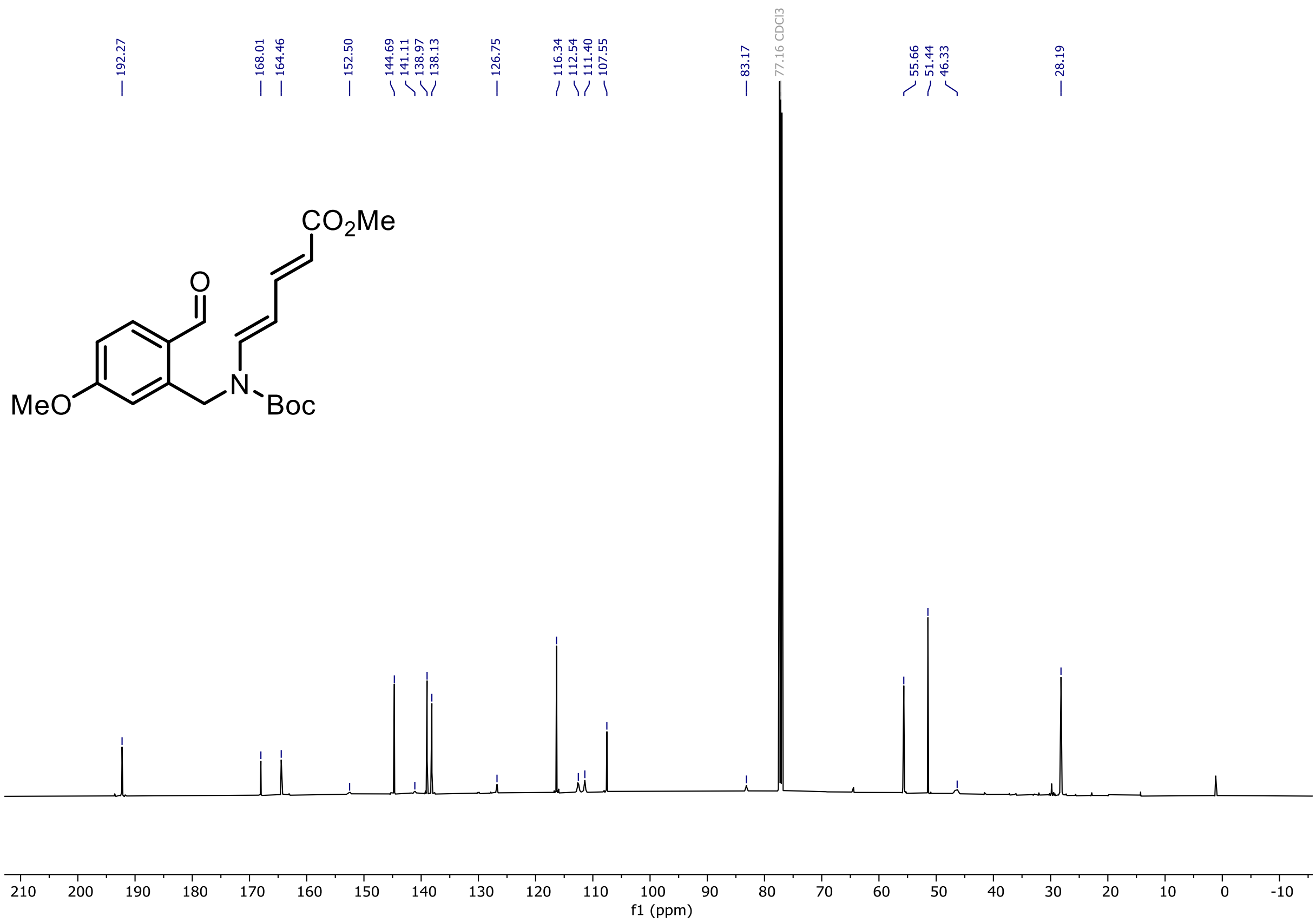

^{1}H NMR (600 MHz, $CDCl_3$; top) and ^{13}C NMR (151 MHz, $CDCl_3$; bottom) of compound **7h**

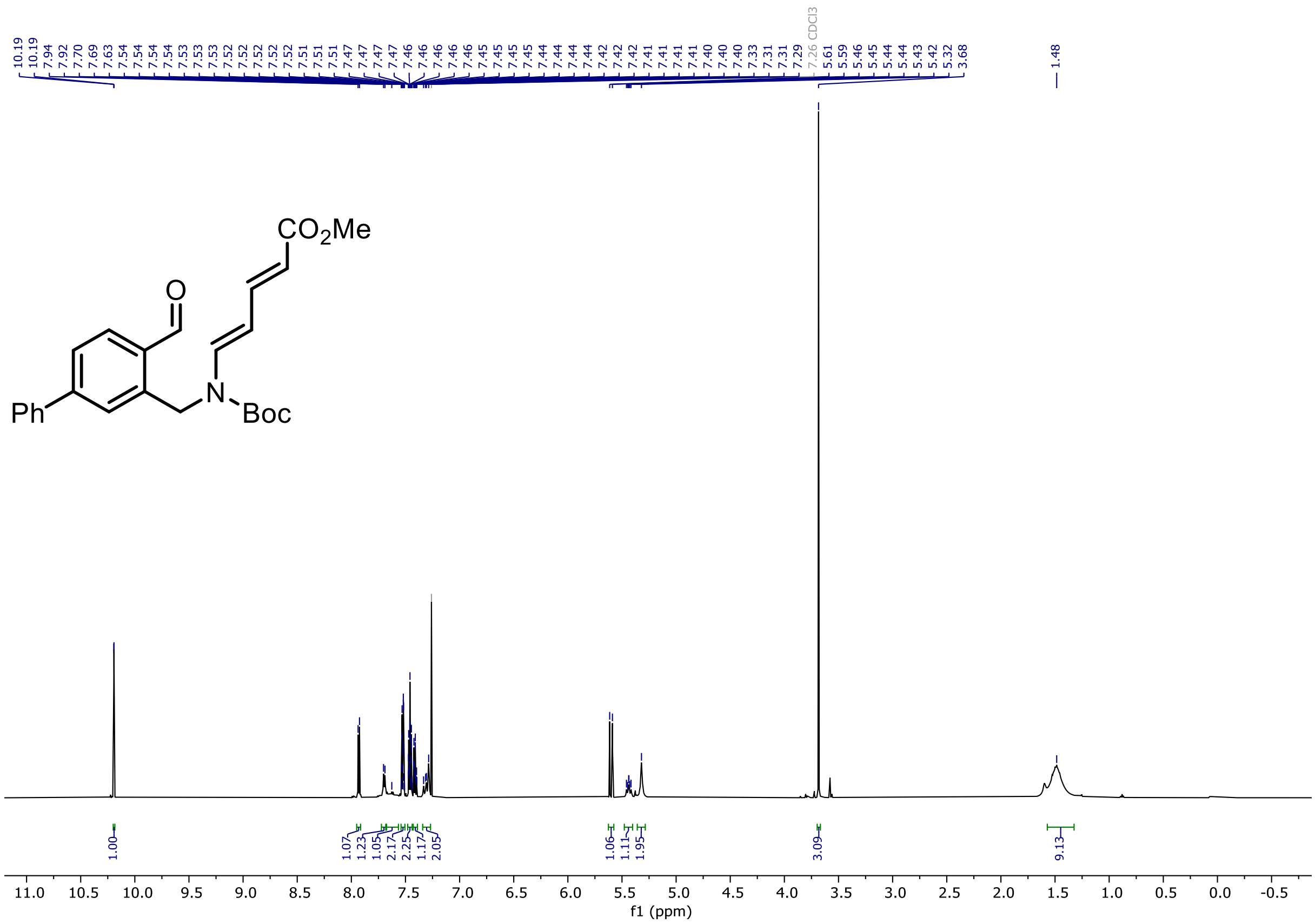

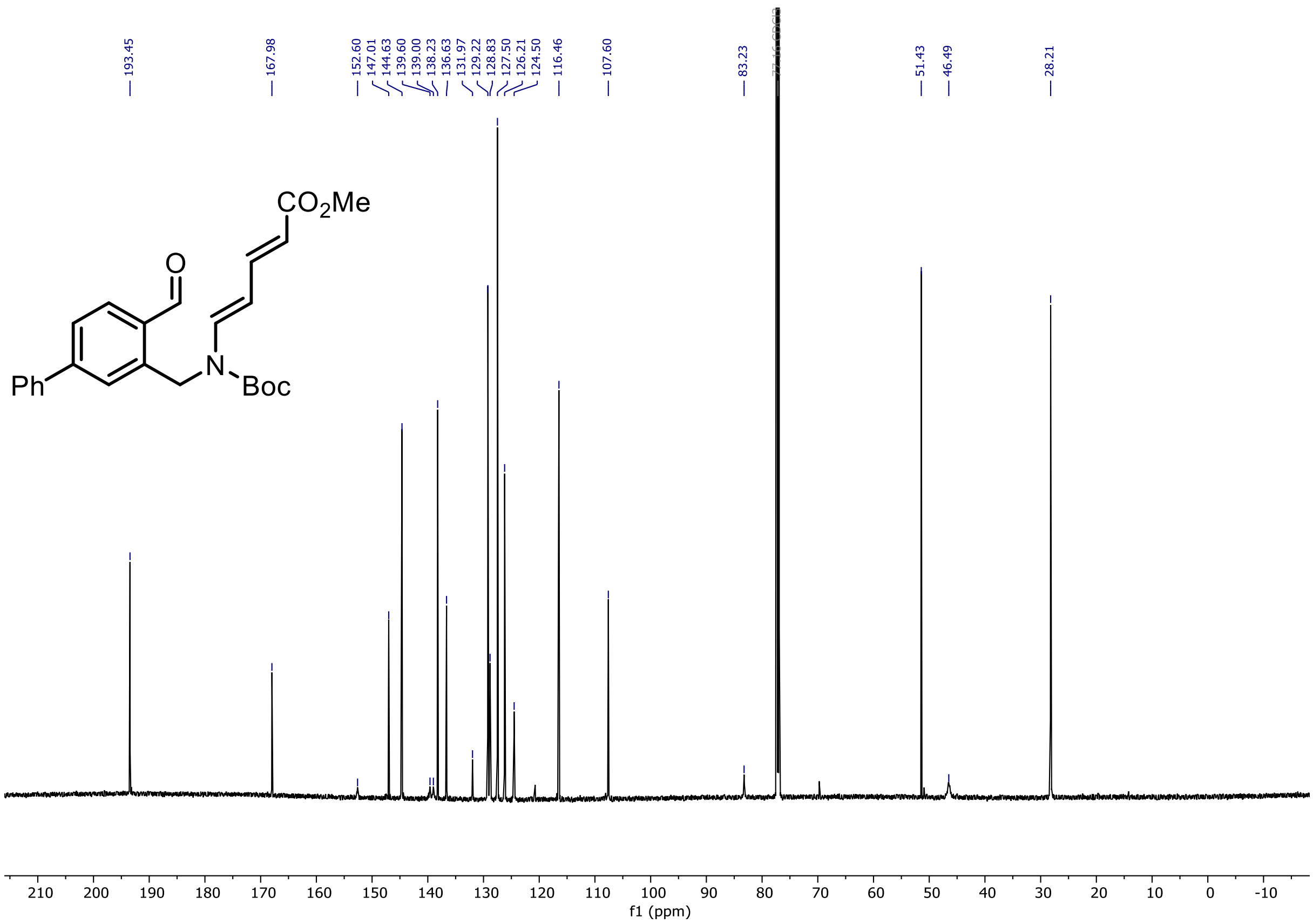

^1H NMR (400 MHz, $CDCl_3$; top) and ^{13}C NMR (101 MHz, $CDCl_3$; bottom) of compound **7i**

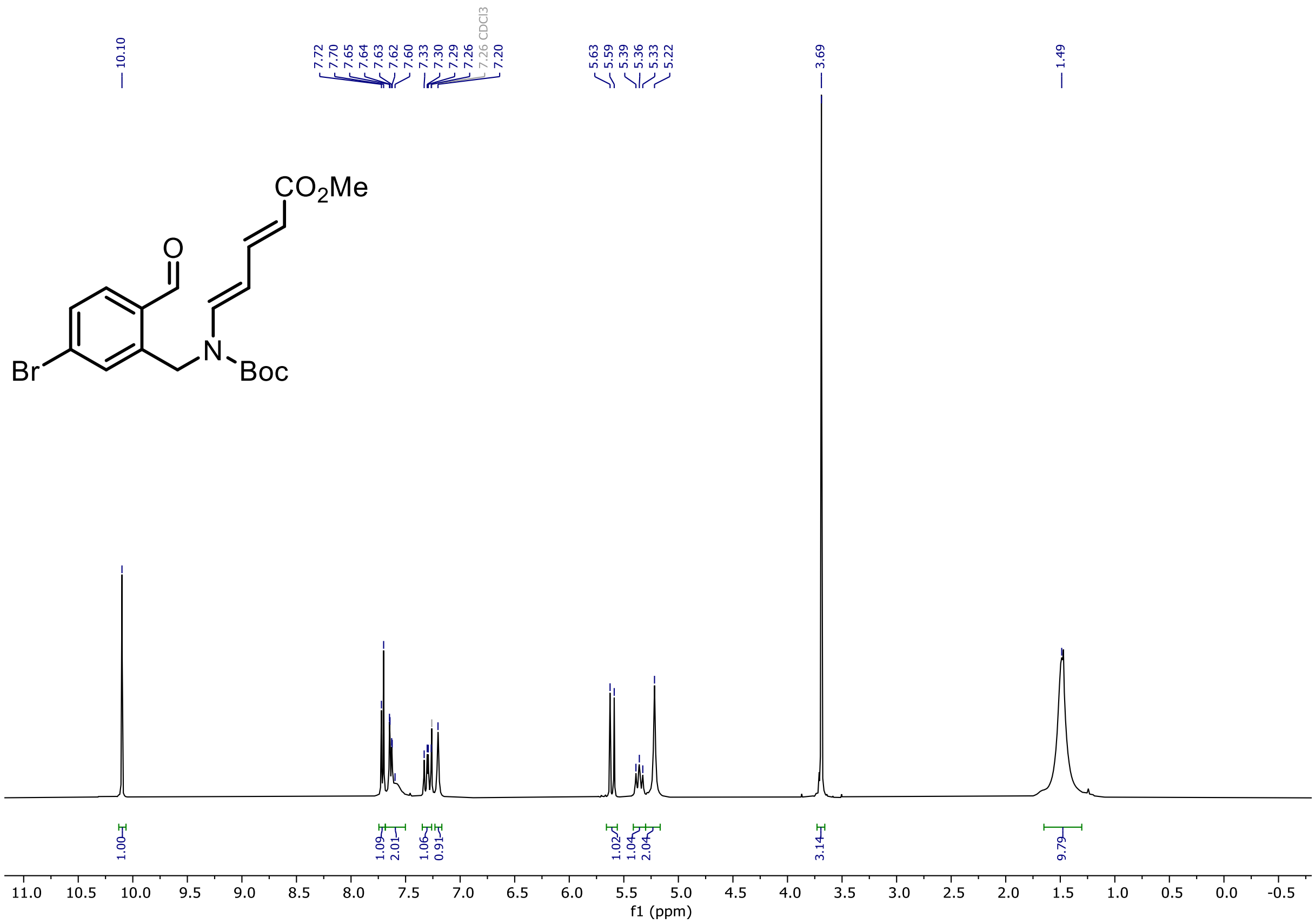

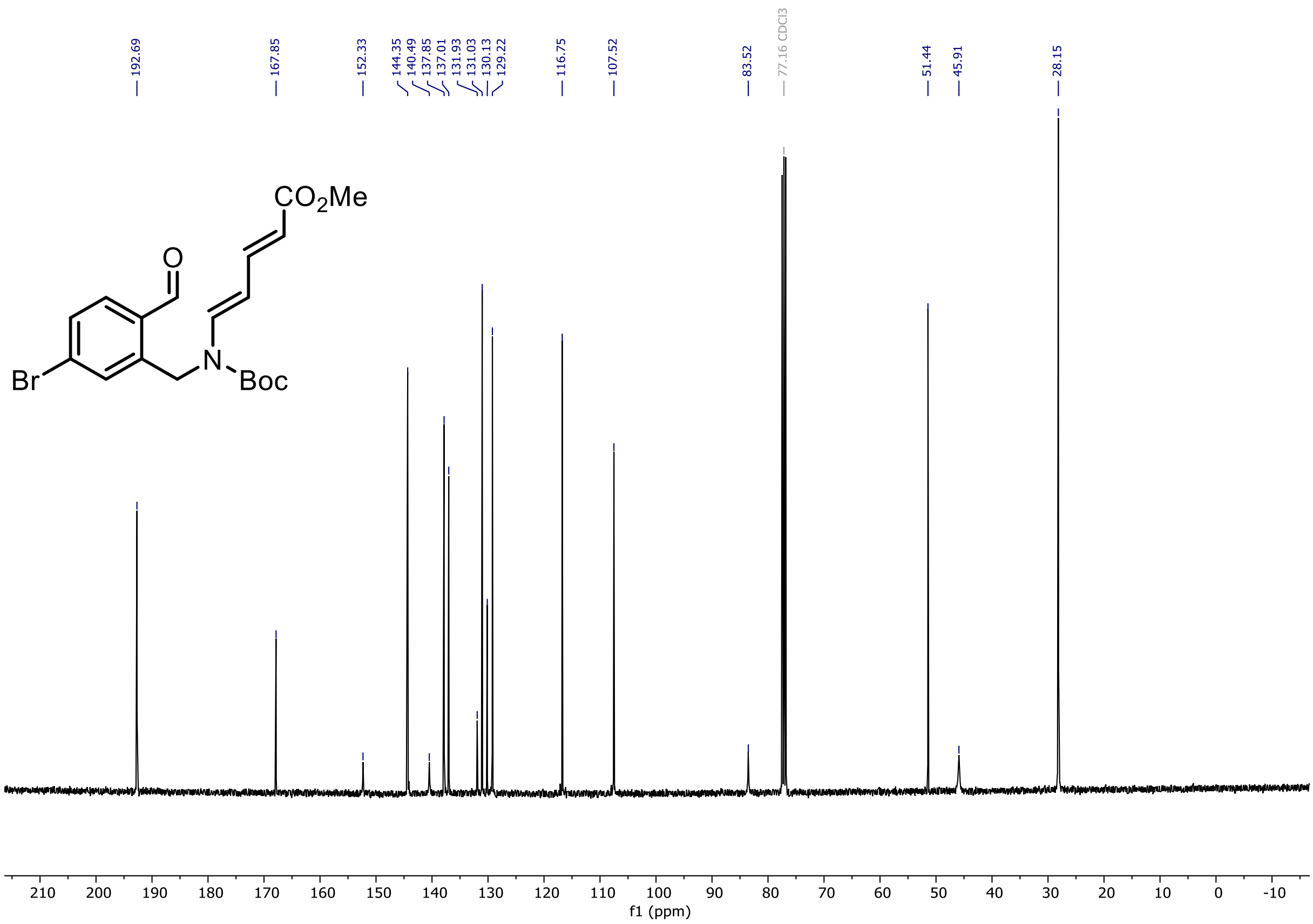

^{1}H NMR (400 MHz, $CDCl_3$; top) and ^{13}C NMR (101 MHz, $CDCl_3$; bottom) of compound **7j**

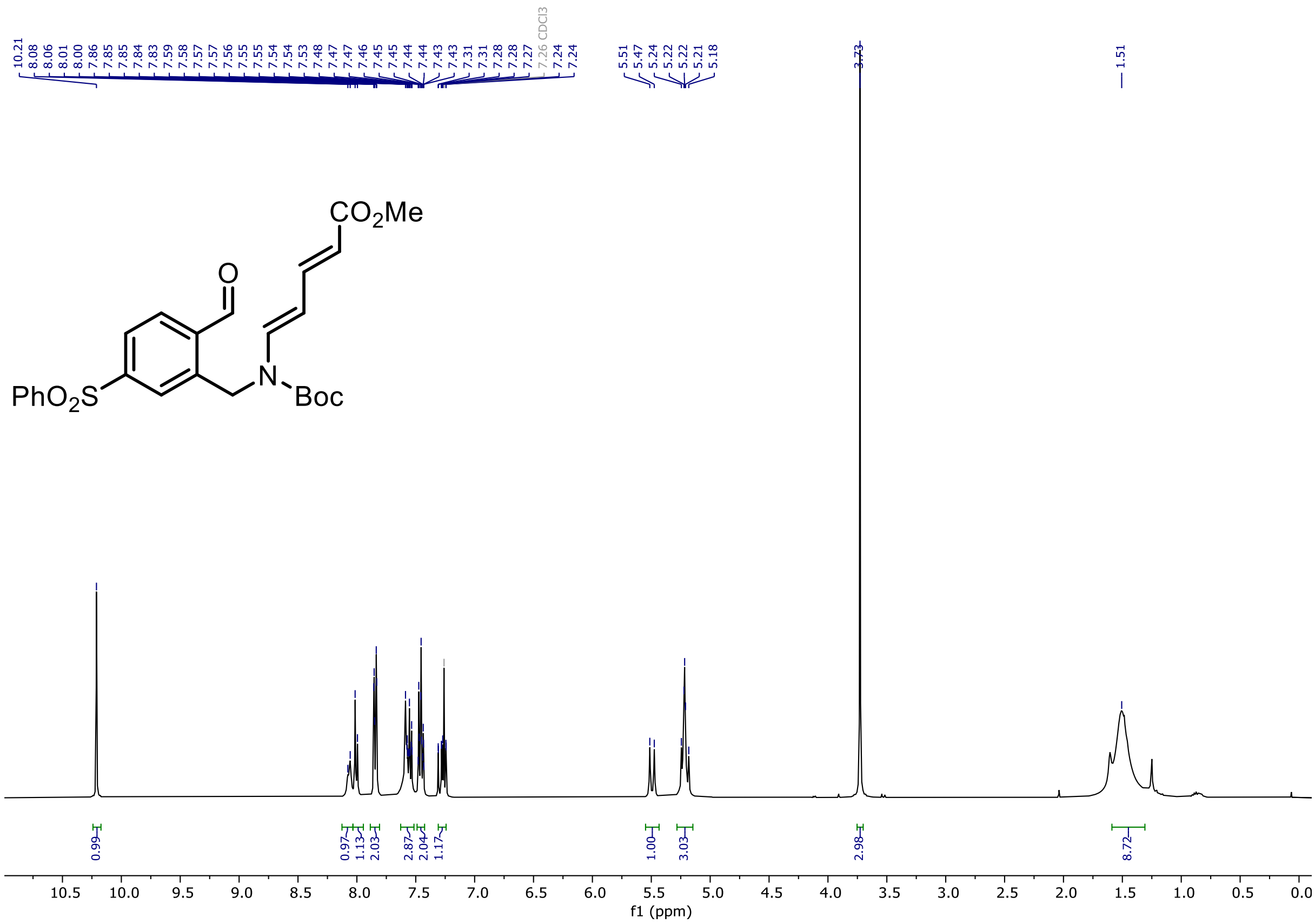

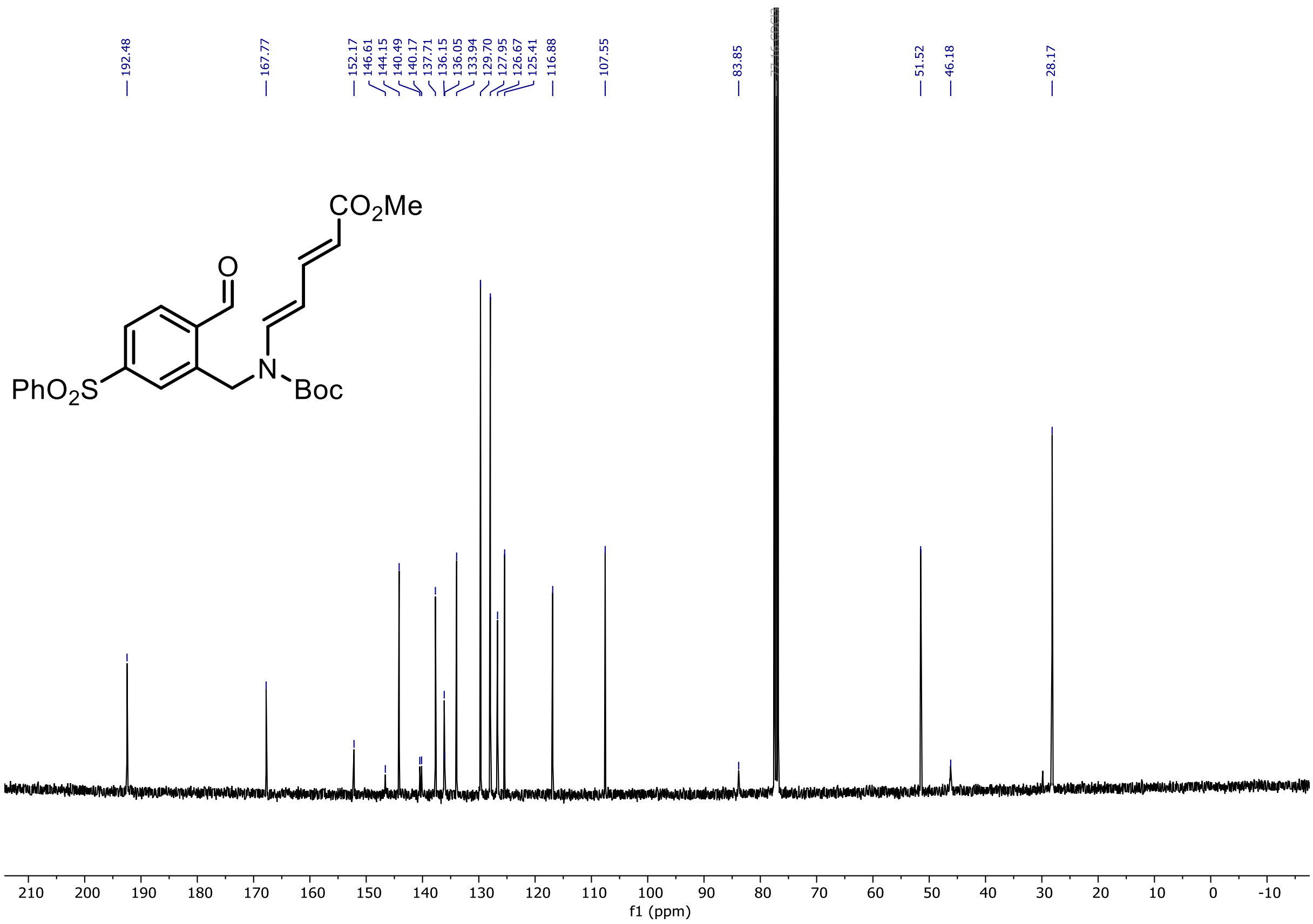

^{1}H NMR (600 MHz, $CDCl_3$; top) and ^{13}C NMR (151 MHz, $CDCl_3$; bottom) of compound **7k**

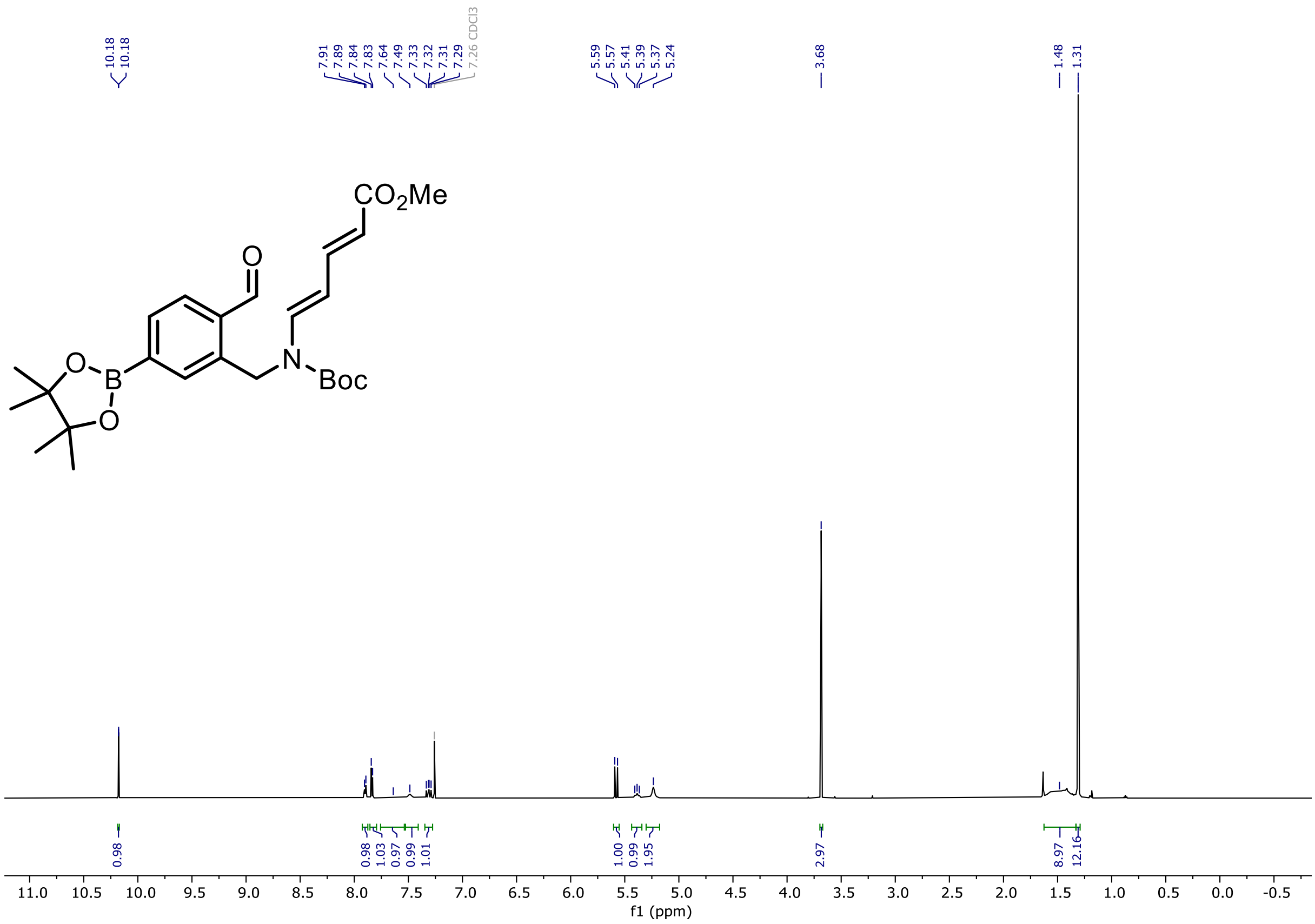

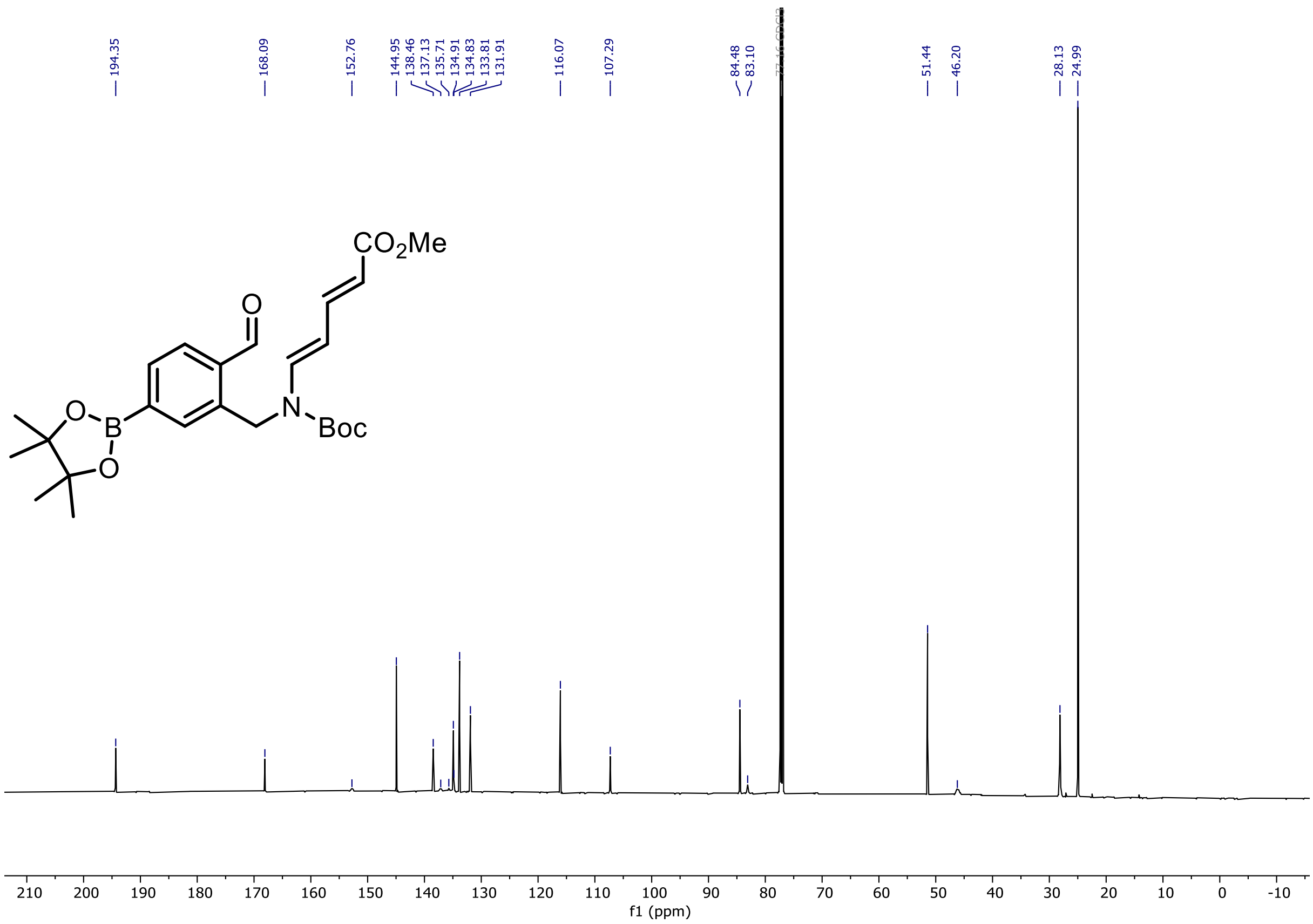

^{1}H NMR (600 MHz, $CDCl_3$; top) and ^{13}C NMR (151 MHz, $CDCl_3$; bottom) of compound **7m**

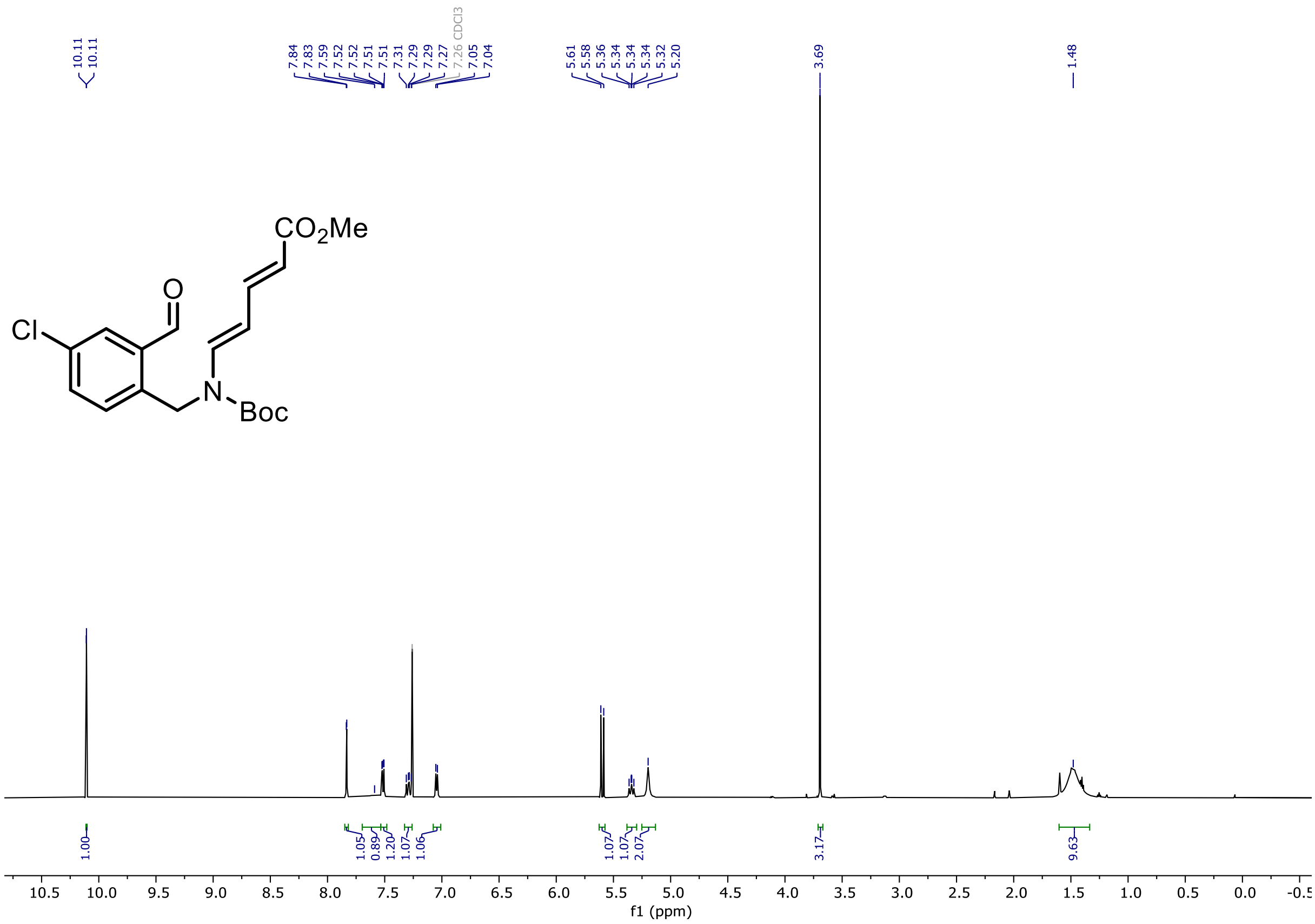

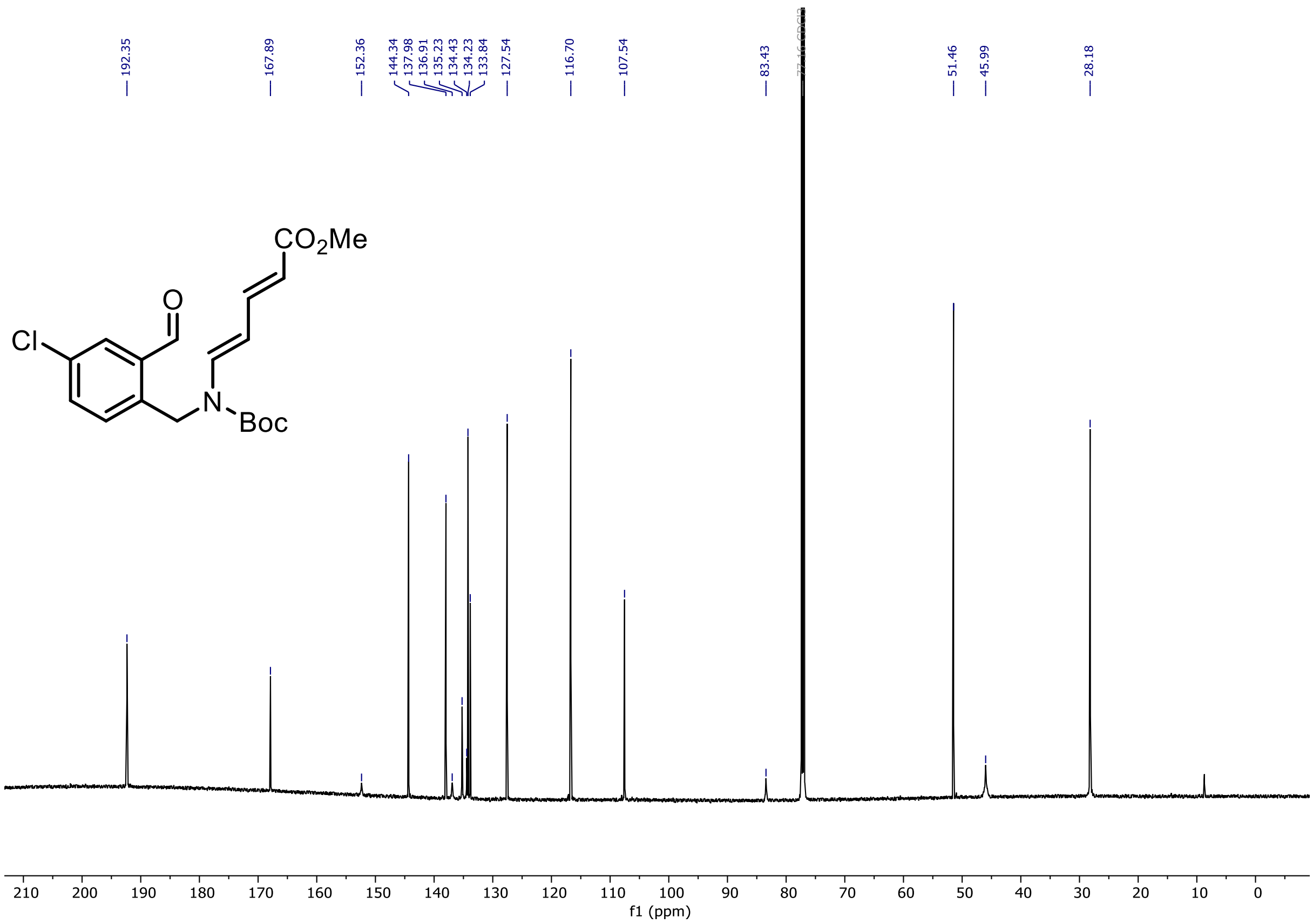

^{1}H NMR (400 MHz, $CDCl_3$; top) and ^{19}F NMR (282 MHz, $CDCl_3$; bottom) of compound **7n**

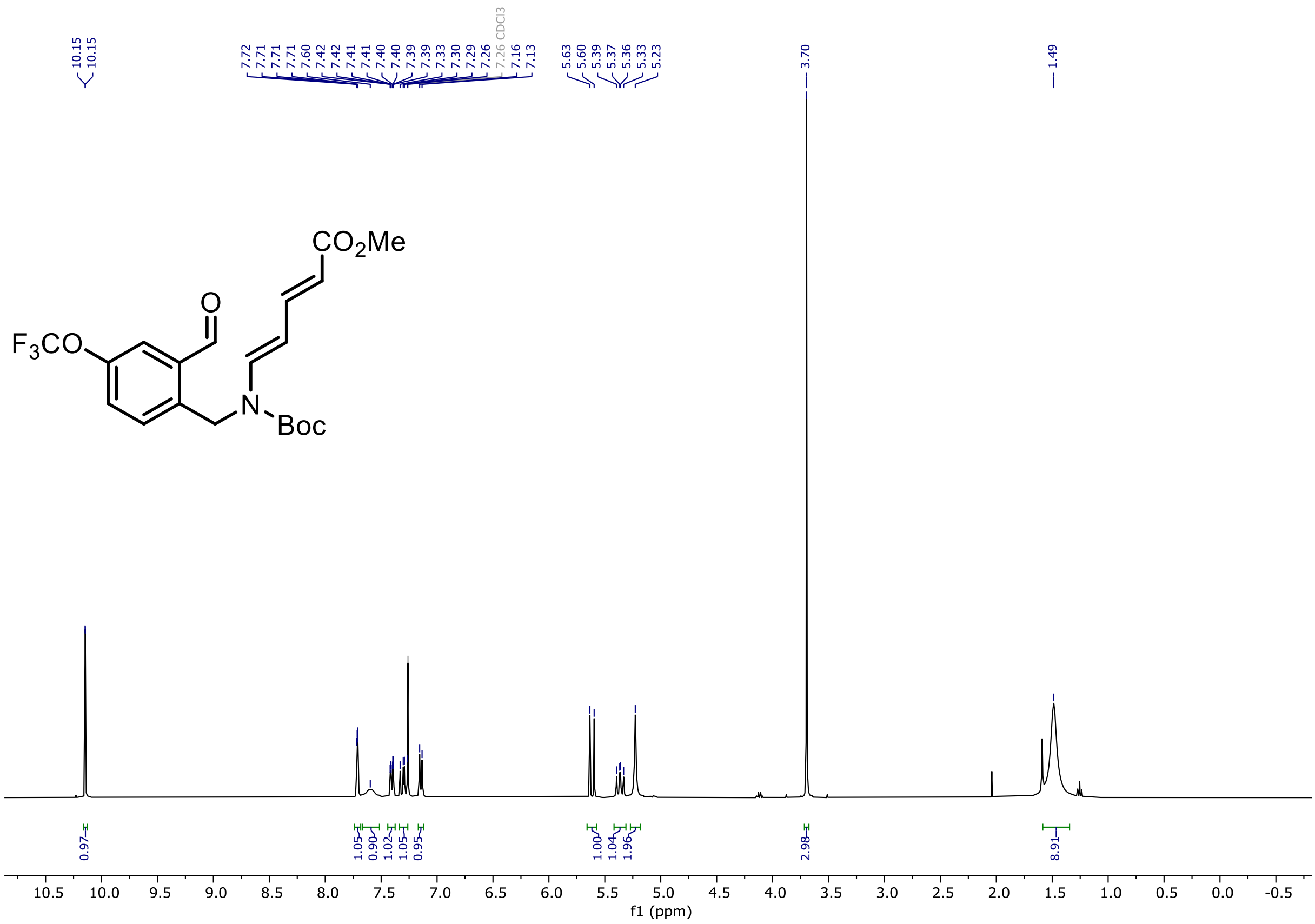

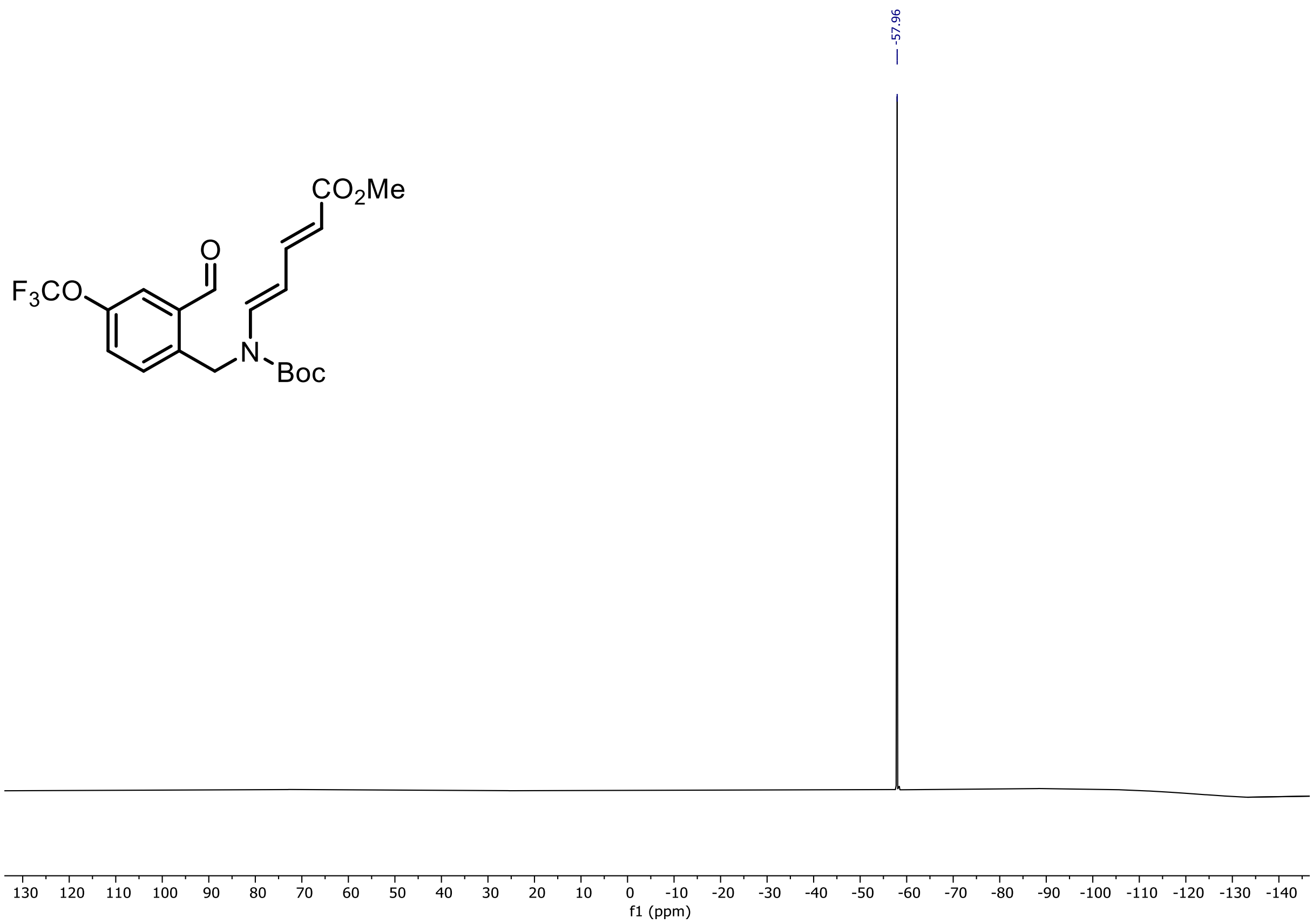

^{13}C NMR (101 MHz, $CDCl_3$) of compound **7n**

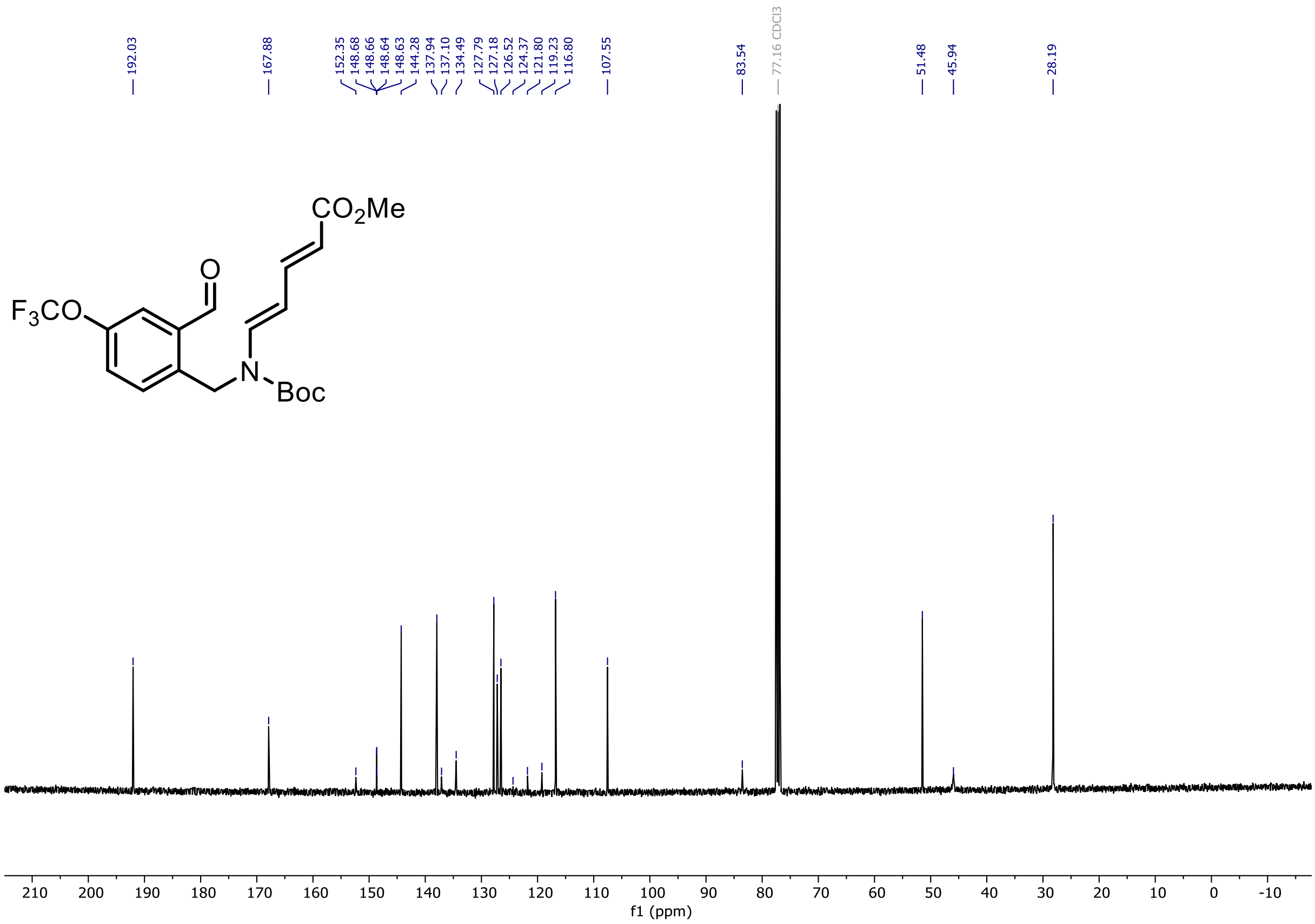

^{1}H NMR (400 MHz, $CDCl_3$; top) and ^{13}C NMR (101 MHz, $CDCl_3$; bottom) of compound **S28**

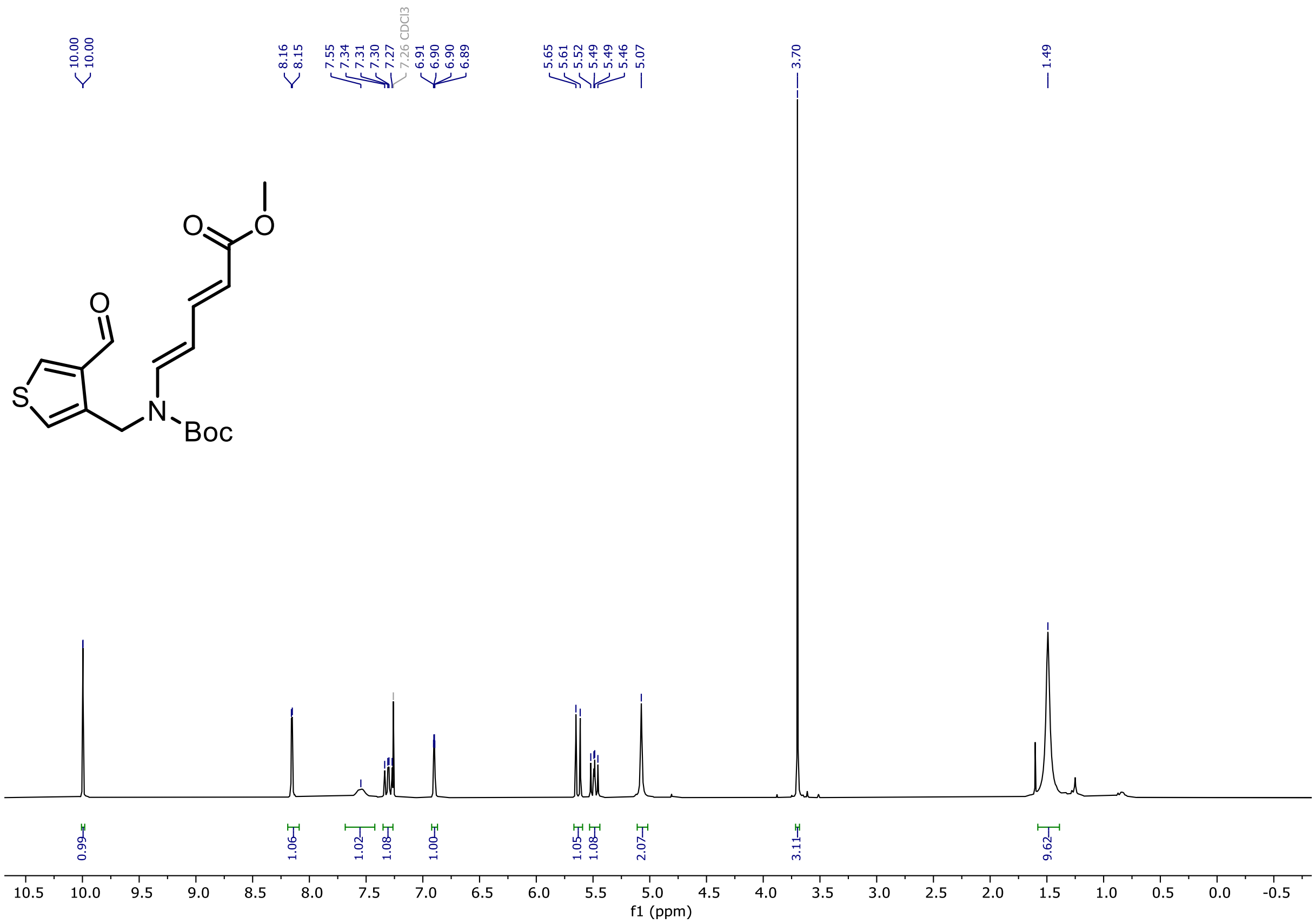

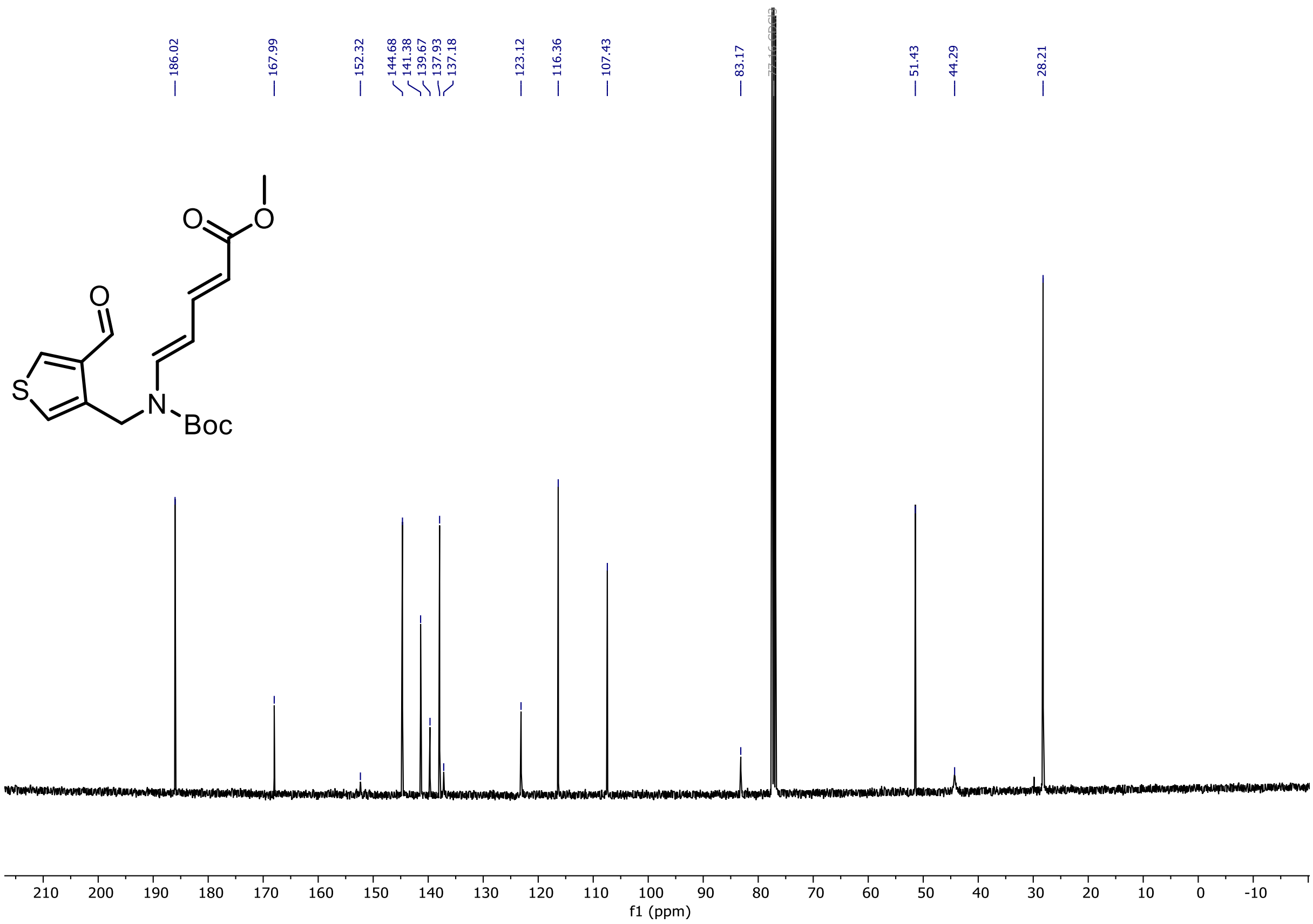

^{1}H NMR (400 MHz, $CDCl_3$; top) and ^{13}C NMR (101 MHz, $CDCl_3$; bottom) of compound **S29**

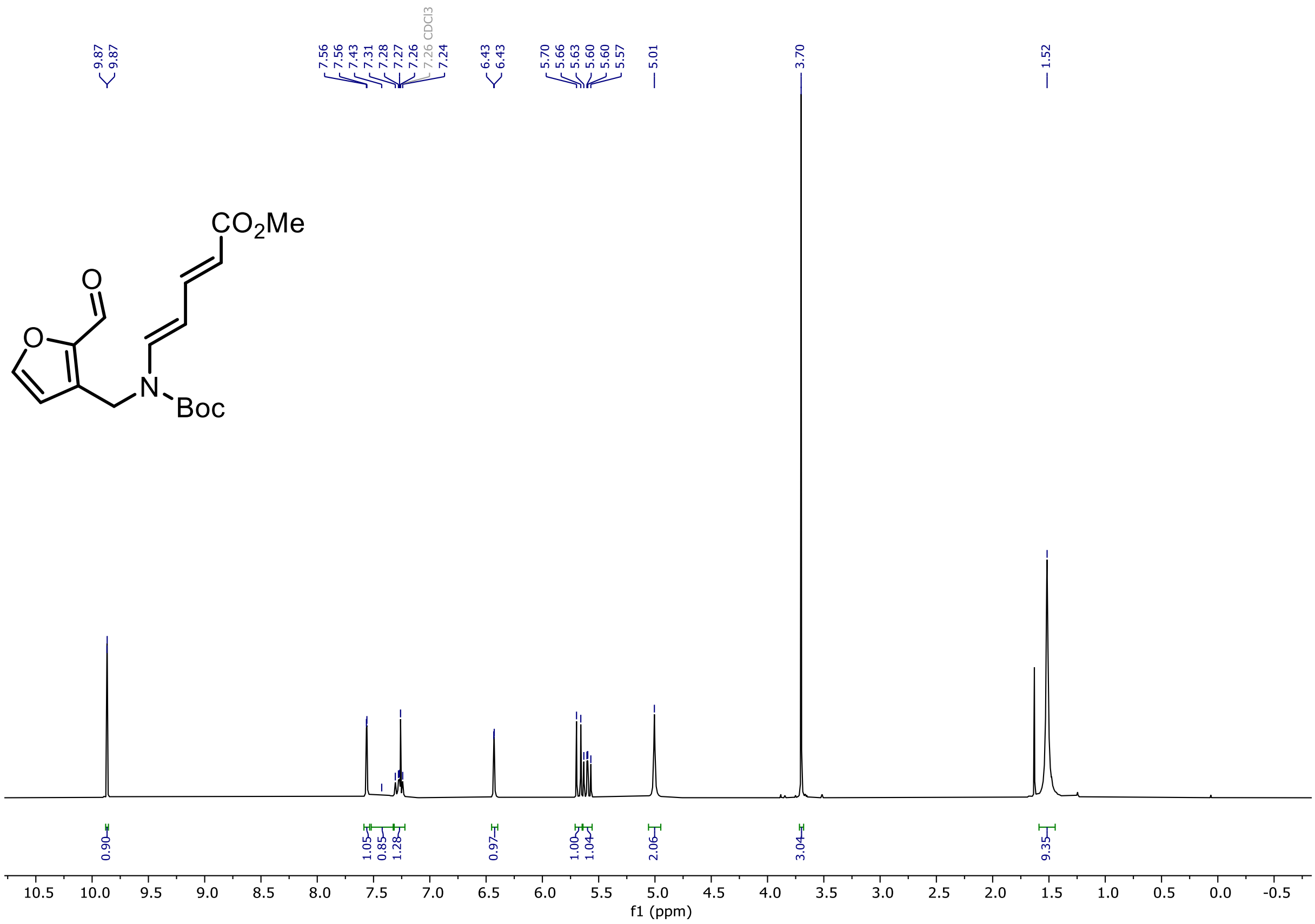

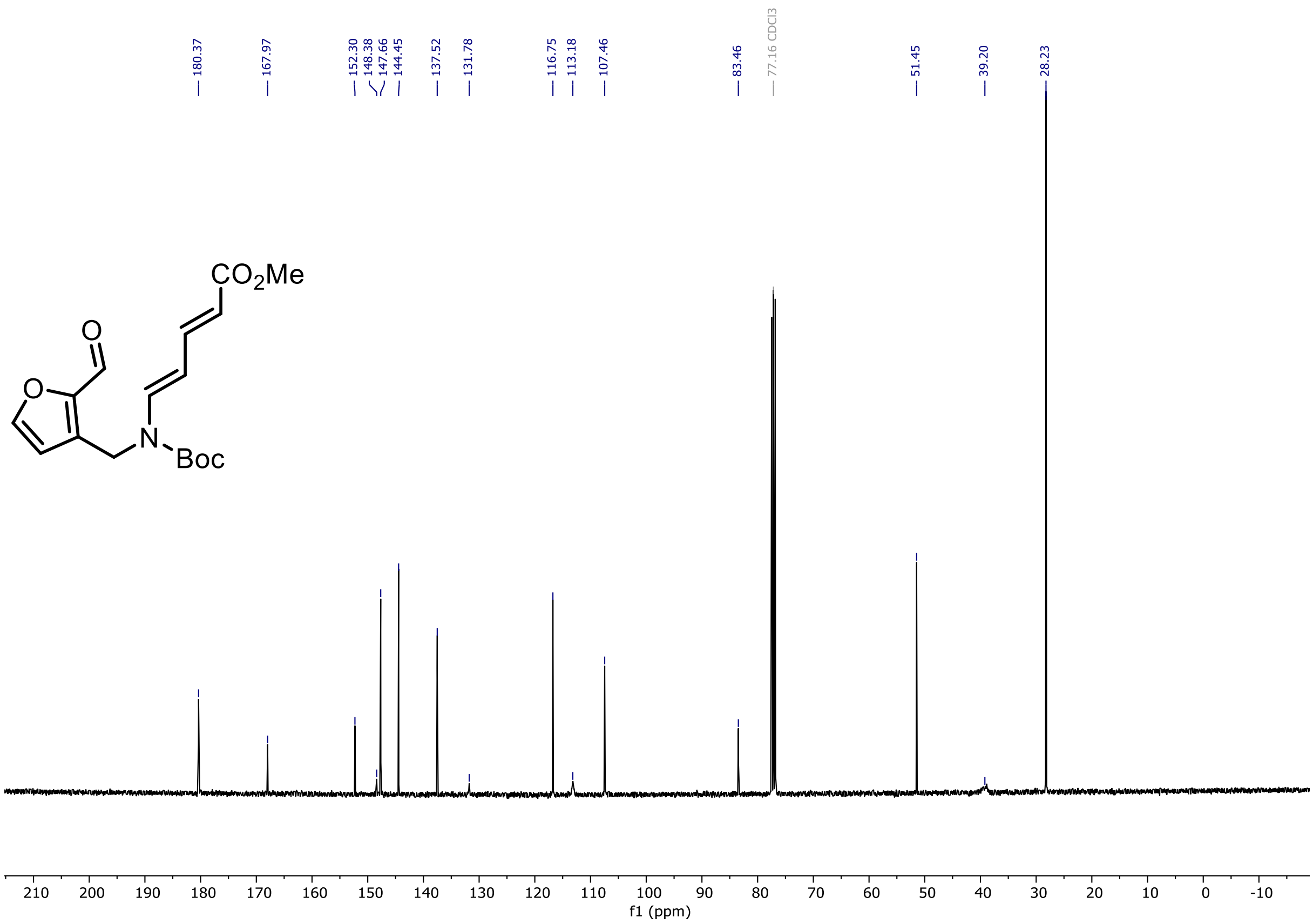

^{1}H NMR (600 MHz, $CDCl_3$; top) and ^{13}C NMR (151 MHz, $CDCl_3$; bottom) of compound **S30**

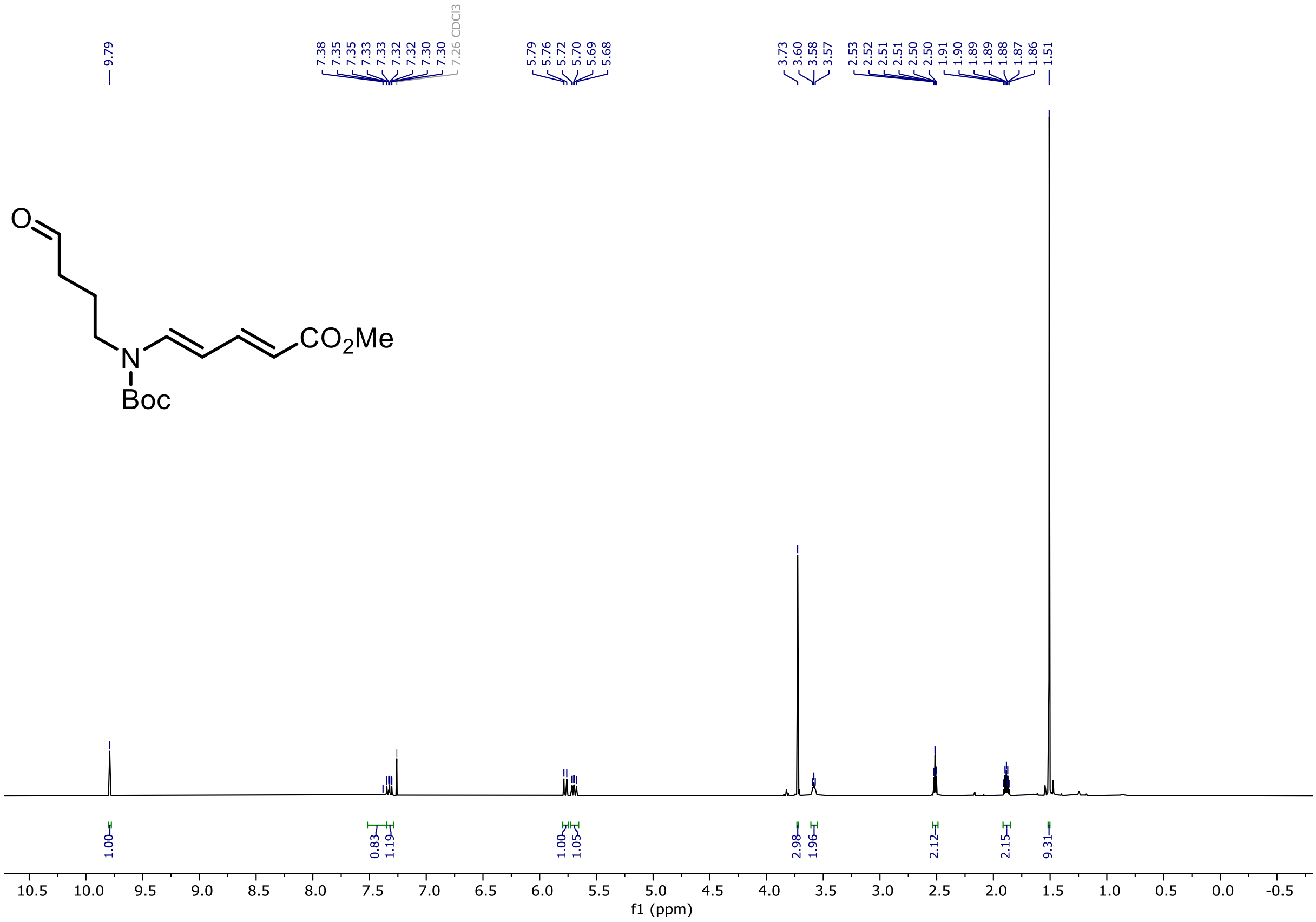

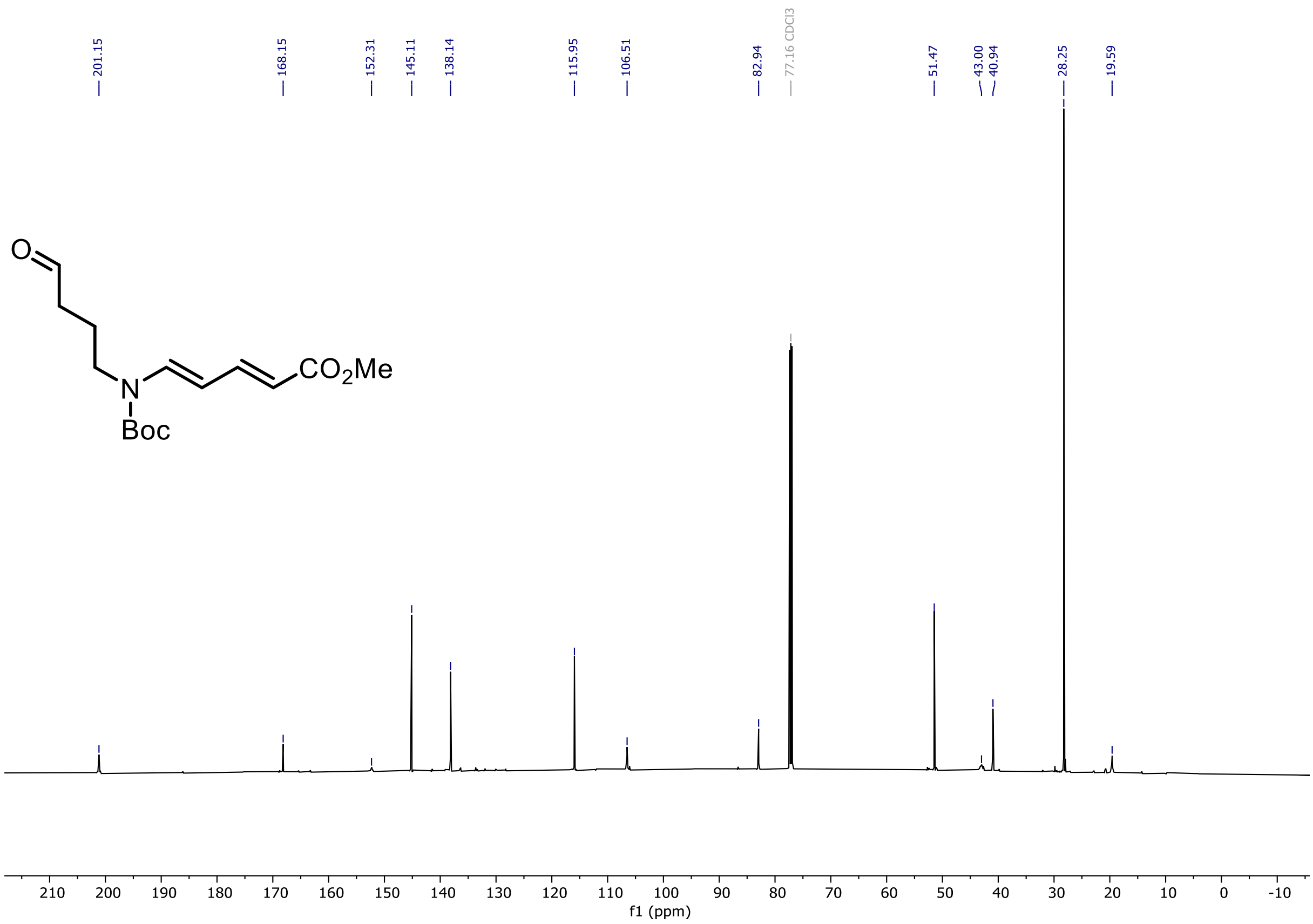

^{1}H NMR (400 MHz, $CDCl_3$; top) and ^{13}C NMR (101 MHz, $CDCl_3$; bottom) of compound **S31**

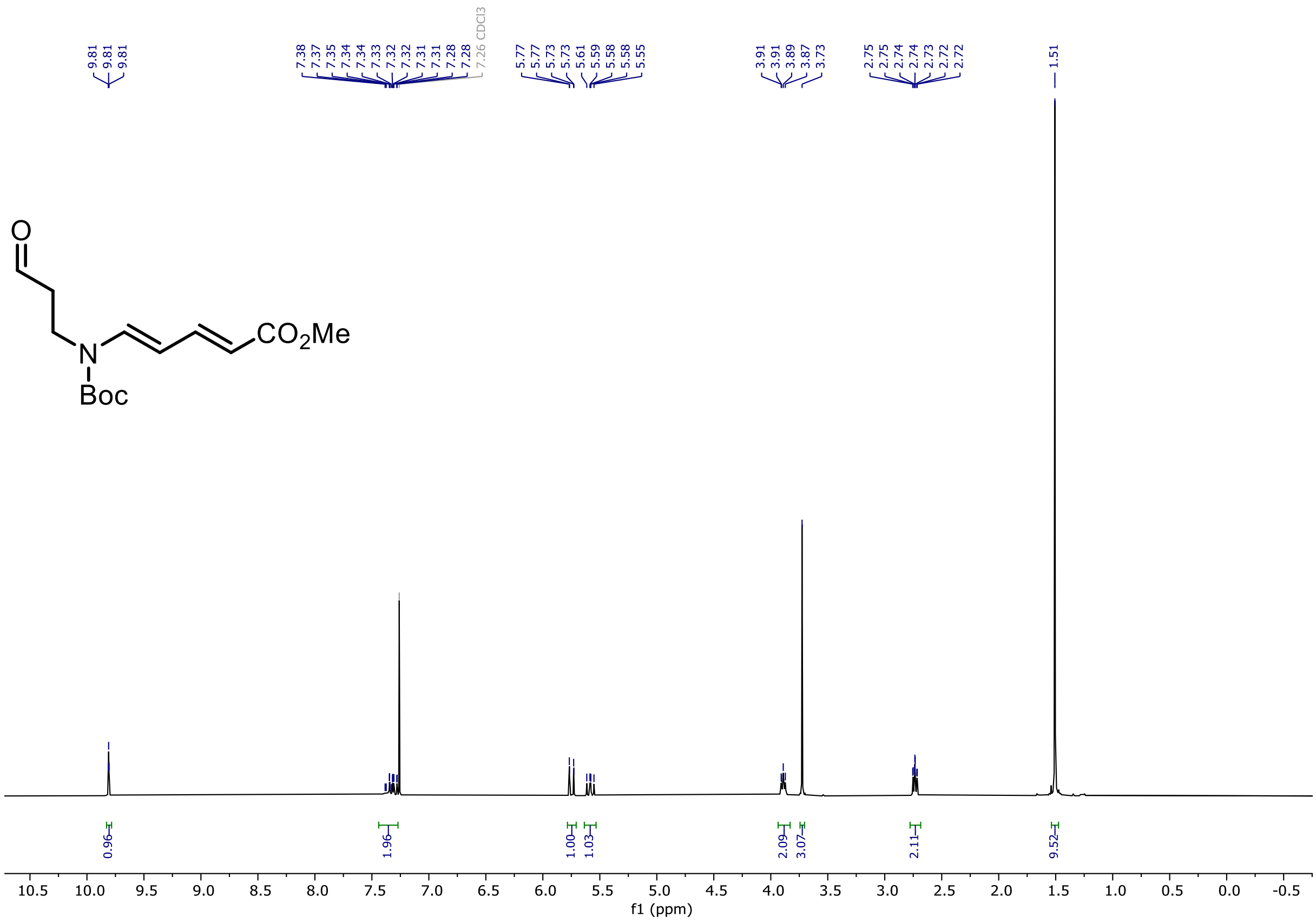

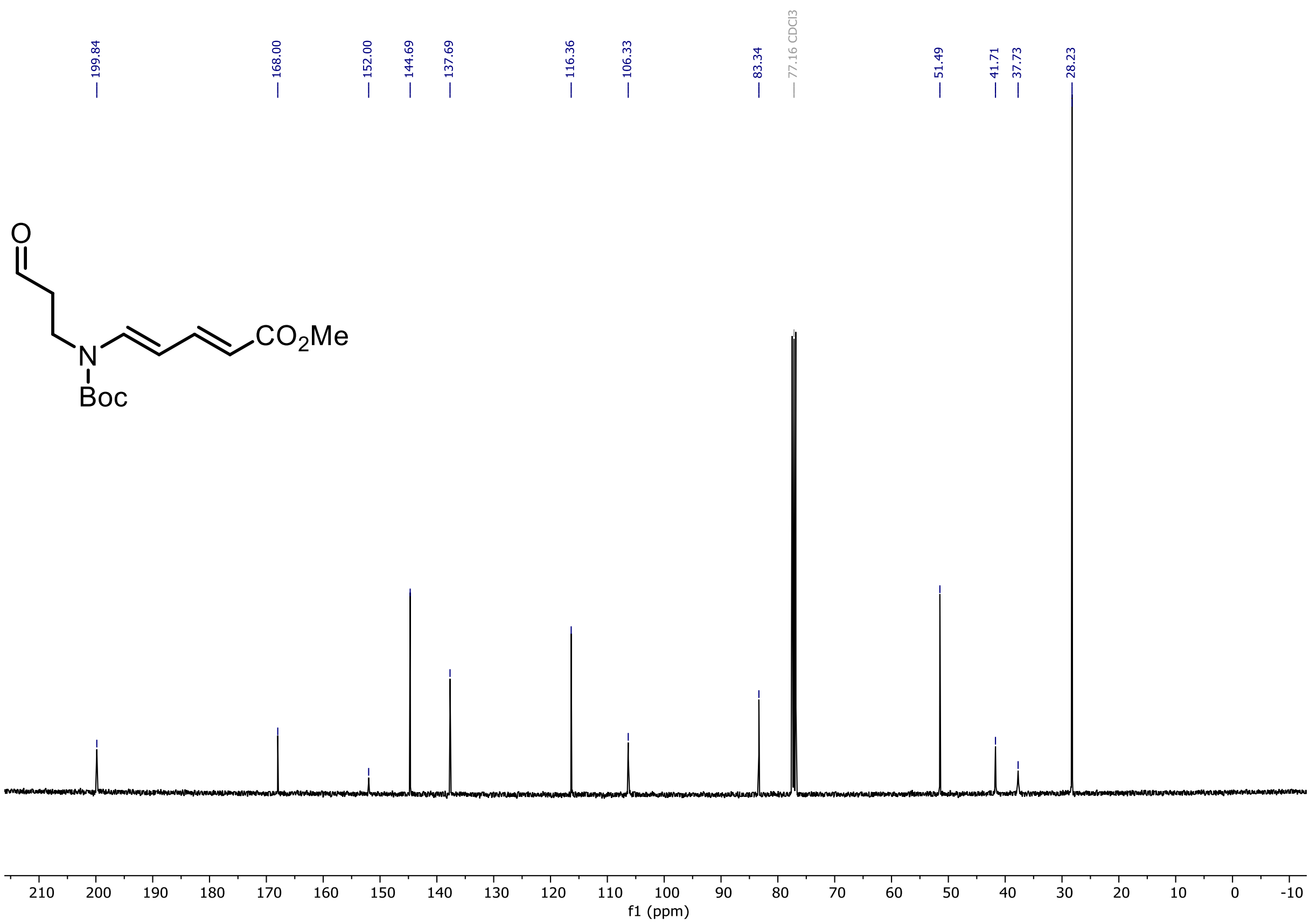

^{1}H NMR (400 MHz, $CDCl_3$; top) and ^{13}C NMR (101 MHz, $CDCl_3$; bottom) of compound **S32**

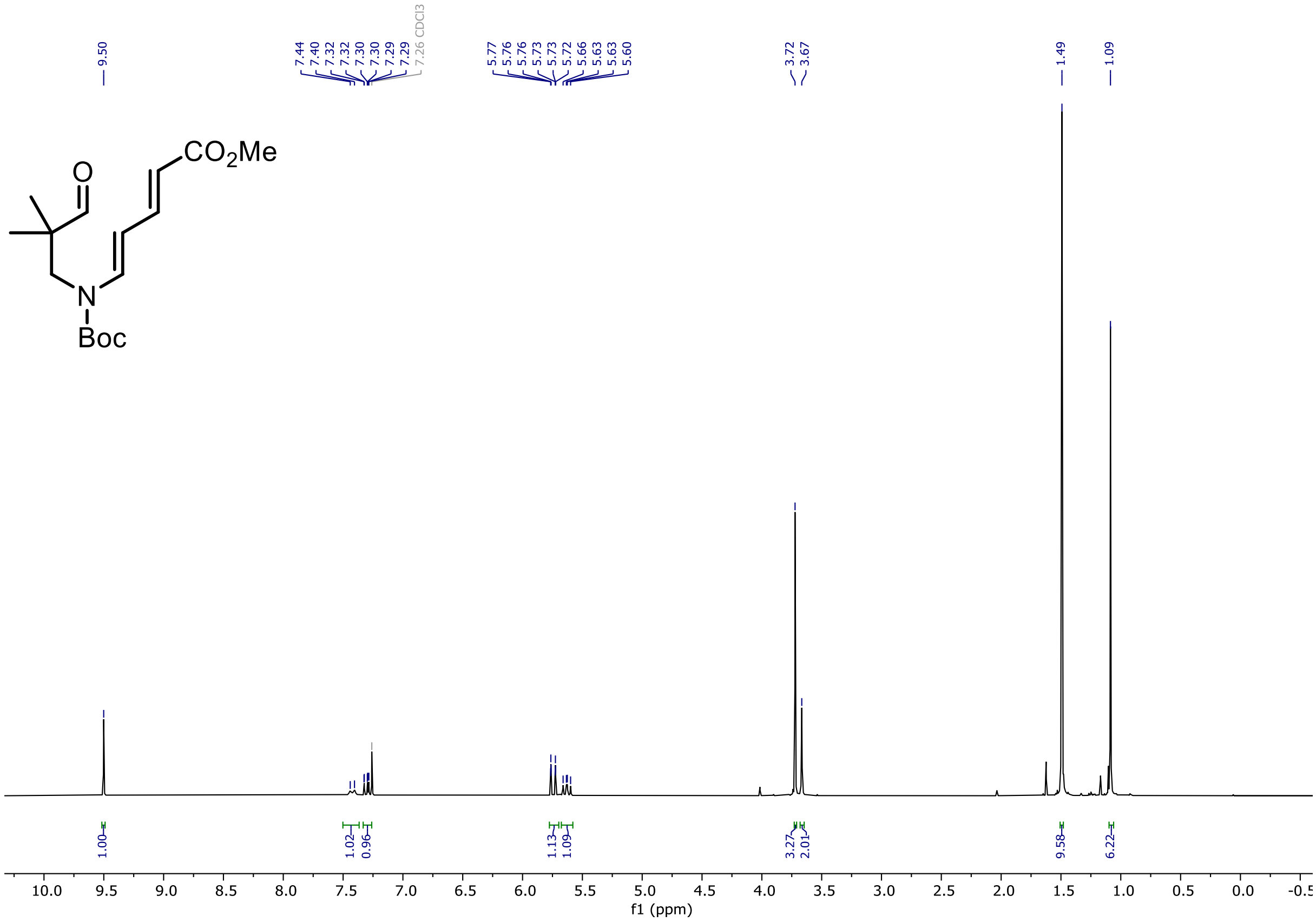

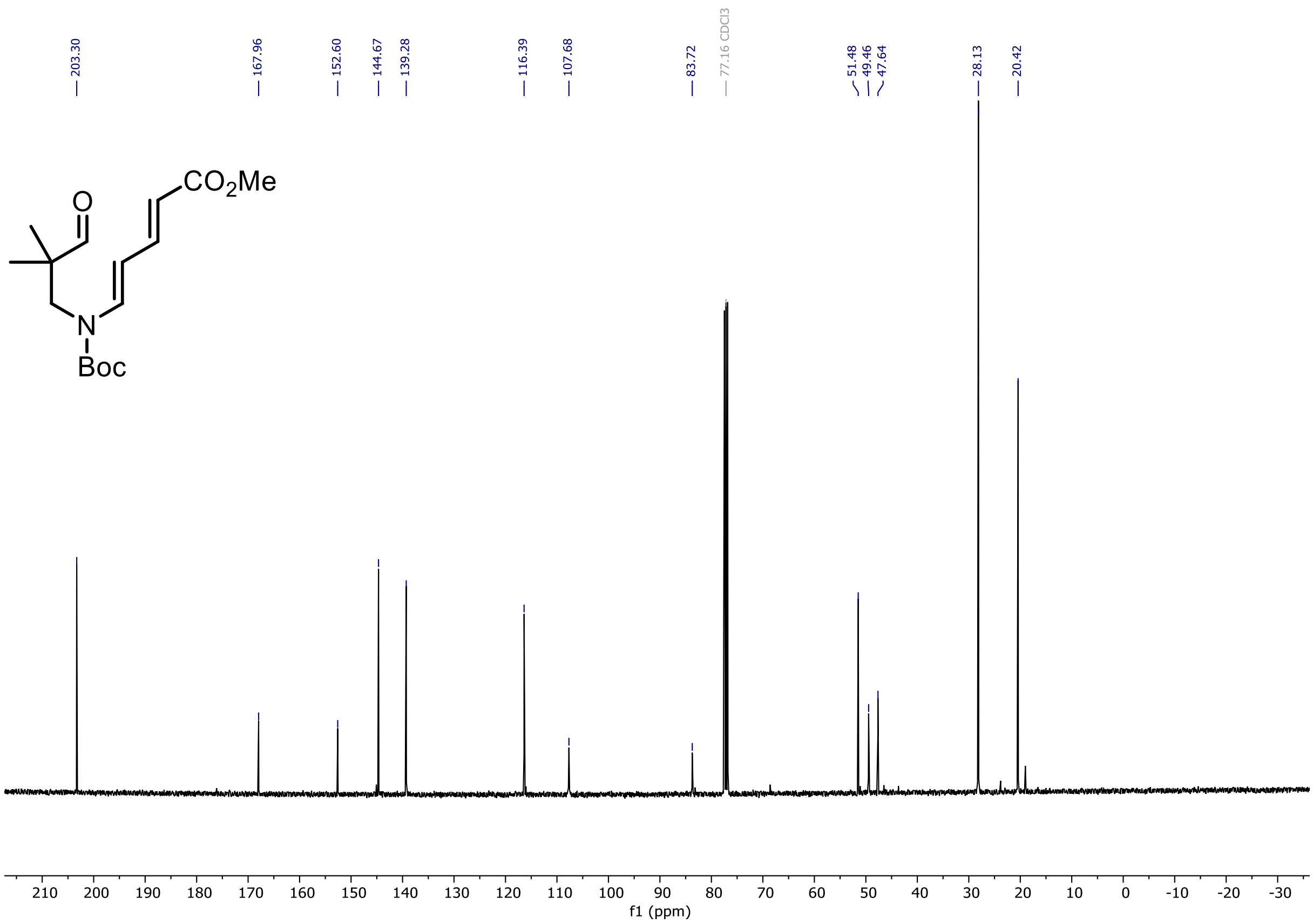

^{1}H NMR (400 MHz, $CDCl_3$; top) and ^{13}C NMR (101 MHz, $CDCl_3$; bottom) of compound **S33**

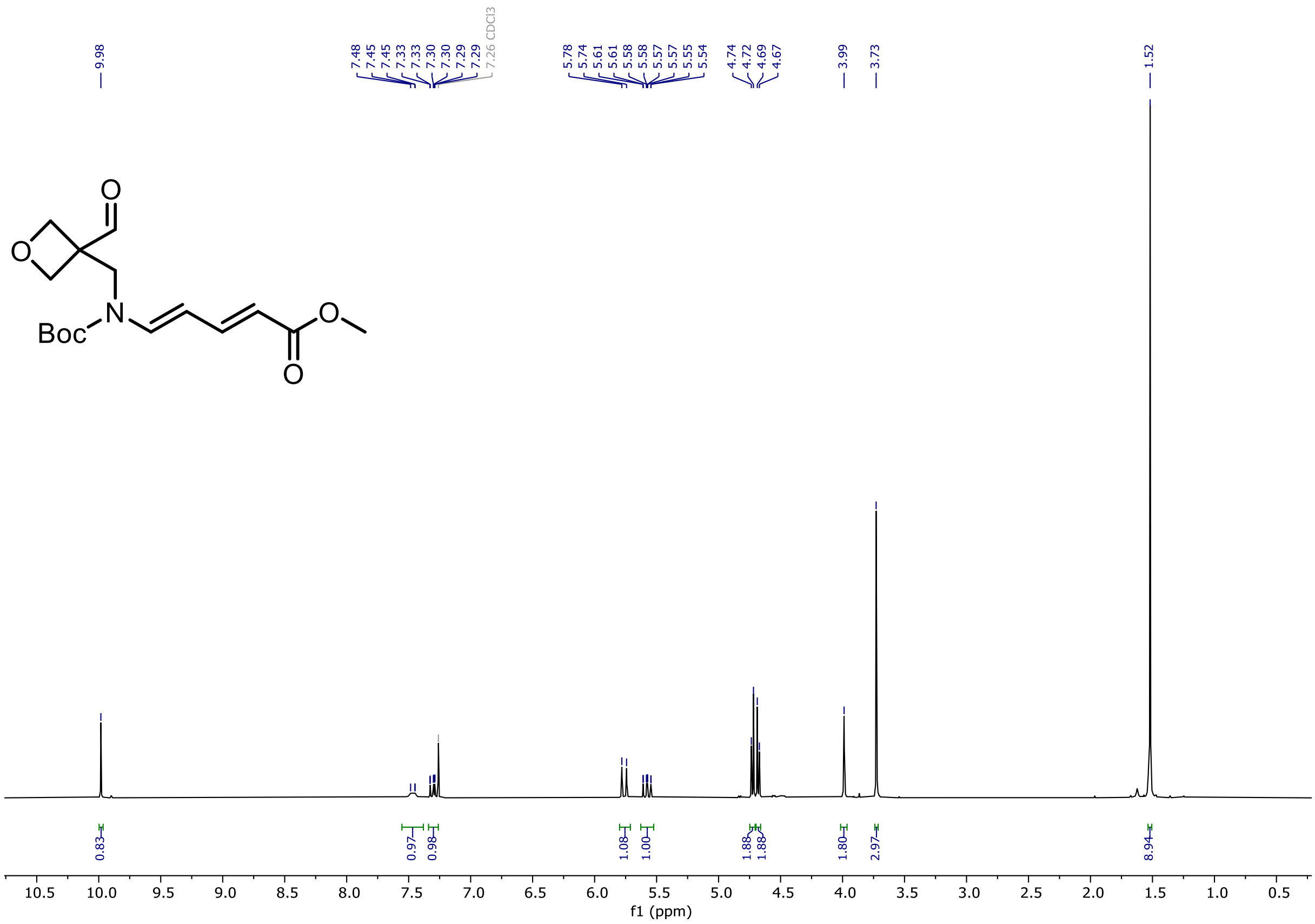

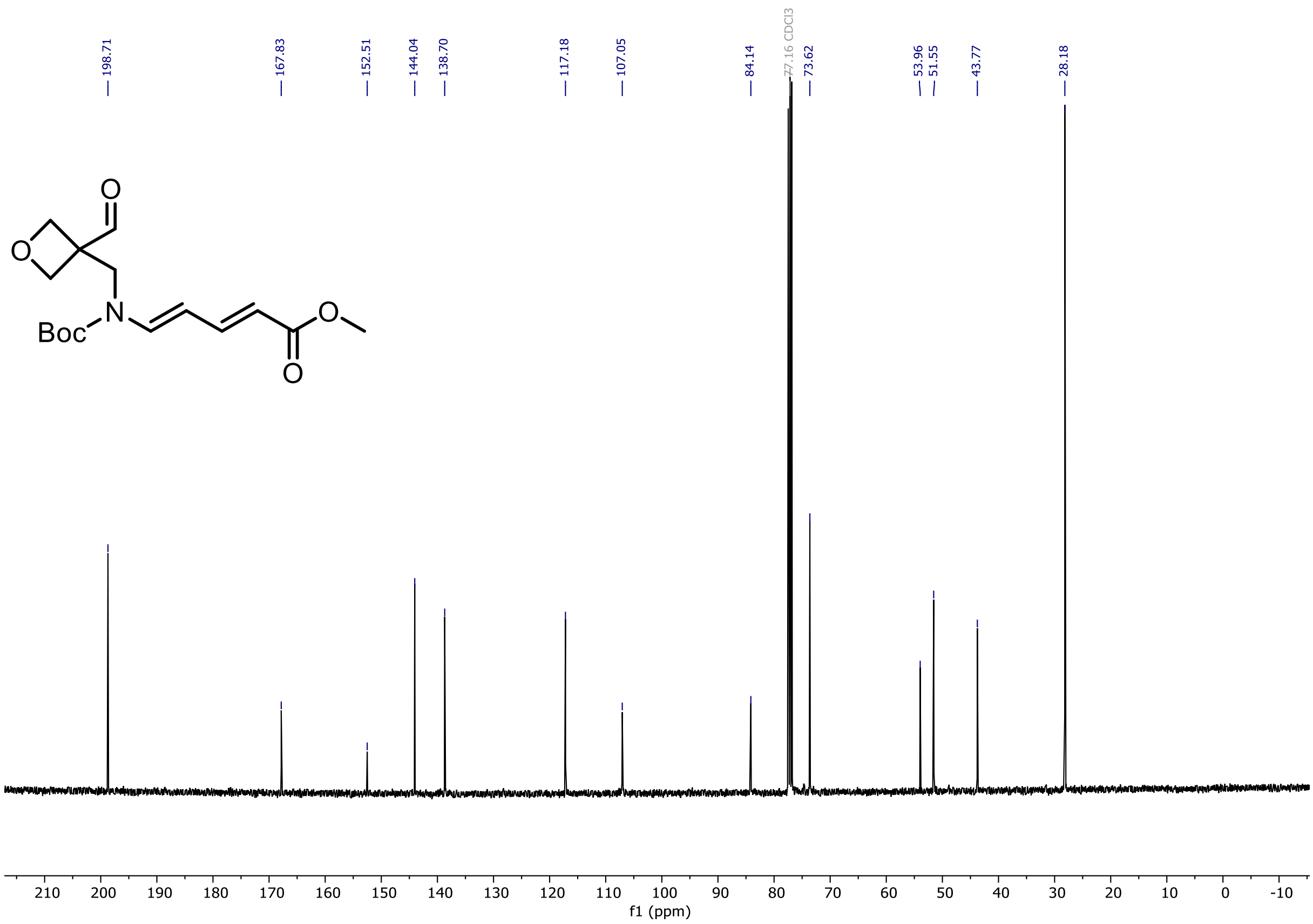

^1H NMR (400 MHz, CD_2Cl_2; top) and ^{13}C NMR (101 MHz, CD_2Cl_2; bottom) of compound **S34**

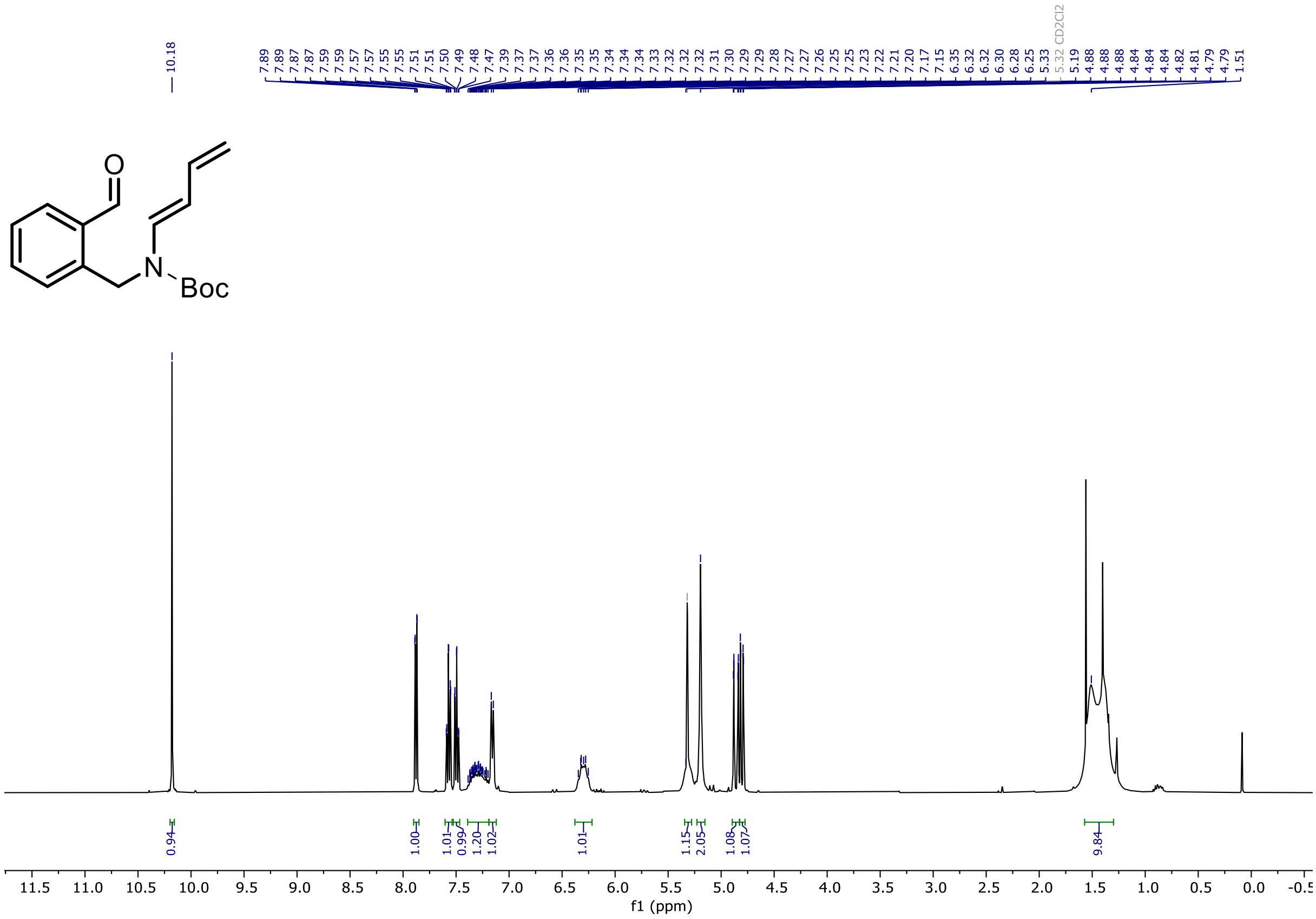

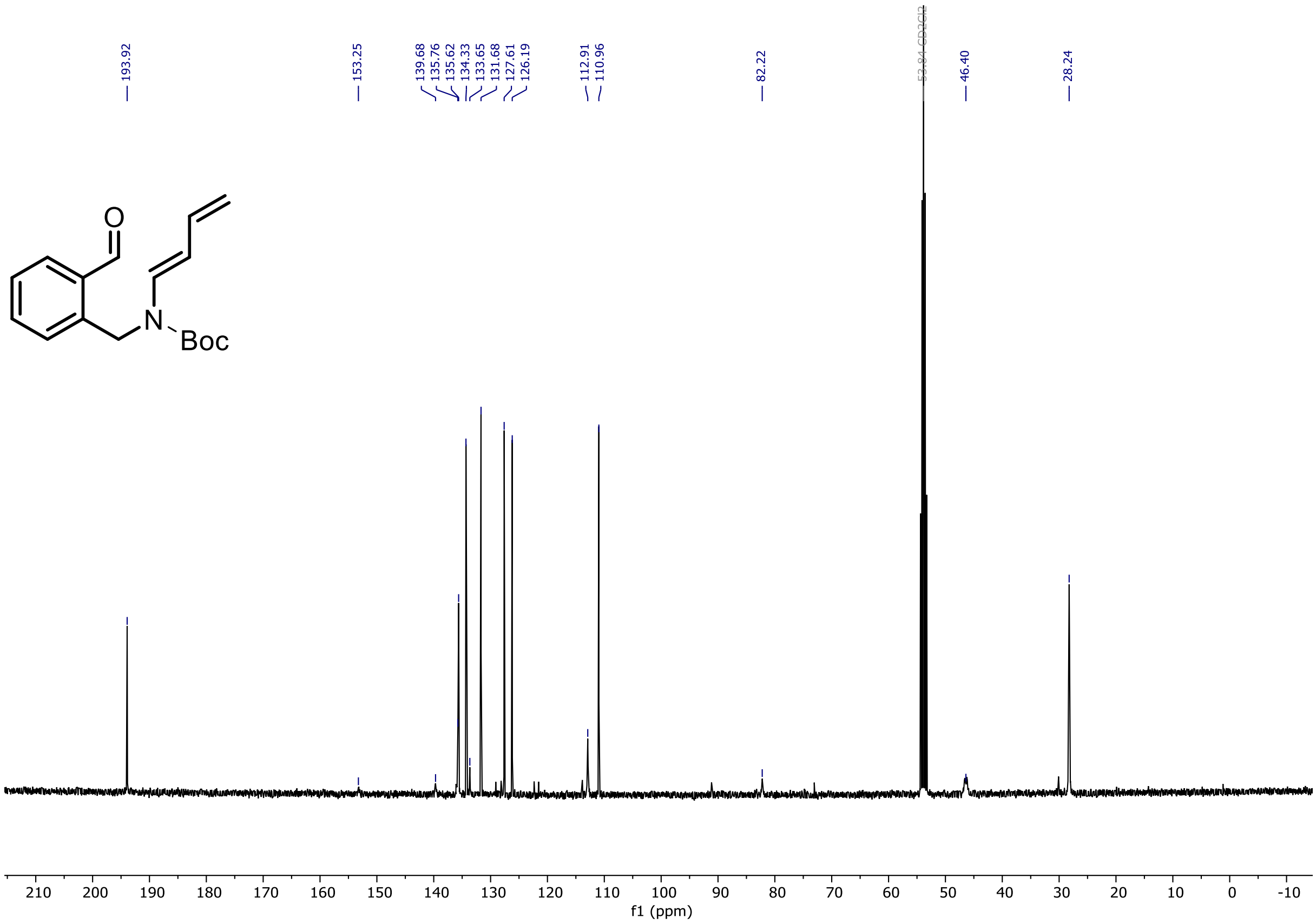

^{1}H NMR (600 MHz, [D$_6$]-DMSO, 383K ; top) and ^{13}C NMR (151 MHz, [D$_6$]-DMSO, 383K; bottom) of compound **S35**

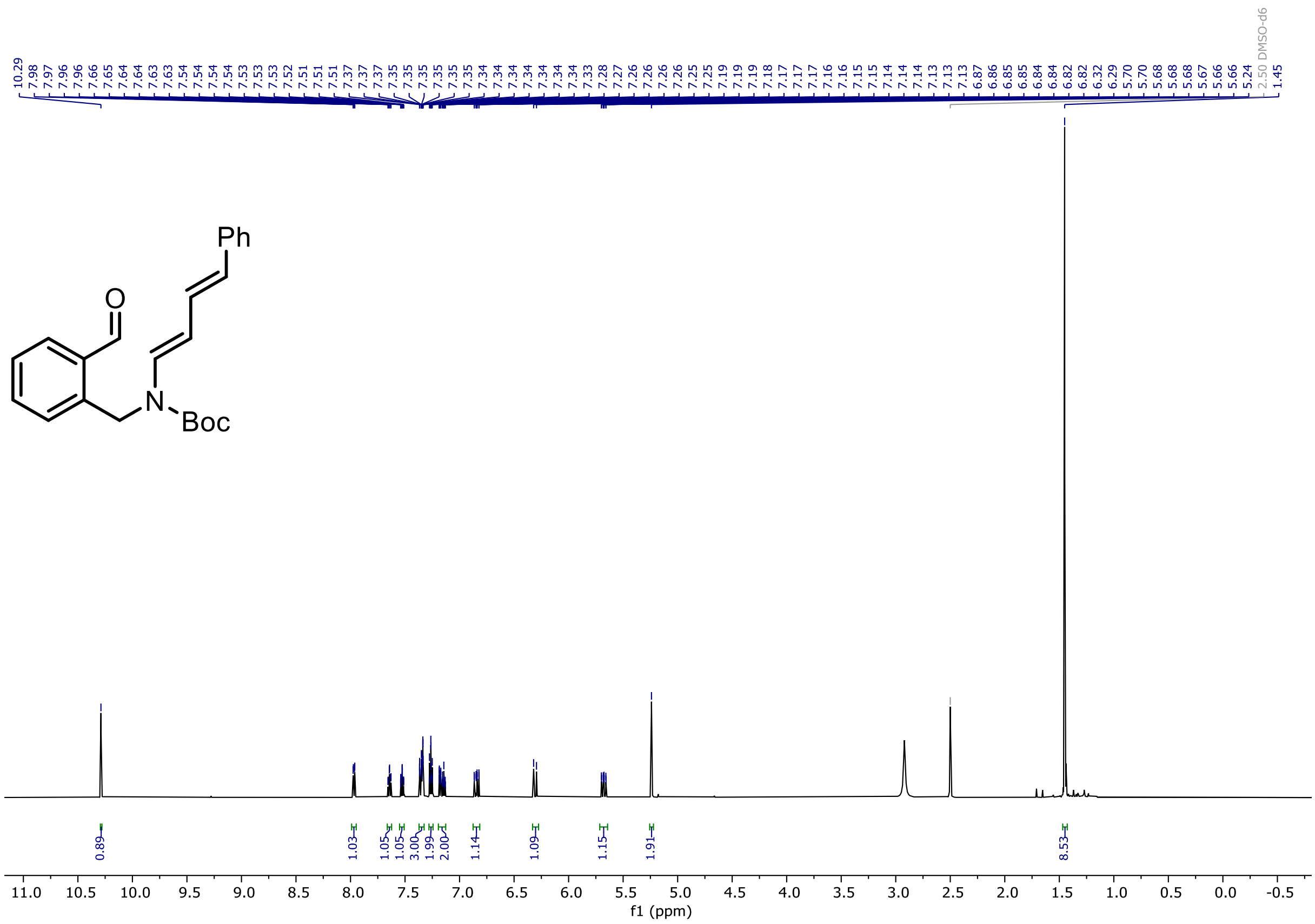

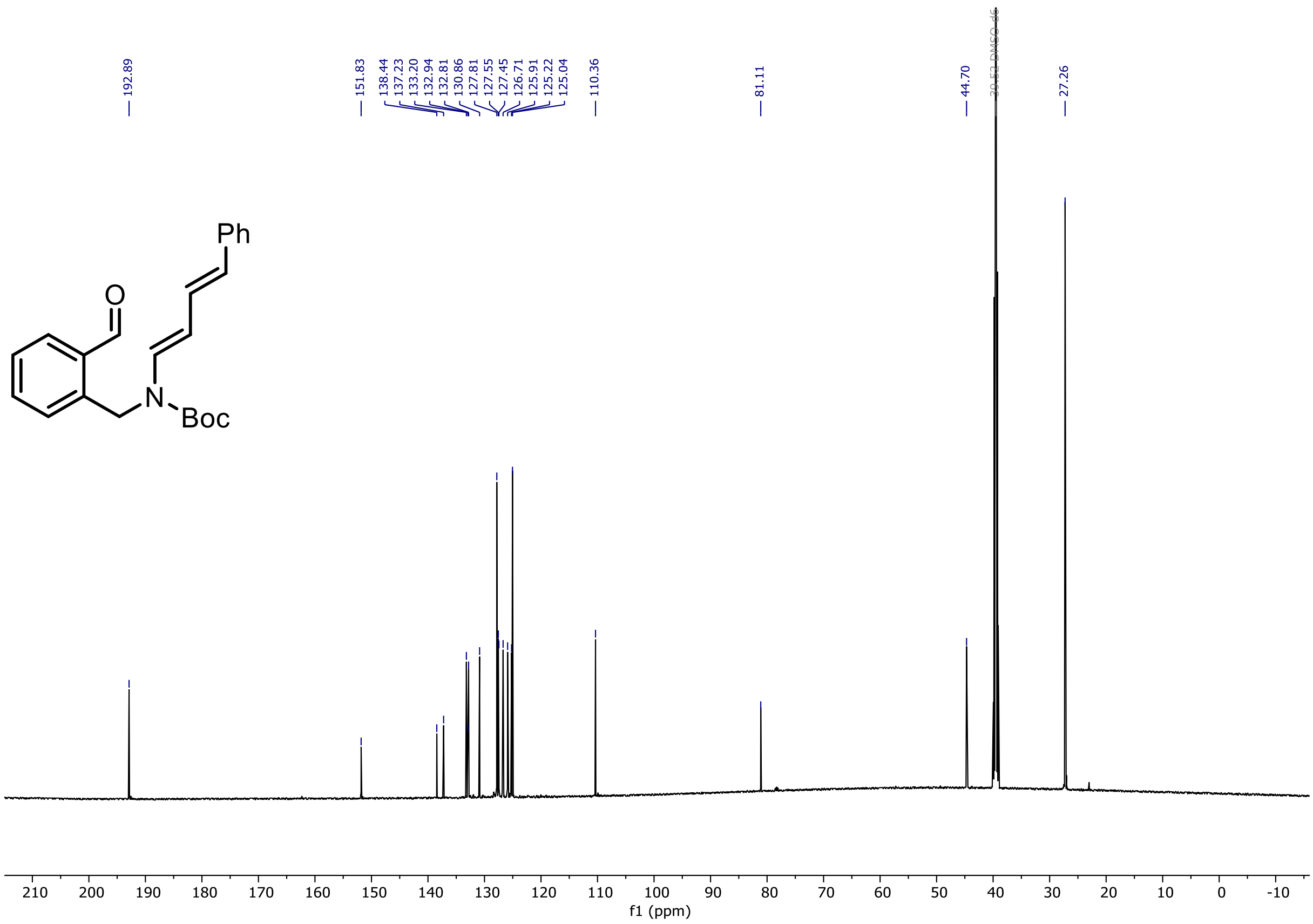

^1H NMR (400 MHz, CD_2Cl_2; top) and ^{13}C NMR (101 MHz, CD_2Cl_2; bottom) of compound **S36**

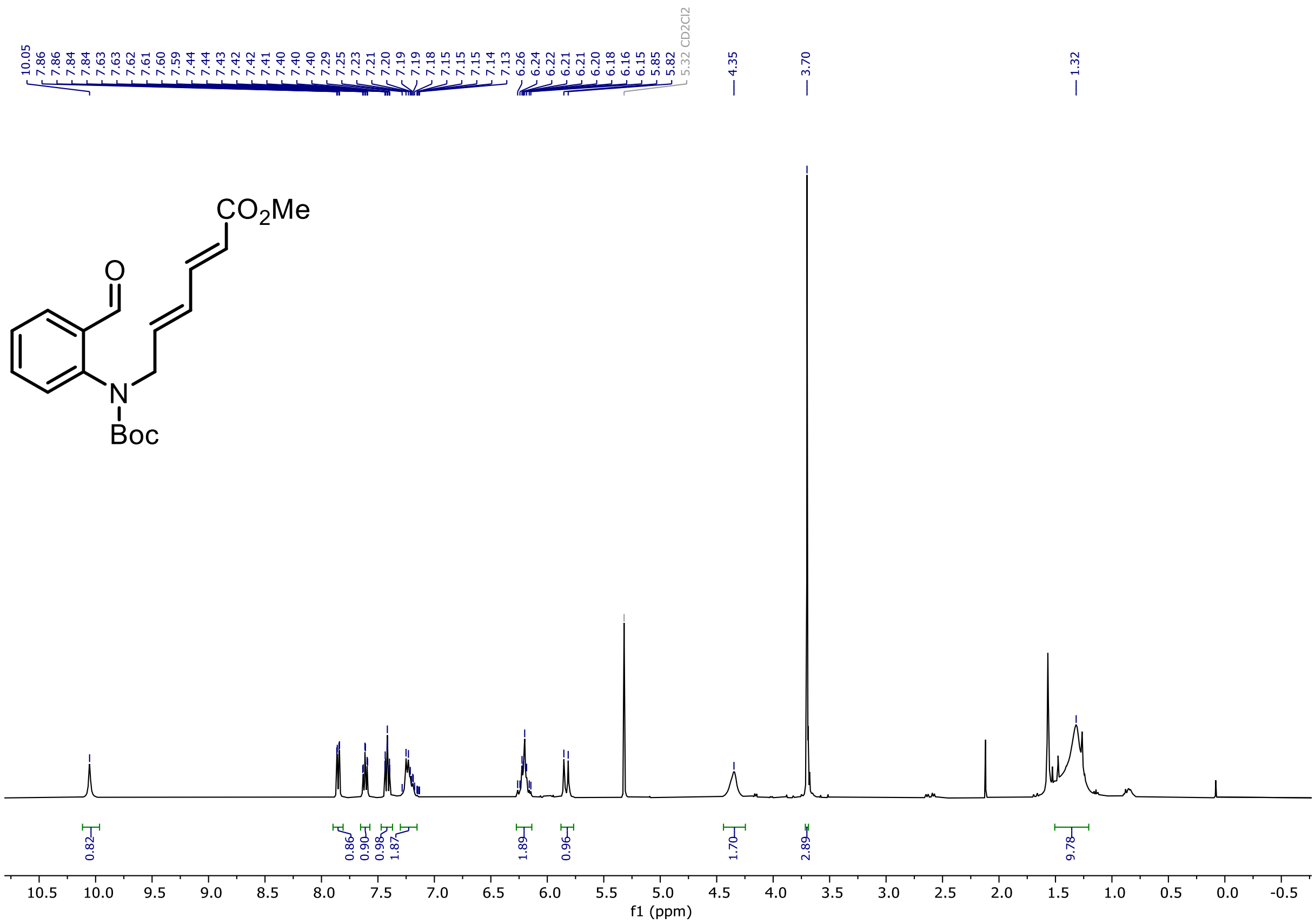

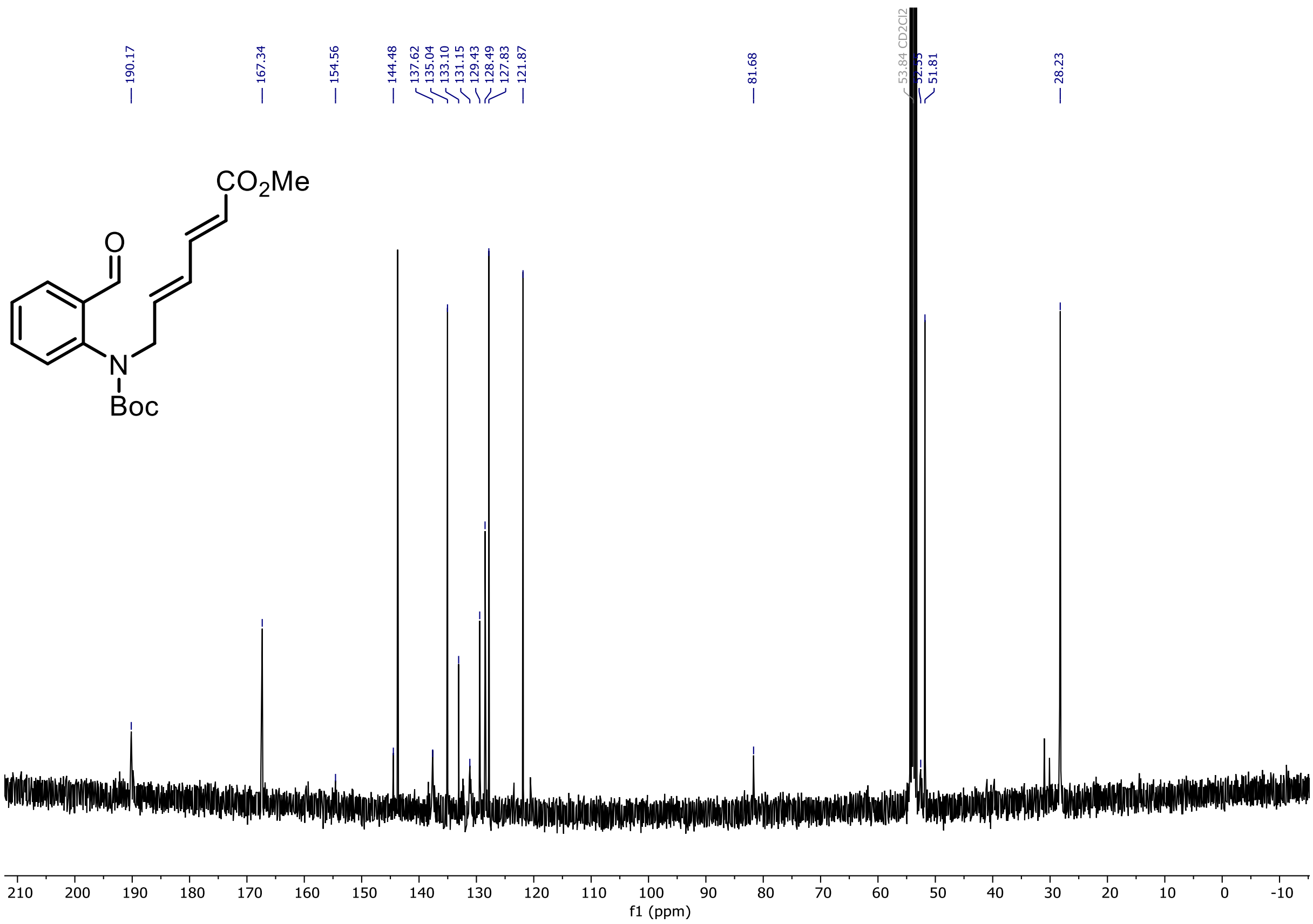

^{1}H NMR (600 MHz, [D$_6$]-DMSO, 383K; top) and ^{13}C NMR (151 MHz, [D$_6$]-DMSO, 383K; bottom) of compound **8a**

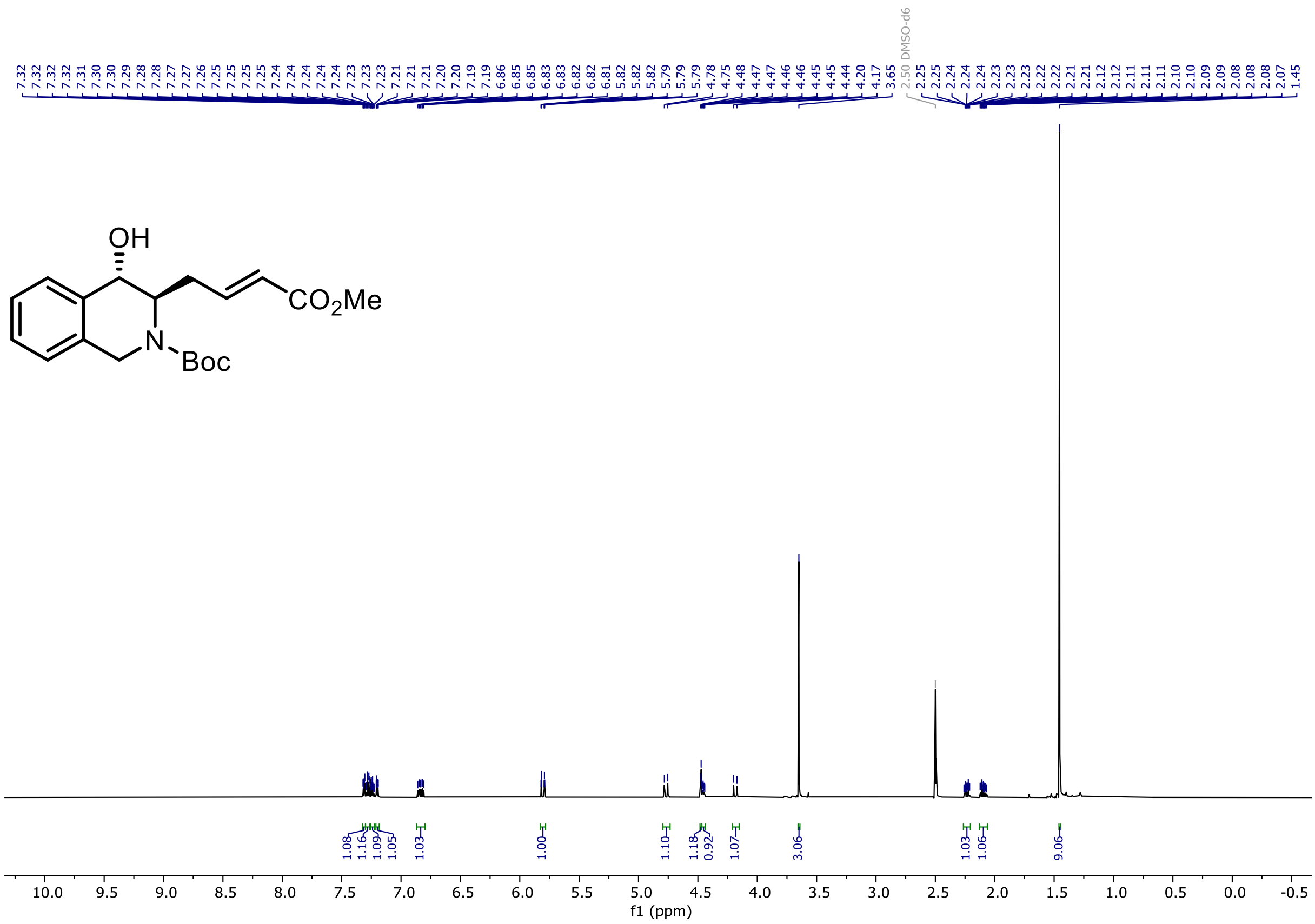

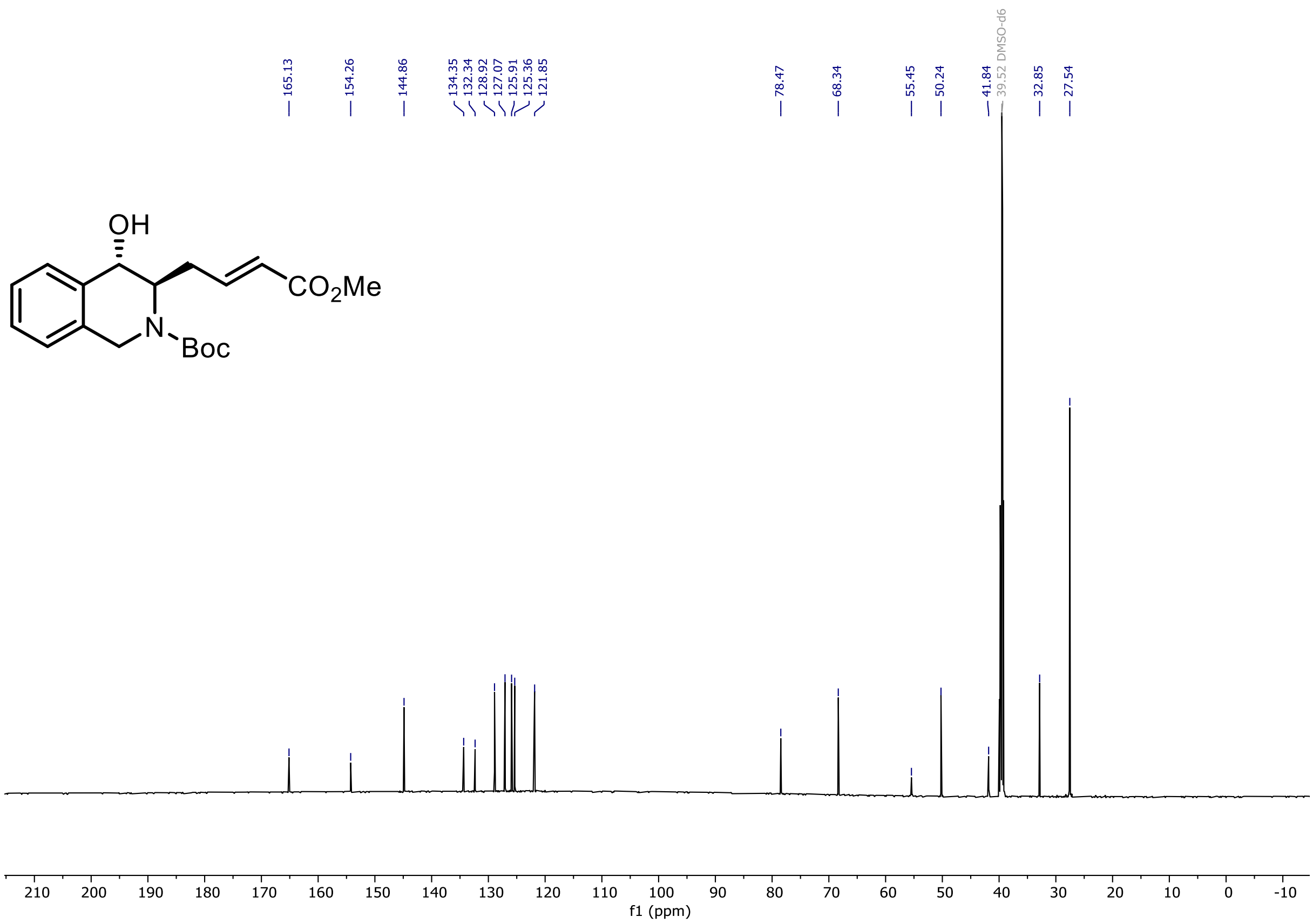

^{1}H NMR (600 MHz, [D$_6$]-DMSO, 383K; top) and ^{13}C NMR (151 MHz, [D$_6$]-DMSO, 383K; bottom) of compound **8b**

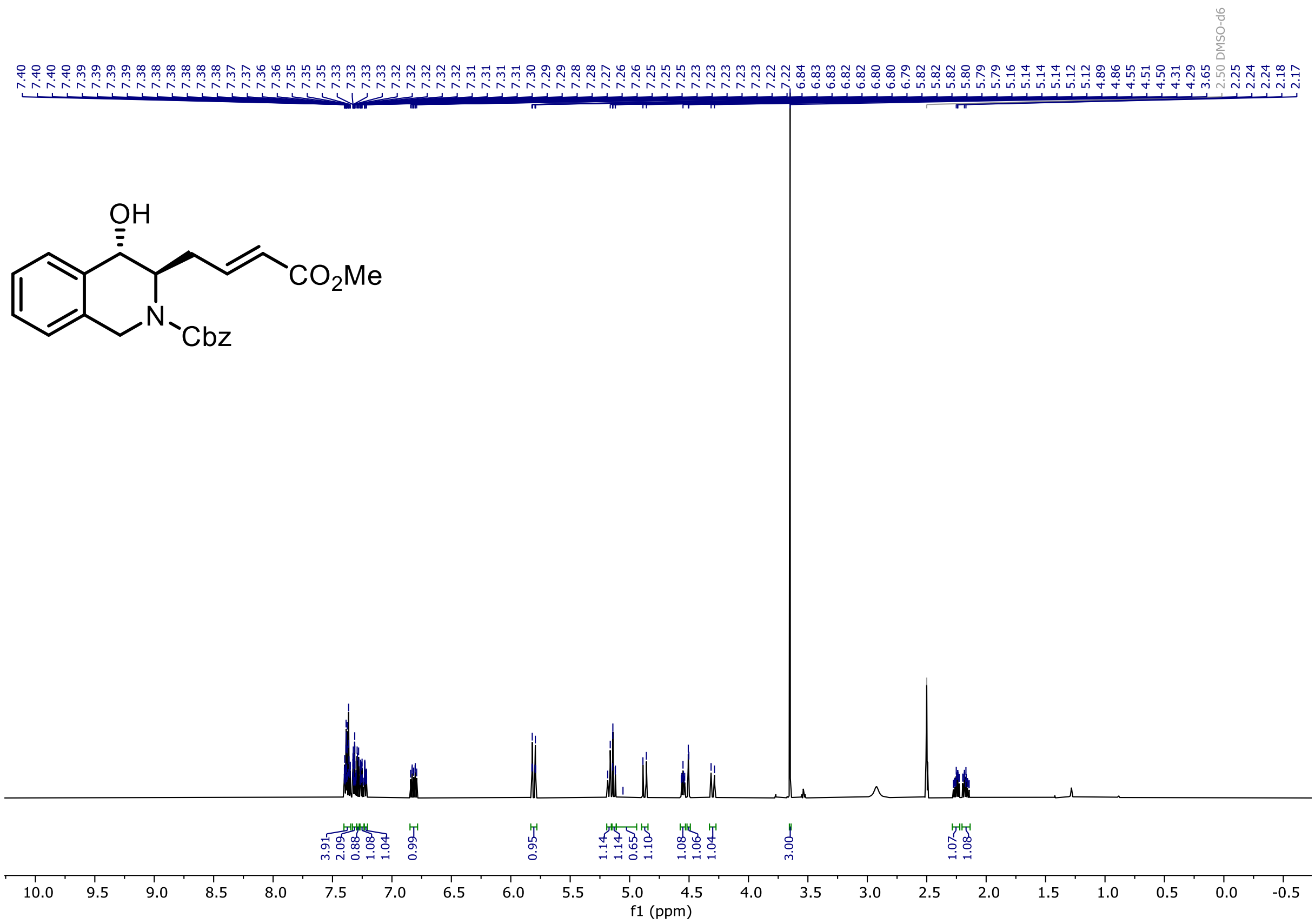

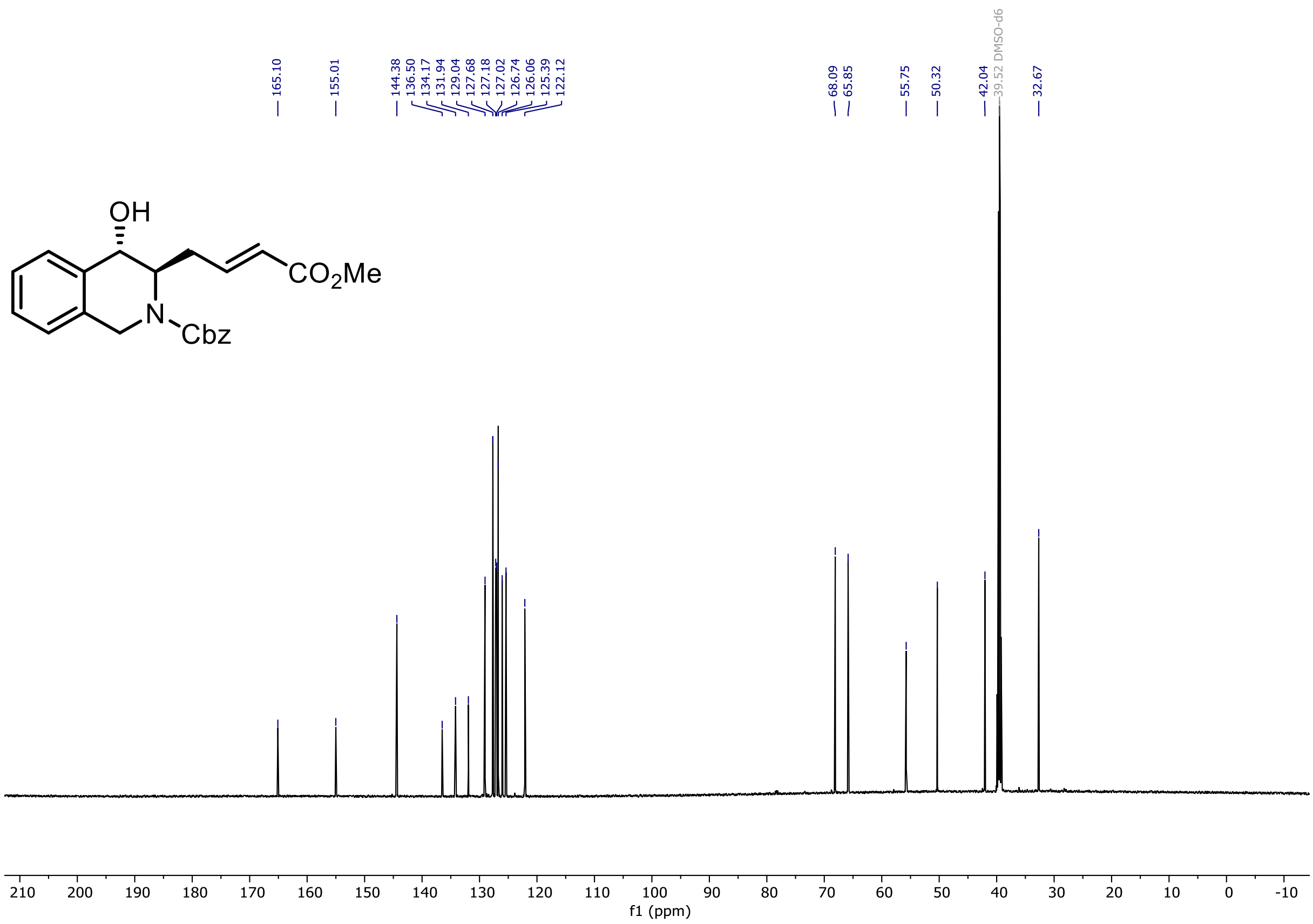

^{1}H NMR (600 MHz, [D$_6$]-DMSO, 383K; top) and ^{19}F NMR (585 MHz, [D$_6$]-DMSO, 383K; bottom) of compound **8c**

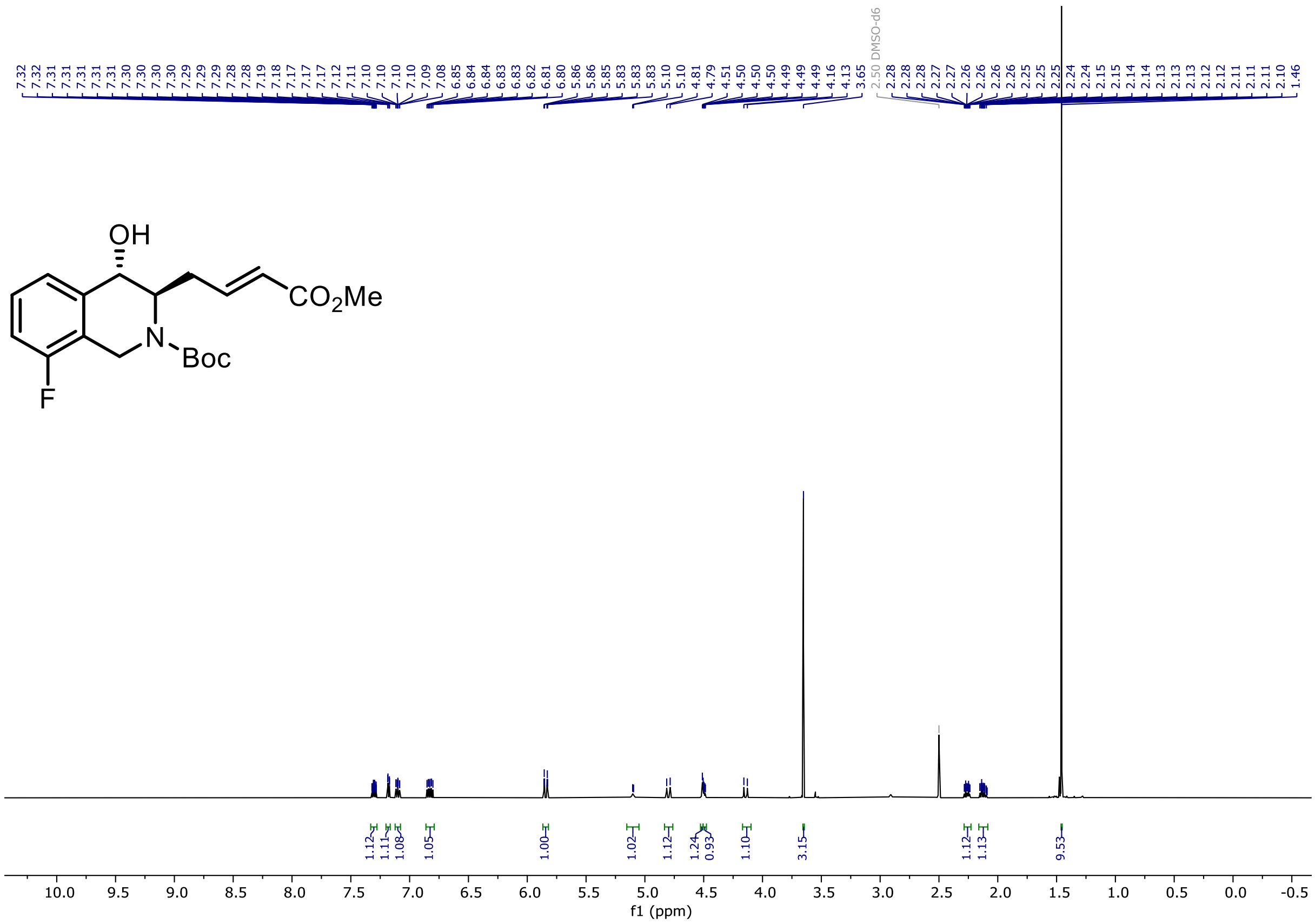

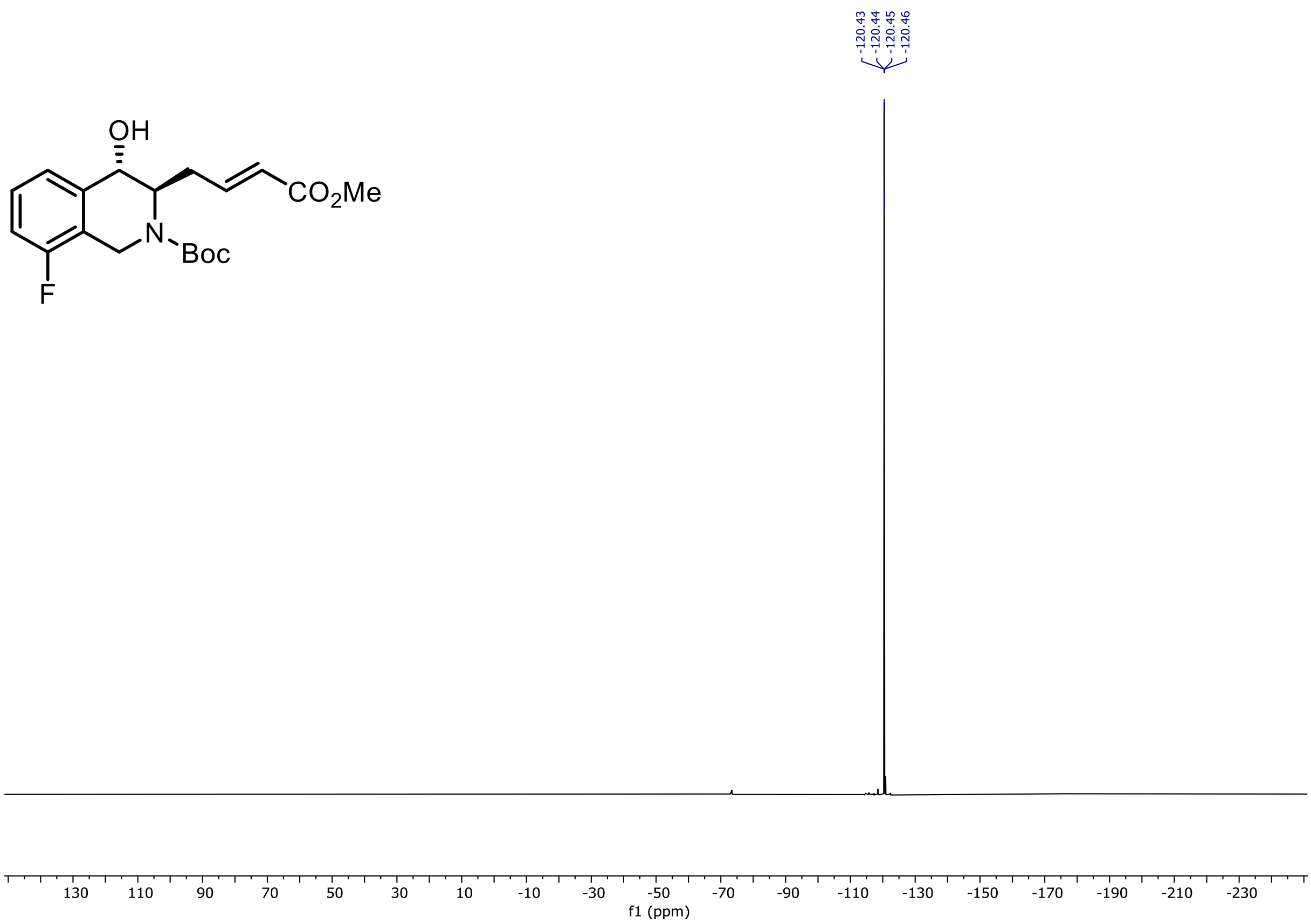

^{13}C NMR (151 MHz, [D6]-DMSO, 383K) of compound **8c**

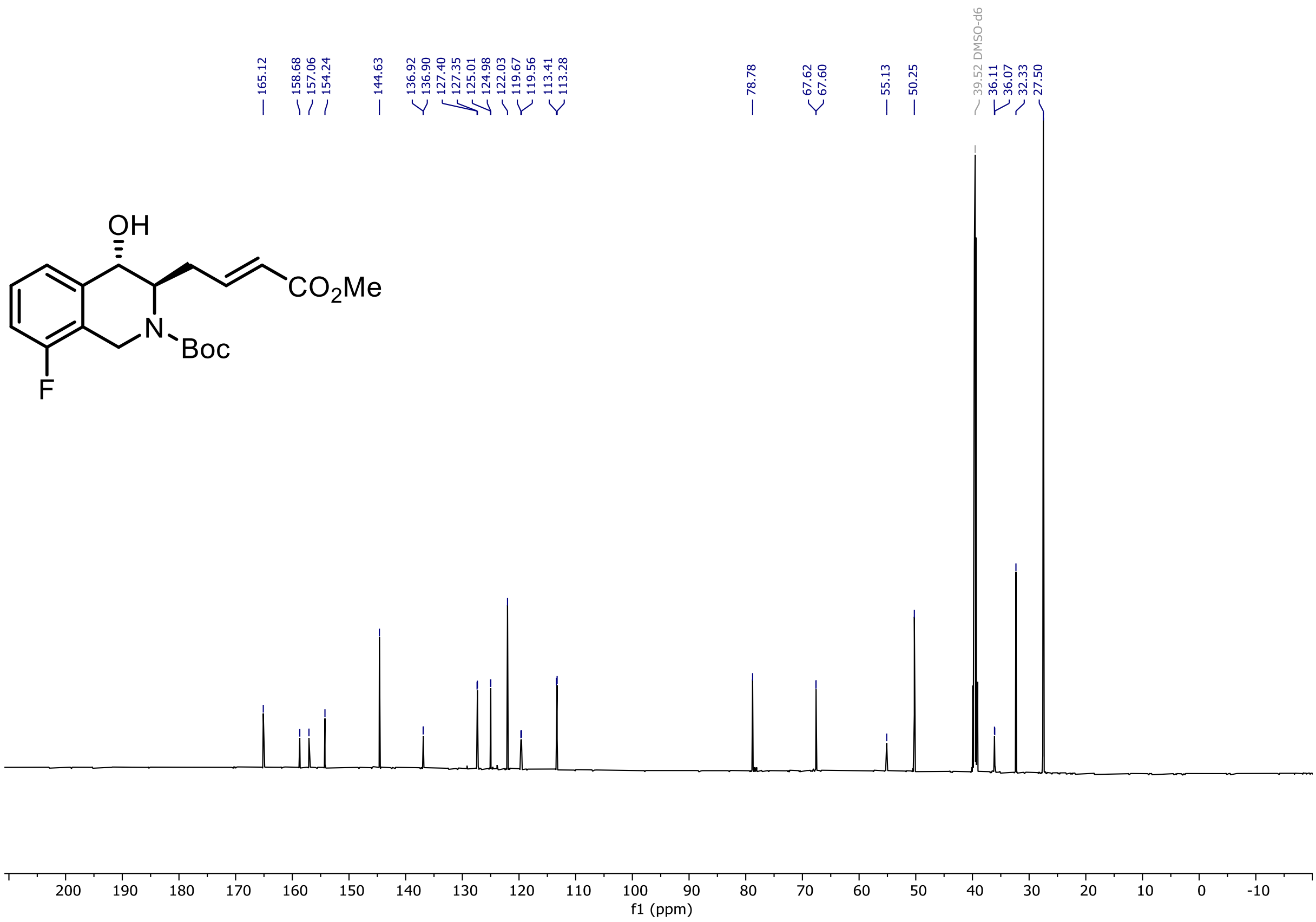

^{1}H NMR (600 MHz, [D$_6$]-DMSO, 383K; top) and ^{19}F NMR (585 MHz, [D$_6$]-DMSO, 383K; bottom) of compound **8d**

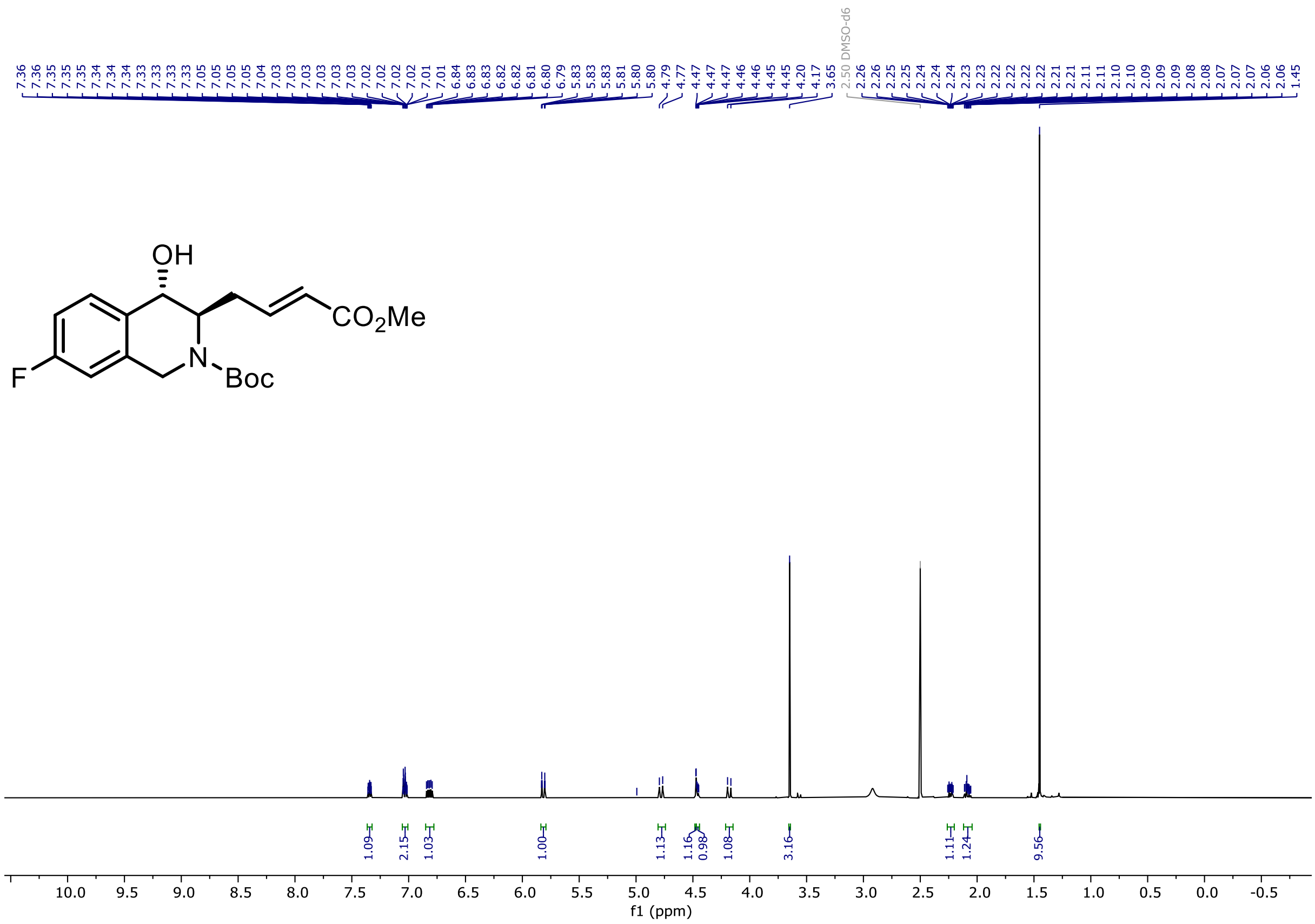

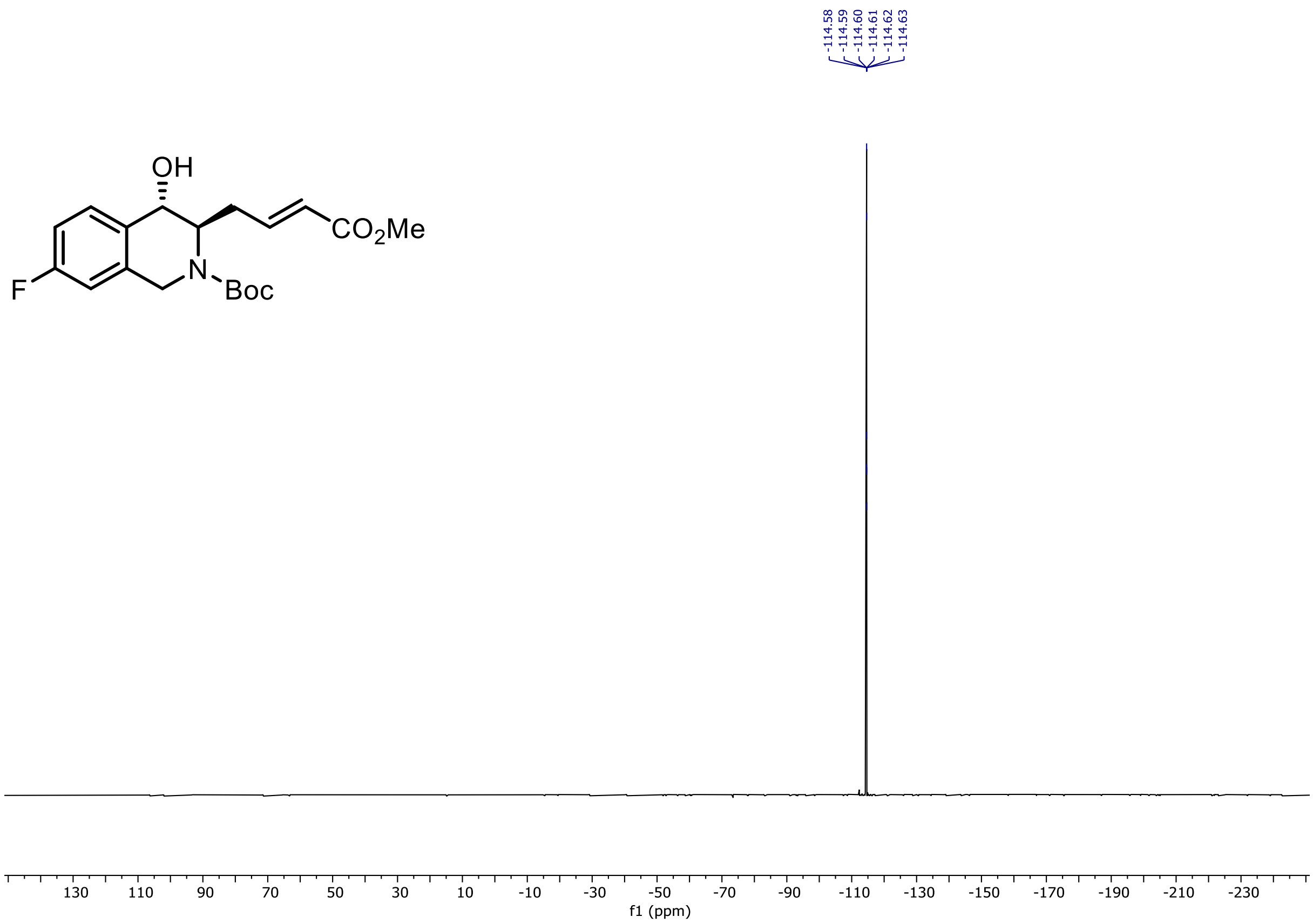

^{13}C NMR (151 MHz, [D$_6$]-DMSO, 383K) of compound **8d**

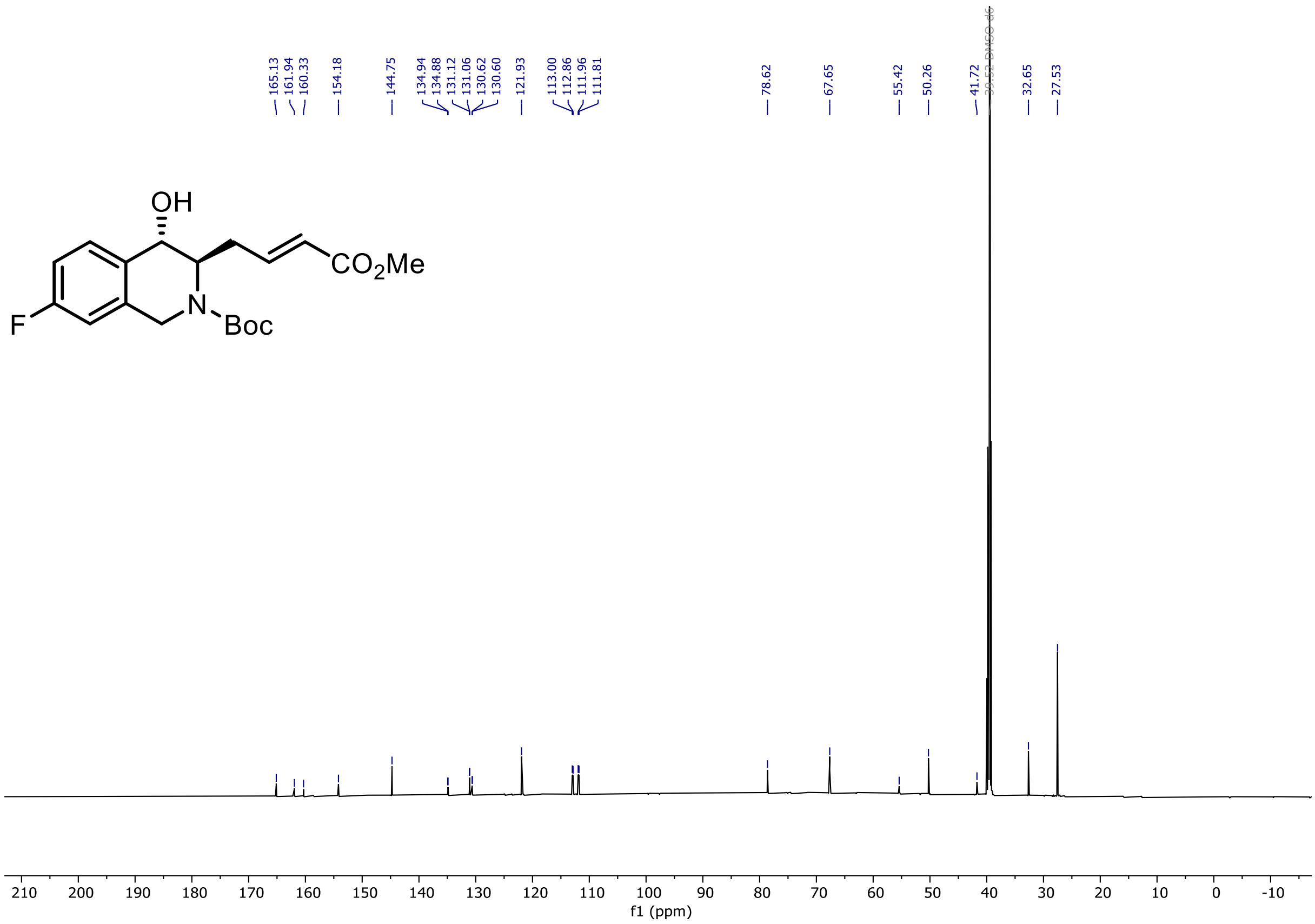

^{1}H NMR (600 MHz, [D$_6$]-DMSO, 383K; top) and ^{19}F NMR (585 MHz, [D$_6$]-DMSO, 383K; bottom) of compound **8e**

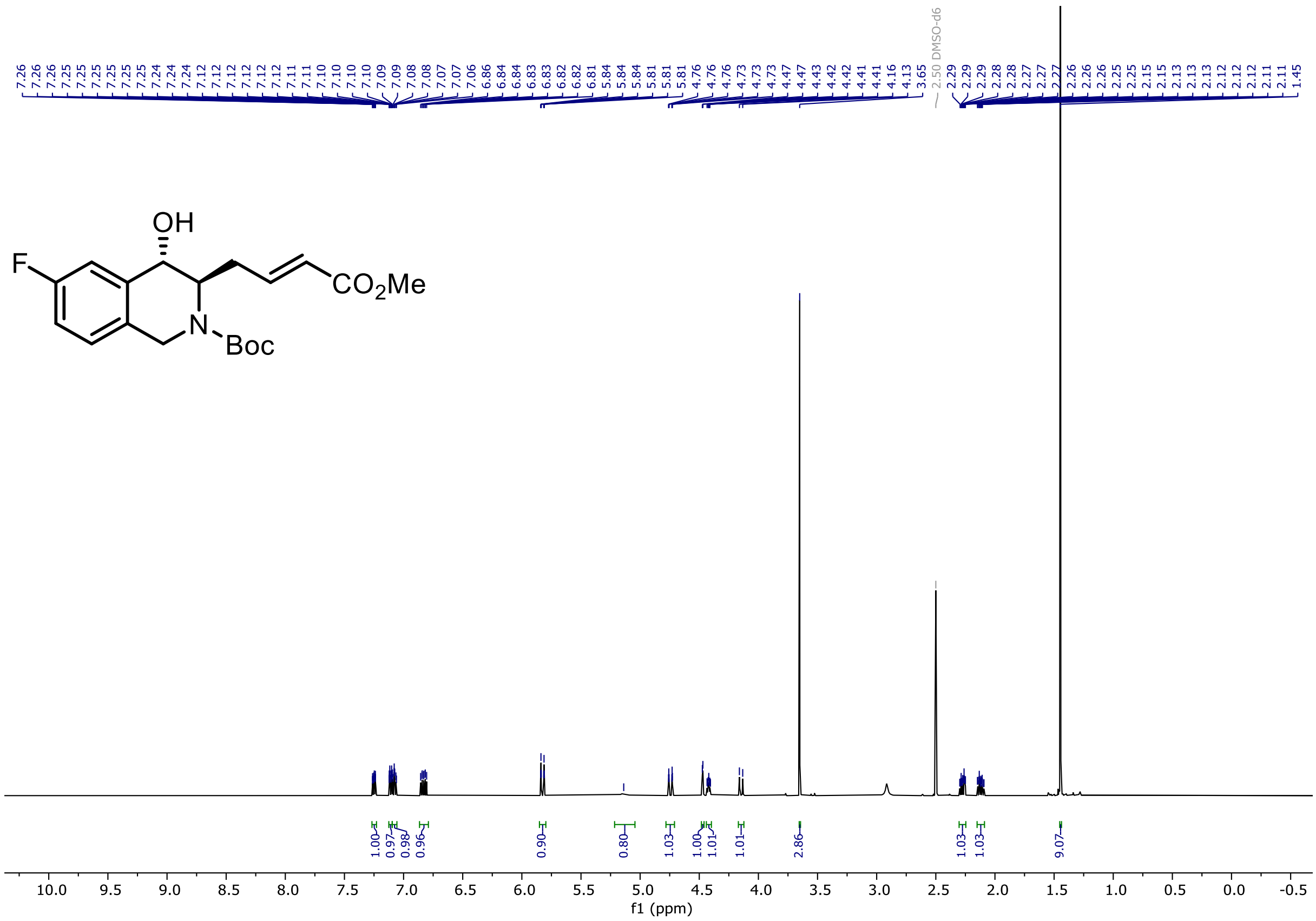

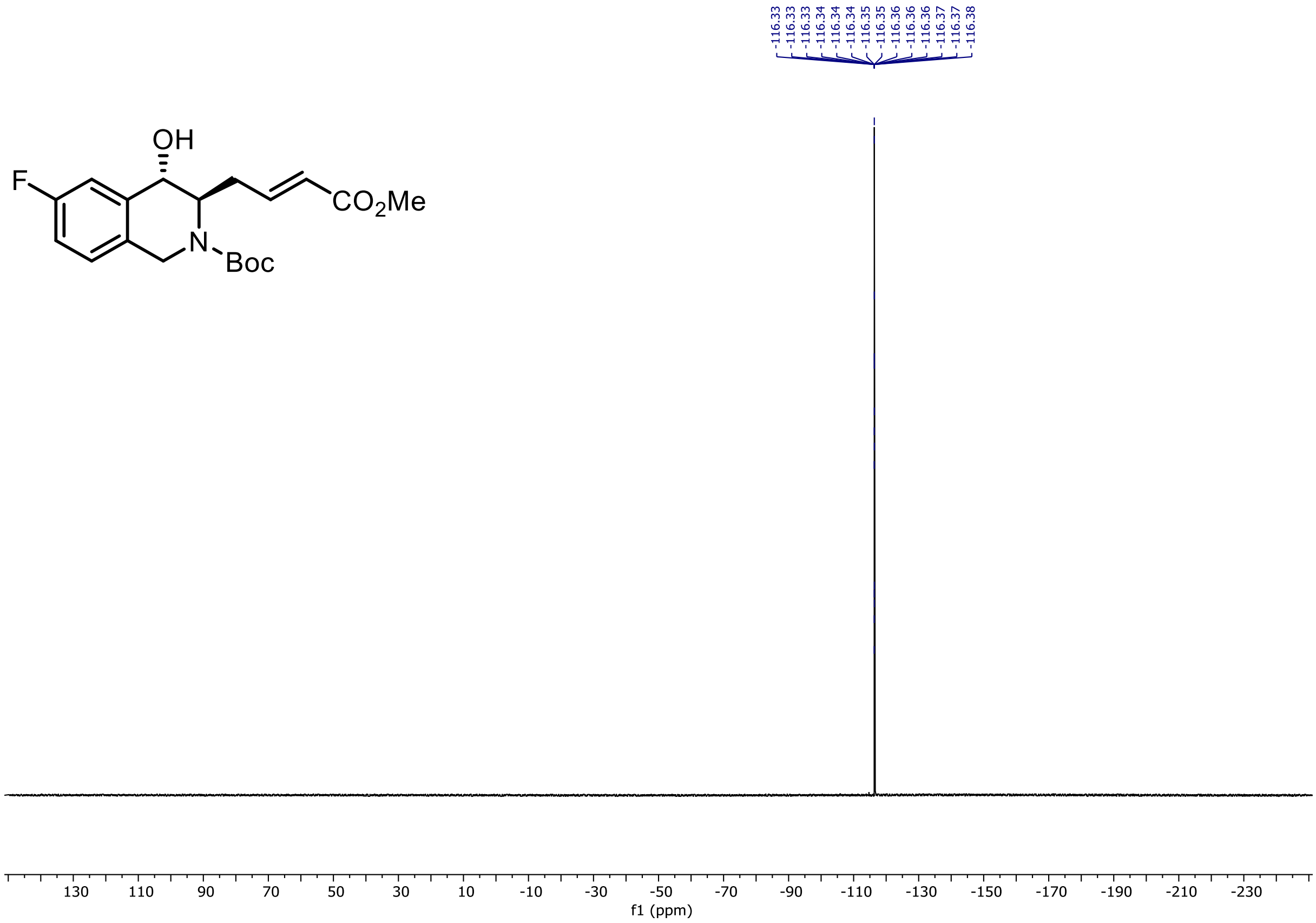

^{13}C NMR (151 MHz, [D$_6$]-DMSO, 383K) of compound **8e**

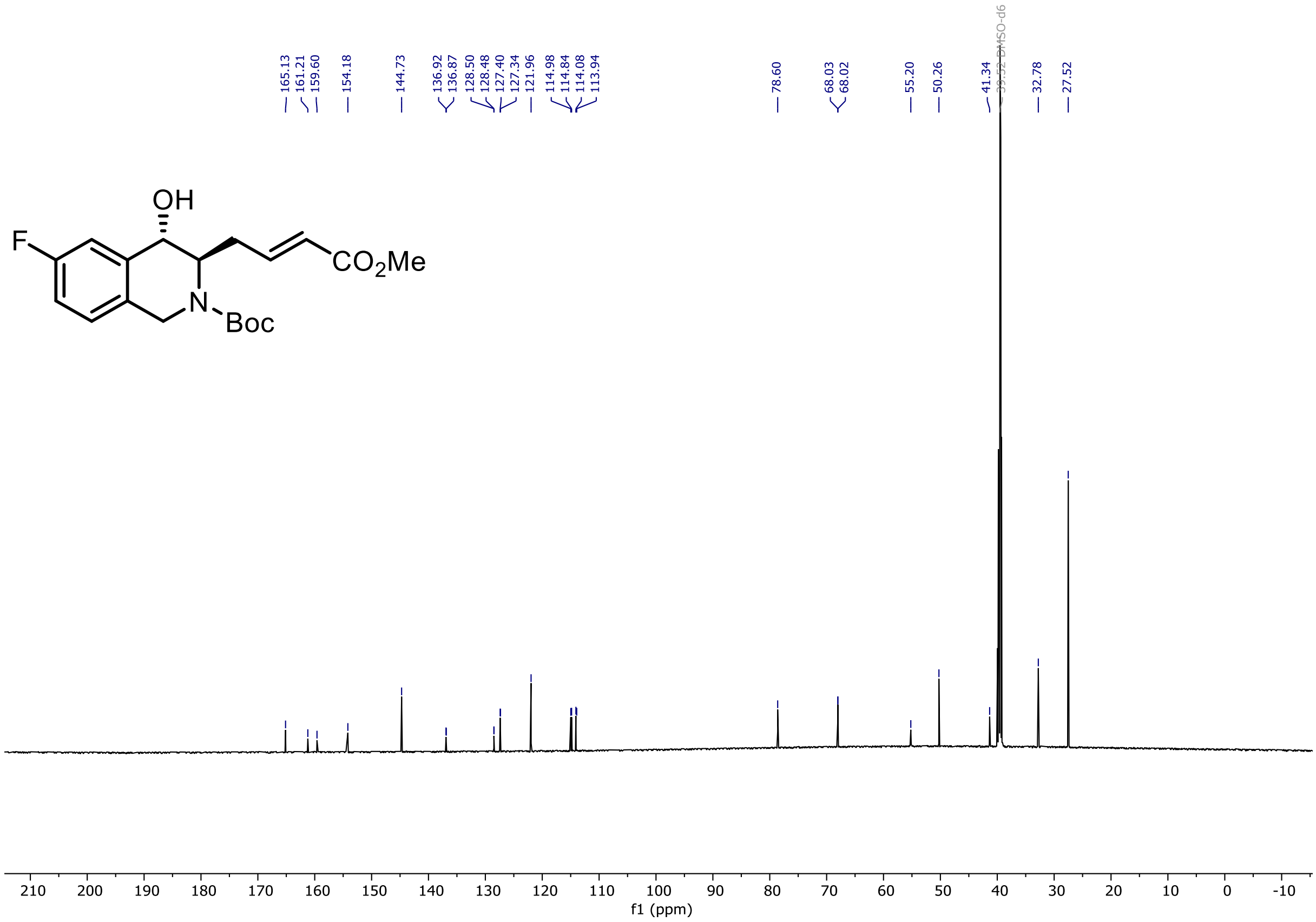

^{1}H NMR (600 MHz, [D$_6$]-DMSO, 383K; top) and ^{19}F NMR (585 MHz, [D$_6$]-DMSO, 383K; bottom) of compound **8f/8f'** (≈ 4:1)

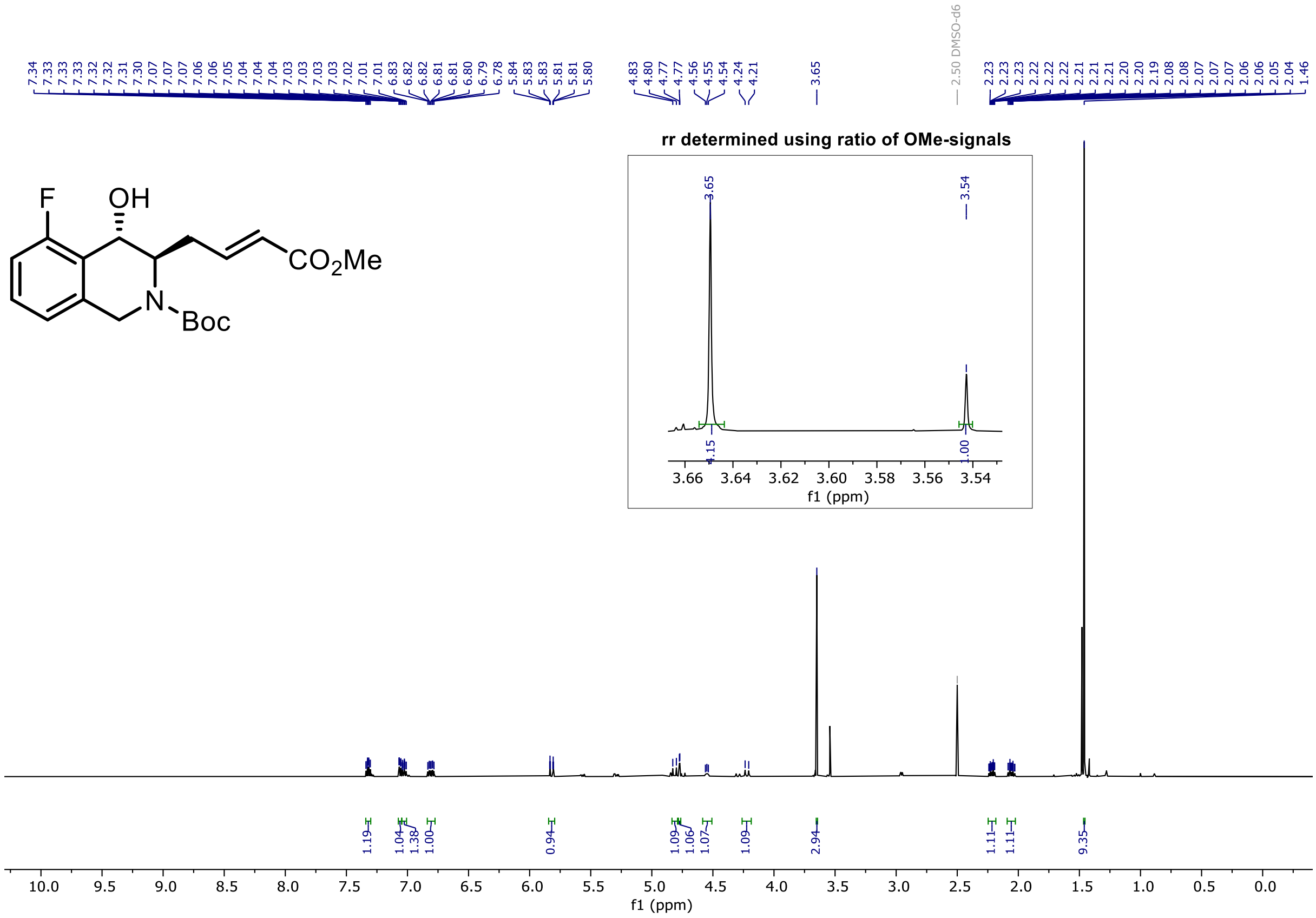

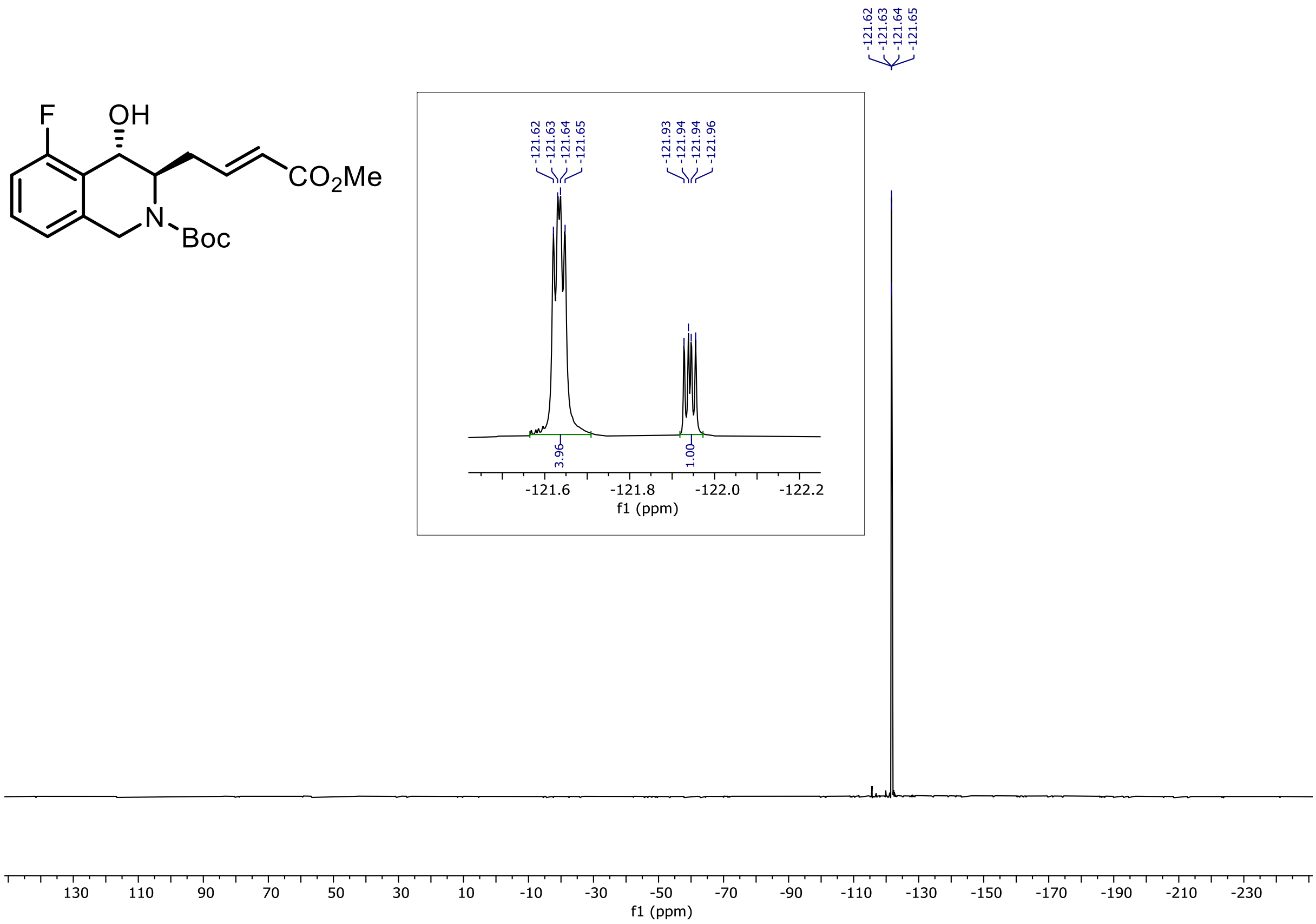

^{13}C NMR (151 MHz, [D_6]-DMSO, 383K) of compound **8f/8f'** (≈ 4:1)

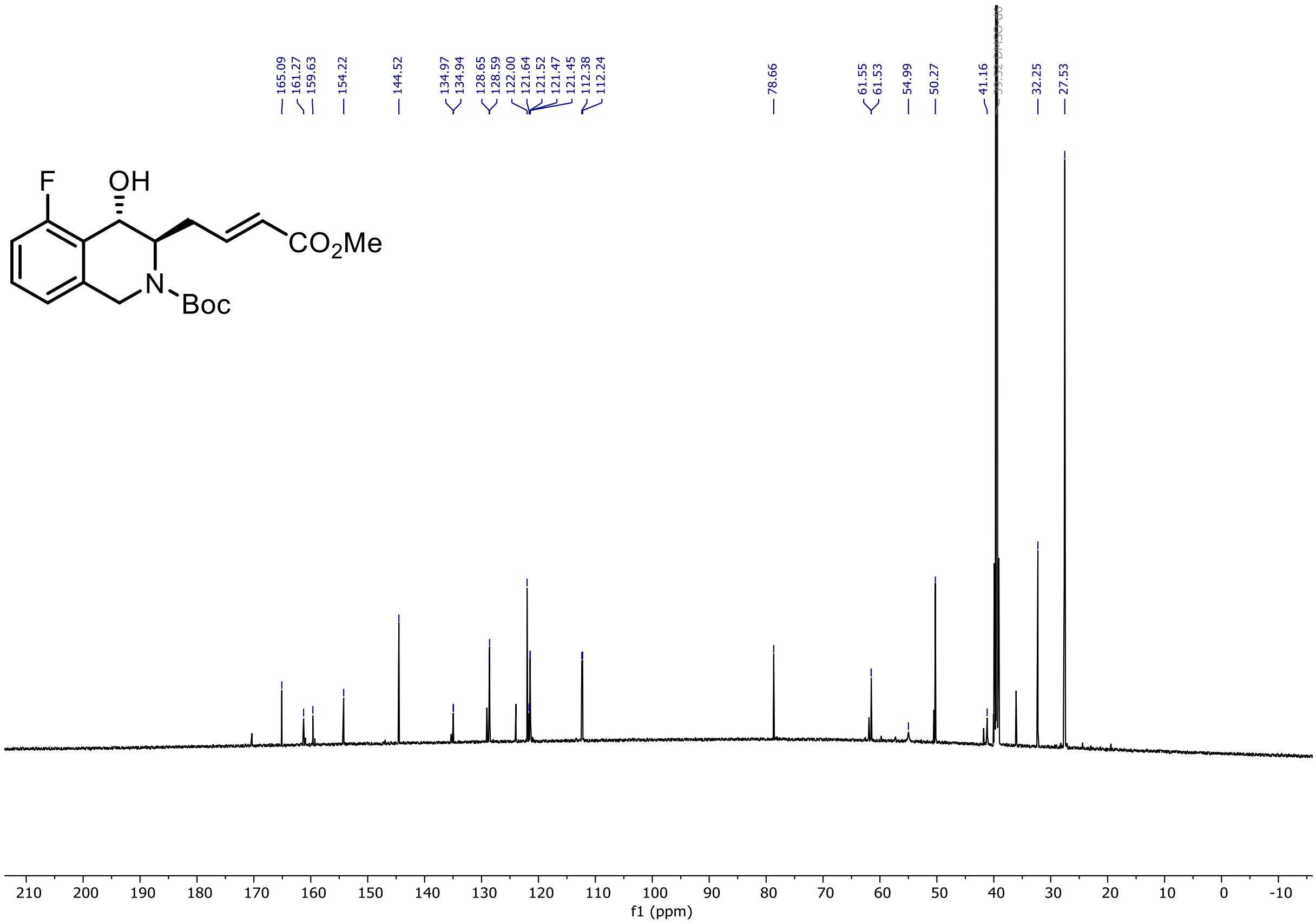

^1H NMR (600 MHz, [D$_6$]-DMSO, 383K; top) and ^{13}C NMR (151 MHz, [D$_6$]-DMSO, 383K; bottom) of compound **8g**

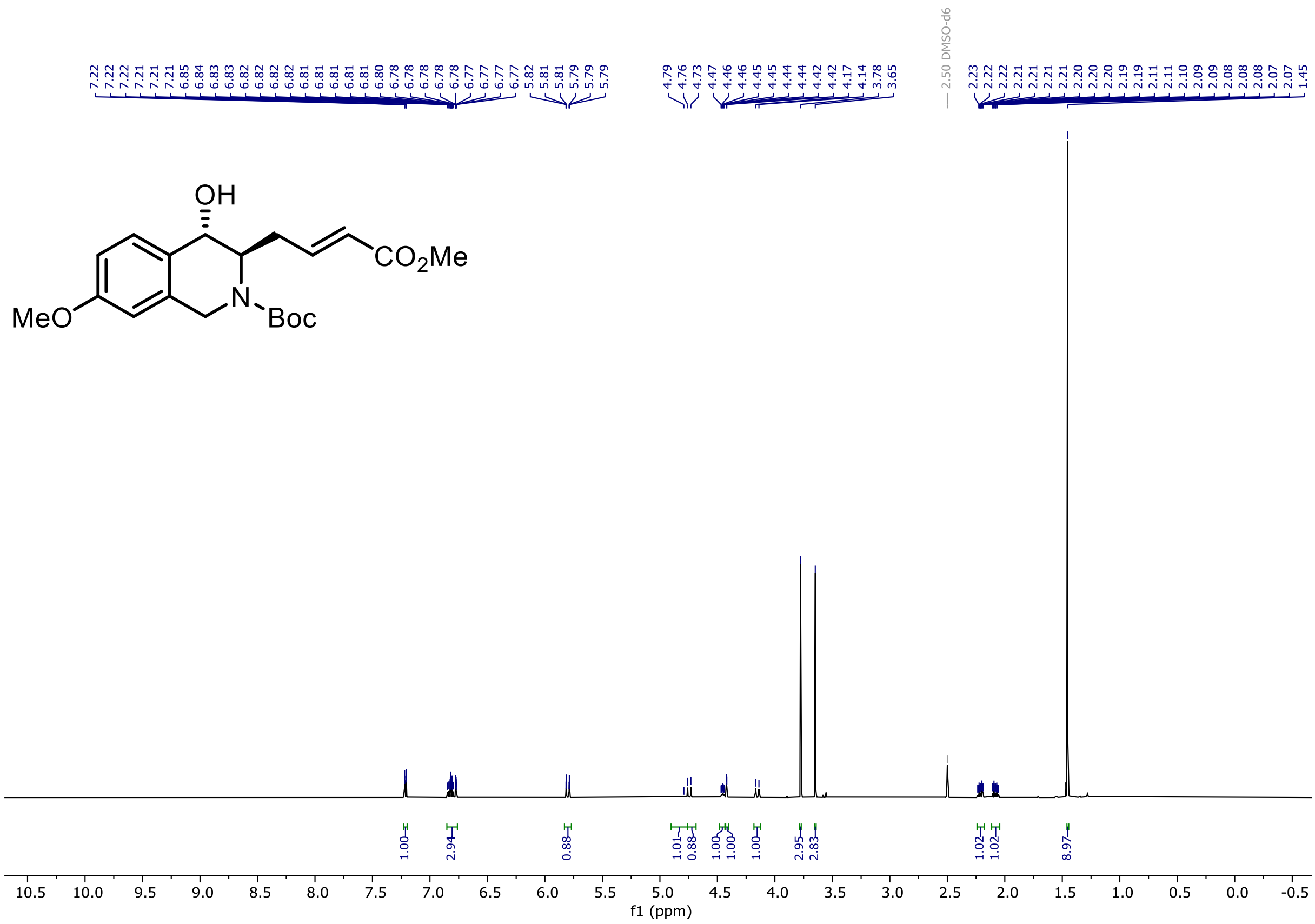

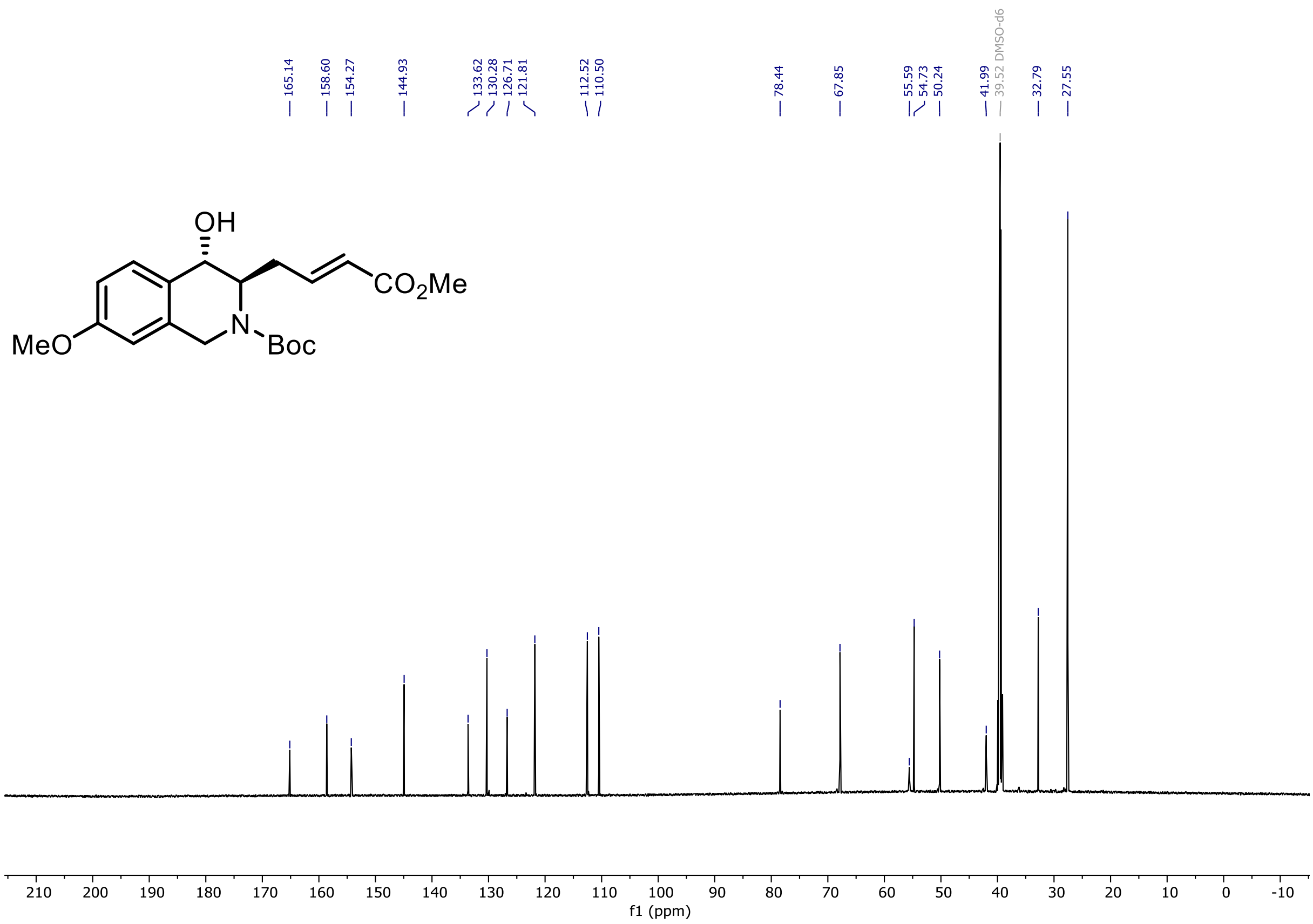

^{1}H NMR (600 MHz, [D$_6$]-DMSO, 383K; top) and ^{13}C NMR (151 MHz, [D$_6$]-DMSO, 383K; bottom) of compound **8h**

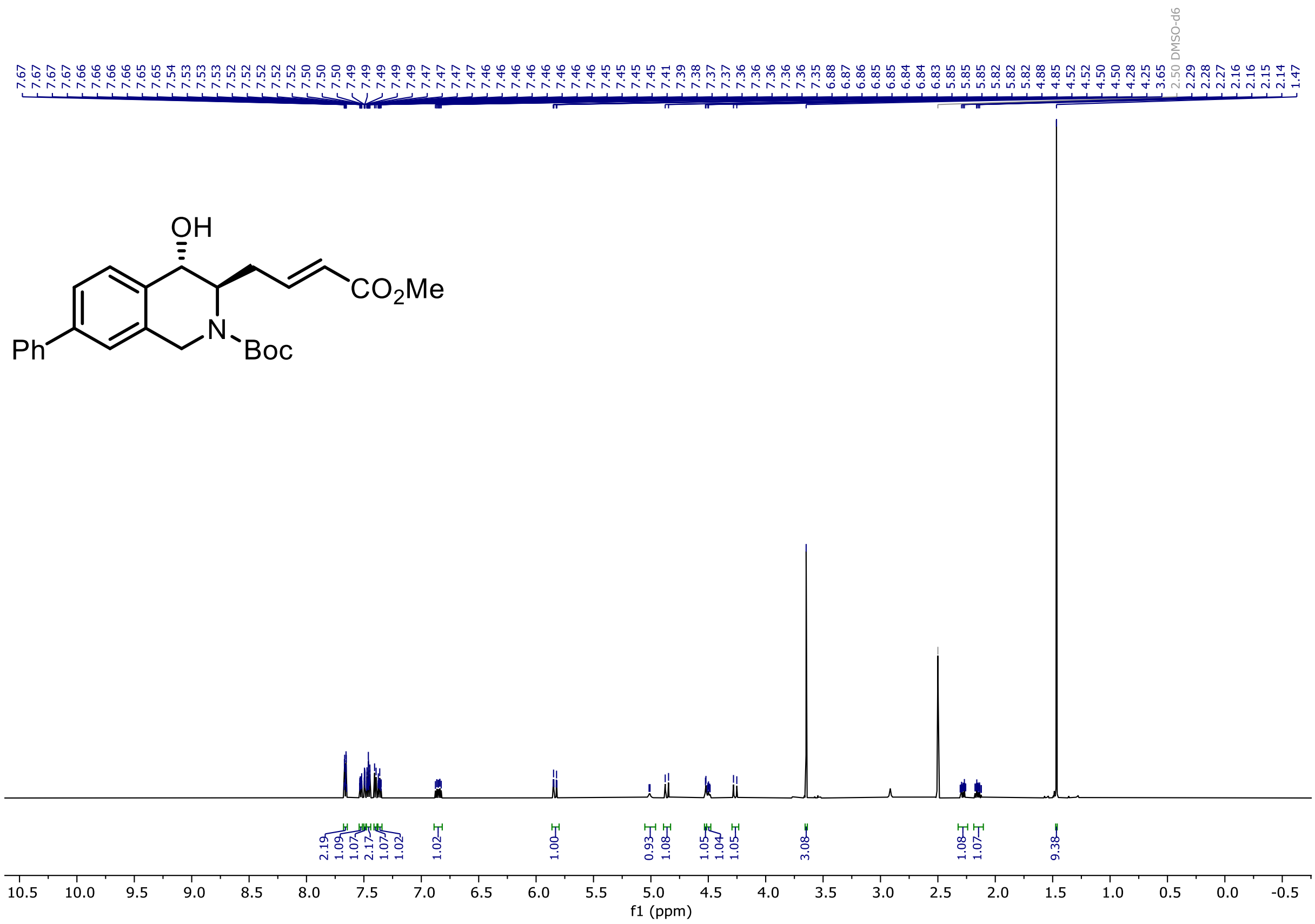

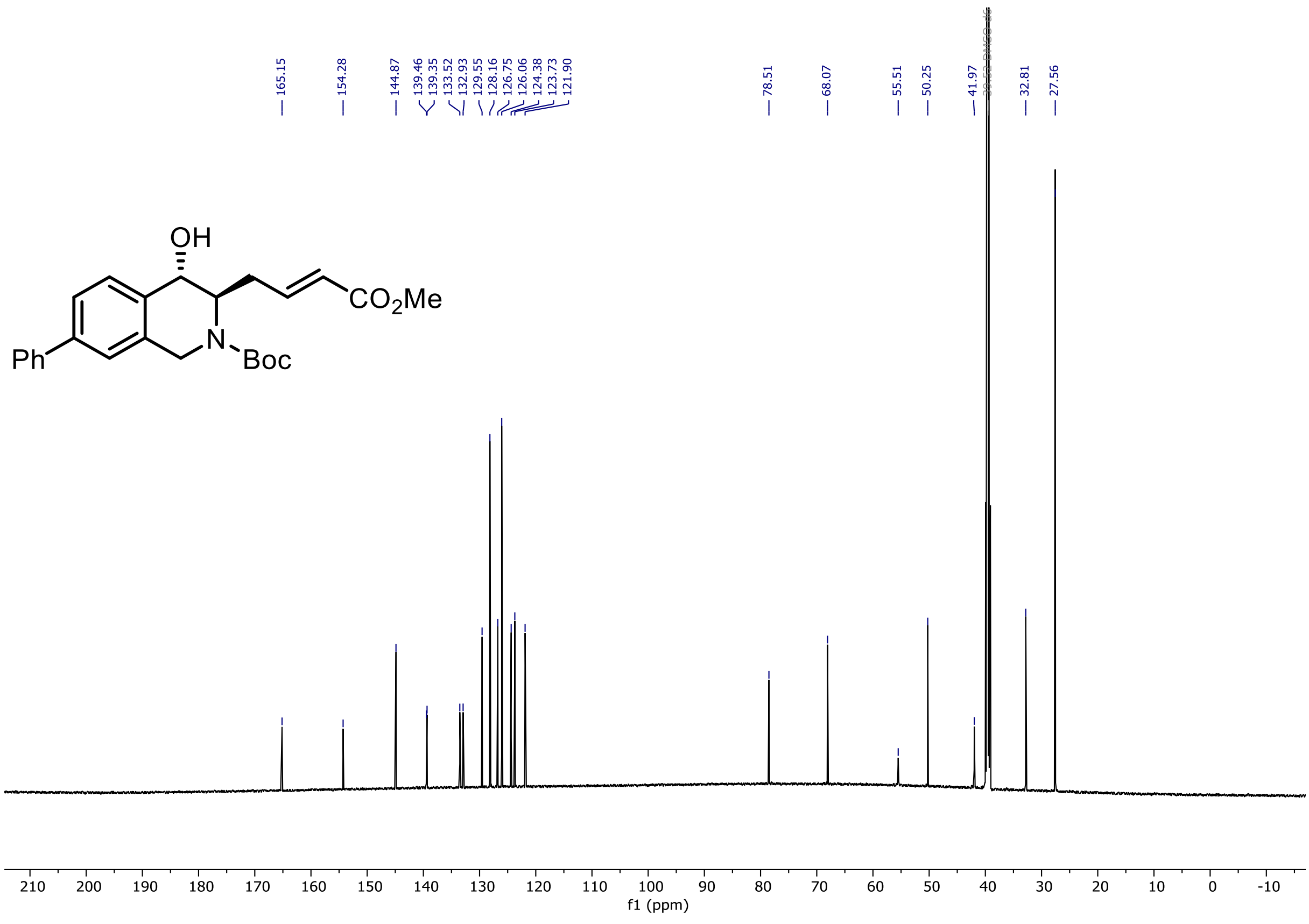

^{1}H NMR (600 MHz, [D$_6$]-DMSO, 383K; top) and ^{13}C NMR (151 MHz, [D$_6$]-DMSO, 383K; bottom) of compound **8i**

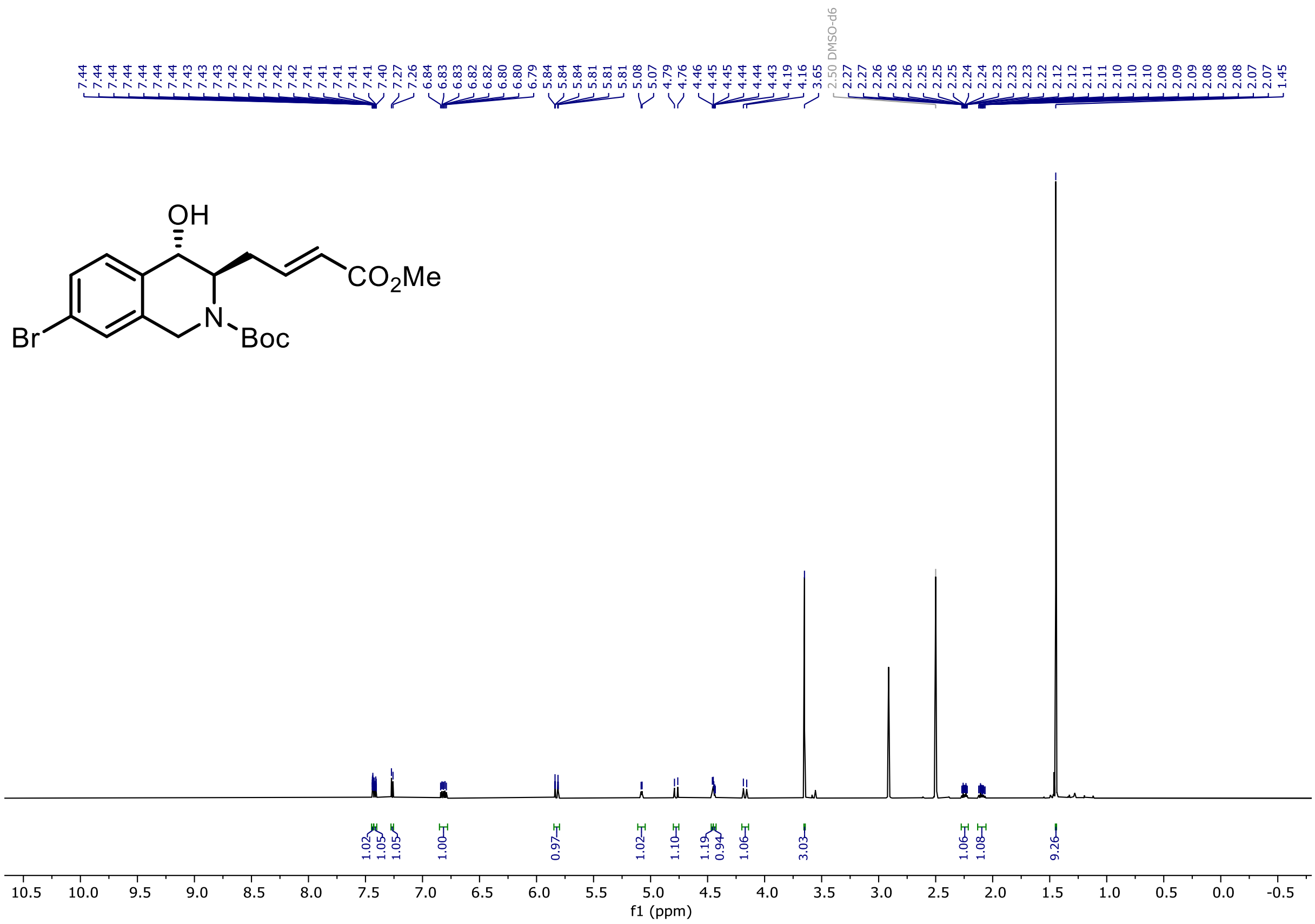

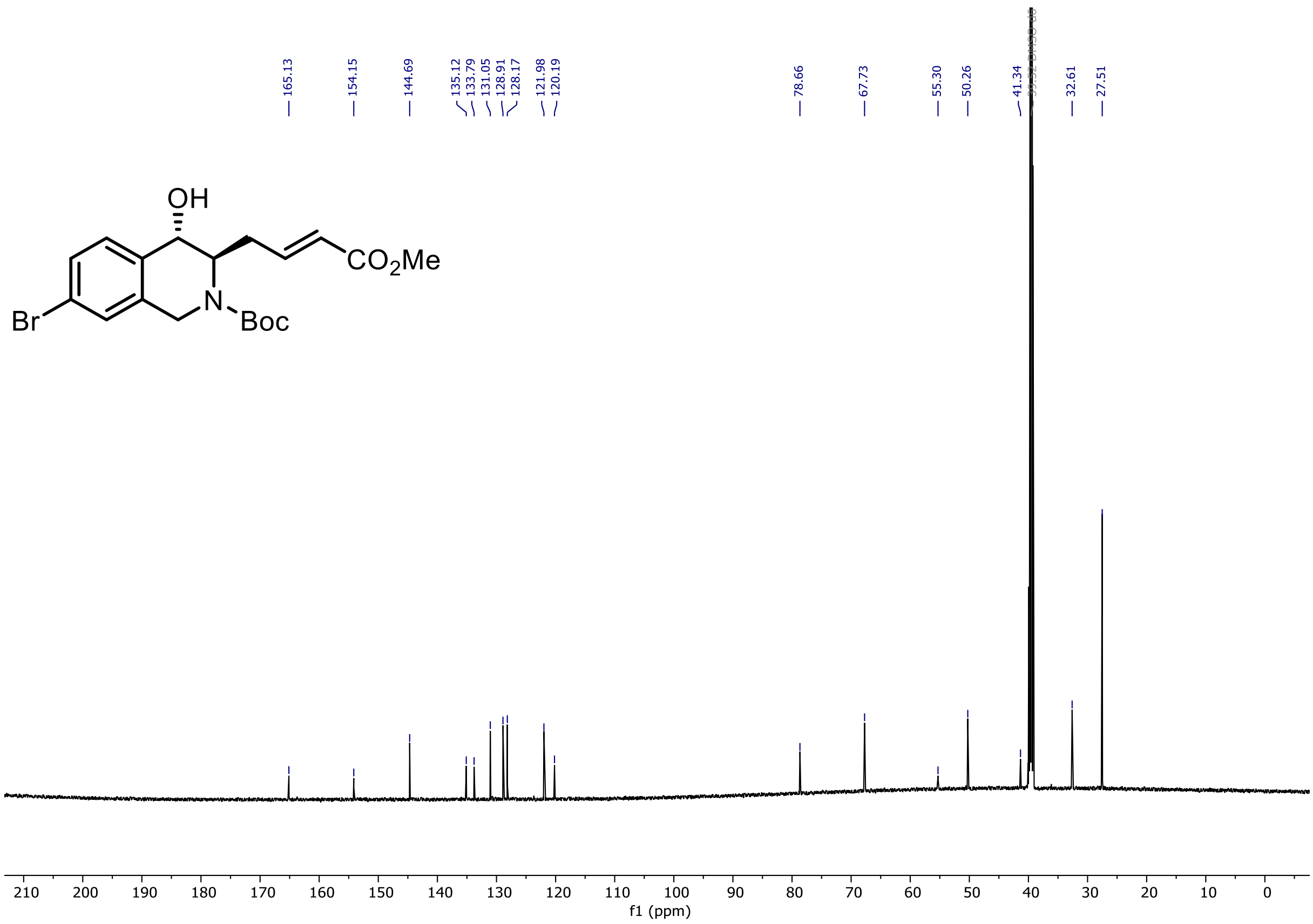

^1H NMR (600 MHz, [D$_6$]-DMSO, 383K; top) and ^{13}C NMR (151 MHz, [D$_6$]-DMSO, 383K; bottom) of compound **8j**

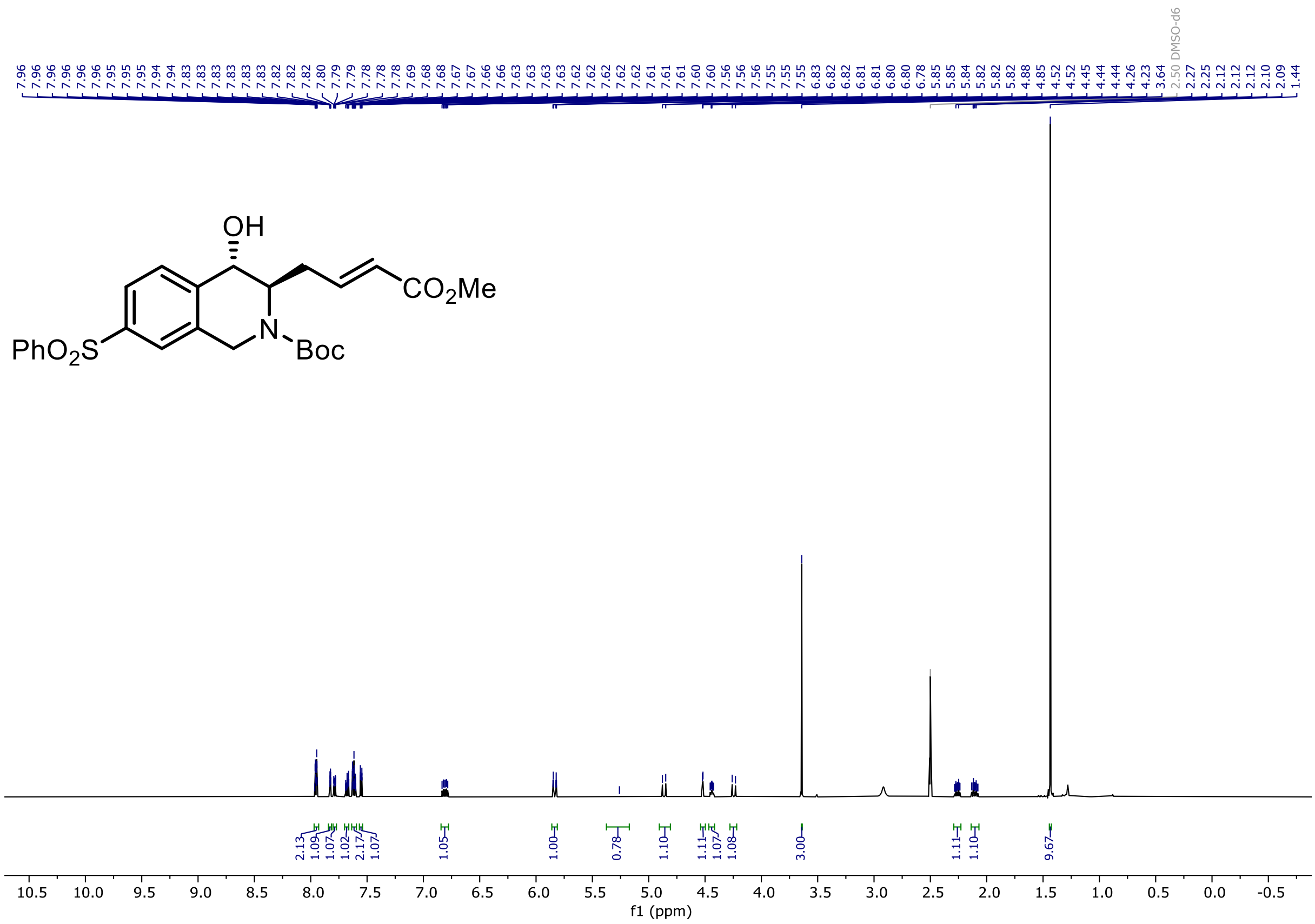

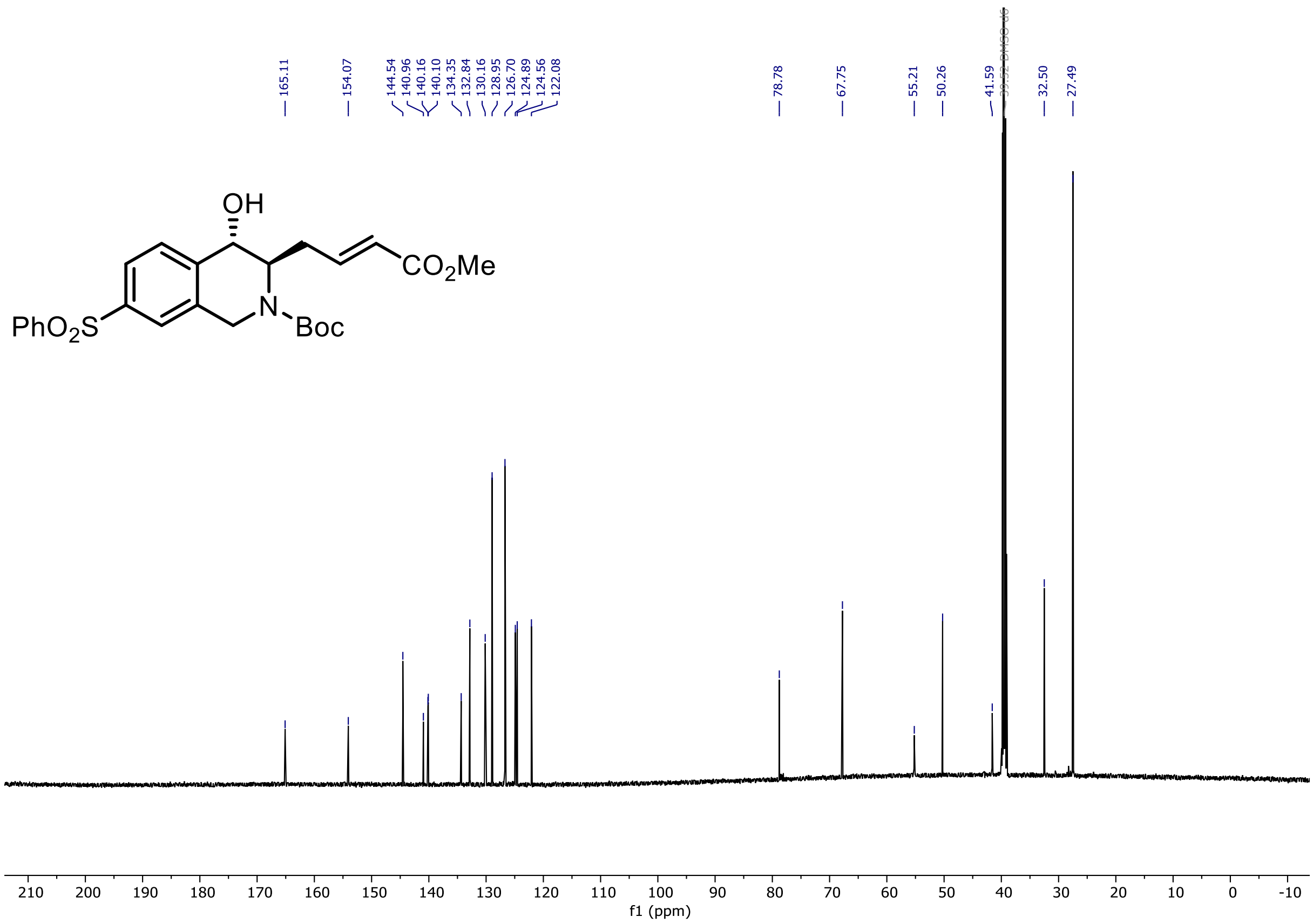

¹H NMR (600 MHz, [D$_6$]-DMSO, 383K; top) and ^{13}C NMR (151 MHz, [D$_6$]-DMSO, 383K; bottom) of compound **8k**

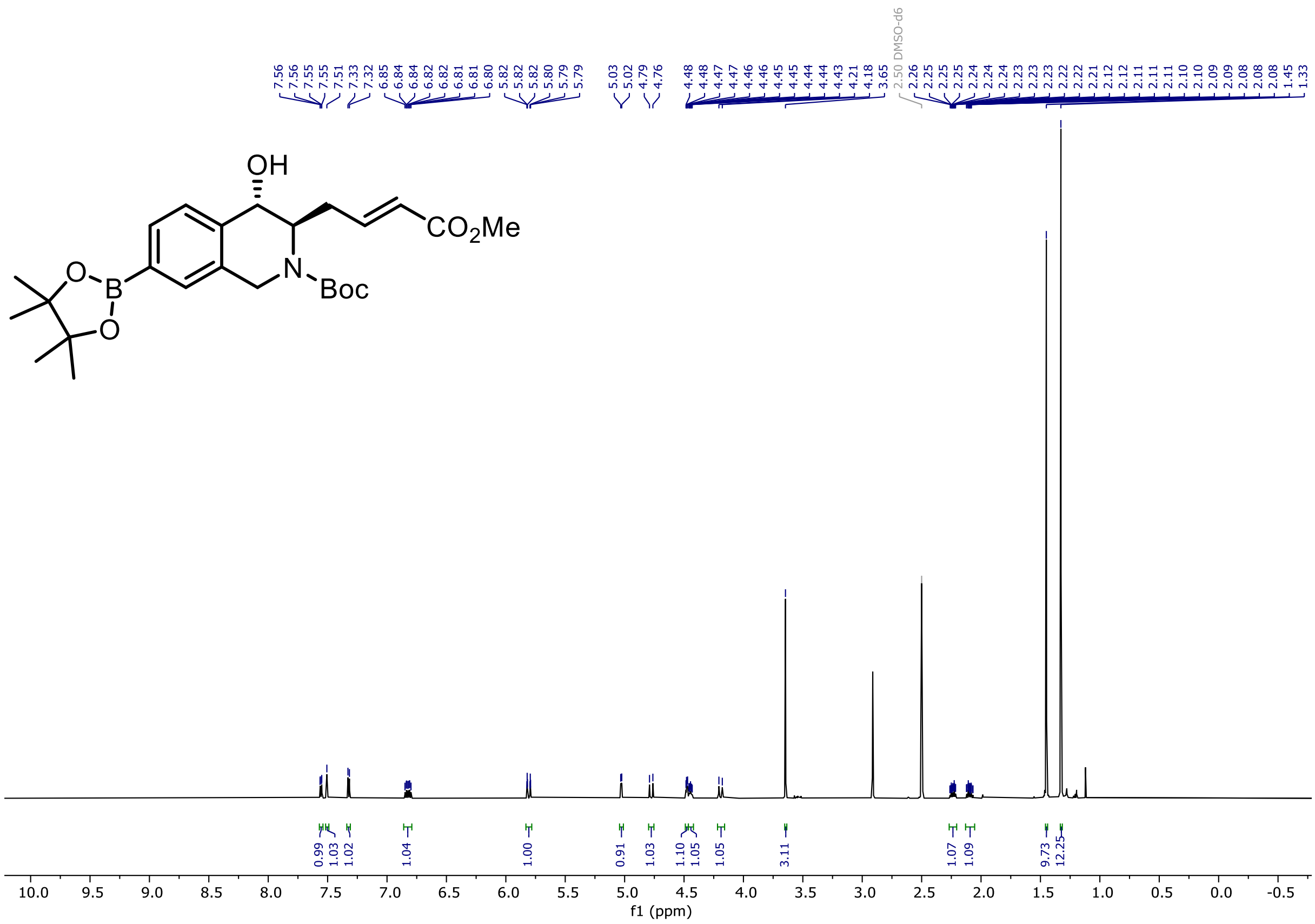

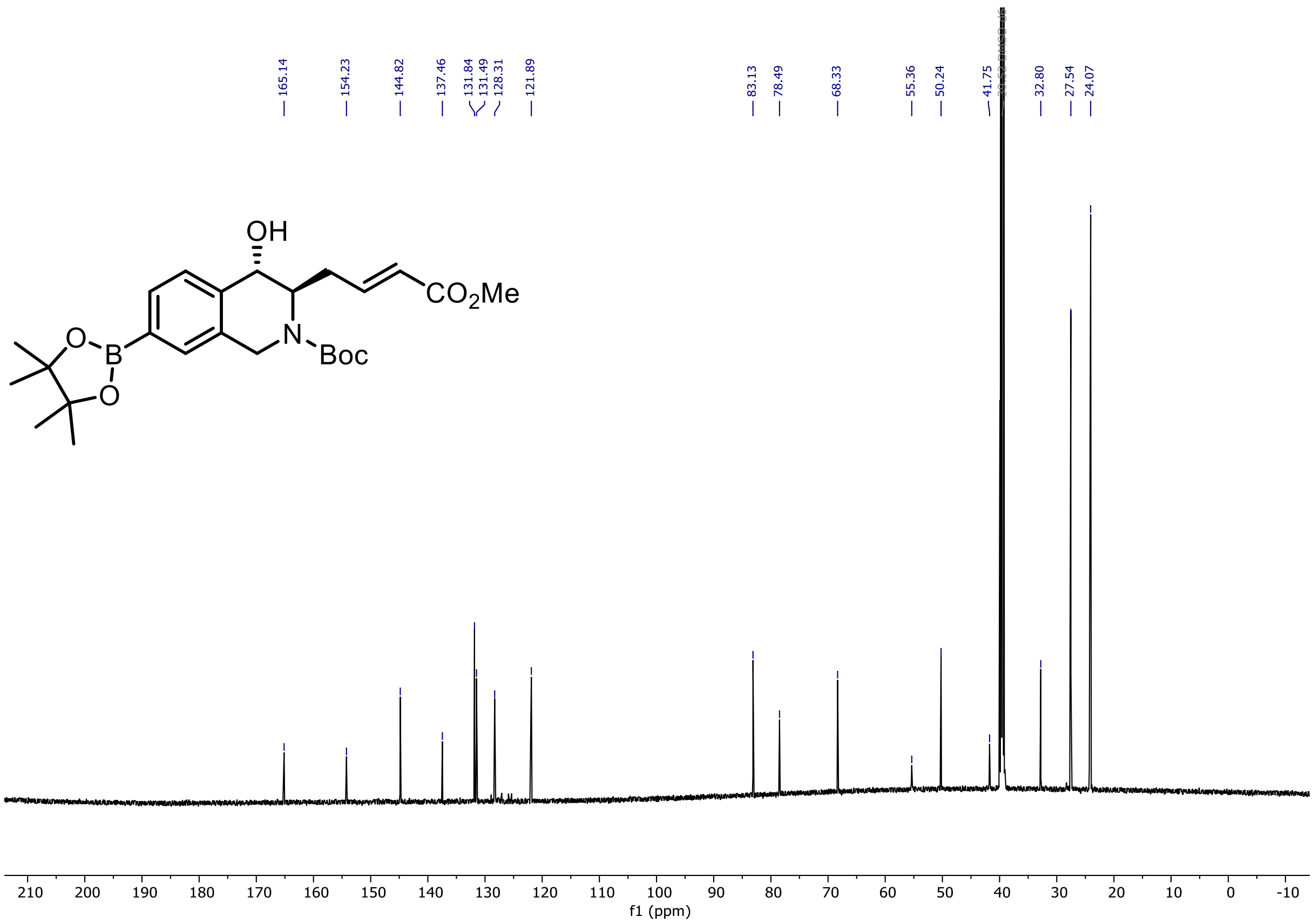

^1H NMR (600 MHz, [D$_6$]-DMSO, 383K; top) and ^{13}C NMR (151 MHz, [D$_6$]-DMSO, 383K; bottom) of compound **8l**

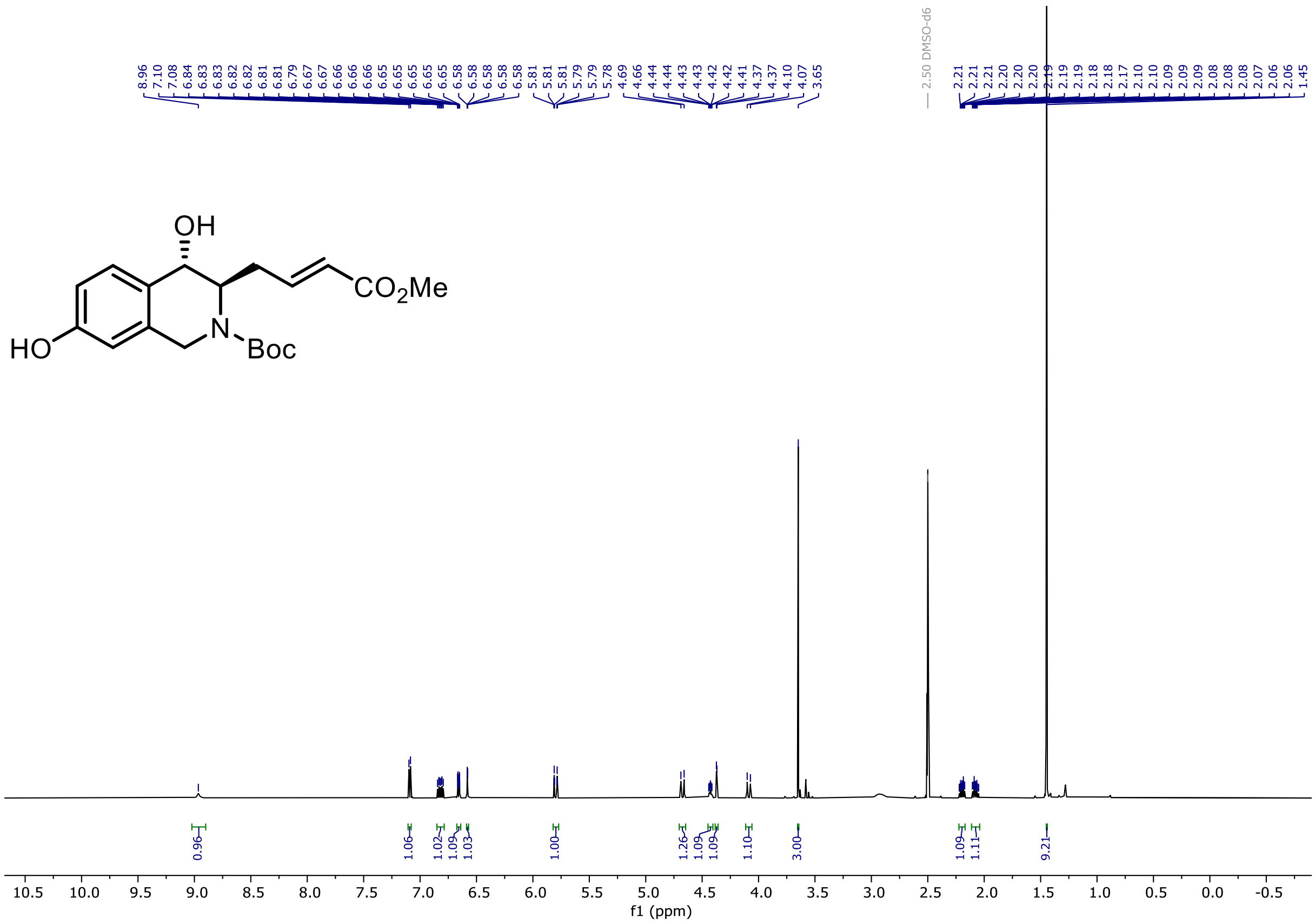

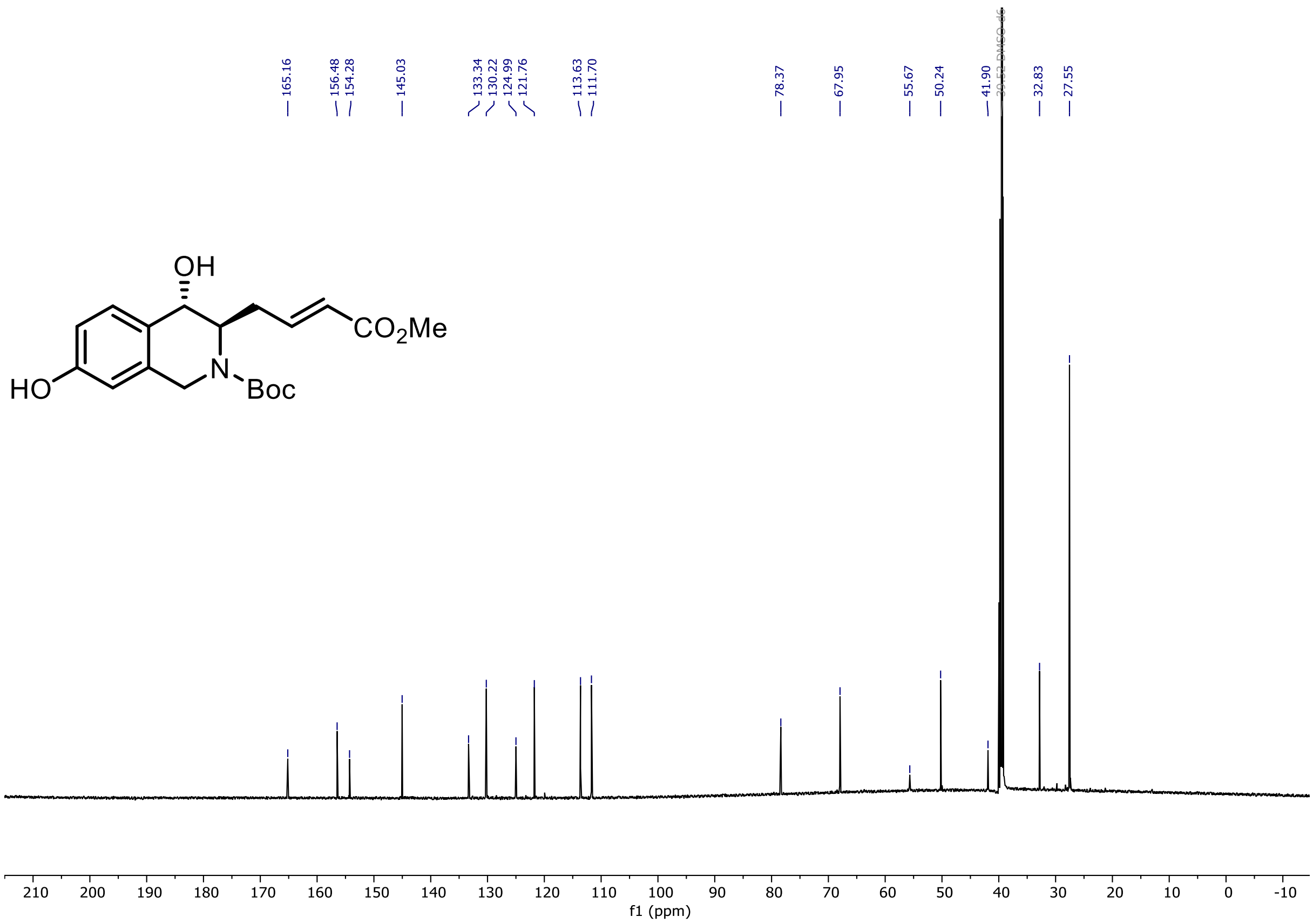

^{1}H NMR (600 MHz, [D_6]-DMSO, 383K; top) and ^{13}C NMR (151 MHz, [D_6]-DMSO, 383K; bottom) of compound **8m**

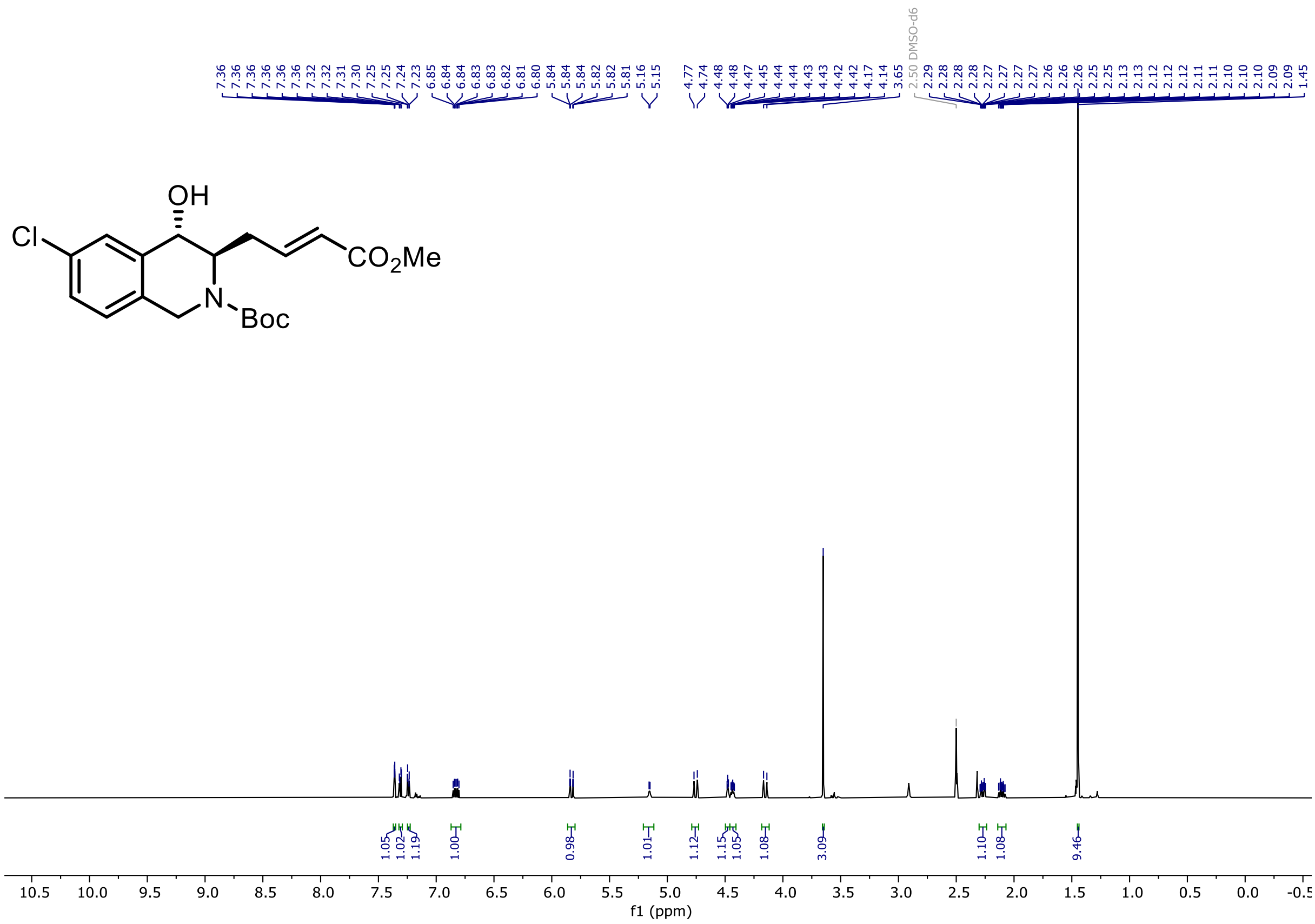

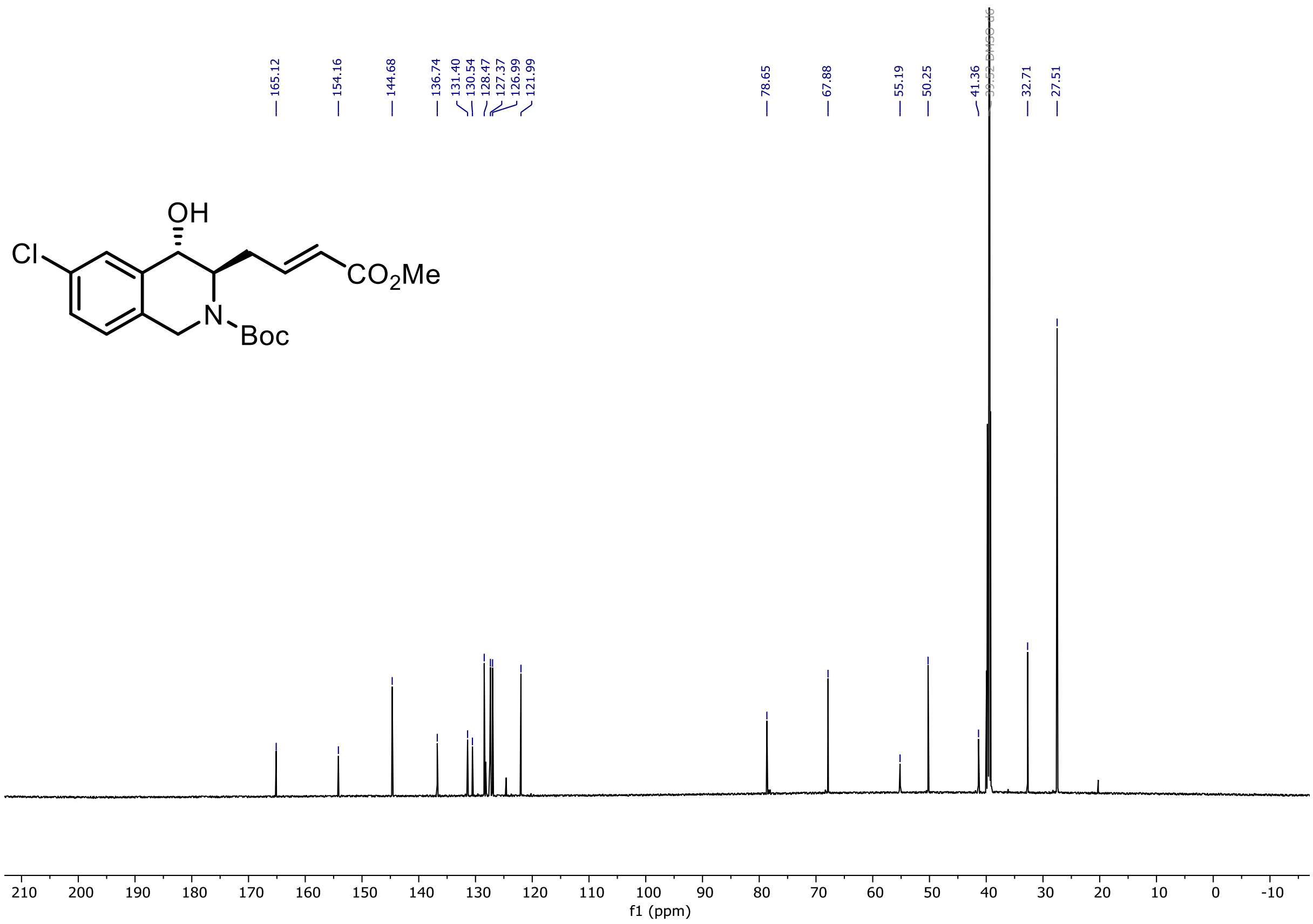

^{1}H NMR (600 MHz, [D_6]-DMSO, 383K; top) and ^{19}F NMR (585 MHz, [D_6]-DMSO, 383K; bottom) of compound **8n**

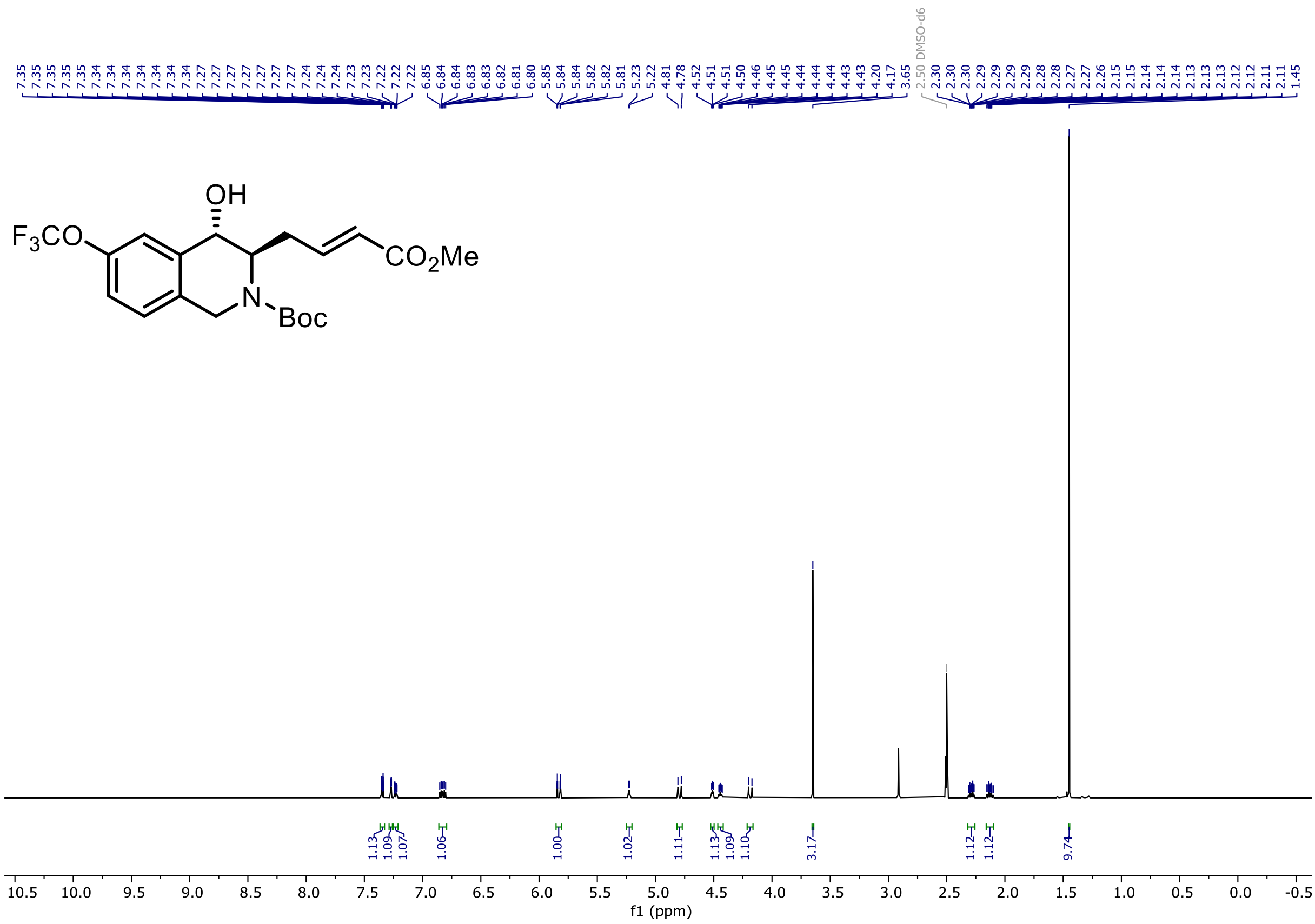

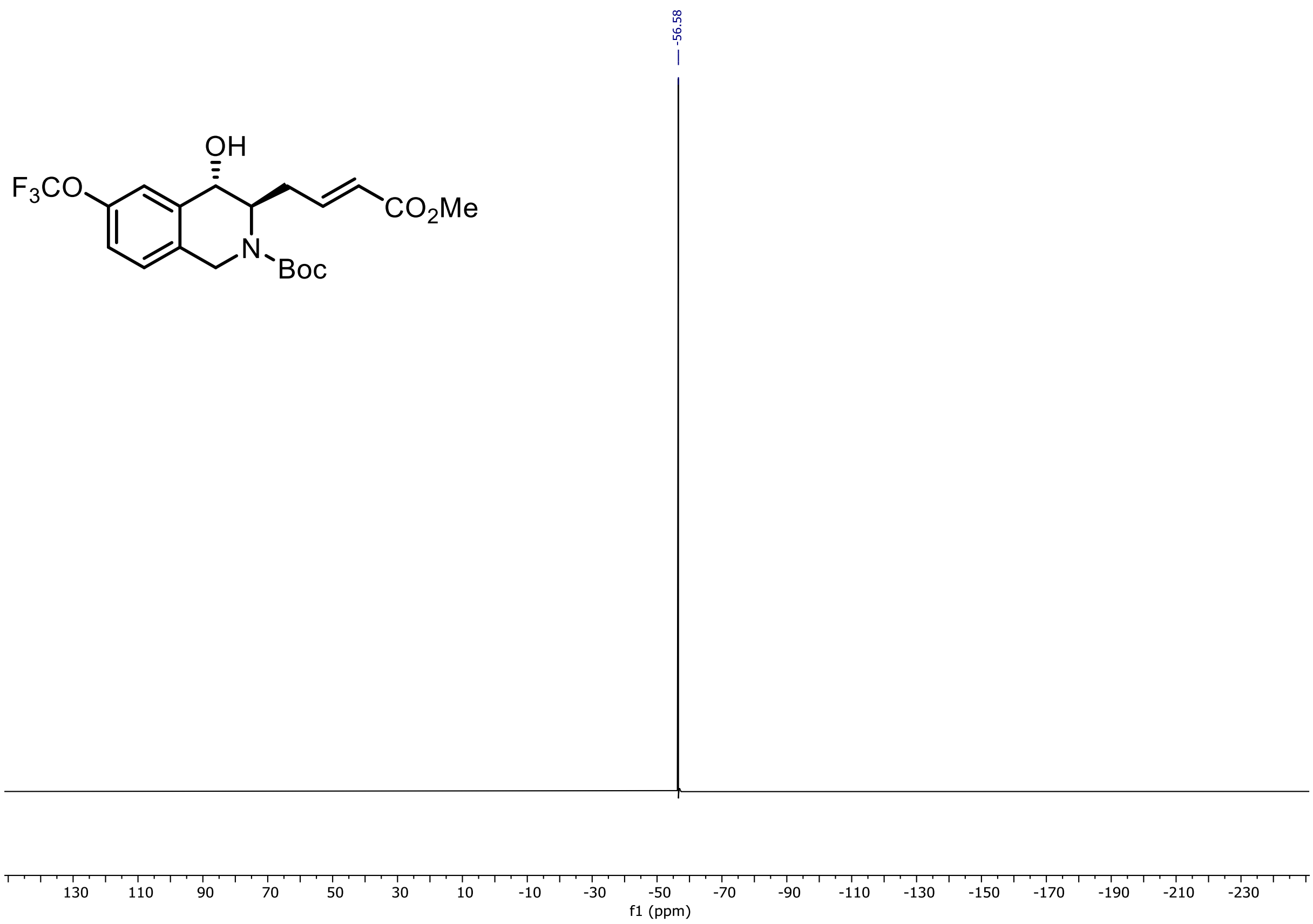

^{13}C NMR (151 MHz, [D_6]-DMSO, 383K) of compound **8n**

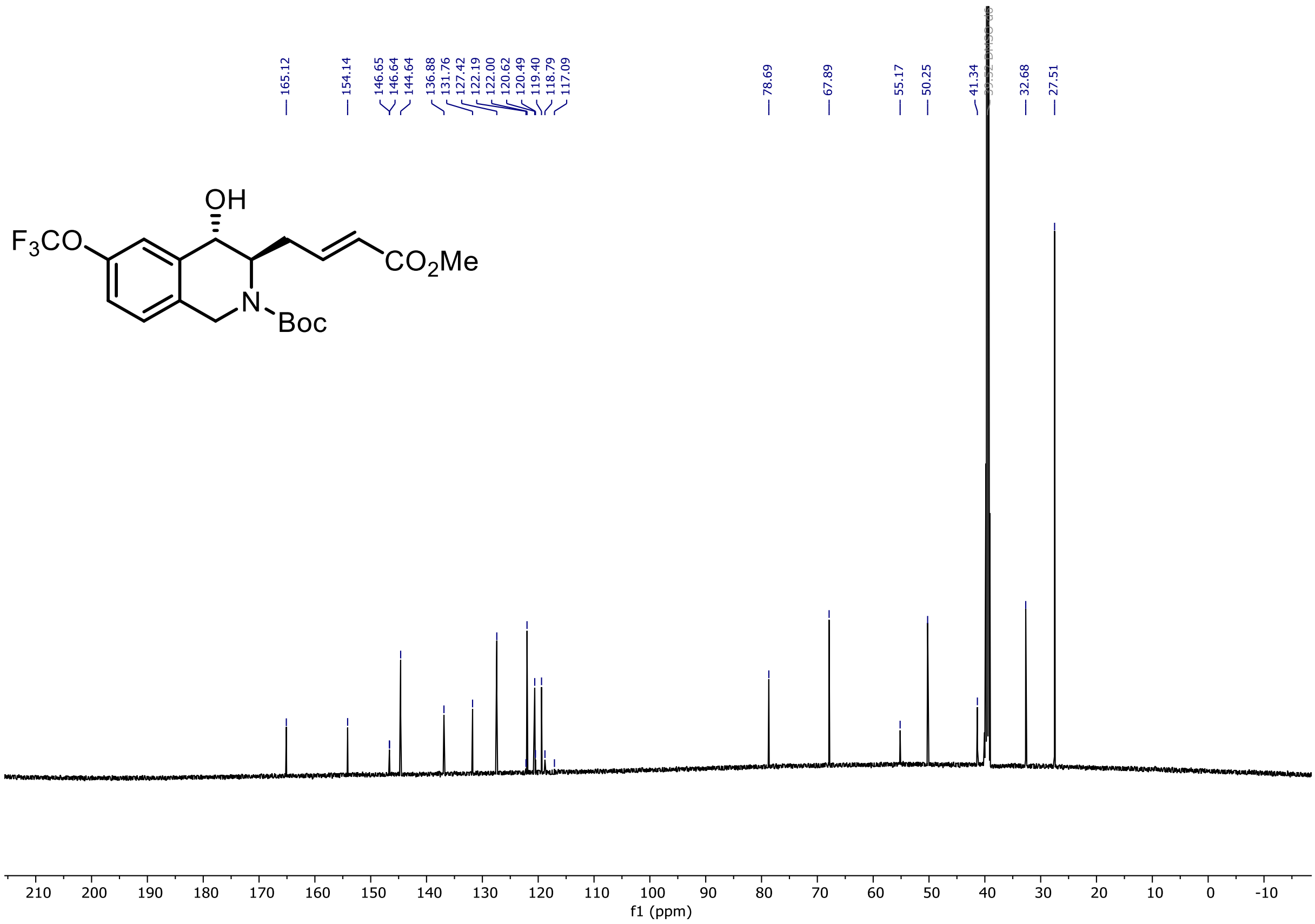

^{1}H NMR (600 MHz, [D$_6$]-DMSO, 383K; top) and ^{13}C NMR (151 MHz, [D$_6$]-DMSO, 383K; bottom) of compound **9**

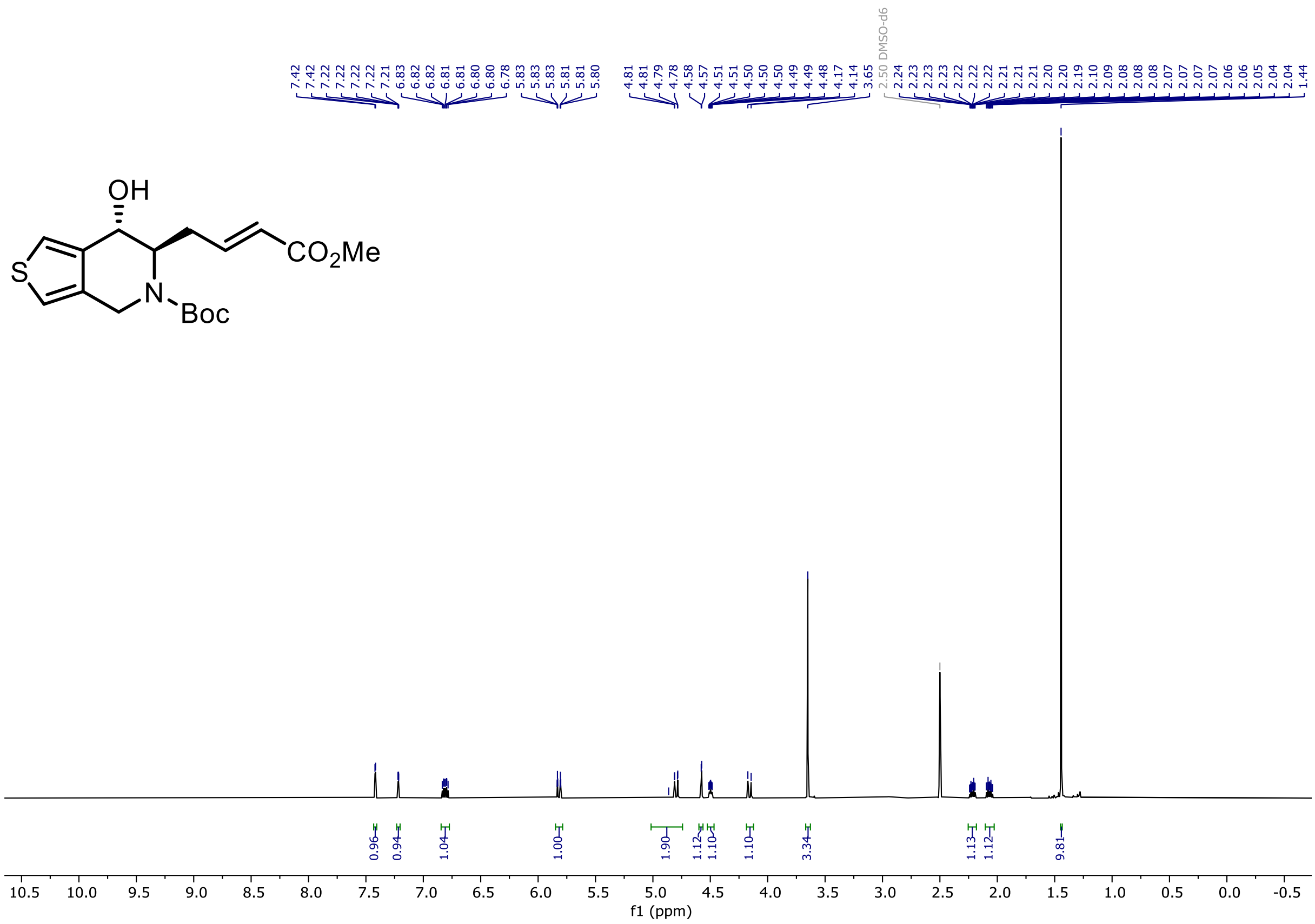

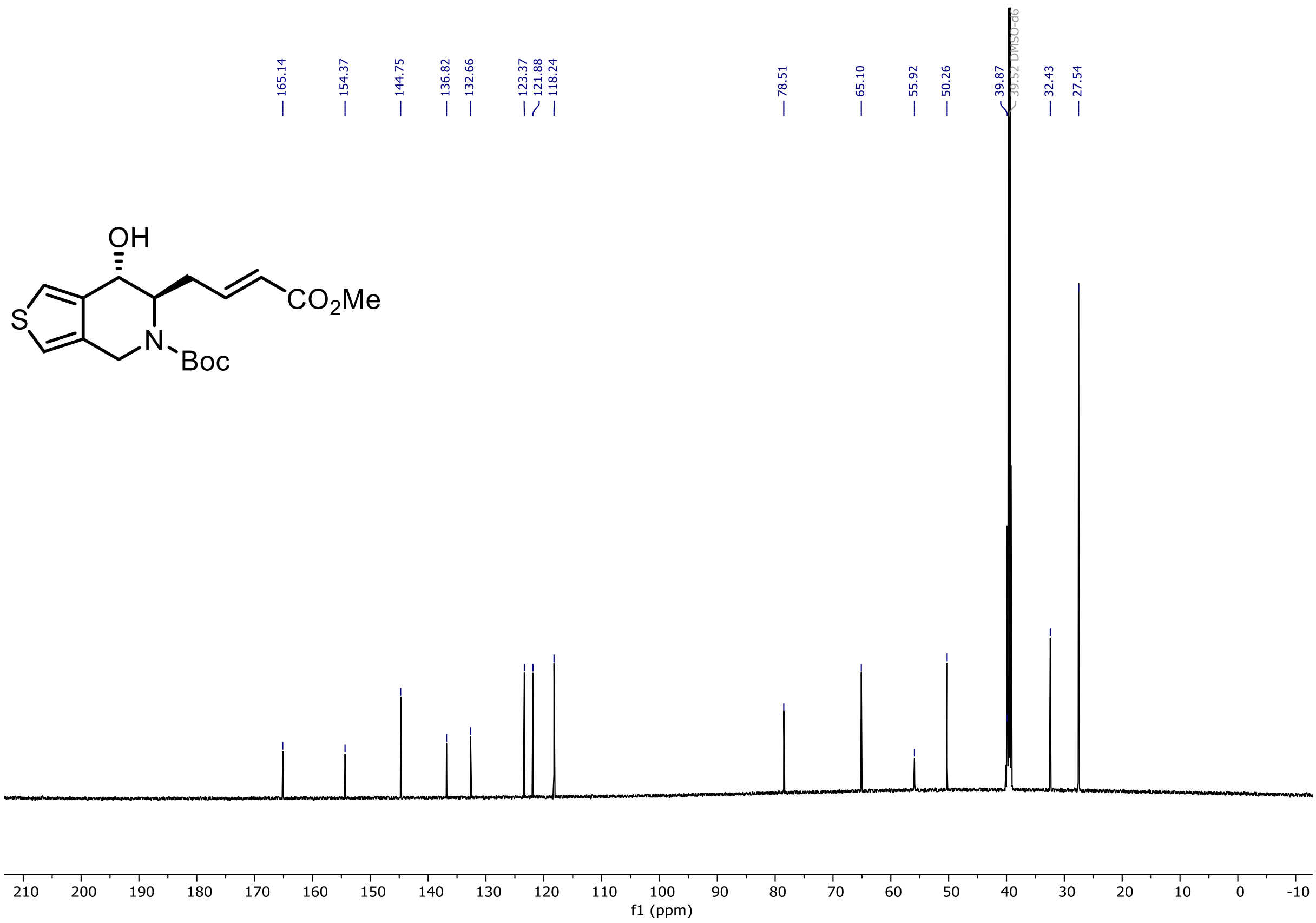

^1H NMR (600 MHz, [D$_6$]-DMSO, 383K; top) and ^{13}C NMR (151 MHz, [D$_6$]-DMSO, 383K; bottom) of compound **10**

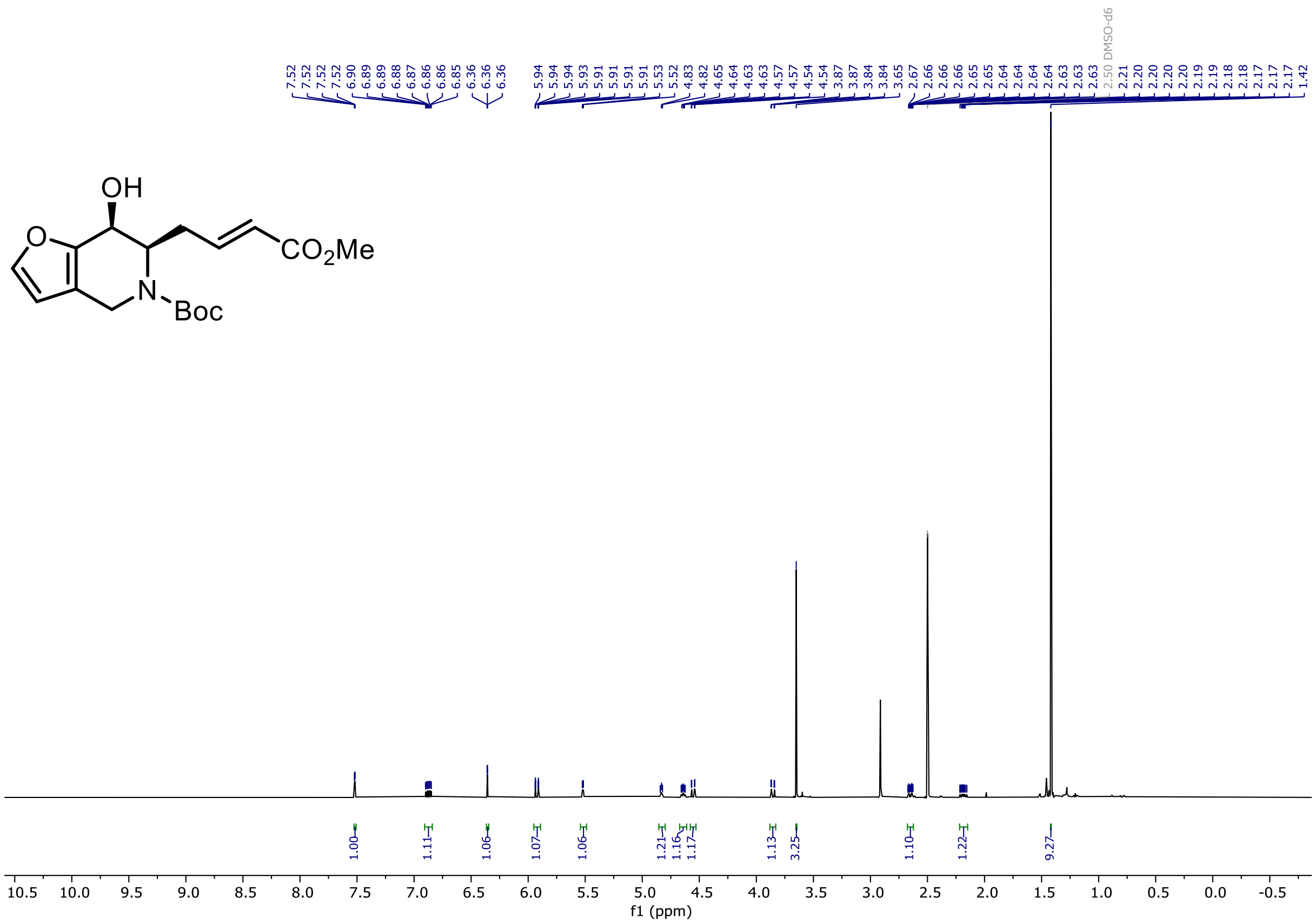

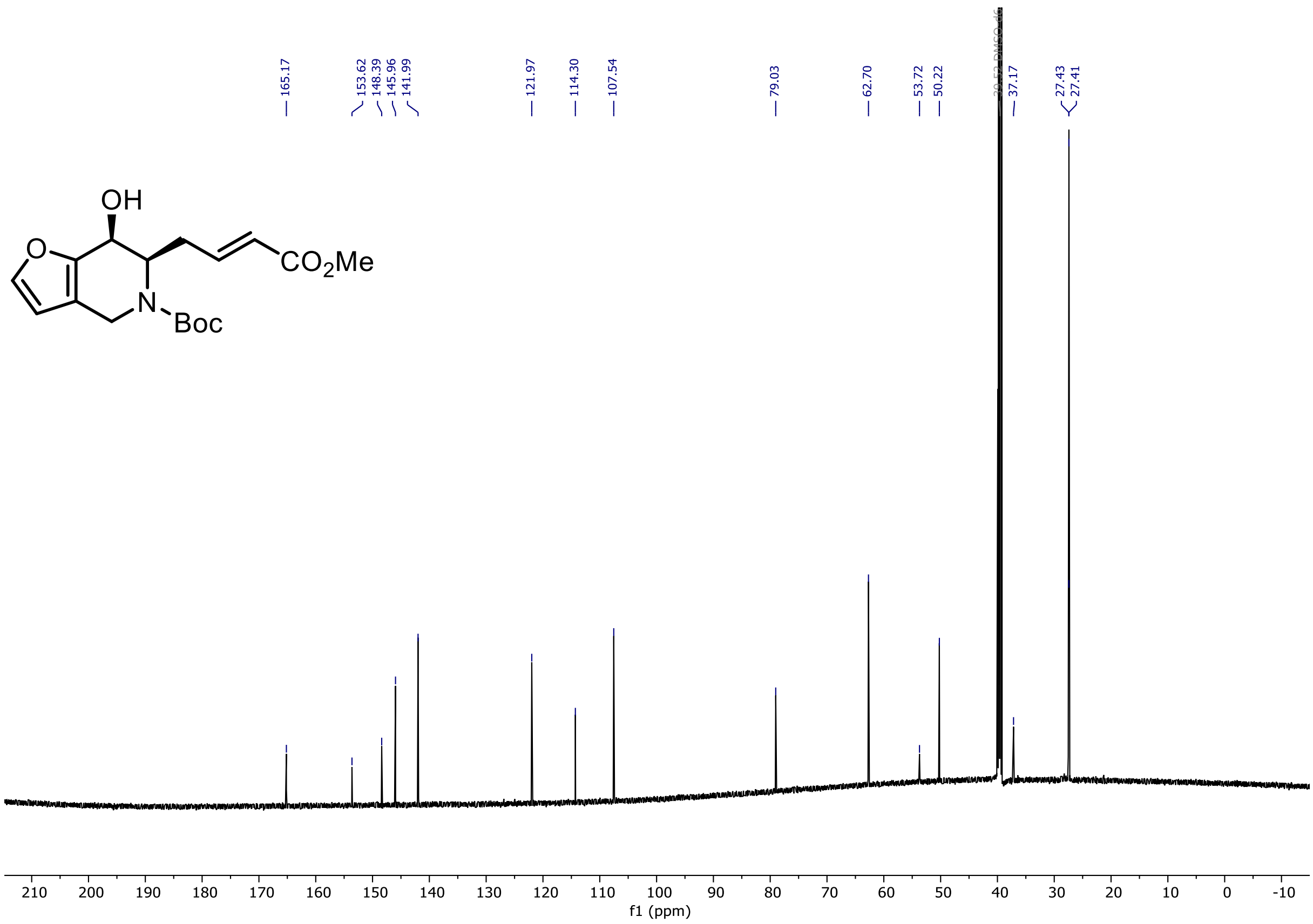

^{1}H NMR (600 MHz, [D₆]-DMSO, 383K; top) and ^{13}C NMR (151 MHz, [D₆]-DMSO, 383K; bottom) of compound **11**

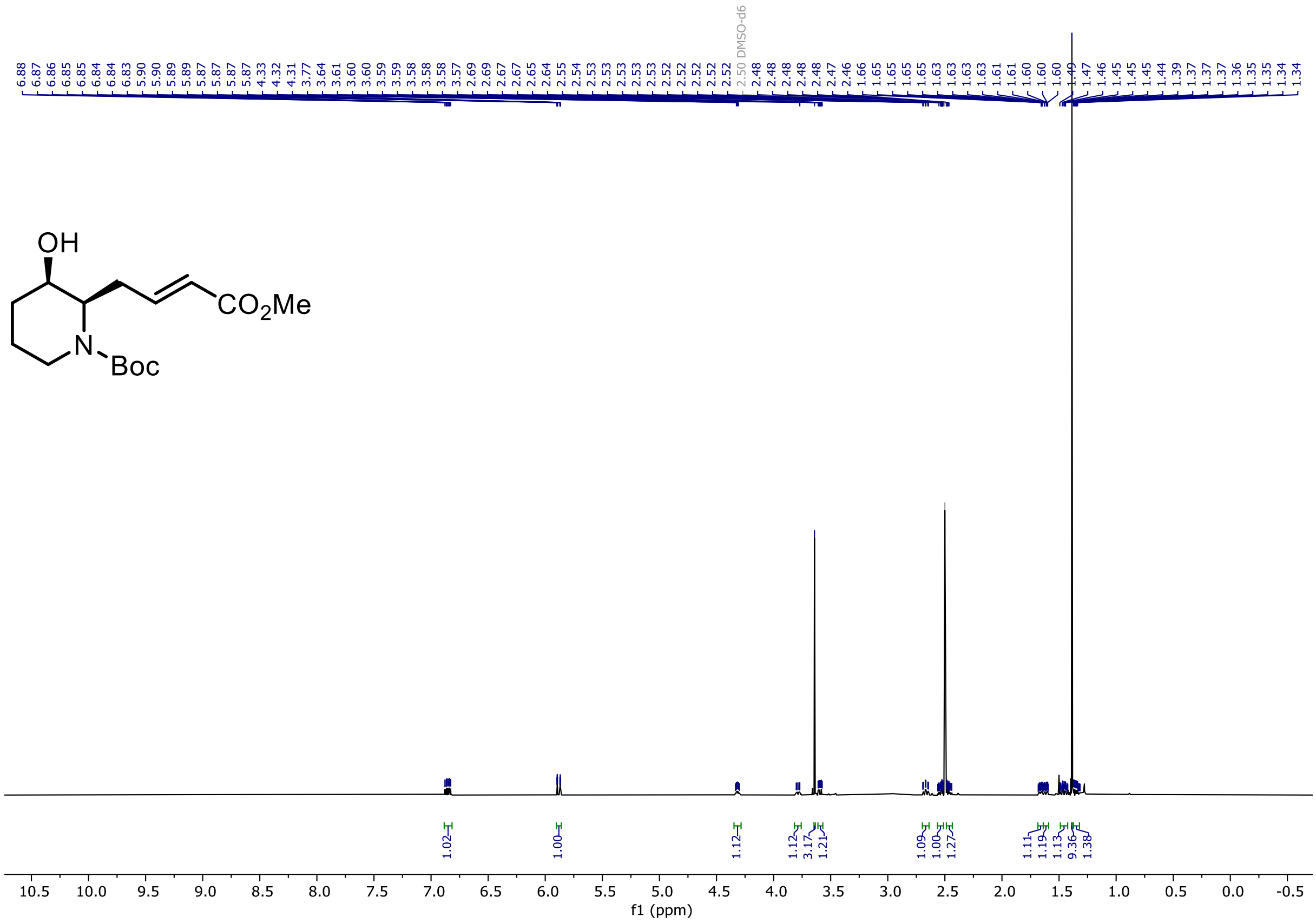

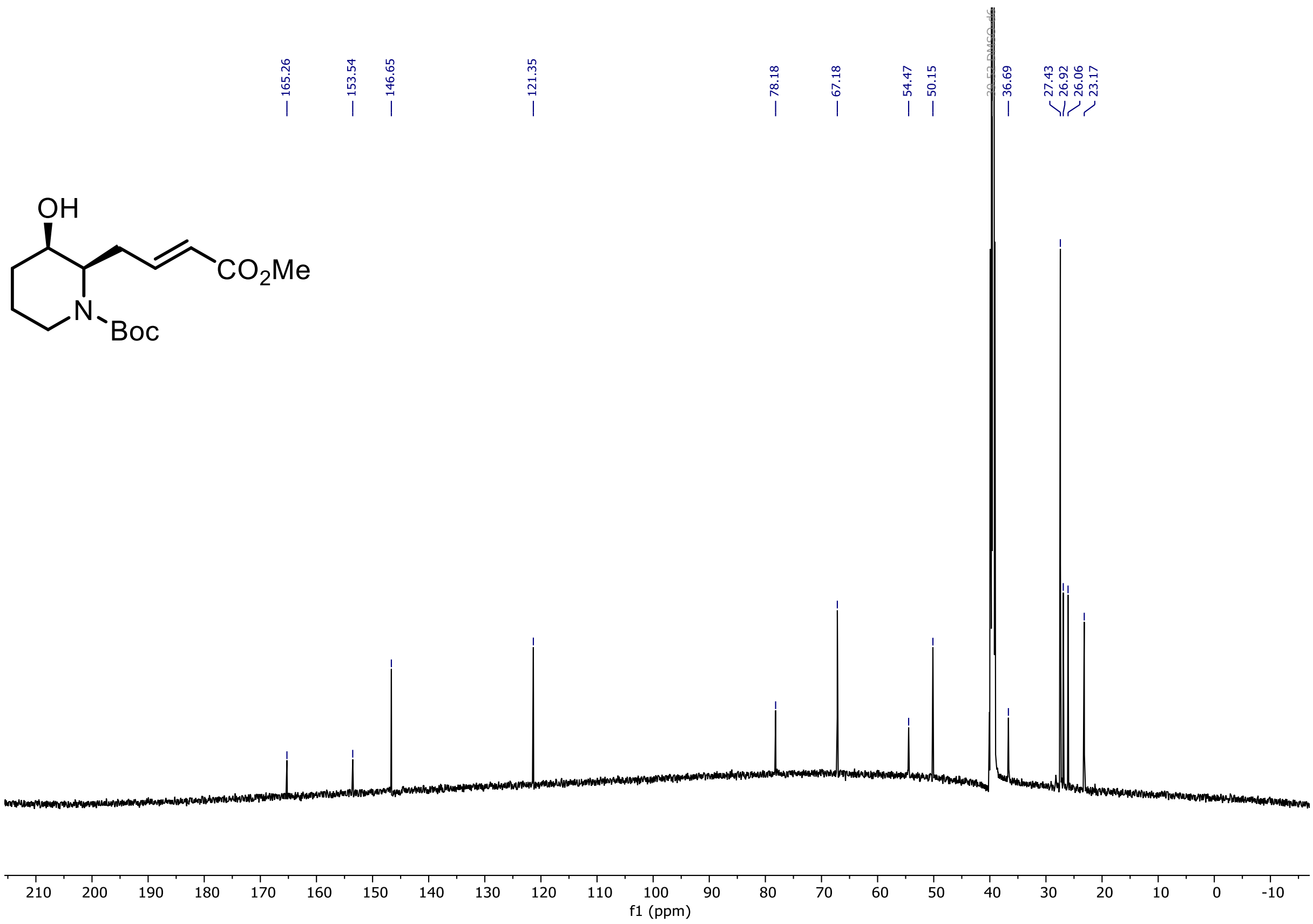

^{1}H NMR (600 MHz, [D$_6$]-DMSO, 373K; top) and ^{13}C NMR (151 MHz, [D$_6$]-DMSO, 373K; bottom) of compound **12a**

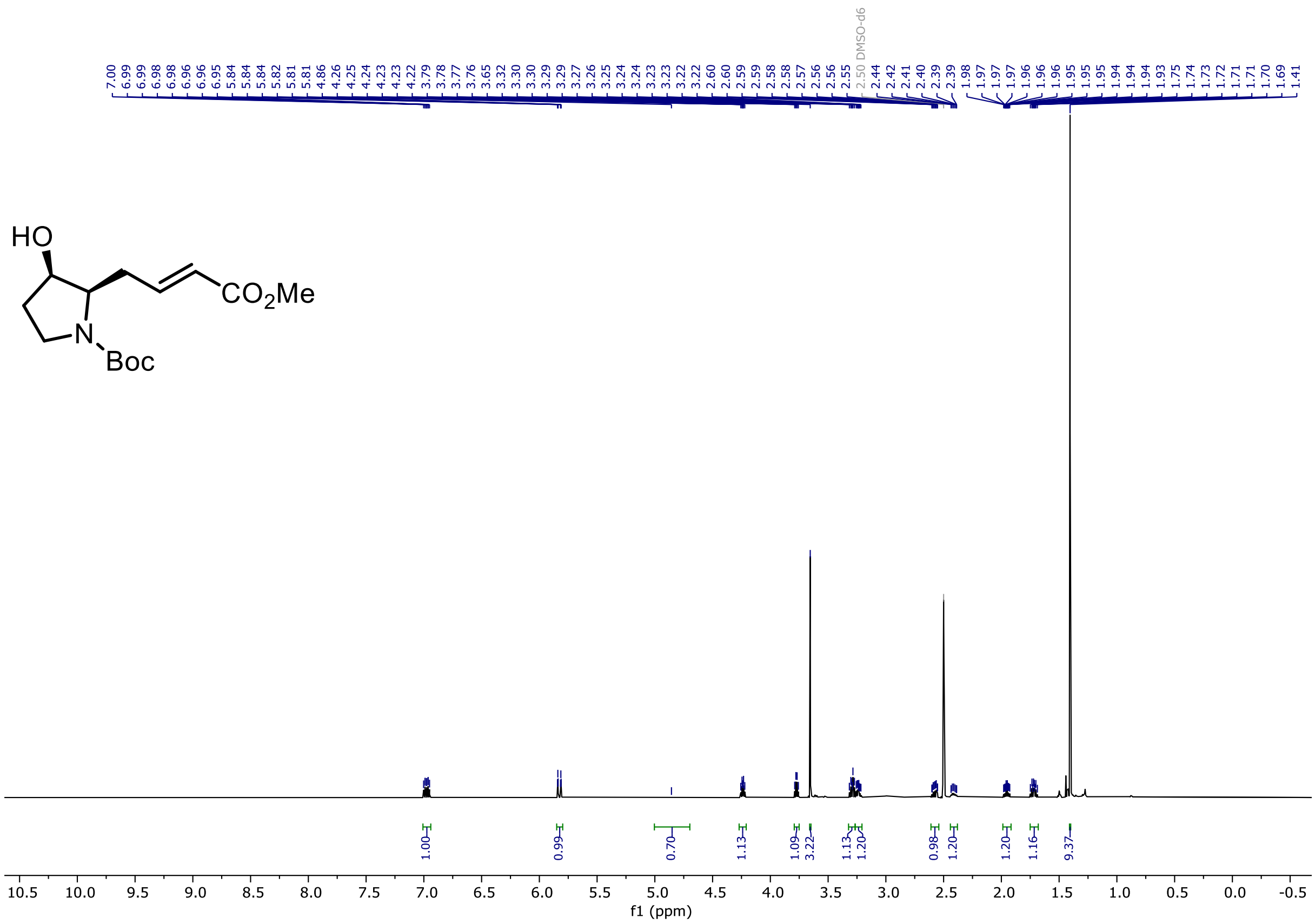

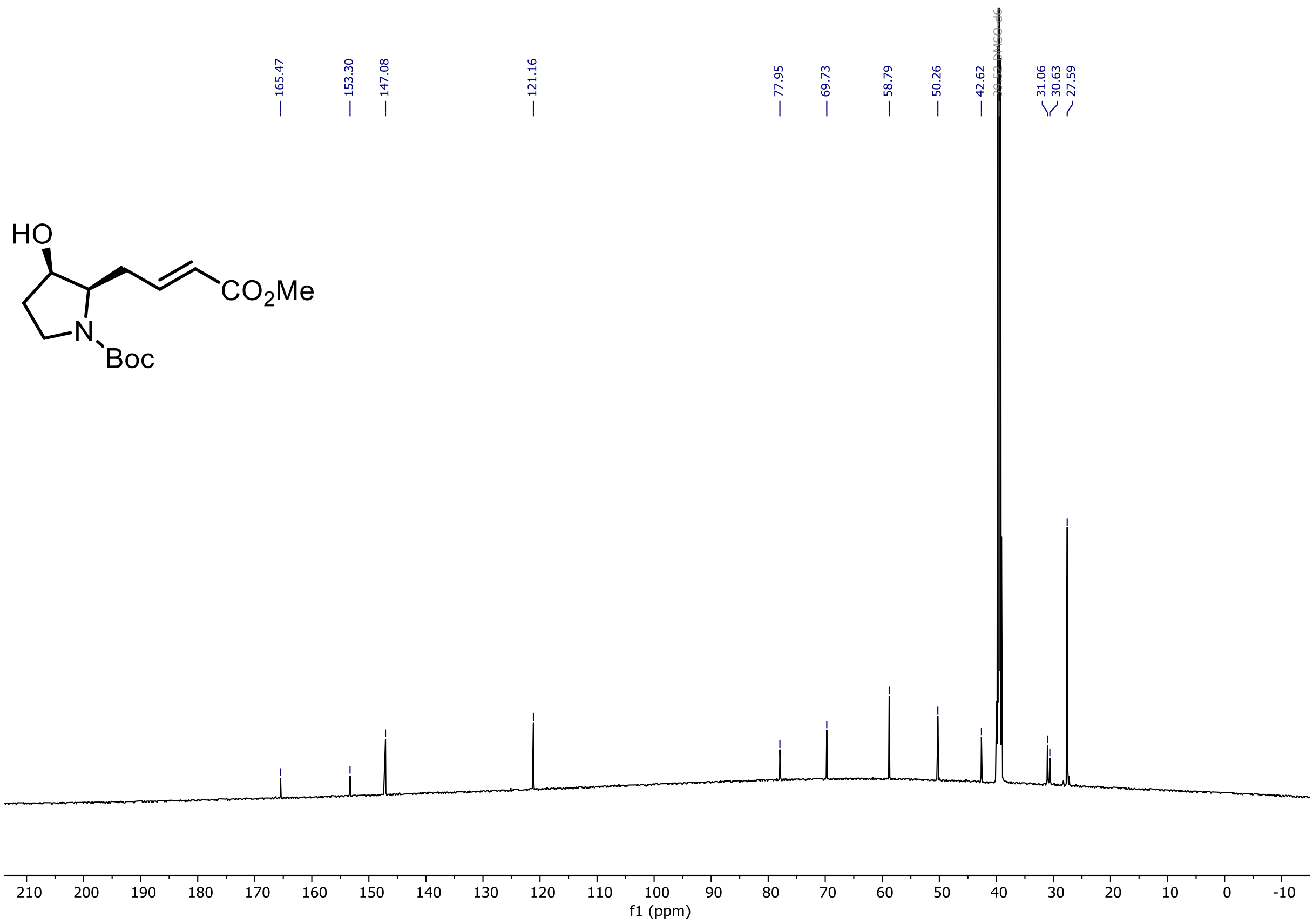

^{1}H NMR (600 MHz, [D$_6$]-DMSO, 373K; top) and ^{13}C NMR (151 MHz, [D$_6$]-DMSO, 373K; bottom) of compound **12b**

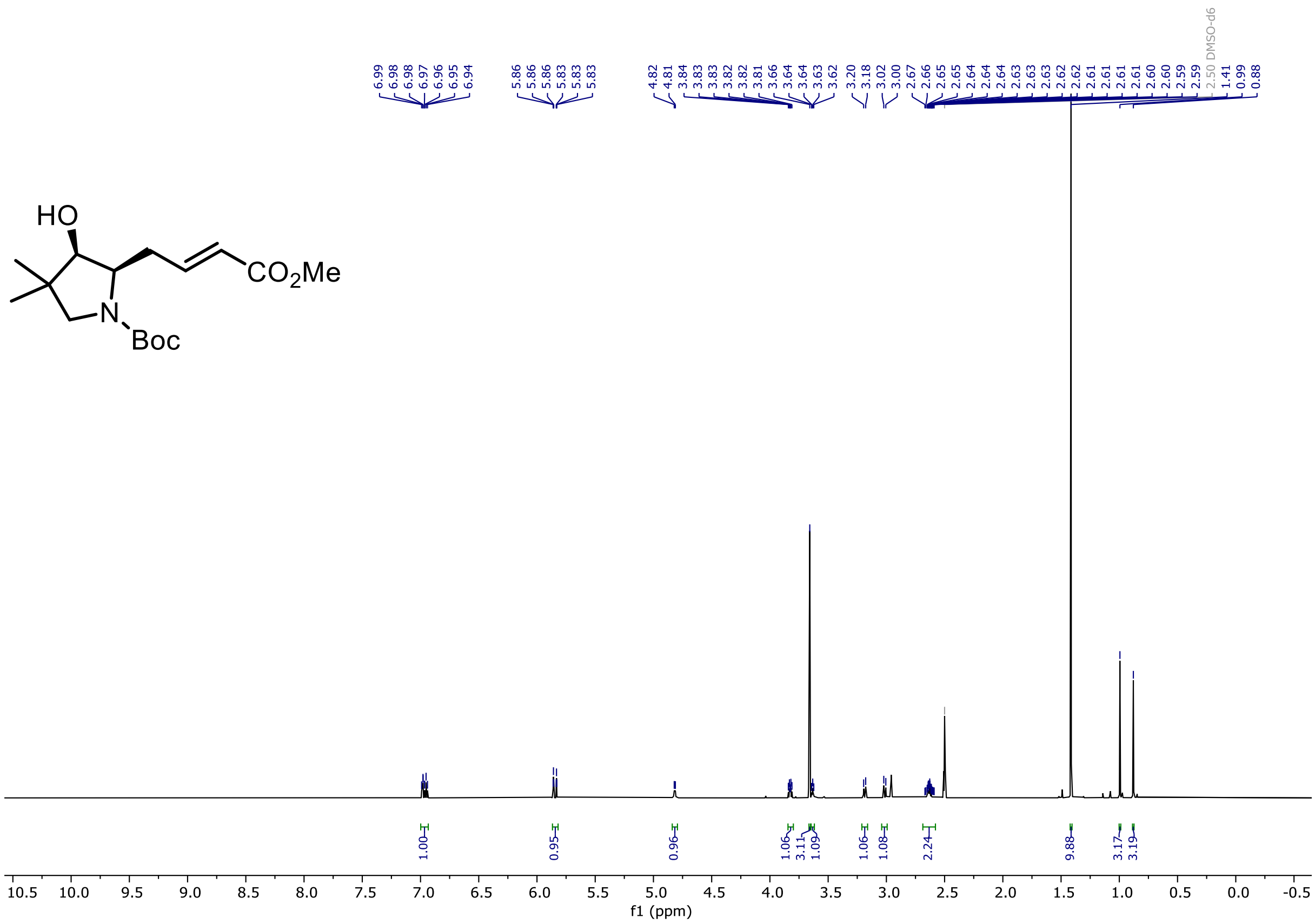

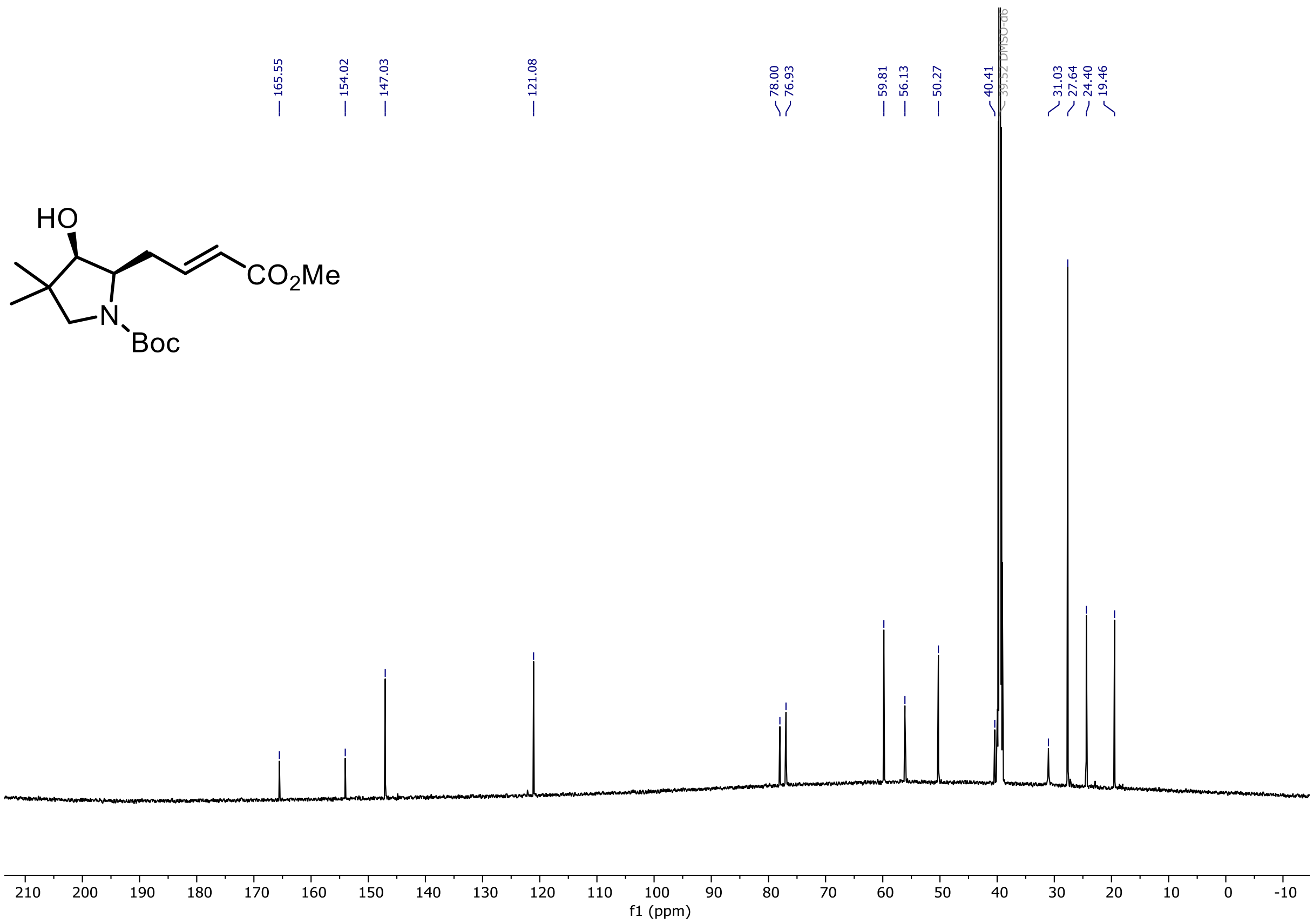

^{1}H NMR (600 MHz, [D$_6$]-DMSO, 373K; top) and ^{13}C NMR (151 MHz, [D$_6$]-DMSO, 373K; bottom) of compound **13**

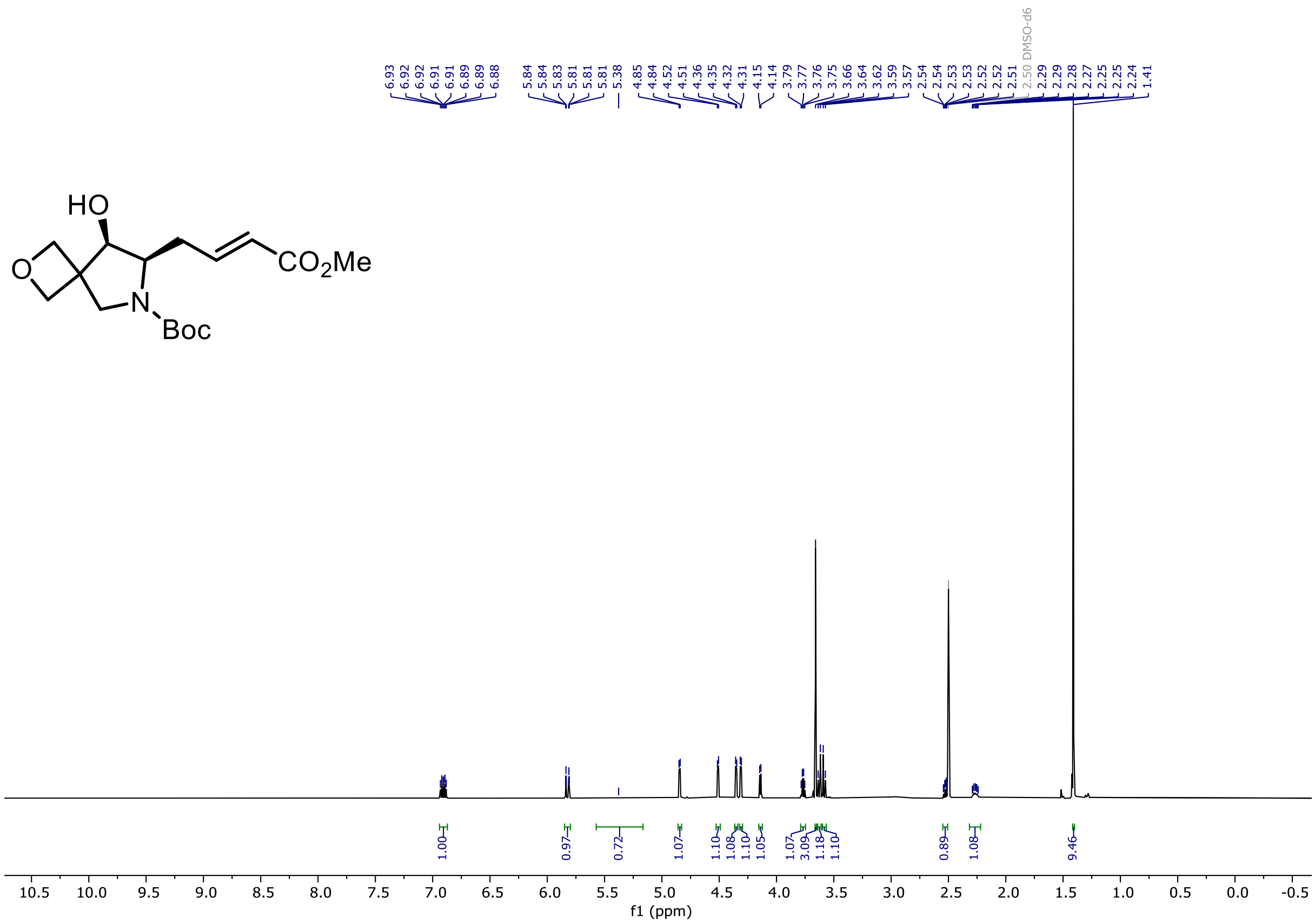

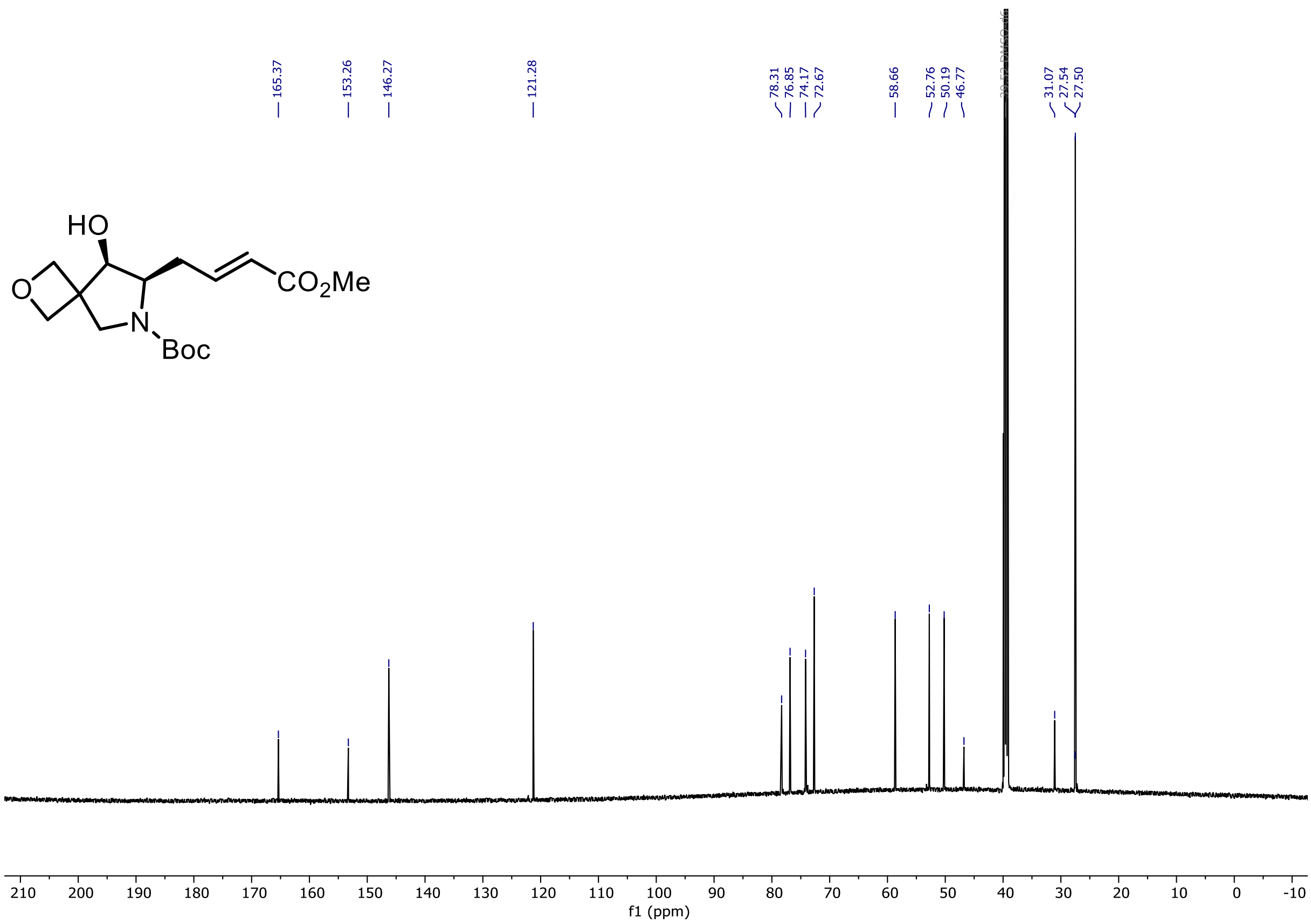

^{1}H NMR (600 MHz, [D_6]-DMSO, 373K; top) and ^{13}C NMR (151 MHz, [D_6]-DMSO, 373K; bottom) of compound **14**

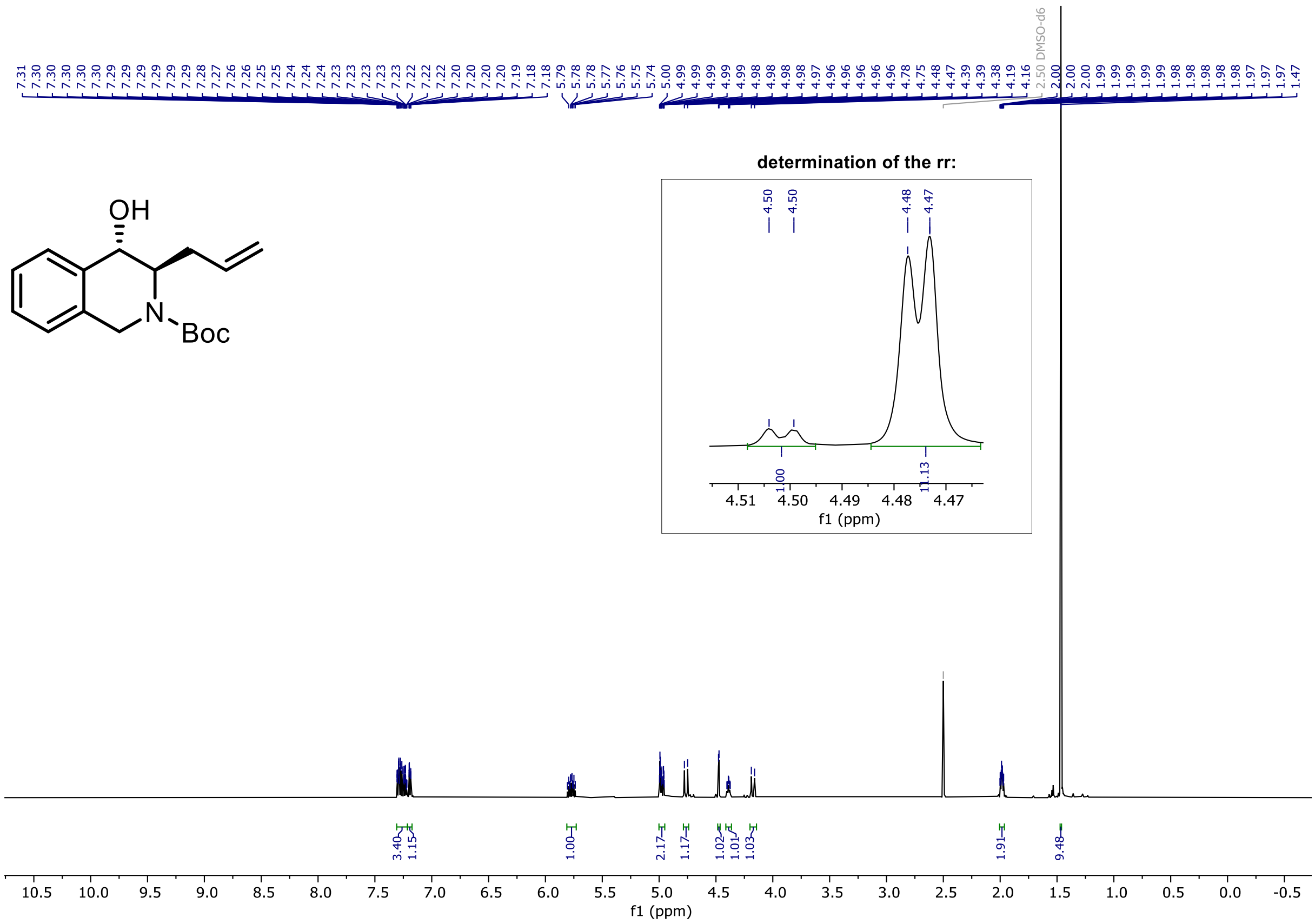

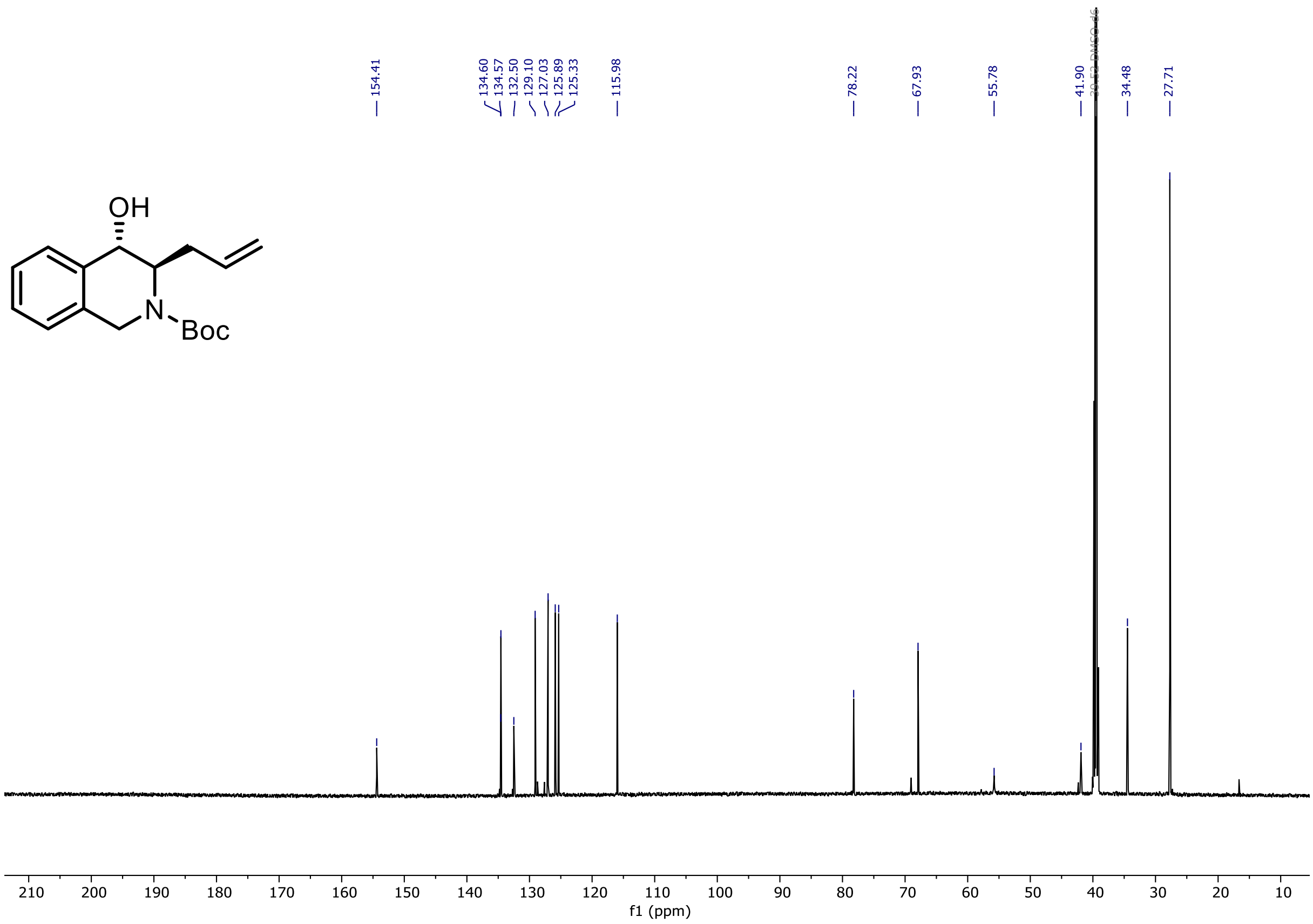

^{1}H NMR (600 MHz, [D$_6$]-DMSO, 383K; top) and ^{13}C NMR (151 MHz, [D$_6$]-DMSO, 383K; bottom) of compound **15**

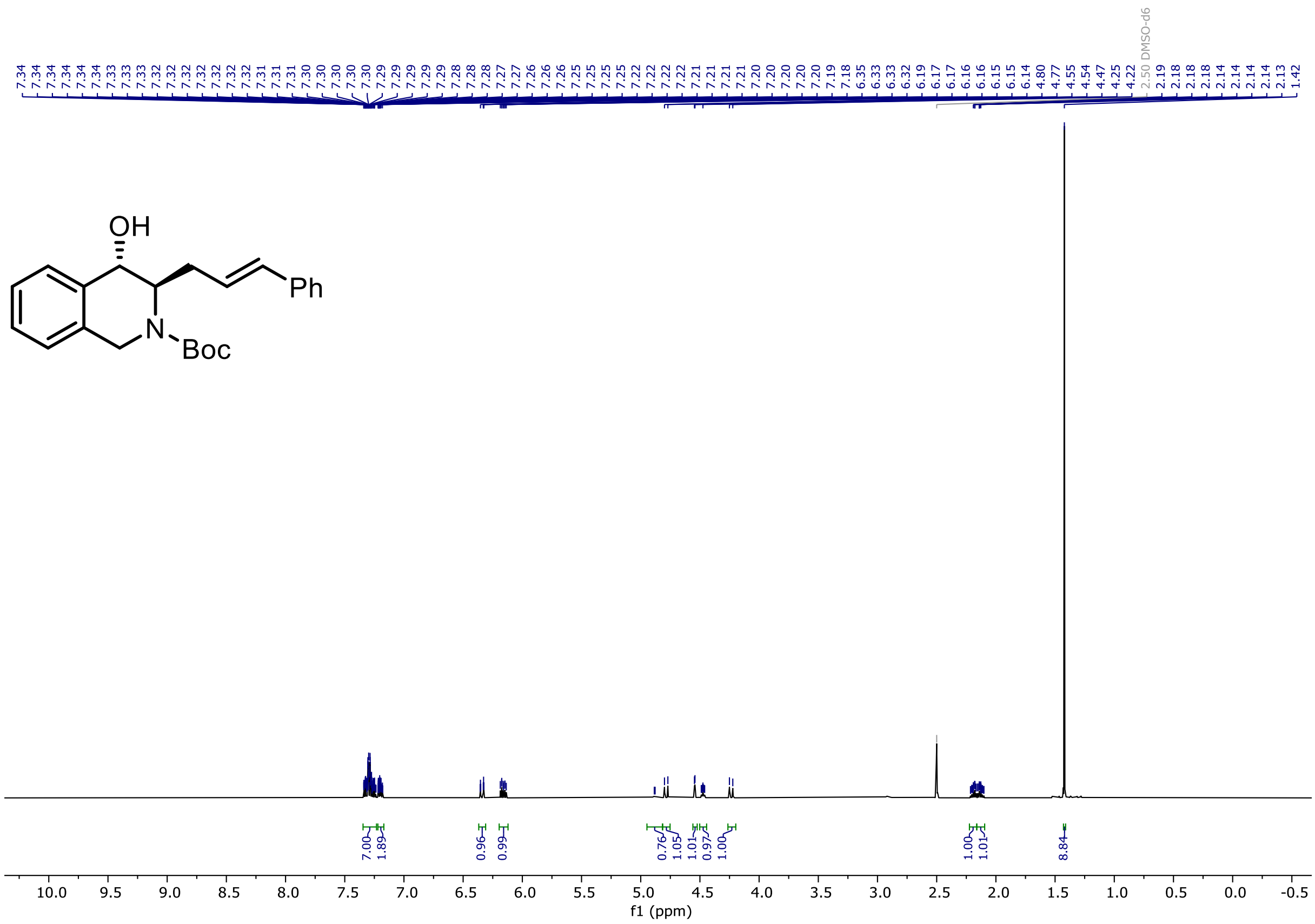

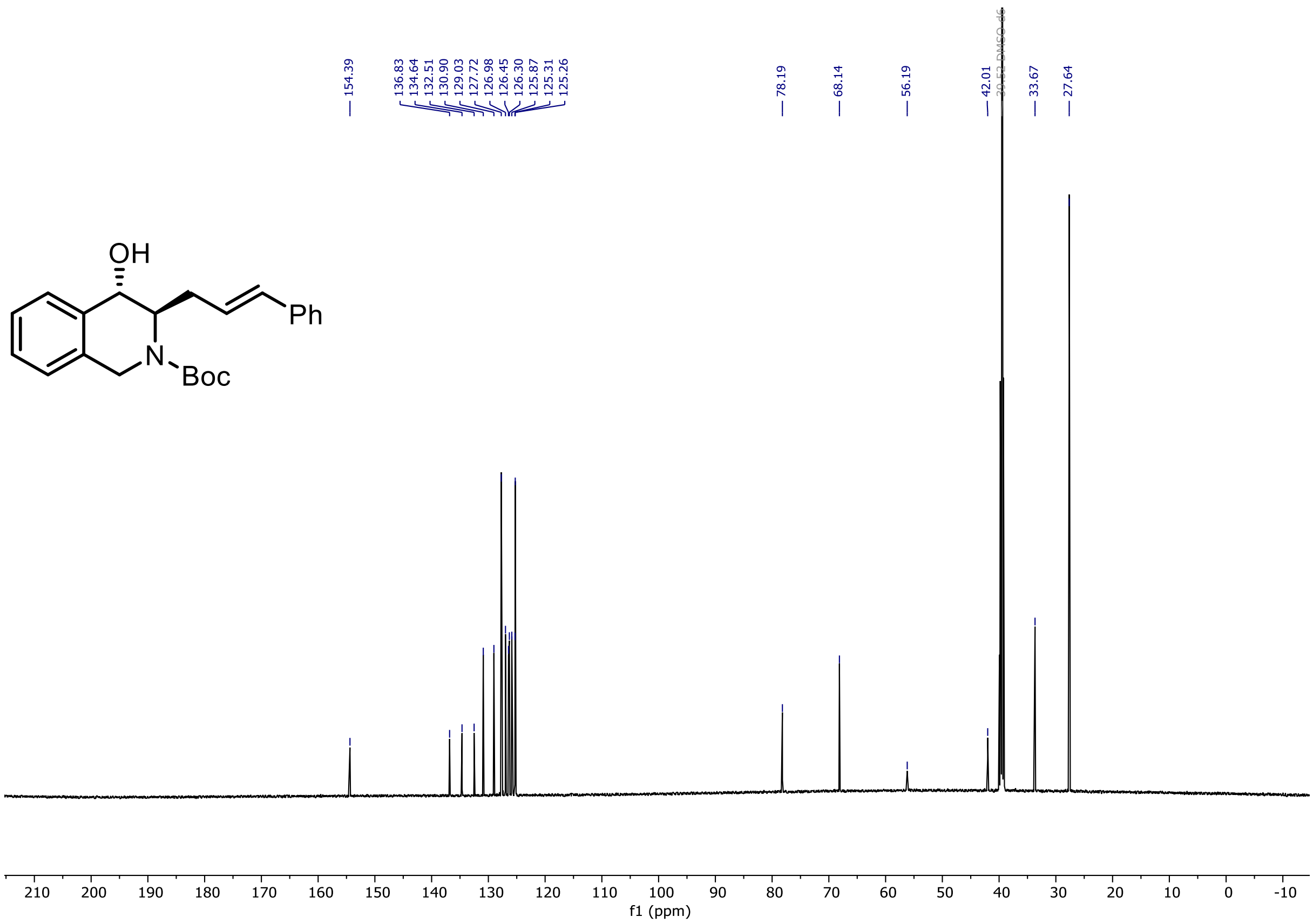

^{1}H NMR (400 MHz, $CDCl_3$; top) and ^{13}C NMR (101 MHz, $CDCl_3$; bottom) of compound **16a**

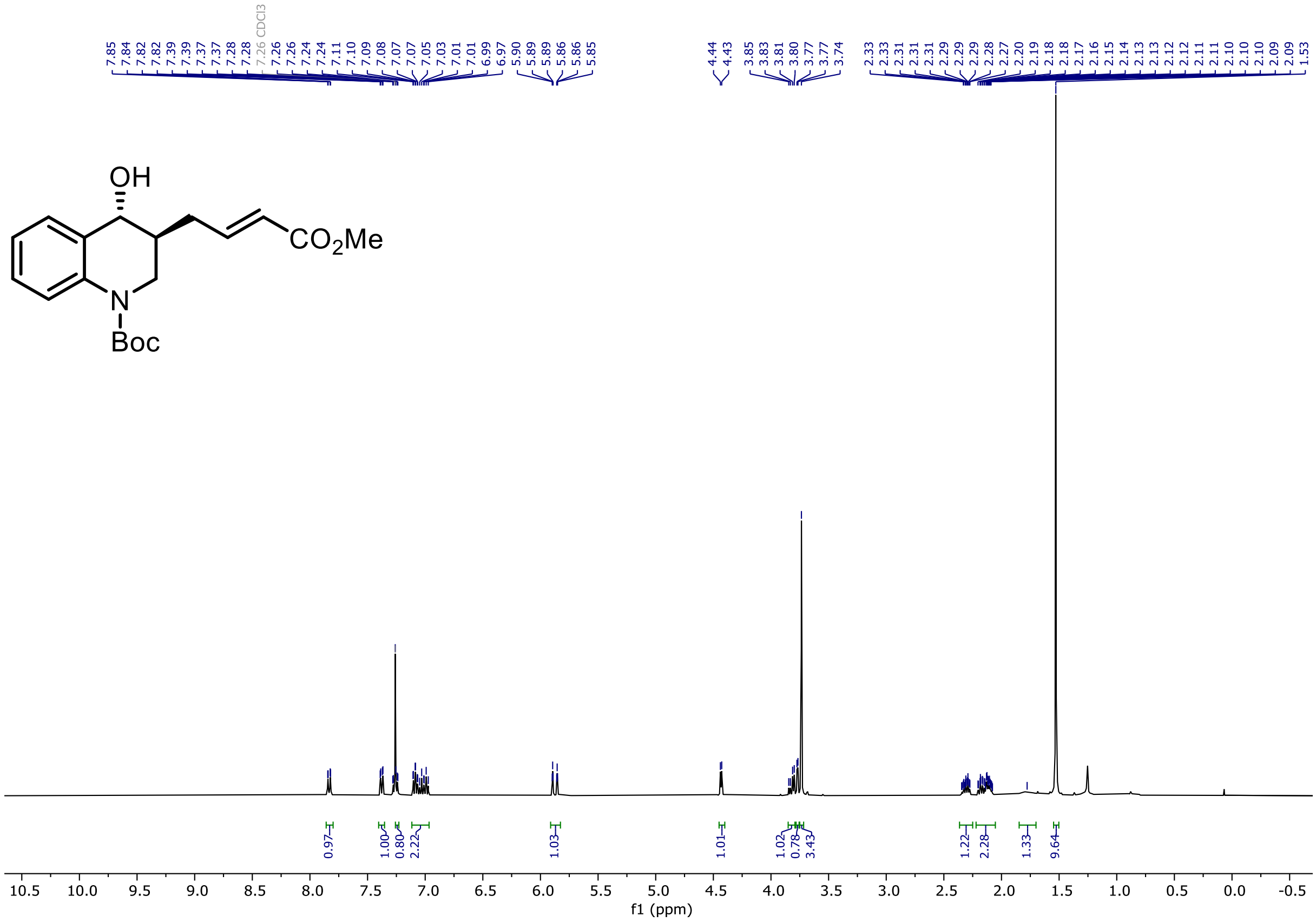

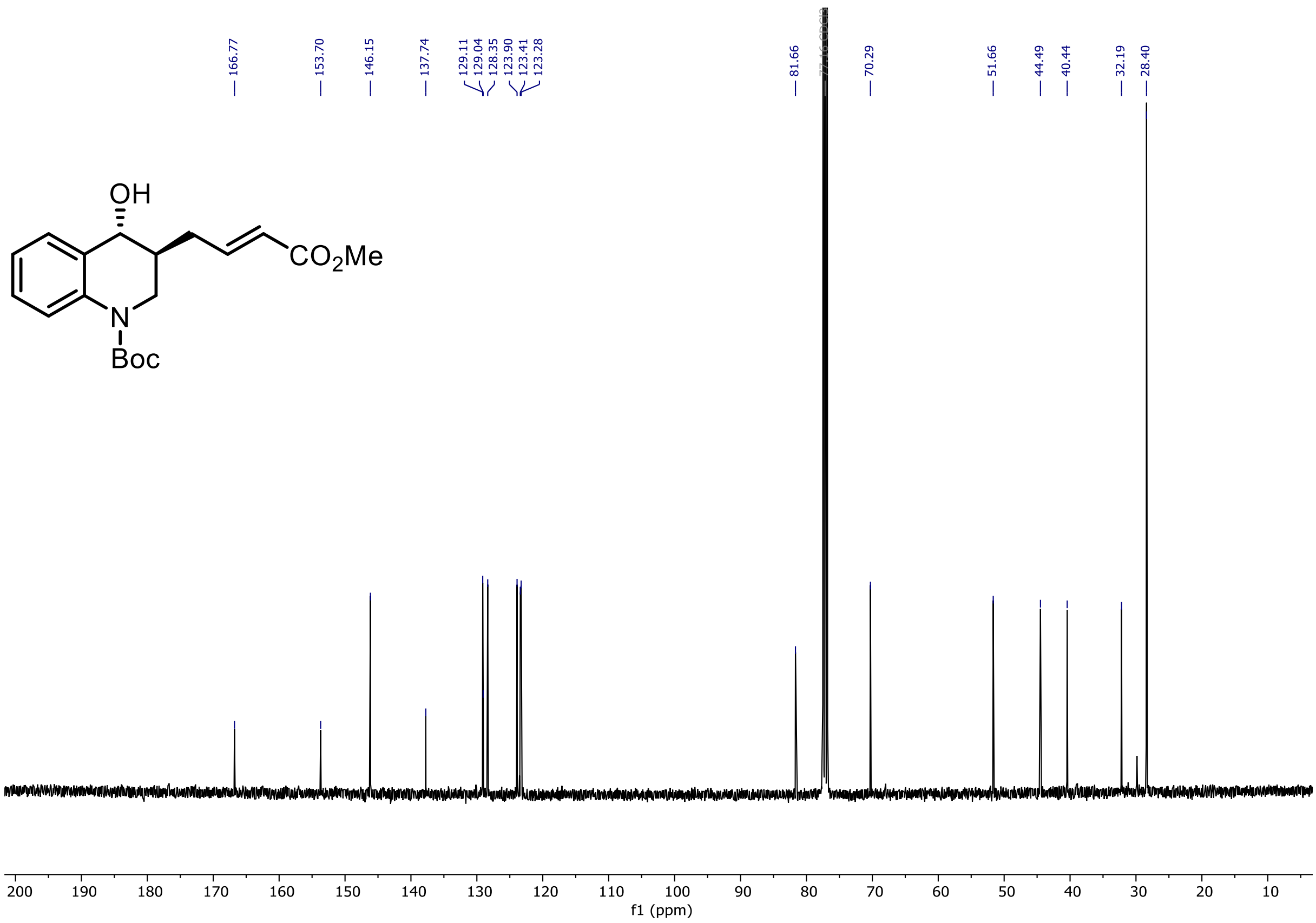

^{1}H NMR (400 MHz, $CDCl_3$; top) and ^{13}C NMR (101 MHz, $CDCl_3$; bottom) of compound **16b**

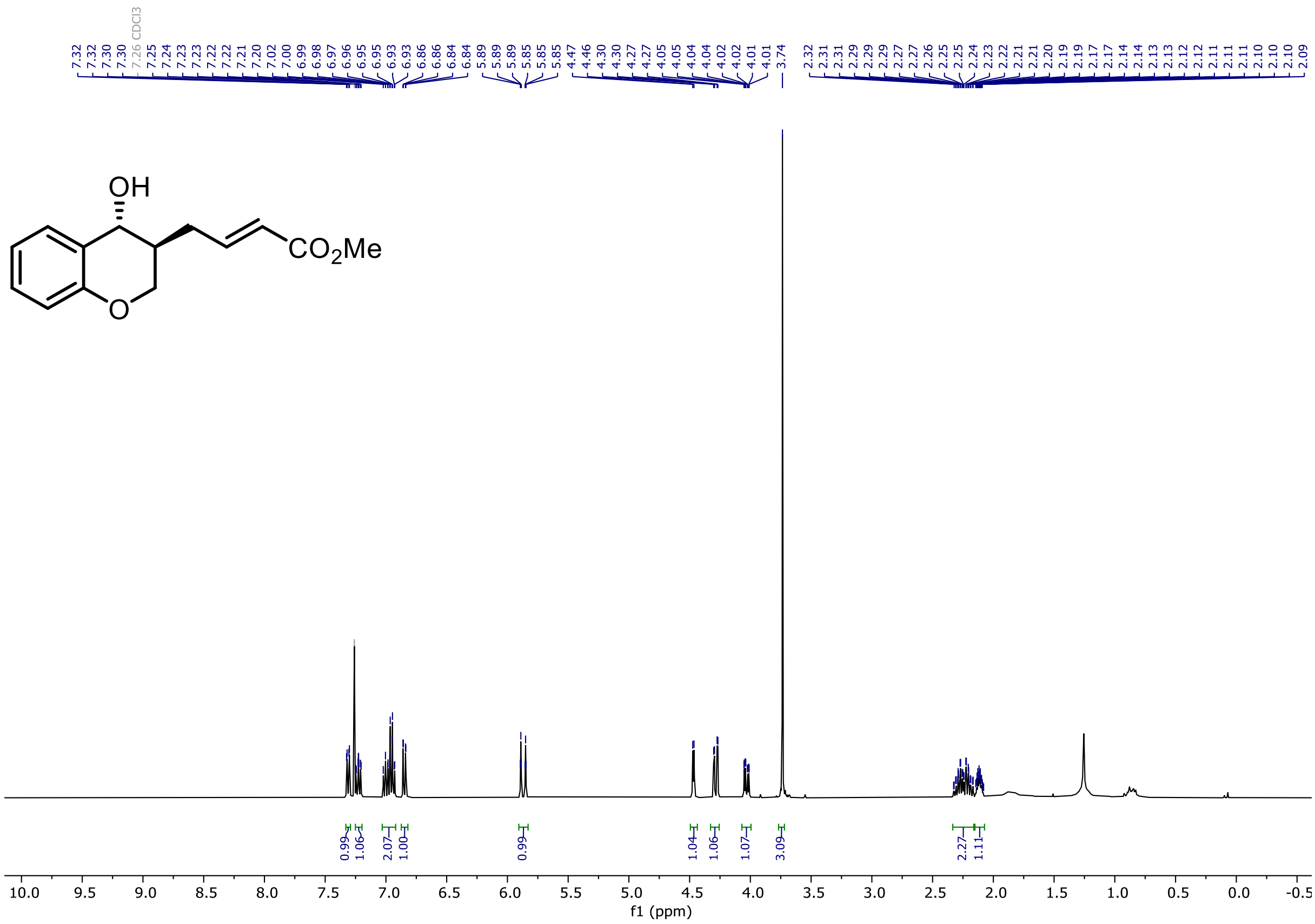

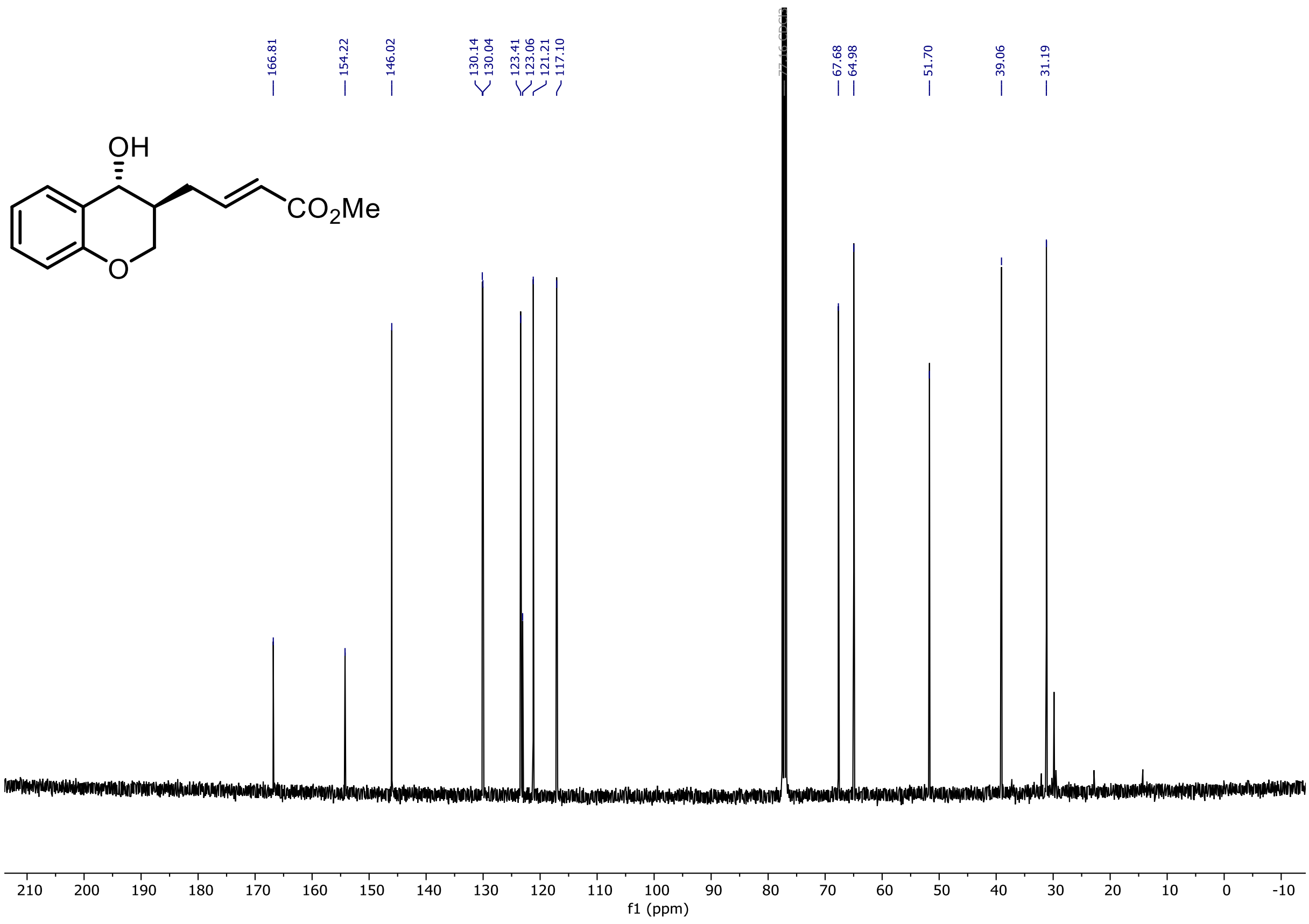

^1H NMR (600 MHz, [D_6]-DMSO, 383K; top) and ^{19}F NMR (585 MHz, [D_6]-DMSO, 383K; bottom) of compound **17**

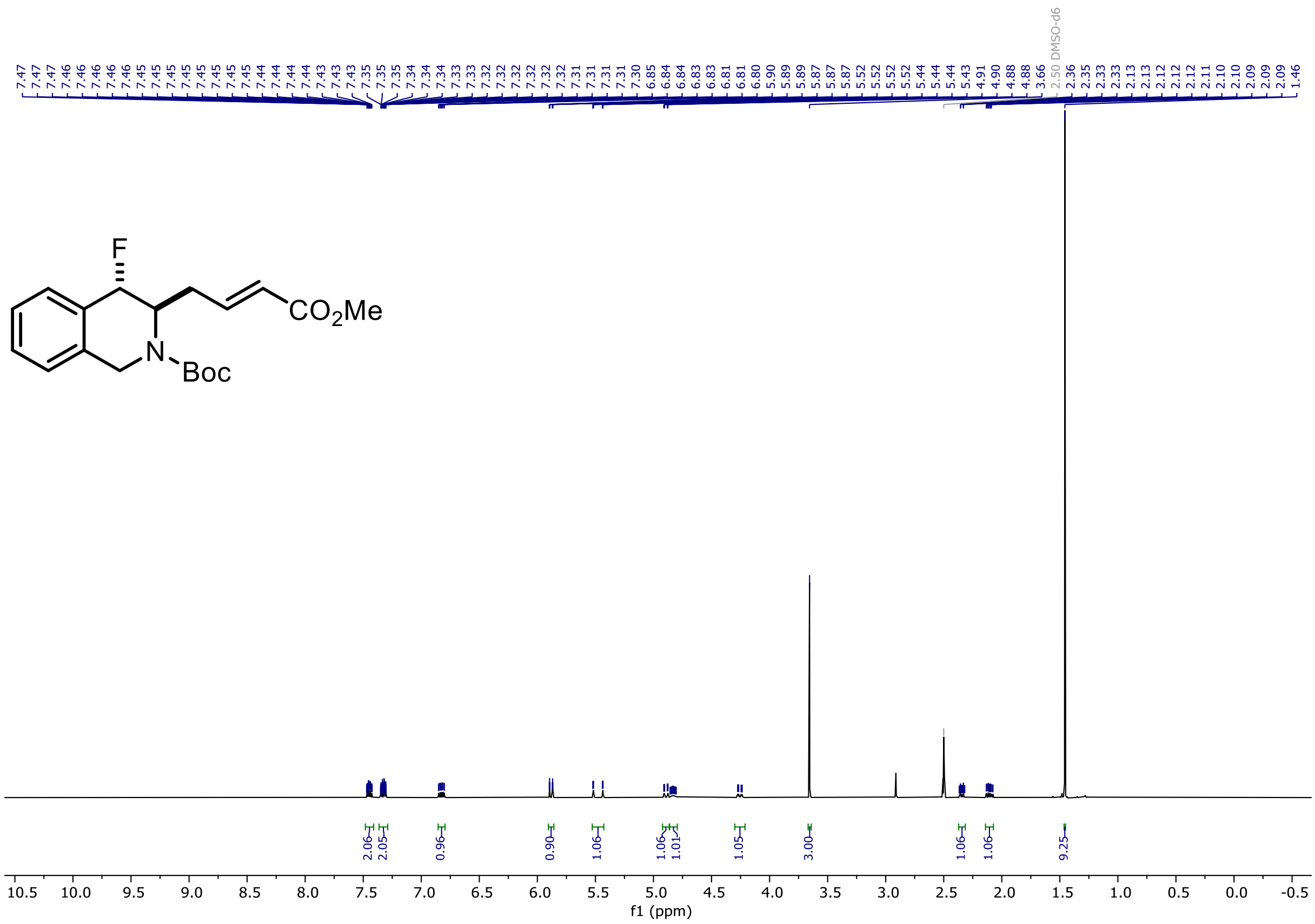

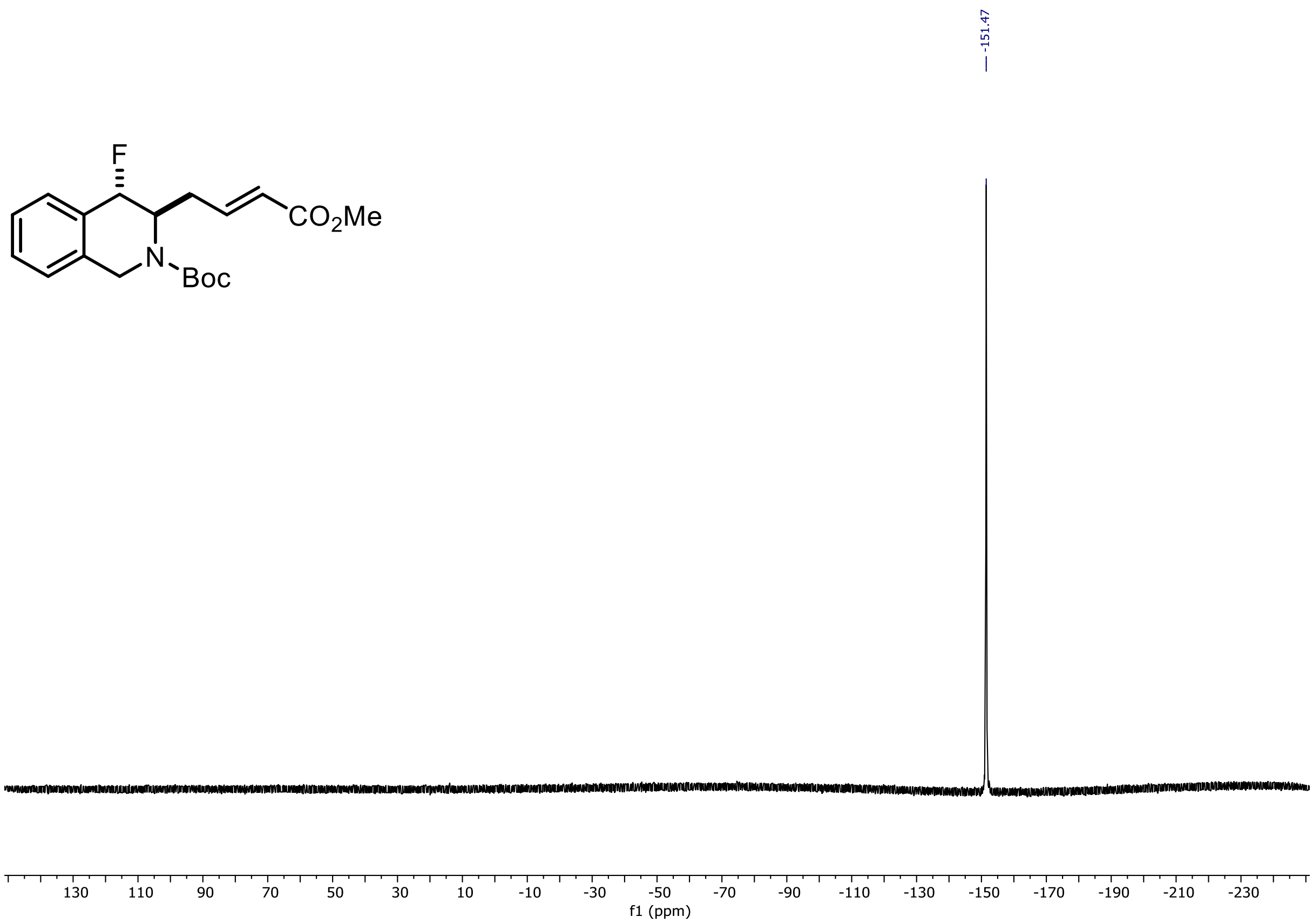

^{13}C NMR (151 MHz, [D$_6$]-DMSO, 383K) of compound **17**

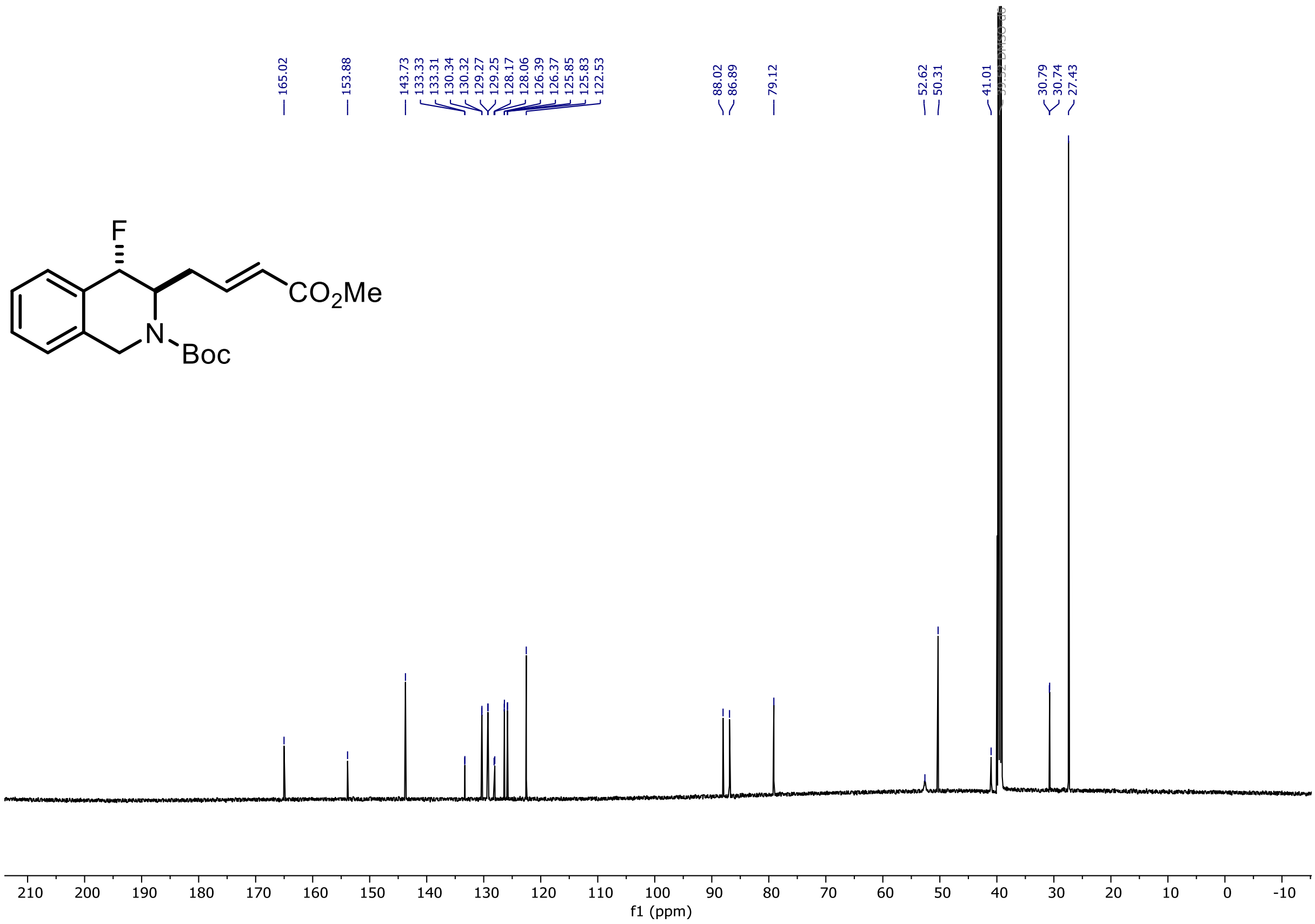

^{1}H NMR (600 MHz, [D$_6$]-DMSO, 373K; top) and ^{19}C NMR (585 MHz, [D$_6$]-DMSO, 373K; bottom) of compound **20**

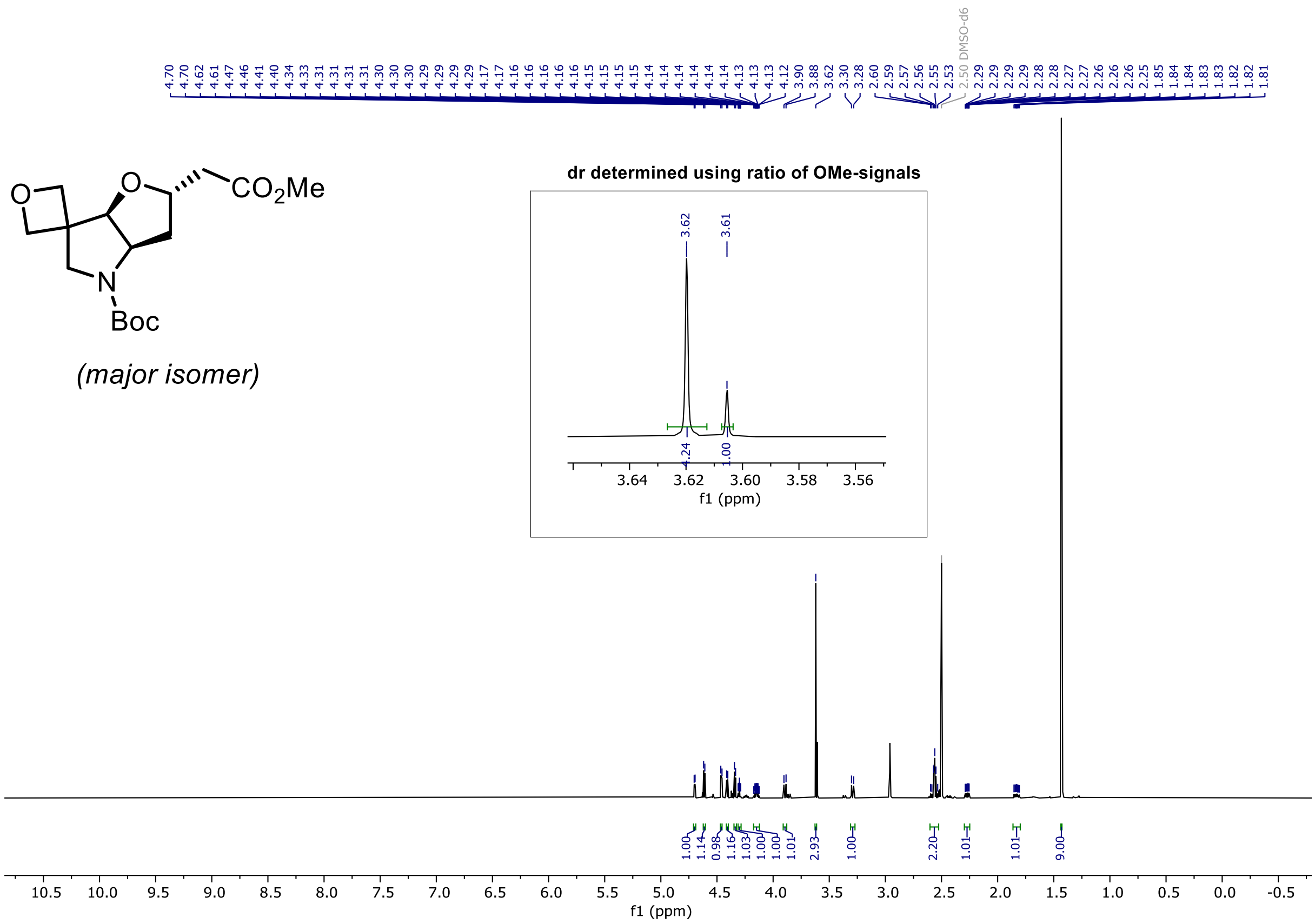

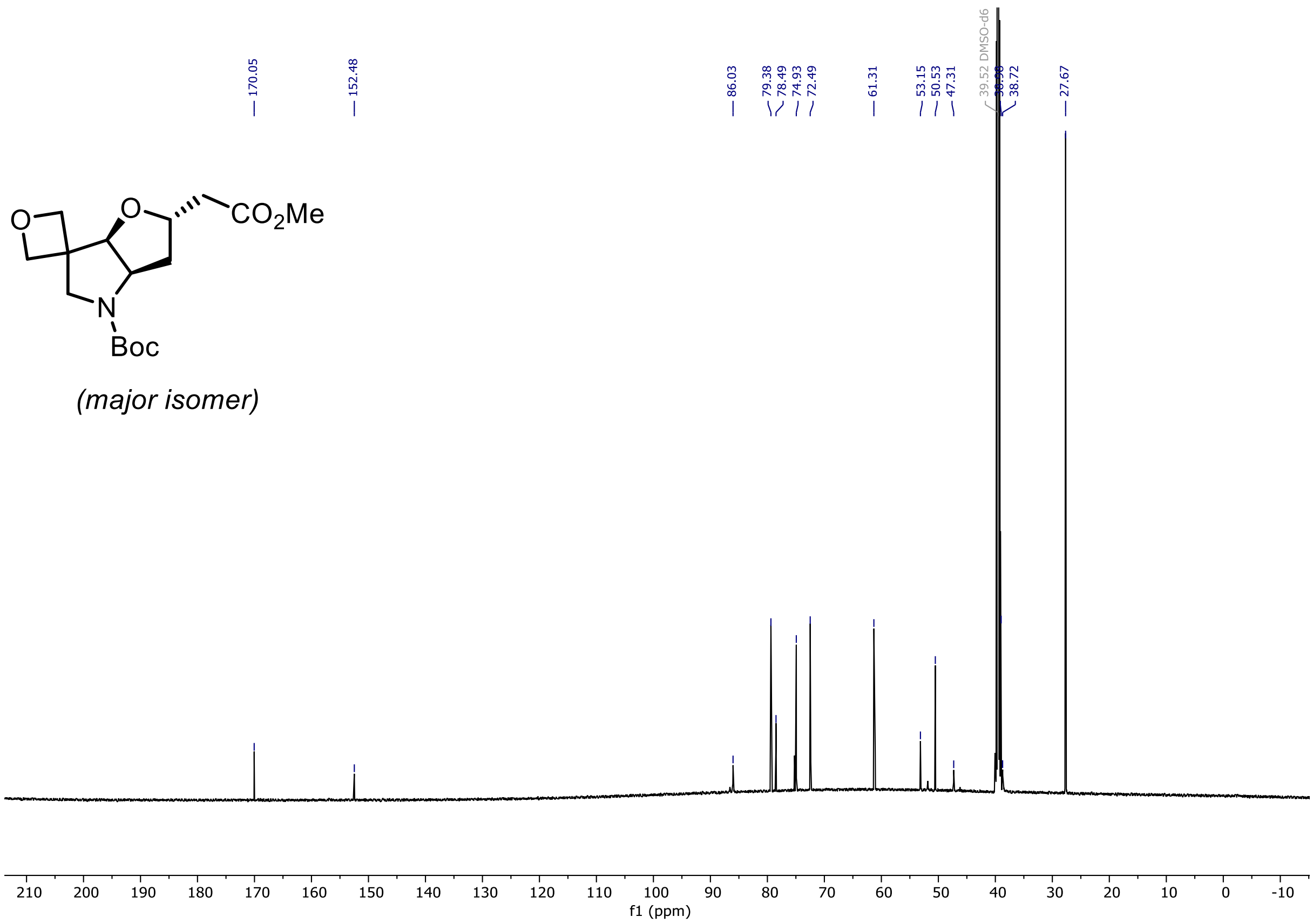

^1H NMR (400 MHz, CD_3OD; top) and ^{13}C NMR (101 MHz, CD_3OD; bottom) of compound **21**

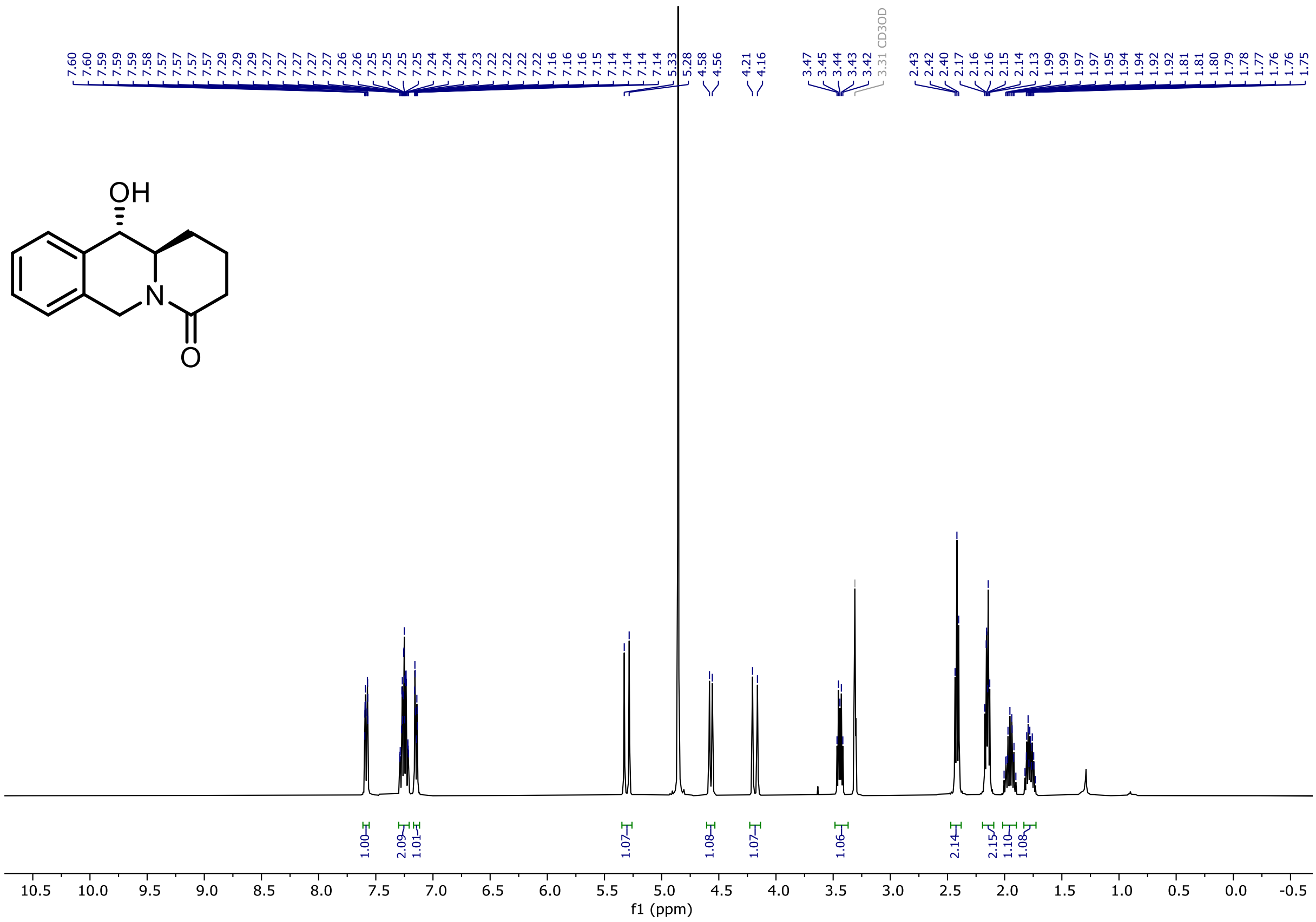

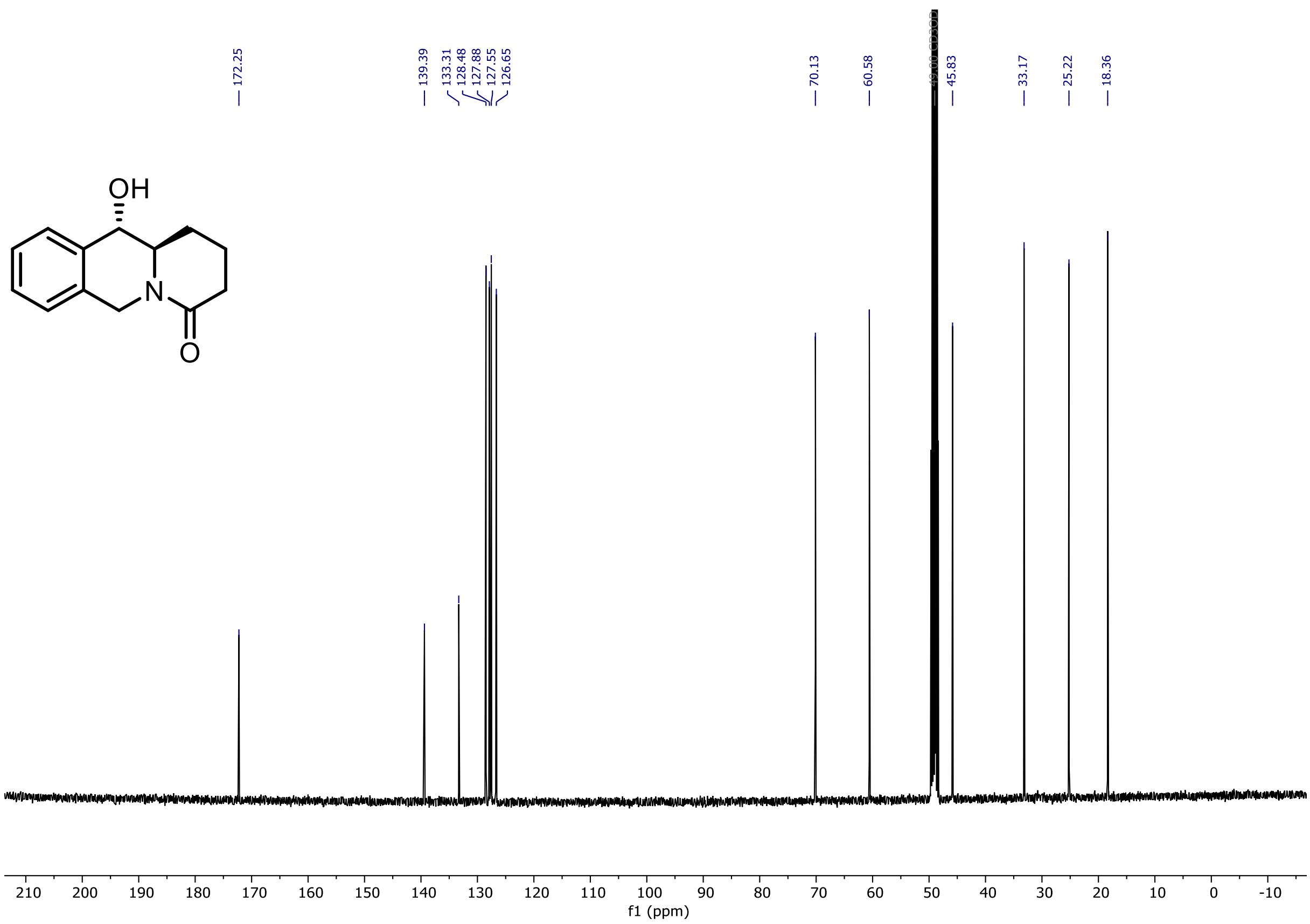

^{1}H NMR (400 MHz, CD_3OD; top) and ^{13}C NMR (101 MHz, CD_3OD; bottom) of compound **22**

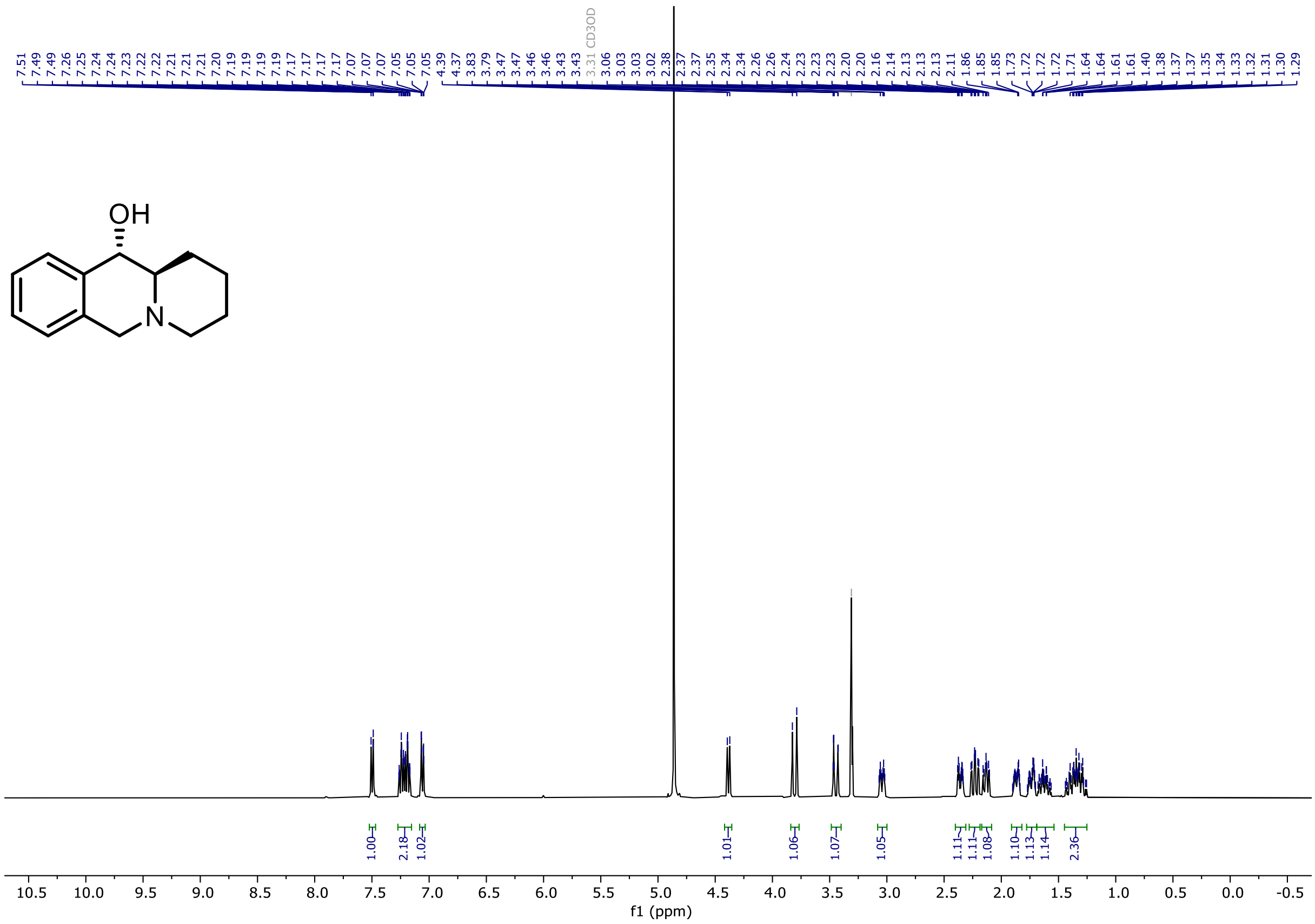

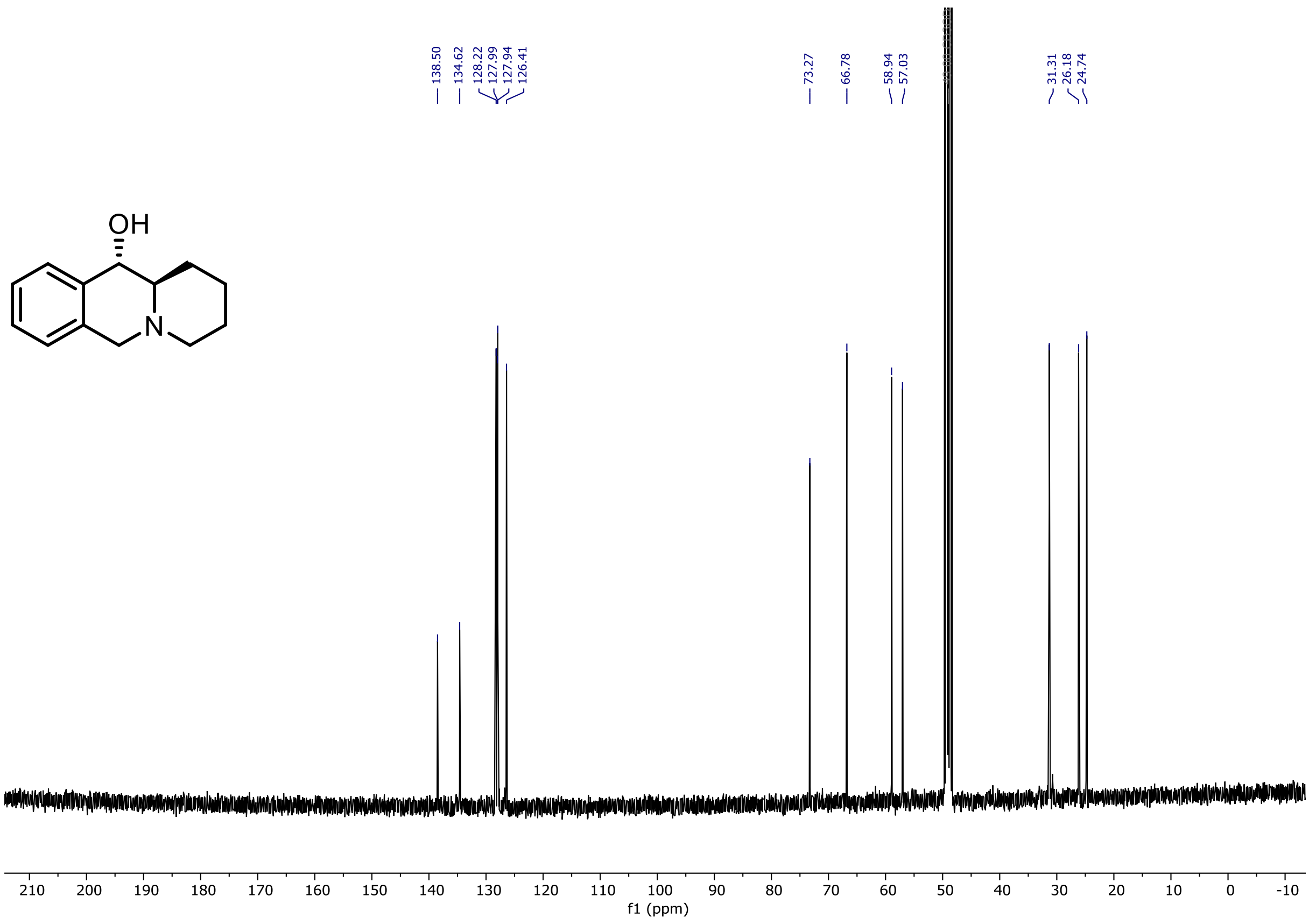

^{1}H NMR (600 MHz, CD_3OD; top) and ^{13}C NMR (151 MHz, CD_3OD; bottom) of compound **23** (dr ≈ 10:1)

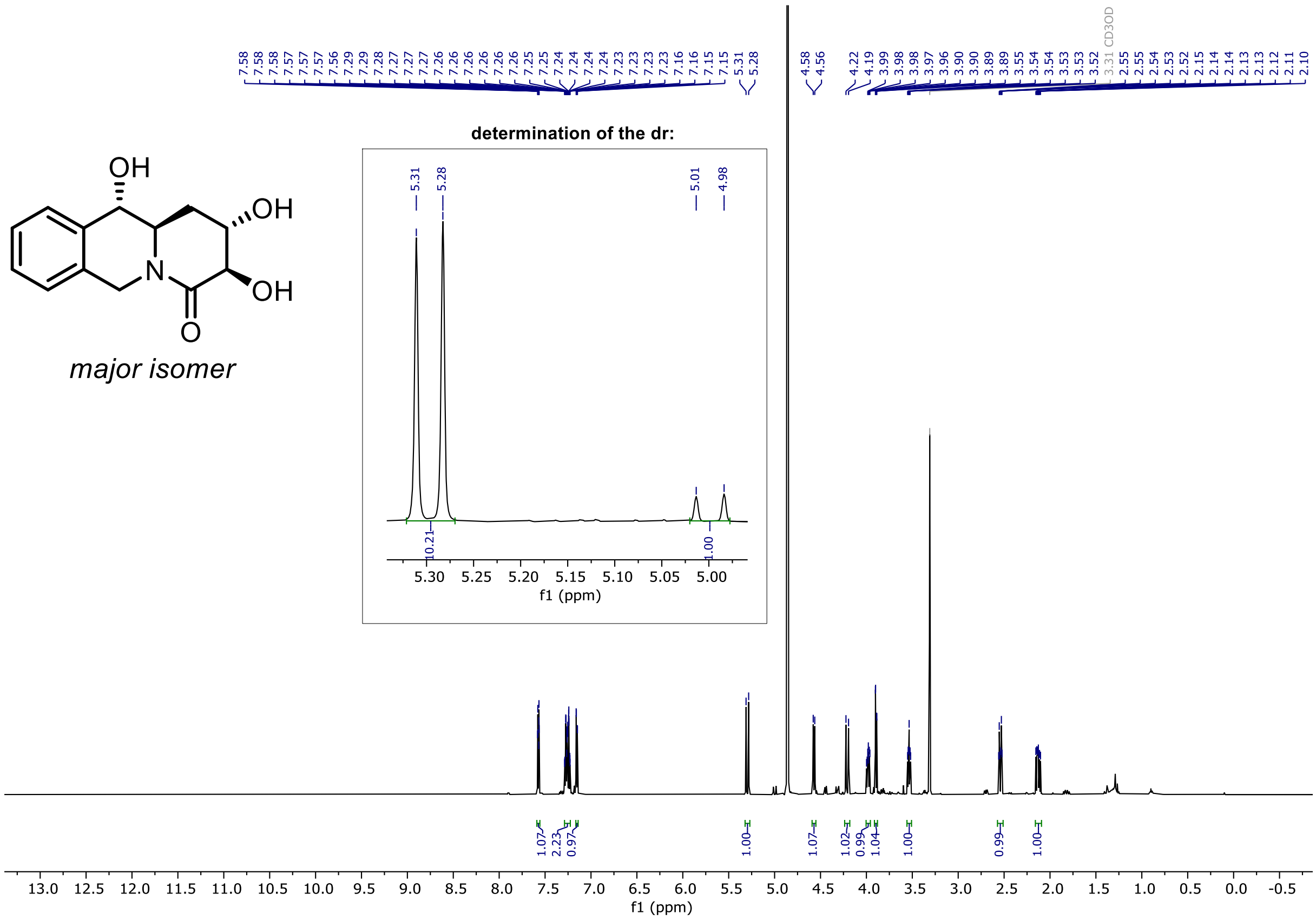

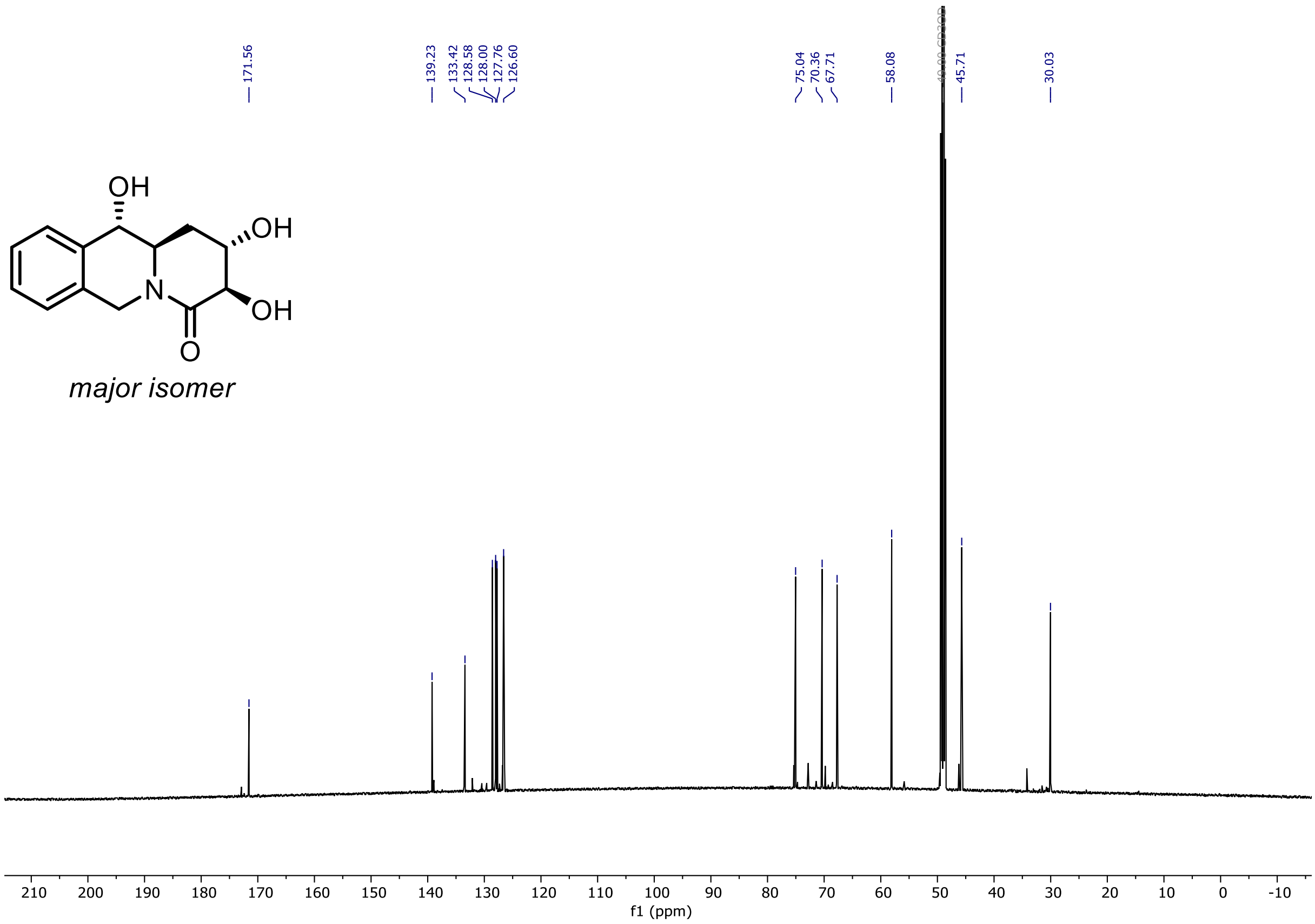

^{1}H NMR (400 MHz, CD_3OD; top) and ^{13}C NMR (101 MHz, CD_3OD; bottom) of compound **24**

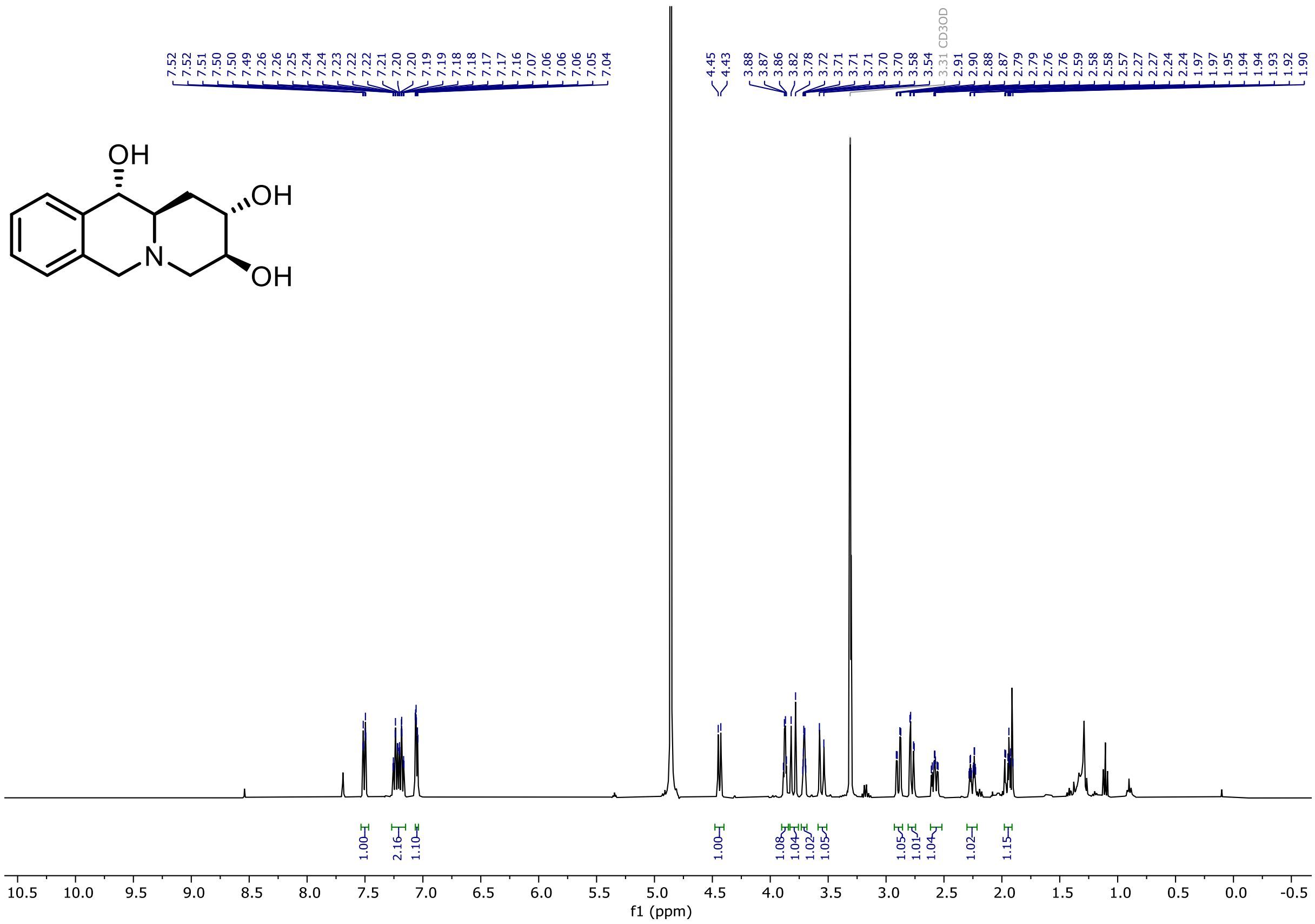

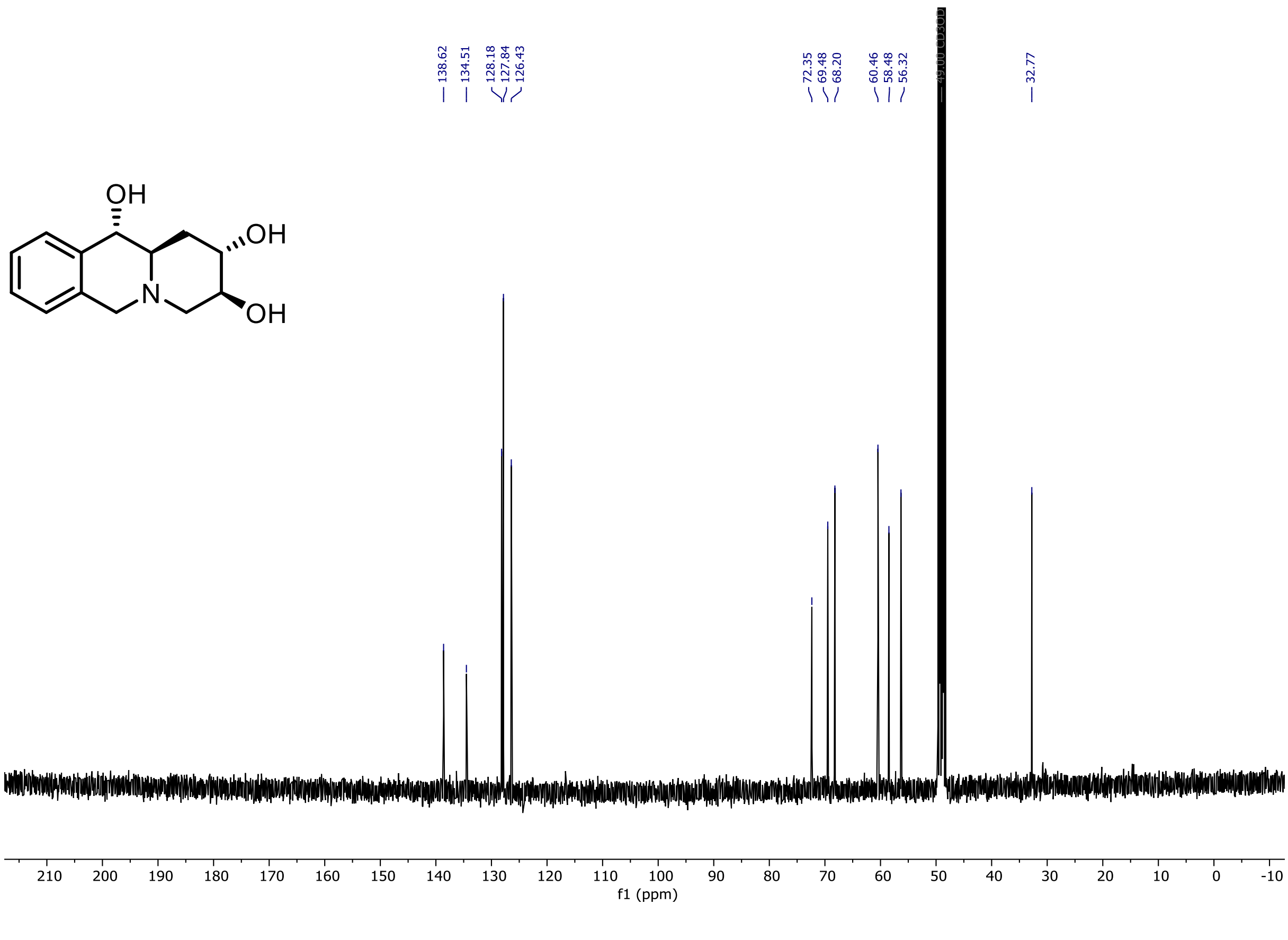

References

[1] T. Bender, A. Fürstner, *J. Am. Chem. Soc.* **2024**, *146*, 33295-33301.
[2] N. Aljaar, J. Conrad, U. Beifuss, *The Journal of Organic Chemistry* **2013**, *78*, 1045-1053.
[3] S. Wang, Y. Zhou, H. Huang, *Organic Letters* **2021**, *23*, 2125-2129.
[4] D. Janssen-Müller, S. Singha, T. Olyschläger, C. G. Daniliuc, F. Glorius, *Organic Letters* **2016**, *18*, 4444-4447.
[5] J. Jauch, *J. Prakt. Chem.* **2000**, *342*, 100-101.
[6] E. V. Litskan, S. V. Lynnyk, R. H. Shypov, B. V. Vashchenko, A. Y. Hoida, O. O. Klymenko-Ulianov, A. O. Gorlova, D. S. Granat, O. O. Grygorenko, D. M. Volochnyuk, S. V. Ryabukhin, *Chemistry – A European Journal* **2025**, *31*, e00940.
[7] S. Hitosugi, D. Tanimoto, W. Nakanishi, H. Isobe, *Chemistry Letters* **2012**, *41*, 972-973.
[8] K. R. Law, C. S. P. McErlean, *Chem. Eur. J.* **2013**, *19*, 15852-15855.
[9] T. R. Hoye, C. S. Jeffrey, F. Shao, *Nat. Protocols* **2007**, *2*, 2451-2458.
[10] S. Berger, S. Braun, 200 and More NMR Experiments, Wiley-VCH. 2004
[11] H. C. Kolb, M. S. VanNieuwenhze, K. B. Sharpless, *Chem. Rev.* **1994**, *94*, 2483-2547.
[12] Y. Sato, N. Saito, M. Mori, *J. Am. Chem. Soc.* **2000**, *122*, 2371-2372.
[13] Y. Sato, M. Takimoto, K. Hayashi, T. Katsuhara, K. Takagi, M. Mori, *J. Am. Chem. Soc.* **1994**, *116*, 9771-9772.
[14] M. D. Hanwell, D. E. Curtis, D. C. Lonie, T. Vandermeersch, E. Zurek, G. R. Hutchison, *Journal of Cheminformatics* **2012**, *4*, 17.
[15] F. Neese, *WIREs Computational Molecular Science* **2025**, *15*, e70019.
